# Comparison between Heat-Clearing Medicine and Antirheumatic Medicine in Treatment of Gastric Cancer Based on Network Pharmacology, Molecular Docking, and Tumor Immune Infiltration Analysis

**DOI:** 10.1155/2022/7490279

**Published:** 2022-01-11

**Authors:** Jiamin Xu, Fuqin Kang, Wei Wang, Shujun Liu, Jianhui Xie, Xiaobo Yang

**Affiliations:** ^1^State Key Laboratory of Dampness Syndrome of Chinese Medicine, The Second Clinical College of Guangzhou University of Chinese Medicine, Guangzhou, China; ^2^The Second Clinical College of Guangzhou University of Chinese Medicine, Guangzhou, China; ^3^Gastrointestinal Surgery, Guangdong Provincial Hospital of Chinese Medicine, Guangzhou, China; ^4^State Key Laboratory of Dampness Syndrome of Chinese Medicine, The Second Affiliated Hospital of Guangzhou University of Chinese Medicine, Guangzhou, China; ^5^Guangdong Provincial Key Laboratory of Clinical Research on Traditional Chinese Medicine Syndrome, Guangzhou, China; ^6^The Second Affiliated Hospital, Guangzhou University of Chinese Medicine, Guangzhou, China

## Abstract

**Background:**

Clinical research found that TCM is therapeutic in treating gastric cancer. Clearing heat is the most common method, while some antirheumatic medicines are widely used in treatment as well. To explore the pharmacological mechanism, we researched the comparison between heat-clearing medicine and antirheumatic medicine in treating gastric cancer.

**Methods:**

First, related ingredients and targets were searched, respectively, and are shown in an active ingredient-target network. Combining the relevant targets of gastric cancer, we constructed a PPI network and MCODE network. Then, GO and KEGG enrichment analyses were conducted. Molecular docking experiments were performed to verify the affinity of targets and ligands. Finally, we analyzed the tumor immune infiltration on gene expression, somatic CNA, and clinical outcome.

**Results:**

A total of 31 ingredients and 90 targets of heat-clearing medicine, 31 ingredients and 186 targets of antirheumatic medicine, and 12,155 targets of gastric cancer were collected. Antirheumatic medicine ranked the top in all the enrichment analyses. In the KEGG pathway, both types of medicines were related to pathways in cancer. In the KEGG map, AR, MMP2, ERBB2, and TP53 were the most crucial targets. Key targets and ligands were docked with low binding energy. Analysis of tumor immune infiltration showed that the expressions of AR and ERBB2 were correlated with the abundance of immune infiltration and made a difference in clinical outcomes.

**Conclusions:**

Quercetin is an important ingredient in both heat-clearing medicine and antirheumatic medicine. AR signaling pathway exists in both types of medicines. The mechanism of the antitumor effect in antirheumatic medicine was similar to trastuzumab, a targeted drug aimed at ERBB2. Both types of medicines were significant in tumor immune infiltration. The immunology of gastric tumor deserves further research.

## 1. Introduction

According to the data of the International Agency for Research on Cancer (IARC) [1] in 2020, there were about 1.089 million new gastric cancer cases all over the world, ranking fifth among malignant tumors. In 2020, 769 thousand people died from gastric cancer and 48.6% of death took place in China. High morbidity and mortality of gastric cancer are huge burdens to the Chinese medical system. For early-stage gastric cancer, endoscopic resection, surgery, and chemoradiotherapy are clinically recommended. However, surgery and chemoradiotherapy are not suitable for all patients with gastric cancer [2]. Tumor invasion, lymph node metastases, distant metastasis, and peritoneal implantation are not suitable for surgery. Chemoradiotherapy is not recommended to patients in poor general condition, hypoproteinemia, anemia, malnutrition, and underlying diseases [3–7]. Systemic antitumor therapy is the only option left for situations mentioned above, which generally include chemotherapeutic and molecular targeted drugs. Several trials showed that those therapeutics could not improve overall survival and had significant toxicities [8–13]. The common toxicities include neutropenia, anorexia, anemia, nausea, and vomiting. It is hard to balance the benefits and risks. What's more, due to the lack of adequate medical knowledge and regular physical examination, most gastric cancers are at an advanced stage once detected.

Recent clinical research found that traditional Chinese medicine (TCM) is therapeutic in the treatment of gastric cancer. TCM shows advantages of lowering recurrence rate, preventing adverse reactions of chemoradiotherapy, prolonging survival, and clinically strengthening immunity. Ma and Liu [14] found that formula for heat-clearing medicine and benefiting qi had a better clinical efficacy rate, Karnofsky score, quality of survival, and fewer adverse reactions, compared with DCF chemotherapy. Both Th17 and Treg cells are subsets of CD4+ T cell. Th17 secretes proinflammatory factors (IL-17), while Treg secretes anti-inflammatory factors (IL-10 and TGF-*β*). Th17, Treg cells, and the rate of Th17/Treg significantly decreased after TCM therapy, which means the mechanism of treating cancer with TCM might be related to immunity and inflammation [15, 16]. One clinical study for advanced gastric cancer found that formula for heat-clearing and dissipating phlegm had better efficacy and health status than general chemotherapy [17]. After 4-week therapy, IL-6, IL-8, TNF-*α*, and CRP remarkably reduced. It revealed that TCM therapy might be associated with anti-inflammatory and metabolic processes.

Clearing heat is a common TCM method for treating cancer. Based on theories of TCM, heat-clearing medicine is a typical type of medicine with a cool attribute and effects of clearing heat and removing toxicity. Recent pharmacological studies have shown that heat-clearing medicines could remove pathogenic microorganisms, endotoxicity, inflammation, and enhance immunity [18–20]. Besides, some medicines are broadly clinically used in both cancer and rheumatic diseases. Some latest studies found that immunity and inflammation are important features of cancer [21, 22]. Cytokines such as interleukin (IL), chemokines, and lymphocytes play a key role in tumor biology and pathology [23–25]. Tumor microenvironment (TME) consists of tumor cells, fibroblasts, immune cells, glial cells, and other acellular components [26, 27]. For example, the expression of IL-6 expression is closely related to tumor stage, metastasis, and prognosis. Higher plasma IL-6 level often clinically indicates advanced and metastasis tumor [28]. In addition, studies of IL-1*β*, IL-11, IL-17, IL-18, and TNF also indicate that proinflammatory cytokines are key regulators for TME in inhibiting tumor cell proliferation, reducing inflammation, and preventing tumor metastasis [29, 30].

Some researchers explored the ingredients and antitumor activities of *Tripterygii Radix*, *Actinidia chinensis Planch*, *Polygoni Cuspidati Rhizoma Et Radix,* and so on. In an in vitro experiment, Arora et al. found that triptolide could decrease the viability of gastric cancer cells and increase apoptosis [31]. Lei et al. isolated cisresveratrol and transresveratrol from *Polygoni Cuspidati Rhizoma Et Radix* and found tumor growth inhibition of resveratrol [32]. The extract of *Actinidia chinensis Planch* was proved to inhibit the proliferation and metastasis of gastric cancer cells [33–35]. However, no study focused on the association between the category of antirheumatic medicine and cancer. To explore this issue, we hypothesized that the antitumor mechanism of general heat-clearing medicines was different from antirheumatic herbal medicines.

Network pharmacology is a multidisciplinary method that integrates medicine, biology, and bioinformatics. This method could provide an insight into the complex mechanisms of TCM on diseases, which conforms to the multicomponent, multitarget, and multipathway of TCM. Computer-aided learning could simulate computer operation to predict the interactions between molecules and targets at a molecular level, which is widely used in pharmaceutical research. Some researchers explored the association between TCM and diabetes using network analysis and molecular docking [36]. Oh et al. investigated the bioactivities of *Zanthoxylum piperitum* fruits in treating rheumatoid arthritis based on network pharmacology [37]. Previous studies showed that computer-aided learning is an appropriate method for pharmaceutical research.

**Figure 1:**
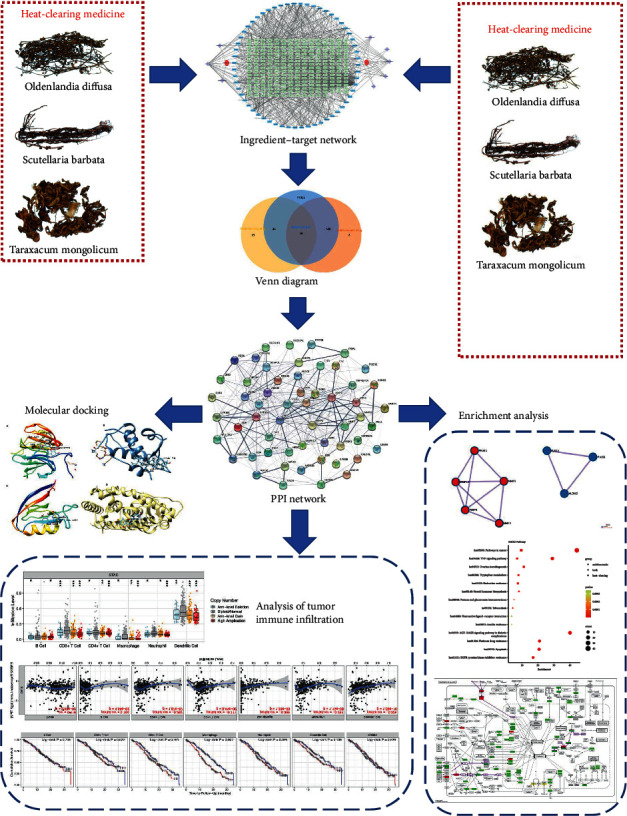
Schematic flow diagram: searching medicines, constructing ingredient-target network, Venn diagram, PPI network, enrichment analysis, molecular docking, and analysis of tumor immune infiltration.

Therefore, we did research based on network pharmacology to find the key ingredients and targets. Then molecular docking experiment was performed to validate the interaction activity of key proteins and ligands. Finally, to verify the association of immunity and gastric cancer, analysis of tumor immune infiltration was operated based on The Cancer Genome Atlas (TCGA). The flow diagram is shown in Figure 1.

## 2. Method

### 2.1. Searching and Screening Related Ingredients and Targets

According to the classification of Chinese materia medica, representative medicine of gastric cancer was selected in this research. *Oldenlandia diffusa* (Chinese Pinyin name: Baihuasheshecao), *Scutellaria barbata* (Chinese Pinyin name: Ban zhi lian), and *Taraxacum mongolicum* (Chinese Pinyin name: Pugongying) represented the commonly used heat-clearing medicine. The typical antirheumatic herbal medicines were *Rabdosia rubescens* (Chinese Pinyin name: Dong ling cao), *Duchesnea indica* (Chinese Pinyin name: Shemei), *Smilax glabra* (Chinese Pinyin name: Tufuling), *Akebia quinata* (Chinese Pinyin name: Yuzhizi), and *Actinidia chinensis Planch* (Chinese Pinyin name: Tengligen). The ingredients of herbal medicines mentioned above were searched through the traditional Chinese medicine systems pharmacology database and analysis platform (TCMSP database https://old.tcmsp-e.com/tcmsp.php) and anticancer herbs database of systems pharmacology (CancerHSP database https://old.tcmsp-e.com/CancerHSP.php) [38]. Two ADME values including oral availability (OB) ≥30% and drug likeness (DL) ≥0.18 were screened for eligible ingredients. We also searched related ingredients from the PubMed database according to relative articles. According to ingredients collected before, we obtained targets from the TCMSP database, CancerHSP database, and relative articles in the same way mentioned above. To standardize the information of targets, we unified all the included targets based on the UniProt protein database (https://www.uniprot.org) [39].

### 2.2. Constructing an Active Ingredient-Target Network

The active ingredient-target network of heat-clearing medicine and antirheumatic medicine was constructed and analyzed through Cytoscape 3.8.1 [40]. Nodes denoted ingredients or targets. Edges represented that some ingredients could activate or inhibit the connected targets, according to previous studies. The Network Analyzer tool in Cytoscape calculated the characteristic parameters in a network, including degree, betweenness, and closeness.

### 2.3. Searching and Screening Related Targets of Gastric Cancer

To ensure that relevant targets of gastric cancer were comprehensive in this study, we searched in four gene databases, including GeneCards database [41] (https://www.genecards.org), OMIM database [42] (https://www.omim.org), TTD database [43] (https://bidd.nus.edu.sg/group/cjttd), and DrugBank database [44] (https://www.drugbank.ca). Relevant targets were downloaded from databases and integrated as a dataset about gastric cancer.

### 2.4. Constructing PPI Network

To clarify the relationship between heat-clearing medicine, antirheumatic medicine, and gastric cancer, a protein-protein interaction (PPI) network was constructed in the STRING 11.0 database [45, 46] (https://string-db.org). Biological species were limited to Homo sapiens, and the minimum interaction threshold was set as the highest confidence (>0.9). After constructing the PPI network, the potential protein functional modules and biological processes were analyzed by applying the molecular complex detection (MCODE) algorithm [47]. Using the MCODE plugin and its default parameters in Cytoscape software, the MCODE algorithm could identify neighborhoods of densely connected proteins by clustering and classifying proteins.

### 2.5. Enrichment Analysis

Gene Ontology (GO) [48] enrichment and Kyoto Encyclopedia of Genes and Genomes (KEGG) [49] enrichment were analyzed in Metascape [50] (https://metascape.org). Molecular function, biological process, cellular component, and KEGG pathway were analyzed and selected with a *p*-value <0.01, a minimum count of 3, and an enrichment factor >1.5. The results were visualized in R software.

### 2.6. Molecular Docking

The three-dimensional structure of ligands was built in ChemOffice [51] software and saved in MOL2 format of minimized energy. The 3D structures of targets were downloaded from Protein Data Bank (PDB) [52] (https://www.rcsb.org/). PyMOL software [53] was used to remove water and add hydrogen to the target. We performed blind docking of ligands and targets in AutoDock Vina software [54]. The three coordinates of the grid box were adjusted to enclose the whole protein. AutoDock Vina calculated all the possible binding residues and presented the parameter containing residues, binding energy, cluster, and so on.

### 2.7. Analysis of Tumor Immune Infiltration

In the TIMER database [55] (https://cistrome.shinyapps.io/timer/), the deconvolution algorithm could estimate the correlation between gene expression and abundances of six immune infiltrates (B cells, CD4+ T cells, CD8+ T cells, neutrophils, macrophages, and dendritic cells). GISTIC 2.0 [56, 57] could identify regions of the genome that are significantly amplified or deleted across a set of samples. In this study, the association between immune infiltrates and somatic copy number alterations (SCNAs) of a gene across TCGA cancer types was evaluated in GISTIC 2.0. Based on The Cancer Genome Atlas (TCGA) database [58] (https://portal.gdc.cancer.gov), prediction of clinical outcome and abundance of immune infiltrates were calculated in a multivariable Cox proportional hazard model.

## 3. Result

### 3.1. Active Ingredients and Relevant Targets of Medicines

Ingredients were selected based on oral availability and drug likeness for good absorption, distribution, metabolism, and excretion in vivo. A total of 31 active ingredients of heat-clearing medicine and 31 active ingredients of antirheumatic medicine were collected from the TCMSP database, CancerHSP database, and related articles (Table 1). There were 90 relevant targets of heat-clearing medicine and 186 relevant targets of antirheumatic medicine (Supplementary Table 1).

### 3.2. Ingredient-Target Network

**Figure 2:**
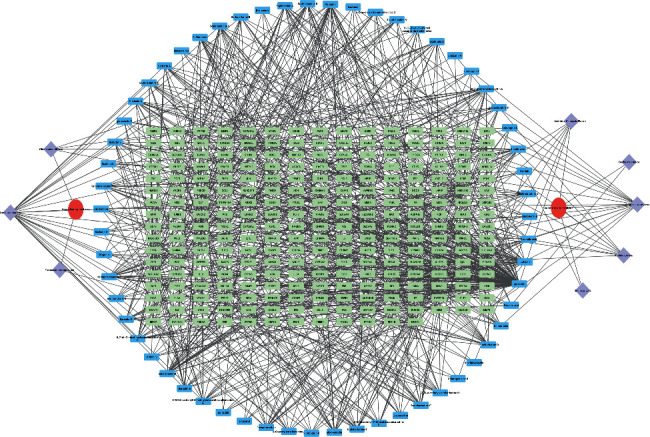
Ingredient-target network of heat-clearing medicine and antirheumatic medicine. The red ellipses, purple diamonds, blue rectangles, and green rectangles represent two types of medicines, eight medicines, 62 ingredients, and 825 targets, respectively. The gray lines denote their relationship.

The ingredient-target network of heat-clearing medicine and antirheumatic medicine was constructed in Cytoscape 3.8.1. As shown in Figure 2, two red ellipses represented two types of medicines, and eight diamonds denoted medicines. Blue rectangles were active ingredients and green ones were relevant targets. The gray lines showed us the relationship between medicines, ingredients, and targets. The network topological parameters including degree, betweenness, and closeness are shown in Table 2.

### 3.3. Relevant Targets of Gastric Cancer

There were 12,155 targets in the GeneCards database, 427 targets in the OMIM database, 42 targets in the TTD database, and 32 targets in the DrugBank database. Gastric cancer-relevant targets were collected, respectively, which were deduplicated and integrated as a dataset containing 12,155 targets (Supplementary Table 2).

### 3.4. Protein-Protein Interaction Network

**Figure 3:**
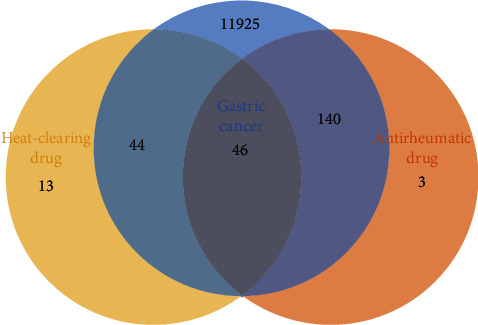
Venn diagram of relevant targets of medicines and gastric cancer. The blue, yellow, and orange circles denote targets of gastric cancer, heat-clearing medicines, and antirheumatic medicines, respectively.

With all the relevant targets of medicines and gastric cancer, we constructed a Venn diagram to display the relationship (shown in Figure 3). The blue circle represented targets of gastric cancer. The yellow circle represented targets of heat-clearing medicines. The orange circles represented targets of antirheumatic medicines. Among all the gastric cancer-relevant targets, 46 targets were the intersection of two types of medicines. A total of 44 targets were related to heat-clearing medicine, while 140 targets were related to antirheumatic medicines.

**Figure 4:**
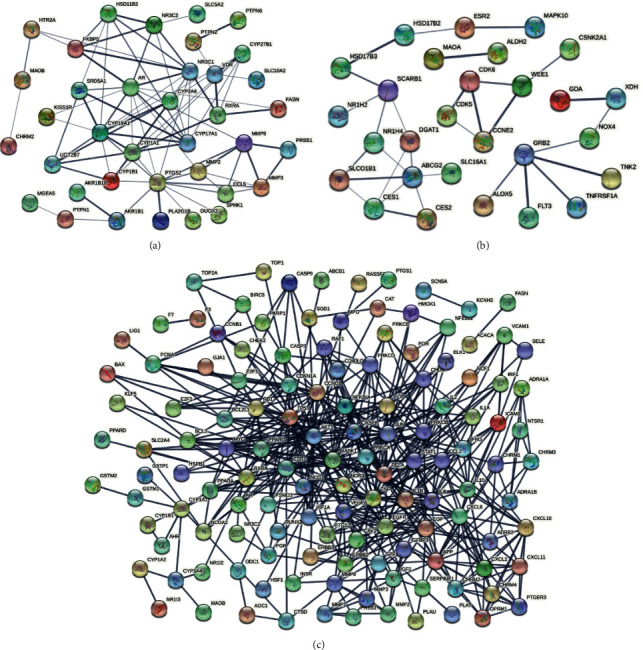
Protein-protein interaction network of coexpression genes: (a) genes expression in both types of medicines, (b) heat-clearing medicine, and (c) antirheumatic medicine. Each node representes a gene. Each line denotes the relationship between genes, and the thickness represents the strength of evidence.

**Figure 5:**
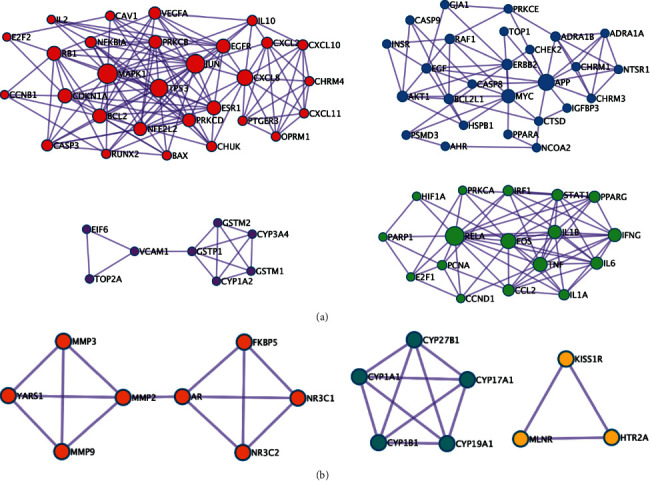
MCODE network of key targets. (a) 80 key targets of antirheumatic medicine were constructed in four MCODE networks. (b) 16 key targets of both types of medicines were constructed in three MCODE networks.

Based on coexpression analysis, gene detection, and gene interaction, the interactive targets were constructed into a PPI network (shown in Figure 4). The thickness of lines indicated the strength of data support from text mining, experiments, databases, coexpression, gene fusion, and cooccurrence. Targets of both medicines (Figure 4(a)) and targets of heat-clearing medicines (Figure 4(b)) constructed relatively simple networks. Targets of antirheumatic medicines constructed a quite complex network with lots of thick lines (Figure 4(c)). The area with thicker lines was considered as a module with higher biological significance, which needed further analysis based on the MCODE algorithm (shown in Figure 5 and Table 3). The MCODE algorithm was applied to identify neighborhoods where proteins were densely connected. Each MCODE network was assigned a unique color. As shown in Figure 5(a), four clusters were found in targets of antirheumatic medicines. Three clusters existed in targets of both medicines (Figure 5(b)). No cluster was found in targets of heat-clearing medicines.

### 3.5. Enrichment Analysis and Visualization

**Figure 6:**
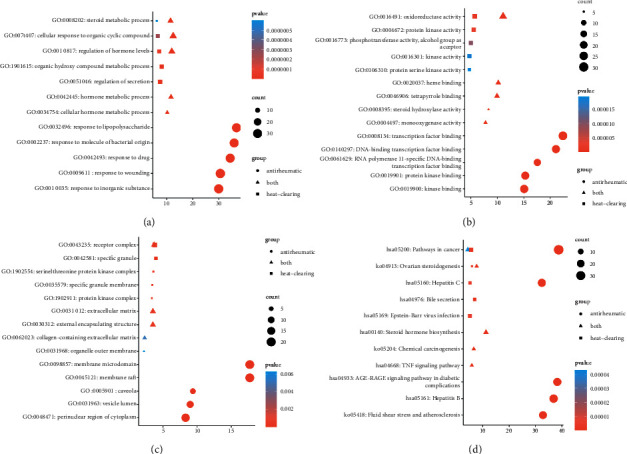
GO and KEGG enrichment analysis: (a) the top enriched pathways of biological process, (b) molecular function, (c) cellular component, and (d) KEGG pathway. The circles, squares, and triangles denote antirheumatic medicine, heat-clearing medicine, and both types of medicine. The node size represents a degree of enrichment. Red color represents more significance of enrichment.

**Figure 7:**
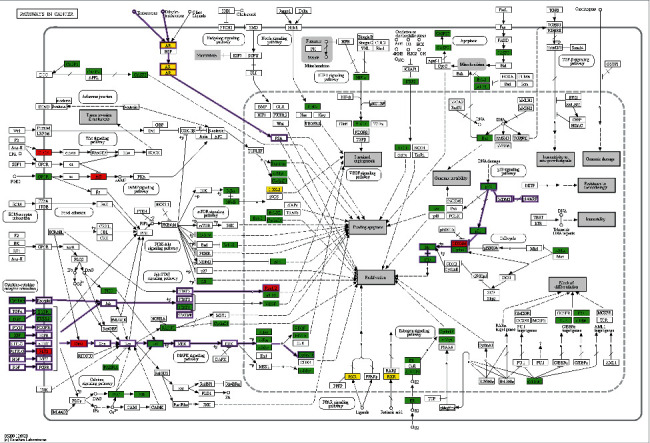
KEGG map of pathways in cancer. The red rectangles represent 6 proteins relevant to heat-clearing medicine. The green rectangles represent 65 proteins of antirheumatic medicine. Six yellow rectangles represent the protein relevant to both types of medicines.

Enrichment analysis was performed based on the hypergeometric test and Benjamini-Hochberg *p*-value correction algorithm. The results of molecular function, biological process, cellular component, and KEGG pathway were visualized in a bubble chart using R software. As shown in Figure 6, antirheumatic medicine ranked the top in all the enrichment analyses. The top enrichment of biological processes included a response to lipopolysaccharide, response to molecule of bacterial origin, and response to the drug (Figure 6(a)). Enriched molecular functions mainly covered transcription factor binding, DNA-binding transcription factor binding, and RNA polymerase II-specific DNA-binding transcription factor binding (Figure 6(b)). Cellular components were mainly involved in the membrane microdomain and membrane raft (Figure 6(c)). As shown in Figure 6(d), both heat-clearing medicine and antirheumatic medicine were involved in pathways of cancer. Antirheumatic medicine had a larger enrichment score. In the KEGG map of pathways in cancer (Figure 7), 65 targets of antirheumatic medicine were labeled green, 6 targets of heat-clearing medicine were marked red, and 6 targets relating to both medicines were in yellow. These targets took part in some important signaling pathways of cancer.

### 3.6. Molecular Docking

According to the result of MCODE algorithm (Table 3) and relevance score (Supplementary Table 2), the proteins with a high correlation with gastric cancer were selected for docking. The ligands were the ingredients related to the selected proteins (Supplementary Table 1).

**Figure 8:**
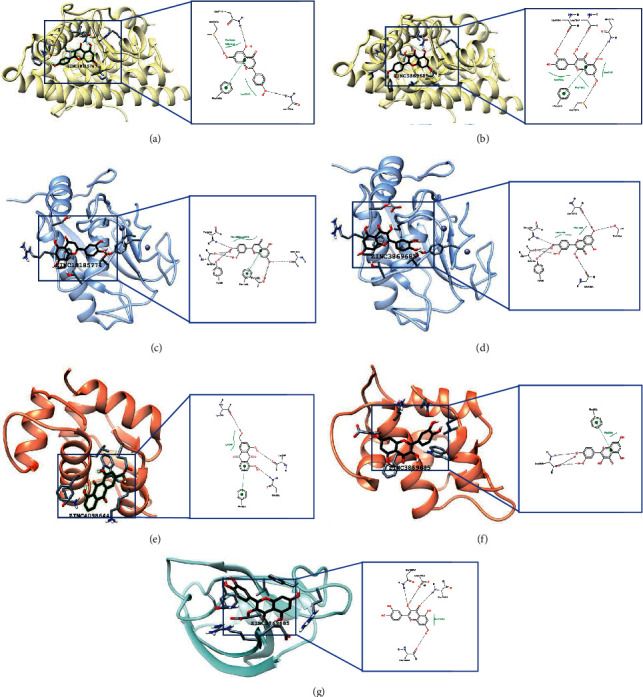
Molecular docking of key targets and ligands. (a) AR and apigenin. (b) AR and quercetin. (c) MMP2 and luteolin. (d) MMP2 and quercetin. (e) TP53 and aloe-emodin. (f) TP53 and quercetin. (g) ERBB2 and quercetin. The ribbons represent the targets, the black sticks represent the ligands, and the gray sticks represent the intermolecular bonding.

After blind docking of ligands and proteins, we classified the docking results. It is generally considered that the lower affinity means a higher possibility of binding. The binding results are shown in Figure 8. The affinity is shown in Table 4. The binding affinity of AR and apigenin (ZINC3871576) was −8.48 kcal/mol. The active binding residues were GLN711, MET787, PHE764, and ASN705 (Figure 8(a)). MMP2 and luteolin (ZINC18185774) were bound with an affinity of −7.93 kcal/mol. The binding residues were THR143, ILE141, TYR3, PHE148, THR145, and ASN147(Figure 8(c)). The binding affinity of aloe-emodin (ZINC4098644) on TP53 was −6.08 kcal/mol. The binding residues were LYS24, PHE55, LEU54, and GLN59 (Figure 8(e)). Quercetin (ZINC3869685) could bind to AR, MMP2, TP53, and ERBB2. The binding of quercetin and AR was −7.82 kcal/mol. The active binding residues were GLN711, MET787, PHE764, LEU704, and LEU873 (Figure 8(b)). Quercetin and MMP2 were bound with an affinity of −7.90 kcal/mol. The binding residues were THR143, ILE141, TYR3, PHE148, THR145, and ASN147(Figure 8(d)). The binding affinity of quercetin on TP53 was −5.49 kcal/mol. The binding residues were PHE55 and LEU26(Figure 8(f)). Quercetin bound ERBB2 in residues of GLN1329, ASP1332, GLU1368, and PHE1306, with an affinity of −5.45 kcal/mol (Figure 8(g)). Among all the docking results, the binding of AR and apigenin had the lowest affinity, which meant that they were the most significant binding in this study.

### 3.7. Analysis of Tumor Immune Infiltration

**Figure 9:**
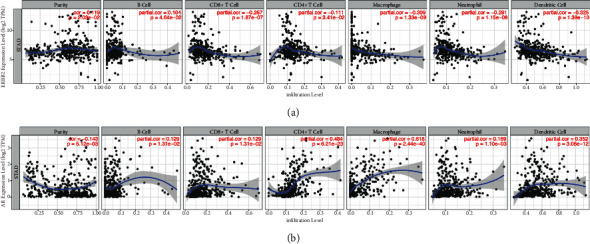
The correlation between gene expression and abundances of six immune infiltrates in gastric cancer: (a) ERBB2, and (b) AR.

Tumor immune infiltration is considered as a key factor of prognosis of tumor. We analyzed AR, MMP2, TP53, and ERBB2 in gastric tumor immune infiltration, and MMP2 and TP53 did not show significant result in this analysis. As shown in Figure 9(a), ERBB2 was negatively correlated with CD8+ T cell (partial.cor = −0.267, *p*=1.87*E* − 07), macrophage (partial.cor = −0.309, *p*=1.33*E* − 09), neutrophil (partial.cor = −0.291, *p*=1.15*E* − 08), and dendritic cell (partial.cor = −0.325, *p*=1.39*E* − 10). As shown in Figure 9(b), AR was positively correlated with CD4+ T cell (partial.cor = 0.484, *p*=6.21*E* − 23), macrophage (partial.cor = 0.618, *p*=2.44*E* − 40), and dendritic cell (partial.cor = 0.352, *p*=3.06*E* − 12).

**Figure 10:**
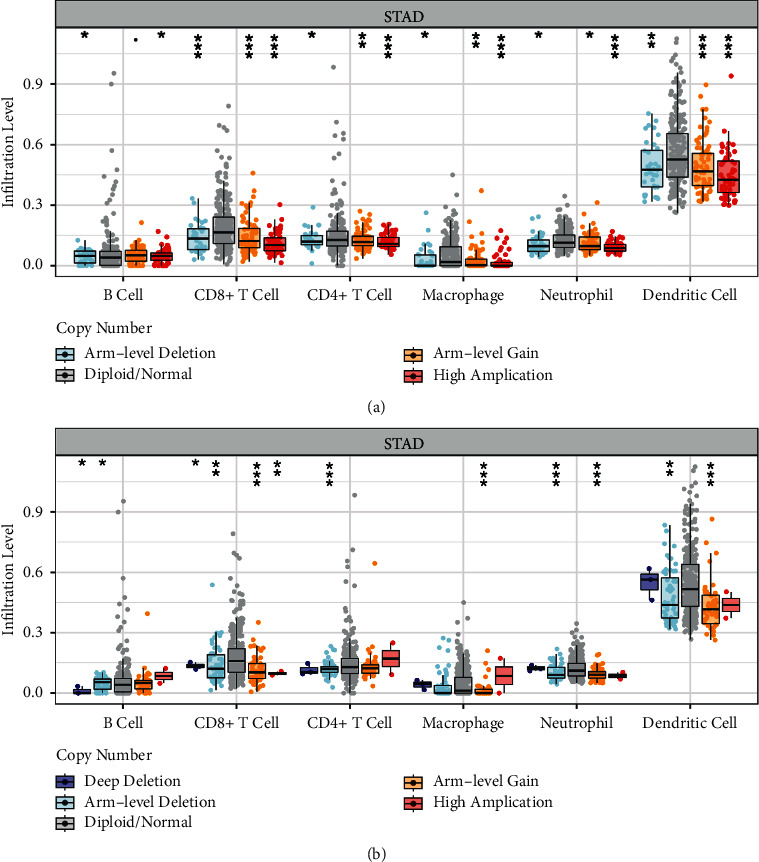
The correlation between immune infiltrates and somatic copy number alterations (SCNAs) of gene expression of gastric cancer: (a) ERBB2, and (b) AR.

sCNA was applied to compare immune infiltration distribution by the sCNA status of gene in STAD (abbreviations of stomach adenocarcinoma in TCGA cancer types). As shown in Table 5, high amplification of ERBB2 was in high correlation with six immune infiltrates (*p* < 0.05, Figure 10(a)). Arm-level deletion and gain of AR were significantly associated with CD8+ T cell, neutrophil, and dendritic cell (*p* < 0.05, Figure 10(b)).

**Figure 11:**
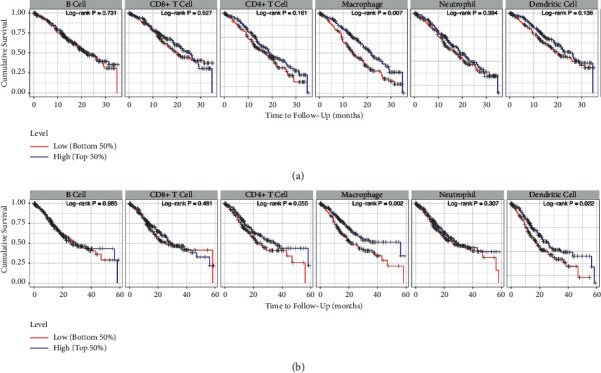
The survival curves of gastric cancer and abundance of immune infiltrate: (a) 3-year time point and (b) 5-year time point.

Based on the TCGA database, the clinical outcome of gastric cancer was used to explore the relevance of tumor immune infiltration with multiple covariates in a multivariable Cox proportional hazard model. The survival curves of gastric cancer showed a significant difference in macrophages at both 3-year time point (log-rank *p*=0.007, Figure 11(a)) and 5-year time point (log-rank *p*=0.002, Figure 11(b)).

## 4. Discussion

Heat-clearing medicine is a category of medicines that have the effect of clearing up internal heat in cases of externally contracted febrile diseases or fever due to yin deficiency. Tumor tissues are commonly in a state of rapid proliferation and high metabolism. The state is consistent with the feature of heat in TCM theory [59]. Based on this theory, several studies explored the mechanism of treating liver cancer, colon cancer, and lung cancer with heat-clearing medicine [60–65]. In clinical application, antirheumatic medicines are often used to treat tumors. HU Dan et al. found the ingredients and antitumor activity of *Tripterygii Radix* [66]. The chemical components of *Actinidia chinensis Planch* were extracted, and the antitumor effects were explored [67–69]. However, no study focused on the antitumor effects of the category of antirheumatic medicine. Antirheumatic medicine is a category of medicine that dispels wind and dampness, mainly for relieving rheumatism and related conditions. Aiming to figure out the mechanism of treating gastric cancer with heat-clearing medicine and antirheumatic medicine, we searched relative ingredients and targets and constructed a network with gastric cancer-related targets. The result of enrichment analysis showed that two types of medicines were involved in pathways of cancer in different degrees of enrichment (Figure 6). All the relevant targets were marked in different colors in the KEGG map of pathways in cancer (Figure 7).

As shown in the KEGG map, testosterone and dihydrotestosterone are connected with androgen receptors (ARs) and expressed as the prostate-specific antigen (PSA). PSA had an indirect function in evading apoptosis (Figure 7). AR is a target closely related to two types of medicine and gastric cancer (relevant score = 54.93). AR is related to two ingredients, namely, apigenin and quercetin. Molecular docking was performed to verify the binding ability of AR and ingredients. The binding strength of AR and apigenin was the strongest among all the bindings in this study (Table 4). AR and quercetin were bounded with a weaker affinity. The result showed that the antitumor effects of both heat-clearing medicine and antirheumatic medicine were closely related to androgen action.

In the KEGG map of pathways in cancer, matrix metalloproteinases (MMPs) were involved in process of sustained angiogenesis (Figure 7). It is generally accepted that the mechanism of MMPs is mostly related to matrix reconstruction, including decomposition of extracellular matrix proteins and cell surface receptors in cancer proliferation [70]. MMP9 and MMP2 are gelatinases, a subtype of matrix metalloproteinases (MMPs). The levels of serum MMP9 and MMP2 in patients with gastric cancer were significantly higher than normal people in clinical practice [71]. MMP9 and MMP2 accelerate tumor metastasis by invading lymphatic vessels and small vessels [72, 73]. On one hand, helicobacter pylori infection might enhance the expression of MMPs, induce cell proliferation, and activate gene oncogenes [74, 75]. On the other hand, MMP9 and MMP2 could degrade type IV collagen in the extracellular matrix and basement membranes. This process plays an important role in tumor invasion and metastasis [76–79]. In this study, the results of MCODE analysis indicated that MMP9 and MMP2 were key targets in the antitumor function of both heat-clearing medicine and antirheumatic medicine. In molecular docking experiments, MMP2 bounded luteolin and quercetin with low binding energy (Table 4). However, MMP9 failed to bind any related ingredients of heat-clearing medicine and antirheumatic medicine.

Based on the p53 signaling pathway in the KEGG map (Figure 7), tumor protein p53 missed the interaction with cyclin-dependent kinase inhibitor 1A(p21). p21 is a mediator of p53 tumor suppressor activity based on functions of growth arresting, differentiation, and senescence [80]. p21 inhibits the complex formation of cyclin-dependent kinase 4/6(CDK4/6) and G1/S-specific cyclin D1(CyclinD) [80]. CDK4/6 plays a key role in cell cycle regulation. After phosphorylation, transcription factor E2F1(E2F) dissociates from retinoblastoma-associated protein (Rb) and expresses as proliferation [81]. To predict the interaction of TP53 and related ingredients of antirheumatic medicine, we performed molecular docking experiments. The binding strength of TP53 and aloe-emodin, TP53, and quercetin was moderate (Table 4). The results revealed that antirheumatic medicine was in closed relation to the p53 signaling pathway.

In the KEGG pathway map, epidermal growth factor (EGF) interacts with epidermal growth factor receptor (EGFR) and receptor tyrosine-protein kinase erbB-2 (ERBB2) (Figure 7). Both EGFR and ERBB2 are receptor protein-tyrosine kinases with a transmembrane domain [82]. These receptors have two ways of activation. One way is activating with growth factor receptor-bound protein 2(Grb2) in the MAPK signaling pathway [83, 84]. The other way is activating with Janus kinase 1(Jak) and phosphorylated as an activator of transcription 1 (STAT1). STAT1 is expressed as a vascular endothelial growth factor (VEGF), with an indirect function of sustained angiogenesis [85]. The pathway of proliferation and sustained angiogenesis mainly happened with antirheumatic medicine. Aiming to testify the binding ability of ERBB2 and its related ingredients, molecular docking experiments were performed. The result showed that the binding strength of ERBB2 and quercetin was moderate (Table 4).

Summarizing the docking results above, quercetin could bind several gastric cancer-related targets. Quercetin existed in both types of medicines. It is an important ingredient in both heat-clearing medicine and antirheumatic medicine. Previous studies showed that quercetin was effective in treating gastric cancer. In a scratch wound healing assay, Jia et al. [86] found that the TGF*β*-1 and quercetin group had worse proliferation capability and migration ability of human gastric carcinoma BGC-803 cells. It revealed that quercetin can effectively inhibit the metastasis and invasion of gastric cancer. Yu. [87] treated gastric cancer MGC-803 cells with quercetin for 48 hours and found that quercetin could decrease the expression of VEGF-C and VEGFR-3. This process is involved in inhibiting gastric cancer proliferation and lymph node metastasis. MC Kim et al. [88] found that quercetin could inhibit mitogen-activated protein kinases (MAPKs) and accelerate apoptosis of AGS cells in gastric cancer.

Based on the theory of tumor microenvironment and antitumor immunity, we analyze the tumor immune infiltration of the key proteins in this study to explore the immune response in tumor tissue. The result of tumor immune infiltration showed that AR was positively correlated with CD4+ T cells, macrophages, and dendritic cells, while ERBB2 was negatively correlated with CD8+ T cells, macrophages, neutrophils, and dendritic cells (Figure 9). However, TP53 and MMP2 showed no significant correlation in tumor immune infiltration analysis. The result revealed that the tumor immune infiltration was different in AR and ERBB2 expression. If the expression of ERBB2 increased, the relevant immune cells decreased. If the expression of AR increased, the relevant immune cells increased.

Combining the results of enrichment analysis and tumor immune infiltration analysis, the pathway of androgen was correlated with both heat-clearing medicine and antirheumatic medicine in treating gastric cancer. There are some possible reasons. First, the incidence of male and female patients with gastric cancer is approximately in the ratio 2 : 1 (estimated number of new gastric cancer in age-standardized rates per ten thousand: men = 15.8, women = 7.0 [1]). A prospective cohort study on upper gastrointestinal cancers and hormonal and reproductive factors showed that male pattern baldness was associated with gastric cancer risk in an adjusted hazard ratio of 1.35 [89]. The evidence above revealed a close association with gastric cancer and sex hormones, especially androgen. Second, men prefer food with stronger flavors, such as barbecue, red meat, and alcohol. Heavy meal combining with yang excessiveness constitution generally leads to heat syndrome. Third, androgens can reduce antibody production and suppress the immune system. Clinically, men are less likely to develop autoimmune diseases than women do [90, 91]. AR signaling has been proved to have a direct or indirect influence on immune cell function. Above all, AR is an important target in both heat-clearing medicine and antirheumatic medicine. Male patients or female patients with androgen excess are more likely to activate AR signaling pathway. Further searches on gastric cancer of sex hormones are required.

In tumor progression, ERBB2 interacts with epidermal growth factor receptors (EGFRs) and activates signaling pathways of tumor proliferation. ERBB2 plays a role in tumor cells by binding to other epidermal growth factor receptors (EGFRs) and then generating a dimer form that activates signaling pathways related to tumor proliferation [92–95]. Trastuzumab is a monoclonal antibody targeting the extracellular region IV of ERBB2. As a targeted drug for gastric cancer, trastuzumab could stop the formation of ERBB2 homologous dimers, prevent the activation of the signaling pathways of cell proliferation, and kill tumor cells through antibody-dependent cellular cytotoxicity [94]. Pertuzumab is another targeted drug for gastric cancer based on ERBB2. It shows positive efficacy in patients with advanced gastric cancer with high expression of ERBB2. In this study, antirheumatic medicine had a similar pathway to trastuzumab and pertuzumab [96]. It inferred that antirheumatic medicine could stop the formation of ERBB2 homologous dimers, prevent cell proliferation, and kill tumor cells. Further research for dose-response analysis and compatibility of drugs is needed.

The results of gastric cancer survival curves showed that macrophage was significantly different at both the 3-year time point and 5-year time point (Figure 11). It revealed that the number of macrophages might influence gastric cancer prognosis. Previous studies found that macrophages are important components of tumor inflammatory infiltrating cells [97–99]. M1 subtype promotes antitumor immunity, while the M2 subtype is associated with tumor progression. The research found that high density of M2 tumor-associated macrophages (TAMs) was correlated with poor disease-free survival and cancer-specific survival [100]. Intraperitoneal TAMs were generally polarized to the M2 phenotype in patients with gastric cancer with peritoneal dissemination [101]. Infiltration of polarized TAMs combined with the TNM stage could be prognostic factors for gastric cancer [102].

This study creatively combined network pharmacology and molecular docking with tumor immune infiltration analysis to explore the pharmacological mechanism of treating gastric cancer with heat-clearing medicine and antirheumatic medicine. Not only the relationship of ingredients and targets were investigated but also the tumor immune infiltration was analyzed. It is a new way to explore TCM in tumor immunology. Here are some limitations of this study. First, the ingredients of medicines are currently not comprehensive. It might lead to some deviation. Further research on the ingredients of herbal medicines is needed. Second, molecular docking experiments were performed to verify the binding of targets and ligands, but the result was based on molecular simulation. Extra in vitro experiments, animal studies, and clinical trials are required to validate the inference.

In conclusion, quercetin is an important ingredient in both heat-clearing medicine and antirheumatic medicine. AR signaling pathway exists in both types of medicines. The mechanism of antitumor effect in antirheumatic medicine was similar to trastuzumab, a targeted drug aimed at ERBB2. Both types of medicines were significant in tumor immune infiltration. The immunology of gastric tumor deserves further research.

## Figures and Tables

**Table 1:** The relevant ingredients of medicines.

Type	Medicine	Ingredient	Ingredient	
Heat-clearing medicine	Oldenlandia diffusa	Oleanolic acid	Oleanolic acid	VDR, HSD11B2, CYP27B1, AKR1B10, POLB, PLA2G1B, PTPN1, RXRA, CYP17A1, SRD5A1, DUOX2, UGT2B7
		Ursolic acid	Ursolic acid	VDR, HSD11B2, AKR1B1, CYP19A1, POLB, PTPN1, PTPN6, PTPRA, PTPRE, RXRA, CYP17A1, SRD5A1, PTPN2, DUOX2, UGT2B7
		10(S)-hydroxypheophytin a	10(S)-hydroxypheophytin a	CYP24A1, FNTA, GRB2, WEE1, CALCRL, ELOVL6, FCER2, LGMN, RIOK2, TNK2, BIRC7, CCL5, DUOX2, HPSE, HSD11B2, KISS1R, LNPEP, NR3C1, PGC, PPP3CA, SFRP1, SLC10A2, SPHK1, VDR
		6-O-(E)-p-coumaroyl scandoside methyl ester	6-O-(E)-p-coumaroyl scandoside methyl ester	AVPR1B, F2R, SLCO1B1, ITGAL, GLB1, LNPEP, OGA, YARS1
	Scutellaria barbata	Apigenin	Apigenin	ABCG2, ADCY5, ALDH2, ALOX5, CBR1, CDK5, CES2, CYP1A1, CYP1B1, FLT3, GDA, HSD17B3, MAOA, MAOB, MAPK10, MECR, NOX4, PIM1, PLAA, PTGS2, TNFRSF1A, TNKS2, XDH, AR, CDK6, CSNK2A1, ESR2, HSD17B2, LYZ, CYP2A6, CYP19A1
		Barbatellarine B	Barbatellarine B	AVPR1B, F2R, PTPRB, SLC16A1, CYP27B1, FKBP5, HSD11B2, KISS1R, NR3C1, PGC, SLC10A2, SRD5A1, VDR
		Barbatin A	Barbatin A	AVPR1B, F2R, FNTA, NR1H4, SLCO1B1, BIRC7, CYP27B1, FKBP5, HSD11B2, KISS1R, NR3C1, SFRP1, SLC10A2, SRD5A1
		Barbatin B	Barbatin B	AVPR1B, FNTA, PTPRB, SLC16A1, BIRC7, CYP27B1, FKBP5, HSD11B2, KISS1R, NR3C1, PGC, SFRP1, SLC10A2, SRD5A1, VDR
		Barbatin C	Barbatin C	CYP17A1, CYP27B1, GLB1, HSD11B2, NR3C1, SRD5A1, UGT2B7, VDR
		Barbatin D	Barbatin D	AVPR1B, CYP24A1, F2R, FNTA, NR1H2, PTPRB, SLC16A1, SLCO1B1, BIRC7, CYP27B1, FKBP5, HSD11B2, KISS1R, NR3C1, RASGRP3
		Barbatin E	Barbatin E	AVPR1B, FNTA, NR1H4, PTPRB, SLCO1B1, CES1, BIRC7, CYP27B1, DUOX2, FKBP5, HSD11B2, NR3C1, PGC, RASGRP3, SFRP1, SLC10A2, VDR
		Pheophorbide a	Pheophorbide a	AVPR1B, GRB2, NR1H2, PTPRB, SLC16A1, WEE1, NR3C2, PDE1B, SCARB1, BIRC7, FKBP5, HSD11B2, KISS1R, LNPEP, MLNR, NR3C1
		Scutebarbatine B	Scutebarbatine B	AVPR1B, CYP24A1, F2R, FNTA, NR1H2, NR1H4, PTPRB, SLC16A1, SLCO1B1, BIRC7, CYP27B1, FKBP5, HSD11B2, KISS1R, NR3C1, RASGRP3
		Scutebarbatine C	Scutebarbatine C	AVPR1B, CHRM2, FNTA, NR1H4, PTPRB, SLC16A1, SLCO1B1, CYP27B1, FKBP5, HSD11B2, KISS1R, NR3C1, SFRP1, SLC10A2, SRD5A1, VDR
		Scutebarbatine D	Scutebarbatine D	AVPR1B, CHRM2, F2R, FNTA, NR1H4, PTPRB, SLC16A1, SLCO1B1, CYP27B1, FKBP5, HSD11B2, KISS1R, MLNR, NR3C1, SLC10A2, SRD5A1
		Luteolin	Luteolin	ABCG2, ADCY5, ALDH2, ALOX5, CBR1, CDK5, CES2, CYP1A1, CYP1B1, FLT3, GDA, HSD17B3, MAOA, MAOB, MAPK10, MECR, NOX4, PIM1, PLAA, TNFRSF1A, TNKS2, XDH, ABCB1, CCNE2, FASN, GPR35, KDM4E, LAP3, MMP12, MMP13, MMP2, MMP3, MMP9, PRSS1
		Scutebarbatine G	Scutebarbatine G	F2R, FNTA, SLC16A1, BIRC7, CYP27B1, HSD11B2, SLC10A2, SRD5A1, UGT2B7, VDR
		Scutebarbatine H	Scutebarbatine H	FNTA, PTPRB, BIRC7, CYP27B1, HSD11B2, NR3C1, SLC10A2, SRD5A1, UGT2B7, VDR
		Scutebata A	Scutebata A	AVPR1B, F2R, FNTA, PTPRB, CCL5, CYP27B1, FKBP5, HSD11B2, KISS1R, NR3C1, PGC, SFRP1, SLC10A2, SRD5A1
		Scutehenanine A	Scutehenanine A	FNTA, SLC16A1, BIRC7, CYP27B1, HSD11B2, NR3C1, RASGRP3, SRD5A1, VDR
		Scutehenanine B	Scutehenanine B	AVPR1B, ERG, F2R, FNTA, SLC16A1, SLCO1B1, BIRC7, CYP27B1, FKBP5, HSD11B2, KISS1R, NR3C1, RASGRP3, SFRP1, SLC10A2, SRD5A1, UGT2B7, VDR
		Scutehenanine C	Scutehenanine C	AVPR1B, ERG, F2R, FNTA, SLCO1B1, CCL5, CYP27B1, FKBP5, HSD11B2, KISS1R, NR3C1, PGC, RASGRP3, SFRP1, SLC10A2, SRD5A1, UGT2B7, VDR
		Scutehenanine D	Scutehenanine D	AVPR1B, F2R, FNTA, NR1H4, PTPRB, SLCO1B1, BIRC7, CYP27B1, FKBP5, HSD11B2, KISS1R, NR3C1, RASGRP3, SLC10A2, SRD5A1, VDR
		Scutellarin	Scutellarin	GRB2, GLB1, HTR2A, OGA, SLC5A2, YARS1
		Wogonin	Wogonin	ADCY5, ALDH2, CBR1, CES2, GDA, PTGS2
		6,7-di-O-nicotinoylscutebarbatine G	6,7-di-O-nicotinoylscutebarbatine G	AVPR1B, F2R, FNTA, SLC16A1, BIRC7, CCL5, CYP27B1, FKBP5, HSD11B2, KISS1R, NR3C1, PGC, SFRP1, SLC10A2, SRD5A1, VDR
		6-O-(2-carbonyl-3-methylbutanoyl)scutehenanine A	6-O-(2-carbonyl-3-methylbutanoyl)scutehenanine A	AVPR1B, F2R, FNTA, NR1H2, PTPRB, SLC16A1, SLCO1B1, BIRC7, CYP27B1, FKBP5, HSD11B2, KISS1R, NR3C1, RASGRP3, SFRP1, SLC10A2, VDR
		6-O-acetylscutehenanine A	6-O-acetylscutehenanine A	AVPR1B, FNTA, BIRC7, CYP27B1, HSD11B2, NR3C1, RASGRP3, SLC10A2, SRD5A1, VDR
		6-O-nicotinoyl-7-O-acetylscutebarbatine G	6-O-nicotinoyl-7-O-acetylscutebarbatine G	AVPR1B, F2R, CYP27B1, FKBP5, HSD11B2, KISS1R, NR3C1, PGC, RASGRP3, SFRP1, SLC10A2, SRD5A1
		7-O-nicotinoylscutebarbatine H	7-O-nicotinoylscutebarbatine H	AVPR1B, F2R, FNTA, PTPRB, SLC16A1, SLCO1B1, CYP27B1, HSD11B2, KISS1R, NR3C1, RASGRP3, SLC10A2, SRD5A1, VDR
	Taraxacum mongolicum	Chlorogenic acid	Chlorogenic acid	GLB1, OGA, YARS1

Antirheumatic medicine	Rabdosia rubescens	D-limonene	D-limonene	AOC1, EBP, ENPEP, CPB2, KDM2A, LIG1, NOS1, PHF8, TAAR1, CYP2A6
		Xindongnin B	Xindongnin B	CYP27B1, GLB1, HSD11B2, RASGRP3, SLC10A2, SRD5A1, UGT2B7, VDR
		Xindongnin C	Xindongnin C	SLC5A8, CYP27B1, HSD11B2, SRD5A1, UGT2B7, VDR
		Xindongnin F	Xindongnin F	GLB1, HSD11B2, SRD5A1, UGT2B7, VDR
		Oleanolic acid	Oleanolic acid	VDR, HSD11B2, CYP27B1, AKR1B10, POLB, PLA2G1B, PTPN1, RXRA, CYP17A1, SRD5A1, DUOX2, UGT2B7
		Ursolic acid	Ursolic acid	VDR, HSD11B2, AKR1B1, CYP19A1, POLB, PTPN1, PTPN6, PTPRA, PTPRE, RXRA, CYP17A1, SRD5A1, PTPN2, DUOX2, UGT2B7
		Isodonol	Isodonol	CYP27B1, GLB1, HSD11B2, SLC10A2, SRD5A1, UGT2B7
		*β*-elemene	*β*-elemene	AOC1, EBP, ENPEP, VDR
		Dawoensin A	Dawoensin A	CYP27B1, HSD11B2, SRD5A1, UGT2B7, VDR
		Glabcensin V	Glabcensin V	CYP27B1, HSD11B2, RASGRP3, SLC10A2, SRD5A1, UGT2B7, VDR
		Guidongnin	Guidongnin	CYP27B1, HSD11B2, NR3C1, RASGRP3, SLC10A2, SRD5A1, UGT2B7, VDR
		Melissoidesin G	Melissoidesin G	CYP27B1, HSD11B2, RASGRP3, SLC10A2, SRD5A1, UGT2B7, VDR
		Oridonin	Oridonin	CYP27B1, GLB1, HSD11B2, SLC10A2, SRD5A1, UGT2B7
		Ponicidin	Ponicidin	CYP27B1, HSD11B2, SFRP1, SRD5A1, UGT2B7
		Xindongnin A	Xindongnin A	BIRC7, CYP27B1, HSD11B2, NR3C1, SLC10A2, SRD5A1, UGT2B7, VDR
	Duchesnea indica	Pomolic acid	Pomolic acid	RXRA, CYP17A1, DUOX2, HSD11B2, SFRP1, UGT2B7, PTPN1
	Smilax glabra	Astilbin	Astilbin	GLB1, HTR2A, LNPEP, OGA, SLC5A2, YARS1
	Akebia quinata	Ariskanin A	Ariskanin A	SLC5A8, TUBB1
		Oleanolic acid	Oleanolic acid	VDR, HSD11B2, CYP27B1, AKR1B10, POLB, PLA2G1B, PTPN1, RXRA, CYP17A1, SRD5A1, DUOX2, UGT2B7
		3-Oxo-olean-12-en-28-oic acid	3-Oxo-olean-12-en-28-oic acid	RXRA, CYP17A1, CYP27B1, DUOX2, HSD11B2, SFRP1, SLC10A2, SRD5A1, UGT2B7, VDR
		Sapindoside A	Sapindoside A	DGAT1, BIRC7, DUOX2, FKBP5, GLB1, HPSE, HSD11B2, KISS1R, LNPEP, MLNR, NR3C1, PGC, PPP3CA, SFRP1, SPHK1, SRD5A1, UGT2B7, VDR
		*β*-Sitosterol	*β*-Sitosterol	DGAT1, EBP, APP, KLF5, SLC10A1, CYP17A1, CYP27B1, DUOX2, HSD11B2, NR3C1, SPHK1, SRD5A1, UGT2B7, VDR, POLB
		Guaianin N	Guaianin N	DUOX2, FKBP5, HPSE, HSD11B2, KISS1R, MLNR, NR3C1, PGC, PPP3CA, SFRP1, SLC10A2, SRD5A1, UGT2B7, VDR
		Kalopanaxsaponin A	Kalopanaxsaponin A	DGAT1, DUOX2, FKBP5, GLB1, HPSE, HSD11B2, KISS1R, LNPEP, MLNR, NR3C1, PGC, SFRP1, SPHK1, SRD5A1, UGT2B7, VDR
		Kalopanaxsaponin I	Kalopanaxsaponin I	NTSR1, CCL5, DUOX2, FKBP5, HPSE, HSD11B2, KISS1R, LNPEP, MLNR, NR3C1, OGA, PGC, PPP3CA, SFRP1, SPHK1, SRD5A1, UGT2B7, VDR
	Actinidia chinensis Planch	Beta-sitosterol	Beta-sitosterol	ADRA1A, ADRA1B, BAX, BCL2, ADRB2, CASP3, CASP8, CASP9, PDE3A, GABRA1, MAP2, OPRM1, CHRM1, CHRM2, CHRM3, CHRM4, CHRNA2, NCOA2, KCNH2, PGR, PTGS1, PTGS2, PRKCA, PON1, SCN5A, SLC6A4, JUN
		Sitosterol	Sitosterol	PGR, NCOA2, NR3C2
		Aloe-emodin	Aloe-emodin	PTGS1, PTGS2, NCOA2, PKIA, CDKN1A, EIF6, BAX, TNF, CASP3, TP53, FASN, PRKCA, PRKCE, PCNA, MYC, IL1B, PRKCD, CCNB1
		(+)-catechin	(+)-catechin	PTGS1, ESR1, PTGS2, NCOA2, RXRA, CAT, HAS2
		Ent-epicatechin	Ent-epicatechin	PTGS1, ESR1, PTGS2
		Quercetin	Quercetin	PTGS1, AR, PTGS2, NCOA2, PRSS1, KCNH2, SCN5A, ADRB2, MMP3, F7, RXRA, ACHE, GABRA1, MAOB, RELA, EGFR, AKT1, VEGFA, CCND1, BCL2, BCL2L1, FOS, CDKN1A, EIF6, BAX, CASP9, PLAU, MMP2, MMP9, MAPK1, IL10, EGF, RB1, TNF, JUN, IL6, AHSA1, CASP3, TP53, ELK1, NFKBIA, POR, ODC1, CASP8, TOP1, RAF1, SOD1, PRKCA, MMP1, HIF1A, STAT1, RUNX1T1, ERBB2, PPARG, ACACA, HMOX1, CYP3A4, CYP1A2, CAV1, MYC, F3, GJA1, CYP1A1, ICAM1, IL1B, CCL2, SELE, VCAM1, PTGER3, CXCL8, PRKCB, BIRC5, DUOX2, NOS3, HSPB1, IL2, NR1I2, CYP1B1, CCNB1, PLAT, THBD, SERPINE1, IFNG, IL1A, MPO, TOP2A, NCF1, HAS2, GSTP1, NFE2L2, PARP1, AHR, PSMD3, SLC2A4, CXCL11, CXCL2, DCAF5, NR1I3, CHEK2, INSR, CLDN4, PPARA, PPARD, HSF1, CXCL10, CHUK, SPP1, RUNX2, RASSF1, E2F1, E2F2, ACP3, CTSD, IGFBP3, IGF2, CD40LG, IRF1, ERBB3, PON1, DIO1, PCOLCE, NPEPPS, HK2, NKX3-1, RASA1, GSTM1, GSTM2

**Table 2:** Network topological parameters.

	Name	Degree	Betweenness	Closeness
Target	HSD11B2	43	5407.4346	0.3171
	SRD5A1	36	2855.4278	0.3033
	CYP27B1	34	1962.1760	0.2977
	VDR	33	3799.5332	0.3092
	NR3C1	29	1692.5265	0.2999
	SLC10A2	27	1111.4990	0.2944
	UGT2B7	25	1637.7742	0.2901
	KISS1R	21	823.8508	0.2875
	FKBP5	20	642.3051	0.2839
	AVPR1B	19	512.6839	0.2805

Ingredient	Quercetin	128	62208.7944	0.4178
	Luteolin	35	11794.5504	0.3194
	Apigenin	32	12547.0981	0.3412
	Beta-sitosterol	28	8122.2824	0.2980
	10(S)-hydroxypheophytin a	25	7106.1379	0.3239
	Kalopanaxsaponin I	19	3471.1257	0.3219
	Aloe-emodin	19	3255.0639	0.2862
	Sapindoside A	19	2787.3090	0.3219
	Scutehenanine C	19	935.7868	0.2740
	Scutehenanine B	19	876.0334	0.2740

**Table 3:** The parameter of the MCODE network.

	Score	Gene	Symbol	Network
Clusters for both types of medicines	1.25	1588	CYP19A1	MyList_SUB1
	1.25	6715	SRD5A1	MyList_SUB1
	1.25	7364	UGT2B7	MyList_SUB1
	1.25	1586	CYP17A1	MyList_SUB1
	1	3356	HTR2A	MyList_SUB2
	1	2862	MLNR	MyList_SUB2
	1	84634	KISS1R	MyList_SUB2

Clusters for heat-clearing medicine	1.79999999999999	5644	PRSS1	MyList_SUB1
	1.79999999999999	4322	MMP13	MyList_SUB1
	1.79999999999999	4314	MMP3	MyList_SUB1
	1.79999999999999	4318	MMP9	MyList_SUB1
	**1.79999999999999**	**4313**	**MMP2**	**Mylist_SUB1**
	1	217	ALDH2	MyList_SUB2
	1	4128	MAOA	MyList_SUB2
	1	4129	MAOB	MyList_SUB2

Clusters for antirheumatic medicine	6.5	1869	E2F1	MyList_SUB2
	6.5	3725	JUN	MyList_SUB2
	**6.5**	**7157**	**TP53**	**Mylist_SUB2**
	6.5	3659	IRF1	MyList_SUB2
	6.5	581	BAX	MyList_SUB2
	6.5	3576	CXCL8	MyList_SUB2
	6.5	5111	PCNA	MyList_SUB2
	6.5	595	CCND1	MyList_SUB2
	6.5	4609	MYC	MyList_SUB2
	6.5	5594	MAPK1	MyList_SUB2
	6.5	5970	RELA	MyList_SUB2
	6.5	1147	CHUK	MyList_SUB2
	6.5	4318	MMP9	MyList_SUB2
	6.5	5925	RB1	MyList_SUB2
	6.5	207	AKT1	MyList_SUB2
	6.5	1956	EGFR	MyList_SUB2
	6.5	2099	ESR1	MyList_SUB2
	6.5	3091	HIF1A	MyList_SUB2
	6.5	860	RUNX2	MyList_SUB2
	6.5	142	PARP1	MyList_SUB2
	6.5	5578	PRKCA	MyList_SUB2
	6.5	3558	IL2	MyList_SUB2
	6.5	2353	FOS	MyList_SUB2
	**6.5**	**367**	**AR**	**MyList_SUB2**
	3.375	6347	CCL2	MyList_SUB4
	3.375	3586	IL10	MyList_SUB4
	3.375	5743	PTGS2	MyList_SUB4
	3.375	5733	PTGER3	MyList_SUB4
	3.375	6373	CXCL11	MyList_SUB4
	3.375	1132	CHRM4	MyList_SUB4
	3.375	3553	IL1B	MyList_SUB4
	3.375	3552	IL1A	MyList_SUB4
	3.375	1129	CHRM2	MyList_SUB4
	3.375	4988	OPRM1	MyList_SUB4
	3.375	2920	CXCL2	MyList_SUB4
	3.375	3458	IFNG	MyList_SUB4
	3.375	3569	IL6	MyList_SUB4
	3.375	3627	CXCL10	MyList_SUB4
	3.375	5468	PPARG	MyList_SUB4
	3.375	6772	STAT1	MyList_SUB4
	3.03125	5709	PSMD3	MyList_SUB1
	3.03125	1576	CYP3A4	MyList_SUB1
	3.03125	3383	ICAM1	MyList_SUB1
	3.03125	1544	CYP1A2	MyList_SUB1
	3.03125	1543	CYP1A1	MyList_SUB1
	3.03125	7124	TNF	MyList_SUB1
	3.03125	2950	GSTP1	MyList_SUB1
	3.03125	3486	IGFBP3	MyList_SUB1
	3.03125	836	CASP3	MyList_SUB1
	3.03125	5465	PPARA	MyList_SUB1
	3.03125	596	BCL2	MyList_SUB1
	3.03125	10499	NCOA2	MyList_SUB1
	3.03125	891	CCNB1	MyList_SUB1
	3.03125	5580	PRKCD	MyList_SUB1
	3.03125	1545	CYP1B1	MyList_SUB1
	3.03125	4846	NOS3	MyList_SUB1
	3.03125	1870	E2F2	MyList_SUB1
	3.03125	1026	CDKN1A	MyList_SUB1
	3.03125	1950	EGF	MyList_SUB1
	3.03125	5579	PRKCB	MyList_SUB1
	3.03125	857	CAV1	MyList_SUB1
	3.03125	7422	VEGFA	MyList_SUB1
	3.03125	5241	PGR	MyList_SUB1
	3.03125	196	AHR	MyList_SUB1
	3.03125	2946	GSTM2	MyList_SUB1
	3.03125	2944	GSTM1	MyList_SUB1
	3.03125	11200	CHEK2	MyList_SUB1
	3.03125	4312	MMP1	MyList_SUB1
	3.03125	3315	HSPB1	MyList_SUB1
	3.03125	6256	RXRA	MyList_SUB1
	3.03125	5894	RAF1	MyList_SUB1
	3.03125	4792	NFKBIA	MyList_SUB1
	1.85	147	ADRA1B	MyList_SUB3
	1.85	7153	TOP2A	MyList_SUB3
	1.85	653361	NCF1	MyList_SUB3
	1.85	7412	VCAM1	MyList_SUB3
	1.85	5581	PRKCE	MyList_SUB3
	1.85	4780	NFE2L2	MyList_SUB3
	1.85	598	BCL2L1	MyList_SUB3
	1.85	148	ADRA1A	MyList_SUB3
	**1.85**	**4313**	**MMP2**	**Mylist_SUB3**
	1.85	4923	NTSR1	MyList_SUB3
	1.85	841	CASP8	MyList_SUB3
	1.85	959	CD40LG	MyList_SUB3
	1.85	351	APP	MyList_SUB3
	1.85	1509	CTSD	MyList_SUB3
	1.85	3481	IGF2	MyList_SUB3
	1.85	2065	ERBB3	MyList_SUB3
	**1.85**	**2064**	**ERBB2**	**MyList_SUB3**
	1.85	1128	CHRM1	MyList_SUB3
	1.85	1131	CHRM3	MyList_SUB3
	1.85	7150	TOP1	MyList_SUB3

Bold represents the targets with high relevance score and selected for docking.

**Table 4:** The result of molecular docking.

Target		Ligand	Binding energy (kcal/mol)	Binding residues	
(a)	AR	Apigenin (ZINC3871576)	−8.48	GLN711, MET787, PHE764, ASN705	15
(b)	AR	Quercetin (ZINC3869685)	−7.82	GLN711, MET787, PHE764, LEU704, LEU873	18
(c)	MMP2	Luteolin (ZINC18185774)	−7.93	THR143, ILE141, TYR3, PHE148, THR145, ASN147	56
(d)	MMP2	Quercetin (ZINC3869685)	−7.90	THR143, ILE141, TYR3, GLY135, THR145, ASN147	58
(e)	TP53	Aloe-emodin (ZINC4098644)	−6.08	LYS24, PHE55, LEU54, GLN59	37
(f)	TP53	Quercetin (ZINC3869685)	−5.49	PHE55, LEU26	38
(g)	ERBB2	Quercetin (ZINC3869685)	−5.45	GLN1329, ASP1332, GLU1368, PHE1306	48

**Table 5:** The correlation between immune infiltrates and somatic copy number alterations.

	ERBB2				
	Arm-level deletion	Diploid/normal	Arm-level gain	High amplification	
B cell	2.74*E* − 02	1.00*E* + 00	9.51*E* − 02	4.20*E* − 02	
CD8+ T cell	4.08*E* − 04	1.00*E* + 00	2.66*E* − 04	4.21*E* − 13	
CD4+ T cell	2.70*E* − 02	1.00*E* + 00	7.32*E* − 03	7.90*E* − 05	
Macrophage	4.45*E* − 02	1.00*E* + 00	2.30*E* − 03	8.61*E* − 07	
Neutrophil	2.03*E* − 02	1.00*E* + 00	1.38*E* − 02	2.22*E* − 12	
Dendritic cell	1.73*E* − 03	1.00*E* + 00	1.42*E* − 04	3.89*E* − 09	

	AR				
	Arm-level deletion	Diploid/normal	Arm-level gain	High amplification	Deep deletion
B cell	3.91*E* − 02	1.00*E* + 00	3.78*E* − 01	6.37*E* − 01	2.44*E* − 02
CD8+ T cell	2.32*E* − 03	1.00*E* + 00	3.69*E* − 07	7.58*E* − 03	1.03*E* − 02
CD4+ T cell	6.56*E* − 04	1.00*E* + 00	2.59*E* − 01	8.27*E* − 01	1.60*E* − 01
Macrophage	3.20*E* − 01	1.00*E*+00	4.75*E* − 05	7.40*E* − 01	6.74*E* − 01
Neutrophil	9.78*E* − 04	1.00*E* + 00	1.26*E* − 06	2.53*E* − 01	9.48*E* − 01
Dendritic cell	1.19*E* − 03	1.00*E* + 00	9.62*E* − 08	3.30*E* − 01	9.63*E* − 01

## Data Availability

The data can be found in the database, including TCMSP database (https://old.tcmsp-e.com/tcmsp.php), CancerHSP database (https://old.tcmsp-e.com/CancerHSP.php), UniProt protein database (https://www.uniprot.org), GeneCards database (https://www.genecards.org), OMIM database (https://www.omim.org), TTD database (https://bidd.nus.edu.sg/group/cjttd), and DrugBank database (https://www.drugbank.ca).

## Supplementary Materials

Supplementary Materials Supplementary Table 1: The relevant targets of medicines. Supplementary Table 2: Gastric cancer-related targets and relevance score.
