# Supplementary material for: Comparison between Heat-Clearing Medicine and Antirheumatic Medicine in Treatment of Gastric Cancer Based on Network Pharmacology, Molecular Docking, and Tumor Immune Infiltration Analysis
Source: Evid Based Complement Alternat Med. 2022 Jan 11;2022:7490279. doi: 10.1155/2022/7490279 (PMC8767399; doi:10.1155/2022/7490279)
Supplement: Supplementary Materials — Supplementary Table 1: The relevant targets of medicines. Supplementary Table 2: Gastric cancer-related targets and relevance score. [file 7490279.f1.zip › 7490279.f1/Supplementary Table 1.pdf]

Supplementary Table 1. The relevant targets of medicines

Type	Ingredient	Relevant target
	Oleanolic acid	VDR, HSD11B2, CYP27B1, AKR1B10, POLB, PLA2G1B, PTPN1, RXRA, CYP17A1, SRD5A1, DUOX2, UGT2B7
	Ursolic acid	VDR, HSD11B2, AKR1B1, CYP19A1, POLB, PTPN1, PTPN6, PTPRA, PTPRE, RXRA, CYP17A1, SRD5A1, PTPN2, DUOX2, UGT2B7
	10(S)-hydroxypheophytin a	CYP24A1, FNTA, GRB2, WEE1, CALCRL, ELOVL6, FCER2, LGMN, RIOK2, TNK2, BIRC7, CCL5, DUOX2, HPSE, HSD11B2, KISS1R, LNPEP, NR3C1, PGC, PPP3CA, SFRP1, SLC10A2, SPHK1, VDR
	6-O-(E)-p-coumaroyl scandoside methyl ester	AVPR1B, F2R, SLCO1B1, ITGAL, GLB1, LNPEP, OGA, YARS1
	Apigenin	ABCG2, ADCY5, ALDH2, ALOX5, CBR1, CDK5, CES2, CYP1A1, CYP1B1, FLT3, GDA, HSD17B3, MAOA, MAOB, MAPK10, MECR, NOX4, PIM1, PLAA, PTGS2, TNFRSF1A, TNKS2, XDH, AR, CDK6, CSNK2A1, ESR2, HSD17B2, LYZ, CYP2A6, CYP19A1
	Barbatellarine B	AVPR1B, F2R, PTPRB, SLC16A1, CYP27B1, FKBP5, HSD11B2, KISS1R, NR3C1, PGC, SLC10A2, SRD5A1, VDR
	Barbatin A	AVPR1B, F2R, FNTA, NR1H4, SLCO1B1, BIRC7, CYP27B1, FKBP5, HSD11B2, KISS1R, NR3C1, SFRP1, SLC10A2, SRD5A1
	Barbatin B	AVPR1B, FNTA, PTPRB, SLC16A1, BIRC7, CYP27B1, FKBP5, HSD11B2, KISS1R, NR3C1, PGC, SFRP1, SLC10A2, SRD5A1, VDR
	Barbatin C	CYP17A1, CYP27B1, GLB1, HSD11B2, NR3C1, SRD5A1, UGT2B7, VDR
	Barbatin D	AVPR1B, CYP24A1, F2R, FNTA, NR1H2, PTPRB, SLC16A1, SLCO1B1, BIRC7, CYP27B1, FKBP5, HSD11B2, KISS1R, NR3C1, RASGRP3
	Barbatin E	AVPR1B, FNTA, NR1H4, PTPRB, SLCO1B1, CES1, BIRC7, CYP27B1, DUOX2, FKBP5, HSD11B2, NR3C1, PGC, RASGRP3, SFRP1, SLC10A2, VDR
	Pheophorbide a	AVPR1B, GRB2, NR1H2, PTPRB, SLC16A1, WEE1, NR3C2, PDE1B, SCARB1, BIRC7, FKBP5, HSD11B2, KISS1R, LNPEP, MLNR, NR3C1
	Scutebarbatine B	AVPR1B, CYP24A1, F2R, FNTA, NR1H2, NR1H4, PTPRB, SLC16A1, SLCO1B1, BIRC7, CYP27B1, FKBP5, HSD11B2, KISS1R, NR3C1, RASGRP3

Heat-clearing medicine	Scutebarbatine C	AVPR1B, CHRM2, FNTA, NR1H4, PTPRB, SLC16A1, SLCO1B1, CYP27B1, FKBP5, HSD11B2, KISS1R, NR3C1, SFRP1, SLC10A2, SRD5A1, VDR
	Scutebarbatine D	AVPR1B, CHRM2, F2R, FNTA, NR1H4, PTPRB, SLC16A1, SLCO1B1, CYP27B1, FKBP5, HSD11B2, KISS1R, MLNR, NR3C1, SLC10A2, SRD5A1
	Luteolin	ABCG2, ADCY5, ALDH2, ALOX5, CBR1, CDK5, CES2, CYP1A1, CYP1B1, FLT3, GDA, HSD17B3, MAOA, MAOB, MAPK10, MECR, NOX4, PIM1, PLAA, TNFRSF1A, TNKS2, XDH, ABCB1, CCNE2, FASN, GPR35, KDM4E, LAP3, MMP12, MMP13, MMP2, MMP3, MMP9, PRSS1
	Scutebarbatine G	F2R, FNTA, SLC16A1, BIRC7, CYP27B1, HSD11B2, SLC10A2, SRD5A1, UGT2B7, VDR
	Scutebarbatine H	FNTA, PTPRB, BIRC7, CYP27B1, HSD11B2, NR3C1, SLC10A2, SRD5A1, UGT2B7, VDR
	Scutebata A	AVPR1B, F2R, FNTA, PTPRB, CCL5, CYP27B1, FKBP5, HSD11B2, KISS1R, NR3C1, PGC, SFRP1, SLC10A2, SRD5A1
	Scutehenanine A	FNTA, SLC16A1, BIRC7, CYP27B1, HSD11B2, NR3C1, RASGRP3, SRD5A1, VDR
	Scutehenanine B	AVPR1B, ERG, F2R, FNTA, SLC16A1, SLCO1B1, BIRC7, CYP27B1, FKBP5, HSD11B2, KISS1R, NR3C1, RASGRP3, SFRP1, SLC10A2, SRD5A1, UGT2B7, VDR
	Scutehenanine C	AVPR1B, ERG, F2R, FNTA, SLCO1B1, CCL5, CYP27B1, FKBP5, HSD11B2, KISS1R, NR3C1, PGC, RASGRP3, SFRP1, SLC10A2, SRD5A1, UGT2B7, VDR
	Scutehenanine D	AVPR1B, F2R, FNTA, NR1H4, PTPRB, SLCO1B1, BIRC7, CYP27B1, FKBP5, HSD11B2, KISS1R, NR3C1, RASGRP3, SLC10A2, SRD5A1, VDR
	Scutellarin	GRB2, GLB1, HTR2A, OGA, SLC5A2, YARS1
	Wogonin	ADCY5, ALDH2, CBR1, CES2, GDA, PTGS2
	6,7-di-O-nicotinoylscutebarbatine G	AVPR1B, F2R, FNTA, SLC16A1, BIRC7, CCL5, CYP27B1, FKBP5, HSD11B2, KISS1R, NR3C1, PGC, SFRP1, SLC10A2, SRD5A1
	6-O-(2-carbonyl-3-methylbutanoyl)scutehenanine A	AVPR1B, F2R, FNTA, NR1H2, PTPRB, SLC16A1, SLCO1B1, BIRC7, CYP27B1, FKBP5, HSD11B2, KISS1R, NR3C1
	6-O-acetylscutehenanine A	AVPR1B, FNTA, BIRC7, CYP27B1, HSD11B2, NR3C1, RASGRP3, SLC10A2, SRD5A1, VDR
	6-O-nicotinoyl-7-O-acetylscutebarbatine G	AVPR1B, F2R, CYP27B1, FKBP5, HSD11B2, KISS1R, NR3C1, PGC, RASGRP3, SFRP1, SLC10A2, SRD5A1
	7-O-nicotinoylscutebarbatine H	AVPR1B, F2R, FNTA, PTPRB, SLC16A1, SLCO1B1, CYP27B1, HSD11B2, KISS1R, NR3C1, RASGRP3, SLC10A2, SRD5A1, VDR

Chlorogenic acid	GLB1, OGA, YARS1
D-limonene	AOC1, EBP, ENPEP, CPB2, KDM2A, LIG1, NOS1, PHF8, TAAR1, CYP2A6
xindongnin B	CYP27B1, GLB1, HSD11B2, RASGRP3, SLC10A2, SRD5A1, UGT2B7, VDR
xindongnin C	SLC5A8, CYP27B1, HSD11B2, SRD5A1, UGT2B7, VDR
xindongnin F	GLB1, HSD11B2, SRD5A1, UGT2B7, VDR
Oleanolic acid	VDR, HSD11B2, CYP27B1, AKR1B10, POLB, PLA2G1B, PTPN1, RXRA, CYP17A1, SRD5A1, DUOX2, UGT2B7
Ursolic acid	VDR, HSD11B2, AKR1B1, CYP19A1, POLB, PTPN1, PTPN6, PTPRA, PTPRE, RXRA, CYP17A1, SRD5A1, PTPN2, DUOX2, UGT2B7
Isodonol	CYP27B1, GLB1, HSD11B2, SLC10A2, SRD5A1, UGT2B7
β-elemene	AOC1, EBP, ENPEP, VDR
dawoensin A	CYP27B1, HSD11B2, SRD5A1, UGT2B7, VDR
glabcensin V	CYP27B1, HSD11B2, RASGRP3, SLC10A2, SRD5A1, UGT2B7, VDR
guidongnin	CYP27B1, HSD11B2, NR3C1, RASGRP3, SLC10A2, SRD5A1, UGT2B7, VDR
melissoidesin G	CYP27B1, HSD11B2, RASGRP3, SLC10A2, SRD5A1, UGT2B7, VDR
oridonin	CYP27B1, GLB1, HSD11B2, SLC10A2, SRD5A1, UGT2B7
ponicidin	CYP27B1, HSD11B2, SFRP1, SRD5A1, UGT2B7
xindongnin A	BIRC7, CYP27B1, HSD11B2, NR3C1, SLC10A2, SRD5A1, UGT2B7, VDR
Pomolic acid	RXRA, CYP17A1, DUOX2, HSD11B2, SFRP1, UGT2B7, PTPN1
Astilbin	GLB1, HTR2A, LNPEP, OGA, SLC5A2, YARS1
ariskanin A	SLC5A8, TUBB1
Oleanolic acid	VDR, HSD11B2, CYP27B1, AKR1B10, POLB, PLA2G1B, PTPN1, RXRA, CYP17A1, SRD5A1, DUOX2, UGT2B7
3-Oxo-olean-12-en-28-oic acid	RXRA, CYP17A1, CYP27B1, DUOX2, HSD11B2, SFRP1, SLC10A2, SRD5A1, UGT2B7, VDR
Sapindoside A	DGAT1, BIRC7, DUOX2, FKBP5, GLB1, HPSE, HSD11B2, KISS1R, LNPEP, MLNR, NR3C1, PGC, PPP3CA, SFRP1, SPHK1, SRD5A1, UGT2B7, VDR

Antirheumatic medicine	β-Sitosterol	DGAT1, EBP, APP, KLF5, SLC10A1, CYP17A1, CYP27B1, DUOX2, HSD11B2, NR3C1, SPHK1, SRD5A1, UGT2B7, VDR, [illegible]
	Guaianin N	DUOX2, FKBP5, HPSE, HSD11B2, KISS1R, MLNR, NR3C1, PGC, PPP3CA, SFRP1, SLC10A2, SRD5A1, UGT2B7, VDR
	Kalopanaxsaponin A	DGAT1, DUOX2, FKBP5, GLB1, HPSE, HSD11B2, KISS1R, LNPEP, MLNR, NR3C1, PGC, SFRP1, SPHK1, SRD5A1, UGT2B7, VDR
	Kalopanaxsaponin I	NTSR1, CCL5, DUOX2, FKBP5, HPSE, HSD11B2, KISS1R, LNPEP, MLNR, NR3C1, OGA, PGC, PPP3CA, SFRP1, SPHK1, SRD5A1, UGT2B7, VDR
	beta-sitosterol	ADRA1A, ADRA1B, BAX, BCL2, ADRB2, CASP3, CASP8, CASP9, PDE3A, GABRA1, MAP2, OPRM1, CHRM1, CHRM2, CHRM3, CHRM4, CHRNA2, NCOA2, KCNH2, PGR, PTGS1, PTGS2, PRKCA, PON1, SCN5A, SLC6A4, JUN
	sitosterol	PGR, NCOA2, NR3C2
	aloe-emodin	PTGS1, PTGS2, NCOA2, PKIA, CDKN1A, EIF6, BAX, TNF, CASP3, TP53, FASN, PRKCA, PRKCE, PCNA, MYC, IL1B, PRKCD, CCNB1
	(+)-catechin	PTGS1, ESR1, PTGS2, NCOA2, RXRA, CAT, HAS2
	ent-Epicatechin	PTGS1, ESR1, PTGS2
	quercetin	PTGS1, AR, PTGS2, NCOA2, PRSS1, KCNH2, SCN5A, ADRB2, MMP3, F7, RXRA, ACHE, GABRA1, MAOB, RELA, EGFR, AKT1, VEGFA, CCND1, BCL2, BCL2L1, FOS, CDKN1A, EIF6, BAX, CASP9, PLAU, MMP2, MMP9, MAPK1, IL10, EGF, RB1, TNF, JUN, IL6, AHSA1, CASP3, TP53, ELK1, NFKBIA, POR, ODC1, CASP8, TOP1, RAF1, SOD1, PRKCA, MMP1, HIF1A, STAT1, RUNX1T1, ERBB2, PPARG, ACACA, HMOX1, CYP3A4, CYP1A2, CAV1, MYC, F3, GJA1, CYP1A1, ICAM1, IL1B, CCL2, SELE, VCAM1, PTGER3, CXCL8, PRKCB, BIRC5, DUOX2, NOS3, HSPB1, IL2, NR1I2, CYP1B1, CCNB1, PLAT, THBD, SERPINE1, IFNG, IL1A, MPO, TOP2A, NCF1, HAS2, GSTP1, NFE2L2, PARP1, AHR, PSMD3, SLC2A4, CXCL11, CXCL2, DCAF5, NR1I3, CHEK2, INSR, CLDN4, PPARA, PPARD, HSF1, CXCL10, CHUK, SPP1, RUNX2, RASSF1, E2F1, E2F2, ACP3, CTSD, IGFBP3, IGF2, CD40LG, IRF1, ERBB3, PON1, DIO1, PCOLCE, NPEPPS
