# Supplementary material for: Comparison between Heat-Clearing Medicine and Antirheumatic Medicine in Treatment of Gastric Cancer Based on Network Pharmacology, Molecular Docking, and Tumor Immune Infiltration Analysis
Source: Evid Based Complement Alternat Med. 2022 Jan 11;2022:7490279. doi: 10.1155/2022/7490279 (PMC8767399; doi:10.1155/2022/7490279)
Supplement: Supplementary Materials — Supplementary Table 1: The relevant targets of medicines. Supplementary Table 2: Gastric cancer-related targets and relevance score. [file 7490279.f1.zip › 7490279.f1/Supplementary Table 2.pdf]

Supplementary Table 2. Gastric cancer-related targets and relevance score

Gene Symbol	Description	Relevance score
CDH1	Cadherin 1	220.10843
BRCA2	BRCA2 DNA Repair Associated	204.89577
BRCA1	BRCA1 DNA Repair Associated	196.31686
TP53	Tumor Protein P53	171.68176
APC	APC Regulator Of WNT Signaling Pathway	159.09920
KRAS	KRAS Proto-Oncogene, GTPase	139.90942
MSH2	MutS Homolog 2	127.80608
PTEN	Phosphatase And Tensin Homolog	126.92107
MSH6	MutS Homolog 6	126.88795
ATM	ATM Serine/Threonine Kinase	123.91252
MLH1	MutL Homolog 1	123.36346
ERBB2	Erb-B2 Receptor Tyrosine Kinase 2	120.35851
CHEK2	Checkpoint Kinase 2	116.40714
PALB2	Partner And Localizer Of BRCA2	116.35959
PIK3CA	Phosphatidylinositol-4,5-Bisphosphate 3-Kinase Catalytic Subunit Alpha	109.66935
MUTYH	MutY DNA Glycosylase	104.85806
PMS2	PMS1 Homolog 2, Mismatch Repair System Component	101.51447
MIR21	MicroRNA 21	101.15137
EGFR	Epidermal Growth Factor Receptor	100.90176
CDKN2A	Cyclin Dependent Kinase Inhibitor 2A	97.21557
BRIP1	BRCA1 Interacting Helicase 1	93.85802
MIR34A	MicroRNA 34a	90.08591
BRAF	B-Raf Proto-Oncogene, Serine/Threonine Kinase	86.68905
POLE	DNA Polymerase Epsilon, Catalytic Subunit	86.01951
STK11	Serine/Threonine Kinase 11	85.88132
BARD1	BRCA1 Associated RING Domain 1	85.80699
SMAD4	SMAD Family Member 4	85.66779
MET	MET Proto-Oncogene, Receptor Tyrosine Kinase	84.34692
CTNNB1	Catenin Beta 1	83.95222
CTNNA1	Catenin Alpha 1	83.60220
IL1B	Interleukin 1 Beta	83.02031
AKT1	AKT Serine/Threonine Kinase 1	82.88190
NBN	Nibrin	81.86231
MIR221	MicroRNA 221	80.48935
RAD51C	RAD51 Paralog C	78.63350
RAD51D	RAD51 Paralog D	77.24120
MIR145	MicroRNA 145	77.15990
RB1	RB Transcriptional Corepressor 1	76.71037
MIR222	MicroRNA 222	76.13478
FGFR2	Fibroblast Growth Factor Receptor 2	75.38242
RET	Ret Proto-Oncogene	74.17009

MIR200C	MicroRNA 200c	74.01199
MIR141	MicroRNA 141	73.81945
MIR20A	MicroRNA 20a	73.27239
NF1	Neurofibromin 1	72.18862
AXIN2	Axin 2	72.05137
C11orf65	Chromosome 11 Open Reading Frame 65	70.93073
IL1RN	Interleukin 1 Receptor Antagonist	70.84823
DICER1	Dicer 1, Ribonuclease III	69.69965
MIR143	MicroRNA 143	67.94745
POLD1	DNA Polymerase Delta 1, Catalytic Subunit	67.61906
MIR203A	MicroRNA 203a	66.62958
TGFBR2	Transforming Growth Factor Beta Receptor 2	65.59235
CCND1	Cyclin D1	64.88373
MIR17	MicroRNA 17	64.70063
MIR126	MicroRNA 126	64.44769
MIR27A	MicroRNA 27a	64.27605
CASP8	Caspase 8	62.84025
ESR1	Estrogen Receptor 1	62.53878
NRAS	NRAS Proto-Oncogene, GTPase	62.31985
RAD50	RAD50 Double Strand Break Repair Protein	62.19757
EPCAM	Epithelial Cell Adhesion Molecule	61.74236
SMARCA4	SWI/SNF Related, Matrix Associated, Actin Dependent Regulator Of Chromatin, Subfamily A, Member 4	61.71597
MIR146A	MicroRNA 146a	61.22657
IRF1	Interferon Regulatory Factor 1	60.77074
MIR93	MicroRNA 93	59.98841
HRAS	HRas Proto-Oncogene, GTPase	59.91721
MIR106B	MicroRNA 106b	59.54659
BMPR1A	Bone Morphogenetic Protein Receptor Type 1A	59.49880
KIT	KIT Proto-Oncogene, Receptor Tyrosine Kinase	59.37038
KLF6	Kruppel Like Factor 6	59.32110
MIR451A	MicroRNA 451a	58.57856
MIR200B	MicroRNA 200b	57.57771
PTCH1	Patched 1	56.96302
MIR125A	MicroRNA 125a	56.68200
CDKN1B	Cyclin Dependent Kinase Inhibitor 1B	56.41122
FGFR3	Fibroblast Growth Factor Receptor 3	55.92256
MRE11	MRE11 Homolog, Double Strand Break Repair Nuclease	55.10709
AR	Androgen Receptor	54.78814
MIR205	MicroRNA 205	54.44110
CDK4	Cyclin Dependent Kinase 4	54.37112

TERT	Telomerase Reverse Transcriptase	54.14210
MIR25	MicroRNA 25	53.85631
TSC2	TSC Complex Subunit 2	53.68873
BAX	BCL2 Associated X, Apoptosis Regulator	53.06767
MIR192	MicroRNA 192	52.86935
MIR155	MicroRNA 155	52.66563
BLM	BLM RecQ Like Helicase	52.32129
MIR200A	MicroRNA 200a	52.26886
XRCC2	X-Ray Repair Cross Complementing 2	52.17210
FANCC	FA Complementation Group C	51.85302
SDHB	Succinate Dehydrogenase Complex Iron Sulfur Subunit B	51.55140
MSH3	MutS Homolog 3	51.48293
MLH3	MutL Homolog 3	51.42154
MAP2K1	Mitogen-Activated Protein Kinase Kinase 1	51.41180
TSC1	TSC Complex Subunit 1	50.56140
MDM2	MDM2 Proto-Oncogene	50.28489
MIR34C	MicroRNA 34c	50.06559
MIR31	MicroRNA 31	49.58886
MEN1	Menin 1	49.48357
CASP10	Caspase 10	49.26771
MIR122	MicroRNA 122	49.24193
RAD51	RAD51 Recombinase	48.99364
BAP1	BRCA1 Associated Protein 1	48.88947
MIR127	MicroRNA 127	48.26155
ALK	ALK Receptor Tyrosine Kinase	48.20362
FASLG	Fas Ligand	47.81522
FLCN	Folliculin	47.27985
MIR15A	MicroRNA 15a	47.06211
MIR215	MicroRNA 215	47.01202
EGF	Epidermal Growth Factor	46.78460
MIR150	MicroRNA 150	46.66998
FH	Fumarate Hydratase	46.54221
IGF2	Insulin Like Growth Factor 2	46.52563
SDHD	Succinate Dehydrogenase Complex Subunit D	46.16196
EP300	E1A Binding Protein P300	46.06517
MAP3K6	Mitogen-Activated Protein Kinase Kinase Kinase 6	45.99519
MIR15B	MicroRNA 15b	45.82567
MYC	MYC Proto-Oncogene, BHLH Transcription Factor	45.66581
MIR34B	MicroRNA 34b	45.49993
SMARCB1	SWI/SNF Related, Matrix Associated, Actin Dependent Regulator Of Chromatin, Subfamily B, Member 1	45.31979
VEGFA	Vascular Endothelial Growth Factor A	45.12320

AURKA	Aurora Kinase A	45.10878
MIR210	MicroRNA 210	44.89948
MIR10B	MicroRNA 10b	44.87817
MIR373	MicroRNA 373	44.76119
VHL	Von Hippel-Lindau Tumor Suppressor	44.42033
XRCC3	X-Ray Repair Cross Complementing 3	44.27421
IL6	Interleukin 6	43.78212
MIR30E	MicroRNA 30e	43.60668
SDHC	Succinate Dehydrogenase Complex Subunit C	43.59707
PPARG	Peroxisome Proliferator Activated Receptor Gamma	43.33532
SRC	SRC Proto-Oncogene, Non-Receptor Tyrosine Kinase	42.98079
MIR214	MicroRNA 214	42.89107
MIR223	MicroRNA 223	42.86693
PIK3R1	Phosphoinositide-3-Kinase Regulatory Subunit 1	42.69799
TNF	Tumor Necrosis Factor	42.52522
MIR29A	MicroRNA 29a	42.09545
SDHA	Succinate Dehydrogenase Complex Flavoprotein Subunit A	41.96544
BCL10	BCL10 Immune Signaling Adaptor	41.96244
DCC	DCC Netrin 1 Receptor	41.92966
MIR29C	MicroRNA 29c	41.91791
STAT3	Signal Transducer And Activator Of Transcription 3	41.65553
MIR196B	MicroRNA 196b	41.46423
MIRLET7C	MicroRNA Let-7c	41.45618
PTGS2	Prostaglandin-Endoperoxide Synthase 2	41.22626
PTPN11	Protein Tyrosine Phosphatase Non-Receptor Type 11	41.20174
CDC73	Cell Division Cycle 73	40.58453
ARID1A	AT-Rich Interaction Domain 1A	40.58173
MIR148A	MicroRNA 148a	40.50044
ABRAXAS1	Abraxas 1, BRCA1 A Complex Subunit	40.29498
ZFHX3	Zinc Finger Homeobox 3	39.70560
MTOR	Mechanistic Target Of Rapamycin Kinase	39.69067
PMS1	PMS1 Homolog 1, Mismatch Repair System Component	39.14163
ERCC6	ERCC Excision Repair 6, Chromatin Remodeling Factor	38.96574
MIR429	MicroRNA 429	38.95093
MIR96	MicroRNA 96	38.65808
MIRLET7D	MicroRNA Let-7d	38.61024
MIR18A	MicroRNA 18a	38.59803
FBXW7	F-Box And WD Repeat Domain Containing 7	38.28791

TGFB1	Transforming Growth Factor Beta 1	38.13654
CDKN1A	Cyclin Dependent Kinase Inhibitor 1A	38.05436
MIR182	MicroRNA 182	37.97836
MIR19A	MicroRNA 19a	37.87105
PDGFRA	Platelet Derived Growth Factor Receptor Alpha	37.82620
MIR106A	MicroRNA 106a	37.48164
MIRLET7G	MicroRNA Let-7g	37.35225
ROS1	ROS Proto-Oncogene 1, Receptor Tyrosine Kinase	37.32583
DLEC1	DLEC1 Cilia And Flagella Associated Protein	37.25860
GSTM1	Glutathione S-Transferase Mu 1	37.13505
SLC22A18	Solute Carrier Family 22 Member 18	36.89343
MIR107	MicroRNA 107	36.70359
MIR183	MicroRNA 183	36.68717
BCL2	BCL2 Apoptosis Regulator	36.65810
MMP9	Matrix Metallopeptidase 9	36.53144
MUC1	Mucin 1, Cell Surface Associated	36.47073
ERCC2	ERCC Excision Repair 2, TFIIH Core Complex Helicase Subunit	36.43175
PRKN	Parkin RBR E3 Ubiquitin Protein Ligase	36.33903
FGFR4	Fibroblast Growth Factor Receptor 4	36.29834
GSTP1	Glutathione S-Transferase Pi 1	36.26314
MIR23B	MicroRNA 23b	36.13396
GAST	Gastrin	36.09293
PPM1D	Protein Phosphatase, Mg2+/Mn2+ Dependent 1D	36.08588
EPHB2	EPH Receptor B2	36.02847
LRRC56	Leucine Rich Repeat Containing 56	35.93671
MIR140	MicroRNA 140	35.83157
MIR335	MicroRNA 335	35.74971
MIR16-1	MicroRNA 16-1	35.61163
MIR204	MicroRNA 204	35.48037
CXCL8	C-X-C Motif Chemokine Ligand 8	35.37512
ERCC1	ERCC Excision Repair 1, Endonuclease Non-Catalytic Subunit	35.29079
EGFR-AS1	EGFR Antisense RNA 1	35.01124
RNASEL	Ribonuclease L	34.93192
SUFU	SUFU Negative Regulator Of Hedgehog Signaling	34.91922
HIF1A	Hypoxia Inducible Factor 1 Subunit Alpha	34.86561
WWOX	WW Domain Containing Oxidoreductase	34.86047
MIR499A	MicroRNA 499a	34.80113
BUB1B	BUB1 Mitotic Checkpoint Serine/Threonine Kinase B	34.76058
MIRLET7B	MicroRNA Let-7b	34.65150

CASP3	Caspase 3	34.61523
MMP2	Matrix Metallopeptidase 2	34.39545
MIR130A	MicroRNA 130a	34.05116
FANCM	FA Complementation Group M	33.95774
MAPK1	Mitogen-Activated Protein Kinase 1	33.82357
HNF1B	HNF1 Homeobox B	33.81010
H19	H19 Imprinted Maternally Expressed Transcript	33.77155
TFF1	Trefoil Factor 1	33.69397
MSR1	Macrophage Scavenger Receptor 1	33.67422
ABCB1	ATP Binding Cassette Subfamily B Member 1	33.57090
MTHFR	Methylenetetrahydrofolate Reductase	33.49448
MUC6	Mucin 6, Oligomeric Mucus/Gel-Forming	33.28987
KLLN	Killin, P53 Regulated DNA Replication Inhibitor	33.27075
MIR100	MicroRNA 100	33.21288
MCC	MCC Regulator Of WNT Signaling Pathway	33.08290
BUB1	BUB1 Mitotic Checkpoint Serine/Threonine Kinase	33.02299
JAK2	Janus Kinase 2	33.01109
MIR486-1	MicroRNA 486-1	32.98858
SOX9	SRY-Box Transcription Factor 9	32.81243
MIR146B	MicroRNA 146b	32.70439
FAS	Fas Cell Surface Death Receptor	32.67705
BIRC5	Baculoviral IAP Repeat Containing 5	32.63872
SMAD7	SMAD Family Member 7	32.59109
IL10	Interleukin 10	32.45646
MIR185	MicroRNA 185	32.40923
MIR193B	MicroRNA 193b	32.39524
NOTCH1	Notch Receptor 1	32.25543
TLR2	Toll Like Receptor 2	32.20625
MIR483	MicroRNA 483	32.10470
PHB	Prohibitin	32.00045
TP73	Tumor Protein P73	31.97916
IGF1R	Insulin Like Growth Factor 1 Receptor	31.96451
MUC5AC	Mucin 5AC, Oligomeric Mucus/Gel-Forming	31.93814
MIRLET7A1	MicroRNA Let-7a-1	31.87748
PPP2R1B	Protein Phosphatase 2 Scaffold Subunit Abeta	31.82829
GREM1	Gremlin 1, DAN Family BMP Antagonist	31.67880
PSCA	Prostate Stem Cell Antigen	31.66901
XRCC1	X-Ray Repair Cross Complementing 1	31.63739
POT1	Protection Of Telomeres 1	31.60426
RAD54L	RAD54 Like	31.56247

MIR133B	MicroRNA 133b	31.55103
MT-CYB	Mitochondrially Encoded Cytochrome B	31.44494
SMARCE1	SWI/SNF Related, Matrix Associated, Actin Dependent Regulator Of Chromatin, Subfamily E, Member 1	31.09151
DLC1	DLC1 Rho GTPase Activating Protein	31.07331
TYMS	Thymidylate Synthetase	31.00219
CD44	CD44 Molecule (Indian Blood Group)	30.97589
ELAC2	ElaC Ribonuclease Z 2	30.95644
CEACAM5	CEA Cell Adhesion Molecule 5	30.94503
FGFR1	Fibroblast Growth Factor Receptor 1	30.85196
MGMT	O-6-Methylguanine-DNA Methyltransferase	30.80603
MIR206	MicroRNA 206	30.74110
MIR137	MicroRNA 137	30.74017
ESR2	Estrogen Receptor 2	30.72054
MIR128-2	MicroRNA 128-2	30.70624
FHIT	Fragile Histidine Triad Diadenosine Triphosphatase	30.70069
UCA1	Urothelial Cancer Associated 1	30.51815
JUN	Jun Proto-Oncogene, AP-1 Transcription Factor Subunit	30.46012
MIR196A2	MicroRNA 196a-2	30.43237
AXIN1	Axin 1	30.41698
MIRLET7A3	MicroRNA Let-7a-3	30.31553
MAD1L1	Mitotic Arrest Deficient 1 Like 1	30.05044
GNAS	GNAS Complex Locus	29.97758
MIRLET7E	MicroRNA Let-7e	29.91474
TOP2A	DNA Topoisomerase II Alpha	29.88868
MAX	MYC Associated Factor X	29.83746
TMEM127	Transmembrane Protein 127	29.82213
MIR331	MicroRNA 331	29.76698
MIR22	MicroRNA 22	29.55159
IGF1	Insulin Like Growth Factor 1	29.46155
AOPEP	Aminopeptidase O (Putative)	29.26243
ATP4A	ATPase H+/K+ Transporting Subunit Alpha	29.21485
CD274	CD274 Molecule	29.17456
BCL2L1	BCL2 Like 1	29.15145
FGF2	Fibroblast Growth Factor 2	29.09393
CYP2A6	Cytochrome P450 Family 2 Subfamily A Member 6	29.01654
GACAT2	Gastric Cancer Associated Transcript 2	28.95443
NTHL1	Nth Like DNA Glycosylase 1	28.88826
MIR195	MicroRNA 195	28.81009
MIR26A1	MicroRNA 26a-1	28.79324
SMAD3	SMAD Family Member 3	28.75094

MAP3K1	Mitogen-Activated Protein Kinase Kinase Kinase 1	28.69099
MSMB	Microseminoprotein Beta	28.66306
CXCR4	C-X-C Motif Chemokine Receptor 4	28.62328
ERBB3	Erb-B2 Receptor Tyrosine Kinase 3	28.61432
CDX2	Caudal Type Homeobox 2	28.57933
POLK	DNA Polymerase Kappa	28.50189
RAF1	Raf-1 Proto-Oncogene, Serine/Threonine Kinase	28.46984
TGFA	Transforming Growth Factor Alpha	28.46381
DDB2	Damage Specific DNA Binding Protein 2	28.44911
VEGFC	Vascular Endothelial Growth Factor C	28.42087
HGF	Hepatocyte Growth Factor	28.39530
NFKB1	Nuclear Factor Kappa B Subunit 1	28.33223
NKX2-1	NK2 Homeobox 1	28.20410
MIR342	MicroRNA 342	28.16436
PLAU	Plasminogen Activator, Urokinase	28.12581
RASSF1	Ras Association Domain Family Member 1	28.03919
TERC	Telomerase RNA Component	27.99522
LZTS1	Leucine Zipper Tumor Suppressor 1	27.98529
LOC10730334(	3p25 Von Hippel-Lindau Tumor Suppressor, E3 Ubiquitin Protein Ligase Alu-Mediated Recombination Region	27.94540
PGR	Progesterone Receptor	27.93201
EZH2	Enhancer Of Zeste 2 Polycomb Repressive Complex 2 Subunit	27.92636
MIRLET7I	MicroRNA Let-7i	27.88847
HOTAIR	HOX Transcript Antisense RNA	27.80662
ABCG2	ATP Binding Cassette Subfamily G Member 2 (Junior Blood Group)	27.76030
CCAT1	Colon Cancer Associated Transcript 1	27.73186
GACAT3	Gastric Cancer Associated Transcript 3	27.71692
HMMR	Hyaluronan Mediated Motility Receptor	27.68821
SOD2	Superoxide Dismutase 2	27.55619
TYMP	Thymidine Phosphorylase	27.52563
GALNT12	Polypeptide N-Acetylgalactosaminyltransferase 12	27.49014
GCRG224	Gastric Cancer-Related Gene GCRG224	27.46300
DNMT1	DNA Methyltransferase 1	27.32402
PLA2G2A	Phospholipase A2 Group IIA	27.28148
SP1	Sp1 Transcription Factor	27.16087
SDHAF2	Succinate Dehydrogenase Complex Assembly Factor 2	27.11622
TNFRSF10B	TNF Receptor Superfamily Member 10b	27.11314
MIR10A	MicroRNA 10a	27.03778
GIPR	Gastric Inhibitory Polypeptide Receptor	27.01587

MIR30D	MicroRNA 30d	26.99588
PDGFRL	Platelet Derived Growth Factor Receptor Like	26.78990
CCAT2	Colon Cancer Associated Transcript 2	26.78919
MXI1	MAX Interactor 1, Dimerization Protein	26.65548
CDKN2B	Cyclin Dependent Kinase Inhibitor 2B	26.62749
NQO1	NAD(P)H Quinone Dehydrogenase 1	26.58588
TWIST1	Twist Family BHLH Transcription Factor 1	26.54136
CASP9	Caspase 9	26.50628
MALAT1	Metastasis Associated Lung Adenocarcinoma Transcript 1	26.46283
MIR372	MicroRNA 372	26.44859
PTPRJ	Protein Tyrosine Phosphatase Receptor Type J	26.42940
PDGFRB	Platelet Derived Growth Factor Receptor Beta	26.39726
IGF2R	Insulin Like Growth Factor 2 Receptor	26.37899
INS	Insulin	26.37197
CDKN3	Cyclin Dependent Kinase Inhibitor 3	26.35904
MAP3K8	Mitogen-Activated Protein Kinase Kinase Kinase 8	26.35303
MMP7	Matrix Metallopeptidase 7	26.33115
DPYD	Dihydropyrimidine Dehydrogenase	26.32841
KRT20	Keratin 20	26.28210
SST	Somatostatin	26.12685
PLAUR	Plasminogen Activator, Urokinase Receptor	26.08883
AKT2	AKT Serine/Threonine Kinase 2	26.05550
MIR197	MicroRNA 197	26.01512
GHET1	Gastric Carcinoma Proliferation Enhancing Transcript 1	25.97925
KDR	Kinase Insert Domain Receptor	25.96685
GLI1	GLI Family Zinc Finger 1	25.93936
GSTT1	Glutathione S-Transferase Theta 1	25.92520
WT1	WT1 Transcription Factor	25.88098
ETV6	ETS Variant Transcription Factor 6	25.85451
KRT19	Keratin 19	25.84488
CASC2	Cancer Susceptibility 2	25.77207
MAPK3	Mitogen-Activated Protein Kinase 3	25.76373
RNF43	Ring Finger Protein 43	25.75410
CDK2	Cyclin Dependent Kinase 2	25.74626
NQO2	N-Ribosyldihydronicotinamide:Quinone Reductase 2	25.72255
TGFBR1	Transforming Growth Factor Beta Receptor 1	25.70161
TCF7L2	Transcription Factor 7 Like 2	25.65339
HFE	Homeostatic Iron Regulator	25.64143

CYP1A1	Cytochrome P450 Family 1 Subfamily A Member 1	25.60025
MIR128-1	MicroRNA 128-1	25.54505
ABCC1	ATP Binding Cassette Subfamily C Member 1	25.49612
PRKAR1A	Protein Kinase CAMP-Dependent Type I Regulatory Subunit Alpha	25.49080
MIR181A1	MicroRNA 181a-1	25.43617
BIRC3	Baculoviral IAP Repeat Containing 3	25.43212
MMP1	Matrix Metallopeptidase 1	25.32407
RBBP8	RB Binding Protein 8, Endonuclease	25.24870
MIR191	MicroRNA 191	25.18564
MT-CO1	Mitochondrially Encoded Cytochrome C Oxidase I	25.15152
GACAT1	Gastric Cancer Associated Transcript 1	25.14476
CTLA4	Cytotoxic T-Lymphocyte Associated Protein 4	25.10873
IDH1	Isocitrate Dehydrogenase (NADP(+)) 1	25.07231
RHOA	Ras Homolog Family Member A	25.06219
ATR	ATR Serine/Threonine Kinase	25.03276
CTAG1B	Cancer/Testis Antigen 1B	24.98322
ALB	Albumin	24.96321
PCNA	Proliferating Cell Nuclear Antigen	24.90182
RAD54B	RAD54 Homolog B	24.84642
MMP14	Matrix Metallopeptidase 14	24.83905
PCAT1	Prostate Cancer Associated Transcript 1	24.79444
E2F1	E2F Transcription Factor 1	24.76781
RUNX3	RUNX Family Transcription Factor 3	24.74317
GIP	Gastric Inhibitory Polypeptide	24.73208
KRT7	Keratin 7	24.70899
MIR23A	MicroRNA 23a	24.64401
HRH2	Histamine Receptor H2	24.61287
IDH2	Isocitrate Dehydrogenase (NADP(+)) 2	24.58613
S100A8	S100 Calcium Binding Protein A8	24.54441
YAP1	Yes1 Associated Transcriptional Regulator	24.48214
FRGCA	FOXM1-Regulated, Gastric Cancer Associated	24.39876
SMAD2	SMAD Family Member 2	24.38498
CDK6	Cyclin Dependent Kinase 6	24.37347
HERC2	HECT And RLD Domain Containing E3 Ubiquitin Protein Ligase 2	24.34949
CYCS	Cytochrome C, Somatic	24.23504
MIR9-3	MicroRNA 9-3	24.22942
AFP	Alpha Fetoprotein	24.19831
LOC10050734(	Uncharacterized LOC100507346	24.19373
MITF	Melanocyte Inducing Transcription Factor	24.15265
ITGB1	Integrin Subunit Beta 1	24.13839

OGG1	8-Oxoguanine DNA Glycosylase	24.08091
CHGA	Chromogranin A	24.07161
SNAI1	Snail Family Transcriptional Repressor 1	24.06277
PTK2	Protein Tyrosine Kinase 2	24.03134
SLC2A1	Solute Carrier Family 2 Member 1	24.02702
MIR142	MicroRNA 142	24.02207
DMD	Dystrophin	23.99386
SOX2	SRY-Box Transcription Factor 2	23.99109
RPS6KB1	Ribosomal Protein S6 Kinase B1	23.96792
CCNE1	Cyclin E1	23.92054
RUNX1	RUNX Family Transcription Factor 1	23.90772
FLT4	Fms Related Receptor Tyrosine Kinase 4	23.86911
PTPN12	Protein Tyrosine Phosphatase Non-Receptor Type 12	23.85639
IL2	Interleukin 2	23.84788
OPCML	Opioid Binding Protein/Cell Adhesion Molecule Like	23.64778
MIR16-2	MicroRNA 16-2	23.63934
MIR9-1	MicroRNA 9-1	23.56673
PVT1	Pvt1 Oncogene	23.56225
DROSHA	Drosha Ribonuclease III	23.54187
MEG3	Maternally Expressed 3	23.46421
NFKBIA	NFKB Inhibitor Alpha	23.45377
RNF6	Ring Finger Protein 6	23.44416
MIR199A1	MicroRNA 199a-1	23.43831
MAPK8	Mitogen-Activated Protein Kinase 8	23.40841
MIR224	MicroRNA 224	23.39703
LIPF	Lipase F, Gastric Type	23.32012
CCKBR	Cholecystokinin B Receptor	23.30317
RARB	Retinoic Acid Receptor Beta	23.29824
FOS	Fos Proto-Oncogene, AP-1 Transcription Factor Subunit	23.26982
MIR449A	MicroRNA 449a	23.22964
NF2	Neurofibromin 2	23.19262
FZD3	Frizzled Class Receptor 3	23.19250
CRNN	Cornulin	23.17194
ING1	Inhibitor Of Growth Family Member 1	23.16738
ERCC4	ERCC Excision Repair 4, Endonuclease Catalytic Subunit	23.11658
MIR24-2	MicroRNA 24-2	23.10940
MIR502	MicroRNA 502	23.09302
NFE2L2	Nuclear Factor, Erythroid 2 Like 2	23.07315
MUC2	Mucin 2, Oligomeric Mucus/Gel-Forming	23.05596
PRKCA	Protein Kinase C Alpha	23.04539
CBLIF	Cobalamin Binding Intrinsic Factor	23.00758
GHRL	Ghrelin And Obestatin Prepropeptide	22.95441
CXCL12	C-X-C Motif Chemokine Ligand 12	22.92655

ABL1	ABL Proto-Oncogene 1, Non-Receptor Tyrosine Kinase	22.92217
KRT18	Keratin 18	22.91938
PLK1	Polo Like Kinase 1	22.88639
NCOA3	Nuclear Receptor Coactivator 3	22.88158
IGFBP3	Insulin Like Growth Factor Binding Protein 3	22.83597
PKHD1	PKHD1 Ciliary IPT Domain Containing Fibrocystin/Polyductin	22.81973
MIR661	MicroRNA 661	22.72309
GSK3B	Glycogen Synthase Kinase 3 Beta	22.69831
BCAR4	Breast Cancer Anti-Estrogen Resistance 4	22.62740
KLK3	Kallikrein Related Peptidase 3	22.55065
NME1	NME/NM23 Nucleoside Diphosphate Kinase 1	22.54480
BAK1	BCL2 Antagonist/Killer 1	22.52220
ATRX	ATRX Chromatin Remodeler	22.50161
ACTB	Actin Beta	22.48902
SETD2	SET Domain Containing 2, Histone Lysine Methyltransferase	22.47181
TIMP1	TIMP Metallopeptidase Inhibitor 1	22.45944
ATP12A	ATPase H+/K+ Transporting Non-Gastric Alpha2 Subunit	22.40010
THBS1	Thrombospondin 1	22.37415
CCNB1	Cyclin B1	22.32962
IFNG	Interferon Gamma	22.29906
CDKN2B-AS1	CDKN2B Antisense RNA 1	22.27972
WRAP53	WD Repeat Containing Antisense To TP53	22.22433
CYP19A1	Cytochrome P450 Family 19 Subfamily A Member 1	22.19539
PARP1	Poly(ADP-Ribose) Polymerase 1	22.12415
SERPINA3	Serpin Family A Member 3	22.11530
TFF2	Trefoil Factor 2	22.07464
MT-ND4L	Mitochondrially Encoded NADH:Ubiquinone Oxidoreductase Core Subunit 4L	22.03687
AMER1	APC Membrane Recruitment Protein 1	22.03289
CTNND1	Catenin Delta 1	21.97417
NTRK1	Neurotrophic Receptor Tyrosine Kinase 1	21.96473
AREG	Amphiregulin	21.96242
TIMP3	TIMP Metallopeptidase Inhibitor 3	21.89876
CCK	Cholecystokinin	21.89318
PROM1	Prominin 1	21.88833
DNMT3A	DNA Methyltransferase 3 Alpha	21.82908
MYCN	MYCN Proto-Oncogene, BHLH Transcription Factor	21.82498

E2F3	E2F Transcription Factor 3	21.81514
MIR181A2	MicroRNA 181a-2	21.80817
CCND2	Cyclin D2	21.77503
SHH	Sonic Hedgehog Signaling Molecule	21.75231
CDK1	Cyclin Dependent Kinase 1	21.72514
STAT1	Signal Transducer And Activator Of Transcription 1	21.67723
DNMT3B	DNA Methyltransferase 3 Beta	21.66596
MUC16	Mucin 16, Cell Surface Associated	21.60529
NEAT1	Nuclear Paraspeckle Assembly Transcript 1	21.55377
SPP1	Secreted Phosphoprotein 1	21.51229
TIMP2	TIMP Metallopeptidase Inhibitor 2	21.48973
GNAQ	G Protein Subunit Alpha Q	21.41842
MIR181B1	MicroRNA 181b-1	21.38670
TLR4	Toll Like Receptor 4	21.34876
BLACAT1	Bladder Cancer Associated Transcript 1	21.34197
TNFSF10	TNF Superfamily Member 10	21.26667
TGFB2	Transforming Growth Factor Beta 2	21.25217
EPHA2	EPH Receptor A2	21.24540
SERPINB5	Serpin Family B Member 5	21.21421
ENG	Endoglin	21.19773
NOS2	Nitric Oxide Synthase 2	21.19760
TP63	Tumor Protein P63	21.14117
MKI67	Marker Of Proliferation Ki-67	21.12648
GAS5	Growth Arrest Specific 5	21.08851
TACC1	Transforming Acidic Coiled-Coil Containing Protein 1	21.06743
ENO2	Enolase 2	20.99028
HBEGF	Heparin Binding EGF Like Growth Factor	20.89133
PDCD1	Programmed Cell Death 1	20.86130
LEP	Leptin	20.84268
FN1	Fibronectin 1	20.84187
CTAG2	Cancer/Testis Antigen 2	20.83091
XPA	XPA, DNA Damage Recognition And Repair Factor	20.80423
MIR375	MicroRNA 375	20.77451
MCL1	MCL1 Apoptosis Regulator, BCL2 Family Member	20.72603
BAD	BCL2 Associated Agonist Of Cell Death	20.70117
MUC4	Mucin 4, Cell Surface Associated	20.69833
GAPLINC	Gastric Adenocarcinoma Associated, Positive CD44 Regulator, Long Intergenic Non-Coding RNA	20.68561
MAPK14	Mitogen-Activated Protein Kinase 14	20.63041
PTGS1	Prostaglandin-Endoperoxide Synthase 1	20.62220
PDGFB	Platelet Derived Growth Factor Subunit B	20.58423

RELA	RELA Proto-Oncogene, NF-KB Subunit	20.47146
TCERG1	Transcription Elongation Regulator 1	20.44027
ZEB1	Zinc Finger E-Box Binding Homeobox 1	20.42673
DAPK1	Death Associated Protein Kinase 1	20.41740
XIAP	X-Linked Inhibitor Of Apoptosis	20.41429
SHC1	SHC Adaptor Protein 1	20.34510
ODC1	Ornithine Decarboxylase 1	20.32011
CDH3	Cadherin 3	20.28876
PIK3CB	Phosphatidylinositol-4,5-Bisphosphate 3-Kinase Catalytic Subunit Beta	20.28790
ANXA5	Annexin A5	20.26839
CCR6	C-C Motif Chemokine Receptor 6	20.20862
VDR	Vitamin D Receptor	20.20082
TUG1	Taurine Up-Regulated 1	20.18530
WNT5A	Wnt Family Member 5A	20.18349
XPC	XPC Complex Subunit, DNA Damage Recognition And Repair Factor	20.11114
CCNA2	Cyclin A2	20.10189
GRP	Gastrin Releasing Peptide	20.03610
S100A4	S100 Calcium Binding Protein A4	20.03368
CAV1	Caveolin 1	19.98613
ERCC5	ERCC Excision Repair 5, Endonuclease	19.96596
FBN1	Fibrillin 1	19.86678
KCNQ1OT1	KCNQ1 Opposite Strand/Antisense Transcript 1	19.84855
SNAI2	Snail Family Transcriptional Repressor 2	19.79506
WNT1	Wnt Family Member 1	19.75189
HSPB1	Heat Shock Protein Family B (Small) Member 1	19.73990
PAK1	P21 (RAC1) Activated Kinase 1	19.69873
VEGFD	Vascular Endothelial Growth Factor D	19.68064
PRKCD	Protein Kinase C Delta	19.63384
FOXP3	Forkhead Box P3	19.62535
GNA11	G Protein Subunit Alpha 11	19.62394
CDH17	Cadherin 17	19.58390
SKP2	S-Phase Kinase Associated Protein 2	19.57195
SYP	Synaptophysin	19.55273
PRNCR1	Prostate Cancer Associated Non-Coding RNA 1	19.43678
CD34	CD34 Molecule	19.41885
GRB2	Growth Factor Receptor Bound Protein 2	19.36637
CREBBP	CREB Binding Protein	19.35955
INS-IGF2	INS-IGF2 Readthrough	19.33957
CDH2	Cadherin 2	19.33357
ERG	ETS Transcription Factor ERG	19.32980
TOE1	Target Of EGR1, Exonuclease	19.32109

GKN2	Gastrokine 2	19.29385
CCL2	C-C Motif Chemokine Ligand 2	19.28397
RIPK1	Receptor Interacting Serine/Threonine Kinase 1	19.25045
CDKN1C	Cyclin Dependent Kinase Inhibitor 1C	19.24394
GCG	Glucagon	19.15897
CYP2E1	Cytochrome P450 Family 2 Subfamily E Member 1	19.14542
MAGEA3	MAGE Family Member A3	19.13424
HSP90AA1	Heat Shock Protein 90 Alpha Family Class A Member 1	19.12333
MAGEA1	MAGE Family Member A1	19.11769
CHEK1	Checkpoint Kinase 1	19.11376
CASC9	Cancer Susceptibility 9	19.10401
HABP2	Hyaluronan Binding Protein 2	19.09752
HPSE	Heparanase	19.08511
IL4	Interleukin 4	19.06816
NAT2	N-Acetyltransferase 2	19.06662
MALT1	MALT1 Paracaspase	19.06254
XIST	X Inactive Specific Transcript	19.00464
RARA	Retinoic Acid Receptor Alpha	18.99352
MIR139	MicroRNA 139	18.96016
KLF4	Kruppel Like Factor 4	18.94616
LEF1	Lymphoid Enhancer Binding Factor 1	18.93776
MIR30A	MicroRNA 30a	18.93453
KRT8	Keratin 8	18.92171
MIR149	MicroRNA 149	18.86218
FLT1	Fms Related Receptor Tyrosine Kinase 1	18.83930
RAC1	Rac Family Small GTPase 1	18.83851
CD82	CD82 Molecule	18.79424
CRP	C-Reactive Protein	18.74450
MIR181C	MicroRNA 181c	18.73177
CA9	Carbonic Anhydrase 9	18.66933
CSF3	Colony Stimulating Factor 3	18.63679
FGF7	Fibroblast Growth Factor 7	18.63074
BMI1	BMI1 Proto-Oncogene, Polycomb Ring Finger	18.62917
HULC	Hepatocellular Carcinoma Up-Regulated Long Non-Coding RNA	18.60291
AKT3	AKT Serine/Threonine Kinase 3	18.59911
CAGE1	Cancer Antigen 1	18.58529
CSF2	Colony Stimulating Factor 2	18.57967
HDAC1	Histone Deacetylase 1	18.54943
TOP1	DNA Topoisomerase I	18.51686
ERBB4	Erb-B2 Receptor Tyrosine Kinase 4	18.51637
RHBDF2	Rhomboid 5 Homolog 2	18.48975
MTUS1	Microtubule Associated Scaffold Protein 1	18.48529
H2AC18	H2A Clustered Histone 18	18.46419

VIM	Vimentin	18.42975
PIK3R3	Phosphoinositide-3-Kinase Regulatory Subunit 3	18.41945
FOXP1	Forkhead Box P1	18.39923
CTSB	Cathepsin B	18.34393
FGF1	Fibroblast Growth Factor 1	18.33145
ITGA6	Integrin Subunit Alpha 6	18.27433
TRIP13	Thyroid Hormone Receptor Interactor 13	18.26993
MAP2K2	Mitogen-Activated Protein Kinase Kinase 2	18.25297
CYP3A4	Cytochrome P450 Family 3 Subfamily A Member 4	18.19470
CREB1	CAMP Responsive Element Binding Protein 1	18.15995
KEAP1	Kelch Like ECH Associated Protein 1	18.15902
E2F2	E2F Transcription Factor 2	18.13805
CYP1B1	Cytochrome P450 Family 1 Subfamily B Member 1	18.03577
DHFR	Dihydrofolate Reductase	17.99301
HMOX1	Heme Oxygenase 1	17.98318
PIK3CG	Phosphatidylinositol-4,5-Bisphosphate 3-Kinase Catalytic Subunit Gamma	17.97287
CYP17A1	Cytochrome P450 Family 17 Subfamily A Member 1	17.90142
PBOV1	Prostate And Breast Cancer Overexpressed 1	17.89511
NOTCH2	Notch Receptor 2	17.88105
TPX2	TPX2 Microtubule Nucleation Factor	17.86369
JUP	Junction Plakoglobin	17.85021
HOTTIP	HOXA Distal Transcript Antisense RNA	17.80014
SPINK1	Serine Peptidase Inhibitor Kazal Type 1	17.72987
SMO	Smoothened, Frizzled Class Receptor	17.72617
GKN1	Gastrokine 1	17.71817
PDPN	Podoplanin	17.71451
ACVR1B	Activin A Receptor Type 1B	17.71225
PLCG1	Phospholipase C Gamma 1	17.71036
MIR29B1	MicroRNA 29b-1	17.71012
CYP2D6	Cytochrome P450 Family 2 Subfamily D Member 6	17.70926
JAG1	Jagged Canonical Notch Ligand 1	17.69780
HSPA5	Heat Shock Protein Family A (Hsp70) Member 5	17.64622
FOXO1	Forkhead Box O1	17.62543
MTDH	Metadherin	17.59744
FOXE1	Forkhead Box E1	17.55455
ALDH2	Aldehyde Dehydrogenase 2 Family Member	17.55005
EZR	Ezrin	17.53443

POU5F1	POU Class 5 Homeobox 1	17.53162
ABCC2	ATP Binding Cassette Subfamily C Member 2	17.51327
LGALS3	Galectin 3	17.48903
LINC-ROR	Long Intergenic Non-Protein Coding RNA, Regulator Of Reprogramming	17.45902
ZEB2	Zinc Finger E-Box Binding Homeobox 2	17.43855
HMGB1	High Mobility Group Box 1	17.43805
DRAIC	Downregulated RNA In Cancer, Inhibitor Of Cell Invasion And Migration	17.43249
SPRY4-IT1	SPRY4 Intronic Transcript 1	17.40322
PCAT29	Prostate Cancer Associated Transcript 29	17.35259
RXRA	Retinoid X Receptor Alpha	17.34916
CYTOR	Cytoskeleton Regulator RNA	17.33641
CTSD	Cathepsin D	17.31660
ZFAS1	ZNFX1 Antisense RNA 1	17.29870
FGF4	Fibroblast Growth Factor 4	17.28346
WRN	WRN RecQ Like Helicase	17.27948
EIF4EBP1	Eukaryotic Translation Initiation Factor 4E Binding Protein 1	17.25987
MIR193A	MicroRNA 193a	17.20937
ECT2	Epithelial Cell Transforming 2	17.20595
CASC15	Cancer Susceptibility 15	17.20330
ETS1	ETS Proto-Oncogene 1, Transcription Factor	17.19114
HLA-DQB1	Major Histocompatibility Complex, Class II, DQ Beta 1	17.18989
MIR320A	MicroRNA 320a	17.14877
NOTCH3	Notch Receptor 3	17.14856
CLDN4	Claudin 4	17.14816
MIR196A1	MicroRNA 196a-1	17.14206
COMT	Catechol-O-Methyltransferase	17.09802
TNFSF11	TNF Superfamily Member 11	17.08738
BMP2	Bone Morphogenetic Protein 2	17.07948
SCT	Secretin	17.05582
CBL	Cbl Proto-Oncogene	17.05461
SOS1	SOS Ras/Rac Guanine Nucleotide Exchange Factor 1	17.05403
CFLAR	CASP8 And FADD Like Apoptosis Regulator	16.97515
FASN	Fatty Acid Synthase	16.96352
FANCI	FA Complementation Group I	16.95593
GPER1	G Protein-Coupled Estrogen Receptor 1	16.95567
GPC3	Glypican 3	16.95296
LUCAT1	Lung Cancer Associated Transcript 1	16.93672

GADD45A	Growth Arrest And DNA Damage Inducible Alpha	16.92664
MMP3	Matrix Metallopeptidase 3	16.91387
GCAWKR	Gastric Cancer Associated WDR5 And KAT2A Binding LncRNA	16.89718
EXT2	Exostosin Glycosyltransferase 2	16.86050
NPM1	Nucleophosmin 1	16.85288
CD24	CD24 Molecule	16.83959
MIR152	MicroRNA 152	16.83779
COLCA2	Colorectal Cancer Associated 2	16.83545
CALCA	Calcitonin Related Polypeptide Alpha	16.83385
MIR144	MicroRNA 144	16.82997
MIR124-1	MicroRNA 124-1	16.82605
GJA1	Gap Junction Protein Alpha 1	16.80597
PDCD4	Programmed Cell Death 4	16.79137
FOXM1	Forkhead Box M1	16.72980
MIR424	MicroRNA 424	16.67923
FLT3	Fms Related Receptor Tyrosine Kinase 3	16.67024
FSCN1	Fascin Actin-Bundling Protein 1	16.67011
MIR338	MicroRNA 338	16.58604
TNFRSF10A	TNF Receptor Superfamily Member 10a	16.55409
S100A6	S100 Calcium Binding Protein A6	16.52754
PTHLH	Parathyroid Hormone Like Hormone	16.52588
IL17A	Interleukin 17A	16.52148
SNHG1	Small Nucleolar RNA Host Gene 1	16.51118
EWSR1	EWS RNA Binding Protein 1	16.39265
SPARC	Secreted Protein Acidic And Cysteine Rich	16.38902
IFI27	Interferon Alpha Inducible Protein 27	16.38832
HLA-DRB1	Major Histocompatibility Complex, Class II, DR Beta 1	16.37851
PGA3	Pepsinogen A3	16.37070
PAX5	Paired Box 5	16.35685
BMP6	Bone Morphogenetic Protein 6	16.34997
IFNA2	Interferon Alpha 2	16.31798
NDRG1	N-Myc Downstream Regulated 1	16.27115
CD40	CD40 Molecule	16.27036
MPO	Myeloperoxidase	16.25173
EBAG9	Estrogen Receptor Binding Site Associated Antigen 9	16.23826
HSPA4	Heat Shock Protein Family A (Hsp70) Member 4	16.18148
KLF5	Kruppel Like Factor 5	16.13105
TRIM28	Tripartite Motif Containing 28	16.11224
B2M	Beta-2-Microglobulin	16.09968
SRGAP1	SLIT-ROBO Rho GTPase Activating Protein 1	16.08655
TFF3	Trefoil Factor 3	16.08406
CCNE2	Cyclin E2	16.07646

WNT6	Wnt Family Member 6	16.07441
LOC10678082[illegible]	FOXM1-Regulated, Gastric Cancer Associated-Like	16.04230
LOC10050632[illegible]	Uncharacterized LOC100506321	16.03884
COLCA1	Colorectal Cancer Associated 1	16.03462
FOXO3	Forkhead Box O3	16.03060
BCL6	BCL6 Transcription Repressor	15.99702
DKK1	Dickkopf WNT Signaling Pathway Inhibitor 1	15.98963
ITGAV	Integrin Subunit Alpha V	15.98305
IQGAP1	IQ Motif Containing GTPase Activating Protein 1	15.94286
IL7	Interleukin 7	15.93747
BCAR1	BCAR1 Scaffold Protein, Cas Family Member	15.92646
NTRK3	Neurotrophic Receptor Tyrosine Kinase 3	15.92537
NCAM1	Neural Cell Adhesion Molecule 1	15.90935
CASC8	Cancer Susceptibility 8	15.90093
WNT2B	Wnt Family Member 2B	15.87572
WNT3	Wnt Family Member 3	15.82843
MIR24-1	MicroRNA 24-1	15.82607
SERPINE1	Serpin Family E Member 1	15.81923
EFNA1	Ephrin A1	15.81340
GRB7	Growth Factor Receptor Bound Protein 7	15.79026
PIK3R2	Phosphoinositide-3-Kinase Regulatory Subunit 2	15.77635
MIR27B	MicroRNA 27b	15.75286
CLDN3	Claudin 3	15.71804
MIR423	MicroRNA 423	15.70811
PGA4	Pepsinogen A4	15.70692
PTENP1	Phosphatase And Tensin Homolog Pseudogene 1	15.68919
SNHG16	Small Nucleolar RNA Host Gene 16	15.67711
HDAC9	Histone Deacetylase 9	15.67108
IL1A	Interleukin 1 Alpha	15.66386
CT83	Cancer/Testis Antigen 83	15.66373
MDM4	MDM4 Regulator Of P53	15.62123
BCAR3	BCAR3 Adaptor Protein, NSP Family Member	15.62074
CYP2C19	Cytochrome P450 Family 2 Subfamily C Member 19	15.61108
BCL2L11	BCL2 Like 11	15.61019
ICOSLG	Inducible T Cell Costimulator Ligand	15.60905
PAX8	Paired Box 8	15.59476
DANCR	Differentiation Antagonizing Non-Protein Coding RNA	15.56455
IL2RA	Interleukin 2 Receptor Subunit Alpha	15.55701

RECQL4	RecQ Like Helicase 4	15.55176
TET2	Tet Methylcytosine Dioxygenase 2	15.54951
PGA5	Pepsinogen A5	15.54160
ANXA2	Annexin A2	15.53710
CCND3	Cyclin D3	15.52474
FANCD2	FA Complementation Group D2	15.48984
SOX4	SRY-Box Transcription Factor 4	15.48320
LRP5	LDL Receptor Related Protein 5	15.45702
MTA1	Metastasis Associated 1	15.44892
JAK1	Janus Kinase 1	15.43766
BMP4	Bone Morphogenetic Protein 4	15.43498
STMN1	Stathmin 1	15.41204
CALR	Calreticulin	15.38544
PTPRC	Protein Tyrosine Phosphatase Receptor Type C	15.35784
BECN1	Beclin 1	15.35078
RAD51B	RAD51 Paralog B	15.35056
CLDN7	Claudin 7	15.34329
HLA-G	Major Histocompatibility Complex, Class I, G	15.32708
U2AF1	U2 Small Nuclear RNA Auxiliary Factor 1	15.30508
FENDRR	FOXF1 Adjacent Non-Coding Developmental Regulatory RNA	15.30221
IRS1	Insulin Receptor Substrate 1	15.28939
HNF4A	Hepatocyte Nuclear Factor 4 Alpha	15.24994
AXL	AXL Receptor Tyrosine Kinase	15.23123
KDM4C	Lysine Demethylase 4C	15.22997
YBX1	Y-Box Binding Protein 1	15.21928
CCL5	C-C Motif Chemokine Ligand 5	15.20982
STAT5B	Signal Transducer And Activator Of Transcription 5B	15.20889
LINC00673	Long Intergenic Non-Protein Coding RNA 673	15.19471
GATA3	GATA Binding Protein 3	15.18125
HOXA11-AS	HOXA11 Antisense RNA	15.17955
WNT3A	Wnt Family Member 3A	15.17935
NRG1	Neuregulin 1	15.12856
RMRP	RNA Component Of Mitochondrial RNA Processing Endoribonuclease	15.12431
LOX	Lysyl Oxidase	15.08061
WNT10B	Wnt Family Member 10B	15.06117
BSG	Basigin (Ok Blood Group)	15.03493
EGR1	Early Growth Response 1	15.02898
AFAP1-AS1	AFAP1 Antisense RNA 1	15.00990
MME	Membrane Metalloendopeptidase	15.00394
CAPN9	Calpain 9	14.99675
ICAM1	Intercellular Adhesion Molecule 1	14.98272
EPHX1	Epoxide Hydrolase 1	14.97184

LCP1	Lymphocyte Cytosolic Protein 1	14.97180
IKBKB	Inhibitor Of Nuclear Factor Kappa B Kinase Subunit Beta	14.97112
ALDH1A1	Aldehyde Dehydrogenase 1 Family Member A1	14.96629
GAPDH	Glyceraldehyde-3-Phosphate Dehydrogenase	14.95961
CD4	CD4 Molecule	14.95943
ELAVL1	ELAV Like RNA Binding Protein 1	14.94522
KITLG	KIT Ligand	14.92457
MIR532	MicroRNA 532	14.84836
CLDN18	Claudin 18	14.80530
ETV4	ETS Variant Transcription Factor 4	14.79543
IGFBP2	Insulin Like Growth Factor Binding Protein 2	14.79517
GDF15	Growth Differentiation Factor 15	14.78955
CXCL1	C-X-C Motif Chemokine Ligand 1	14.77773
PANDAR	Promoter Of CDKN1A Antisense DNA Damage Activated RNA	14.76752
MIR99A	MicroRNA 99a	14.76689
TMPRSS2	Transmembrane Serine Protease 2	14.74727
IFNA1	Interferon Alpha 1	14.71717
UMPS	Uridine Monophosphate Synthetase	14.70015
KMT2A	Lysine Methyltransferase 2A	14.69931
FANCG	FA Complementation Group G	14.68138
CASP7	Caspase 7	14.67884
MIAT	Myocardial Infarction Associated Transcript	14.67031
MCM4	Minichromosome Maintenance Complex Component 4	14.66902
TLR9	Toll Like Receptor 9	14.66831
HLA-A	Major Histocompatibility Complex, Class I, A	14.66138
LBR	Lamin B Receptor	14.65827
COL1A1	Collagen Type I Alpha 1 Chain	14.60369
SERPINA1	Serpin Family A Member 1	14.60109
DDR2	Discoidin Domain Receptor Tyrosine Kinase 2	14.58452
MIR129-1	MicroRNA 129-1	14.54752
ANXA1	Annexin A1	14.54740
ATAD2	ATPase Family AAA Domain Containing 2	14.53292
CCR7	C-C Motif Chemokine Receptor 7	14.51213
CRNDE	Colorectal Neoplasia Differentially Expressed	14.50481
BANCR	BRAF-Activated Non-Protein Coding RNA	14.49767
CCNA1	Cyclin A1	14.49524
PCAT6	Prostate Cancer Associated Transcript 6	14.49312

PLK2	Polo Like Kinase 2	14.45185
ADIPOQ	Adiponectin, C1Q And Collagen Domain Containing	14.42585
SOX2-OT	SOX2 Overlapping Transcript	14.38728
ARAF	A-Raf Proto-Oncogene, Serine/Threonine Kinase	14.37646
CEACAM6	CEA Cell Adhesion Molecule 6	14.37420
LINC00261	Long Intergenic Non-Protein Coding RNA 261	14.36888
FOLH1	Folate Hydrolase 1	14.36277
NRP1	Neuropilin 1	14.36210
SIRT1	Sirtuin 1	14.35900
LGALS1	Galectin 1	14.35884
FZD7	Frizzled Class Receptor 7	14.35285
REG4	Regenerating Family Member 4	14.34844
MIR324	MicroRNA 324	14.34258
CHUK	Component Of Inhibitor Of Nuclear Factor Kappa B Kinase Complex	14.33987
JAK3	Janus Kinase 3	14.32812
REST	RE1 Silencing Transcription Factor	14.28961
ANGPT2	Angiopoietin 2	14.26971
GATA2	GATA Binding Protein 2	14.23698
RPL15	Ribosomal Protein L15	14.23379
TNFRSF1B	TNF Receptor Superfamily Member 1B	14.22210
IL4R	Interleukin 4 Receptor	14.21889
CIB1	Calcium And Integrin Binding 1	14.21481
APEX1	Apurinic/Apyrimidinic Endodeoxyribonuclease 1	14.19459
MYB	MYB Proto-Oncogene, Transcription Factor	14.18635
CSNK1A1	Casein Kinase 1 Alpha 1	14.18341
KRT5	Keratin 5	14.16923
DELEC1	Deleted In Esophageal Cancer 1	14.16305
HNF1A	HNF1 Homeobox A	14.14215
NFKB2	Nuclear Factor Kappa B Subunit 2	14.08896
WNT2	Wnt Family Member 2	14.07919
PKM	Pyruvate Kinase M1/2	14.06184
CDKN2C	Cyclin Dependent Kinase Inhibitor 2C	14.05923
MACC1	MET Transcriptional Regulator MACC1	14.05282
CXCR2	C-X-C Motif Chemokine Receptor 2	14.03899
MIR30B	MicroRNA 30b	14.02491
CSNK2A1	Casein Kinase 2 Alpha 1	14.00764
ETV1	ETS Variant Transcription Factor 1	14.00525
URGCP	Upregulator Of Cell Proliferation	13.97086
HNF1A-AS1	HNF1A Antisense RNA 1	13.96985
CDX1	Caudal Type Homeobox 1	13.96656
TCF4	Transcription Factor 4	13.96603
SNCG	Synuclein Gamma	13.95875

UGT1A1	UDP Glucuronosyltransferase Family 1 Member A1	13.95820
MMP11	Matrix Metallopeptidase 11	13.93089
TG	Thyroglobulin	13.90227
NAT1	N-Acetyltransferase 1	13.90009
CDC25A	Cell Division Cycle 25A	13.89014
IGH	Immunoglobulin Heavy Locus	13.88999
MYD88	MYD88 Innate Immune Signal Transduction Adaptor	13.88186
SEMA4A	Semaphorin 4A	13.87687
MIR296	MicroRNA 296	13.87466
MIR125B1	MicroRNA 125b-1	13.87320
CSF1	Colony Stimulating Factor 1	13.87093
F2	Coagulation Factor II, Thrombin	13.86943
PRKACA	Protein Kinase CAMP-Activated Catalytic Subunit Alpha	13.86483
HLA-B	Major Histocompatibility Complex, Class I, B	13.85044
SQSTM1	Sequestosome 1	13.81104
MVP	Major Vault Protein	13.81031
SETBP1	SET Binding Protein 1	13.79638
ITGB3	Integrin Subunit Beta 3	13.79136
CYP1A2	Cytochrome P450 Family 1 Subfamily A Member 2	13.78440
LMNB2	Lamin B2	13.77918
LASP1	LIM And SH3 Protein 1	13.76746
KLK10	Kallikrein Related Peptidase 10	13.73941
PRDM2	PR/SET Domain 2	13.72238
EPHB4	EPH Receptor B4	13.70231
TNFRSF1A	TNF Receptor Superfamily Member 1A	13.68843
VIP	Vasoactive Intestinal Peptide	13.68367
STAT5A	Signal Transducer And Activator Of Transcription 5A	13.67932
CTNNA2	Catenin Alpha 2	13.65865
NTS	Neurotensin	13.63096
PML	PML Nuclear Body Scaffold	13.61440
CTTN	Cortactin	13.60546
CDK12	Cyclin Dependent Kinase 12	13.59918
CBFB	Core-Binding Factor Subunit Beta	13.59401
TP53COR1	Tumor Protein P53 Pathway Corepressor 1	13.58720
SOCS3	Suppressor Of Cytokine Signaling 3	13.58208
HMGA2	High Mobility Group AT-Hook 2	13.56055
DUSP1	Dual Specificity Phosphatase 1	13.55040
LEPR	Leptin Receptor	13.54655
MIR31HG	MIR31 Host Gene	13.54609
APOE	Apolipoprotein E	13.52957
CBR3-AS1	CBR3 Antisense RNA 1	13.49926

RECK	Reversion Inducing Cysteine Rich Protein With Kazal Motifs	13.49046
HAGLR	HOXD Antisense Growth-Associated Long Non-Coding RNA	13.48993
CASC11	Cancer Susceptibility 11	13.46950
LNCRNA-ATB	Long Noncoding RNA Activated By TGF-Beta	13.44183
BID	BH3 Interacting Domain Death Agonist	13.41714
MAP2K4	Mitogen-Activated Protein Kinase Kinase 4	13.40726
TGFB3	Transforming Growth Factor Beta 3	13.40574
CDC42	Cell Division Cycle 42	13.39336
CDH13	Cadherin 13	13.37746
BCYRN1	Brain Cytoplasmic RNA 1	13.35394
IL6R	Interleukin 6 Receptor	13.35391
TEK	TEK Receptor Tyrosine Kinase	13.32007
PXN	Paxillin	13.31032
MIR32	MicroRNA 32	13.30436
HDAC2	Histone Deacetylase 2	13.29922
HK2	Hexokinase 2	13.29364
MSLN	Mesothelin	13.28363
MIF	Macrophage Migration Inhibitory Factor	13.26489
SNHG20	Small Nucleolar RNA Host Gene 20	13.25697
DMBT1	Deleted In Malignant Brain Tumors 1	13.25648
MAGEA4	MAGE Family Member A4	13.25023
WNT9A	Wnt Family Member 9A	13.23985
CLU	Clusterin	13.23461
PRSS1	Serine Protease 1	13.23279
MMP13	Matrix Metallopeptidase 13	13.23075
CSF1R	Colony Stimulating Factor 1 Receptor	13.22247
COL18A1	Collagen Type XVIII Alpha 1 Chain	13.21511
ALOX5	Arachidonate 5-Lipoxygenase	13.21225
RUNX2	RUNX Family Transcription Factor 2	13.20915
BIRC2	Baculoviral IAP Repeat Containing 2	13.20775
CEACAM1	CEA Cell Adhesion Molecule 1	13.18478
POLH	DNA Polymerase Eta	13.18254
SNHG7	Small Nucleolar RNA Host Gene 7	13.18075
INSR	Insulin Receptor	13.17474
FANCE	FA Complementation Group E	13.15056
SSX2	SSX Family Member 2	13.14652
MTR	5-Methyltetrahydrofolate-Homocysteine Methyltransferase	13.13577
PTK2B	Protein Tyrosine Kinase 2 Beta	13.13167
ILK	Integrin Linked Kinase	13.12751
DAXX	Death Domain Associated Protein	13.12576
NOTCH4	Notch Receptor 4	13.11238
PTPN3	Protein Tyrosine Phosphatase Non-Receptor Type 3	13.11204

TFRC	Transferrin Receptor	13.11199
NANOG	Nanog Homeobox	13.10758
STAT6	Signal Transducer And Activator Of Transcription 6	13.09457
SNHG15	Small Nucleolar RNA Host Gene 15	13.07904
CDC25C	Cell Division Cycle 25C	13.06368
CASR	Calcium Sensing Receptor	13.04124
MIR29B2	MicroRNA 29b-2	13.03759
TSG101	Tumor Susceptibility 101	13.03256
SNHG12	Small Nucleolar RNA Host Gene 12	13.03099
CHD1	Chromodomain Helicase DNA Binding Protein 1	13.01693
CCNG1	Cyclin G1	12.99853
BRMS1	BRMS1 Transcriptional Repressor And Anoikis Regulator	12.98731
EDNRA	Endothelin Receptor Type A	12.98592
GNRH1	Gonadotropin Releasing Hormone 1	12.98028
PRKAA1	Protein Kinase AMP-Activated Catalytic Subunit Alpha 1	12.97492
MIR101-1	MicroRNA 101-1	12.96857
GSN	Gelsolin	12.96780
HSD17B1	Hydroxysteroid 17-Beta Dehydrogenase 1	12.96629
TCIM	Transcriptional And Immune Response Regulator	12.94586
ST14	ST14 Transmembrane Serine Protease Matriptase	12.93978
AHR	Aryl Hydrocarbon Receptor	12.93269
TINCR	TINCR Ubiquitin Domain Containing	12.92446
EIF4E	Eukaryotic Translation Initiation Factor 4E	12.91207
FOXA1	Forkhead Box A1	12.90673
ANO1	Anoctamin 1	12.89779
TNFRSF6B	TNF Receptor Superfamily Member 6b	12.89468
SSTR2	Somatostatin Receptor 2	12.89358
WNT8B	Wnt Family Member 8B	12.84006
IL3	Interleukin 3	12.83666
RUFY3	RUN And FYVE Domain Containing 3	12.83460
WNT7A	Wnt Family Member 7A	12.82144
ADH1B	Alcohol Dehydrogenase 1B (Class I), Beta Polypeptide	12.80579
NSD1	Nuclear Receptor Binding SET Domain Protein 1	12.79680
PTTG1	PTTG1 Regulator Of Sister Chromatid Separation, Securin	12.78926
SHBG	Sex Hormone Binding Globulin	12.78651
PPP2R1A	Protein Phosphatase 2 Scaffold Subunit Aalpha	12.77656
XRCC5	X-Ray Repair Cross Complementing 5	12.77518

FADD	Fas Associated Via Death Domain	12.77491
RIOX2	Ribosomal Oxygenase 2	12.75044
IL24	Interleukin 24	12.74613
BDNF	Brain Derived Neurotrophic Factor	12.73199
CD28	CD28 Molecule	12.72972
NCOR1	Nuclear Receptor Corepressor 1	12.72204
SEC23B	SEC23 Homolog B, COPII Coat Complex Component	12.67924
YY1	YY1 Transcription Factor	12.67584
HPGD	15-Hydroxyprostaglandin Dehydrogenase	12.67572
SGK1	Serum/Glucocorticoid Regulated Kinase 1	12.67464
ITGA5	Integrin Subunit Alpha 5	12.66219
PRL	Prolactin	12.65816
PRMT7	Protein Arginine Methyltransferase 7	12.63374
DIABLO	Diablo IAP-Binding Mitochondrial Protein	12.62645
ZKSCAN1	Zinc Finger With KRAB And SCAN Domains 1	12.62438
UBE2C	Ubiquitin Conjugating Enzyme E2 C	12.61744
PIP	Prolactin Induced Protein	12.60678
NAB2	NGFI-A Binding Protein 2	12.60651
POLG	DNA Polymerase Gamma, Catalytic Subunit	12.57718
MC1R	Melanocortin 1 Receptor	12.57465
FLI1	Fli-1 Proto-Oncogene, ETS Transcription Factor	12.57334
FZD8	Frizzled Class Receptor 8	12.56948
KCNQ1	Potassium Voltage-Gated Channel Subfamily Q Member 1	12.54380
PDGFA	Platelet Derived Growth Factor Subunit A	12.54262
LDHA	Lactate Dehydrogenase A	12.52289
ITGB4	Integrin Subunit Beta 4	12.51348
FALEC	Focally Amplified Long Non-Coding RNA In Epithelial Cancer	12.50866
SF3B1	Splicing Factor 3b Subunit 1	12.50505
NOS3	Nitric Oxide Synthase 3	12.50167
APAF1	Apoptotic Peptidase Activating Factor 1	12.50148
AURKB	Aurora Kinase B	12.49881
CD80	CD80 Molecule	12.49181
ID1	Inhibitor Of DNA Binding 1, HLH Protein	12.46653
MIR92A1	MicroRNA 92a-1	12.46208
TTN	Titin	12.45451
ENO1	Enolase 1	12.44369
TXN	Thioredoxin	12.43581

YWHAE	Tyrosine 3-Monooxygenase/Tryptophan 5-Monooxygenase Activation Protein Epsilon	12.42391
PBRM1	Polybromo 1	12.41002
IGFBP1	Insulin Like Growth Factor Binding Protein 1	12.40332
EREG	Epiregulin	12.40119
LIMK1	LIM Domain Kinase 1	12.40049
HLA-DQA1	Major Histocompatibility Complex, Class II, DQ Alpha 1	12.39556
SLC19A1	Solute Carrier Family 19 Member 1	12.39519
MIR455	MicroRNA 455	12.39010
IGFBP5	Insulin Like Growth Factor Binding Protein 5	12.39005
LAMC2	Laminin Subunit Gamma 2	12.38091
CD19	CD19 Molecule	12.38067
BLCAP	BLCAP Apoptosis Inducing Factor	12.34738
MIR133A1	MicroRNA 133a-1	12.33610
ARID4B	AT-Rich Interaction Domain 4B	12.32584
CASC19	Cancer Susceptibility 19	12.31683
PTGER4	Prostaglandin E Receptor 4	12.31607
ABCC3	ATP Binding Cassette Subfamily C Member 3	12.30313
FLNC	Filamin C	12.30276
F3	Coagulation Factor III, Tissue Factor	12.29874
ALCAM	Activated Leukocyte Cell Adhesion Molecule	12.29742
TCF7L1	Transcription Factor 7 Like 1	12.29300
FGF3	Fibroblast Growth Factor 3	12.28692
RHOB	Ras Homolog Family Member B	12.27954
EXO1	Exonuclease 1	12.25046
XRCC6	X-Ray Repair Cross Complementing 6	12.23881
ANPEP	Alanyl Aminopeptidase, Membrane	12.23668
NR3C1	Nuclear Receptor Subfamily 3 Group C Member 1	12.21728
CD9	CD9 Molecule	12.21497
PLG	Plasminogen	12.21240
CXCR5	C-X-C Motif Chemokine Receptor 5	12.20650
MPL	MPL Proto-Oncogene, Thrombopoietin Receptor	12.19751
WIF1	WNT Inhibitory Factor 1	12.18965
CNDP2	Carnosine Dipeptidase 2	12.17985
FGF8	Fibroblast Growth Factor 8	12.16280
HOXA-AS2	HOXA Cluster Antisense RNA 2	12.15296
LOC11080626:	TERT 5' Regulatory Region	12.15023
CCN1	Cellular Communication Network Factor 1	12.14983
GPX1	Glutathione Peroxidase 1	12.13782

TMEFF2	Transmembrane Protein With EGF Like And Two Follistatin Like Domains 2	12.12145
RPS20	Ribosomal Protein S20	12.11469
TUSC7	Tumor Suppressor Candidate 7	12.10793
ITGA3	Integrin Subunit Alpha 3	12.10748
HMGA1	High Mobility Group AT-Hook 1	12.08366
ASXL1	ASXL Transcriptional Regulator 1	12.08297
SERPINB2	Serpin Family B Member 2	12.07522
NCOR2	Nuclear Receptor Corepressor 2	12.07466
HIC1	HIC ZBTB Transcriptional Repressor 1	12.07197
NOS1	Nitric Oxide Synthase 1	12.06814
RTEL1	Regulator Of Telomere Elongation Helicase 1	12.06644
FZD5	Frizzled Class Receptor 5	12.05768
PCAT18	Prostate Cancer Associated Transcript 18	12.05208
GNB1	G Protein Subunit Beta 1	12.04668
CEBPA	CCAAT Enhancer Binding Protein Alpha	12.04474
CT45A3	Cancer/Testis Antigen Family 45 Member A3	12.03176
POMC	Proopiomelanocortin	11.99675
PIK3CD	Phosphatidylinositol-4,5-Bisphosphate 3-Kinase Catalytic Subunit Delta	11.99603
TGIF1	TGFB Induced Factor Homeobox 1	11.95467
POSTN	Periostin	11.94747
TKT	Transketolase	11.92480
PRLR	Prolactin Receptor	11.89818
GADD45G	Growth Arrest And DNA Damage Inducible Gamma	11.89405
POLR1C	RNA Polymerase I And III Subunit C	11.87921
GH1	Growth Hormone 1	11.87713
DKC1	Dyskerin Pseudouridine Synthase 1	11.87573
FZD2	Frizzled Class Receptor 2	11.87041
KISS1	KiSS-1 Metastasis Suppressor	11.86592
ARID1B	AT-Rich Interaction Domain 1B	11.85755
CHKA	Choline Kinase Alpha	11.85457
EPHA3	EPH Receptor A3	11.85234
LOC11158921[illegible]	BRCA1 Promoter Region	11.84953
EDN1	Endothelin 1	11.84714
GSTM3	Glutathione S-Transferase Mu 3	11.81535
MST1R	Macrophage Stimulating 1 Receptor	11.81046
ZEB1-AS1	ZEB1 Antisense RNA 1	11.80333
ACE	Angiotensin I Converting Enzyme	11.80040
BAG1	BAG Cochaperone 1	11.79598
SCAI	Suppressor Of Cancer Cell Invasion	11.78194
IL13	Interleukin 13	11.76843
BTK	Bruton Tyrosine Kinase	11.74875
TNFRSF8	TNF Receptor Superfamily Member 8	11.74783

HDAC4	Histone Deacetylase 4	11.74549
SNHG5	Small Nucleolar RNA Host Gene 5	11.73146
CCN2	Cellular Communication Network Factor 2	11.72295
TCF7	Transcription Factor 7	11.71416
CADM1	Cell Adhesion Molecule 1	11.70069
SPHK1	Sphingosine Kinase 1	11.70027
LCN2	Lipocalin 2	11.69972
EPAS1	Endothelial PAS Domain Protein 1	11.68780
AATBC	Apoptosis Associated Transcript In Bladder Cancer	11.68060
FGF9	Fibroblast Growth Factor 9	11.67627
EGOT	Eosinophil Granule Ontogeny Transcript	11.67409
UCHL1	Ubiquitin C-Terminal Hydrolase L1	11.65195
AMACR	Alpha-Methylacyl-CoA Racemase	11.64160
IFNB1	Interferon Beta 1	11.63999
DAB2IP	DAB2 Interacting Protein	11.63503
NAA15	N-Alpha-Acetyltransferase 15, NatA Auxiliary Subunit	11.63379
POLR1H	RNA Polymerase I Subunit H	11.63221
ALDOA	Aldolase, Fructose-Bisphosphate A	11.63158
CASC16	Cancer Susceptibility 16	11.62459
IL11	Interleukin 11	11.61751
MIR92A2	MicroRNA 92a-2	11.59865
APC2	APC Regulator Of WNT Signaling Pathway 2	11.59171
NOD2	Nucleotide Binding Oligomerization Domain Containing 2	11.58657
HAVCR2	Hepatitis A Virus Cellular Receptor 2	11.58506
PDPK1	3-Phosphoinositide Dependent Protein Kinase 1	11.57596
CAT	Catalase	11.56603
HDC	Histidine Decarboxylase	11.55787
MYH9	Myosin Heavy Chain 9	11.55106
BCR	BCR Activator Of RhoGEF And GTPase	11.51858
KRT14	Keratin 14	11.51311
FER1L4	Fer-1 Like Family Member 4 (Pseudogene)	11.50576
CCN4	Cellular Communication Network Factor 4	11.47882
GPR68	G Protein-Coupled Receptor 68	11.47826
PECAM1	Platelet And Endothelial Cell Adhesion Molecule 1	11.46988
FGF19	Fibroblast Growth Factor 19	11.45124
MIR330	MicroRNA 330	11.44337
HIF1A-AS2	HIF1A Antisense RNA 2	11.44304
SOCS1	Suppressor Of Cytokine Signaling 1	11.43118
SSX1	SSX Family Member 1	11.41245

CEACAM3	CEA Cell Adhesion Molecule 3	11.38849
IL18	Interleukin 18	11.37633
GATA4	GATA Binding Protein 4	11.37351
LMNA	Lamin A/C	11.37109
XBP1	X-Box Binding Protein 1	11.36740
TOX3	TOX High Mobility Group Box Family Member 3	11.35327
NUMA1	Nuclear Mitotic Apparatus Protein 1	11.34642
DPP4	Dipeptidyl Peptidase 4	11.34518
KIF1B	Kinesin Family Member 1B	11.33015
LRP6	LDL Receptor Related Protein 6	11.32901
CFTR	CF Transmembrane Conductance Regulator	11.31914
PEBP1	Phosphatidylethanolamine Binding Protein 1	11.30170
SYK	Spleen Associated Tyrosine Kinase	11.27345
CIP2A	Cellular Inhibitor Of PP2A	11.26729
KDM1A	Lysine Demethylase 1A	11.25764
CR2	Complement C3d Receptor 2	11.25510
MIR28	MicroRNA 28	11.24233
FEZF1	FEZ Family Zinc Finger 1	11.24148
THPO	Thrombopoietin	11.22470
SCAT1	S-Phase Cancer Associated Transcript 1	11.21900
ITGA2	Integrin Subunit Alpha 2	11.21720
PTCH2	Patched 2	11.20086
GATA6	GATA Binding Protein 6	11.19924
WT1-AS	WT1 Antisense RNA	11.19883
NES	Nestin	11.17883
DPH1	Diphthamide Biosynthesis 1	11.17417
NDUFA13	NADH:Ubiquinone Oxidoreductase Subunit A13	11.16763
MLN	Motilin	11.16519
TUBB	Tubulin Beta Class I	11.14037
RYR1	Ryanodine Receptor 1	11.13265
IKBKG	Inhibitor Of Nuclear Factor Kappa B Kinase Regulatory Subunit Gamma	11.12402
MTAP	Methylthioadenosine Phosphorylase	11.11802
KDM5B	Lysine Demethylase 5B	11.10880
TLR5	Toll Like Receptor 5	11.09472
GATA1	GATA Binding Protein 1	11.08285
S100A2	S100 Calcium Binding Protein A2	11.07967
SOD1	Superoxide Dismutase 1	11.07758
CASP2	Caspase 2	11.06283
MIR574	MicroRNA 574	11.06028
PIWIL1	Piwi Like RNA-Mediated Gene Silencing 1	11.05651
ANGPT1	Angiopoietin 1	11.05580
CUL1	Cullin 1	11.05235
PRKD1	Protein Kinase D1	11.04688

NFIB	Nuclear Factor I B	11.03986
CYP24A1	Cytochrome P450 Family 24 Subfamily A Member 1	11.02950
MIR497	MicroRNA 497	11.02564
PGC	Progastricsin	11.01866
DLL4	Delta Like Canonical Notch Ligand 4	11.01484
ADH1C	Alcohol Dehydrogenase 1C (Class I), Gamma Polypeptide	11.00658
ROCK1	Rho Associated Coiled-Coil Containing Protein Kinase 1	10.99751
SFRP1	Secreted Frizzled Related Protein 1	10.98976
FEZF1-AS1	FEZF1 Antisense RNA 1	10.98629
CHRNA5	Cholinergic Receptor Nicotinic Alpha 5 Subunit	10.98599
RACK1	Receptor For Activated C Kinase 1	10.97862
RRM2	Ribonucleotide Reductase Regulatory Subunit M2	10.97452
SASH1	SAM And SH3 Domain Containing 1	10.96522
DMTF1	Cyclin D Binding Myb Like Transcription Factor 1	10.95663
CEBPB	CCAAT Enhancer Binding Protein Beta	10.94597
DUXAP9	Double Homeobox A Pseudogene 9	10.92950
HSPD1	Heat Shock Protein Family D (Hsp60) Member 1	10.92260
GSDMA	Gasdermin A	10.92159
S100A9	S100 Calcium Binding Protein A9	10.90382
CD79A	CD79a Molecule	10.89865
CENPF	Centromere Protein F	10.89027
SOS2	SOS Ras/Rho Guanine Nucleotide Exchange Factor 2	10.84168
SRA1	Steroid Receptor RNA Activator 1	10.84058
MIR135A1	MicroRNA 135a-1	10.83855
HNRNPK	Heterogeneous Nuclear Ribonucleoprotein K	10.83293
ESM1	Endothelial Cell Specific Molecule 1	10.82961
CTSL	Cathepsin L	10.82784
PYY	Peptide YY	10.81341
CD40LG	CD40 Ligand	10.81197
PTPA	Protein Phosphatase 2 Phosphatase Activator	10.80775
MIR151A	MicroRNA 151a	10.80719
ABHD11-AS1	ABHD11 Antisense RNA 1 (Tail To Tail)	10.80450
BIRC7	Baculoviral IAP Repeat Containing 7	10.80235
NGFR	Nerve Growth Factor Receptor	10.80109
TFAP2A	Transcription Factor AP-2 Alpha	10.79823
TMEM238L	Transmembrane Protein 238 Like	10.79461
MIR4435-2HG	MIR4435-2 Host Gene	10.76535
AGAP2-AS1	AGAP2 Antisense RNA 1	10.76204

HLA-C	Major Histocompatibility Complex, Class I, C	10.75610
TK1	Thymidine Kinase 1	10.74914
NPTN-IT1	NPTN Intronic Transcript 1	10.74101
TUSC3	Tumor Suppressor Candidate 3	10.72959
DDIT3	DNA Damage Inducible Transcript 3	10.70824
CACYBP	Calcyclin Binding Protein	10.70647
HERPUD1	Homocysteine Inducible ER Protein With Ubiquitin Like Domain 1	10.69766
CCDC136	Coiled-Coil Domain Containing 136	10.69493
SELENBP1	Selenium Binding Protein 1	10.68999
MAPK10	Mitogen-Activated Protein Kinase 10	10.68951
PAK4	P21 (RAC1) Activated Kinase 4	10.68149
P2RX7	Purinergic Receptor P2X 7	10.67900
CYP3A5	Cytochrome P450 Family 3 Subfamily A Member 5	10.67246
UHRF1	Ubiquitin Like With PHD And Ring Finger Domains 1	10.66948
SNHG6	Small Nucleolar RNA Host Gene 6	10.66690
GHR	Growth Hormone Receptor	10.65663
TTK	TTK Protein Kinase	10.65545
RRP7A	Ribosomal RNA Processing 7 Homolog A	10.65228
PLCE1	Phospholipase C Epsilon 1	10.63903
FBLN1	Fibulin 1	10.63584
MIR370	MicroRNA 370	10.62244
LINC00511	Long Intergenic Non-Protein Coding RNA 511	10.61794
MCM2	Minichromosome Maintenance Complex Component 2	10.59006
CLDN23	Claudin 23	10.58858
H2AX	H2A.X Variant Histone	10.58722
GLI3	GLI Family Zinc Finger 3	10.57563
PER1	Period Circadian Regulator 1	10.57312
FAP	Fibroblast Activation Protein Alpha	10.56447
CALB2	Calbindin 2	10.54976
CEACAM7	CEA Cell Adhesion Molecule 7	10.54535
HOXA13	Homeobox A13	10.54178
EGFLAM-AS1	EGFLAM Antisense RNA 1	10.53585
CD36	CD36 Molecule	10.52660
MIR377	MicroRNA 377	10.52061
HSP90AB1	Heat Shock Protein 90 Alpha Family Class B Member 1	10.52021
PTGES	Prostaglandin E Synthase	10.51850
MIR26B	MicroRNA 26b	10.51715
GRPR	Gastrin Releasing Peptide Receptor	10.51204
CDK7	Cyclin Dependent Kinase 7	10.50755
KLRK1	Killer Cell Lectin Like Receptor K1	10.50718
HSF1	Heat Shock Transcription Factor 1	10.50579

EPOR	Erythropoietin Receptor	10.50464
TNFRSF10D	TNF Receptor Superfamily Member 10d	10.49142
NR1H2	Nuclear Receptor Subfamily 1 Group H Member 2	10.49117
NORAD	Non-Coding RNA Activated By DNA Damage	10.48536
VWF	Von Willebrand Factor	10.47885
ADAR	Adenosine Deaminase RNA Specific	10.47188
CCR5	C-C Motif Chemokine Receptor 5	10.46879
TRAF2	TNF Receptor Associated Factor 2	10.45998
NTRK2	Neurotrophic Receptor Tyrosine Kinase 2	10.45859
SLC5A8	Solute Carrier Family 5 Member 8	10.45236
LINC01772	Long Intergenic Non-Protein Coding RNA 1772	10.44920
BMPR2	Bone Morphogenetic Protein Receptor Type 2	10.43978
FGF18	Fibroblast Growth Factor 18	10.43766
F2R	Coagulation Factor II Thrombin Receptor	10.43688
SULT1A1	Sulfotransferase Family 1A Member 1	10.43682
DVL1	Dishevelled Segment Polarity Protein 1	10.43522
DDX5	DEAD-Box Helicase 5	10.43063
BRDT	Bromodomain Testis Associated	10.43032
SMAD6	SMAD Family Member 6	10.42917
TDGF1	Teratocarcinoma-Derived Growth Factor 1	10.41612
IGF2BP3	Insulin Like Growth Factor 2 MRNA Binding Protein 3	10.41087
IRS2	Insulin Receptor Substrate 2	10.40562
TACC3	Transforming Acidic Coiled-Coil Containing Protein 3	10.40436
MIR361	MicroRNA 361	10.39663
MMP12	Matrix Metallopeptidase 12	10.39484
MSTO2P	Misato Family Member 2, Pseudogene	10.39396
MYLK	Myosin Light Chain Kinase	10.36927
TIMELESS	Timeless Circadian Regulator	10.36811
ACTN4	Actinin Alpha 4	10.36260
MIR376A1	MicroRNA 376a-1	10.35987
GHRH	Growth Hormone Releasing Hormone	10.35036
BUB3	BUB3 Mitotic Checkpoint Protein	10.34458
HSPA1A	Heat Shock Protein Family A (Hsp70) Member 1A	10.34194
GAB1	GRB2 Associated Binding Protein 1	10.34005
DLL1	Delta Like Canonical Notch Ligand 1	10.33331
LIN28B	Lin-28 Homolog B	10.30930
TCF3	Transcription Factor 3	10.30452
GDNF	Glial Cell Derived Neurotrophic Factor	10.29793

KRT17	Keratin 17	10.29278
CYP27B1	Cytochrome P450 Family 27 Subfamily B Member 1	10.27895
BNIP3	BCL2 Interacting Protein 3	10.27502
GADD45B	Growth Arrest And DNA Damage Inducible Beta	10.26820
MAD2L1	Mitotic Arrest Deficient 2 Like 1	10.25418
LYVE1	Lymphatic Vessel Endothelial Hyaluronan Receptor 1	10.24236
SPDEF	SAM Pointed Domain Containing ETS Transcription Factor	10.24141
MELK	Maternal Embryonic Leucine Zipper Kinase	10.23315
THBS4	Thrombospondin 4	10.23006
LGR5	Leucine Rich Repeat Containing G Protein-Coupled Receptor 5	10.22649
HSP90B1	Heat Shock Protein 90 Beta Family Member 1	10.22398
STK4	Serine/Threonine Kinase 4	10.21481
ELF3	E74 Like ETS Transcription Factor 3	10.17727
DLEU1	Deleted In Lymphocytic Leukemia 1	10.17663
MIR132	MicroRNA 132	10.17306
MST1	Macrophage Stimulating 1	10.16840
BGLAP	Bone Gamma-Carboxyglutamate Protein	10.16702
NGF	Nerve Growth Factor	10.16390
RRAS	RAS Related	10.16227
ROBO1	Roundabout Guidance Receptor 1	10.15357
SLC29A1	Solute Carrier Family 29 Member 1 (Augustine Blood Group)	10.14680
LOC10272356(	Uncharacterized LOC102723566	10.13413
GLI2	GLI Family Zinc Finger 2	10.11732
ACP3	Acid Phosphatase 3	10.11604
SMARCA2	SWI/SNF Related, Matrix Associated, Actin Dependent Regulator Of Chromatin, Subfamily A, Member 2	10.11073
HPN	Hepsin	10.10856
PTPRT	Protein Tyrosine Phosphatase Receptor Type T	10.10587
CEP57	Centrosomal Protein 57	10.10414
SRSF2	Serine And Arginine Rich Splicing Factor 2	10.10152
KLK6	Kallikrein Related Peptidase 6	10.09520
TTR	Transthyretin	10.09408
HSPA8	Heat Shock Protein Family A (Hsp70) Member 8	10.08168
MYCL	MYCL Proto-Oncogene, BHLH Transcription Factor	10.08116
MIR590	MicroRNA 590	10.07819

LTO1	LTO1 Maturation Factor Of ABCE1	10.07208
CYLD	CYLD Lysine 63 Deubiquitinase	10.05408
PTP4A3	Protein Tyrosine Phosphatase 4A3	10.04913
FLNA	Filamin A	10.03893
TDRG1	Testis Development Related 1	10.02949
H3-3A	H3.3 Histone A	10.02571
SIRT3	Sirtuin 3	10.01833
ATP4B	ATPase H+/K+ Transporting Subunit Beta	10.01299
CLDN1	Claudin 1	10.00876
RELB	RELB Proto-Oncogene, NF-KB Subunit	10.00585
SULF1	Sulfatase 1	10.00433
CXCR3	C-X-C Motif Chemokine Receptor 3	10.00090
MAGED2	MAGE Family Member D2	9.99386
DDR1	Discoidin Domain Receptor Tyrosine Kinase 1	9.99188
EPO	Erythropoietin	9.99183
LINC01234	Long Intergenic Non-Protein Coding RNA 1234	9.98601
CD8A	CD8a Molecule	9.97949
RXRB	Retinoid X Receptor Beta	9.96673
UBE2T	Ubiquitin Conjugating Enzyme E2 T	9.96025
ELN	Elastin	9.95617
ACACA	Acetyl-CoA Carboxylase Alpha	9.95178
IL15	Interleukin 15	9.95020
WFDC2	WAP Four-Disulfide Core Domain 2	9.93384
COL7A1	Collagen Type VII Alpha 1 Chain	9.92919
TRAF6	TNF Receptor Associated Factor 6	9.92100
DIRC3	Disrupted In Renal Carcinoma 3	9.91569
CLPTM1L	CLPTM1 Like	9.89746
KAT5	Lysine Acetyltransferase 5	9.89409
SEPTIN9	Septin 9	9.88183
PRKCI	Protein Kinase C Iota	9.88031
SAT1	Spermidine/Spermine N1-Acetyltransferase 1	9.87305
PPARD	Peroxisome Proliferator Activated Receptor Delta	9.87294
ALPP	Alkaline Phosphatase, Placental	9.87205
ACTA2	Actin Alpha 2, Smooth Muscle	9.86160
PTP4A1	Protein Tyrosine Phosphatase 4A1	9.85850
LZTR1	Leucine Zipper Like Transcription Regulator 1	9.84566
CCAR2	Cell Cycle And Apoptosis Regulator 2	9.82716
TGM2	Transglutaminase 2	9.82507
PLAT	Plasminogen Activator, Tissue Type	9.82304
TRIM24	Tripartite Motif Containing 24	9.80824
PIAS1	Protein Inhibitor Of Activated STAT 1	9.80215
H3C2	H3 Clustered Histone 2	9.78566
DDX3X	DEAD-Box Helicase 3 X-Linked	9.77776

MIR542	MicroRNA 542	9.76519
SDC1	Syndecan 1	9.75441
PRDM16-DT	PRDM16 Divergent Transcript	9.75203
RAD52	RAD52 Homolog, DNA Repair Protein	9.74356
GHSR	Growth Hormone Secretagogue Receptor	9.74208
VTN	Vitronectin	9.74199
DOT1L	DOT1 Like Histone Lysine Methyltransferase	9.73710
PTK6	Protein Tyrosine Kinase 6	9.73424
SFN	Stratifin	9.72625
FUT3	Fucosyltransferase 3 (Lewis Blood Group)	9.70259
GRN	Granulin Precursor	9.69881
MAP2K5	Mitogen-Activated Protein Kinase Kinase 5	9.69676
LIFR	LIF Receptor Subunit Alpha	9.69268
POLB	DNA Polymerase Beta	9.68325
ADAM12	ADAM Metallopeptidase Domain 12	9.65697
AKR1C3	Aldo-Keto Reductase Family 1 Member C3	9.64377
LINC00628	Long Intergenic Non-Protein Coding RNA 628	9.63204
FOSL1	FOS Like 1, AP-1 Transcription Factor Subunit	9.63122
LINC00052	Long Intergenic Non-Protein Coding RNA 52	9.62873
DCK	Deoxycytidine Kinase	9.62306
JAG2	Jagged Canonical Notch Ligand 2	9.61592
ADA	Adenosine Deaminase	9.61237
LTA	Lymphotoxin Alpha	9.61051
RBM6	RNA Binding Motif Protein 6	9.60968
PRKCB	Protein Kinase C Beta	9.60684
SEMA3B	Semaphorin 3B	9.60247
RPS19	Ribosomal Protein S19	9.59371
PRKAA2	Protein Kinase AMP-Activated Catalytic Subunit Alpha 2	9.59332
AGER	Advanced Glycosylation End-Product Specific Receptor	9.58097
TNS4	Tensin 4	9.56596
MIR130B	MicroRNA 130b	9.55656
NCOA1	Nuclear Receptor Coactivator 1	9.55538
S100P	S100 Calcium Binding Protein P	9.54807
RCVRN	Recoverin	9.54573
OR3A4P	Olfactory Receptor Family 3 Subfamily A Member 4 Pseudogene	9.52396
MYOD1	Myogenic Differentiation 1	9.52114
MIR326	MicroRNA 326	9.51530
ADAM17	ADAM Metallopeptidase Domain 17	9.51490

PAX3	Paired Box 3	9.50671
SIX1	SIX Homeobox 1	9.50503
PIM1	Pim-1 Proto-Oncogene, Serine/Threonine Kinase	9.50269
BCAS1	Brain Enriched Myelin Associated Protein 1	9.49966
RBBP4	RB Binding Protein 4, Chromatin Remodeling Factor	9.49374
ZFP36L1	ZFP36 Ring Finger Protein Like 1	9.49184
IRF4	Interferon Regulatory Factor 4	9.47707
INHA	Inhibin Subunit Alpha	9.47676
PIN1	Peptidylprolyl Cis/Trans Isomerase, NIMA-Interacting 1	9.47658
AFAP1	Actin Filament Associated Protein 1	9.47213
MIR212	MicroRNA 212	9.47055
SREBF1	Sterol Regulatory Element Binding Transcription Factor 1	9.46350
RPL5	Ribosomal Protein L5	9.45916
TNFAIP3	TNF Alpha Induced Protein 3	9.45531
PRC1	Protein Regulator Of Cytokinesis 1	9.45456
MIR218-1	MicroRNA 218-1	9.45250
MDC1-AS1	MDC1 Antisense RNA 1	9.44789
GBA	Glucosylceramidase Beta	9.44476
MIR184	MicroRNA 184	9.44196
EEF1A1	Eukaryotic Translation Elongation Factor 1 Alpha 1	9.44173
RPL34-DT	RPL34 Divergent Transcript	9.43042
RPS27	Ribosomal Protein S27	9.42376
MIR489	MicroRNA 489	9.42073
APOB	Apolipoprotein B	9.41230
RRM1	Ribonucleotide Reductase Catalytic Subunit M1	9.40632
PDGFD	Platelet Derived Growth Factor D	9.40202
CASP1	Caspase 1	9.38396
ZMIZ1	Zinc Finger MIZ-Type Containing 1	9.38359
COL4A2	Collagen Type IV Alpha 2 Chain	9.38227
NCOA2	Nuclear Receptor Coactivator 2	9.38135
MIR345	MicroRNA 345	9.38017
PRKCE	Protein Kinase C Epsilon	9.37792
PGF	Placental Growth Factor	9.36998
CASC3	CASC3 Exon Junction Complex Subunit	9.34878
BMP1	Bone Morphogenetic Protein 1	9.34522
EMSY	EMSY Transcriptional Repressor, BRCA2 Interacting	9.34501
SLC35C2	Solute Carrier Family 35 Member C2	9.34017
ABCA3	ATP Binding Cassette Subfamily A Member 3	9.33910

ZBTB16	Zinc Finger And BTB Domain Containing 16	9.33710
GZMB	Granzyme B	9.33547
TNC	Tenascin C	9.32624
LIF	LIF Interleukin 6 Family Cytokine	9.32541
MIR135B	MicroRNA 135b	9.31722
CCL20	C-C Motif Chemokine Ligand 20	9.30908
PICART1	P53 Inducible Cancer Associated RNA Transcript 1	9.29990
FCGR2A	Fc Fragment Of IgG Receptor IIa	9.29611
COL11A1	Collagen Type XI Alpha 1 Chain	9.29564
IFITM1	Interferon Induced Transmembrane Protein 1	9.29366
MECOM	MDS1 And EVI1 Complex Locus	9.28689
ETS2	ETS Proto-Oncogene 2, Transcription Factor	9.27839
TFE3	Transcription Factor Binding To IGHM Enhancer 3	9.26907
ALOX12	Arachidonate 12-Lipoxygenase, 12S Type	9.26883
CHI3L1	Chitinase 3 Like 1	9.26682
MIR675	MicroRNA 675	9.26470
LLGL1	LLGL Scribble Cell Polarity Complex Component 1	9.25775
LINC00901	Long Intergenic Non-Protein Coding RNA 901	9.25744
ECRG4	ECRG4 Augurin Precursor	9.25671
ERN1	Endoplasmic Reticulum To Nucleus Signaling 1	9.25609
ATF3	Activating Transcription Factor 3	9.25371
RPS6KA3	Ribosomal Protein S6 Kinase A3	9.25338
AKAP13	A-Kinase Anchoring Protein 13	9.25175
BIN1	Bridging Integrator 1	9.25010
TRAF3	TNF Receptor Associated Factor 3	9.23794
BMP7	Bone Morphogenetic Protein 7	9.22674
STUB1	STIP1 Homology And U-Box Containing Protein 1	9.21720
TINF2	TERF1 Interacting Nuclear Factor 2	9.21685
MIR124-3	MicroRNA 124-3	9.20983
KMT2C	Lysine Methyltransferase 2C	9.20807
SELE	Selectin E	9.20262
SLPI	Secretory Leukocyte Peptidase Inhibitor	9.20061
ELANE	Elastase, Neutrophil Expressed	9.19417
MED12	Mediator Complex Subunit 12	9.18143
MIR103A1	MicroRNA 103a-1	9.17984
RHEB	Ras Homolog, MTORC1 Binding	9.17832
WNT4	Wnt Family Member 4	9.17709
TP73-AS1	TP73 Antisense RNA 1	9.17693

DNAJB4	DnaJ Heat Shock Protein Family (Hsp40) Member B4	9.17215
MIR186	MicroRNA 186	9.16890
SUMO1P3	SUMO1 Pseudogene 3	9.16747
MFN2	Mitofusin 2	9.16193
SPINT2	Serine Peptidase Inhibitor, Kunitz Type 2	9.15840
PLA2G4A	Phospholipase A2 Group IVA	9.15720
BBC3	BCL2 Binding Component 3	9.15553
GATA6-AS1	GATA6 Antisense RNA 1 (Head To Head)	9.15224
CBLL1	Cbl Proto-Oncogene Like 1	9.14944
RHOC	Ras Homolog Family Member C	9.14160
PTPN1	Protein Tyrosine Phosphatase Non-Receptor Type 1	9.13190
PRSS21	Serine Protease 21	9.12945
CXCL5	C-X-C Motif Chemokine Ligand 5	9.12827
EHMT1	Euchromatic Histone Lysine Methyltransferase 1	9.12667
CDC6	Cell Division Cycle 6	9.12196
MAPK9	Mitogen-Activated Protein Kinase 9	9.11906
PSEN2	Presenilin 2	9.11758
GUCY2C	Guanylate Cyclase 2C	9.11423
TCHP	Trichoplein Keratin Filament Binding	9.11274
FZD4	Frizzled Class Receptor 4	9.10760
SH2B3	SH2B Adaptor Protein 3	9.09813
MIR3936HG	MIR3936 Host Gene	9.09311
TLR1	Toll Like Receptor 1	9.09117
NAMPT	Nicotinamide Phosphoribosyltransferase	9.08704
RPL11	Ribosomal Protein L11	9.08290
HP	Haptoglobin	9.08062
ACTC1	Actin Alpha Cardiac Muscle 1	9.07759
PSAP	Prosaposin	9.07137
IL5	Interleukin 5	9.06670
MAPK7	Mitogen-Activated Protein Kinase 7	9.05405
TNFRSF10C	TNF Receptor Superfamily Member 10c	9.04331
SNHG14	Small Nucleolar RNA Host Gene 14	9.02479
CDC20	Cell Division Cycle 20	9.02478
ERVH48-1	Endogenous Retrovirus Group 48 Member 1	9.01709
RARS1	Arginyl-TRNA Synthetase 1	9.01692
CD46	CD46 Molecule	9.00211
SPINT1	Serine Peptidase Inhibitor, Kunitz Type 1	8.99546
SLC25A25-AS	SLC25A25 Antisense RNA 1	8.98793
HDAC6	Histone Deacetylase 6	8.98470
TAP1	Transporter 1, ATP Binding Cassette Subfamily B Member	8.97359

UGT1A7	UDP Glucuronosyltransferase Family 1 Member A7	8.97299
PSG2	Pregnancy Specific Beta-1-Glycoprotein 2	8.97225
NUDT1	Nudix Hydrolase 1	8.96193
WEE1	WEE1 G2 Checkpoint Kinase	8.96061
KCMF1	Potassium Channel Modulatory Factor 1	8.95896
CT69	Cancer/Testis Associated Transcript 69	8.95775
LGALS3BP	Galectin 3 Binding Protein	8.95482
PTPN13	Protein Tyrosine Phosphatase Non-Receptor Type 13	8.95191
BRD4	Bromodomain Containing 4	8.95062
CD86	CD86 Molecule	8.94430
XPO1	Exportin 1	8.94391
PAX6	Paired Box 6	8.94050
SNHG8	Small Nucleolar RNA Host Gene 8	8.93276
WWTR1	WW Domain Containing Transcription Regulator 1	8.92714
LAMB3	Laminin Subunit Beta 3	8.92666
LHCGR	Luteinizing Hormone/Choriogonadotropin Receptor	8.92604
MIR339	MicroRNA 339	8.92491
GPT	Glutamic--Pyruvic Transaminase	8.92122
HDGF	Heparin Binding Growth Factor	8.90512
CSK	C-Terminal Src Kinase	8.89643
CACNA1G	Calcium Voltage-Gated Channel Subunit Alpha1 G	8.89532
MACROD1	Mono-ADP Ribosylhydrolase 1	8.89506
ENSG00000278769		8.89143
EML4	EMAP Like 4	8.88906
CDC25B	Cell Division Cycle 25B	8.88353
ATF1	Activating Transcription Factor 1	8.87774
VCAM1	Vascular Cell Adhesion Molecule 1	8.87462
HMGCR	3-Hydroxy-3-Methylglutaryl-CoA Reductase	8.87456
DIS3L2	DIS3 Like 3'-5' Exoribonuclease 2	8.86758
H19-ICR	H19/IGF2 Imprinting Control Region	8.84693
H4-16	H4 Histone 16	8.84202
MIR202	MicroRNA 202	8.84186
GGT1	Gamma-Glutamyltransferase 1	8.83564
RPSA	Ribosomal Protein SA	8.83541
CD81	CD81 Molecule	8.83532
NCOA6	Nuclear Receptor Coactivator 6	8.83444
HIPK2	Homeodomain Interacting Protein Kinase 2	8.83395
VTRNA2-1	Vault RNA 2-1	8.82126
CD22	CD22 Molecule	8.80982
MMP10	Matrix Metallopeptidase 10	8.80781

VAV3	Vav Guanine Nucleotide Exchange Factor 3	8.80675
TPM3	Tropomyosin 3	8.80324
NOVA1	NOVA Alternative Splicing Regulator 1	8.79032
INTS7	Integrator Complex Subunit 7	8.78893
CERNA2	Competing Endogenous LncRNA 2 For MicroRNA Let-7b	8.77915
TMC8	Transmembrane Channel Like 8	8.77265
MIR485	MicroRNA 485	8.76830
MIR378A	MicroRNA 378a	8.76774
DCLK1	Doublecortin Like Kinase 1	8.76154
RHOBTB2	Rho Related BTB Domain Containing 2	8.75591
PRKCQ	Protein Kinase C Theta	8.74481
ENAH	ENAH Actin Regulator	8.73605
COX5A	Cytochrome C Oxidase Subunit 5A	8.72499
CCKAR	Cholecystokinin A Receptor	8.72021
FUT2	Fucosyltransferase 2	8.71798
AGR2	Anterior Gradient 2, Protein Disulphide Isomerase Family Member	8.71597
TNFRSF13C	TNF Receptor Superfamily Member 13C	8.71585
PPARA	Peroxisome Proliferator Activated Receptor Alpha	8.71558
PHLPP1	PH Domain And Leucine Rich Repeat Protein Phosphatase 1	8.71332
CEBPA-DT	CEBPA Divergent Transcript	8.71321
CSNK1D	Casein Kinase 1 Delta	8.71028
GJB2	Gap Junction Protein Beta 2	8.70082
MUC5B	Mucin 5B, Oligomeric Mucus/Gel-Forming	8.69871
NCRUPAR	Non-Protein Coding RNA, Upstream Of F2R/PAR1	8.69630
HYAL1	Hyaluronidase 1	8.69353
SLC37A3	Solute Carrier Family 37 Member 3	8.69273
IGFBP7	Insulin Like Growth Factor Binding Protein 7	8.68453
AZGP1	Alpha-2-Glycoprotein 1, Zinc-Binding	8.68222
HAGLROS	HAGLR Opposite Strand LncRNA	8.67969
PRMT1	Protein Arginine Methyltransferase 1	8.67325
LINC00941	Long Intergenic Non-Protein Coding RNA 941	8.67247
CCAR1	Cell Division Cycle And Apoptosis Regulator 1	8.66742
PELP1	Proline, Glutamate And Leucine Rich Protein 1	8.66508
MIR625	MicroRNA 625	8.65153
CD164	CD164 Molecule	8.65105
NKILA	NF-KappaB Interacting LncRNA	8.65085

NR3C2	Nuclear Receptor Subfamily 3 Group C Member 2	8.65007
PTH	Parathyroid Hormone	8.64915
DDX53	DEAD-Box Helicase 53	8.64699
SULF2	Sulfatase 2	8.64420
ELK1	ETS Transcription Factor ELK1	8.64339
CXCR1	C-X-C Motif Chemokine Receptor 1	8.63744
NNT-AS1	NNT Antisense RNA 1	8.63576
TP53BP1	Tumor Protein P53 Binding Protein 1	8.63146
PPP1R1B	Protein Phosphatase 1 Regulatory Inhibitor Subunit 1B	8.63146
TP53BP2	Tumor Protein P53 Binding Protein 2	8.63073
AKIP1	A-Kinase Interacting Protein 1	8.62000
CD55	CD55 Molecule (Cromer Blood Group)	8.61790
NET1	Neuroepithelial Cell Transforming 1	8.61427
CA2	Carbonic Anhydrase 2	8.61416
MIR30C1	MicroRNA 30c-1	8.61227
NUP214	Nucleoporin 214	8.61084
DES	Desmin	8.60906
MIR328	MicroRNA 328	8.60815
IBSP	Integrin Binding Sialoprotein	8.58868
RALGDS	Ral Guanine Nucleotide Dissociation Stimulator	8.58079
CLIC1	Chloride Intracellular Channel 1	8.57136
EPHA7	EPH Receptor A7	8.56848
SH3GL1	SH3 Domain Containing GRB2 Like 1, Endophilin A2	8.56502
MECP2	Methyl-CpG Binding Protein 2	8.56239
TSPAN31	Tetraspanin 31	8.55000
IGFBP4	Insulin Like Growth Factor Binding Protein 4	8.54931
HES1	Hes Family BHLH Transcription Factor 1	8.54891
RAB25	RAB25, Member RAS Oncogene Family	8.54603
TUSC2	Tumor Suppressor 2, Mitochondrial Calcium Regulator	8.53881
KLK15	Kallikrein Related Peptidase 15	8.53565
HEATR6	HEAT Repeat Containing 6	8.53504
PPP1CB	Protein Phosphatase 1 Catalytic Subunit Beta	8.53305
E2F4	E2F Transcription Factor 4	8.53123
AKR1B10	Aldo-Keto Reductase Family 1 Member B10	8.52567
FEN1	Flap Structure-Specific Endonuclease 1	8.52213
AMPH	Amphiphysin	8.52065
F2RL1	F2R Like Trypsin Receptor 1	8.51771
NOG	Noggin	8.51737
SFTPD	Surfactant Protein D	8.50535

TSHR	Thyroid Stimulating Hormone Receptor	8.50330
PRKDC	Protein Kinase, DNA-Activated, Catalytic Subunit	8.49746
OVCA2	OVCA2 Serine Hydrolase Domain Containing	8.49686
FABP4	Fatty Acid Binding Protein 4	8.49486
RPS10	Ribosomal Protein S10	8.49353
ZFP36	ZFP36 Ring Finger Protein	8.49288
IKBKE	Inhibitor Of Nuclear Factor Kappa B Kinase Subunit Epsilon	8.48990
PTGER2	Prostaglandin E Receptor 2	8.48165
ASPSCR1	ASPSCR1 Tether For SLC2A4, UBX Domain Containing	8.47878
CYP2C9	Cytochrome P450 Family 2 Subfamily C Member 9	8.47092
FGF6	Fibroblast Growth Factor 6	8.46677
RHO	Rhodopsin	8.46559
PMAIP1	Phorbol-12-Myristate-13-Acetate-Induced Protein 1	8.46506
ARNT	Aryl Hydrocarbon Receptor Nuclear Translocator	8.46368
RXRG	Retinoid X Receptor Gamma	8.45960
ADRB2	Adrenoceptor Beta 2	8.44837
GPC4	Glypican 4	8.44798
WNT10A	Wnt Family Member 10A	8.44668
ECM1	Extracellular Matrix Protein 1	8.44467
MUC3A	Mucin 3A, Cell Surface Associated	8.44301
THY1	Thy-1 Cell Surface Antigen	8.43169
ADH7	Alcohol Dehydrogenase 7 (Class IV), Mu Or Sigma Polypeptide	8.42362
MIR198	MicroRNA 198	8.42227
ATP8B1	ATPase Phospholipid Transporting 8B1	8.41288
ARHGEF2	Rho/Rac Guanine Nucleotide Exchange Factor 2	8.41187
LAMC1	Laminin Subunit Gamma 1	8.41115
ERCC8	ERCC Excision Repair 8, CSA Ubiquitin Ligase Complex Subunit	8.40661
FUS	FUS RNA Binding Protein	8.40439
AKR7L	Aldo-Keto Reductase Family 7 Like (Gene/Pseudogene)	8.39683
MLANA	Melan-A	8.39457
CCL3	C-C Motif Chemokine Ligand 3	8.39025
CTCF	CCCTC-Binding Factor	8.38835
RBX1	Ring-Box 1	8.38661
PARN	Poly(A)-Specific Ribonuclease	8.38169
PIGR	Polymeric Immunoglobulin Receptor	8.38152
MCM7	Minichromosome Maintenance Complex Component 7	8.38054
MAK	Male Germ Cell Associated Kinase	8.37657

STIM1	Stromal Interaction Molecule 1	8.37068
FAM3C	FAM3 Metabolism Regulating Signaling Molecule C	8.36933
DCN	Decorin	8.36806
ATF4	Activating Transcription Factor 4	8.36307
CKS1B	CDC28 Protein Kinase Regulatory Subunit 1B	8.35154
HSD17B3	Hydroxysteroid 17-Beta Dehydrogenase 3	8.35084
MYH11	Myosin Heavy Chain 11	8.34831
ITGAM	Integrin Subunit Alpha M	8.34750
ZMYND8	Zinc Finger MYND-Type Containing 8	8.34684
TRPM8	Transient Receptor Potential Cation Channel Subfamily M Member 8	8.34220
RUNX1T1	RUNX1 Partner Transcriptional Co-Repressor 1	8.34116
HDAC5	Histone Deacetylase 5	8.33940
FBXO24	F-Box Protein 24	8.33133
ARID2	AT-Rich Interaction Domain 2	8.32837
TPBG	Trophoblast Glycoprotein	8.31582
GPNMB	Glycoprotein Nmb	8.31564
SMARCC2	SWI/SNF Related, Matrix Associated, Actin Dependent Regulator Of Chromatin Subfamily C Member 2	8.31554
GFAP	Glial Fibrillary Acidic Protein	8.31326
MAP3K20-AS1	MAP3K20 Antisense RNA 1	8.31157
OCIAD1	OCIA Domain Containing 1	8.30976
RASSF5	Ras Association Domain Family Member 5	8.30839
PTH1R	Parathyroid Hormone 1 Receptor	8.30830
SERPINB3	Serpin Family B Member 3	8.30291
MCM3	Minichromosome Maintenance Complex Component 3	8.29969
ROR2	Receptor Tyrosine Kinase Like Orphan Receptor 2	8.29463
PRF1	Perforin 1	8.28667
HNRNPA1	Heterogeneous Nuclear Ribonucleoprotein A1	8.28584
CDK2AP1	Cyclin Dependent Kinase 2 Associated Protein 1	8.27914
CD14	CD14 Molecule	8.27262
SRD5A1	Steroid 5 Alpha-Reductase 1	8.27192
S100B	S100 Calcium Binding Protein B	8.26523
MT1DP	Metallothionein 1D, Pseudogene	8.26330
AMHR2	Anti-Mullerian Hormone Receptor Type 2	8.25873
MIR494	MicroRNA 494	8.25334
FKBP5	FKBP Prolyl Isomerase 5	8.25074

REV3L	REV3 Like, DNA Directed Polymerase Zeta Catalytic Subunit	8.24344
LINC01133	Long Intergenic Non-Protein Coding RNA 1133	8.24273
PCGEM1	PCGEM1 Prostate-Specific Transcript	8.23328
EPIST	Esophagus Epithelial Intergenic Associated Transcript	8.22804
HLTF	Helicase Like Transcription Factor	8.21942
TFPI2	Tissue Factor Pathway Inhibitor 2	8.21850
MBL2	Mannose Binding Lectin 2	8.21610
WNT5B	Wnt Family Member 5B	8.21410
SHMT1	Serine Hydroxymethyltransferase 1	8.20950
ZMYND10	Zinc Finger MYND-Type Containing 10	8.20906
NR0B2	Nuclear Receptor Subfamily 0 Group B Member 2	8.20654
RARG	Retinoic Acid Receptor Gamma	8.20588
AMIGO2	Adhesion Molecule With Ig Like Domain 2	8.20439
MIR491	MicroRNA 491	8.19537
LPL	Lipoprotein Lipase	8.19477
PICALM	Phosphatidylinositol Binding Clathrin Assembly Protein	8.19002
AGO2	Argonaute RISC Catalytic Component 2	8.18541
ATP7A	ATPase Copper Transporting Alpha	8.17467
CDK5	Cyclin Dependent Kinase 5	8.16830
LINC00460	Long Intergenic Non-Protein Coding RNA 460	8.15101
PRDX1	Peroxiredoxin 1	8.14503
RPS15	Ribosomal Protein S15	8.13605
SLC9A3R1	SLC9A3 Regulator 1	8.13433
MIR503	MicroRNA 503	8.12999
ARL11	ADP Ribosylation Factor Like GTPase 11	8.12738
RPS29	Ribosomal Protein S29	8.12251
PDX1	Pancreatic And Duodenal Homeobox 1	8.10639
SLC34A2	Solute Carrier Family 34 Member 2	8.10536
CD63	CD63 Molecule	8.09229
TACSTD2	Tumor Associated Calcium Signal Transducer 2	8.09195
PRCC	Proline Rich Mitotic Checkpoint Control Factor	8.09111
OCA2	OCA2 Melanosomal Transmembrane Protein	8.09054
F5	Coagulation Factor V	8.09011
PAGE4	PAGE Family Member 4	8.08242
KIAA0100	KIAA0100	8.08215
CD5	CD5 Molecule	8.07933
MACF1	Microtubule Actin Crosslinking Factor 1	8.07443

MUCL1	Mucin Like 1	8.06696
MAP2K3	Mitogen-Activated Protein Kinase Kinase 3	8.06557
MIR490	MicroRNA 490	8.06386
EPHB6	EPH Receptor B6	8.05966
CXCL13	C-X-C Motif Chemokine Ligand 13	8.05573
LOXL2	Lysyl Oxidase Like 2	8.05396
SYNE1	Spectrin Repeat Containing Nuclear Envelope Protein 1	8.05086
ESRRA	Estrogen Related Receptor Alpha	8.05047
EXT1	Exostosin Glycosyltransferase 1	8.05009
SI	Sucrase-Isomaltase	8.04830
ZNRD1ASP	Zinc Ribbon Domain Containing 1 Antisense, Pseudogene	8.04755
MLF1	Myeloid Leukemia Factor 1	8.04550
PROS1	Protein S	8.03651
MIR138-1	MicroRNA 138-1	8.03360
ZAP70	Zeta Chain Of T Cell Receptor Associated Protein Kinase 70	8.03308
CCL11	C-C Motif Chemokine Ligand 11	8.03194
RBM5	RNA Binding Motif Protein 5	8.03104
SFTA1P	Surfactant Associated 1, LncRNA	8.02245
MTHFD1	Methylenetetrahydrofolate Dehydrogenase, Cyclohydrolase And Formyltetrahydrofolate Synthetase 1	8.02156
EDNRB	Endothelin Receptor Type B	8.01227
TIAM1	TIAM Rac1 Associated GEF 1	7.99833
FOXD2-AS1	FOXD2 Adjacent Opposite Strand RNA 1	7.99743
MDK	Midkine	7.99385
DUXAP8	Double Homeobox A Pseudogene 8	7.98127
NR1I2	Nuclear Receptor Subfamily 1 Group I Member 2	7.97392
MS4A1	Membrane Spanning 4-Domains A1	7.96596
ACVR1	Activin A Receptor Type 1	7.96570
PPARGC1A	PPARG Coactivator 1 Alpha	7.96270
GTF2I	General Transcription Factor IIi	7.96262
LY6K	Lymphocyte Antigen 6 Family Member K	7.94991
HOPX	HOP Homeobox	7.94836
TJP1	Tight Junction Protein 1	7.94731
PLCB1	Phospholipase C Beta 1	7.94483
CTBP1	C-Terminal Binding Protein 1	7.93663
SATB2	SATB Homeobox 2	7.93240
LDOC1	LDOC1 Regulator Of NFKB Signaling	7.92949
CASP6	Caspase 6	7.92879
NBAT1	Neuroblastoma Associated Transcript 1	7.92484
XRCC4	X-Ray Repair Cross Complementing 4	7.92254
POU5F1B	POU Class 5 Homeobox 1B	7.91909

TMC6	Transmembrane Channel Like 6	7.91665
MGAT5	Alpha-1,6-Mannosylglycoprotein 6-Beta-N-Acetylglucosaminyltransferase	7.91242
ATP7B	ATPase Copper Transporting Beta	7.90724
LRP1B	LDL Receptor Related Protein 1B	7.90586
NMB	Neuromedin B	7.90523
FGA	Fibrinogen Alpha Chain	7.90436
TES	Testin LIM Domain Protein	7.90425
GPX3	Glutathione Peroxidase 3	7.89450
OR4C5	Olfactory Receptor Family 4 Subfamily C Member 5	7.88871
G6PD	Glucose-6-Phosphate Dehydrogenase	7.88630
ARRB1	Arrestin Beta 1	7.88317
CNOT9	CCR4-NOT Transcription Complex Subunit 9	7.87700
PLAG1	PLAG1 Zinc Finger	7.87656
MIR33A	MicroRNA 33a	7.86580
FUT4	Fucosyltransferase 4	7.86433
HOXB9	Homeobox B9	7.86317
MIR125B2	MicroRNA 125b-2	7.86300
TIE1	Tyrosine Kinase With Immunoglobulin Like And EGF Like Domains 1	7.85983
IL2RB	Interleukin 2 Receptor Subunit Beta	7.85234
PRKD3	Protein Kinase D3	7.85006
WNT7B	Wnt Family Member 7B	7.83695
TNFRSF13B	TNF Receptor Superfamily Member 13B	7.83327
IL12RB1	Interleukin 12 Receptor Subunit Beta 1	7.83065
FOXK2	Forkhead Box K2	7.82481
PKD1	Polycystin 1, Transient Receptor Potential Channel Interacting	7.82413
HNRNPA2B1	Heterogeneous Nuclear Ribonucleoprotein A2/B1	7.82179
MICA	MHC Class I Polypeptide-Related Sequence A	7.81506
AFDN-DT	AFDN Divergent Transcript	7.81439
UBE3A	Ubiquitin Protein Ligase E3A	7.81297
WNT11	Wnt Family Member 11	7.81196
INHBA-AS1	INHBA Antisense RNA 1	7.81059
MIR148B	MicroRNA 148b	7.81049
SALL4	Spalt Like Transcription Factor 4	7.80638
VDAC1	Voltage Dependent Anion Channel 1	7.80531
PTPRG	Protein Tyrosine Phosphatase Receptor Type G	7.80511
SCAT2	S-Phase Cancer Associated Transcript 2	7.80123
IL7R	Interleukin 7 Receptor	7.80078
CCDC26	CCDC26 Long Non-Coding RNA	7.79957
ACTG1	Actin Gamma 1	7.79665
EPHA1	EPH Receptor A1	7.79341

ARFGEF3	ARFGEF Family Member 3	7.79043
MDH2	Malate Dehydrogenase 2	7.78263
KIF20B	Kinesin Family Member 20B	7.77721
PRRC2A	Proline Rich Coiled-Coil 2A	7.77190
LINC01006	Long Intergenic Non-Protein Coding RNA 1006	7.76946
AKAP9	A-Kinase Anchoring Protein 9	7.76757
CLOCK	Clock Circadian Regulator	7.76333
LPAR3	Lysophosphatidic Acid Receptor 3	7.76064
SOX10	SRY-Box Transcription Factor 10	7.75659
TERF1	Telomeric Repeat Binding Factor 1	7.75105
TBX3	T-Box Transcription Factor 3	7.75065
CDCP1	CUB Domain Containing Protein 1	7.74631
DACT1	Dishevelled Binding Antagonist Of Beta Catenin 1	7.74494
ANXA3	Annexin A3	7.74465
STAT4	Signal Transducer And Activator Of Transcription 4	7.74370
TNFRSF10A-A	TNFRSF10A Antisense RNA 1	7.74321
SLC5A4-AS1	SLC5A4 Antisense RNA 1	7.73973
CDH5	Cadherin 5	7.73806
VPS9D1-AS1	VPS9D1 Antisense RNA 1	7.73783
BCAS2	BCAS2 Pre-MRNA Processing Factor	7.73716
MIR512-1	MicroRNA 512-1	7.72239
GSTO2	Glutathione S-Transferase Omega 2	7.71965
HELLS	Helicase, Lymphoid Specific	7.71216
PWRN1	Prader-Willi Region Non-Protein Coding RNA 1	7.71108
PLCG2	Phospholipase C Gamma 2	7.70677
PCNA-AS1	PCNA Antisense RNA 1	7.70572
INPPL1	Inositol Polyphosphate Phosphatase Like 1	7.70284
SOX11	SRY-Box Transcription Factor 11	7.70256
MIR495	MicroRNA 495	7.70193
COL4A3	Collagen Type IV Alpha 3 Chain	7.70147
KIF15	Kinesin Family Member 15	7.69829
INTS6	Integrator Complex Subunit 6	7.69597
SLC7A11-AS1	SLC7A11 Antisense RNA 1	7.69216
EIF2AK2	Eukaryotic Translation Initiation Factor 2 Alpha Kinase 2	7.68451
IAPP	Islet Amyloid Polypeptide	7.67957
AIFM1	Apoptosis Inducing Factor Mitochondria Associated 1	7.67934
SFRP2	Secreted Frizzled Related Protein 2	7.67926
MIR129-2	MicroRNA 129-2	7.67880
ERGIC1	Endoplasmic Reticulum-Golgi Intermediate Compartment 1	7.67103
TLR7	Toll Like Receptor 7	7.66978
CXCL10	C-X-C Motif Chemokine Ligand 10	7.66457

PODXL	Podocalyxin Like	7.66027
ID3	Inhibitor Of DNA Binding 3, HLH Protein	7.65881
MIR17HG	MiR-17-92a-1 Cluster Host Gene	7.65661
SCAT8	S-Phase Cancer Associated Transcript 8	7.65124
S100A7	S100 Calcium Binding Protein A7	7.64994
HPRT1	Hypoxanthine Phosphoribosyltransferase 1	7.64977
LPAR1	Lysophosphatidic Acid Receptor 1	7.64897
RPS24	Ribosomal Protein S24	7.64887
BNC2-AS1	BNC2 Antisense RNA 1	7.64857
IREB2	Iron Responsive Element Binding Protein 2	7.64694
PLCB4	Phospholipase C Beta 4	7.64037
TMPRSS11A	Transmembrane Serine Protease 11A	7.63994
TNK2	Tyrosine Kinase Non Receptor 2	7.63978
SLC4A2	Solute Carrier Family 4 Member 2	7.63363
FST	Follistatin	7.62581
S100A14	S100 Calcium Binding Protein A14	7.62531
UPK2	Uroplakin 2	7.62160
FGF10	Fibroblast Growth Factor 10	7.62144
KLK7	Kallikrein Related Peptidase 7	7.62117
PER2	Period Circadian Regulator 2	7.61925
SUZ12	SUZ12 Polycomb Repressive Complex 2 Subunit	7.61464
IGF2-AS	IGF2 Antisense RNA	7.61065
CXADR	CXADR Ig-Like Cell Adhesion Molecule	7.61006
TRERNA1	Translation Regulatory Long Non-Coding RNA 1	7.60991
CP	Ceruloplasmin	7.60733
PRDM16	PR/SET Domain 16	7.59883
ZNF217	Zinc Finger Protein 217	7.59738
ETV5	ETS Variant Transcription Factor 5	7.58636
ROR1	Receptor Tyrosine Kinase Like Orphan Receptor 1	7.58580
LOC10192975[illegible]	Uncharacterized LOC101929759	7.58251
MAPK12	Mitogen-Activated Protein Kinase 12	7.57779
SSTR1	Somatostatin Receptor 1	7.57738
HSPB2	Heat Shock Protein Family B (Small) Member 2	7.57692
ENSG00000266990		7.57114
ADAM9	ADAM Metallopeptidase Domain 9	7.56917
PTN	Pleiotrophin	7.56803
KRT7-AS	KRT7 Antisense RNA 1	7.56729
OLFM4	Olfactomedin 4	7.56603
SLC7A5	Solute Carrier Family 7 Member 5	7.56598
MIR584	MicroRNA 584	7.56198

HSPB8	Heat Shock Protein Family B (Small) Member 8	7.56095
MNX1-AS1	MNX1 Antisense RNA 1 (Head To Head)	7.55873
ZEB2-AS1	ZEB2 Antisense RNA 1	7.55637
NUDC	Nuclear Distribution C, Dynein Complex Regulator	7.55321
TACC2	Transforming Acidic Coiled-Coil Containing Protein 2	7.54853
LINC00668	Long Intergenic Non-Protein Coding RNA 668	7.53633
SMIM31	Small Integral Membrane Protein 31	7.53414
CLTC	Clathrin Heavy Chain	7.53119
WFS1	Wolframin ER Transmembrane Glycoprotein	7.52657
TRPV4	Transient Receptor Potential Cation Channel Subfamily V Member 4	7.52580
SF3B2	Splicing Factor 3b Subunit 2	7.52545
USH2A	Usherin	7.52530
KNL1	Kinetochore Scaffold 1	7.52436
FGF23	Fibroblast Growth Factor 23	7.52192
TNFSF12	TNF Superfamily Member 12	7.51768
RN7SK	RNA Component Of 7SK Nuclear Ribonucleoprotein	7.51556
TPO	Thyroid Peroxidase	7.51541
SCGB1A1	Secretoglobin Family 1A Member 1	7.50553
KLK8	Kallikrein Related Peptidase 8	7.50040
LPP	LIM Domain Containing Preferred Translocation Partner In Lipoma	7.49855
PRAME	PRAME Nuclear Receptor Transcriptional Regulator	7.49622
TFAP2C	Transcription Factor AP-2 Gamma	7.49592
USP8	Ubiquitin Specific Peptidase 8	7.49084
ENSG00000287486		7.48745
HNRNPU	Heterogeneous Nuclear Ribonucleoprotein U	7.48678
LINC00572	Long Intergenic Non-Protein Coding RNA 572	7.48668
BTRC	Beta-Transducin Repeat Containing E3 Ubiquitin Protein Ligase	7.48645
TCONS_00068	Uncharacterized TCONS_00068220	7.48058
F2RL3	F2R Like Thrombin Or Trypsin Receptor 3	7.47752
SGO1	Shugoshin 1	7.47730
IL12A	Interleukin 12A	7.47596
USP7	Ubiquitin Specific Peptidase 7	7.47226
IDO1	Indoleamine 2,3-Dioxygenase 1	7.47151
TRIM25	Tripartite Motif Containing 25	7.47066
MACC1-AS1	MACC1 Antisense RNA 1	7.46093

NRP2	Neuropilin 2	7.45002
PANTR1	POU3F3 Adjacent Non-Coding Transcript 1	7.44487
LDB1	LIM Domain Binding 1	7.44032
PHLPP2	PH Domain And Leucine Rich Repeat Protein Phosphatase 2	7.43975
LRP1	LDL Receptor Related Protein 1	7.43279
MIR615	MicroRNA 615	7.43026
MORC2	MORC Family CW-Type Zinc Finger 2	7.42931
PLD2	Phospholipase D2	7.42931
SLC5A5	Solute Carrier Family 5 Member 5	7.42875
ADNP	Activity Dependent Neuroprotector Homeobox	7.42720
ZMAT1	Zinc Finger Matrin-Type 1	7.41658
ABI1	Abl Interactor 1	7.41410
LINC01612	Long Intergenic Non-Protein Coding RNA 1612	7.41352
EPB41L3	Erythrocyte Membrane Protein Band 4.1 Like 3	7.41249
LPAR2	Lysophosphatidic Acid Receptor 2	7.41143
TRAF4	TNF Receptor Associated Factor 4	7.39966
JUND	JunD Proto-Oncogene, AP-1 Transcription Factor Subunit	7.39470
XDH	Xanthine Dehydrogenase	7.37052
TNFRSF11A	TNF Receptor Superfamily Member 11a	7.36884
LINC00858	Long Intergenic Non-Protein Coding RNA 858	7.36862
ACSL4	Acyl-CoA Synthetase Long Chain Family Member 4	7.36823
NTN1	Netrin 1	7.36761
CDA	Cytidine Deaminase	7.36676
NLRP3	NLR Family Pyrin Domain Containing 3	7.36647
CERK	Ceramide Kinase	7.36188
KRT1	Keratin 1	7.35838
TLR10	Toll Like Receptor 10	7.35735
NBR2	Neighbor Of BRCA1 LncRNA 2	7.35622
GPR65	G Protein-Coupled Receptor 65	7.35571
CUL5	Cullin 5	7.35393
CALD1	Caldesmon 1	7.35270
H1-0	H1.0 Linker Histone	7.35214
BMP10	Bone Morphogenetic Protein 10	7.35188
INSL3	Insulin Like 3	7.35152
FOLR1	Folate Receptor Alpha	7.34749
KRT13	Keratin 13	7.34735
KRT18P55	Keratin 18 Pseudogene 55	7.34683
CD247	CD247 Molecule	7.34667
CD248	CD248 Molecule	7.34449
SPEN	Spen Family Transcriptional Repressor	7.34237

RBL1	RB Transcriptional Corepressor Like 1	7.34190
S100A1	S100 Calcium Binding Protein A1	7.34070
ROCK2	Rho Associated Coiled-Coil Containing Protein Kinase 2	7.34005
LCK	LCK Proto-Oncogene, Src Family Tyrosine Kinase	7.33985
ARSH	Arylsulfatase Family Member H	7.33775
STARD3	StAR Related Lipid Transfer Domain Containing 3	7.33435
ENTPD1-AS1	ENTPD1 Antisense RNA 1	7.33343
ARMC5	Armadillo Repeat Containing 5	7.33184
IFI16	Interferon Gamma Inducible Protein 16	7.32939
AICDA	Activation Induced Cytidine Deaminase	7.32741
UGT1A6	UDP Glucuronosyltransferase Family 1 Member A6	7.32727
TAC1	Tachykinin Precursor 1	7.31714
MIR22HG	MIR22 Host Gene	7.31678
UPK3A	Uroplakin 3A	7.30686
ABCA1	ATP Binding Cassette Subfamily A Member 1	7.30665
FPGS	Folylpolyglutamate Synthase	7.30544
GUSB	Glucuronidase Beta	7.30265
RAC3	Rac Family Small GTPase 3	7.30111
SUSD2	Sushi Domain Containing 2	7.29998
MIR219A1	MicroRNA 219a-1	7.29820
PON1	Paraoxonase 1	7.29076
CDC27	Cell Division Cycle 27	7.29045
RETN	Resistin	7.28285
MUC12	Mucin 12, Cell Surface Associated	7.28206
AMH	Anti-Mullerian Hormone	7.27598
GNB3	G Protein Subunit Beta 3	7.27380
HBB	Hemoglobin Subunit Beta	7.26146
ATF2	Activating Transcription Factor 2	7.24750
CD276	CD276 Molecule	7.24446
LMO1	LIM Domain Only 1	7.24187
RUNX1-IT1	RUNX1 Intronic Transcript 1	7.24069
COL1A2	Collagen Type I Alpha 2 Chain	7.24054
KLK11	Kallikrein Related Peptidase 11	7.23839
CRH	Corticotropin Releasing Hormone	7.23603
TYRO3	TYRO3 Protein Tyrosine Kinase	7.23423
SRRM3	Serine/Arginine Repetitive Matrix 3	7.23391
KDM5A	Lysine Demethylase 5A	7.23263
CALML4	Calmodulin Like 4	7.23190
RAP1A	RAP1A, Member Of RAS Oncogene Family	7.22026
DSP	Desmoplakin	7.21950
IRF2BP2	Interferon Regulatory Factor 2 Binding Protein 2	7.20956

G6PC1	Glucose-6-Phosphatase Catalytic Subunit 1	7.20935
ENSG00000253389		7.20583
MIR95	MicroRNA 95	7.20282
GSTA1	Glutathione S-Transferase Alpha 1	7.20258
PTPRK	Protein Tyrosine Phosphatase Receptor Type K	7.20234
AKR1C1	Aldo-Keto Reductase Family 1 Member C1	7.19974
SPOP	Speckle Type BTB/POZ Protein	7.19292
ALKBH3	AlkB Homolog 3, Alpha-Ketoglutarate Dependent Dioxygenase	7.19223
MIR337	MicroRNA 337	7.18877
MBD4	Methyl-CpG Binding Domain 4, DNA Glycosylase	7.18270
SPHK2	Sphingosine Kinase 2	7.18125
GPR19	G Protein-Coupled Receptor 19	7.18027
FLNB	Filamin B	7.17929
CSF3R	Colony Stimulating Factor 3 Receptor	7.17523
LINC-PINT	Long Intergenic Non-Protein Coding RNA, P53 Induced Transcript	7.17307
VIPR1	Vasoactive Intestinal Peptide Receptor 1	7.17216
IFNGR1	Interferon Gamma Receptor 1	7.16829
CCDC170	Coiled-Coil Domain Containing 170	7.16505
NACC1	Nucleus Accumbens Associated 1	7.16159
IFNAR1	Interferon Alpha And Beta Receptor Subunit 1	7.15966
MIR181D	MicroRNA 181d	7.15762
PFAS	Phosphoribosylformylglycinamidine Synthase	7.15640
TBL1XR1	TBL1X Receptor 1	7.15329
SCTR	Secretin Receptor	7.14652
FZD10	Frizzled Class Receptor 10	7.14164
GNB5	G Protein Subunit Beta 5	7.13772
SILC1	Sciatic Injury Induced LincRNA Upregulator Of SOX11	7.13430
LOC10537244(	Uncharacterized LOC105372446	7.13305
ATG5	Autophagy Related 5	7.12784
LIG3	DNA Ligase 3	7.12692
CISD2	CDGSH Iron Sulfur Domain 2	7.12655
PIAS3	Protein Inhibitor Of Activated STAT 3	7.12429
ZFHX4-AS1	ZFHX4 Antisense RNA 1	7.11939
TBC1D3	TBC1 Domain Family Member 3	7.11707
KLK13	Kallikrein Related Peptidase 13	7.11595
PDCD1LG2	Programmed Cell Death 1 Ligand 2	7.11468
MIR498	MicroRNA 498	7.11293
ANXA2P2	Annexin A2 Pseudogene 2	7.11288
MIR154	MicroRNA 154	7.10635
TH	Tyrosine Hydroxylase	7.10570

A2M-AS1	A2M Antisense RNA 1	7.10281
KLHDC8B	Kelch Domain Containing 8B	7.10015
MTRR	5-Methyltetrahydrofolate-Homocysteine Methyltransferase Reductase	7.09468
LGR6	Leucine Rich Repeat Containing G Protein-Coupled Receptor 6	7.08684
LSP1	Lymphocyte Specific Protein 1	7.08471
ELOC	Elongin C	7.08464
TNFRSF9	TNF Receptor Superfamily Member 9	7.08095
LINC01194	Long Intergenic Non-Protein Coding RNA 1194	7.07895
TUSC1	Tumor Suppressor Candidate 1	7.07305
WASF3	WASP Family Member 3	7.07047
RAG2	Recombination Activating 2	7.06690
TAGLN	Transgelin	7.06661
SMAD1	SMAD Family Member 1	7.06577
MIR216A	MicroRNA 216a	7.06470
GTF2IRD1	GTF2I Repeat Domain Containing 1	7.06355
CCL21	C-C Motif Chemokine Ligand 21	7.04547
KAT2B	Lysine Acetyltransferase 2B	7.04072
ID4	Inhibitor Of DNA Binding 4, HLH Protein	7.04047
BCOR	BCL6 Corepressor	7.03690
SPINK7	Serine Peptidase Inhibitor Kazal Type 7	7.03399
TMSB4X	Thymosin Beta 4 X-Linked	7.03394
COX7A2L	Cytochrome C Oxidase Subunit 7A2 Like	7.03393
ZFP36L2	ZFP36 Ring Finger Protein Like 2	7.03350
IL1R1	Interleukin 1 Receptor Type 1	7.03261
ENSG00000232406		7.03021
TSNAX-DISC1	TSNAX-DISC1 Readthrough (NMD Candidate)	7.03011
PCLAF	PCNA Clamp Associated Factor	7.02911
EEF1A2	Eukaryotic Translation Elongation Factor 1 Alpha 2	7.02814
ANG	Angiogenin	7.02574
AGT	Angiotensinogen	7.02399
TUBB2A	Tubulin Beta 2A Class IIa	7.02276
GLUL	Glutamate-Ammonia Ligase	7.02074
SAA1	Serum Amyloid A1	7.02073
TGFBR3	Transforming Growth Factor Beta Receptor 3	7.01475
TREX1	Three Prime Repair Exonuclease 1	7.01248
IL12B	Interleukin 12B	7.01221
SPA17	Sperm Autoantigenic Protein 17	7.00466
TGM1	Transglutaminase 1	7.00432
SELP	Selectin P	7.00052
NR0B1	Nuclear Receptor Subfamily 0 Group B Member 1	6.99132

L1CAM	L1 Cell Adhesion Molecule	6.98995
LIN28A	Lin-28 Homolog A	6.97891
EYA2	EYA Transcriptional Coactivator And Phosphatase 2	6.97357
ENSG00000285159		6.97133
CUL3	Cullin 3	6.97082
LGALS4	Galectin 4	6.97032
TAB1	TGF-Beta Activated Kinase 1 (MAP3K7) Binding Protein 1	6.96344
MUC7	Mucin 7, Secreted	6.95979
WNT16	Wnt Family Member 16	6.95201
REG1A	Regenerating Family Member 1 Alpha	6.95186
SUCLG2-AS1	SUCLG2 Antisense RNA 1 (Head To Head)	6.94907
SH3RF3-AS1	SH3RF3 Antisense RNA 1	6.94907
LINC01097	Long Intergenic Non-Protein Coding RNA 1097	6.94907
SEMA3F	Semaphorin 3F	6.94715
NR5A1	Nuclear Receptor Subfamily 5 Group A Member 1	6.94713
ACVR2A	Activin A Receptor Type 2A	6.94486
LIG1	DNA Ligase 1	6.94242
CCNB2	Cyclin B2	6.93997
PTPRH	Protein Tyrosine Phosphatase Receptor Type H	6.93913
ENPP1	Ectonucleotide Pyrophosphatase/Phosphodiesterase 1	6.93912
MYLK-AS1	MYLK Antisense RNA 1	6.93848
LINC02461	Long Intergenic Non-Protein Coding RNA 2461	6.93828
USF2	Upstream Transcription Factor 2, C-Fos Interacting	6.93655
MIR663A	MicroRNA 663a	6.92830
WNT9B	Wnt Family Member 9B	6.92806
CTC1	CST Telomere Replication Complex Component 1	6.92603
TLR8	Toll Like Receptor 8	6.92602
HTR2A	5-Hydroxytryptamine Receptor 2A	6.92601
ENSG00000229717		6.92549
LINC01856	Long Intergenic Non-Protein Coding RNA 1856	6.92549
ENSG00000277200		6.92549
ATRIP	ATR Interacting Protein	6.91291
RALBP1	RalA Binding Protein 1	6.90781
CEMIP	Cell Migration Inducing Hyaluronidase 1	6.90719
SBDS	SBDS Ribosome Maturation Factor	6.90717
IL32	Interleukin 32	6.89795
NAPSA	Napsin A Aspartic Peptidase	6.89670

TGFB1I1	Transforming Growth Factor Beta 1 Induced Transcript 1	6.89477
IL6ST	Interleukin 6 Cytokine Family Signal Transducer	6.89265
PLK4	Polo Like Kinase 4	6.88888
MAP3K2	Mitogen-Activated Protein Kinase Kinase Kinase 2	6.88440
DVL3	Dishevelled Segment Polarity Protein 3	6.88135
CUEDC2	CUE Domain Containing 2	6.87951
AGK	Acylglycerol Kinase	6.87559
FOXF1	Forkhead Box F1	6.86980
SLC52A3	Solute Carrier Family 52 Member 3	6.86933
IGHV4-38-2	Immunoglobulin Heavy Variable 4-38-2	6.86817
NEDD4	NEDD4 E3 Ubiquitin Protein Ligase	6.86577
ENSG00000250406		6.86379
ZNRF3	Zinc And Ring Finger 3	6.85785
NPY	Neuropeptide Y	6.85526
PTGES2	Prostaglandin E Synthase 2	6.84824
CTSK	Cathepsin K	6.84590
MATK	Megakaryocyte-Associated Tyrosine Kinase	6.84359
KMT2D	Lysine Methyltransferase 2D	6.84085
PRDX3	Peroxiredoxin 3	6.84066
IL13RA2	Interleukin 13 Receptor Subunit Alpha 2	6.83929
TRAF1	TNF Receptor Associated Factor 1	6.83700
DNAH8	Dynein Axonemal Heavy Chain 8	6.83614
GPC6	Glypican 6	6.83421
EIF3A	Eukaryotic Translation Initiation Factor 3 Subunit A	6.83197
SLCO1B3	Solute Carrier Organic Anion Transporter Family Member 1B3	6.83174
CGA	Glycoprotein Hormones, Alpha Polypeptide	6.83053
CUBN	Cubilin	6.83008
BACH1	BTB Domain And CNC Homolog 1	6.82724
ELAPOR1	Endosome-Lysosome Associated Apoptosis And Autophagy Regulator 1	6.82671
HSD3B2	Hydroxy-Delta-5-Steroid Dehydrogenase, 3 Beta- And Steroid Delta-Isomerase 2	6.82590
AGTR1	Angiotensin II Receptor Type 1	6.82103
PRDX2	Peroxiredoxin 2	6.81910
KRT10	Keratin 10	6.81893
COL14A1	Collagen Type XIV Alpha 1 Chain	6.81478
CCR1	C-C Motif Chemokine Receptor 1	6.81472
AKAP12	A-Kinase Anchoring Protein 12	6.80922
MCAM	Melanoma Cell Adhesion Molecule	6.80901

TUBB3	Tubulin Beta 3 Class III	6.80787
PEA15	Proliferation And Apoptosis Adaptor Protein 15	6.80663
TMPRSS3	Transmembrane Serine Protease 3	6.80479
EIF3H	Eukaryotic Translation Initiation Factor 3 Subunit H	6.80421
HJURP	Holliday Junction Recognition Protein	6.80375
TF	Transferrin	6.80130
SYT7	Synaptotagmin 7	6.79997
SPAG9	Sperm Associated Antigen 9	6.79825
RPL6	Ribosomal Protein L6	6.78789
BIRC6	Baculoviral IAP Repeat Containing 6	6.78542
PAX8-AS1	PAX8 Antisense RNA 1	6.78514
DMPK	DM1 Protein Kinase	6.78505
FZD1	Frizzled Class Receptor 1	6.78434
CD68	CD68 Molecule	6.78424
DEK	DEK Proto-Oncogene	6.78217
RASA2	RAS P21 Protein Activator 2	6.78217
GRK2	G Protein-Coupled Receptor Kinase 2	6.77695
RASGRP1	RAS Guanyl Releasing Protein 1	6.77031
UIMC1	Ubiquitin Interaction Motif Containing 1	6.76935
RIOX1	Ribosomal Oxygenase 1	6.76737
MRTFA	Myocardin Related Transcription Factor A	6.76671
SLC16A7	Solute Carrier Family 16 Member 7	6.76270
THBD	Thrombomodulin	6.76185
TRRAP	Transformation/Transcription Domain Associated Protein	6.75919
NEDD9	Neural Precursor Cell Expressed, Developmentally Down-Regulated 9	6.75434
MIR425	MicroRNA 425	6.75404
AOC3	Amine Oxidase Copper Containing 3	6.74707
IKZF3	IKAROS Family Zinc Finger 3	6.74319
GPX2	Glutathione Peroxidase 2	6.73595
GPRC5A	G Protein-Coupled Receptor Class C Group 5 Member A	6.73439
PRSS50	Serine Protease 50	6.73334
MIR218-2	MicroRNA 218-2	6.72640
DVL2	Dishevelled Segment Polarity Protein 2	6.72633
LINC00312	Long Intergenic Non-Protein Coding RNA 312	6.72539
KLC1	Kinesin Light Chain 1	6.72466
RSPO1	R-Spondin 1	6.72290
PRKCZ	Protein Kinase C Zeta	6.71195
RPS14	Ribosomal Protein S14	6.71162
RARRES1	Retinoic Acid Receptor Responder 1	6.69861
LUC7L2	LUC7 Like 2, Pre-MRNA Splicing Factor	6.68989

RPL31	Ribosomal Protein L31	6.68970
LINC01554	Long Intergenic Non-Protein Coding RNA 1554	6.68507
QKI	QKI, KH Domain Containing RNA Binding	6.68325
BPIFA1	BPI Fold Containing Family A Member 1	6.68314
COL4A5	Collagen Type IV Alpha 5 Chain	6.67833
SGK3	Serum/Glucocorticoid Regulated Kinase Family Member 3	6.67804
MIR134	MicroRNA 134	6.67208
TTN-AS1	TTN Antisense RNA 1	6.66055
TRPV1	Transient Receptor Potential Cation Channel Subfamily V Member 1	6.65443
MIR301A	MicroRNA 301a	6.65111
SETDB1	SET Domain Bifurcated Histone Lysine Methyltransferase 1	6.65102
ST6GAL1	ST6 Beta-Galactoside Alpha-2,6-Sialyltransferase 1	6.64219
COTL1	Coactosin Like F-Actin Binding Protein 1	6.63354
PPP2R2A	Protein Phosphatase 2 Regulatory Subunit Balpha	6.63285
KRT4	Keratin 4	6.62760
RASSF2	Ras Association Domain Family Member 2	6.62102
ZNF224	Zinc Finger Protein 224	6.61676
IGHMBP2	Immunoglobulin Mu DNA Binding Protein 2	6.61609
LYN	LYN Proto-Oncogene, Src Family Tyrosine Kinase	6.61356
PRSS8	Serine Protease 8	6.61248
RIT1	Ras Like Without CAAX 1	6.61100
KLF10	Kruppel Like Factor 10	6.60400
TYK2	Tyrosine Kinase 2	6.59681
DRD2	Dopamine Receptor D2	6.59645
EHMT2	Euchromatic Histone Lysine Methyltransferase 2	6.59628
FAT4	FAT Atypical Cadherin 4	6.59507
TXNRD1	Thioredoxin Reductase 1	6.58992
SLC6A4	Solute Carrier Family 6 Member 4	6.58698
HK1	Hexokinase 1	6.58285
SKI	SKI Proto-Oncogene	6.58047
FZD6	Frizzled Class Receptor 6	6.57699
KLF17	Kruppel Like Factor 17	6.57503
CDH11	Cadherin 11	6.57049
MIRLET7A2	MicroRNA Let-7a-2	6.56770
MACROH2A1	MacroH2A.1 Histone	6.56766
GNAI2	G Protein Subunit Alpha I2	6.56315

ASCC1	Activating Signal Cointegrator 1 Complex Subunit 1	6.56063
RFWD3	Ring Finger And WD Repeat Domain 3	6.56043
SAFB	Scaffold Attachment Factor B	6.55830
KDM6A	Lysine Demethylase 6A	6.55248
NOX1	NADPH Oxidase 1	6.55206
HOXA5	Homeobox A5	6.55185
LINC01116	Long Intergenic Non-Protein Coding RNA 1116	6.54556
SAG	S-Antigen Visual Arrestin	6.54262
IRS4	Insulin Receptor Substrate 4	6.54016
RINT1	RAD50 Interactor 1	6.53992
GRM1	Glutamate Metabotropic Receptor 1	6.53943
ALPL	Alkaline Phosphatase, Biomineralization Associated	6.53553
ACRBP	Acrosin Binding Protein	6.53452
HOXA10	Homeobox A10	6.53292
ARHGAP26	Rho GTPase Activating Protein 26	6.53036
EFEMP1	EGF Containing Fibulin Extracellular Matrix Protein 1	6.52996
KCNJ11	Potassium Inwardly Rectifying Channel Subfamily J Member 11	6.52982
APPBP2	Amyloid Beta Precursor Protein Binding Protein 2	6.52342
XAGE1B	X Antigen Family Member 1B	6.51558
ABCB11	ATP Binding Cassette Subfamily B Member 11	6.51486
ICOS	Inducible T Cell Costimulator	6.51303
CTRC	Chymotrypsin C	6.51031
HIP1R	Huntingtin Interacting Protein 1 Related	6.50577
MIR708	MicroRNA 708	6.50493
FLG	Filaggrin	6.49360
GSE1	Gse1 Coiled-Coil Protein	6.48506
MIR376C	MicroRNA 376c	6.47645
CHIC2	Cysteine Rich Hydrophobic Domain 2	6.47412
FCGR3A	Fc Fragment Of IgG Receptor IIIa	6.47220
ATOH1	Atonal BHLH Transcription Factor 1	6.47184
PNP	Purine Nucleoside Phosphorylase	6.47138
IRF5	Interferon Regulatory Factor 5	6.47074
MIR371A	MicroRNA 371a	6.46870
BMX	BMX Non-Receptor Tyrosine Kinase	6.46562
RPL26	Ribosomal Protein L26	6.45914
STAG3	Stromal Antigen 3	6.45570
PROX1	Prospero Homeobox 1	6.45110
ECE1	Endothelin Converting Enzyme 1	6.44938
SBF2-AS1	SBF2 Antisense RNA 1	6.44880
ESRRG	Estrogen Related Receptor Gamma	6.44736
SCAP	SREBF Chaperone	6.44588

KCNH2	Potassium Voltage-Gated Channel Subfamily H Member 2	6.44530
NUPR1	Nuclear Protein 1, Transcriptional Regulator	6.43789
FYN	FYN Proto-Oncogene, Src Family Tyrosine Kinase	6.43300
FOXP4-AS1	FOXP4 Antisense RNA 1	6.43240
IGF2BP2	Insulin Like Growth Factor 2 MRNA Binding Protein 2	6.43231
COPS5	COP9 Signalosome Subunit 5	6.43153
RRP1B	Ribosomal RNA Processing 1B	6.43058
PLAGL1	PLAG1 Like Zinc Finger 1	6.42732
SF3A1	Splicing Factor 3a Subunit 1	6.42353
ITGA9	Integrin Subunit Alpha 9	6.41654
RPL18	Ribosomal Protein L18	6.41634
UBA7	Ubiquitin Like Modifier Activating Enzyme 7	6.41559
HSD3B1	Hydroxy-Delta-5-Steroid Dehydrogenase, 3 Beta- And Steroid Delta-Isomerase 1	6.40790
RAD17	RAD17 Checkpoint Clamp Loader Component	6.38997
EPSTI1	Epithelial Stromal Interaction 1	6.38526
SH3PXD2A	SH3 And PX Domains 2A	6.38392
LETMD1	LETM1 Domain Containing 1	6.38326
KL	Klotho	6.37423
CX3CR1	C-X3-C Motif Chemokine Receptor 1	6.37399
SOX5	SRY-Box Transcription Factor 5	6.37355
ZNF703	Zinc Finger Protein 703	6.37341
RBL2	RB Transcriptional Corepressor Like 2	6.36780
NUMB	NUMB Endocytic Adaptor Protein	6.36516
ZFR	Zinc Finger RNA Binding Protein	6.36285
PMEPA1	Prostate Transmembrane Protein, Androgen Induced 1	6.36282
TTLL12	Tubulin Tyrosine Ligase Like 12	6.35623
FDPS	Farnesyl Diphosphate Synthase	6.35553
MIR136	MicroRNA 136	6.34603
ACTA1	Actin Alpha 1, Skeletal Muscle	6.34284
PRNP	Prion Protein	6.34059
DGCR5	DiGeorge Syndrome Critical Region Gene 5	6.33833
RGMB-AS1	RGMB Antisense RNA 1	6.33395
JAZF1	JAZF Zinc Finger 1	6.33042
APOBEC3G	Apolipoprotein B MRNA Editing Enzyme Catalytic Subunit 3G	6.32949
RBBP6	RB Binding Protein 6, Ubiquitin Ligase	6.32843
SLIT2	Slit Guidance Ligand 2	6.32480
CYP17A1-AS1	CYP17A1 Antisense RNA 1	6.32413
GSTO1	Glutathione S-Transferase Omega 1	6.32144

ENDOG	Endonuclease G	6.32091
S100A11	S100 Calcium Binding Protein A11	6.32089
PLK3	Polo Like Kinase 3	6.31888
RBP1	Retinol Binding Protein 1	6.31731
MIR199A2	MicroRNA 199a-2	6.31528
UGT2B15	UDP Glucuronosyltransferase Family 2 Member B15	6.31206
ANGPTL4	Angiopoietin Like 4	6.31118
FAM72D	Family With Sequence Similarity 72 Member D	6.30658
SLC39A1	Solute Carrier Family 39 Member 1	6.30193
DEFB4A	Defensin Beta 4A	6.29962
PUM1	Pumilio RNA Binding Family Member 1	6.29837
CMIP	C-Maf Inducing Protein	6.29708
GSDME	Gasdermin E	6.29510
MSI1	Musashi RNA Binding Protein 1	6.29476
FUT6	Fucosyltransferase 6	6.28905
FZR1	Fizzy And Cell Division Cycle 20 Related 1	6.28454
HOXA11	Homeobox A11	6.27839
COL4A1	Collagen Type IV Alpha 1 Chain	6.27098
FIP1L1	Factor Interacting With PAPOLA And CPSF1	6.26971
SLC39A6	Solute Carrier Family 39 Member 6	6.26908
IGFBP6	Insulin Like Growth Factor Binding Protein 6	6.26612
HACE1	HECT Domain And Ankyrin Repeat Containing E3 Ubiquitin Protein Ligase 1	6.26535
ABCC6	ATP Binding Cassette Subfamily C Member 6	6.26343
UBC	Ubiquitin C	6.26258
ERCC6L2	ERCC Excision Repair 6 Like 2	6.26216
ADM	Adrenomedullin	6.26202
SDF4	Stromal Cell Derived Factor 4	6.26104
NR4A1	Nuclear Receptor Subfamily 4 Group A Member 1	6.26017
HOXA9	Homeobox A9	6.25756
MT-TE	Mitochondrially Encoded TRNA-Glu (GAA/G)	6.25639
SLC9A1	Solute Carrier Family 9 Member A1	6.25575
CD27	CD27 Molecule	6.25485
MIR381	MicroRNA 381	6.25110
MAPRE1	Microtubule Associated Protein RP/EB Family Member 1	6.23024
PPIA	Peptidylprolyl Isomerase A	6.22811
CST6	Cystatin E/M	6.22496
XAGE1A	X Antigen Family Member 1A	6.22489

UNG	Uracil DNA Glycosylase	6.22295
POU2AF1	POU Class 2 Homeobox Associating Factor 1	6.22273
UBD	Ubiquitin D	6.22213
CD38	CD38 Molecule	6.21901
KMT2B	Lysine Methyltransferase 2B	6.21620
EIF4G1	Eukaryotic Translation Initiation Factor 4 Gamma 1	6.21403
PRSS2	Serine Protease 2	6.21117
SEC14L2	SEC14 Like Lipid Binding 2	6.20802
INHBA	Inhibin Subunit Beta A	6.20737
TSPO	Translocator Protein	6.20703
PDZD2	PDZ Domain Containing 2	6.20455
NNMT	Nicotinamide N-Methyltransferase	6.20429
CDR1-AS	CDR1 Antisense RNA	6.20171
ITGAL	Integrin Subunit Alpha L	6.20063
BLK	BLK Proto-Oncogene, Src Family Tyrosine Kinase	6.19979
PRKACB	Protein Kinase CAMP-Activated Catalytic Subunit Beta	6.19862
MPLKIP	M-Phase Specific PLK1 Interacting Protein	6.19683
PPY	Pancreatic Polypeptide	6.19507
TXNRD2	Thioredoxin Reductase 2	6.19277
AKR1A1	Aldo-Keto Reductase Family 1 Member A1	6.18277
CLEC3B	C-Type Lectin Domain Family 3 Member B	6.18217
CHAT	Choline O-Acetyltransferase	6.18148
LAMA4	Laminin Subunit Alpha 4	6.17682
FHL2	Four And A Half LIM Domains 2	6.17175
ITPR3	Inositol 1,4,5-Trisphosphate Receptor Type 3	6.17060
RSF1	Remodeling And Spacing Factor 1	6.16889
PDK1	Pyruvate Dehydrogenase Kinase 1	6.16800
SCGB2A1	Secretoglobin Family 2A Member 1	6.16677
KISS1R	KISS1 Receptor	6.16366
RPL27	Ribosomal Protein L27	6.15863
CHD7	Chromodomain Helicase DNA Binding Protein 7	6.15788
TNFSF13B	TNF Superfamily Member 13b	6.15354
CEP290	Centrosomal Protein 290	6.14850
SERPINC1	Serpin Family C Member 1	6.14528
MIR7-3	MicroRNA 7-3	6.14361
ENTR1	Endosome Associated Trafficking Regulator 1	6.13889
GGPS1	Geranylgeranyl Diphosphate Synthase 1	6.13884
MYBPC3	Myosin Binding Protein C3	6.13641

CREB3L4	CAMP Responsive Element Binding Protein 3 Like 4	6.13575
DPF2	Double PHD Fingers 2	6.13198
ANAPC5	Anaphase Promoting Complex Subunit 5	6.12828
MRAS	Muscle RAS Oncogene Homolog	6.12622
SFTPC	Surfactant Protein C	6.12258
GREB1L	GREB1 Like Retinoic Acid Receptor Coactivator	6.12162
EGLN3	Egl-9 Family Hypoxia Inducible Factor 3	6.12025
DAB2	DAB Adaptor Protein 2	6.12002
NEK8	NIMA Related Kinase 8	6.11149
PSMB8	Proteasome 20S Subunit Beta 8	6.10789
ACSL5	Acyl-CoA Synthetase Long Chain Family Member 5	6.10098
NOL8	Nucleolar Protein 8	6.09226
TRERF1	Transcriptional Regulating Factor 1	6.09000
RHNO1	RAD9-HUS1-RAD1 Interacting Nuclear Orphan 1	6.08765
CRYAA	Crystallin Alpha A	6.08762
GSDMB	Gasdermin B	6.08477
FABP3	Fatty Acid Binding Protein 3	6.08124
AAGAB	Alpha And Gamma Adaptin Binding Protein	6.07501
PTPN9	Protein Tyrosine Phosphatase Non-Receptor Type 9	6.07111
MDC1	Mediator Of DNA Damage Checkpoint 1	6.06808
NFATC1	Nuclear Factor Of Activated T Cells 1	6.06274
MIR582	MicroRNA 582	6.06272
AIRE	Autoimmune Regulator	6.06199
NDC80	NDC80 Kinetochore Complex Component	6.05940
MIEN1	Migration And Invasion Enhancer 1	6.05006
MLNR	Motilin Receptor	6.04395
CTBP1-AS	CTBP1 Antisense RNA	6.03914
MIR605	MicroRNA 605	6.03907
ASS1	Argininosuccinate Synthase 1	6.03895
SRSF1	Serine And Arginine Rich Splicing Factor 1	6.03866
NECTIN4	Nectin Cell Adhesion Molecule 4	6.03859
CTSE	Cathepsin E	6.03373
PSMD10	Proteasome 26S Subunit, Non-ATPase 10	6.03325
HDAC3	Histone Deacetylase 3	6.02313
RAD23B	RAD23 Homolog B, Nucleotide Excision Repair Protein	6.02246
HOXB2	Homeobox B2	6.01580

OSM	Oncostatin M	6.01078
PTPRU	Protein Tyrosine Phosphatase Receptor Type U	6.00770
CASP5	Caspase 5	6.00639
RPA1	Replication Protein A1	6.00430
SRPK1	SRSF Protein Kinase 1	6.00032
RANBP9	RAN Binding Protein 9	5.99842
CTCFL	CCCTC-Binding Factor Like	5.99805
EPHB1	EPH Receptor B1	5.99453
MIR422A	MicroRNA 422a	5.99155
PRKD2	Protein Kinase D2	5.98284
PTPN22	Protein Tyrosine Phosphatase Non-Receptor Type 22	5.97845
PPP5C	Protein Phosphatase 5 Catalytic Subunit	5.97075
SAFB2	Scaffold Attachment Factor B2	5.97060
NBR1	NBR1 Autophagy Cargo Receptor	5.96998
RPS28	Ribosomal Protein S28	5.96814
ITGAX	Integrin Subunit Alpha X	5.96613
DKK3	Dickkopf WNT Signaling Pathway Inhibitor 3	5.96525
EGLN1	Egl-9 Family Hypoxia Inducible Factor 1	5.96073
NPRL2	NPR2 Like, GATOR1 Complex Subunit	5.96070
CD59	CD59 Molecule (CD59 Blood Group)	5.95966
SOX30	SRY-Box Transcription Factor 30	5.95773
ANKRD11	Ankyrin Repeat Domain 11	5.95649
SERBP1	SERPINE1 MRNA Binding Protein 1	5.95553
MED14	Mediator Complex Subunit 14	5.95534
CDO1	Cysteine Dioxygenase Type 1	5.95449
CNR1	Cannabinoid Receptor 1	5.94837
MICB	MHC Class I Polypeptide-Related Sequence B	5.94684
MBP	Myelin Basic Protein	5.94490
CYBA	Cytochrome B-245 Alpha Chain	5.93747
LGALS9	Galectin 9	5.93543
CPOX	Coproporphyrinogen Oxidase	5.93279
TTC7A	Tetratricopeptide Repeat Domain 7A	5.93159
BATF2	Basic Leucine Zipper ATF-Like Transcription Factor 2	5.92051
EIF5A2	Eukaryotic Translation Initiation Factor 5A2	5.91460
FRAT1	FRAT Regulator Of WNT Signaling Pathway 1	5.91268
GSTM2	Glutathione S-Transferase Mu 2	5.90866
CCNT2	Cyclin T2	5.89773
REL	REL Proto-Oncogene, NF-KB Subunit	5.88786
ALOX15B	Arachidonate 15-Lipoxygenase Type B	5.88443
RREB1	Ras Responsive Element Binding Protein 1	5.88435

MIR363	MicroRNA 363	5.88395
MIR302A	MicroRNA 302a	5.88255
CARS1	Cysteinyl-TRNA Synthetase 1	5.88211
PDE11A	Phosphodiesterase 11A	5.88175
TCOF1	Treacle Ribosome Biogenesis Factor 1	5.87958
TPM4	Tropomyosin 4	5.87673
PSMB9	Proteasome 20S Subunit Beta 9	5.87649
DPEP1	Dipeptidase 1	5.87354
PCID2	PCI Domain Containing 2	5.87140
SNCA	Synuclein Alpha	5.86944
BRD7	Bromodomain Containing 7	5.86778
CDCA7	Cell Division Cycle Associated 7	5.86556
GSR	Glutathione-Disulfide Reductase	5.86396
LINC00958	Long Intergenic Non-Protein Coding RNA 958	5.86351
CBLB	Cbl Proto-Oncogene B	5.85781
ACTG2	Actin Gamma 2, Smooth Muscle	5.85728
QSOX1	Quiescin Sulfhydryl Oxidase 1	5.85650
BCL3	BCL3 Transcription Coactivator	5.85518
CGB3	Chorionic Gonadotropin Subunit Beta 3	5.85361
RPS26	Ribosomal Protein S26	5.85119
RPS27A	Ribosomal Protein S27a	5.84333
COL5A1	Collagen Type V Alpha 1 Chain	5.84240
CD79B	CD79b Molecule	5.84202
LCOR	Ligand Dependent Nuclear Receptor Corepressor	5.83877
TIA1	TIA1 Cytotoxic Granule Associated RNA Binding Protein	5.83564
ADIPOR2	Adiponectin Receptor 2	5.83481
ANTXR1	ANTXR Cell Adhesion Molecule 1	5.82823
HLA-E	Major Histocompatibility Complex, Class I, E	5.82784
TEAD1	TEA Domain Transcription Factor 1	5.82765
CTAGE1	Cutaneous T Cell Lymphoma-Associated Antigen 1	5.82691
BMPR1B	Bone Morphogenetic Protein Receptor Type 1B	5.82156
MAP3K20	Mitogen-Activated Protein Kinase Kinase Kinase 20	5.82044
PLA2G10	Phospholipase A2 Group X	5.82020
ITGA4	Integrin Subunit Alpha 4	5.81981
MIR608	MicroRNA 608	5.81871
LTF	Lactotransferrin	5.81670
ERLNC1	Estrogen Receptor Responsive LncRNA 1	5.81573
KLF2	Kruppel Like Factor 2	5.81562
CD7	CD7 Molecule	5.81321
SELENOP	Selenoprotein P	5.81223

TM9SF4	Transmembrane 9 Superfamily Member 4	5.80918
USP15	Ubiquitin Specific Peptidase 15	5.80717
SLC6A3	Solute Carrier Family 6 Member 3	5.80573
ADORA1	Adenosine A1 Receptor	5.79497
EFNA3	Ephrin A3	5.79098
THRB	Thyroid Hormone Receptor Beta	5.78908
CYP2A13	Cytochrome P450 Family 2 Subfamily A Member 13	5.78902
DNTT	DNA Nucleotidylexotransferase	5.78757
TJP2	Tight Junction Protein 2	5.78545
TPD52L2	TPD52 Like 2	5.77819
ADRB3	Adrenoceptor Beta 3	5.77639
SEMA4F	Ssemaphorin 4F	5.77190
LINC00857	Long Intergenic Non-Protein Coding RNA 857	5.76915
YWHAZ	Tyrosine 3-Monooxygenase/Tryptophan 5-Monooxygenase Activation Protein Zeta	5.76894
APOA1	Apolipoprotein A1	5.76868
NR4A2	Nuclear Receptor Subfamily 4 Group A Member 2	5.76471
SHOC2	SHOC2 Leucine Rich Repeat Scaffold Protein	5.75932
DNM2	Dynamin 2	5.75757
TNFRSF11B	TNF Receptor Superfamily Member 11b	5.75426
PPHLN1	Periphilin 1	5.75222
PEG10	Paternally Expressed 10	5.75131
CTBP2	C-Terminal Binding Protein 2	5.74939
PRPF8	Pre-MRNA Processing Factor 8	5.74627
BRD3	Bromodomain Containing 3	5.74615
IHH	Indian Hedgehog Signaling Molecule	5.74143
FOXC2-AS1	FOXC2 Antisense RNA 1	5.73766
HID1	HID1 Domain Containing	5.73558
NKX2-8	NK2 Homeobox 8	5.73494
PRODH	Proline Dehydrogenase 1	5.73221
BRAT1	BRCA1 Associated ATM Activator 1	5.72652
THEMIS2	Thymocyte Selection Associated Family Member 2	5.72510
ZBTB20	Zinc Finger And BTB Domain Containing 20	5.72369
FUT8	Fucosyltransferase 8	5.72327
SLC9A4	Solute Carrier Family 9 Member A4	5.72253
PENK	Proenkephalin	5.72132
NCR1	Natural Cytotoxicity Triggering Receptor 1	5.71721
CYP11A1	Cytochrome P450 Family 11 Subfamily A Member 1	5.71663

ABCC12	ATP Binding Cassette Subfamily C Member 12	5.71199
RNF14	Ring Finger Protein 14	5.71099
FAM120A	Family With Sequence Similarity 120A	5.70824
FOXA2	Forkhead Box A2	5.70576
DUXAP10	Double Homeobox A Pseudogene 10	5.70431
TRADD	TNFRSF1A Associated Via Death Domain	5.70368
FOXC2	Forkhead Box C2	5.70109
C4A	Complement C4A (Rodgers Blood Group)	5.70060
TFPI	Tissue Factor Pathway Inhibitor	5.70050
ZNF793	Zinc Finger Protein 793	5.69993
PLD1	Phospholipase D1	5.69844
MIR637	MicroRNA 637	5.69612
TERF2	Telomeric Repeat Binding Factor 2	5.69404
TGM3	Transglutaminase 3	5.69332
CACNA1G-AS	CACNA1G Antisense RNA 1	5.68656
ADA2	Adenosine Deaminase 2	5.68632
UVRAG	UV Radiation Resistance Associated	5.68226
ZFTA	Zinc Finger Translocation Associated	5.67982
TEX14	Testis Expressed 14, Intercellular Bridge Forming Factor	5.67960
CDH23	Cadherin Related 23	5.67813
PRMT5	Protein Arginine Methyltransferase 5	5.67589
GAS6-AS1	GAS6 Antisense RNA 1	5.67122
MAF	MAF BZIP Transcription Factor	5.67024
CUX1	Cut Like Homeobox 1	5.67022
ANAPC1	Anaphase Promoting Complex Subunit 1	5.66785
BACE1-AS	BACE1 Antisense RNA	5.66637
NUCB2	Nucleobindin 2	5.66471
CARD11	Caspase Recruitment Domain Family Member 11	5.66444
STN1	STN1 Subunit Of CST Complex	5.66336
DHDH	Dihydrodiol Dehydrogenase	5.66205
STARD13	StAR Related Lipid Transfer Domain Containing 13	5.65705
MIR628	MicroRNA 628	5.65543
TNFSF13	TNF Superfamily Member 13	5.65489
TMPRSS4	Transmembrane Serine Protease 4	5.65457
SCN10A	Sodium Voltage-Gated Channel Alpha Subunit 10	5.64481
USP9X	Ubiquitin Specific Peptidase 9 X-Linked	5.64423
NSD2	Nuclear Receptor Binding SET Domain Protein 2	5.64291
ATF7IP	Activating Transcription Factor 7 Interacting Protein	5.64266

LINC01589	Long Intergenic Non-Protein Coding RNA 1589	5.64213
TNS1	Tensin 1	5.63775
CSNK1E	Casein Kinase 1 Epsilon	5.63442
MIR452	MicroRNA 452	5.63027
GPA33	Glycoprotein A33	5.62796
SCGB3A1	Secretoglobin Family 3A Member 1	5.62715
ASAH1	N-Acylsphingosine Amidohydrolase 1	5.62592
ABCB4	ATP Binding Cassette Subfamily B Member 4	5.62556
PMEL	Premelanosome Protein	5.62078
CLCN6	Chloride Voltage-Gated Channel 6	5.61603
RPS15A	Ribosomal Protein S15a	5.61168
LDLR	Low Density Lipoprotein Receptor	5.60272
FAM215A	Family With Sequence Similarity 215 Member A	5.59750
TNFRSF4	TNF Receptor Superfamily Member 4	5.59454
IL23R	Interleukin 23 Receptor	5.59253
SPON2	Spondin 2	5.59250
OCIAD2	OCIA Domain Containing 2	5.59201
YES1	YES Proto-Oncogene 1, Src Family Tyrosine Kinase	5.58873
SVEP1	Sushi, Von Willebrand Factor Type A, EGF And Pentraxin Domain Containing 1	5.58869
USHBP1	USH1 Protein Network Component Harmonin Binding Protein 1	5.58633
DMTN	Dematin Actin Binding Protein	5.58449
PHLDA1	Pleckstrin Homology Like Domain Family A Member 1	5.58283
NOP16	NOP16 Nucleolar Protein	5.58245
ALAD	Aminolevulinate Dehydratase	5.58175
VOPP1	VOPP1 WW Domain Binding Protein	5.58160
PLEC	Plectin	5.57800
CYBB	Cytochrome B-245 Beta Chain	5.57776
NCL	Nucleolin	5.57749
FXYD5	FXYD Domain Containing Ion Transport Regulator 5	5.57666
SPTAN1	Spectrin Alpha, Non-Erythrocytic 1	5.57627
RPL35	Ribosomal Protein L35	5.57584
MIR92B	MicroRNA 92b	5.57134
BAZ1B	Bromodomain Adjacent To Zinc Finger Domain 1B	5.56998
FBXO5	F-Box Protein 5	5.56367
POLR2E	RNA Polymerase II, I And III Subunit E	5.56090
MIR103A2	MicroRNA 103a-2	5.55381
ALOX15	Arachidonate 15-Lipoxygenase	5.55182
CSN2	Casein Beta	5.55176
PLA2G6	Phospholipase A2 Group VI	5.55071

SOX17	SRY-Box Transcription Factor 17	5.55052
KLF9	Kruppel Like Factor 9	5.54961
CD99	CD99 Molecule (Xg Blood Group)	5.54368
PPIEL	Peptidylprolyl Isomerase E Like Pseudogene	5.54311
PTPN6	Protein Tyrosine Phosphatase Non-Receptor Type 6	5.54170
WNT8A	Wnt Family Member 8A	5.53915
TNNI3	Troponin I3, Cardiac Type	5.53249
CHFR	Checkpoint With Forkhead And Ring Finger Domains	5.53143
SRSF6	Serine And Arginine Rich Splicing Factor 6	5.52977
CXCL3	C-X-C Motif Chemokine Ligand 3	5.51902
IL17F	Interleukin 17F	5.51642
CCR2	C-C Motif Chemokine Receptor 2	5.51438
UCN	Urocortin	5.50995
STEAP4	STEAP4 Metalloreductase	5.50986
ACP5	Acid Phosphatase 5, Tartrate Resistant	5.50734
FNDC3B	Fibronectin Type III Domain Containing 3B	5.50619
MIR873	MicroRNA 873	5.50536
NMBR	Neuromedin B Receptor	5.50508
SMCHD1	Structural Maintenance Of Chromosomes Flexible Hinge Domain Containing 1	5.50050
PPP1CA	Protein Phosphatase 1 Catalytic Subunit Alpha	5.50039
SAMD9	Sterile Alpha Motif Domain Containing 9	5.49967
CRKL	CRK Like Proto-Oncogene, Adaptor Protein	5.49868
ARG1	Arginase 1	5.49801
MLXIPL	MLX Interacting Protein Like	5.49726
BRD2	Bromodomain Containing 2	5.49467
DLGAP5	DLG Associated Protein 5	5.49428
PFKFB3	6-Phosphofructo-2-Kinase/Fructose-2,6-Biphosphatase 3	5.48932
KCNK12	Potassium Two Pore Domain Channel Subfamily K Member 12	5.48361
PRPF31	Pre-MRNA Processing Factor 31	5.47950
NUTM2B	NUT Family Member 2B	5.47844
CDK9	Cyclin Dependent Kinase 9	5.47570
TUBA1A	Tubulin Alpha 1a	5.47253
NEMF	Nuclear Export Mediator Factor	5.46939
LINC00963	Long Intergenic Non-Protein Coding RNA 963	5.46781
FURIN	Furin, Paired Basic Amino Acid Cleaving Enzyme	5.46574

GJB1	Gap Junction Protein Beta 1	5.46465
TCN2	Transcobalamin 2	5.46423
TNXB	Tenascin XB	5.45833
UGT1A9	UDP Glucuronosyltransferase Family 1 Member A9	5.45592
PPP1R13L	Protein Phosphatase 1 Regulatory Subunit 13 Like	5.45586
IL16	Interleukin 16	5.45126
SMC1A	Structural Maintenance Of Chromosomes 1A	5.45115
ADAMTS13	ADAM Metallopeptidase With Thrombospondin Type 1 Motif 13	5.45022
VEGFB	Vascular Endothelial Growth Factor B	5.44723
GATA5	GATA Binding Protein 5	5.44692
ADGRB1	Adhesion G Protein-Coupled Receptor B1	5.44406
NCF4	Neutrophil Cytosolic Factor 4	5.44323
RBP4	Retinol Binding Protein 4	5.43986
MB	Myoglobin	5.43936
NUAK1	NUAK Family Kinase 1	5.43882
MED19	Mediator Complex Subunit 19	5.43881
PSAT1	Phosphoserine Aminotransferase 1	5.43850
CRK	CRK Proto-Oncogene, Adaptor Protein	5.43766
HEIH	Hepatocellular Carcinoma Up-Regulated EZH2-Associated Long Non-Coding RNA	5.43763
SMARCD1	SWI/SNF Related, Matrix Associated, Actin Dependent Regulator Of Chromatin, Subfamily D, Member 1	5.43550
PSMB7	Proteasome 20S Subunit Beta 7	5.43472
BTC	Betacellulin	5.43040
ADAMTS9-AS	ADAMTS9 Antisense RNA 2	5.42823
SCN5A	Sodium Voltage-Gated Channel Alpha Subunit 5	5.42779
LINC00092	Long Intergenic Non-Protein Coding RNA 92	5.42724
COL6A3	Collagen Type VI Alpha 3 Chain	5.42687
AGAP2	ArfGAP With GTPase Domain, Ankyrin Repeat And PH Domain 2	5.42435
NT5E	5'-Nucleotidase Ecto	5.42399
FBXO11	F-Box Protein 11	5.41577
ADAM10	ADAM Metallopeptidase Domain 10	5.41491
LINC00365	Long Intergenic Non-Protein Coding RNA 365	5.41335
ABCC4	ATP Binding Cassette Subfamily C Member 4	5.41251
SLC25A11	Solute Carrier Family 25 Member 11	5.40996
LAPTM4B	Lysosomal Protein Transmembrane 4 Beta	5.40554

POLE2	DNA Polymerase Epsilon 2, Accessory Subunit	5.40007
SSTR3	Somatostatin Receptor 3	5.39512
AMBP	Alpha-1-Microglobulin/Bikunin Precursor	5.38876
LPXN	Leupaxin	5.38834
ZNF276	Zinc Finger Protein 276	5.38760
GLS	Glutaminase	5.38502
METTL3	Methyltransferase Like 3	5.38293
ACTL6A	Actin Like 6A	5.37874
PARK7	Parkinsonism Associated Deglycase	5.37749
ADIPOR1	Adiponectin Receptor 1	5.37591
ASPM	Assembly Factor For Spindle Microtubules	5.36922
VCP	Valosin Containing Protein	5.36757
CAMTA1	Calmodulin Binding Transcription Activator 1	5.36652
SART1	Spliceosome Associated Factor 1, Recruiter Of U4/U6.U5 Tri-SnRNP	5.36413
ITGAE	Integrin Subunit Alpha E	5.36131
TFG	Trafficking From ER To Golgi Regulator	5.35571
H3-3B	H3.3 Histone B	5.35449
NWD1	NACHT And WD Repeat Domain Containing 1	5.35133
FGF5	Fibroblast Growth Factor 5	5.34943
ERGIC3	ERGIC And Golgi 3	5.34931
ARTN	Artemin	5.34670
AQP4	Aquaporin 4	5.34406
DUOX2	Dual Oxidase 2	5.34022
MAGEA2	MAGE Family Member A2	5.33688
GCGR	Glucagon Receptor	5.33348
RFC3	Replication Factor C Subunit 3	5.32992
GIPC1	GIPC PDZ Domain Containing Family Member 1	5.32952
CBY1	Chibby Family Member 1, Beta Catenin Antagonist	5.32931
NAGLU	N-Acetyl-Alpha-Glucosaminidase	5.32708
AQP3	Aquaporin 3 (Gill Blood Group)	5.32464
KRT16	Keratin 16	5.32229
POU5F1P4	POU Class 5 Homeobox 1 Pseudogene 4	5.32179
ZNF148	Zinc Finger Protein 148	5.32149
ASNS	Asparagine Synthetase (Glutamine-Hydrolyzing)	5.32141
TNIP1	TNFAIP3 Interacting Protein 1	5.31943
PDCD4-AS1	PDCD4 Antisense RNA 1	5.31861
CD209	CD209 Molecule	5.31644
MIB2	MIB E3 Ubiquitin Protein Ligase 2	5.31482

LINC00473	Long Intergenic Non-Protein Coding RNA 473	5.31221
CD1A	CD1a Molecule	5.30829
OGFR	Opioid Growth Factor Receptor	5.30810
IRF8	Interferon Regulatory Factor 8	5.30558
CREB3	CAMP Responsive Element Binding Protein 3	5.30511
SSTR5	Somatostatin Receptor 5	5.30477
RPS6KB2	Ribosomal Protein S6 Kinase B2	5.29567
NMRAL2P	NmrA Like Redox Sensor 2, Pseudogene	5.29563
ZNF300P1	Zinc Finger Protein 300 Pseudogene 1	5.29544
LITAF	Lipopolysaccharide Induced TNF Factor	5.29464
SERPINF1	Serpin Family F Member 1	5.28867
MAPT	Microtubule Associated Protein Tau	5.28829
PRKAB1	Protein Kinase AMP-Activated Non-Catalytic Subunit Beta 1	5.28770
SKIV2L	Ski2 Like RNA Helicase	5.28728
ADGRE5	Adhesion G Protein-Coupled Receptor E5	5.28286
MAP1LC3A	Microtubule Associated Protein 1 Light Chain 3 Alpha	5.28240
CXCL14	C-X-C Motif Chemokine Ligand 14	5.28083
MAP3K5	Mitogen-Activated Protein Kinase Kinase Kinase 5	5.28002
CXCL11	C-X-C Motif Chemokine Ligand 11	5.27872
SLC4A1	Solute Carrier Family 4 Member 1 (Diego Blood Group)	5.27621
HSPA14	Heat Shock Protein Family A (Hsp70) Member 14	5.27588
CIZ1	CDKN1A Interacting Zinc Finger Protein 1	5.26907
AVP	Arginine Vasopressin	5.26752
NOP2	NOP2 Nucleolar Protein	5.26703
CEP55	Centrosomal Protein 55	5.26303
GPSM2	G Protein Signaling Modulator 2	5.26259
TULP1	TUB Like Protein 1	5.26119
RALGAPA1	Ral GTPase Activating Protein Catalytic Subunit Alpha 1	5.26054
MIR7-2	MicroRNA 7-2	5.26013
IGF2BP1	Insulin Like Growth Factor 2 MRNA Binding Protein 1	5.25994
FTX	FTX Transcript, XIST Regulator	5.25949
OXT	Oxytocin/Neurophysin I Prepropeptide	5.25930
B4GALNT3	Beta-1,4-N-Acetyl-Galactosaminyltransferase 3	5.25711
DNAJB1	DnaJ Heat Shock Protein Family (Hsp40) Member B1	5.25489

REG3A	Regenerating Family Member 3 Alpha	5.25224
ACVRL1	Activin A Receptor Like Type 1	5.25185
CGB5	Chorionic Gonadotropin Subunit Beta 5	5.24951
TYRP1	Tyrosinase Related Protein 1	5.24761
RBM39	RNA Binding Motif Protein 39	5.24631
GAS6	Growth Arrest Specific 6	5.24598
HTR3A	5-Hydroxytryptamine Receptor 3A	5.24401
KRT15	Keratin 15	5.24193
KCNH1	Potassium Voltage-Gated Channel Subfamily H Member 1	5.24141
DLST	Dihydrolipoamide S-Succinyltransferase	5.23965
DLEU2	Deleted In Lymphocytic Leukemia 2	5.23627
NODAL	Nodal Growth Differentiation Factor	5.23367
SPN	Sialophorin	5.22933
ANXA11	Annexin A11	5.22896
MIR454	MicroRNA 454	5.22773
ANXA6	Annexin A6	5.21808
STK33	Serine/Threonine Kinase 33	5.21797
MX1	MX Dynamin Like GTPase 1	5.21763
CLCA1	Chloride Channel Accessory 1	5.21741
MUS81	MUS81 Structure-Specific Endonuclease Subunit	5.21588
ARHGAP5	Rho GTPase Activating Protein 5	5.21143
GOLPH3	Golgi Phosphoprotein 3	5.20407
C3	Complement C3	5.20407
CFH	Complement Factor H	5.20341
LINC00339	Long Intergenic Non-Protein Coding RNA 339	5.20271
TCN1	Transcobalamin 1	5.20117
MYH8	Myosin Heavy Chain 8	5.19992
AVPR2	Arginine Vasopressin Receptor 2	5.18958
H3C1	H3 Clustered Histone 1	5.18915
TCIRG1	T Cell Immune Regulator 1, ATPase H+ Transporting V0 Subunit A3	5.18611
ATXN2	Ataxin 2	5.18135
CLDN6	Claudin 6	5.17923
TMCO1	Transmembrane And Coiled-Coil Domains 1	5.17571
RAG1	Recombination Activating 1	5.17442
LAMA3	Laminin Subunit Alpha 3	5.17391
TBK1	TANK Binding Kinase 1	5.17208
UGT1A4	UDP Glucuronosyltransferase Family 1 Member A4	5.17136
PTGER3	Prostaglandin E Receptor 3	5.16934
LMOD1	Leiomodin 1	5.16851
HPS3	HPS3 Biogenesis Of Lysosomal Organelles Complex 2 Subunit 1	5.16728
OCLN	Occludin	5.16499

HLA-DPB1	Major Histocompatibility Complex, Class II, DP Beta 1	5.16253
UBR5	Ubiquitin Protein Ligase E3 Component N-Recognin 5	5.16015
MC4R	Melanocortin 4 Receptor	5.15783
SMURF1	SMAD Specific E3 Ubiquitin Protein Ligase 1	5.15141
NEU1	Neuraminidase 1	5.14764
DCST1-AS1	DCST1 Antisense RNA 1	5.14738
ALKBH1	AlkB Homolog 1, Histone H2A Dioxygenase	5.14680
ELP4	Elongator Acetyltransferase Complex Subunit 4	5.14665
RPS6KA5	Ribosomal Protein S6 Kinase A5	5.14443
MIR302C	MicroRNA 302c	5.14200
COL17A1	Collagen Type XVII Alpha 1 Chain	5.14013
CXCL9	C-X-C Motif Chemokine Ligand 9	5.13956
FCGR2B	Fc Fragment Of IgG Receptor IIb	5.13813
GLE1	GLE1 RNA Export Mediator	5.13724
RUSC1-AS1	RUSC1 Antisense RNA 1	5.13699
SREBF2	Sterol Regulatory Element Binding Transcription Factor 2	5.12622
RPS13	Ribosomal Protein S13	5.12550
MIR513A1	MicroRNA 513a-1	5.12206
DHX37	DEAH-Box Helicase 37	5.11941
KMT5B	Lysine Methyltransferase 5B	5.11759
GHRHR	Growth Hormone Releasing Hormone Receptor	5.11560
SMPD1	Sphingomyelin Phosphodiesterase 1	5.11220
CKB	Creatine Kinase B	5.10875
RRM2B	Ribonucleotide Reductase Regulatory TP53 Inducible Subunit M2B	5.10564
IL33	Interleukin 33	5.10469
ZNF461	Zinc Finger Protein 461	5.10407
LOC283335	Uncharacterized LOC283335	5.10336
CFLAR-AS1	CFLAR Antisense RNA 1	5.10211
MIR508	MicroRNA 508	5.10031
CSTB	Cystatin B	5.09923
ATP1B1	ATPase Na+/K+ Transporting Subunit Beta 1	5.09784
CD163	CD163 Molecule	5.09749
PRKAR1B	Protein Kinase CAMP-Dependent Type I Regulatory Subunit Beta	5.09706
SLC31A1	Solute Carrier Family 31 Member 1	5.09438
MMP26	Matrix Metallopeptidase 26	5.08168
MIR515-1	MicroRNA 515-1	5.07725
HOXB8	Homeobox B8	5.07723
MYHAS	Myosin Heavy Chain Gene Cluster Antisense RNA	5.07496

E2F7	E2F Transcription Factor 7	5.07426
ACKR3	Atypical Chemokine Receptor 3	5.07151
AQP1	Aquaporin 1 (Colton Blood Group)	5.06767
APRT	Adenine Phosphoribosyltransferase	5.06541
XPO5	Exportin 5	5.06468
FOXP4	Forkhead Box P4	5.06464
CA12	Carbonic Anhydrase 12	5.06453
NOP14	NOP14 Nucleolar Protein	5.06193
VCAN	Versican	5.06154
KLF3-AS1	KLF3 Antisense RNA 1	5.06126
MIR9-2	MicroRNA 9-2	5.06065
ENPP7	Ectonucleotide Pyrophosphatase/Phosphodiesterase 7	5.06057
TPMT	Thiopurine S-Methyltransferase	5.05680
FOXCUT	FOXC1 Upstream Transcript	5.05124
REN	Renin	5.04756
SLC26A7	Solute Carrier Family 26 Member 7	5.04570
MANCR	Mitotically Associated Long Non Coding RNA	5.04385
GUCY1B2	Guanylate Cyclase 1 Soluble Subunit Beta 2 (Pseudogene)	5.04287
MC2R	Melanocortin 2 Receptor	5.04185
GATD3A	Glutamine Amidotransferase Like Class 1 Domain Containing 3A	5.04113
SNHG3	Small Nucleolar RNA Host Gene 3	5.03958
LRBA	LPS Responsive Beige-Like Anchor Protein	5.03363
YTHDF1	YTH N6-Methyladenosine RNA Binding Protein 1	5.03215
RICTOR	RPTOR Independent Companion Of MTOR Complex 2	5.03146
UBR4	Ubiquitin Protein Ligase E3 Component N-Recognin 4	5.03137
MIR1304	MicroRNA 1304	5.02714
HSPG2	Heparan Sulfate Proteoglycan 2	5.02698
BCL2L2	BCL2 Like 2	5.02420
TNFSF15	TNF Superfamily Member 15	5.02280
MIR511	MicroRNA 511	5.01954
NUP98	Nucleoporin 98 And 96 Precursor	5.01911
XAF1	XIAP Associated Factor 1	5.01887
HHIP	Hedgehog Interacting Protein	5.01840
ZDHHC2	Zinc Finger DHHC-Type Palmitoyltransferase 2	5.01758
PANK2	Pantothenate Kinase 2	5.01140
TERF2IP	TERF2 Interacting Protein	4.99638
WDR26	WD Repeat Domain 26	4.99606
SMYD3	SET And MYND Domain Containing 3	4.99524
B3GALT5	Beta-1,3-Galactosyltransferase 5	4.99494
F10	Coagulation Factor X	4.99373

MIR744	MicroRNA 744	4.99362
CBS	Cystathionine Beta-Synthase	4.99340
PBX1	PBX Homeobox 1	4.99293
TET1	Tet Methylcytosine Dioxygenase 1	4.98965
LIMS1	LIM Zinc Finger Domain Containing 1	4.98335
CIITA	Class II Major Histocompatibility Complex Transactivator	4.98179
F13A1	Coagulation Factor XIII A Chain	4.98073
HTATIP2	HIV-1 Tat Interactive Protein 2	4.98031
ADCY10	Adenylate Cyclase 10	4.97724
SATB1	SATB Homeobox 1	4.97698
NR2F2	Nuclear Receptor Subfamily 2 Group F Member 2	4.97510
CCDC88C	Coiled-Coil Domain Containing 88C	4.97509
MIR340	MicroRNA 340	4.97192
DEFB1	Defensin Beta 1	4.97045
DPP9	Dipeptidyl Peptidase 9	4.96887
CNTNAP1	Contactin Associated Protein 1	4.96134
MIR99B	MicroRNA 99b	4.95735
JUNB	JunB Proto-Oncogene, AP-1 Transcription Factor Subunit	4.95195
C1S	Complement C1s	4.95145
IFNAR2	Interferon Alpha And Beta Receptor Subunit 2	4.94719
FAH	Fumarylacetoacetate Hydrolase	4.94583
DFFA	DNA Fragmentation Factor Subunit Alpha	4.94554
NKX2-5	NK2 Homeobox 5	4.94372
HTRA1	HtrA Serine Peptidase 1	4.94304
PBK	PDZ Binding Kinase	4.93808
GPR35	G Protein-Coupled Receptor 35	4.93798
HNRNPL	Heterogeneous Nuclear Ribonucleoprotein L	4.93590
HOTAIRM1	HOXA Transcript Antisense RNA, Myeloid-Specific 1	4.93162
CPT2	Carnitine Palmitoyltransferase 2	4.92927
MMP8	Matrix Metallopeptidase 8	4.92915
RAB11B	RAB11B, Member RAS Oncogene Family	4.92856
NEK2	NIMA Related Kinase 2	4.92820
SLC16A1	Solute Carrier Family 16 Member 1	4.92686
MIR124-2	MicroRNA 124-2	4.92645
MIR622	MicroRNA 622	4.92079
RFC2	Replication Factor C Subunit 2	4.91839
HMOX2	Heme Oxygenase 2	4.91747
SPG7	SPG7 Matrix AAA Peptidase Subunit, Paraplegin	4.91285
ATP2B3	ATPase Plasma Membrane Ca2+ Transporting 3	4.91214

MT2A	Metallothionein 2A	4.91114
SAGE1	Sarcoma Antigen 1	4.90898
CD70	CD70 Molecule	4.90757
RECQL5	RecQ Like Helicase 5	4.90750
LAMA2	Laminin Subunit Alpha 2	4.90652
LINC00598	Long Intergenic Non-Protein Coding RNA 598	4.90639
NFIC	Nuclear Factor I C	4.90246
MMP15	Matrix Metallopeptidase 15	4.89579
BCHE	Butyrylcholinesterase	4.89485
CR1	Complement C3b/C4b Receptor 1 (Knops Blood Group)	4.88956
TUBA4B	Tubulin Alpha 4b	4.88791
APP	Amyloid Beta Precursor Protein	4.88557
TRA	T Cell Receptor Alpha Locus	4.88446
SLCO1B1	Solute Carrier Organic Anion Transporter Family Member 1B1	4.88190
SLC2A2	Solute Carrier Family 2 Member 2	4.88119
DNM3OS	DNM3 Opposite Strand/Antisense RNA	4.87924
LARP1	La Ribonucleoprotein 1, Translational Regulator	4.87731
TPM1	Tropomyosin 1	4.87395
MADD	MAP Kinase Activating Death Domain	4.87297
PES1	Pescadillo Ribosomal Biogenesis Factor 1	4.87251
RANBP2	RAN Binding Protein 2	4.87243
DDX19A-DT	DDX19A Divergent Transcript	4.87219
SARS1	Seryl-TRNA Synthetase 1	4.87030
NOD1	Nucleotide Binding Oligomerization Domain Containing 1	4.86969
MNT	MAX Network Transcriptional Repressor	4.86968
CHD4	Chromodomain Helicase DNA Binding Protein 4	4.86961
CACNA1H	Calcium Voltage-Gated Channel Subunit Alpha1 H	4.86894
PHLDB1	Pleckstrin Homology Like Domain Family B Member 1	4.86536
BTG2	BTG Anti-Proliferation Factor 2	4.86420
CLIP2	CAP-Gly Domain Containing Linker Protein 2	4.85817
KCNE2	Potassium Voltage-Gated Channel Subfamily E Regulatory Subunit 2	4.85671
FNDC5	Fibronectin Type III Domain Containing 5	4.85597
NUP107	Nucleoporin 107	4.85543
SERPINH1	Serpin Family H Member 1	4.85017
FOXP2	Forkhead Box P2	4.84943
GUCA2B	Guanylate Cyclase Activator 2B	4.84714

PLPP4	Phospholipid Phosphatase 4	4.84408
MUC13	Mucin 13, Cell Surface Associated	4.84376
KIR3DL1	Killer Cell Immunoglobulin Like Receptor, Three Ig Domains And Long Cytoplasmic Tail 1	4.84078
PSEN1	Presenilin 1	4.83893
NEIL2	Nei Like DNA Glycosylase 2	4.83871
COL3A1	Collagen Type III Alpha 1 Chain	4.83825
PEX6	Peroxisomal Biogenesis Factor 6	4.83612
PROCR	Protein C Receptor	4.83454
GAS1	Growth Arrest Specific 1	4.83231
MALINC1	Mitosis Associated Long Intergenic Non-Coding RNA 1	4.82951
KCNE1	Potassium Voltage-Gated Channel Subfamily E Regulatory Subunit 1	4.82781
ASCL2	Achaete-Scute Family BHLH Transcription Factor 2	4.82718
MIR409	MicroRNA 409	4.82636
HAMP	Hepcidin Antimicrobial Peptide	4.82478
NEDD4L	NEDD4 Like E3 Ubiquitin Protein Ligase	4.82417
FLT3LG	Fms Related Receptor Tyrosine Kinase 3 Ligand	4.82060
STOM	Stomatin	4.82049
LYL1	LYL1 Basic Helix-Loop-Helix Family Member	4.81880
TOB1	Transducer Of ERBB2, 1	4.81871
FOXG1	Forkhead Box G1	4.81758
GAL	Galanin And GMAP Prepropeptide	4.81676
GRIN2A	Glutamate Ionotropic Receptor NMDA Type Subunit 2A	4.81630
KNG1	Kininogen 1	4.81613
VSIG1	V-Set And Immunoglobulin Domain Containing 1	4.81573
TACR1	Tachykinin Receptor 1	4.81517
RNASEH2B	Ribonuclease H2 Subunit B	4.81279
MIR627	MicroRNA 627	4.81182
ASPH	Aspartate Beta-Hydroxylase	4.81171
CYBC1	Cytochrome B-245 Chaperone 1	4.80842
CARD8-AS1	CARD8 Antisense RNA 1	4.80575
LDHB	Lactate Dehydrogenase B	4.80182
IL1RL1	Interleukin 1 Receptor Like 1	4.79969
H1-3	H1.3 Linker Histone, Cluster Member	4.79858
LACTB	Lactamase Beta	4.79814
CCR3	C-C Motif Chemokine Receptor 3	4.79728
MLKL	Mixed Lineage Kinase Domain Like Pseudokinase	4.79663
ATP2A3	ATPase Sarcoplasmic/Endoplasmic Reticulum Ca2+ Transporting 3	4.79654

HTT	Huntingtin	4.79244
UVSSA	UV Stimulated Scaffold Protein A	4.79079
UFC1	Ubiquitin-Fold Modifier Conjugating Enzyme 1	4.79001
LINC01671	Long Intergenic Non-Protein Coding RNA 1671	4.78950
MAMDC2-AS1	MAMDC2 Antisense RNA 1	4.78908
MAP1LC3B	Microtubule Associated Protein 1 Light Chain 3 Beta	4.78745
DNASE1	Deoxyribonuclease 1	4.78703
CD1D	CD1d Molecule	4.78084
ZFYVE26	Zinc Finger FYVE-Type Containing 26	4.77924
RPS7	Ribosomal Protein S7	4.77796
SMC3	Structural Maintenance Of Chromosomes 3	4.77369
CCL4	C-C Motif Chemokine Ligand 4	4.77299
PFKFB4	6-Phosphofructo-2-Kinase/Fructose-2,6-Biphosphatase 4	4.77290
KDM3A	Lysine Demethylase 3A	4.77276
FUCA1	Alpha-L-Fucosidase 1	4.77269
LINC01089	Long Intergenic Non-Protein Coding RNA 1089	4.77268
OPRM1	Opioid Receptor Mu 1	4.77191
TSC22D1	TSC22 Domain Family Member 1	4.77187
CD2	CD2 Molecule	4.77051
PTMA	Prothymosin Alpha	4.76441
CEL	Carboxyl Ester Lipase	4.76213
YY1AP1	YY1 Associated Protein 1	4.75574
ARFGAP3	ADP Ribosylation Factor GTPase Activating Protein 3	4.75521
MIR19B1	MicroRNA 19b-1	4.75495
SLC2A4	Solute Carrier Family 2 Member 4	4.75342
CCL22	C-C Motif Chemokine Ligand 22	4.75079
NDRG2	NDRG Family Member 2	4.74634
DUSP5	Dual Specificity Phosphatase 5	4.74473
ALX4	ALX Homeobox 4	4.74432
STARD10	StAR Related Lipid Transfer Domain Containing 10	4.74289
THBS3	Thrombospondin 3	4.74254
P4HB	Prolyl 4-Hydroxylase Subunit Beta	4.74006
CKS2	CDC28 Protein Kinase Regulatory Subunit 2	4.73948
LRP4	LDL Receptor Related Protein 4	4.73728
STAG2	Stromal Antigen 2	4.73724
ATG16L1	Autophagy Related 16 Like 1	4.73578
ITPR1	Inositol 1,4,5-Trisphosphate Receptor Type 1	4.73548
PRDX5	Peroxiredoxin 5	4.73506
ITGB2	Integrin Subunit Beta 2	4.73424

TIGAR	TP53 Induced Glycolysis Regulatory Phosphatase	4.73372
CA4	Carbonic Anhydrase 4	4.73371
NR4A3	Nuclear Receptor Subfamily 4 Group A Member 3	4.73295
ANKRD26	Ankyrin Repeat Domain 26	4.73092
CFI	Complement Factor I	4.72790
BLOC1S3	Biogenesis Of Lysosomal Organelles Complex 1 Subunit 3	4.72788
PITX1	Paired Like Homeodomain 1	4.72466
ENSG00000255224		4.72458
ST8SIA2	ST8 Alpha-N-Acetyl-Neuraminide Alpha-2,8-Sialyltransferase 2	4.72321
EEF2	Eukaryotic Translation Elongation Factor 2	4.72285
MARK4	Microtubule Affinity Regulating Kinase 4	4.72141
IL21	Interleukin 21	4.72137
PLIN2	Perilipin 2	4.72118
CARD10	Caspase Recruitment Domain Family Member 10	4.72094
RAI1	Retinoic Acid Induced 1	4.72085
IRAK1	Interleukin 1 Receptor Associated Kinase 1	4.71782
C20orf204	Chromosome 20 Open Reading Frame 204	4.71575
HCRT	Hypocretin Neuropeptide Precursor	4.71485
ADAMTS1	ADAM Metallopeptidase With Thrombospondin Type 1 Motif 1	4.71432
SKP1	S-Phase Kinase Associated Protein 1	4.71346
CCR4	C-C Motif Chemokine Receptor 4	4.71331
MSN	Moesin	4.70940
TFR2	Transferrin Receptor 2	4.70917
SLC17A5	Solute Carrier Family 17 Member 5	4.70912
SPRR3	Small Proline Rich Protein 3	4.70715
MIR374A	MicroRNA 374a	4.70614
SPI1	Spi-1 Proto-Oncogene	4.70583
MMP16	Matrix Metallopeptidase 16	4.70417
SLC11A1	Solute Carrier Family 11 Member 1	4.70397
CYP11B2	Cytochrome P450 Family 11 Subfamily B Member 2	4.70248
IRX1	Iroquois Homeobox 1	4.69882
CREB3L1	CAMP Responsive Element Binding Protein 3 Like 1	4.69873
DOCK8	Dedicator Of Cytokinesis 8	4.69848
IL22	Interleukin 22	4.69333
GCM2	Glial Cells Missing Transcription Factor 2	4.69006
IL15RA	Interleukin 15 Receptor Subunit Alpha	4.68185

TCF12	Transcription Factor 12	4.67988
PEMT	Phosphatidylethanolamine N-Methyltransferase	4.67985
SOX6	SRY-Box Transcription Factor 6	4.67978
KCNJ5	Potassium Inwardly Rectifying Channel Subfamily J Member 5	4.67977
POLG2	DNA Polymerase Gamma 2, Accessory Subunit	4.67863
FAT1	FAT Atypical Cadherin 1	4.67721
TBL2	Transducin Beta Like 2	4.67642
PRPF4	Pre-MRNA Processing Factor 4	4.67478
HJV	Hemojuvelin BMP Co-Receptor	4.67375
ANXA10	Annexin A10	4.67193
CCL17	C-C Motif Chemokine Ligand 17	4.67184
CYP2C8	Cytochrome P450 Family 2 Subfamily C Member 8	4.66950
NRTN	Neurturin	4.66754
PRKAG2	Protein Kinase AMP-Activated Non-Catalytic Subunit Gamma 2	4.66614
PATZ1	POZ/BTB And AT Hook Containing Zinc Finger 1	4.66529
SLC2A3	Solute Carrier Family 2 Member 3	4.66410
CRTC1	CREB Regulated Transcription Coactivator 1	4.65805
FGL1	Fibrinogen Like 1	4.65765
SMARCAL1	SWI/SNF Related, Matrix Associated, Actin Dependent Regulator Of Chromatin, Subfamily A Like 1	4.65549
IL23A	Interleukin 23 Subunit Alpha	4.65539
HCG11	HLA Complex Group 11	4.65372
CELF1	CUGBP Elav-Like Family Member 1	4.65364
C5	Complement C5	4.65239
PYCARD	PYD And CARD Domain Containing	4.65125
SNHG17	Small Nucleolar RNA Host Gene 17	4.64961
FOXO4	Forkhead Box O4	4.64584
ENPP2	Ectonucleotide Pyrophosphatase/Phosphodiesterase 2	4.64558
IL18R1	Interleukin 18 Receptor 1	4.64537
WASF2	WASP Family Member 2	4.64389
ADD1	Adducin 1	4.64322
SLC22A2	Solute Carrier Family 22 Member 2	4.64173
ENSG00000229282		4.64039
EPHA4	EPH Receptor A4	4.63054
ATRIP-TREX1	ATRIP-TREX1 Readthrough	4.62970
PHF21A	PHD Finger Protein 21A	4.62926
MAP2	Microtubule Associated Protein 2	4.62919
MGLL	Monoglyceride Lipase	4.62719
NTSR1	Neurotensin Receptor 1	4.62627
DEFA5	Defensin Alpha 5	4.62605

AOC4P	Amine Oxidase Copper Containing 4, Pseudogene	4.62423
RASSF1-AS1	RASSF1 Antisense RNA 1	4.62354
RPS6	Ribosomal Protein S6	4.62227
CSNK2B	Casein Kinase 2 Beta	4.62199
EVI2A	Ecotropic Viral Integration Site 2A	4.62074
LOXL4	Lysyl Oxidase Like 4	4.61912
CGB7	Chorionic Gonadotropin Subunit Beta 7	4.61888
TREM1	Triggering Receptor Expressed On Myeloid Cells 1	4.61632
GUCA2A	Guanylate Cyclase Activator 2A	4.61466
SRF	Serum Response Factor	4.61460
F7	Coagulation Factor VII	4.61213
MRPS23	Mitochondrial Ribosomal Protein S23	4.60797
IRF2	Interferon Regulatory Factor 2	4.60460
FGF21	Fibroblast Growth Factor 21	4.59810
CDKN2D	Cyclin Dependent Kinase Inhibitor 2D	4.59308
BST2	Bone Marrow Stromal Cell Antigen 2	4.58997
ZNF154	Zinc Finger Protein 154	4.58008
MUC17	Mucin 17, Cell Surface Associated	4.57969
SERPINB6	Serpin Family B Member 6	4.57888
GLP1R	Glucagon Like Peptide 1 Receptor	4.57851
RAPSN	Receptor Associated Protein Of The Synapse	4.57655
FOXQ1	Forkhead Box Q1	4.57639
BHLHE40	Basic Helix-Loop-Helix Family Member E40	4.57090
EYA1	EYA Transcriptional Coactivator And Phosphatase 1	4.56848
LAMC1-AS1	LAMC1 Antisense RNA 1	4.56797
SDCBP	Syndecan Binding Protein	4.56524
PNMT	Phenylethanolamine N-Methyltransferase	4.56342
WDR11	WD Repeat Domain 11	4.56170
ID2	Inhibitor Of DNA Binding 2	4.56166
IL3RA	Interleukin 3 Receptor Subunit Alpha	4.55952
HBA1	Hemoglobin Subunit Alpha 1	4.55762
SEMA3B-AS1	SEMA3B Antisense RNA 1 (Head To Head)	4.55662
TSHZ1	Teashirt Zinc Finger Homeobox 1	4.55555
PER3	Period Circadian Regulator 3	4.55533
APOL1	Apolipoprotein L1	4.55312
RHOD	Ras Homolog Family Member D	4.55044
PGK1	Phosphoglycerate Kinase 1	4.55000
SFTPA1	Surfactant Protein A1	4.54883
SEC14L1	SEC14 Like Lipid Binding 1	4.54833
CYP21A2	Cytochrome P450 Family 21 Subfamily A Member 2	4.54820
DRD3	Dopamine Receptor D3	4.54639

RFC4	Replication Factor C Subunit 4	4.54540
KAT6B	Lysine Acetyltransferase 6B	4.54445
EPHB3	EPH Receptor B3	4.54124
SMARCC1	SWI/SNF Related, Matrix Associated, Actin Dependent Regulator Of Chromatin Subfamily C Member 1	4.54110
CIC	Capicua Transcriptional Repressor	4.54001
BDKRB1	Bradykinin Receptor B1	4.53613
APTX	Aprataxin	4.53591
LIPA	Lipase A, Lysosomal Acid Type	4.53590
DDB1	Damage Specific DNA Binding Protein 1	4.53449
PRKCH	Protein Kinase C Eta	4.53367
RPS16	Ribosomal Protein S16	4.53347
TXNIP	Thioredoxin Interacting Protein	4.53194
EGLN2	Egl-9 Family Hypoxia Inducible Factor 2	4.53170
LRIG1	Leucine Rich Repeats And Immunoglobulin Like Domains 1	4.53029
ATXN3	Ataxin 3	4.53007
SAMHD1	SAM And HD Domain Containing Deoxynucleoside Triphosphate Triphosphohydrolase 1	4.52944
EIF4A3	Eukaryotic Translation Initiation Factor 4A3	4.52868
MIR100HG	Mir-100-Let-7a-2-Mir-125b-1 Cluster Host Gene	4.52745
CDK8	Cyclin Dependent Kinase 8	4.52669
TNFSF4	TNF Superfamily Member 4	4.51930
VTCN1	V-Set Domain Containing T Cell Activation Inhibitor 1	4.51901
IFIH1	Interferon Induced With Helicase C Domain 1	4.51699
LAG3	Lymphocyte Activating 3	4.51695
NIPBL	NIPBL Cohesin Loading Factor	4.51220
CUL2	Cullin 2	4.50974
FAM168A	Family With Sequence Similarity 168 Member A	4.50887
CHD8	Chromodomain Helicase DNA Binding Protein 8	4.50881
NCF2	Neutrophil Cytosolic Factor 2	4.50751
PCBP1	Poly(RC) Binding Protein 1	4.50301
DIRAS3	DIRAS Family GTPase 3	4.50199
TSHZ2	Teashirt Zinc Finger Homeobox 2	4.50025
WNK1	WNK Lysine Deficient Protein Kinase 1	4.49723
PORCN	Porcupine O-Acyltransferase	4.49529
CARS2	Cysteinyl-TRNA Synthetase 2, Mitochondrial	4.49426
MAGEA6	MAGE Family Member A6	4.49048

CD151	CD151 Molecule (Raph Blood Group)	4.48832
TNFRSF19	TNF Receptor Superfamily Member 19	4.48455
TLR6	Toll Like Receptor 6	4.48445
CES2	Carboxylesterase 2	4.48410
PCGF2	Polycomb Group Ring Finger 2	4.48054
FTO	FTO Alpha-Ketoglutarate Dependent Dioxygenase	4.47989
ANK1	Ankyrin 1	4.47789
COL4A6	Collagen Type IV Alpha 6 Chain	4.47632
RAP1GAP	RAP1 GTPase Activating Protein	4.47228
SLC34A1	Solute Carrier Family 34 Member 1	4.46842
HORMAD1	HORMA Domain Containing 1	4.46800
FBXL19-AS1	FBXL19 Antisense RNA 1	4.46482
MIR1-1	MicroRNA 1-1	4.46352
ARNTL	Aryl Hydrocarbon Receptor Nuclear Translocator Like	4.46248
INPP5E	Inositol Polyphosphate-5-Phosphatase E	4.46238
CHD1L	Chromodomain Helicase DNA Binding Protein 1 Like	4.46112
CTNNA3	Catenin Alpha 3	4.46102
FMR1	FMRP Translational Regulator 1	4.46084
CHD5	Chromodomain Helicase DNA Binding Protein 5	4.45961
UGCG	UDP-Glucose Ceramide Glucosyltransferase	4.45632
PPP6C	Protein Phosphatase 6 Catalytic Subunit	4.45335
FOXL2	Forkhead Box L2	4.45302
SOCS2-AS1	SOCS2 Antisense RNA 1	4.45278
IL1R2	Interleukin 1 Receptor Type 2	4.45162
MEIS1	Meis Homeobox 1	4.44973
CCDC62	Coiled-Coil Domain Containing 62	4.44631
GNG7	G Protein Subunit Gamma 7	4.44461
PAK6	P21 (RAC1) Activated Kinase 6	4.44413
EFEMP2	EGF Containing Fibulin Extracellular Matrix Protein 2	4.44254
PIK3C2A	Phosphatidylinositol-4-Phosphate 3-Kinase Catalytic Subunit Type 2 Alpha	4.44065
FAAP24	FA Core Complex Associated Protein 24	4.43880
HEY1	Hes Related Family BHLH Transcription Factor With YRPW Motif 1	4.43792
AMBRA1	Autophagy And Beclin 1 Regulator 1	4.43712
TRMU	TRNA Mitochondrial 2-Thiouridylase	4.43561
PIM2	Pim-2 Proto-Oncogene, Serine/Threonine Kinase	4.43555
ACLY	ATP Citrate Lyase	4.43535
IL1RAPL2	Interleukin 1 Receptor Accessory Protein Like 2	4.43341
BIK	BCL2 Interacting Killer	4.43284
GAGE1	G Antigen 1	4.43019

ACHE	Acetylcholinesterase (Cartwright Blood Group)	4.42951
IGSF9B	Immunoglobulin Superfamily Member 9B	4.42705
BAGE	B Melanoma Antigen	4.42542
DCAF12	DDB1 And CUL4 Associated Factor 12	4.42511
CHTF8	Chromosome Transmission Fidelity Factor 8	4.42462
ACTN1	Actinin Alpha 1	4.42327
ABO	ABO, Alpha 1-3-N-Acetylgalactosaminyltransferase And Alpha 1-3-Galactosyltransferase	4.42212
CDC37	Cell Division Cycle 37, HSP90 Cochaperone	4.42135
PTK7	Protein Tyrosine Kinase 7 (Inactive)	4.42080
LRATD2	LRAT Domain Containing 2	4.41375
FGF17	Fibroblast Growth Factor 17	4.41315
PFKM	Phosphofructokinase, Muscle	4.41290
MAT1A	Methionine Adenosyltransferase 1A	4.41132
ARHGEF16	Rho Guanine Nucleotide Exchange Factor 16	4.40954
NOB1	NIN1 (RPN12) Binding Protein 1 Homolog	4.40189
LTBP4	Latent Transforming Growth Factor Beta Binding Protein 4	4.39718
DDIT4	DNA Damage Inducible Transcript 4	4.39700
ANXA4	Annexin A4	4.39498
UBE2I	Ubiquitin Conjugating Enzyme E2 I	4.39327
MED1	Mediator Complex Subunit 1	4.39136
DEAF1	DEAF1 Transcription Factor	4.39048
CCN5	Cellular Communication Network Factor 5	4.38966
SCO2	Synthesis Of Cytochrome C Oxidase 2	4.38639
DHRS9	Dehydrogenase/Reductase 9	4.38593
NUDT6	Nudix Hydrolase 6	4.38317
FECH	Ferrochelatase	4.38273
ADSS1	Adenylosuccinate Synthase 1	4.38245
CCM2	CCM2 Scaffold Protein	4.38115
SRSF3	Serine And Arginine Rich Splicing Factor 3	4.37959
SUV39H1	Suppressor Of Variegation 3-9 Homolog 1	4.37943
PURA	Purine Rich Element Binding Protein A	4.37922
APOC3	Apolipoprotein C3	4.37880
RAC2	Rac Family Small GTPase 2	4.37792
CBFA2T3	CBFA2/RUNX1 Partner Transcriptional Co-Repressor 3	4.37738
GINS2	GINS Complex Subunit 2	4.37595

EFTUD2	Elongation Factor Tu GTP Binding Domain Containing 2	4.37560
PGAP3	Post-GPI Attachment To Proteins Phospholipase 3	4.37198
RERE	Arginine-Glutamic Acid Dipeptide Repeats	4.36968
MGA	MAX Dimerization Protein MGA	4.36753
CYP26A1	Cytochrome P450 Family 26 Subfamily A Member 1	4.36580
PRDM1	PR/SET Domain 1	4.36278
CHRNA7	Cholinergic Receptor Nicotinic Alpha 7 Subunit	4.36043
WRNIP1	WRN Helicase Interacting Protein 1	4.36017
PPFIBP2	PPFIA Binding Protein 2	4.35993
PTPRN	Protein Tyrosine Phosphatase Receptor Type N	4.35976
STC2	Stanniocalcin 2	4.35526
KDM6B	Lysine Demethylase 6B	4.35458
ELF3-AS1	ELF3 Antisense RNA 1	4.35418
DNM1L	Dynamin 1 Like	4.35362
APBB3	Amyloid Beta Precursor Protein Binding Family B Member 3	4.35309
HSD11B2	Hydroxysteroid 11-Beta Dehydrogenase 2	4.35151
NBPF3	NBPF Member 3	4.35066
E2F6	E2F Transcription Factor 6	4.35065
GAA	Alpha Glucosidase	4.34963
HOXB6	Homeobox B6	4.34557
A2ML1	Alpha-2-Macroglobulin Like 1	4.34361
MIR7-1	MicroRNA 7-1	4.34357
ZBTB7A	Zinc Finger And BTB Domain Containing 7A	4.34229
COPA	COPI Coat Complex Subunit Alpha	4.33976
SIK1	Salt Inducible Kinase 1	4.33881
MIR187	MicroRNA 187	4.33874
UPF1	UPF1 RNA Helicase And ATPase	4.33866
LINC00623	Long Intergenic Non-Protein Coding RNA 623	4.33420
CASK	Calcium/Calmodulin Dependent Serine Protein Kinase	4.33406
A4GNT	Alpha-1,4-N-Acetylglucosaminyltransferase	4.33021
HSPH1	Heat Shock Protein Family H (Hsp110) Member 1	4.32706
PINX1	PIN2 (TERF1) Interacting Telomerase Inhibitor 1	4.32633
EPPK1	Epiplakin 1	4.32575
UGT1A8	UDP Glucuronosyltransferase Family 1 Member A8	4.32366

DEPDC5	DEP Domain Containing 5, GATOR1 Subcomplex Subunit	4.32230
CYP2B6	Cytochrome P450 Family 2 Subfamily B Member 6	4.32093
LMO2	LIM Domain Only 2	4.32079
POU3F3	POU Class 3 Homeobox 3	4.31957
SELL	Selectin L	4.31903
GCK	Glucokinase	4.31556
CYP27A1	Cytochrome P450 Family 27 Subfamily A Member 1	4.31499
MADCAM1	Mucosal Vascular Addressin Cell Adhesion Molecule 1	4.31384
NPPA	Natriuretic Peptide A	4.31316
UPK1A	Uroplakin 1A	4.31156
DERPC	DERPC Proline And Glycine Rich Nuclear Protein	4.31115
GAS8-AS1	GAS8 Antisense RNA 1	4.30999
PIK3C2B	Phosphatidylinositol-4-Phosphate 3-Kinase Catalytic Subunit Type 2 Beta	4.30928
PNLIP	Pancreatic Lipase	4.30816
AHI1	Abelson Helper Integration Site 1	4.30183
SETD5	SET Domain Containing 5	4.30157
ASAP3	ArfGAP With SH3 Domain, Ankyrin Repeat And PH Domain 3	4.30142
CITED2	Cbp/P300 Interacting Transactivator With Glu/Asp Rich Carboxy-Terminal Domain 2	4.30050
FRZB	Frizzled Related Protein	4.29879
PLOD1	Procollagen-Lysine,2-Oxoglutarate 5-Dioxygenase 1	4.29877
GTF2E2	General Transcription Factor IIE Subunit 2	4.29803
CDK3	Cyclin Dependent Kinase 3	4.29753
BAIAP2L1	BAR/IMD Domain Containing Adaptor Protein 2 Like 1	4.29264
IL2RG	Interleukin 2 Receptor Subunit Gamma	4.29242
FADS1	Fatty Acid Desaturase 1	4.29238
FABP1	Fatty Acid Binding Protein 1	4.29228
RPS3	Ribosomal Protein S3	4.29182
SLC22A5	Solute Carrier Family 22 Member 5	4.29117
GRIN2B	Glutamate Ionotropic Receptor NMDA Type Subunit 2B	4.28950
DUSP6	Dual Specificity Phosphatase 6	4.28911
VIL1	Villin 1	4.28904
UBB	Ubiquitin B	4.28375
RPA2	Replication Protein A2	4.27908
NAA80	N-Alpha-Acetyltransferase 80, NatH Catalytic Subunit	4.27889

DPM1	Dolichyl-Phosphate Mannosyltransferase Subunit 1, Catalytic	4.27843
SPTBN1	Spectrin Beta, Non-Erythrocytic 1	4.27657
SIRT7	Sirtuin 7	4.27630
TTC4	Tetratricopeptide Repeat Domain 4	4.27470
KDM5C	Lysine Demethylase 5C	4.27289
IER3	Immediate Early Response 3	4.26991
HNMT	Histamine N-Methyltransferase	4.26991
RLBP1	Retinaldehyde Binding Protein 1	4.26961
NR1H4	Nuclear Receptor Subfamily 1 Group H Member 4	4.26786
ENSG00000260196		4.26268
LGALS7	Galectin 7	4.26252
LAMP2	Lysosomal Associated Membrane Protein 2	4.26236
LINC00659	Long Intergenic Non-Protein Coding RNA 659	4.26037
DSG2	Desmoglein 2	4.25965
PC	Pyruvate Carboxylase	4.25942
ARSA	Arylsulfatase A	4.25700
FOXJ1	Forkhead Box J1	4.25614
CUL4A	Cullin 4A	4.25613
PRIMPOL	Primase And DNA Directed Polymerase	4.25532
RPS6KA1	Ribosomal Protein S6 Kinase A1	4.25415
CCL18	C-C Motif Chemokine Ligand 18	4.24893
PFN1	Profilin 1	4.24545
TNKS	Tankyrase	4.24490
SCARB1	Scavenger Receptor Class B Member 1	4.24438
SURF1	SURF1 Cytochrome C Oxidase Assembly Factor	4.24109
GSTA4	Glutathione S-Transferase Alpha 4	4.24024
PDLIM1	PDZ And LIM Domain 1	4.23939
SET	SET Nuclear Proto-Oncogene	4.23911
RRP7BP	Ribosomal RNA Processing 7 Homolog B, Pseudogene	4.23910
SRARP	Steroid Receptor Associated And Regulated Protein	4.23789
TMEM97	Transmembrane Protein 97	4.23688
TRPM1	Transient Receptor Potential Cation Channel Subfamily M Member 1	4.23520
CYSLTR2	Cysteinyl Leukotriene Receptor 2	4.23117
PIK3C3	Phosphatidylinositol 3-Kinase Catalytic Subunit Type 3	4.22827
STC1	Stanniocalcin 1	4.22769
TAP2	Transporter 2, ATP Binding Cassette Subfamily B Member	4.22536
RNASE1	Ribonuclease A Family Member 1, Pancreatic	4.22522

CPS1	Carbamoyl-Phosphate Synthase 1	4.22356
MKS1	MKS Transition Zone Complex Subunit 1	4.22301
IL9	Interleukin 9	4.22258
MRI1	Methylthioribose-1-Phosphate Isomerase 1	4.22225
IRF3	Interferon Regulatory Factor 3	4.22126
ADAMTS8	ADAM Metallopeptidase With Thrombospondin Type 1 Motif 8	4.22060
DNAAF2	Dynein Axonemal Assembly Factor 2	4.22050
MIR551A	MicroRNA 551a	4.21811
CD74	CD74 Molecule	4.21788
AUTS2	Activator Of Transcription And Developmental Regulator AUTS2	4.21663
TARDBP	TAR DNA Binding Protein	4.21600
CEACAM19	CEA Cell Adhesion Molecule 19	4.21534
CYP11B1	Cytochrome P450 Family 11 Subfamily B Member 1	4.21527
LINC02154	Long Intergenic Non-Protein Coding RNA 2154	4.20970
SUMO1	Small Ubiquitin Like Modifier 1	4.20787
DRD4	Dopamine Receptor D4	4.20641
FCGR3B	Fc Fragment Of IgG Receptor IIIb	4.20595
SYNPO	Synaptopodin	4.20595
LINC02156	Long Intergenic Non-Protein Coding RNA 2156	4.20564
LINC02157	Long Intergenic Non-Protein Coding RNA 2157	4.20564
LINC02155	Long Intergenic Non-Protein Coding RNA 2155	4.20564
KRTCAP2	Keratinocyte Associated Protein 2	4.20512
CXCR6	C-X-C Motif Chemokine Receptor 6	4.20371
RAD9A	RAD9 Checkpoint Clamp Component A	4.19914
TRPC6	Transient Receptor Potential Cation Channel Subfamily C Member 6	4.19884
EMCN	Endomucin	4.19861
CXCL2	C-X-C Motif Chemokine Ligand 2	4.19704
PTPRD	Protein Tyrosine Phosphatase Receptor Type D	4.19695
ING4	Inhibitor Of Growth Family Member 4	4.19649
CCT3	Chaperonin Containing TCP1 Subunit 3	4.19633
SRP54	Signal Recognition Particle 54	4.19432
TGFBI	Transforming Growth Factor Beta Induced	4.19328
ZFPM2-AS1	ZFPM2 Antisense RNA 1	4.18956
SLC26A4	Solute Carrier Family 26 Member 4	4.18928
CREB3L2	CAMP Responsive Element Binding Protein 3 Like 2	4.18719

KHDRBS1	KH RNA Binding Domain Containing, Signal Transduction Associated 1	4.18617
SPOCK1	SPARC (Osteonectin), Cwcv And Kazal Like Domains Proteoglycan 1	4.18531
CHRM3	Cholinergic Receptor Muscarinic 3	4.18232
TNXA	Tenascin XA (Pseudogene)	4.18202
GRK5	G Protein-Coupled Receptor Kinase 5	4.18016
RAB3GAP1	RAB3 GTPase Activating Protein Catalytic Subunit 1	4.17746
ACIN1	Apoptotic Chromatin Condensation Inducer 1	4.17676
GFRA1	GDNF Family Receptor Alpha 1	4.17632
IL21R	Interleukin 21 Receptor	4.17550
MIB1	MIB E3 Ubiquitin Protein Ligase 1	4.17339
IST1	IST1 Factor Associated With ESCRT-III	4.17248
PYGO2	Pygopus Family PHD Finger 2	4.17197
DLL3	Delta Like Canonical Notch Ligand 3	4.17103
PIEZO2	Piezo Type Mechanosensitive Ion Channel Component 2	4.17102
MIR611	MicroRNA 611	4.16845
PCDH10	Protocadherin 10	4.16776
CDR1	Cerebellar Degeneration Related Protein 1	4.16762
HDAC7	Histone Deacetylase 7	4.16646
GNL3	G Protein Nucleolar 3	4.16401
SMN1	Survival Of Motor Neuron 1, Telomeric	4.16110
LIPG	Lipase G, Endothelial Type	4.15994
ALDH9A1	Aldehyde Dehydrogenase 9 Family Member A1	4.15918
STK24	Serine/Threonine Kinase 24	4.15881
HPS6	HPS6 Biogenesis Of Lysosomal Organelles Complex 2 Subunit 3	4.15831
ENSG00000261211		4.15767
ARF6	ADP Ribosylation Factor 6	4.15755
ARHGEF1	Rho Guanine Nucleotide Exchange Factor 1	4.15408
UCN2	Urocortin 2	4.15318
CTHRC1	Collagen Triple Helix Repeat Containing 1	4.15055
NUDT16L1	Nudix Hydrolase 16 Like 1	4.15043
UCP2	Uncoupling Protein 2	4.14964
MORC4	MORC Family CW-Type Zinc Finger 4	4.14797
ZNF77	Zinc Finger Protein 77	4.14418
NSUN2	NOP2/Sun RNA Methyltransferase 2	4.13965
PACS1	Phosphofurin Acidic Cluster Sorting Protein 1	4.13828
PPIF	Peptidylprolyl Isomerase F	4.13755
DLGAP4-AS1	DLGAP4 Antisense RNA 1	4.13709
GLO1	Glyoxalase I	4.13472

IL18RAP	Interleukin 18 Receptor Accessory Protein	4.13446
SLC9A2	Solute Carrier Family 9 Member A2	4.13176
SLC40A1	Solute Carrier Family 40 Member 1	4.13038
RGS10	Regulator Of G Protein Signaling 10	4.13029
HSPA1L	Heat Shock Protein Family A (Hsp70) Member 1 Like	4.12862
HDAC8	Histone Deacetylase 8	4.12825
ADGRV1	Adhesion G Protein-Coupled Receptor V1	4.12759
GNG12	G Protein Subunit Gamma 12	4.12686
KRR1	KRR1 Small Subunit Processome Component Homolog	4.12606
MIR518A1	MicroRNA 518a-1	4.12590
EIF3D	Eukaryotic Translation Initiation Factor 3 Subunit D	4.12245
BRI3BP	BRI3 Binding Protein	4.12205
IL17B	Interleukin 17B	4.12130
PSMA7	Proteasome 20S Subunit Alpha 7	4.12108
THBS2	Thrombospondin 2	4.12022
HMBS	Hydroxymethylbilane Synthase	4.11749
MBD2	Methyl-CpG Binding Domain Protein 2	4.11742
CHIT1	Chitinase 1	4.11741
PMM2	Phosphomannomutase 2	4.11715
CASZ1	Castor Zinc Finger 1	4.11704
EXOSC3	Exosome Component 3	4.11578
S100A12	S100 Calcium Binding Protein A12	4.11499
PCBP2	Poly(RC) Binding Protein 2	4.11435
RPTOR	Regulatory Associated Protein Of MTOR Complex 1	4.11395
FBXO31	F-Box Protein 31	4.11360
EIF5A	Eukaryotic Translation Initiation Factor 5A	4.10925
SIRT6	Sirtuin 6	4.10532
SMAD9	SMAD Family Member 9	4.10503
POLR2A	RNA Polymerase II Subunit A	4.10328
CPE	Carboxypeptidase E	4.10226
SMTN	Smoothelin	4.09972
BEST1	Bestrophin 1	4.09786
RO60	Ro60, Y RNA Binding Protein	4.09119
RBPJ	Recombination Signal Binding Protein For Immunoglobulin Kappa J Region	4.09088
PDYN	Prodynorphin	4.09039
ITGB5	Integrin Subunit Beta 5	4.08970
SORBS1	Sorbin And SH3 Domain Containing 1	4.08947
CAMKK2	Calcium/Calmodulin Dependent Protein Kinase Kinase 2	4.08658
ACTR3	Actin Related Protein 3	4.08521

ELL	Elongation Factor For RNA Polymerase II	4.08430
CLSPN	Claspin	4.08219
BCORL1	BCL6 Corepressor Like 1	4.08172
CAPZB	Capping Actin Protein Of Muscle Z-Line Subunit Beta	4.08138
LRRK2	Leucine Rich Repeat Kinase 2	4.08118
GANAB	Glucosidase II Alpha Subunit	4.07921
GTF2H4	General Transcription Factor IIH Subunit 4	4.07801
MIR26A2	MicroRNA 26a-2	4.07694
ALDH1L1	Aldehyde Dehydrogenase 1 Family Member L1	4.07360
CCL26	C-C Motif Chemokine Ligand 26	4.07315
NR5A2	Nuclear Receptor Subfamily 5 Group A Member 2	4.07310
UGT1A	UDP Glucuronosyltransferase Family 1 Member A Complex Locus	4.07220
MLLT1	MLLT1 Super Elongation Complex Subunit	4.07126
PAFAH1B1	Platelet Activating Factor Acetylhydrolase 1b Regulatory Subunit 1	4.07116
PURPL	P53 Upregulated Regulator Of P53 Levels	4.07099
CAPN2	Calpain 2	4.06956
POU2F1	POU Class 2 Homeobox 1	4.06948
GALNS	Galactosamine (N-Acetyl)-6-Sulfatase	4.06856
TUBB2B	Tubulin Beta 2B Class IIb	4.06647
CAMK2G	Calcium/Calmodulin Dependent Protein Kinase II Gamma	4.05539
DCLRE1C	DNA Cross-Link Repair 1C	4.05481
MIR711	MicroRNA 711	4.05438
DIRC1	Disrupted In Renal Carcinoma 1	4.05349
PERP	P53 Apoptosis Effector Related To PMP22	4.05325
AKR1B1	Aldo-Keto Reductase Family 1 Member B	4.05200
KIF7	Kinesin Family Member 7	4.05155
SCARB2	Scavenger Receptor Class B Member 2	4.05073
GC	GC Vitamin D Binding Protein	4.04985
TFEB	Transcription Factor EB	4.04835
TMPO	Thymopoietin	4.04651
PRKCG	Protein Kinase C Gamma	4.04503
POLD3	DNA Polymerase Delta 3, Accessory Subunit	4.04278
RIOK1	RIO Kinase 1	4.04164
TNNI2	Troponin I2, Fast Skeletal Type	4.04135
CSAG2	CSAG Family Member 2	4.04104

LIPC	Lipase C, Hepatic Type	4.03960
NME2	NME/NM23 Nucleoside Diphosphate Kinase 2	4.03781
DDHD2	DDHD Domain Containing 2	4.03523
HLA-DPA1	Major Histocompatibility Complex, Class II, DP Alpha 1	4.03486
LGR4	Leucine Rich Repeat Containing G Protein-Coupled Receptor 4	4.03383
PKN3	Protein Kinase N3	4.03300
POR	Cytochrome P450 Oxidoreductase	4.03194
SMN2	Survival Of Motor Neuron 2, Centromeric	4.02985
MAGEA12	MAGE Family Member A12	4.02600
TFDP3	Transcription Factor Dp Family Member 3	4.02597
HSPA1B	Heat Shock Protein Family A (Hsp70) Member 1B	4.02429
CEP192	Centrosomal Protein 192	4.02384
RTKN	Rhotekin	4.01880
FLAD1	Flavin Adenine Dinucleotide Synthetase 1	4.01818
GPBAR1	G Protein-Coupled Bile Acid Receptor 1	4.01783
LAMA5	Laminin Subunit Alpha 5	4.01410
TOP3A	DNA Topoisomerase III Alpha	4.01077
HSD11B1	Hydroxysteroid 11-Beta Dehydrogenase 1	4.01028
BTG1	BTG Anti-Proliferation Factor 1	4.00835
MIR518D	MicroRNA 518d	4.00554
TRPM7	Transient Receptor Potential Cation Channel Subfamily M Member 7	4.00319
STK36	Serine/Threonine Kinase 36	4.00229
SLC25A4	Solute Carrier Family 25 Member 4	4.00124
CANX	Calnexin	4.00089
LNCNEF	LncRNA Neighboring Enhancer Of FOXA2	3.99625
PKD2	Polycystin 2, Transient Receptor Potential Cation Channel	3.99623
CLDN2	Claudin 2	3.99470
GET4	Guided Entry Of Tail-Anchored Proteins Factor 4	3.99447
RAB23	RAB23, Member RAS Oncogene Family	3.99391
TRIP11	Thyroid Hormone Receptor Interactor 11	3.99381
ITPKB	Inositol-Trisphosphate 3-Kinase B	3.99380
ATF7	Activating Transcription Factor 7	3.99215
VAMP1	Vesicle Associated Membrane Protein 1	3.98975
ILF3	Interleukin Enhancer Binding Factor 3	3.98944
SLC6A6	Solute Carrier Family 6 Member 6	3.98860

APPL1	Adaptor Protein, Phosphotyrosine Interacting With PH Domain And Leucine Zipper 1	3.98747
NEK6	NIMA Related Kinase 6	3.98700
LOXL1	Lysyl Oxidase Like 1	3.98636
ISG20	Interferon Stimulated Exonuclease Gene 20	3.98453
HPS5	HPS5 Biogenesis Of Lysosomal Organelles Complex 2 Subunit 2	3.98300
ERAS	ES Cell Expressed Ras	3.98207
PAF1	PAF1 Homolog, Paf1/RNA Polymerase II Complex Component	3.98158
TACR2	Tachykinin Receptor 2	3.98154
TNS3	Tensin 3	3.98097
CX3CL1	C-X3-C Motif Chemokine Ligand 1	3.98022
ARAP3	ArfGAP With RhoGAP Domain, Ankyrin Repeat And PH Domain 3	3.97889
TLX1	T Cell Leukemia Homeobox 1	3.97503
KRT6A	Keratin 6A	3.97282
RFX1	Regulatory Factor X1	3.97225
CRYAB	Crystallin Alpha B	3.96998
ACP1	Acid Phosphatase 1	3.96915
NAP1L4	Nucleosome Assembly Protein 1 Like 4	3.96897
H3C4	H3 Clustered Histone 4	3.96881
ITGB7	Integrin Subunit Beta 7	3.96876
MT-ND5	Mitochondrially Encoded NADH:Ubiquinone Oxidoreductase Core Subunit 5	3.96857
ACER3	Alkaline Ceramidase 3	3.96834
TBC1D24	TBC1 Domain Family Member 24	3.96497
DPAGT1	Dolichyl-Phosphate N-Acetylglucosaminephosphotransferase 1	3.96488
CTNNBIP1	Catenin Beta Interacting Protein 1	3.96336
TUBG1	Tubulin Gamma 1	3.96164
ACAN	Aggrecan	3.95996
TMPRSS6	Transmembrane Serine Protease 6	3.95806
MRPS11	Mitochondrial Ribosomal Protein S11	3.95777
UROD	Uroporphyrinogen Decarboxylase	3.95676
FAM189B	Family With Sequence Similarity 189 Member B	3.95596
CFL1	Cofilin 1	3.95360
CALM1	Calmodulin 1	3.95309
GALR1	Galanin Receptor 1	3.95233
THRA	Thyroid Hormone Receptor Alpha	3.95035
TGIF2	TGFB Induced Factor Homeobox 2	3.94800
TOPBP1	DNA Topoisomerase II Binding Protein 1	3.94673
DGKE	Diacylglycerol Kinase Epsilon	3.94619
SIX4	SIX Homeobox 4	3.94607

NFIX	Nuclear Factor I X	3.94450
KIF4A	Kinesin Family Member 4A	3.94177
RPLP0	Ribosomal Protein Lateral Stalk Subunit P0	3.93880
ITK	IL2 Inducible T Cell Kinase	3.93867
AFAP1L2	Actin Filament Associated Protein 1 Like 2	3.93810
TPT1	Tumor Protein, Translationally-Controlled 1	3.93732
TNFRSF17	TNF Receptor Superfamily Member 17	3.93654
DNAAF5	Dynein Axonemal Assembly Factor 5	3.93470
SPRY2	Sprouty RTK Signaling Antagonist 2	3.93440
SFTPB	Surfactant Protein B	3.93320
SSBP3	Single Stranded DNA Binding Protein 3	3.93168
TNFRSF12A	TNF Receptor Superfamily Member 12A	3.93156
SIPA1	Signal-Induced Proliferation-Associated 1	3.93111
ADRA2A	Adrenoceptor Alpha 2A	3.93038
B3GNT6	UDP-GlcNAc:BetaGal Beta-1,3-N-Acetylglucosaminyltransferase 6	3.92928
MLST8	MTOR Associated Protein, LST8 Homolog	3.92818
HES5	Hes Family BHLH Transcription Factor 5	3.92807
HSPA9	Heat Shock Protein Family A (Hsp70) Member 9	3.92559
MEF2D	Myocyte Enhancer Factor 2D	3.92543
ZNF350	Zinc Finger Protein 350	3.92529
YBX3	Y-Box Binding Protein 3	3.92470
ST3GAL3	ST3 Beta-Galactoside Alpha-2,3-Sialyltransferase 3	3.92459
RDX	Radixin	3.92351
MSTO1	Misato Mitochondrial Distribution And Morphology Regulator 1	3.92171
SDC4	Syndecan 4	3.91969
AIM2	Absent In Melanoma 2	3.91861
SP7	Sp7 Transcription Factor	3.91582
CRAT	Carnitine O-Acetyltransferase	3.91538
TRAF7	TNF Receptor Associated Factor 7	3.91003
MINDY3	MINDY Lysine 48 Deubiquitinase 3	3.90966
CLDN11	Claudin 11	3.90769
FXN	Frataxin	3.90672
CAPN8	Calpain 8	3.90663
POU2F3	POU Class 2 Homeobox 3	3.90599
PEPD	Peptidase D	3.90535
NDN	Necdin, MAGE Family Member	3.90470
ODF2	Outer Dense Fiber Of Sperm Tails 2	3.90302
FLOT1	Flotillin 1	3.90281

ADCYAP1	Adenylate Cyclase Activating Polypeptide 1	3.90216
SLC16A4	Solute Carrier Family 16 Member 4	3.90111
LGI4	Leucine Rich Repeat LGI Family Member 4	3.90066
EBF3	EBF Transcription Factor 3	3.89998
UCP3	Uncoupling Protein 3	3.89918
BCLAF1	BCL2 Associated Transcription Factor 1	3.89753
EIF4A1	Eukaryotic Translation Initiation Factor 4A1	3.89725
G3BP2	G3BP Stress Granule Assembly Factor 2	3.89688
FGF20	Fibroblast Growth Factor 20	3.89263
MYO7A	Myosin VIIA	3.89176
NCF1	Neutrophil Cytosolic Factor 1	3.89111
RNF114	Ring Finger Protein 114	3.89040
CCL13	C-C Motif Chemokine Ligand 13	3.88696
MYCT1	MYC Target 1	3.88449
DLG1	Discs Large MAGUK Scaffold Protein 1	3.88378
GNS	Glucosamine (N-Acetyl)-6-Sulfatase	3.88277
TBXAS1	Thromboxane A Synthase 1	3.88222
NBEAL2	Neurobeachin Like 2	3.88161
TMEM170B	Transmembrane Protein 170B	3.88113
BBIP1	BBSome Interacting Protein 1	3.88074
USP48	Ubiquitin Specific Peptidase 48	3.88074
HIVEP1	HIVEP Zinc Finger 1	3.87952
SLC1A5	Solute Carrier Family 1 Member 5	3.87909
C5AR1	Complement C5a Receptor 1	3.87692
PRPS1	Phosphoribosyl Pyrophosphate Synthetase 1	3.87622
VASP	Vasodilator Stimulated Phosphoprotein	3.87557
CCR9	C-C Motif Chemokine Receptor 9	3.87513
TOP2B	DNA Topoisomerase II Beta	3.87457
CD69	CD69 Molecule	3.87360
ASH1L	ASH1 Like Histone Lysine Methyltransferase	3.87280
EFNA4	Ephrin A4	3.87261
MIR217	MicroRNA 217	3.87209
PSMG4	Proteasome Assembly Chaperone 4	3.87162
MIR367	MicroRNA 367	3.86870
BCL2A1	BCL2 Related Protein A1	3.86746
NTF3	Neurotrophin 3	3.86663
FLOT2	Flotillin 2	3.86549
NDFIP1	Nedd4 Family Interacting Protein 1	3.86416
TM4SF1	Transmembrane 4 L Six Family Member 1	3.86372
PLCD1	Phospholipase C Delta 1	3.86204
PFKL	Phosphofructokinase, Liver Type	3.86192
SLC9B1	Solute Carrier Family 9 Member B1	3.86164
ADRA1A	Adrenoceptor Alpha 1A	3.86074

ATXN10	Ataxin 10	3.86061
ADGRG1	Adhesion G Protein-Coupled Receptor G1	3.86036
LIMK2	LIM Domain Kinase 2	3.85908
GLDC	Glycine Decarboxylase	3.85853
GDF3	Growth Differentiation Factor 3	3.85780
CILK1	Ciliogenesis Associated Kinase 1	3.85761
MARK2	Microtubule Affinity Regulating Kinase 2	3.85702
UBTF	Upstream Binding Transcription Factor	3.85618
SFPQ	Splicing Factor Proline And Glutamine Rich	3.85533
CLK2	CDC Like Kinase 2	3.85410
CLASRP	CLK4 Associating Serine/Arginine Rich Protein	3.85109
TCTN3	Tectonic Family Member 3	3.85076
DAG1	Dystroglycan 1	3.84987
FABP6	Fatty Acid Binding Protein 6	3.84922
CFAP410	Cilia And Flagella Associated Protein 410	3.84893
WDR5	WD Repeat Domain 5	3.84849
ENSG00000266680		3.84754
TMEM51-AS1	TMEM51 Antisense RNA 1	3.84686
GALNT3	Polypeptide N-Acetylgalactosaminyltransferase 3	3.84672
ATP1A1	ATPase Na+/K+ Transporting Subunit Alpha 1	3.84570
OIP5	Opa Interacting Protein 5	3.84500
GCH1	GTP Cyclohydrolase 1	3.84381
AZIN1	Antizyme Inhibitor 1	3.84272
MIR874	MicroRNA 874	3.83993
MAN2B1	Mannosidase Alpha Class 2B Member 1	3.83951
KDM8	Lysine Demethylase 8	3.83935
CCL14	C-C Motif Chemokine Ligand 14	3.83865
MARK1	Microtubule Affinity Regulating Kinase 1	3.83764
HAX1	HCLS1 Associated Protein X-1	3.83755
KIF14	Kinesin Family Member 14	3.83540
FADS2	Fatty Acid Desaturase 2	3.83110
IVL	Involucrin	3.83066
VGF	VGF Nerve Growth Factor Inducible	3.82915
H2BC21	H2B Clustered Histone 21	3.82849
WARS1	Tryptophanyl-TRNA Synthetase 1	3.82727
SNW1	SNW Domain Containing 1	3.82657
PIM3	Pim-3 Proto-Oncogene, Serine/Threonine Kinase	3.82635
VCL	Vinculin	3.82631
TICAM1	Toll Like Receptor Adaptor Molecule 1	3.82583

MAP3K14	Mitogen-Activated Protein Kinase Kinase Kinase 14	3.82429
MAPK11	Mitogen-Activated Protein Kinase 11	3.82398
MLPH	Melanophilin	3.82256
CISH	Cytokine Inducible SH2 Containing Protein	3.82241
IFNGR2	Interferon Gamma Receptor 2	3.82156
MR1	Major Histocompatibility Complex, Class I-Related	3.82051
DZIP1L	DAZ Interacting Zinc Finger Protein 1 Like	3.81917
FBP1	Fructose-Bisphosphatase 1	3.81844
ALPG	Alkaline Phosphatase, Germ Cell	3.81740
PPARGC1B	PPARG Coactivator 1 Beta	3.81731
HCK	HCK Proto-Oncogene, Src Family Tyrosine Kinase	3.81679
CLMP	CXADR Like Membrane Protein	3.81535
RBM47	RNA Binding Motif Protein 47	3.81475
RELN	Reelin	3.81379
BRF1	BRF1 RNA Polymerase III Transcription Initiation Factor Subunit	3.81288
DSCC1	DNA Replication And Sister Chromatid Cohesion 1	3.81263
ZCCHC8	Zinc Finger CCHC-Type Containing 8	3.81258
SCNN1A	Sodium Channel Epithelial 1 Subunit Alpha	3.81147
TPI1	Triosephosphate Isomerase 1	3.81070
HTR4	5-Hydroxytryptamine Receptor 4	3.81004
SLC18A2	Solute Carrier Family 18 Member A2	3.80919
SIRT4	Sirtuin 4	3.80875
ABHD16A	Abhydrolase Domain Containing 16A, Phospholipase	3.80875
NARS1	Asparaginyl-TRNA Synthetase 1	3.80695
NEO1	Neogenin 1	3.80683
PF4	Platelet Factor 4	3.80675
TWIST2	Twist Family BHLH Transcription Factor 2	3.80623
TASOR2	Transcription Activation Suppressor Family Member 2	3.80610
ACY1	Aminoacylase 1	3.80567
XAB2	XPA Binding Protein 2	3.80367
TARBP2	TARBP2 Subunit Of RISC Loading Complex	3.80360
UBAP2L	Ubiquitin Associated Protein 2 Like	3.80312
MAOA	Monoamine Oxidase A	3.80300
ST6GALNAC6	ST6 N-Acetylgalactosaminide Alpha-2,6-Sialyltransferase 6	3.80210
DDX21	DExD-Box Helicase 21	3.80168
TBX1	T-Box Transcription Factor 1	3.79982

ACAP1	ArfGAP With Coiled-Coil, Ankyrin Repeat And PH Domains 1	3.79712
VEZT	Vezatin, Adherens Junctions Transmembrane Protein	3.79508
KAT2A	Lysine Acetyltransferase 2A	3.79493
TNFAIP2	TNF Alpha Induced Protein 2	3.79309
DLEU7-AS1	DLEU7 Antisense RNA 1	3.79256
ICAM2	Intercellular Adhesion Molecule 2	3.79015
LAMB1	Laminin Subunit Beta 1	3.78930
PIWIL2	Piwi Like RNA-Mediated Gene Silencing 2	3.78817
BCL2L12	BCL2 Like 12	3.78798
MAPKAPK3	MAPK Activated Protein Kinase 3	3.78785
SUPT5H	SPT5 Homolog, DSIF Elongation Factor Subunit	3.78595
KARS1	Lysyl-TRNA Synthetase 1	3.78543
PFDN1	Prefoldin Subunit 1	3.78426
USP36	Ubiquitin Specific Peptidase 36	3.78407
MIR20B	MicroRNA 20b	3.78329
LONP1	Lon Peptidase 1, Mitochondrial	3.78300
PTBP3	Polypyrimidine Tract Binding Protein 3	3.78089
SIN3A	SIN3 Transcription Regulator Family Member A	3.77992
CYP4B1	Cytochrome P450 Family 4 Subfamily B Member 1	3.77900
BLOC1S6	Biogenesis Of Lysosomal Organelles Complex 1 Subunit 6	3.77583
RPRD1B	Regulation Of Nuclear Pre-MRNA Domain Containing 1B	3.77346
HYOU1	Hypoxia Up-Regulated 1	3.77247
EVPL	Envoplakin	3.77205
PAX4	Paired Box 4	3.77102
VSIR	V-Set Immunoregulatory Receptor	3.76917
PDK2	Pyruvate Dehydrogenase Kinase 2	3.76752
THADA	THADA Armadillo Repeat Containing	3.76738
EED	Embryonic Ectoderm Development	3.76547
MSH5	MutS Homolog 5	3.76546
CKAP2	Cytoskeleton Associated Protein 2	3.76505
DKK2	Dickkopf WNT Signaling Pathway Inhibitor 2	3.76434
MAP3K11	Mitogen-Activated Protein Kinase Kinase Kinase 11	3.76214
PHBP1	Prohibitin Pseudogene 1	3.76137
BAG6	BAG Cochaperone 6	3.76093
MERTK	MER Proto-Oncogene, Tyrosine Kinase	3.76082
MMEL1	Membrane Metalloendopeptidase Like 1	3.76033
EIF4B	Eukaryotic Translation Initiation Factor 4B	3.75935
MEAF6	MYST/Esa1 Associated Factor 6	3.75833

TEX101	Testis Expressed 101	3.75728
PTCSC3	Papillary Thyroid Carcinoma Susceptibility Candidate 3	3.75705
PDLIM2	PDZ And LIM Domain 2	3.75696
ATP1A3	ATPase Na+/K+ Transporting Subunit Alpha 3	3.75659
CAHM	Colon Adenocarcinoma Hypermethylated	3.75459
GPI	Glucose-6-Phosphate Isomerase	3.75450
POLR2F	RNA Polymerase II, I And III Subunit F	3.75070
LINC02098	Long Intergenic Non-Protein Coding RNA 2098	3.75058
LINC02231	Long Intergenic Non-Protein Coding RNA 2231	3.75058
PSPC1-AS2	PSPC1 Antisense RNA 2	3.75058
ZNF292	Zinc Finger Protein 292	3.74902
AAMP	Angio Associated Migratory Cell Protein	3.74898
GNA13	G Protein Subunit Alpha 13	3.74888
DAP3	Death Associated Protein 3	3.74771
COPS3	COP9 Signalosome Subunit 3	3.74757
ACKR1	Atypical Chemokine Receptor 1 (Duffy Blood Group)	3.74647
ENSG00000284669		3.74563
TAF1	TATA-Box Binding Protein Associated Factor 1	3.74314
NAIF1	Nuclear Apoptosis Inducing Factor 1	3.74287
PITX2	Paired Like Homeodomain 2	3.74284
MROCKI	MARCKS Cis Regulating LncRNA Promoter Of Cytokines And Inflammation	3.74267
MFSD2A	Major Facilitator Superfamily Domain Containing 2A	3.74233
SGSH	N-Sulfoglucosamine Sulfohydrolase	3.74133
MRPL58	Mitochondrial Ribosomal Protein L58	3.73974
HDAC10	Histone Deacetylase 10	3.73847
DKKL1	Dickkopf Like Acrosomal Protein 1	3.73450
LOC10662798	GBA Recombination Region	3.73389
PRDM5	PR/SET Domain 5	3.73380
SCN4A	Sodium Voltage-Gated Channel Alpha Subunit 4	3.73133
GUK1	Guanylate Kinase 1	3.73022
CCL7	C-C Motif Chemokine Ligand 7	3.72811
GUCA1A	Guanylate Cyclase Activator 1A	3.72741
NCF4-AS1	NCF4 Antisense RNA 1	3.72702
TRPM2	Transient Receptor Potential Cation Channel Subfamily M Member 2	3.72583
FLVCR1	FLVCR Heme Transporter 1	3.72539

MAGI1	Membrane Associated Guanylate Kinase, WW And PDZ Domain Containing 1	3.72526
NR1H3	Nuclear Receptor Subfamily 1 Group H Member 3	3.72426
TP53RK	TP53 Regulating Kinase	3.72339
CAMK2A	Calcium/Calmodulin Dependent Protein Kinase II Alpha	3.72260
SULT2A1	Sulfotransferase Family 2A Member 1	3.72258
OSGIN2	Oxidative Stress Induced Growth Inhibitor Family Member 2	3.71826
SCG5	Secretogranin V	3.71817
SP3	Sp3 Transcription Factor	3.71729
RNF216	Ring Finger Protein 216	3.71728
JMJD1C	Jumonji Domain Containing 1C	3.71623
BCCIP	BRCA2 And CDKN1A Interacting Protein	3.71566
MIR299	MicroRNA 299	3.71499
CAMP	Cathelicidin Antimicrobial Peptide	3.71477
MTM1	Myotubularin 1	3.71474
RAB11FIP4	RAB11 Family Interacting Protein 4	3.71461
GSK3A	Glycogen Synthase Kinase 3 Alpha	3.71439
CLCN7	Chloride Voltage-Gated Channel 7	3.71417
HSD17B2	Hydroxysteroid 17-Beta Dehydrogenase 2	3.71383
MIR760	MicroRNA 760	3.71364
RHPN2	Rhophilin Rho GTPase Binding Protein 2	3.71234
DHX9	DExH-Box Helicase 9	3.71171
RNF8	Ring Finger Protein 8	3.71134
POLR1D	RNA Polymerase I And III Subunit D	3.71099
RAD18	RAD18 E3 Ubiquitin Protein Ligase	3.71042
MARK3	Microtubule Affinity Regulating Kinase 3	3.70949
PRKAR2A	Protein Kinase CAMP-Dependent Type II Regulatory Subunit Alpha	3.70844
LORICRIN	Loricrin Cornified Envelope Precursor Protein	3.70796
GALK1	Galactokinase 1	3.70700
GAD1	Glutamate Decarboxylase 1	3.70682
DAP	Death Associated Protein	3.70666
RHBDF1	Rhomboid 5 Homolog 1	3.70614
POGZ	Pogo Transposable Element Derived With ZNF Domain	3.70554
FHL1	Four And A Half LIM Domains 1	3.70395
XYLT2	Xylosyltransferase 2	3.70369
FBN2	Fibrillin 2	3.70151
MXD1	MAX Dimerization Protein 1	3.70127
MPG	N-Methylpurine DNA Glycosylase	3.69988

EME1	Essential Meiotic Structure-Specific Endonuclease 1	3.69599
CHTF18	Chromosome Transmission Fidelity Factor 18	3.69585
SLC26A2	Solute Carrier Family 26 Member 2	3.69537
TAF8	TATA-Box Binding Protein Associated Factor 8	3.69482
CAVIN3	Caveolae Associated Protein 3	3.69456
UBE2V1	Ubiquitin Conjugating Enzyme E2 V1	3.69449
SLC22A4	Solute Carrier Family 22 Member 4	3.69303
ECD	Ecdysoneless Cell Cycle Regulator	3.69110
E2F8	E2F Transcription Factor 8	3.69093
TRIM8	Tripartite Motif Containing 8	3.69088
FTH1	Ferritin Heavy Chain 1	3.69065
HARBI1	Harbinger Transposase Derived 1	3.69038
SMARCA1	SWI/SNF Related, Matrix Associated, Actin Dependent Regulator Of Chromatin, Subfamily A, Member 1	3.68896
NCAPG	Non-SMC Condensin I Complex Subunit G	3.68850
TSEN54	TRNA Splicing Endonuclease Subunit 54	3.68826
CCN3	Cellular Communication Network Factor 3	3.68759
PHF20	PHD Finger Protein 20	3.68632
PPP1R15A	Protein Phosphatase 1 Regulatory Subunit 15A	3.68281
TTF1	Transcription Termination Factor 1	3.68116
ADAM15	ADAM Metallopeptidase Domain 15	3.68015
MZB1	Marginal Zone B And B1 Cell Specific Protein	3.67925
F8	Coagulation Factor VIII	3.67901
AQP5	Aquaporin 5	3.67776
MIR1266	MicroRNA 1266	3.67715
PGPEP1	Pyroglutamyl-Peptidase I	3.67457
CA1	Carbonic Anhydrase 1	3.67424
USP10	Ubiquitin Specific Peptidase 10	3.67332
ANAPC16	Anaphase Promoting Complex Subunit 16	3.67227
RABIF	RAB Interacting Factor	3.67146
ISL1	ISL LIM Homeobox 1	3.67134
KPNA2	Karyopherin Subunit Alpha 2	3.67126
ACYP2	Acylphosphatase 2	3.67044
MAPKAPK2	MAPK Activated Protein Kinase 2	3.66921
FRS2	Fibroblast Growth Factor Receptor Substrate 2	3.66854
LZTS2	Leucine Zipper Tumor Suppressor 2	3.66848
TEP1	Telomerase Associated Protein 1	3.66838

LAMP1	Lysosomal Associated Membrane Protein 1	3.66571
CYB561D2	Cytochrome B561 Family Member D2	3.66405
MMP19	Matrix Metallopeptidase 19	3.66381
FATE1	Fetal And Adult Testis Expressed 1	3.66137
PUF60	Poly(U) Binding Splicing Factor 60	3.66048
DYRK1A	Dual Specificity Tyrosine Phosphorylation Regulated Kinase 1A	3.65790
OBSCN	Obscurin, Cytoskeletal Calmodulin And Titin-Interacting RhoGEF	3.65669
PDIA3	Protein Disulfide Isomerase Family A Member 3	3.65548
ALDOB	Aldolase, Fructose-Bisphosphate B	3.65525
MCM3AP	Minichromosome Maintenance Complex Component 3 Associated Protein	3.65477
GON4L	Gon-4 Like	3.65411
ELOB	Elongin B	3.65329
CPT1A	Carnitine Palmitoyltransferase 1A	3.65304
TRIM29	Tripartite Motif Containing 29	3.65277
PSMC4	Proteasome 26S Subunit, ATPase 4	3.65002
EPS8	Epidermal Growth Factor Receptor Pathway Substrate 8	3.64885
GSTA2	Glutathione S-Transferase Alpha 2	3.64660
MARS1	Methionyl-TRNA Synthetase 1	3.64492
NEK9	NIMA Related Kinase 9	3.63966
MZF1	Myeloid Zinc Finger 1	3.63791
STAT2	Signal Transducer And Activator Of Transcription 2	3.63708
FUCA2	Alpha-L-Fucosidase 2	3.63677
CDK11A	Cyclin Dependent Kinase 11A	3.63675
SLC26A3	Solute Carrier Family 26 Member 3	3.63589
SEC11A	SEC11 Homolog A, Signal Peptidase Complex Subunit	3.63522
FARP1	FERM, ARH/RhoGEF And Pleckstrin Domain Protein 1	3.63425
OCRL	OCRL Inositol Polyphosphate-5-Phosphatase	3.63360
DM1-AS	DM1 Locus Antisense RNA	3.63059
PACSIN2	Protein Kinase C And Casein Kinase Substrate In Neurons 2	3.63042
PBXIP1	PBX Homeobox Interacting Protein 1	3.62971
SLC7A7	Solute Carrier Family 7 Member 7	3.62964
SAPCD2	Suppressor APC Domain Containing 2	3.62831
EIF2AK3	Eukaryotic Translation Initiation Factor 2 Alpha Kinase 3	3.62720
LRRC3B	Leucine Rich Repeat Containing 3B	3.62683
GFI1	Growth Factor Independent 1 Transcriptional Repressor	3.62672

FZD9	Frizzled Class Receptor 9	3.62624
RAD23A	RAD23 Homolog A, Nucleotide Excision Repair Protein	3.62447
ENSG00000261220		3.62435
NAA40	N-Alpha-Acetyltransferase 40, NatD Catalytic Subunit	3.62416
MICOS10	Mitochondrial Contact Site And Cristae Organizing System Subunit 10	3.62259
FCN2	Ficolin 2	3.62246
EPHA8	EPH Receptor A8	3.62239
HUS1	HUS1 Checkpoint Clamp Component	3.62182
MATR3	Matrin 3	3.62041
INSM1	INSM Transcriptional Repressor 1	3.61992
LATS1	Large Tumor Suppressor Kinase 1	3.61939
TRPM4	Transient Receptor Potential Cation Channel Subfamily M Member 4	3.61857
ACTL8	Actin Like 8	3.61671
GRM6	Glutamate Metabotropic Receptor 6	3.61616
ST3GAL1	ST3 Beta-Galactoside Alpha-2,3-Sialyltransferase 1	3.61484
OGN	Osteoglycin	3.61472
FKBP10	FKBP Prolyl Isomerase 10	3.61209
RPL23	Ribosomal Protein L23	3.61204
MIA	MIA SH3 Domain Containing	3.61138
NUF2	NUF2 Component Of NDC80 Kinetochore Complex	3.60997
SERPINB1	Serpin Family B Member 1	3.60984
TEAD3	TEA Domain Transcription Factor 3	3.60977
AHDC1	AT-Hook DNA Binding Motif Containing 1	3.60823
IRX2	Iroquois Homeobox 2	3.60509
CCN6	Cellular Communication Network Factor 6	3.60458
ATAD3B	ATPase Family AAA Domain Containing 3B	3.60323
IRF7	Interferon Regulatory Factor 7	3.60300
GCLM	Glutamate-Cysteine Ligase Modifier Subunit	3.60173
HNRNPF	Heterogeneous Nuclear Ribonucleoprotein F	3.60140
PA2G4	Proliferation-Associated 2G4	3.60112
MYEOV	Myeloma Overexpressed	3.60085
GRK6	G Protein-Coupled Receptor Kinase 6	3.60032
PLAC8	Placenta Associated 8	3.59979
IL19	Interleukin 19	3.59865
LAMB2	Laminin Subunit Beta 2	3.59863
KALRN	Kalirin RhoGEF Kinase	3.59846
MYBL2	MYB Proto-Oncogene Like 2	3.59835
BANP	BTG3 Associated Nuclear Protein	3.59763

PTGER1	Prostaglandin E Receptor 1	3.59687
NDUFS8	NADH:Ubiquinone Oxidoreductase Core Subunit S8	3.59660
ARHGAP45	Rho GTPase Activating Protein 45	3.59506
NUP85	Nucleoporin 85	3.59434
SIM2	SIM BHLH Transcription Factor 2	3.59372
GNA14	G Protein Subunit Alpha 14	3.59304
CEP78	Centrosomal Protein 78	3.59303
ZFX	Zinc Finger Protein X-Linked	3.59294
FBL	Fibrillarin	3.59262
ZIC1	Zic Family Member 1	3.59110
FAAH	Fatty Acid Amide Hydrolase	3.59053
SSTR4	Somatostatin Receptor 4	3.59045
GCDH	Glutaryl-CoA Dehydrogenase	3.58929
PRKAR2B	Protein Kinase CAMP-Dependent Type II Regulatory Subunit Beta	3.58917
SOX18	SRY-Box Transcription Factor 18	3.58902
SEMA5A	Semaphorin 5A	3.58847
UGT1A10	UDP Glucuronosyltransferase Family 1 Member A10	3.58842
HRH1	Histamine Receptor H1	3.58719
PHF13	PHD Finger Protein 13	3.58667
NRXN1	Neurexin 1	3.58635
CAPN1	Calpain 1	3.58457
SCN9A	Sodium Voltage-Gated Channel Alpha Subunit 9	3.58228
LGI1	Leucine Rich Glioma Inactivated 1	3.58048
CNOT3	CCR4-NOT Transcription Complex Subunit 3	3.58008
CCNG2	Cyclin G2	3.57944
C12orf57	Chromosome 12 Open Reading Frame 57	3.57848
TSSK6	Testis Specific Serine Kinase 6	3.57812
MAP3K3	Mitogen-Activated Protein Kinase Kinase Kinase 3	3.57782
SH2D1A	SH2 Domain Containing 1A	3.57724
KCNN4	Potassium Calcium-Activated Channel Subfamily N Member 4	3.57624
TNFSF8	TNF Superfamily Member 8	3.57616
CUL4B	Cullin 4B	3.57509
GNAI3	G Protein Subunit Alpha I3	3.57474
TCF21	Transcription Factor 21	3.57447
ACTR2	Actin Related Protein 2	3.57437
NSD3	Nuclear Receptor Binding SET Domain Protein 3	3.57408
EEF2K	Eukaryotic Elongation Factor 2 Kinase	3.57364
RPN2	Ribophorin II	3.57362
H2AC20	H2A Clustered Histone 20	3.57333
MTMR10	Myotubularin Related Protein 10	3.56915

SNRPN	Small Nuclear Ribonucleoprotein Polypeptide N	3.56835
GPX4	Glutathione Peroxidase 4	3.56481
SCG2	Secretogranin II	3.56453
TRH	Thyrotropin Releasing Hormone	3.56432
MSI2	Musashi RNA Binding Protein 2	3.56350
TNRC6C	Trinucleotide Repeat Containing Adaptor 6C	3.56293
CAPRIN2	Caprin Family Member 2	3.56241
SLC39A7	Solute Carrier Family 39 Member 7	3.55986
MIR101-2	MicroRNA 101-2	3.55862
COL6A1	Collagen Type VI Alpha 1 Chain	3.55858
CETP	Cholesteryl Ester Transfer Protein	3.55760
MIR421	MicroRNA 421	3.55722
RNASEH2A	Ribonuclease H2 Subunit A	3.55617
COL5A2	Collagen Type V Alpha 2 Chain	3.55614
SOCS2	Suppressor Of Cytokine Signaling 2	3.55471
CYB5R3	Cytochrome B5 Reductase 3	3.55270
CHRNA9	Cholinergic Receptor Nicotinic Alpha 9 Subunit	3.55260
RAB27A	RAB27A, Member RAS Oncogene Family	3.55240
LIMA1	LIM Domain And Actin Binding 1	3.55160
WDR45B	WD Repeat Domain 45B	3.55121
PDHA1	Pyruvate Dehydrogenase E1 Subunit Alpha 1	3.55098
FAF1	Fas Associated Factor 1	3.55091
SIK2	Salt Inducible Kinase 2	3.54758
RPS9	Ribosomal Protein S9	3.54671
MT-ND3	Mitochondrially Encoded NADH:Ubiquinone Oxidoreductase Core Subunit 3	3.54572
RNASE3	Ribonuclease A Family Member 3	3.54550
CUL7	Cullin 7	3.54510
VAV1	Vav Guanine Nucleotide Exchange Factor 1	3.54159
CTRL	Chymotrypsin Like	3.54142
STK19	Serine/Threonine Kinase 19	3.54113
ZDHHC7	Zinc Finger DHHC-Type Palmitoyltransferase 7	3.54050
ANXA7	Annexin A7	3.53849
KLHL7	Kelch Like Family Member 7	3.53794
GFPT1	Glutamine--Fructose-6-Phosphate Transaminase 1	3.53701
PTGDS	Prostaglandin D2 Synthase	3.53516
UNC13D	Unc-13 Homolog D	3.53402
ST6GALNAC1	ST6 N-Acetylgalactosaminide Alpha-2,6-Sialyltransferase 1	3.53354

PWP2	PWP2 Small Subunit Processome Component	3.53265
HTR1A	5-Hydroxytryptamine Receptor 1A	3.53030
CPQ	Carboxypeptidase Q	3.52798
ANK2	Ankyrin 2	3.52759
MYLK2	Myosin Light Chain Kinase 2	3.52624
MTA2	Metastasis Associated 1 Family Member 2	3.52602
EIF3M	Eukaryotic Translation Initiation Factor 3 Subunit M	3.52548
EHF	ETS Homologous Factor	3.52370
DDC	Dopa Decarboxylase	3.52102
LLGL2	LLGL Scribble Cell Polarity Complex Component 2	3.52097
ABCC8	ATP Binding Cassette Subfamily C Member 8	3.52054
KHDC4	KH Domain Containing 4, Pre-MRNA Splicing Factor	3.52041
INF2	Inverted Formin 2	3.51969
WHRN	Whirlin	3.51905
PMP22	Peripheral Myelin Protein 22	3.51841
ZNF563	Zinc Finger Protein 563	3.51654
C7orf50	Chromosome 7 Open Reading Frame 50	3.51297
FAAP100	FA Core Complex Associated Protein 100	3.51162
PHF8	PHD Finger Protein 8	3.51143
NMU	Neuromedin U	3.51088
LEPQTL1	Leptin, Serum Levels Of	3.50909
UFD1	Ubiquitin Recognition Factor In ER Associated Degradation 1	3.50908
MYO6	Myosin VI	3.50742
ZFPM1	Zinc Finger Protein, FOG Family Member 1	3.50730
NDP	Norrin Cystine Knot Growth Factor NDP	3.50725
GGA1	Golgi Associated, Gamma Adaptin Ear Containing, ARF Binding Protein 1	3.50681
IFRD2	Interferon Related Developmental Regulator 2	3.50616
PHF10	PHD Finger Protein 10	3.50455
ELK4	ETS Transcription Factor ELK4	3.50290
AMFR	Autocrine Motility Factor Receptor	3.50215
SNRPA	Small Nuclear Ribonucleoprotein Polypeptide A	3.50059
DCAF15	DDB1 And CUL4 Associated Factor 15	3.49866
HIP1	Huntingtin Interacting Protein 1	3.49764
SSR4	Signal Sequence Receptor Subunit 4	3.49684
DSTN	Destrin, Actin Depolymerizing Factor	3.49678

IRF2BPL	Interferon Regulatory Factor 2 Binding Protein Like	3.49607
BAG3	BAG Cochaperone 3	3.49523
STXBP1	Syntaxin Binding Protein 1	3.49450
LINC00672	Long Intergenic Non-Protein Coding RNA 672	3.49448
CD83	CD83 Molecule	3.49405
LMTK2	Lemur Tyrosine Kinase 2	3.49322
SPTB	Spectrin Beta, Erythrocytic	3.49282
HOXB7	Homeobox B7	3.49277
MFAP2	Microfibril Associated Protein 2	3.49198
HSPA2	Heat Shock Protein Family A (Hsp70) Member 2	3.49002
CAV2	Caveolin 2	3.48878
ETS1-AS1	ETS1 Antisense RNA 1	3.48863
SIGLEC5	Sialic Acid Binding Ig Like Lectin 5	3.48690
PLCD4	Phospholipase C Delta 4	3.48548
RAB40B	RAB40B, Member RAS Oncogene Family	3.48517
AIF1	Allograft Inflammatory Factor 1	3.48445
NID1	Nidogen 1	3.48435
TNPO3	Transportin 3	3.48326
MTSS1	MTSS I-BAR Domain Containing 1	3.48285
FKBP8	FKBP Prolyl Isomerase 8	3.48282
CSPG4	Chondroitin Sulfate Proteoglycan 4	3.48147
MYH3	Myosin Heavy Chain 3	3.47862
STK10	Serine/Threonine Kinase 10	3.47852
BRAP	BRCA1 Associated Protein	3.47725
MT-ND2	Mitochondrially Encoded NADH:Ubiquinone Oxidoreductase Core Subunit 2	3.47720
CDK10	Cyclin Dependent Kinase 10	3.47677
MAPK8IP1	Mitogen-Activated Protein Kinase 8 Interacting Protein 1	3.47590
SMIM24	Small Integral Membrane Protein 24	3.47347
DMAP1	DNA Methyltransferase 1 Associated Protein 1	3.47318
DACH1	Dachshund Family Transcription Factor 1	3.47219
ETV7	ETS Variant Transcription Factor 7	3.47136
PNOC	Prepronociceptin	3.47029
SCNN1B	Sodium Channel Epithelial 1 Subunit Beta	3.46966
CENPS	Centromere Protein S	3.46958
DNAH17	Dynein Axonemal Heavy Chain 17	3.46941
TONSL	Tonsoku Like, DNA Repair Protein	3.46835
TRIP12	Thyroid Hormone Receptor Interactor 12	3.46827
GALE	UDP-Galactose-4-Epimerase	3.46814

FOXH1	Forkhead Box H1	3.46777
LHPP	Phospholysine Phosphohistidine Inorganic Pyrophosphate Phosphatase	3.46744
U2AF1L4	U2 Small Nuclear RNA Auxiliary Factor 1 Like 4	3.46361
MOS	MOS Proto-Oncogene, Serine/Threonine Kinase	3.46315
PDCD5	Programmed Cell Death 5	3.46286
SEMA3C	Semaphorin 3C	3.46220
UGT2B7	UDP Glucuronosyltransferase Family 2 Member B7	3.46219
FSCN2	Fascin Actin-Bundling Protein 2, Retinal	3.46201
EDN3	Endothelin 3	3.45987
NPPB	Natriuretic Peptide B	3.45955
SLC22A23	Solute Carrier Family 22 Member 23	3.45948
CHD9	Chromodomain Helicase DNA Binding Protein 9	3.45834
SLCO2A1	Solute Carrier Organic Anion Transporter Family Member 2A1	3.45758
PAX1	Paired Box 1	3.45741
EVL	Enah/Vasp-Like	3.45714
SART3	Spliceosome Associated Factor 3, U4/U6 Recycling Protein	3.45705
RSPH1	Radial Spoke Head Component 1	3.45575
MUC3B	Mucin 3B, Cell Surface Associated	3.45530
MIR194-1	MicroRNA 194-1	3.45368
MTMR3	Myotubularin Related Protein 3	3.44925
SHARPIN	SHANK Associated RH Domain Interactor	3.44843
SSPN	Sarcospan	3.44712
H6PD	Hexose-6-Phosphate Dehydrogenase/Glucose 1-Dehydrogenase	3.44605
MAP3K7	Mitogen-Activated Protein Kinase Kinase Kinase 7	3.44569
DDX39A	DExD-Box Helicase 39A	3.44564
ZNF180	Zinc Finger Protein 180	3.44508
TNFRSF14	TNF Receptor Superfamily Member 14	3.44223
SLC3A2	Solute Carrier Family 3 Member 2	3.44062
NOXA1	NADPH Oxidase Activator 1	3.43753
MARCKS	Myristoylated Alanine Rich Protein Kinase C Substrate	3.43701
HUWE1	HECT, UBA And WWE Domain Containing E3 Ubiquitin Protein Ligase 1	3.43682
PHF6	PHD Finger Protein 6	3.43664
PLEKHA6	Pleckstrin Homology Domain Containing A6	3.43642
TDP2	Tyrosyl-DNA Phosphodiesterase 2	3.43416

LGALS2	Galectin 2	3.43329
NEPRO	Nucleolus And Neural Progenitor Protein	3.43188
MFGE8	Milk Fat Globule EGF And Factor V/VIII Domain Containing	3.43186
VANGL1	VANGL Planar Cell Polarity Protein 1	3.43160
ATP8B2	ATPase Phospholipid Transporting 8B2	3.43040
TAGLN2	Transgelin 2	3.43039
CST3	Cystatin C	3.42971
CLUAP1	Clusterin Associated Protein 1	3.42902
B3GAT1	Beta-1,3-Glucuronyltransferase 1	3.42883
PRPF3	Pre-MRNA Processing Factor 3	3.42581
LAMTOR5	Late Endosomal/Lysosomal Adaptor, MAPK And MTOR Activator 5	3.42537
PTBP1	Polypyrimidine Tract Binding Protein 1	3.42491
VPS52	VPS52 Subunit Of GARP Complex	3.42436
C12orf60	Chromosome 12 Open Reading Frame 60	3.42418
COL9A1	Collagen Type IX Alpha 1 Chain	3.42409
MPRIP	Myosin Phosphatase Rho Interacting Protein	3.42311
ZNF599	Zinc Finger Protein 599	3.42306
WWC1	WW And C2 Domain Containing 1	3.42303
GAD2	Glutamate Decarboxylase 2	3.42105
CCL19	C-C Motif Chemokine Ligand 19	3.41946
ALYREF	Aly/REF Export Factor	3.41926
RRBP1	Ribosome Binding Protein 1	3.41845
CSN1S1	Casein Alpha S1	3.41760
TXN2	Thioredoxin 2	3.41741
SETD1A	SET Domain Containing 1A, Histone Lysine Methyltransferase	3.41735
NEIL1	Nei Like DNA Glycosylase 1	3.41729
KDM4B	Lysine Demethylase 4B	3.41715
NBPF1	NBPF Member 1	3.41592
TET3	Tet Methylcytosine Dioxygenase 3	3.41571
FARSB	Phenylalanyl-TRNA Synthetase Subunit Beta	3.41417
RSPO2	R-Spondin 2	3.41356
HOXC6	Homeobox C6	3.41326
NIBAN2	Niban Apoptosis Regulator 2	3.41250
TOMM20	Translocase Of Outer Mitochondrial Membrane 20	3.41207
ABCD1	ATP Binding Cassette Subfamily D Member 1	3.41158
RFC5	Replication Factor C Subunit 5	3.41135
STAR	Steroidogenic Acute Regulatory Protein	3.41034
IER5L	Immediate Early Response 5 Like	3.40954
CCNC	Cyclin C	3.40927
GNAI1	G Protein Subunit Alpha I1	3.40628

AHNAK	AHNAK Nucleoprotein	3.40602
LPIN2	Lipin 2	3.40506
HNRNPH1	Heterogeneous Nuclear Ribonucleoprotein H1	3.40461
RBFOX2	RNA Binding Fox-1 Homolog 2	3.40290
JRK	Jrk Helix-Turn-Helix Protein	3.40235
PPP1R3A	Protein Phosphatase 1 Regulatory Subunit 3A	3.40183
RPL27A	Ribosomal Protein L27a	3.40112
MIR500A	MicroRNA 500a	3.39949
SURF4	Surfeit 4	3.39791
SYNGAP1	Synaptic Ras GTPase Activating Protein 1	3.39751
NLK	Nemo Like Kinase	3.39743
HGS	Hepatocyte Growth Factor-Regulated Tyrosine Kinase Substrate	3.39496
KCNJ10	Potassium Inwardly Rectifying Channel Subfamily J Member 10	3.39353
MEF2C	Myocyte Enhancer Factor 2C	3.39219
FBXO4	F-Box Protein 4	3.39203
FXYD3	FXYD Domain Containing Ion Transport Regulator 3	3.39123
PDHX	Pyruvate Dehydrogenase Complex Component X	3.39041
ACO2	Aconitase 2	3.38962
MAML3	Mastermind Like Transcriptional Coactivator 3	3.38724
TRAF3IP2	TRAF3 Interacting Protein 2	3.38604
YWHAB	Tyrosine 3-Monooxygenase/Tryptophan 5-Monooxygenase Activation Protein Beta	3.38571
EMC1	ER Membrane Protein Complex Subunit 1	3.38420
BRD9	Bromodomain Containing 9	3.38362
CCNT1	Cyclin T1	3.38145
DUSP13	Dual Specificity Phosphatase 13	3.38071
IQCB1	IQ Motif Containing B1	3.37977
TBX21	T-Box Transcription Factor 21	3.37954
PSPN	Persephin	3.37914
PLA2G7	Phospholipase A2 Group VII	3.37902
CFP	Complement Factor Properdin	3.37813
LGMN	Legumain	3.37812
GPN1	GPN-Loop GTPase 1	3.37788
DCST1	DC-STAMP Domain Containing 1	3.37726
KRTAP5-AS1	KRTAP5-1/KRTAP5-2 Antisense RNA 1	3.37708
RACGAP1	Rac GTPase Activating Protein 1	3.37692
MIR302D	MicroRNA 302d	3.37584
BBS1	Bardet-Biedl Syndrome 1	3.37582

ERF	ETS2 Repressor Factor	3.37507
KIF11	Kinesin Family Member 11	3.37443
RAB14	RAB14, Member RAS Oncogene Family	3.37346
BTNL2	Butyrophilin Like 2	3.37340
ARPC2	Actin Related Protein 2/3 Complex Subunit 2	3.37281
HOXA1	Homeobox A1	3.37027
DNAJC2	DnaJ Heat Shock Protein Family (Hsp40) Member C2	3.37017
TENT5C	Terminal Nucleotidyltransferase 5C	3.37008
KRT19P3	Keratin 19 Pseudogene 3	3.37007
GPD2	Glycerol-3-Phosphate Dehydrogenase 2	3.36908
LATS2	Large Tumor Suppressor Kinase 2	3.36710
PRSS58	Serine Protease 58	3.36685
FAM107B	Family With Sequence Similarity 107 Member B	3.36629
TSPAN8	Tetraspanin 8	3.36599
NONO	Non-POU Domain Containing Octamer Binding	3.36482
CLDN10	Claudin 10	3.36482
EPRS1	Glutamyl-Prolyl-TRNA Synthetase 1	3.36387
RPL12	Ribosomal Protein L12	3.36317
ATN1	Atrophin 1	3.36258
PREX2	Phosphatidylinositol-3,4,5-Trisphosphate Dependent Rac Exchange Factor 2	3.36166
NUP133	Nucleoporin 133	3.36011
IL13RA1	Interleukin 13 Receptor Subunit Alpha 1	3.35698
NBL1	NBL1, DAN Family BMP Antagonist	3.35673
PTPRS	Protein Tyrosine Phosphatase Receptor Type S	3.35605
FDFT1	Farnesyl-Diphosphate Farnesyltransferase 1	3.35547
SOD3	Superoxide Dismutase 3	3.35357
ZNF318	Zinc Finger Protein 318	3.35304
KIF2A	Kinesin Family Member 2A	3.35239
NUP188	Nucleoporin 188	3.35135
TBCD	Tubulin Folding Cofactor D	3.35030
GAB2	GRB2 Associated Binding Protein 2	3.34989
NT5C2	5'-Nucleotidase, Cytosolic II	3.34876
PLS3	Plastin 3	3.34850
TRMT2A	TRNA Methyltransferase 2 Homolog A	3.34810
CRHR1	Corticotropin Releasing Hormone Receptor 1	3.34809
CRABP2	Cellular Retinoic Acid Binding Protein 2	3.34691
SRSF5	Serine And Arginine Rich Splicing Factor 5	3.34626

CEMIP2	Cell Migration Inducing Hyaluronidase 2	3.34597
TDG	Thymine DNA Glycosylase	3.34508
CDC45	Cell Division Cycle 45	3.34284
HPSE2	Heparanase 2 (Inactive)	3.34265
H1-4	H1.4 Linker Histone, Cluster Member	3.34251
RCC2	Regulator Of Chromosome Condensation 2	3.34207
APOA4	Apolipoprotein A4	3.33985
RBPMS	RNA Binding Protein, MRNA Processing Factor	3.33847
MAPKAPK5	MAPK Activated Protein Kinase 5	3.33784
CASP4	Caspase 4	3.33687
RGS3	Regulator Of G Protein Signaling 3	3.33613
RBBP5	RB Binding Protein 5, Histone Lysine Methyltransferase Complex Subunit	3.33547
KIAA1671	KIAA1671	3.33542
CHAC1	ChaC Glutathione Specific Gamma-Glutamylcyclotransferase 1	3.33538
PPP2CA	Protein Phosphatase 2 Catalytic Subunit Alpha	3.33532
SCRIB	Scribble Planar Cell Polarity Protein	3.33303
ZNF331	Zinc Finger Protein 331	3.33263
MYL3	Myosin Light Chain 3	3.33214
DCTN1	Dynactin Subunit 1	3.33206
CTH	Cystathionine Gamma-Lyase	3.33189
SLC39A11	Solute Carrier Family 39 Member 11	3.33005
DDX6	DEAD-Box Helicase 6	3.32793
DDX17	DEAD-Box Helicase 17	3.32702
DPPA2	Developmental Pluripotency Associated 2	3.32677
EIF2S1	Eukaryotic Translation Initiation Factor 2 Subunit Alpha	3.32657
ITPKC	Inositol-Trisphosphate 3-Kinase C	3.32648
FOXL1	Forkhead Box L1	3.32618
HM13	Histocompatibility Minor 13	3.32582
RMI2	RecQ Mediated Genome Instability 2	3.32521
SEMA4D	Semaphorin 4D	3.32446
BLOC1S5	Biogenesis Of Lysosomal Organelles Complex 1 Subunit 5	3.32431
PRPF4B	Pre-MRNA Processing Factor 4B	3.32320
CEP89	Centrosomal Protein 89	3.32302
POLM	DNA Polymerase Mu	3.32238
IRX5	Iroquois Homeobox 5	3.32103
SLC66A1	Solute Carrier Family 66 Member 1	3.32100
MAZ	MYC Associated Zinc Finger Protein	3.32055
PRKACG	Protein Kinase CAMP-Activated Catalytic Subunit Gamma	3.32041
PLAC1	Placenta Enriched 1	3.31881

MT-RNR1	Mitochondrially Encoded 12S RRNA	3.31870
CNBP	CCHC-Type Zinc Finger Nucleic Acid Binding Protein	3.31736
FOXF2	Forkhead Box F2	3.31717
PUM2	Pumilio RNA Binding Family Member 2	3.31717
PDCD6	Programmed Cell Death 6	3.31670
SLC39A4	Solute Carrier Family 39 Member 4	3.31656
FAU	FAU Ubiquitin Like And Ribosomal Protein S30 Fusion	3.31642
CTIF	Cap Binding Complex Dependent Translation Initiation Factor	3.31598
MAEL	Maelstrom Spermatogenic Transposon Silencer	3.31496
MT-TS1	Mitochondrially Encoded TRNA-Ser (UCN) 1	3.31434
DNAJA3	DnaJ Heat Shock Protein Family (Hsp40) Member A3	3.31208
ARHGDIB	Rho GDP Dissociation Inhibitor Beta	3.31176
GLIS2	GLIS Family Zinc Finger 2	3.30956
RPL10	Ribosomal Protein L10	3.30887
PDE4A	Phosphodiesterase 4A	3.30806
TST	Thiosulfate Sulfurtransferase	3.30784
GART	Phosphoribosylglycinamide Formyltransferase, Phosphoribosylglycinamide Synthetase, Phosphoribosylaminoimidazole Synthetase	3.30768
ERP29	Endoplasmic Reticulum Protein 29	3.30640
EPM2AIP1	EPM2A Interacting Protein 1	3.30498
GBAP1	Glucosylceramidase Beta Pseudogene 1	3.30489
GGH	Gamma-Glutamyl Hydrolase	3.30210
PPIL3	Peptidylprolyl Isomerase Like 3	3.30200
BRINP3	BMP/Retinoic Acid Inducible Neural Specific 3	3.30057
RAP1GAP2	RAP1 GTPase Activating Protein 2	3.30002
RORA	RAR Related Orphan Receptor A	3.29847
GNG10	G Protein Subunit Gamma 10	3.29655
ADRA1B	Adrenoceptor Alpha 1B	3.29626
PIEZO1	Piezo Type Mechanosensitive Ion Channel Component 1	3.29616
EPHA10	EPH Receptor A10	3.29510
ZC3H18	Zinc Finger CCCH-Type Containing 18	3.29492
TP53I3	Tumor Protein P53 Inducible Protein 3	3.29482
ADAM19	ADAM Metallopeptidase Domain 19	3.29423
ZKSCAN3	Zinc Finger With KRAB And SCAN Domains 3	3.29409
CEBPE	CCAAT Enhancer Binding Protein Epsilon	3.29408

MMACHC	Metabolism Of Cobalamin Associated C	3.29397
SH3BP4	SH3 Domain Binding Protein 4	3.29319
OXA1L	OXA1L Mitochondrial Inner Membrane Protein	3.29304
ARF1	ADP Ribosylation Factor 1	3.29255
FOSL2	FOS Like 2, AP-1 Transcription Factor Subunit	3.29183
UBQLN4	Ubiquilin 4	3.29140
HLA-F	Major Histocompatibility Complex, Class I, F	3.29112
RAB35	RAB35, Member RAS Oncogene Family	3.29099
LOC10602931[illegible]	Williams-Beuren Syndrome Medial Block B Recombination Region	3.28994
CLCNKB	Chloride Voltage-Gated Channel Kb	3.28639
CBX7	Chromobox 7	3.28614
LINC00115	Long Intergenic Non-Protein Coding RNA 115	3.28420
DDX39B	DExD-Box Helicase 39B	3.28378
YWHAG	Tyrosine 3-Monooxygenase/Tryptophan 5-Monooxygenase Activation Protein Gamma	3.28311
LINC01273	Long Intergenic Non-Protein Coding RNA 1273	3.28188
TRIM21	Tripartite Motif Containing 21	3.28164
CDH4	Cadherin 4	3.28159
GRAMD1A	GRAM Domain Containing 1A	3.28056
TTC8	Tetratricopeptide Repeat Domain 8	3.28001
P2RY2	Purinergic Receptor P2Y2	3.27881
USP40	Ubiquitin Specific Peptidase 40	3.27732
CD47	CD47 Molecule	3.27715
UNC5C	Unc-5 Netrin Receptor C	3.27634
UBA52	Ubiquitin A-52 Residue Ribosomal Protein Fusion Product 1	3.27538
SFXN4	Sideroflexin 4	3.27512
SLC17A9	Solute Carrier Family 17 Member 9	3.27506
SLC26A6	Solute Carrier Family 26 Member 6	3.27324
LUM	Lumican	3.27315
GJB6	Gap Junction Protein Beta 6	3.27299
CNGB1	Cyclic Nucleotide Gated Channel Subunit Beta 1	3.27249
RPS21	Ribosomal Protein S21	3.27235
SLC2A10	Solute Carrier Family 2 Member 10	3.27211
LTBP2	Latent Transforming Growth Factor Beta Binding Protein 2	3.27047
SLCO2B1	Solute Carrier Organic Anion Transporter Family Member 2B1	3.26979
PABPN1	Poly(A) Binding Protein Nuclear 1	3.26927

FOXN3	Forkhead Box N3	3.26924
COL9A3	Collagen Type IX Alpha 3 Chain	3.26920
NCR2	Natural Cytotoxicity Triggering Receptor 2	3.26884
LDHC	Lactate Dehydrogenase C	3.26869
LZTFL1	Leucine Zipper Transcription Factor Like 1	3.26781
ZRANB1	Zinc Finger RANBP2-Type Containing 1	3.26638
ARVCF	ARVCF Delta Catenin Family Member	3.26586
DPM3	Dolichyl-Phosphate Mannosyltransferase Subunit 3, Regulatory	3.26555
MTX1	Metaxin 1	3.26341
DGCR8	DGCR8 Microprocessor Complex Subunit	3.26175
GNMT	Glycine N-Methyltransferase	3.25962
COL10A1	Collagen Type X Alpha 1 Chain	3.25918
FKBP1A	FKBP Prolyl Isomerase 1A	3.25900
AMT	Aminomethyltransferase	3.25876
C19orf33	Chromosome 19 Open Reading Frame 33	3.25827
ALPK2	Alpha Kinase 2	3.25686
RETREG1	Reticulophagy Regulator 1	3.25630
SOX13	SRY-Box Transcription Factor 13	3.25571
INPP5D	Inositol Polyphosphate-5-Phosphatase D	3.25525
BGN	Biglycan	3.25451
PLA2G3	Phospholipase A2 Group III	3.25391
GLOD4	Glyoxalase Domain Containing 4	3.25058
CPNE1	Copine 1	3.25056
WDR4	WD Repeat Domain 4	3.24856
NDUFAF6	NADH:Ubiquinone Oxidoreductase Complex Assembly Factor 6	3.24829
TXNDC15	Thioredoxin Domain Containing 15	3.24814
SPATA18	Spermatogenesis Associated 18	3.24714
PSMG1	Proteasome Assembly Chaperone 1	3.24652
ZNF442	Zinc Finger Protein 442	3.24616
ZNF655	Zinc Finger Protein 655	3.24584
YWHAQ	Tyrosine 3-Monooxygenase/Tryptophan 5-Monooxygenase Activation Protein Theta	3.24547
SMAD5	SMAD Family Member 5	3.24540
CXCL16	C-X-C Motif Chemokine Ligand 16	3.24455
RBPMS-AS1	RBPMS Antisense RNA 1	3.24394
MIR654	MicroRNA 654	3.24319
CAPN3	Calpain 3	3.24096
CLCNKA	Chloride Voltage-Gated Channel Ka	3.24055
ESS2	Ess-2 Splicing Factor Homolog	3.24051

BCL9L	BCL9 Like	3.24023
FRAT2	FRAT Regulator Of WNT Signaling Pathway 2	3.24007
NDST1	N-Deacetylase And N-Sulfotransferase 1	3.23991
DSPP	Dentin Sialophosphoprotein	3.23926
BMF	Bcl2 Modifying Factor	3.23839
DAPK3	Death Associated Protein Kinase 3	3.23673
S100A10	S100 Calcium Binding Protein A10	3.23577
HADHA	Hydroxyacyl-CoA Dehydrogenase Trifunctional Multienzyme Complex Subunit Alpha	3.23486
RIPOR3	RIPOR Family Member 3	3.23441
PCSK1	Proprotein Convertase Subtilisin/Kexin Type 1	3.23435
TUT1	Terminal Uridylyl Transferase 1, U6 SnRNA-Specific	3.23249
ADRM1	ADRM1 26S Proteasome Ubiquitin Receptor	3.23213
PKN1	Protein Kinase N1	3.23109
SCAMP3	Secretory Carrier Membrane Protein 3	3.23103
GNB2	G Protein Subunit Beta 2	3.23060
COIL	Coilin	3.23047
HTRA2	HtrA Serine Peptidase 2	3.23044
EIF4G3	Eukaryotic Translation Initiation Factor 4 Gamma 3	3.22982
RPA3	Replication Protein A3	3.22929
ST7	Suppression Of Tumorigenicity 7	3.22816
BAMBI	BMP And Activin Membrane Bound Inhibitor	3.22771
DDX27	DEAD-Box Helicase 27	3.22700
ADSL	Adenylosuccinate Lyase	3.22680
H3C3	H3 Clustered Histone 3	3.22615
TIMM22	Translocase Of Inner Mitochondrial Membrane 22	3.22612
ITGA1	Integrin Subunit Alpha 1	3.22611
SLC12A3	Solute Carrier Family 12 Member 3	3.22582
ARL3	ADP Ribosylation Factor Like GTPase 3	3.22578
H4C1	H4 Clustered Histone 1	3.22569
DUOX1	Dual Oxidase 1	3.22510
PLCB2	Phospholipase C Beta 2	3.22504
KAT8	Lysine Acetyltransferase 8	3.22388
TREM2	Triggering Receptor Expressed On Myeloid Cells 2	3.22381
PREX1	Phosphatidylinositol-3,4,5-Trisphosphate Dependent Rac Exchange Factor 1	3.22364
ARPC1B	Actin Related Protein 2/3 Complex Subunit 1B	3.22363
FAT2	FAT Atypical Cadherin 2	3.22195

TMEM43	Transmembrane Protein 43	3.22083
ARHGDIA	Rho GDP Dissociation Inhibitor Alpha	3.21987
EPHX2	Epoxide Hydrolase 2	3.21939
TRAPPC10	Trafficking Protein Particle Complex Subunit 10	3.21877
TFAP4	Transcription Factor AP-4	3.21832
BRI3	Brain Protein I3	3.21831
OXTR	Oxytocin Receptor	3.21764
AGO1	Argonaute RISC Component 1	3.21717
SPAG1	Sperm Associated Antigen 1	3.21599
PTX3	Pentraxin 3	3.21582
C14orf93	Chromosome 14 Open Reading Frame 93	3.21553
PDLIM5	PDZ And LIM Domain 5	3.21546
A2M	Alpha-2-Macroglobulin	3.21412
IGHE	Immunoglobulin Heavy Constant Epsilon	3.21331
MANBAL	Mannosidase Beta Like	3.21223
MCM5	Minichromosome Maintenance Complex Component 5	3.21148
PPP4R3A	Protein Phosphatase 4 Regulatory Subunit 3A	3.20950
PPP1R14A	Protein Phosphatase 1 Regulatory Inhibitor Subunit 14A	3.20937
CCHCR1	Coiled-Coil Alpha-Helical Rod Protein 1	3.20852
KDM4A	Lysine Demethylase 4A	3.20829
MYO9B	Myosin IXB	3.20779
TTC36	Tetratricopeptide Repeat Domain 36	3.20732
ZNF155	Zinc Finger Protein 155	3.20711
CRYBG2	Crystallin Beta-Gamma Domain Containing 2	3.20692
SERF2	Small EDRK-Rich Factor 2	3.20686
DTL	Denticleless E3 Ubiquitin Protein Ligase Homolog	3.20630
YWHAH	Tyrosine 3-Monooxygenase/Tryptophan 5-Monooxygenase Activation Protein Eta	3.20467
OGT	O-Linked N-Acetylglucosamine (GlcNAc) Transferase	3.20269
FBLN5	Fibulin 5	3.20249
FOLR2	Folate Receptor Beta	3.20219
TCF20	Transcription Factor 20	3.20086
LPCAT1	Lysophosphatidylcholine Acyltransferase 1	3.20082
EPN3	Epsin 3	3.20058
ABL2	ABL Proto-Oncogene 2, Non-Receptor Tyrosine Kinase	3.20041
CIT	Citron Rho-Interacting Serine/Threonine Kinase	3.19813

CD58	CD58 Molecule	3.19615
MICAL2	Microtubule Associated Monooxygenase, Calponin And LIM Domain Containing 2	3.19553
LRRFIP1	LRR Binding FLII Interacting Protein 1	3.19540
NKD2	NKD Inhibitor Of WNT Signaling Pathway 2	3.19449
EMILIN2	Elastin Microfibril Interfacer 2	3.19335
CHD2	Chromodomain Helicase DNA Binding Protein 2	3.19239
CD177	CD177 Molecule	3.19143
CDC5L	Cell Division Cycle 5 Like	3.19136
HMGB2	High Mobility Group Box 2	3.19077
MCRS1	Microspherule Protein 1	3.18962
ULK1	Unc-51 Like Autophagy Activating Kinase 1	3.18874
CAPNS1	Calpain Small Subunit 1	3.18798
CHGB	Chromogranin B	3.18729
LALBA	Lactalbumin Alpha	3.18571
GOLM1	Golgi Membrane Protein 1	3.18477
MYH1	Myosin Heavy Chain 1	3.18463
HECTD4	HECT Domain E3 Ubiquitin Protein Ligase 4	3.18350
NXN	Nucleoredoxin	3.18017
HLA-DRA	Major Histocompatibility Complex, Class II, DR Alpha	3.17923
CTSH	Cathepsin H	3.17835
CNTN1	Contactin 1	3.17666
TM4SF5	Transmembrane 4 L Six Family Member 5	3.17479
PSMD9	Proteasome 26S Subunit, Non-ATPase 9	3.17469
MYO1C	Myosin IC	3.17449
PMVK	Phosphomevalonate Kinase	3.17409
RPL3	Ribosomal Protein L3	3.17392
PPP1R10	Protein Phosphatase 1 Regulatory Subunit 10	3.17076
EGR2	Early Growth Response 2	3.17027
EEF1G	Eukaryotic Translation Elongation Factor 1 Gamma	3.16959
RPLP1	Ribosomal Protein Lateral Stalk Subunit P1	3.16949
PTCD3	Pentatricopeptide Repeat Domain 3	3.16840
PHC3	Polyhomeotic Homolog 3	3.16816
PLCB3	Phospholipase C Beta 3	3.16777
CARTPT	CART Prepropeptide	3.16769
AFF4	AF4/FMR2 Family Member 4	3.16692
CUX2	Cut Like Homeobox 2	3.16659
GNAT1	G Protein Subunit Alpha Transducin 1	3.16574

ABCB7	ATP Binding Cassette Subfamily B Member 7	3.16536
PEX14	Peroxisomal Biogenesis Factor 14	3.16482
OLR1	Oxidized Low Density Lipoprotein Receptor 1	3.16388
TULP2	TUB Like Protein 2	3.16366
PDXK	Pyridoxal Kinase	3.16352
EIF6	Eukaryotic Translation Initiation Factor 6	3.16309
STX10	Syntaxin 10	3.16281
MGRN1	Mahogunin Ring Finger 1	3.16166
BNIP3L	BCL2 Interacting Protein 3 Like	3.16114
PRRT2	Proline Rich Transmembrane Protein 2	3.16072
PHC1	Polyhomeotic Homolog 1	3.16067
EIF3I	Eukaryotic Translation Initiation Factor 3 Subunit I	3.16046
PPIG	Peptidylprolyl Isomerase G	3.16006
EMD	Emerin	3.15971
KDM2A	Lysine Demethylase 2A	3.15859
RIN2	Ras And Rab Interactor 2	3.15805
RBM3	RNA Binding Motif Protein 3	3.15795
APOL2	Apolipoprotein L2	3.15791
RPL13	Ribosomal Protein L13	3.15705
AIFM2	Apoptosis Inducing Factor Mitochondria Associated 2	3.15584
SGCA	Sarcoglycan Alpha	3.15518
VILL	Villin Like	3.15439
PTP4A2	Protein Tyrosine Phosphatase 4A2	3.15415
TRIM31	Tripartite Motif Containing 31	3.15363
ADGRG6	Adhesion G Protein-Coupled Receptor G6	3.15216
GALNT2	Polypeptide N-Acetylgalactosaminyltransferase 2	3.15159
G3BP1	G3BP Stress Granule Assembly Factor 1	3.15124
DHPS	Deoxyhypusine Synthase	3.14940
TELO2	Telomere Maintenance 2	3.14931
PPOX	Protoporphyrinogen Oxidase	3.14908
FCGBP	Fc Fragment Of IgG Binding Protein	3.14908
DNMT3L	DNA Methyltransferase 3 Like	3.14820
ALKBH5	AlkB Homolog 5, RNA Demethylase	3.14732
CSNK2A2	Casein Kinase 2 Alpha 2	3.14721
SSRP1	Structure Specific Recognition Protein 1	3.14711
PDE6A	Phosphodiesterase 6A	3.14694
MT-TK	Mitochondrially Encoded TRNA-Lys (AAA/G)	3.14375
TOPORS	TOP1 Binding Arginine/Serine Rich Protein, E3 Ubiquitin Ligase	3.14292
RPL17	Ribosomal Protein L17	3.14268
TSPAN4	Tetraspanin 4	3.14203

SSR1	Signal Sequence Receptor Subunit 1	3.14158
CTDSPL	CTD Small Phosphatase Like	3.14104
CORO1C	Coronin 1C	3.14038
BHLHA15	Basic Helix-Loop-Helix Family Member A15	3.13967
GSTT2	Glutathione S-Transferase Theta 2 (Gene/Pseudogene)	3.13940
ABCC10	ATP Binding Cassette Subfamily C Member 10	3.13842
ANKHD1	Ankyrin Repeat And KH Domain Containing 1	3.13817
OSMR	Oncostatin M Receptor	3.13801
PPM1F	Protein Phosphatase, Mg2+/Mn2+ Dependent 1F	3.13614
RNPEP	Arginyl Aminopeptidase	3.13595
KCNJ1	Potassium Inwardly Rectifying Channel Subfamily J Member 1	3.13522
MUC15	Mucin 15, Cell Surface Associated	3.13514
SIAH2	Siah E3 Ubiquitin Protein Ligase 2	3.13304
SPATA20	Spermatogenesis Associated 20	3.13245
FNIP1	Folliculin Interacting Protein 1	3.13123
C17orf80	Chromosome 17 Open Reading Frame 80	3.13109
FOSB	FosB Proto-Oncogene, AP-1 Transcription Factor Subunit	3.12888
CETN2	Centrin 2	3.12885
CBARP	CACN Subunit Beta Associated Regulatory Protein	3.12885
SERPINE2	Serpin Family E Member 2	3.12620
TRAPPC2L	Trafficking Protein Particle Complex Subunit 2L	3.12571
PCBP4	Poly(RC) Binding Protein 4	3.12498
AKAP8	A-Kinase Anchoring Protein 8	3.12464
VLDLR	Very Low Density Lipoprotein Receptor	3.12417
HS1BP3	HCLS1 Binding Protein 3	3.12310
UBE2V2	Ubiquitin Conjugating Enzyme E2 V2	3.12276
ITGB6	Integrin Subunit Beta 6	3.12216
VPS37A	VPS37A Subunit Of ESCRT-I	3.12178
OIP5-AS1	OIP5 Antisense RNA 1	3.11986
POFUT1	Protein O-Fucosyltransferase 1	3.11962
SERPINB9	Serpin Family B Member 9	3.11896
FKTN	Fukutin	3.11853
RASAL1	RAS Protein Activator Like 1	3.11797
BANF1	BAF Nuclear Assembly Factor 1	3.11759
TMED1	Transmembrane P24 Trafficking Protein 1	3.11677
PSMA5	Proteasome 20S Subunit Alpha 5	3.11452
BCAM	Basal Cell Adhesion Molecule (Lutheran Blood Group)	3.11375

TTC12	Tetratricopeptide Repeat Domain 12	3.11340
ACKR4	Atypical Chemokine Receptor 4	3.11328
APLNR	Apelin Receptor	3.11222
UGT1A3	UDP Glucuronosyltransferase Family 1 Member A3	3.11145
SLX1A	SLX1 Homolog A, Structure-Specific Endonuclease Subunit	3.11114
PFKP	Phosphofructokinase, Platelet	3.11016
UBE2N	Ubiquitin Conjugating Enzyme E2 N	3.10945
MT-ND4	Mitochondrially Encoded NADH:Ubiquinone Oxidoreductase Core Subunit 4	3.10914
OGDH	Oxoglutarate Dehydrogenase	3.10909
EBF1	EBF Transcription Factor 1	3.10820
EMG1	EMG1 N1-Specific Pseudouridine Methyltransferase	3.10804
CACNA1A	Calcium Voltage-Gated Channel Subunit Alpha1 A	3.10769
HPD	4-Hydroxyphenylpyruvate Dioxygenase	3.10723
MINCR	MYC-Induced Long Non-Coding RNA	3.10702
LOC11028362	SPP1 5' Regulatory Region	3.10599
BTD	Biotinidase	3.10536
IARS2	Isoleucyl-TRNA Synthetase 2, Mitochondrial	3.10484
ZNF521	Zinc Finger Protein 521	3.10383
PCSK2	Proprotein Convertase Subtilisin/Kexin Type 2	3.10356
MPZ	Myelin Protein Zero	3.10259
IL11RA	Interleukin 11 Receptor Subunit Alpha	3.10247
THRAP3	Thyroid Hormone Receptor Associated Protein 3	3.10217
PCDH12	Protocadherin 12	3.10122
PRG2	Proteoglycan 2, Pro Eosinophil Major Basic Protein	3.10109
BBS9	Bardet-Biedl Syndrome 9	3.09950
CRTC3	CREB Regulated Transcription Coactivator 3	3.09921
NFKBIL1	NFKB Inhibitor Like 1	3.09905
FNDC11	Fibronectin Type III Domain Containing 11	3.09867
HOXD10	Homeobox D10	3.09844
MARCKSL1	MARCKS Like 1	3.09754
VASH1	Vasohibin 1	3.09718
GAL3ST1	Galactose-3-O-Sulfotransferase 1	3.09700
LIPE	Lipase E, Hormone Sensitive Type	3.09690
OPA3	Outer Mitochondrial Membrane Lipid Metabolism Regulator OPA3	3.09449

MGAT3	Beta-1,4-Mannosyl-Glycoprotein 4-Beta-N-Acetylglucosaminyltransferase	3.09260
CMA1	Chymase 1	3.09242
AATK	Apoptosis Associated Tyrosine Kinase	3.08998
MAP2K7	Mitogen-Activated Protein Kinase Kinase 7	3.08929
DBN1	Drebrin 1	3.08909
RFX6	Regulatory Factor X6	3.08688
CAMK2B	Calcium/Calmodulin Dependent Protein Kinase II Beta	3.08657
FBXO6	F-Box Protein 6	3.08637
SCD	Stearoyl-CoA Desaturase	3.08604
SLC26A9	Solute Carrier Family 26 Member 9	3.08504
CBR1	Carbonyl Reductase 1	3.08504
ATP1A2	ATPase Na+/K+ Transporting Subunit Alpha 2	3.08437
LINC00665	Long Intergenic Non-Protein Coding RNA 665	3.08368
PI3	Peptidase Inhibitor 3	3.08355
ENSG00000271267		3.08338
SLC12A7	Solute Carrier Family 12 Member 7	3.08316
MRGBP	MRG Domain Binding Protein	3.08311
RUVBL1	RuvB Like AAA ATPase 1	3.08257
CAST	Calpastatin	3.08165
AP1M1	Adaptor Related Protein Complex 1 Subunit Mu 1	3.08139
UCP1	Uncoupling Protein 1	3.08066
TRAF3IP2-AS1	TRAF3IP2 Antisense RNA 1	3.08005
MCU	Mitochondrial Calcium Uniporter	3.07902
MRC1	Mannose Receptor C-Type 1	3.07808
PEDS1	Plasmanylethanolamine Desaturase 1	3.07742
EPB41L1	Erythrocyte Membrane Protein Band 4.1 Like 1	3.07712
ABCB6	ATP Binding Cassette Subfamily B Member 6 (Langereis Blood Group)	3.07672
SLC44A2	Solute Carrier Family 44 Member 2	3.07658
CACNA1C	Calcium Voltage-Gated Channel Subunit Alpha1 C	3.07578
CERCAM	Cerebral Endothelial Cell Adhesion Molecule	3.07543
STXBP2	Syntaxin Binding Protein 2	3.07455
CAD	Carbamoyl-Phosphate Synthetase 2, Aspartate Transcarbamylase, And Dihydroorotase	3.07334
PRPSAP1	Phosphoribosyl Pyrophosphate Synthetase Associated Protein 1	3.07302
RTN4	Reticulon 4	3.07196
DEPTOR	DEP Domain Containing MTOR Interacting Protein	3.07189

CENPA	Centromere Protein A	3.07144
BAIAP2	BAR/IMD Domain Containing Adaptor Protein 2	3.06931
WDR62	WD Repeat Domain 62	3.06799
NANOGP8	Nanog Homeobox Retrogene P8	3.06737
GPC1	Glypican 1	3.06643
TPCN2	Two Pore Segment Channel 2	3.06643
PTPRB	Protein Tyrosine Phosphatase Receptor Type B	3.06562
CPSF1	Cleavage And Polyadenylation Specific Factor 1	3.06558
CTNS	Cystinosin, Lysosomal Cystine Transporter	3.06535
MAG	Myelin Associated Glycoprotein	3.06350
RBCK1	RANBP2-Type And C3HC4-Type Zinc Finger Containing 1	3.06263
ARFGAP2	ADP Ribosylation Factor GTPase Activating Protein 2	3.06256
CALCR	Calcitonin Receptor	3.06251
FOXK1	Forkhead Box K1	3.06233
SPAST	Spastin	3.05960
DAZAP1	DAZ Associated Protein 1	3.05723
SMARCA5	SWI/SNF Related, Matrix Associated, Actin Dependent Regulator Of Chromatin, Subfamily A, Member 5	3.05494
ESCO2	Establishment Of Sister Chromatid Cohesion N-Acetyltransferase 2	3.05490
CCR10	C-C Motif Chemokine Receptor 10	3.05410
KCNMA1	Potassium Calcium-Activated Channel Subfamily M Alpha 1	3.05289
ADH5	Alcohol Dehydrogenase 5 (Class III), Chi Polypeptide	3.05136
ATF6	Activating Transcription Factor 6	3.05022
COL11A2	Collagen Type XI Alpha 2 Chain	3.05019
LEMD1	LEM Domain Containing 1	3.05014
KXD1	KxDL Motif Containing 1	3.04900
DCTN4	Dynactin Subunit 4	3.04817
SERPINF2	Serpin Family F Member 2	3.04769
SLC6A14	Solute Carrier Family 6 Member 14	3.04640
IK	IK Cytokine	3.04567
ENSG00000232043		3.04471
LAMA1	Laminin Subunit Alpha 1	3.04453
CALCRL	Calcitonin Receptor Like Receptor	3.04366
PIF1	PIF1 5'-To-3' DNA Helicase	3.04348
FAM53B	Family With Sequence Similarity 53 Member B	3.04340
PABPC1	Poly(A) Binding Protein Cytoplasmic 1	3.04289
SGSM3	Small G Protein Signaling Modulator 3	3.04226
CFAP77	Cilia And Flagella Associated Protein 77	3.04218

ARID4A	AT-Rich Interaction Domain 4A	3.04102
PHB2	Prohibitin 2	3.04082
FAM107A	Family With Sequence Similarity 107 Member A	3.04012
TEAD4	TEA Domain Transcription Factor 4	3.03948
CDC26	Cell Division Cycle 26	3.03895
MAN1B1	Mannosidase Alpha Class 1B Member 1	3.03882
IL17RA	Interleukin 17 Receptor A	3.03861
ZBTB17	Zinc Finger And BTB Domain Containing 17	3.03806
PFKFB2	6-Phosphofructo-2-Kinase/Fructose-2,6-Biphosphatase 2	3.03802
MIR369	MicroRNA 369	3.03762
NEUROD1	Neuronal Differentiation 1	3.03749
CAVIN1	Caveolae Associated Protein 1	3.03672
SLC7A11	Solute Carrier Family 7 Member 11	3.03571
COX4I1	Cytochrome C Oxidase Subunit 4I1	3.03570
ALDH1A3	Aldehyde Dehydrogenase 1 Family Member A3	3.03340
LIN9	Lin-9 DREAM MuvB Core Complex Component	3.03272
ARL2BP	ADP Ribosylation Factor Like GTPase 2 Binding Protein	3.03235
TRIM63	Tripartite Motif Containing 63	3.03129
CDK5RAP3	CDK5 Regulatory Subunit Associated Protein 3	3.03095
LMNB1	Lamin B1	3.03076
LEMD3	LEM Domain Containing 3	3.03059
FERMT2	FERM Domain Containing Kindlin 2	3.03054
ALG11	ALG11 Alpha-1,2-Mannosyltransferase	3.03003
HK3	Hexokinase 3	3.02986
PEX5	Peroxisomal Biogenesis Factor 5	3.02970
PDS5B	PDS5 Cohesin Associated Factor B	3.02920
CHST14	Carbohydrate Sulfotransferase 14	3.02865
FABP5	Fatty Acid Binding Protein 5	3.02727
PEAK1	Pseudopodium Enriched Atypical Kinase 1	3.02584
ACAT1	Acetyl-CoA Acetyltransferase 1	3.02574
OS9	OS9 Endoplasmic Reticulum Lectin	3.02514
CALM2	Calmodulin 2	3.02431
PTRH2	Peptidyl-TRNA Hydrolase 2	3.02396
RPS18	Ribosomal Protein S18	3.02371
SETD6	SET Domain Containing 6, Protein Lysine Methyltransferase	3.02322
ONECUT2	One Cut Homeobox 2	3.02317
HGSNAT	Heparan-Alpha-Glucosaminide N-Acetyltransferase	3.02312
PLA2G1B	Phospholipase A2 Group IB	3.02264
ATG12	Autophagy Related 12	3.02258

DHX8	DEAH-Box Helicase 8	3.02031
BFSP1	Beaded Filament Structural Protein 1	3.02022
ABR	ABR Activator Of RhoGEF And GTPase	3.01931
SS18L1	SS18L1 Subunit Of BAF Chromatin Remodeling Complex	3.01898
TMEM70	Transmembrane Protein 70	3.01873
ADCY4	Adenylate Cyclase 4	3.01844
DND1	DND MicroRNA-Mediated Repression Inhibitor 1	3.01690
BCS1L	BCS1 Homolog, Ubiquinol-Cytochrome C Reductase Complex Chaperone	3.01644
RAB10	RAB10, Member RAS Oncogene Family	3.01556
NOX4	NADPH Oxidase 4	3.01541
SON	SON DNA And RNA Binding Protein	3.01408
MYH6	Myosin Heavy Chain 6	3.01348
MAGEL2	MAGE Family Member L2	3.01318
KIF2C	Kinesin Family Member 2C	3.01281
CARD8	Caspase Recruitment Domain Family Member 8	3.01130
TFAM	Transcription Factor A, Mitochondrial	3.01123
LCAT	Lecithin-Cholesterol Acyltransferase	3.01101
LTBR	Lymphotoxin Beta Receptor	3.01043
PNKD	PNKD Metallo-Beta-Lactamase Domain Containing	3.00987
CETN3	Centrin 3	3.00780
CDK14	Cyclin Dependent Kinase 14	3.00672
DPF1	Double PHD Fingers 1	3.00652
PHLDA2	Pleckstrin Homology Like Domain Family A Member 2	3.00634
USP22	Ubiquitin Specific Peptidase 22	3.00578
CAPN6	Calpain 6	3.00321
ADCY8	Adenylate Cyclase 8	3.00277
SEMA4B	Semaphorin 4B	3.00001
SELENON	Selenoprotein N	2.99990
KCTD11	Potassium Channel Tetramerization Domain Containing 11	2.99977
MIR211	MicroRNA 211	2.99825
DNAJB12	DnaJ Heat Shock Protein Family (Hsp40) Member B12	2.99753
LRP2	LDL Receptor Related Protein 2	2.99671
PHEX	Phosphate Regulating Endopeptidase Homolog X-Linked	2.99497
MIR506	MicroRNA 506	2.99449
SSBP4	Single Stranded DNA Binding Protein 4	2.99389
TSPAN32	Tetraspanin 32	2.99350
HNRNPUL1	Heterogeneous Nuclear Ribonucleoprotein U Like 1	2.99317
CSNK1A1L	Casein Kinase 1 Alpha 1 Like	2.99250

SLCO4A1	Solute Carrier Organic Anion Transporter Family Member 4A1	2.99217
APOBEC3B	Apolipoprotein B MRNA Editing Enzyme Catalytic Subunit 3B	2.98972
PFKFB1	6-Phosphofructo-2-Kinase/Fructose-2,6-Biphosphatase 1	2.98942
TP53INP1	Tumor Protein P53 Inducible Nuclear Protein 1	2.98917
ANKRD46	Ankyrin Repeat Domain 46	2.98917
MIR208A	MicroRNA 208a	2.98850
CALML3	Calmodulin Like 3	2.98817
GRHL1	Grainyhead Like Transcription Factor 1	2.98813
NDE1	NudE Neurodevelopment Protein 1	2.98733
ANGPTL2	Angiopoietin Like 2	2.98692
SIRT2	Sirtuin 2	2.98653
CATSPERG	Cation Channel Sperm Associated Auxiliary Subunit Gamma	2.98643
B9D2	B9 Domain Containing 2	2.98572
MCPH1	Microcephalin 1	2.98564
COQ6	Coenzyme Q6, Monooxygenase	2.98542
ARFGEF2	ADP Ribosylation Factor Guanine Nucleotide Exchange Factor 2	2.98540
ENOX2	Ecto-NOX Disulfide-Thiol Exchanger 2	2.98526
B3GALNT2	Beta-1,3-N-Acetylgalactosaminyltransferase 2	2.98483
AGBL2	AGBL Carboxypeptidase 2	2.98478
BCL9	BCL9 Transcription Coactivator	2.98478
DCD	Dermcidin	2.98312
SOAT2	Sterol O-Acyltransferase 2	2.98258
DPM2	Dolichyl-Phosphate Mannosyltransferase Subunit 2, Regulatory	2.98221
GTF2H1	General Transcription Factor IIH Subunit 1	2.98161
SCN1B	Sodium Voltage-Gated Channel Beta Subunit 1	2.98106
CBX5	Chromobox 5	2.98093
ATIC	5-Aminoimidazole-4-Carboxamide Ribonucleotide Formyltransferase/IMP Cyclohydrolase	2.97937
S100PBP	S100P Binding Protein	2.97928
VAV2	Vav Guanine Nucleotide Exchange Factor 2	2.97779
ZNF230	Zinc Finger Protein 230	2.97707
FCHSD2	FCH And Double SH3 Domains 2	2.97383
MIR301B	MicroRNA 301b	2.97327
MAML1	Mastermind Like Transcriptional Coactivator 1	2.97211
AOC1	Amine Oxidase Copper Containing 1	2.97201

KIF5A	Kinesin Family Member 5A	2.97089
ADAMTS18	ADAM Metallopeptidase With Thrombospondin Type 1 Motif 18	2.97061
H3C14	H3 Clustered Histone 14	2.97055
EIF3L	Eukaryotic Translation Initiation Factor 3 Subunit L	2.97009
TMEM216	Transmembrane Protein 216	2.96883
ALDH18A1	Aldehyde Dehydrogenase 18 Family Member A1	2.96843
TWNK	Twinkle MtDNA Helicase	2.96746
BBS2	Bardet-Biedl Syndrome 2	2.96652
KRT6B	Keratin 6B	2.96646
HSCB	HscB Mitochondrial Iron-Sulfur Cluster Cochaperone	2.96624
TKTL1	Transketolase Like 1	2.96514
BOK	BCL2 Family Apoptosis Regulator BOK	2.96400
TARS1	Threonyl-TRNA Synthetase 1	2.96241
RAPGEF1	Rap Guanine Nucleotide Exchange Factor 1	2.96074
GATAD2B	GATA Zinc Finger Domain Containing 2B	2.96052
ANKRD17	Ankyrin Repeat Domain 17	2.96051
HCFC1R1	Host Cell Factor C1 Regulator 1	2.96017
APIP	APAF1 Interacting Protein	2.95993
KHSRP	KH-Type Splicing Regulatory Protein	2.95963
SEMA5B	Semaphorin 5B	2.95931
DAPK2	Death Associated Protein Kinase 2	2.95909
S1PR1	Sphingosine-1-Phosphate Receptor 1	2.95908
BLVRB	Biliverdin Reductase B	2.95878
KLF14	Kruppel Like Factor 14	2.95770
TRMT1	TRNA Methyltransferase 1	2.95766
HDLBP	High Density Lipoprotein Binding Protein	2.95737
GTF2H3	General Transcription Factor IIH Subunit 3	2.95664
AURKC	Aurora Kinase C	2.95636
BLID	BH3-Like Motif Containing, Cell Death Inducer	2.95617
BMP8B	Bone Morphogenetic Protein 8b	2.95614
CST7	Cystatin F	2.95596
SIX5	SIX Homeobox 5	2.95541
TTLL3	Tubulin Tyrosine Ligase Like 3	2.95484
NEURL1	Neuralized E3 Ubiquitin Protein Ligase 1	2.95415
USP44	Ubiquitin Specific Peptidase 44	2.95366
PON2	Paraoxonase 2	2.95154
TMEM205	Transmembrane Protein 205	2.95136
ZCCHC14	Zinc Finger CCHC-Type Containing 14	2.95096
USP39	Ubiquitin Specific Peptidase 39	2.94967

PPBP	Pro-Platelet Basic Protein	2.94935
SNAP25	Synaptosome Associated Protein 25	2.94913
RPL10A	Ribosomal Protein L10a	2.94892
CDCA3	Cell Division Cycle Associated 3	2.94766
SLC45A1	Solute Carrier Family 45 Member 1	2.94659
AIMP1	Aminoacyl TRNA Synthetase Complex Interacting Multifunctional Protein 1	2.94656
TSPAN9	Tetraspanin 9	2.94637
XPNPEP3	X-Prolyl Aminopeptidase 3	2.94608
HLA-DQA2	Major Histocompatibility Complex, Class II, DQ Alpha 2	2.94571
HOXC9	Homeobox C9	2.94507
MRPS7	Mitochondrial Ribosomal Protein S7	2.94484
POMGNT2	Protein O-Linked Mannose N-Acetylglucosaminyltransferase 2 (Beta 1,4-)	2.94479
CDON	Cell Adhesion Associated, Oncogene Regulated	2.94330
KLF13	Kruppel Like Factor 13	2.94228
RBM4	RNA Binding Motif Protein 4	2.94213
IL17RC	Interleukin 17 Receptor C	2.94169
HAVCR1	Hepatitis A Virus Cellular Receptor 1	2.94159
CDC14A	Cell Division Cycle 14A	2.94132
DUSP4	Dual Specificity Phosphatase 4	2.94037
CYSTM1	Cysteine Rich Transmembrane Module Containing 1	2.94001
FUT5	Fucosyltransferase 5	2.93988
RAMP1	Receptor Activity Modifying Protein 1	2.93964
NR2C2	Nuclear Receptor Subfamily 2 Group C Member 2	2.93861
UBA2	Ubiquitin Like Modifier Activating Enzyme 2	2.93801
RND2	Rho Family GTPase 2	2.93761
RANBP3	RAN Binding Protein 3	2.93677
ZNF558	Zinc Finger Protein 558	2.93674
PIP5K1C	Phosphatidylinositol-4-Phosphate 5-Kinase Type 1 Gamma	2.93532
AGGF1	Angiogenic Factor With G-Patch And FHA Domains 1	2.93442
COL8A2	Collagen Type VIII Alpha 2 Chain	2.93308
RPL19	Ribosomal Protein L19	2.93269
CRY1	Cryptochrome Circadian Regulator 1	2.93179
TSLP	Thymic Stromal Lymphopoietin	2.93160
MYOF	Myoferlin	2.93092
ORC2	Origin Recognition Complex Subunit 2	2.93025
MDFI	MyoD Family Inhibitor	2.93023
ZNF300	Zinc Finger Protein 300	2.92992
ZC4H2	Zinc Finger C4H2-Type Containing	2.92991
USP6	Ubiquitin Specific Peptidase 6	2.92950

ASH1L-AS1	ASH1L Antisense RNA 1	2.92882
LY86	Lymphocyte Antigen 86	2.92842
KMT2E	Lysine Methyltransferase 2E (Inactive)	2.92730
TMEM8B	Transmembrane Protein 8B	2.92693
NNAT	Neuronatin	2.92673
ADH1A	Alcohol Dehydrogenase 1A (Class I), Alpha Polypeptide	2.92586
GTPBP3	GTP Binding Protein 3, Mitochondrial	2.92515
SLC10A2	Solute Carrier Family 10 Member 2	2.92494
NEK3	NIMA Related Kinase 3	2.92455
PRUNE1	Prune Exopolyphosphatase 1	2.92424
RAN	RAN, Member RAS Oncogene Family	2.92345
CAPG	Capping Actin Protein, Gelsolin Like	2.92345
UTP14C	UTP14C Small Subunit Processome Component	2.92344
RAPGEF3	Rap Guanine Nucleotide Exchange Factor 3	2.92293
F12	Coagulation Factor XII	2.92258
POLR2L	RNA Polymerase II, I And III Subunit L	2.92131
BRSK1	BR Serine/Threonine Kinase 1	2.92054
DYNLRB2	Dynein Light Chain Roadblock-Type 2	2.91936
ARHGEF39	Rho Guanine Nucleotide Exchange Factor 39	2.91920
MLX	MAX Dimerization Protein MLX	2.91707
NELFA	Negative Elongation Factor Complex Member A	2.91681
CDH10	Cadherin 10	2.91660
IGLL1	Immunoglobulin Lambda Like Polypeptide 1	2.91638
MAPK13	Mitogen-Activated Protein Kinase 13	2.91624
BCL7C	BAF Chromatin Remodeling Complex Subunit BCL7C	2.91537
TRPM3	Transient Receptor Potential Cation Channel Subfamily M Member 3	2.91519
ESPL1	Extra Spindle Pole Bodies Like 1, Separase	2.91507
CYSLTR1	Cysteinyl Leukotriene Receptor 1	2.91447
HSF4	Heat Shock Transcription Factor 4	2.91425
CD226	CD226 Molecule	2.91377
ARL6IP5	ADP Ribosylation Factor Like GTPase 6 Interacting Protein 5	2.91307
NSMCE2	NSE2 (MMS21) Homolog, SMC5-SMC6 Complex SUMO Ligase	2.91173
RNF26	Ring Finger Protein 26	2.91032
APLN	Apelin	2.91022
SORT1	Sortilin 1	2.90977
DRD5	Dopamine Receptor D5	2.90900
RAD51-AS1	RAD51 Antisense RNA 1	2.90844

PPP1R15B	Protein Phosphatase 1 Regulatory Subunit 15B	2.90817
IGSF11	Immunoglobulin Superfamily Member 11	2.90704
GPS1	G Protein Pathway Suppressor 1	2.90573
LOC11080626[illegible]	Solute Carrier Family 6 Member 4 Gene Promoter	2.90415
STEAP1	STEAP Family Member 1	2.90408
EPX	Eosinophil Peroxidase	2.90395
MYO10	Myosin X	2.90368
SCN3B	Sodium Voltage-Gated Channel Beta Subunit 3	2.90254
RBM20	RNA Binding Motif Protein 20	2.90141
EDN2	Endothelin 2	2.90137
ACSL1	Acyl-CoA Synthetase Long Chain Family Member 1	2.90126
TRIP4	Thyroid Hormone Receptor Interactor 4	2.90113
RSL1D1	Ribosomal L1 Domain Containing 1	2.90082
TAX1BP1	Tax1 Binding Protein 1	2.89980
ZNF607	Zinc Finger Protein 607	2.89974
FUBP1	Far Upstream Element Binding Protein 1	2.89889
HEY2	Hes Related Family BHLH Transcription Factor With YRPW Motif 2	2.89717
BCAS4	Breast Carcinoma Amplified Sequence 4	2.89716
MKLN1	Muskelin 1	2.89696
ALDH3A1	Aldehyde Dehydrogenase 3 Family Member A1	2.89665
CXXC5	CXXC Finger Protein 5	2.89540
DIDO1	Death Inducer-Obliterator 1	2.89236
FABP2	Fatty Acid Binding Protein 2	2.89225
CSAG3	CSAG Family Member 3	2.89221
SH3BP2	SH3 Domain Binding Protein 2	2.89122
LUC7L3	LUC7 Like 3 Pre-MRNA Splicing Factor	2.89042
SLC12A4	Solute Carrier Family 12 Member 4	2.89020
TIFA	TRAF Interacting Protein With Forkhead Associated Domain	2.88930
PDZD7	PDZ Domain Containing 7	2.88850
ARMCX1	Armadillo Repeat Containing X-Linked 1	2.88822
GID8	GID Complex Subunit 8 Homolog	2.88796
MEI1	Meiotic Double-Stranded Break Formation Protein 1	2.88662
NRG2	Neuregulin 2	2.88576
PHF20L1	PHD Finger Protein 20 Like 1	2.88505
CLN3	CLN3 Lysosomal/Endosomal Transmembrane Protein, Battenin	2.88372
FRMD4A	FERM Domain Containing 4A	2.88372

RNASEH2C	Ribonuclease H2 Subunit C	2.88235
SPOUT1	SPOUT Domain Containing Methyltransferase 1	2.88226
CDR2	Cerebellar Degeneration Related Protein 2	2.88138
CD52	CD52 Molecule	2.88092
CDC16	Cell Division Cycle 16	2.88085
PNN	Pinin, Desmosome Associated Protein	2.88074
PPL	Periplakin	2.88040
TRIM14	Tripartite Motif Containing 14	2.87779
CTSS	Cathepsin S	2.87758
RFX2	Regulatory Factor X2	2.87731
MAEA	Macrophage Erythroblast Attacher, E3 Ubiquitin Ligase	2.87718
KIAA1217	KIAA1217	2.87643
HEXA	Hexosaminidase Subunit Alpha	2.87635
PTS	6-Pyruvoyltetrahydropterin Synthase	2.87403
PBX3	PBX Homeobox 3	2.87393
CDCA8	Cell Division Cycle Associated 8	2.87389
CELSR1	Cadherin EGF LAG Seven-Pass G-Type Receptor 1	2.87264
LTBP3	Latent Transforming Growth Factor Beta Binding Protein 3	2.87263
ZNF668	Zinc Finger Protein 668	2.87220
SLC30A8	Solute Carrier Family 30 Member 8	2.87217
WDR43	WD Repeat Domain 43	2.87211
PPP2R5D	Protein Phosphatase 2 Regulatory Subunit B'Delta	2.87196
IMPDH2	Inosine Monophosphate Dehydrogenase 2	2.87130
ZNF573	Zinc Finger Protein 573	2.87086
ALG1	ALG1 Chitobiosyldiphosphodolichol Beta-Mannosyltransferase	2.87079
NDUFS4	NADH:Ubiquinone Oxidoreductase Subunit S4	2.87041
NTMT1	N-Terminal Xaa-Pro-Lys N-Methyltransferase 1	2.86953
STOML2	Stomatin Like 2	2.86922
INCENP	Inner Centromere Protein	2.86841
UBAP1	Ubiquitin Associated Protein 1	2.86809
FCHO1	FCH And Mu Domain Containing Endocytic Adaptor 1	2.86648
SLC7A6	Solute Carrier Family 7 Member 6	2.86640
GBE1	1,4-Alpha-Glucan Branching Enzyme 1	2.86634
RHBDD2	Rhomboid Domain Containing 2	2.86449
RAB8A	RAB8A, Member RAS Oncogene Family	2.86403
DEGS2	Delta 4-Desaturase, Sphingolipid 2	2.86398

FAM3B	FAM3 Metabolism Regulating Signaling Molecule B	2.86302
LEFTY2	Left-Right Determination Factor 2	2.85973
PTF1A	Pancreas Associated Transcription Factor 1a	2.85828
SPOCD1	SPOC Domain Containing 1	2.85770
TCP1	T-Complex 1	2.85689
ANKLE1	Ankyrin Repeat And LEM Domain Containing 1	2.85636
MIR885	MicroRNA 885	2.85587
HINT2	Histidine Triad Nucleotide Binding Protein 2	2.85518
NDUFC2	NADH:Ubiquinone Oxidoreductase Subunit C2	2.85509
BLZF1	Basic Leucine Zipper Nuclear Factor 1	2.85502
KCNN3	Potassium Calcium-Activated Channel Subfamily N Member 3	2.85500
NUP205	Nucleoporin 205	2.85482
CAMK2D	Calcium/Calmodulin Dependent Protein Kinase II Delta	2.85391
MRPL28	Mitochondrial Ribosomal Protein L28	2.85363
LINC00707	Long Intergenic Non-Protein Coding RNA 707	2.85255
RFC1	Replication Factor C Subunit 1	2.85188
MIR501	MicroRNA 501	2.84982
CEP63	Centrosomal Protein 63	2.84914
UBR2	Ubiquitin Protein Ligase E3 Component N-Recognin 2	2.84901
GGA3	Golgi Associated, Gamma Adaptin Ear Containing, ARF Binding Protein 3	2.84854
COQ8B	Coenzyme Q8B	2.84786
AP1B1	Adaptor Related Protein Complex 1 Subunit Beta 1	2.84758
PRDX4	Peroxiredoxin 4	2.84678
LPAR5	Lysophosphatidic Acid Receptor 5	2.84635
LYPD3	LY6/PLAUR Domain Containing 3	2.84549
OPTN	Optineurin	2.84518
TAPBP	TAP Binding Protein	2.84501
RPRM	Reprimo, TP53 Dependent G2 Arrest Mediator Homolog	2.84480
TUBB1	Tubulin Beta 1 Class VI	2.84417
PAGR1	PAXIP1 Associated Glutamate Rich Protein 1	2.84397
AHSG	Alpha 2-HS Glycoprotein	2.84379
TUBG2	Tubulin Gamma 2	2.84286
ATG9B	Autophagy Related 9B	2.84226
MIR770	MicroRNA 770	2.84209
C1orf109	Chromosome 1 Open Reading Frame 109	2.84182

SERTAD3	SERTA Domain Containing 3	2.84179
PNPLA3	Patatin Like Phospholipase Domain Containing 3	2.84140
IMPDH1	Inosine Monophosphate Dehydrogenase 1	2.84059
NBPF2P	NBPF Member 2, Pseudogene	2.83721
ADAT3	Adenosine Deaminase TRNA Specific 3	2.83690
LMX1A	LIM Homeobox Transcription Factor 1 Alpha	2.83657
RNF185	Ring Finger Protein 185	2.83585
MIIP	Migration And Invasion Inhibitory Protein	2.83576
NCAPD2	Non-SMC Condensin I Complex Subunit D2	2.83565
HNRNPM	Heterogeneous Nuclear Ribonucleoprotein M	2.83499
CHAF1A	Chromatin Assembly Factor 1 Subunit A	2.83490
RAMP2	Receptor Activity Modifying Protein 2	2.83243
ORAI1	ORAI Calcium Release-Activated Calcium Modulator 1	2.83217
GCN1	GCN1 Activator Of EIF2AK4	2.83188
VAMP8	Vesicle Associated Membrane Protein 8	2.83186
BARX1	BARX Homeobox 1	2.83079
NOLC1	Nucleolar And Coiled-Body Phosphoprotein 1	2.83053
TBC1D10A	TBC1 Domain Family Member 10A	2.82956
MAT2A	Methionine Adenosyltransferase 2A	2.82911
PPA2	Inorganic Pyrophosphatase 2	2.82892
AK2	Adenylate Kinase 2	2.82874
PRRX1	Paired Related Homeobox 1	2.82788
EIF4A2	Eukaryotic Translation Initiation Factor 4A2	2.82746
TUBA4A	Tubulin Alpha 4a	2.82737
ZNF469	Zinc Finger Protein 469	2.82673
LINC01512	Long Intergenic Non-Protein Coding RNA 1512	2.82608
SENP2	SUMO Specific Peptidase 2	2.82604
SNRNP70	Small Nuclear Ribonucleoprotein U1 Subunit 70	2.82540
VPS13A	Vacuolar Protein Sorting 13 Homolog A	2.82514
ENDOV	Endonuclease V	2.82496
PSMC3	Proteasome 26S Subunit, ATPase 3	2.82435
ICE2	Interactor Of Little Elongation Complex ELL Subunit 2	2.82428
BAIAP2-DT	BAIAP2 Divergent Transcript	2.82301
SDHAF1	Succinate Dehydrogenase Complex Assembly Factor 1	2.82141

SHCBP1	SHC Binding And Spindle Associated 1	2.81963
MIR30C2	MicroRNA 30c-2	2.81855
SRI	Sorcin	2.81840
ATG14	Autophagy Related 14	2.81837
A4GALT	Alpha 1,4-Galactosyltransferase (P Blood Group)	2.81833
TLE2	TLE Family Member 2, Transcriptional Corepressor	2.81570
ZNF750	Zinc Finger Protein 750	2.81565
SMYD2	SET And MYND Domain Containing 2	2.81549
ELF1	E74 Like ETS Transcription Factor 1	2.81536
RPS25	Ribosomal Protein S25	2.81432
MED26	Mediator Complex Subunit 26	2.81391
RASGRF2	Ras Protein Specific Guanine Nucleotide Releasing Factor 2	2.81388
CBR3	Carbonyl Reductase 3	2.81314
PLEKHA7	Pleckstrin Homology Domain Containing A7	2.81294
MT-TQ	Mitochondrially Encoded TRNA-Gln (CAA/G)	2.81292
HEYL	Hes Related Family BHLH Transcription Factor With YRPW Motif Like	2.81278
PRPF6	Pre-MRNA Processing Factor 6	2.81235
GPT2	Glutamic--Pyruvic Transaminase 2	2.80943
TRIM26	Tripartite Motif Containing 26	2.80851
CRISPLD2	Cysteine Rich Secretory Protein LCCL Domain Containing 2	2.80774
GAGE12I	G Antigen 12I	2.80766
KIF24	Kinesin Family Member 24	2.80751
STIL	STIL Centriolar Assembly Protein	2.80722
EIF3B	Eukaryotic Translation Initiation Factor 3 Subunit B	2.80710
PSD4	Pleckstrin And Sec7 Domain Containing 4	2.80688
NPR1	Natriuretic Peptide Receptor 1	2.80676
CNKSR1	Connector Enhancer Of Kinase Suppressor Of Ras 1	2.80662
HMGN1	High Mobility Group Nucleosome Binding Domain 1	2.80647
SCO1	Synthesis Of Cytochrome C Oxidase 1	2.80637
SEC16A	SEC16 Homolog A, Endoplasmic Reticulum Export Factor	2.80627
NTN4	Netrin 4	2.80623
MTHFSD	Methenyltetrahydrofolate Synthetase Domain Containing	2.80622
PHF1	PHD Finger Protein 1	2.80587
FBXO17	F-Box Protein 17	2.80522
LACC1	Laccase Domain Containing 1	2.80456

RASSF3	Ras Association Domain Family Member 3	2.80335
TSC22D3	TSC22 Domain Family Member 3	2.80323
ADAP1	ArfGAP With Dual PH Domains 1	2.80266
EYA3	EYA Transcriptional Coactivator And Phosphatase 3	2.80192
ABHD2	Abhydrolase Domain Containing 2, Acylglycerol Lipase	2.79842
SCUBE2	Signal Peptide, CUB Domain And EGF Like Domain Containing 2	2.79833
ERC1	ELKS/RAB6-Interacting/CAST Family Member 1	2.79721
PTGDR2	Prostaglandin D2 Receptor 2	2.79672
CTNNBL1	Catenin Beta Like 1	2.79615
ENSG00000285708		2.79552
NOP53	NOP53 Ribosome Biogenesis Factor	2.79501
PKLR	Pyruvate Kinase L/R	2.79486
KLK12	Kallikrein Related Peptidase 12	2.79387
SLA	Src Like Adaptor	2.79376
EIF3F	Eukaryotic Translation Initiation Factor 3 Subunit F	2.79357
RAB5IF	RAB5 Interacting Factor	2.79319
TSPAN1	Tetraspanin 1	2.79289
SRPK2	SRSF Protein Kinase 2	2.79264
TRAPPC4	Trafficking Protein Particle Complex Subunit 4	2.79261
OXCT1	3-Oxoacid CoA-Transferase 1	2.79247
MPV17	Mitochondrial Inner Membrane Protein MPV17	2.79162
DPYS	Dihydropyrimidinase	2.79125
HAND1	Heart And Neural Crest Derivatives Expressed 1	2.79111
MMP17	Matrix Metallopeptidase 17	2.78894
SOST	Sclerostin	2.78785
LAPTM4A	Lysosomal Protein Transmembrane 4 Alpha	2.78738
AKR7A2	Aldo-Keto Reductase Family 7 Member A2	2.78724
CFB	Complement Factor B	2.78686
RPL4	Ribosomal Protein L4	2.78666
PHF3	PHD Finger Protein 3	2.78663
MYL9	Myosin Light Chain 9	2.78643
RBM8A	RNA Binding Motif Protein 8A	2.78637
GATAD2A	GATA Zinc Finger Domain Containing 2A	2.78524
SDCBP2	Syndecan Binding Protein 2	2.78503
AP4M1	Adaptor Related Protein Complex 4 Subunit Mu 1	2.78463
TNFSF9	TNF Superfamily Member 9	2.78455

SHKBP1	SH3KBP1 Binding Protein 1	2.78428
MIR645	MicroRNA 645	2.78343
FGF16	Fibroblast Growth Factor 16	2.78311
C1R	Complement C1r	2.78305
RPL7A	Ribosomal Protein L7a	2.78240
ESCO1	Establishment Of Sister Chromatid Cohesion N-Acetyltransferase 1	2.78209
GRIN1	Glutamate Ionotropic Receptor NMDA Type Subunit 1	2.78084
PIDD1	P53-Induced Death Domain Protein 1	2.78052
ITGB1BP1	Integrin Subunit Beta 1 Binding Protein 1	2.78049
KCNJ2	Potassium Inwardly Rectifying Channel Subfamily J Member 2	2.77950
SUN1	Sad1 And UNC84 Domain Containing 1	2.77927
SLK	STE20 Like Kinase	2.77829
SMG8	SMG8 Nonsense Mediated MRNA Decay Factor	2.77717
CAPN5	Calpain 5	2.77693
GLRX	Glutaredoxin	2.77669
VAMP2	Vesicle Associated Membrane Protein 2	2.77598
LIMD2	LIM Domain Containing 2	2.77567
SPRED2	Sprouty Related EVH1 Domain Containing 2	2.77553
PGAM1	Phosphoglycerate Mutase 1	2.77470
TCAP	Titin-Cap	2.77429
ZNF142	Zinc Finger Protein 142	2.77362
RPL26L1	Ribosomal Protein L26 Like 1	2.77310
GNLY	Granulysin	2.77278
CORO1B	Coronin 1B	2.77248
BLNK	B Cell Linker	2.77225
SLC39A14	Solute Carrier Family 39 Member 14	2.77212
RAB5C	RAB5C, Member RAS Oncogene Family	2.77199
CDK11B	Cyclin Dependent Kinase 11B	2.77156
DYNC2LI1	Dynein Cytoplasmic 2 Light Intermediate Chain 1	2.76988
GABRA1	Gamma-Aminobutyric Acid Type A Receptor Subunit Alpha1	2.76957
HLA-DMA	Major Histocompatibility Complex, Class II, DM Alpha	2.76889
TPH1	Tryptophan Hydroxylase 1	2.76883
MBD5	Methyl-CpG Binding Domain Protein 5	2.76841
RYK	Receptor Like Tyrosine Kinase	2.76787
MYBBP1A	MYB Binding Protein 1a	2.76769
LRRC8A	Leucine Rich Repeat Containing 8 VRAC Subunit A	2.76728
NFYA	Nuclear Transcription Factor Y Subunit Alpha	2.76688

TRDMT1	TRNA Aspartic Acid Methyltransferase 1	2.76404
BBS4	Bardet-Biedl Syndrome 4	2.76328
ADORA2A	Adenosine A2a Receptor	2.76321
MAGED1	MAGE Family Member D1	2.76205
POMT1	Protein O-Mannosyltransferase 1	2.76155
RIPK4	Receptor Interacting Serine/Threonine Kinase 4	2.76055
TONSL-AS1	TONSL Antisense RNA 1	2.76011
PTPN23	Protein Tyrosine Phosphatase Non-Receptor Type 23	2.76006
RIF1	Replication Timing Regulatory Factor 1	2.75999
PRSS23	Serine Protease 23	2.75987
CTSZ	Cathepsin Z	2.75915
DLX6-AS1	DLX6 Antisense RNA 1	2.75885
COX4I2	Cytochrome C Oxidase Subunit 4I2	2.75847
HNRNPA3	Heterogeneous Nuclear Ribonucleoprotein A3	2.75839
ENSG00000258798		2.75809
SMOX	Spermine Oxidase	2.75680
TMED10	Transmembrane P24 Trafficking Protein 10	2.75653
ZIC2	Zic Family Member 2	2.75643
PSME3	Proteasome Activator Subunit 3	2.75613
PSMB11	Proteasome Subunit Beta 11	2.75522
ZNF384	Zinc Finger Protein 384	2.75510
FNBP1L	Formin Binding Protein 1 Like	2.75469
RAB7A	RAB7A, Member RAS Oncogene Family	2.75462
HBG2	Hemoglobin Subunit Gamma 2	2.75448
SNRPD1	Small Nuclear Ribonucleoprotein D1 Polypeptide	2.75408
CIRBP	Cold Inducible RNA Binding Protein	2.75237
GUCA1B	Guanylate Cyclase Activator 1B	2.75223
RING1	Ring Finger Protein 1	2.75218
SUMO2	Small Ubiquitin Like Modifier 2	2.75160
SLC15A1	Solute Carrier Family 15 Member 1	2.75004
ADCY1	Adenylate Cyclase 1	2.74950
SIN3B	SIN3 Transcription Regulator Family Member B	2.74889
FBLIM1	Filamin Binding LIM Protein 1	2.74839
LY6G5B	Lymphocyte Antigen 6 Family Member G5B	2.74814
LOC10012906(	Uncharacterized LOC100129066	2.74668
TSFM	Ts Translation Elongation Factor, Mitochondrial	2.74638
HLA-DRB5	Major Histocompatibility Complex, Class II, DR Beta 5	2.74606

WWP2	WW Domain Containing E3 Ubiquitin Protein Ligase 2	2.74598
FTL	Ferritin Light Chain	2.74548
PPP1R12A	Protein Phosphatase 1 Regulatory Subunit 12A	2.74543
RNF126	Ring Finger Protein 126	2.74475
PARD3	Par-3 Family Cell Polarity Regulator	2.74444
CARD9	Caspase Recruitment Domain Family Member 9	2.74415
SETD4	SET Domain Containing 4	2.74404
LRSAM1	Leucine Rich Repeat And Sterile Alpha Motif Containing 1	2.74301
BANK1	B Cell Scaffold Protein With Ankyrin Repeats 1	2.74241
OMP	Olfactory Marker Protein	2.74201
AKAP4	A-Kinase Anchoring Protein 4	2.74175
TXNDC11	Thioredoxin Domain Containing 11	2.74147
ILF3-DT	ILF3 Divergent Transcript	2.74063
MIR33B	MicroRNA 33b	2.74056
CLK1	CDC Like Kinase 1	2.74001
ALKBH2	AlkB Homolog 2, Alpha-Ketoglutarate Dependent Dioxygenase	2.73945
COPS6	COP9 Signalosome Subunit 6	2.73779
MGAM	Maltase-Glucoamylase	2.73736
TRIM32	Tripartite Motif Containing 32	2.73596
TUBGCP6	Tubulin Gamma Complex Associated Protein 6	2.73574
RCOR1	REST Corepressor 1	2.73404
TAPBPL	TAP Binding Protein Like	2.73390
EZH1	Enhancer Of Zeste 1 Polycomb Repressive Complex 2 Subunit	2.73358
NID2	Nidogen 2	2.73358
ZC3H11A	Zinc Finger CCCH-Type Containing 11A	2.73327
MCAT	Malonyl-CoA-Acyl Carrier Protein Transacylase	2.73310
CHST3	Carbohydrate Sulfotransferase 3	2.73304
TOM1L2	Target Of Myb1 Like 2 Membrane Trafficking Protein	2.73283
HAUS8	HAUS Augmin Like Complex Subunit 8	2.73279
MIR411	MicroRNA 411	2.73162
PAN2	Poly(A) Specific Ribonuclease Subunit PAN2	2.73063
FA2H	Fatty Acid 2-Hydroxylase	2.73025
GSTA3	Glutathione S-Transferase Alpha 3	2.72988
RCC1	Regulator Of Chromosome Condensation 1	2.72973
CCDC50	Coiled-Coil Domain Containing 50	2.72937

FBH1	F-Box DNA Helicase 1	2.72933
ATG13	Autophagy Related 13	2.72922
SECTM1	Secreted And Transmembrane 1	2.72913
EPB41	Erythrocyte Membrane Protein Band 4.1	2.72897
AGAP1	ArfGAP With GTPase Domain, Ankyrin Repeat And PH Domain 1	2.72807
EEF1D	Eukaryotic Translation Elongation Factor 1 Delta	2.72726
PLXND1	Plexin D1	2.72701
DSC2	Desmocollin 2	2.72681
MHRT	Myosin Heavy Chain Associated RNA Transcript	2.72665
LSM2	LSM2 Homolog, U6 Small Nuclear RNA And MRNA Degradation Associated	2.72601
GRHL2	Grainyhead Like Transcription Factor 2	2.72554
SLC37A4	Solute Carrier Family 37 Member 4	2.72492
LTB4R	Leukotriene B4 Receptor	2.72485
MT1G	Metallothionein 1G	2.72437
FRS3	Fibroblast Growth Factor Receptor Substrate 3	2.72363
PRSS3	Serine Protease 3	2.72321
UCK2	Uridine-Cytidine Kinase 2	2.72282
IGHM	Immunoglobulin Heavy Constant Mu	2.72215
PDE5A	Phosphodiesterase 5A	2.72166
PTPN2	Protein Tyrosine Phosphatase Non-Receptor Type 2	2.72156
DCTN2	Dynactin Subunit 2	2.71959
CHRND	Cholinergic Receptor Nicotinic Delta Subunit	2.71918
RAB27B	RAB27B, Member RAS Oncogene Family	2.71905
DGUOK	Deoxyguanosine Kinase	2.71875
NMNAT1	Nicotinamide Nucleotide Adenylyltransferase 1	2.71862
ARFGAP1	ADP Ribosylation Factor GTPase Activating Protein 1	2.71851
RPN1	Ribophorin I	2.71797
CYREN	Cell Cycle Regulator Of NHEJ	2.71792
HYAL2	Hyaluronidase 2	2.71754
DMP1	Dentin Matrix Acidic Phosphoprotein 1	2.71657
GALNT5	Polypeptide N-Acetylgalactosaminyltransferase 5	2.71650
BUD23	BUD23 RRNA Methyltransferase And Ribosome Maturation Factor	2.71607
DIS3L	DIS3 Like Exosome 3'-5' Exoribonuclease	2.71530
MIR492	MicroRNA 492	2.71478

ZNF408	Zinc Finger Protein 408	2.71457
MAP3K9	Mitogen-Activated Protein Kinase Kinase Kinase 9	2.71446
DDIAS	DNA Damage Induced Apoptosis Suppressor	2.71420
HMGN2	High Mobility Group Nucleosomal Binding Domain 2	2.71410
BPTF	Bromodomain PHD Finger Transcription Factor	2.71287
PAK2	P21 (RAC1) Activated Kinase 2	2.71269
PSMA6	Proteasome 20S Subunit Alpha 6	2.71262
GREM2	Gremlin 2, DAN Family BMP Antagonist	2.71213
CLCN5	Chloride Voltage-Gated Channel 5	2.71201
AGTR2	Angiotensin II Receptor Type 2	2.71197
STRAP	Serine/Threonine Kinase Receptor Associated Protein	2.71049
TNKS1BP1	Tankyrase 1 Binding Protein 1	2.71038
EFHD2	EF-Hand Domain Family Member D2	2.70994
UBE2J2	Ubiquitin Conjugating Enzyme E2 J2	2.70974
CCDC97	Coiled-Coil Domain Containing 97	2.70890
SMARCD2	SWI/SNF Related, Matrix Associated, Actin Dependent Regulator Of Chromatin, Subfamily D, Member 2	2.70853
SNRPE	Small Nuclear Ribonucleoprotein Polypeptide E	2.70830
KLF11	Kruppel Like Factor 11	2.70773
PIK3AP1	Phosphoinositide-3-Kinase Adaptor Protein 1	2.70767
UHRF2	Ubiquitin Like With PHD And Ring Finger Domains 2	2.70764
APOC1	Apolipoprotein C1	2.70750
SKIL	SKI Like Proto-Oncogene	2.70673
SLC9A8	Solute Carrier Family 9 Member A8	2.70598
IFNL3	Interferon Lambda 3	2.70588
CDKAL1	CDK5 Regulatory Subunit Associated Protein 1 Like 1	2.70537
CUEDC1	CUE Domain Containing 1	2.70488
PPP3CA	Protein Phosphatase 3 Catalytic Subunit Alpha	2.70463
DISP1	Dispatched RND Transporter Family Member 1	2.70439
PLEKHG2	Pleckstrin Homology And RhoGEF Domain Containing G2	2.70361
MIR638	MicroRNA 638	2.70216
TMEM51	Transmembrane Protein 51	2.70177
KCNA4	Potassium Voltage-Gated Channel Subfamily A Member 4	2.70174
PHF19	PHD Finger Protein 19	2.70146

FOXR1	Forkhead Box R1	2.70063
CCND3P1	Cyclin D3 Pseudogene 1	2.69982
P2RX3	Purinergic Receptor P2X 3	2.69971
PSMC3IP	PSMC3 Interacting Protein	2.69869
ATG4A	Autophagy Related 4A Cysteine Peptidase	2.69545
SNTB2	Syntrophin Beta 2	2.69502
RAB1B	RAB1B, Member RAS Oncogene Family	2.69457
MSX2	Msh Homeobox 2	2.69367
PROK1	Prokineticin 1	2.69358
FGF22	Fibroblast Growth Factor 22	2.69302
SLC10A1	Solute Carrier Family 10 Member 1	2.69239
KRT9	Keratin 9	2.69191
CAPN10	Calpain 10	2.69114
CLEC4M	C-Type Lectin Domain Family 4 Member M	2.69031
SPC24	SPC24 Component Of NDC80 Kinetochore Complex	2.68883
KLF8	Kruppel Like Factor 8	2.68696
STX5	Syntaxin 5	2.68684
APBA3	Amyloid Beta Precursor Protein Binding Family A Member 3	2.68610
KCNE3	Potassium Voltage-Gated Channel Subfamily E Regulatory Subunit 3	2.68596
PIP4K2B	Phosphatidylinositol-5-Phosphate 4-Kinase Type 2 Beta	2.68589
GRIN2D	Glutamate Ionotropic Receptor NMDA Type Subunit 2D	2.68447
COMP	Cartilage Oligomeric Matrix Protein	2.68328
CACTIN	Cactin, Spliceosome C Complex Subunit	2.68251
MIR7-3HG	MIR7-3 Host Gene	2.68232
SF3A2	Splicing Factor 3a Subunit 2	2.68212
NECTIN2	Nectin Cell Adhesion Molecule 2	2.68084
CNTRL	Centriolin	2.68068
PPP1R8	Protein Phosphatase 1 Regulatory Subunit 8	2.68043
CLCF1	Cardiotrophin Like Cytokine Factor 1	2.68031
IL10RA	Interleukin 10 Receptor Subunit Alpha	2.67781
BACH2	BTB Domain And CNC Homolog 2	2.67778
SRPX2	Sushi Repeat Containing Protein X-Linked 2	2.67730
WBP1L	WW Domain Binding Protein 1 Like	2.67728
UBE2S	Ubiquitin Conjugating Enzyme E2 S	2.67694
SAMD4B	Sterile Alpha Motif Domain Containing 4B	2.67603
SPEG	Striated Muscle Enriched Protein Kinase	2.67557
SCYL1	SCY1 Like Pseudokinase 1	2.67409
P3H3	Prolyl 3-Hydroxylase 3	2.67389

HSD3B7	Hydroxy-Delta-5-Steroid Dehydrogenase, 3 Beta- And Steroid Delta-Isomerase 7	2.67385
PPP1R12C	Protein Phosphatase 1 Regulatory Subunit 12C	2.67370
SURF6	Surfeit 6	2.67335
TRIM16	Tripartite Motif Containing 16	2.67315
GEMIN4	Gem Nuclear Organelle Associated Protein 4	2.67291
YJU2B	YJU2 Splicing Factor Homolog B	2.67232
CENPH	Centromere Protein H	2.67119
MYDGF	Myeloid Derived Growth Factor	2.67016
ENSG00000272489		2.66989
LTA4H	Leukotriene A4 Hydrolase	2.66969
ADCY9	Adenylate Cyclase 9	2.66950
FPR2	Formyl Peptide Receptor 2	2.66923
SH3PXD2B	SH3 And PX Domains 2B	2.66829
MYOM1	Myomesin 1	2.66734
MIR433	MicroRNA 433	2.66730
LINC01969	Long Intergenic Non-Protein Coding RNA 1969	2.66721
TMEM106C	Transmembrane Protein 106C	2.66579
TAF4	TATA-Box Binding Protein Associated Factor 4	2.66542
IQGAP2	IQ Motif Containing GTPase Activating Protein 2	2.66448
ZMIZ1-AS1	ZMIZ1 Antisense RNA 1	2.66425
NPPC	Natriuretic Peptide C	2.66385
PIP5KL1	Phosphatidylinositol-4-Phosphate 5-Kinase Like 1	2.66368
PPP1R13B	Protein Phosphatase 1 Regulatory Subunit 13B	2.66202
NASP	Nuclear Autoantigenic Sperm Protein	2.66165
TRAK1	Trafficking Kinesin Protein 1	2.66164
TREH	Trehalase	2.66160
PTPN14	Protein Tyrosine Phosphatase Non-Receptor Type 14	2.66125
ZNF648	Zinc Finger Protein 648	2.65991
CIB2	Calcium And Integrin Binding Family Member 2	2.65989
DCAF5	DDB1 And CUL4 Associated Factor 5	2.65914
MIR383	MicroRNA 383	2.65899
THEM6	Thioesterase Superfamily Member 6	2.65888
RGL2	Ral Guanine Nucleotide Dissociation Stimulator Like 2	2.65877
MYLK3	Myosin Light Chain Kinase 3	2.65838
AGRP	Agouti Related Neuropeptide	2.65830
SALL1	Spalt Like Transcription Factor 1	2.65821
ENSG00000238221		2.65791

EIF2AK4	Eukaryotic Translation Initiation Factor 2 Alpha Kinase 4	2.65781
PCDH1	Protocadherin 1	2.65764
MLF2	Myeloid Leukemia Factor 2	2.65700
MAGEE1	MAGE Family Member E1	2.65660
IL20	Interleukin 20	2.65657
VPS11	VPS11 Core Subunit Of CORVET And HOPS Complexes	2.65625
SYVN1	Synoviolin 1	2.65585
CDCA5	Cell Division Cycle Associated 5	2.65555
NISCH	Nischarin	2.65525
PPP2CB	Protein Phosphatase 2 Catalytic Subunit Beta	2.65512
NHEJ1	Non-Homologous End Joining Factor 1	2.65475
MAL	Mal, T Cell Differentiation Protein	2.65462
CLCN2	Chloride Voltage-Gated Channel 2	2.65456
HSPE1	Heat Shock Protein Family E (Hsp10) Member 1	2.65356
FMOD	Fibromodulin	2.65195
JMJD6	Jumonji Domain Containing 6, Arginine Demethylase And Lysine Hydroxylase	2.65166
RANBP1	RAN Binding Protein 1	2.65159
ENSG00000256407		2.65143
MKKS	MKKS Centrosomal Shuttling Protein	2.65106
EFNB1	Ephrin B1	2.65050
ZIC5	Zic Family Member 5	2.65008
ASXL2	ASXL Transcriptional Regulator 2	2.64995
CD2AP	CD2 Associated Protein	2.64974
H2AC21	H2A Clustered Histone 21	2.64921
PRDM15	PR/SET Domain 15	2.64895
LINC01503	Long Intergenic Non-Protein Coding RNA 1503	2.64871
PLXNB1	Plexin B1	2.64857
MORC1	MORC Family CW-Type Zinc Finger 1	2.64841
MIR188	MicroRNA 188	2.64810
RETNLB	Resistin Like Beta	2.64773
ZFP90	ZFP90 Zinc Finger Protein	2.64761
AS3MT	Arsenite Methyltransferase	2.64755
P3H1	Prolyl 3-Hydroxylase 1	2.64739
SNIP1	Smad Nuclear Interacting Protein 1	2.64703
P4HA2	Prolyl 4-Hydroxylase Subunit Alpha 2	2.64699
F11R	F11 Receptor	2.64674
IFT140	Intraflagellar Transport 140	2.64647
PDSS2	Decaprenyl Diphosphate Synthase Subunit 2	2.64646
PYCR1	Pyrroline-5-Carboxylate Reductase 1	2.64631
RTN4R	Reticulon 4 Receptor	2.64598

SRMS	Src-Related Kinase Lacking C-Terminal Regulatory Tyrosine And N-Terminal Myristylation Sites	2.64587
NFKBIB	NFKB Inhibitor Beta	2.64549
IL18BP	Interleukin 18 Binding Protein	2.64538
TCFL5	Transcription Factor Like 5	2.64537
MAST2	Microtubule Associated Serine/Threonine Kinase 2	2.64525
ZC3H7A	Zinc Finger CCCH-Type Containing 7A	2.64462
HLCS	Holocarboxylase Synthetase	2.64426
GTF3C4	General Transcription Factor IIIC Subunit 4	2.64369
WDR48	WD Repeat Domain 48	2.64354
NAPA	NSF Attachment Protein Alpha	2.64315
NALT1	NOTCH1 Associated LncRNA In T Cell Acute Lymphoblastic Leukemia 1	2.64310
C10orf105	Chromosome 10 Open Reading Frame 105	2.64221
ARHGEF6	Rac/Cdc42 Guanine Nucleotide Exchange Factor 6	2.64198
ARNT2	Aryl Hydrocarbon Receptor Nuclear Translocator 2	2.64179
ATP1B2	ATPase Na+/K+ Transporting Subunit Beta 2	2.64158
PLD6	Phospholipase D Family Member 6	2.64148
IQCH	IQ Motif Containing H	2.64071
ENSG00000269688		2.64071
CEBPD	CCAAT Enhancer Binding Protein Delta	2.64025
PPP1R12B	Protein Phosphatase 1 Regulatory Subunit 12B	2.63987
PPP6R3	Protein Phosphatase 6 Regulatory Subunit 3	2.63864
RPS12	Ribosomal Protein S12	2.63834
SLC25A51	Solute Carrier Family 25 Member 51	2.63829
PPP1R9B	Protein Phosphatase 1 Regulatory Subunit 9B	2.63783
FCER2	Fc Fragment Of IgE Receptor II	2.63627
ANOS1	Anosmin 1	2.63585
TNFAIP8L2	TNF Alpha Induced Protein 8 Like 2	2.63468
GJB3	Gap Junction Protein Beta 3	2.63389
CCDC12	Coiled-Coil Domain Containing 12	2.63364
MORC3	MORC Family CW-Type Zinc Finger 3	2.63279
ELL2	Elongation Factor For RNA Polymerase II 2	2.63254
DYNLL1	Dynein Light Chain LC8-Type 1	2.63133
ARC	Activity Regulated Cytoskeleton Associated Protein	2.63084
CLDN14	Claudin 14	2.63066
CALML5	Calmodulin Like 5	2.62966

ENTPD1	Ectonucleoside Triphosphate Diphosphohydrolase 1	2.62894
DHX38	DEAH-Box Helicase 38	2.62893
AAK1	AP2 Associated Kinase 1	2.62886
CCT7	Chaperonin Containing TCP1 Subunit 7	2.62874
SHROOM3	Shroom Family Member 3	2.62816
MYLK4	Myosin Light Chain Kinase Family Member 4	2.62759
CTSC	Cathepsin C	2.62725
RGS5	Regulator Of G Protein Signaling 5	2.62638
LTBP1	Latent Transforming Growth Factor Beta Binding Protein 1	2.62636
SLC4A7	Solute Carrier Family 4 Member 7	2.62617
APOH	Apolipoprotein H	2.62575
CELF2	CUGBP Elav-Like Family Member 2	2.62409
SF3B4	Splicing Factor 3b Subunit 4	2.62275
RSPH9	Radial Spoke Head Component 9	2.62256
TPM2	Tropomyosin 2	2.62243
PRIM1	DNA Primase Subunit 1	2.62169
DEFA1	Defensin Alpha 1	2.62142
PEX11B	Peroxisomal Biogenesis Factor 11 Beta	2.62112
AIF1L	Allograft Inflammatory Factor 1 Like	2.62070
MAGEF1	MAGE Family Member F1	2.62055
CYTH1	Cytohesin 1	2.62003
WDR20	WD Repeat Domain 20	2.61996
AAR2	AAR2 Splicing Factor	2.61978
BICRA	BRD4 Interacting Chromatin Remodeling Complex Associated Protein	2.61756
HTR1B	5-Hydroxytryptamine Receptor 1B	2.61728
LSR	Lipolysis Stimulated Lipoprotein Receptor	2.61726
CFL2	Cofilin 2	2.61724
TOR1B	Torsin Family 1 Member B	2.61684
KLF9-DT	KLF9 Divergent Transcript	2.61678
FGGY	FGGY Carbohydrate Kinase Domain Containing	2.61645
HOXC10	Homeobox C10	2.61593
RND3	Rho Family GTPase 3	2.61582
RASAL2	RAS Protein Activator Like 2	2.61524
ENSG00000246203		2.61502
SULT1A3	Sulfotransferase Family 1A Member 3	2.61464
B4GALT5	Beta-1,4-Galactosyltransferase 5	2.61430
LOC11263999	ABO 3' Regulatory Region	2.61417
SRGAP3	SLIT-ROBO Rho GTPase Activating Protein 3	2.61368
PKNOX1	PBX/Knotted 1 Homeobox 1	2.61307
ARCN1	Archain 1	2.61273
NAT10	N-Acetyltransferase 10	2.61257

UNC45A	Unc-45 Myosin Chaperone A	2.61217
PLOD2	Procollagen-Lysine,2-Oxoglutarate 5-Dioxygenase 2	2.61156
COASY	Coenzyme A Synthase	2.61018
CCT2	Chaperonin Containing TCP1 Subunit 2	2.61010
CLDN8	Claudin 8	2.60980
DNAL1	Dynein Axonemal Light Chain 1	2.60933
BRPF1	Bromodomain And PHD Finger Containing 1	2.60867
CARM1	Coactivator Associated Arginine Methyltransferase 1	2.60867
RPL22	Ribosomal Protein L22	2.60855
MIR362	MicroRNA 362	2.60841
CAPN15	Calpain 15	2.60807
SLC36A1	Solute Carrier Family 36 Member 1	2.60773
MTMR2	Myotubularin Related Protein 2	2.60688
SLURP1	Secreted LY6/PLAUR Domain Containing 1	2.60564
CCNJ	Cyclin J	2.60563
HCFC1	Host Cell Factor C1	2.60560
TRIOBP	TRIO And F-Actin Binding Protein	2.60546
SBNO1	Strawberry Notch Homolog 1	2.60490
BCL7A	BAF Chromatin Remodeling Complex Subunit BCL7A	2.60481
GKN3P	Gastrokine 3, Pseudogene	2.60445
KAT7	Lysine Acetyltransferase 7	2.60422
SMUG1	Single-Strand-Selective Monofunctional Uracil-DNA Glycosylase 1	2.60421
EEF1AKMT2	EEF1A Lysine Methyltransferase 2	2.60385
ACKR2	Atypical Chemokine Receptor 2	2.60385
MNX1	Motor Neuron And Pancreas Homeobox 1	2.60283
KCNJ15	Potassium Inwardly Rectifying Channel Subfamily J Member 15	2.60257
GNGT2	G Protein Subunit Gamma Transducin 2	2.60169
VPS18	VPS18 Core Subunit Of CORVET And HOPS Complexes	2.60156
COQ8A	Coenzyme Q8A	2.60144
CCT4	Chaperonin Containing TCP1 Subunit 4	2.60065
SKA2	Spindle And Kinetochore Associated Complex Subunit 2	2.59935
ADCY3	Adenylate Cyclase 3	2.59928
SMNDC1	Survival Motor Neuron Domain Containing 1	2.59911
AQP2	Aquaporin 2	2.59904
TCEA1	Transcription Elongation Factor A1	2.59894
NDRG4	NDRG Family Member 4	2.59700
BYSL	Bystin Like	2.59670
CTSG	Cathepsin G	2.59636

PROC	Protein C, Inactivator Of Coagulation Factors Va And VIIIa	2.59626
AAAS	Aladin WD Repeat Nucleoporin	2.59541
GADD45GIP1	GADD45G Interacting Protein 1	2.59539
TSSC4	Tumor Suppressing Subtransferable Candidate 4	2.59475
STK26	Serine/Threonine Kinase 26	2.59383
GRB10	Growth Factor Receptor Bound Protein 10	2.59372
GMCL1	Germ Cell-Less 1, Spermatogenesis Associated	2.59355
SSR2	Signal Sequence Receptor Subunit 2	2.59342
KLF3	Kruppel Like Factor 3	2.59329
ZNF609	Zinc Finger Protein 609	2.59262
STRA6	Signaling Receptor And Transporter Of Retinol STRA6	2.59140
EBNA1BP2	EBNA1 Binding Protein 2	2.59121
SORD	Sorbitol Dehydrogenase	2.59120
GPAA1	Glycosylphosphatidylinositol Anchor Attachment 1	2.59095
ENPP6	Ectonucleotide Pyrophosphatase/Phosphodiesterase 6	2.59082
DCAF8	DDB1 And CUL4 Associated Factor 8	2.59073
ABCG1	ATP Binding Cassette Subfamily G Member 1	2.59041
ATF6B	Activating Transcription Factor 6 Beta	2.58981
SAP30BP	SAP30 Binding Protein	2.58981
FAM91A1	Family With Sequence Similarity 91 Member A1	2.58936
RNF113B	Ring Finger Protein 113B	2.58925
STYXL1	Serine/Threonine/Tyrosine Interacting Like 1	2.58850
ARHGEF11	Rho Guanine Nucleotide Exchange Factor 11	2.58848
TSHB	Thyroid Stimulating Hormone Subunit Beta	2.58811
PRDX6	Peroxiredoxin 6	2.58650
PRKCSH	Protein Kinase C Substrate 80K-H	2.58578
USF1	Upstream Transcription Factor 1	2.58489
ZDHHC4	Zinc Finger DHHC-Type Palmitoyltransferase 4	2.58475
ITCH	Itchy E3 Ubiquitin Protein Ligase	2.58421
TENT5B	Terminal Nucleotidyltransferase 5B	2.58398
LANCL2	LanC Like 2	2.58350
MIR302B	MicroRNA 302b	2.58236
EIF5	Eukaryotic Translation Initiation Factor 5	2.58226
CDCA7L	Cell Division Cycle Associated 7 Like	2.58087
OTUD7A	OTU Deubiquitinase 7A	2.57958

HCRTR1	Hypocretin Receptor 1	2.57929
WTAP	WT1 Associated Protein	2.57914
TUBA1B	Tubulin Alpha 1b	2.57861
NR2F2-AS1	NR2F2 Antisense RNA 1	2.57858
STING1	Stimulator Of Interferon Response CGAMP Interactor 1	2.57756
DNAJA2	DnaJ Heat Shock Protein Family (Hsp40) Member A2	2.57663
FBXL14	F-Box And Leucine Rich Repeat Protein 14	2.57660
KIAA1522	KIAA1522	2.57510
MBTD1	Mbt Domain Containing 1	2.57508
ART3	ADP-Ribosyltransferase 3 (Inactive)	2.57499
MT3	Metallothionein 3	2.57495
YTHDF2	YTH N6-Methyladenosine RNA Binding Protein 2	2.57474
DST	Dystonin	2.57445
KCNA5	Potassium Voltage-Gated Channel Subfamily A Member 5	2.57415
EPN1	Epsin 1	2.57373
G6PC3	Glucose-6-Phosphatase Catalytic Subunit 3	2.57338
MCOLN1	Mucolipin TRP Cation Channel 1	2.57221
CDIP1	Cell Death Inducing P53 Target 1	2.57091
DCST2	DC-STAMP Domain Containing 2	2.57020
MIR138-2	MicroRNA 138-2	2.56987
ODAM	Odontogenic, Ameloblast Associated	2.56987
XIRP1	Xin Actin Binding Repeat Containing 1	2.56888
RNF183	Ring Finger Protein 183	2.56734
MGAT4A	Alpha-1,3-Mannosyl-Glycoprotein 4-Beta-N-Acetylglucosaminyltransferase A	2.56647
RAB4B-EGLN2	RAB4B-EGLN2 Readthrough (NMD Candidate)	2.56594
KDM2B	Lysine Demethylase 2B	2.56559
PIP5K1A	Phosphatidylinositol-4-Phosphate 5-Kinase Type 1 Alpha	2.56395
RETREG3	Reticulophagy Regulator Family Member 3	2.56386
MTMR12	Myotubularin Related Protein 12	2.56300
EFNB2	Ephrin B2	2.56239
EGFL7	EGF Like Domain Multiple 7	2.56152
USP14	Ubiquitin Specific Peptidase 14	2.56065
SENP3	SUMO Specific Peptidase 3	2.56038
SPECC1L	Sperm Antigen With Calponin Homology And Coiled-Coil Domains 1 Like	2.56006
RBMS1	RNA Binding Motif Single Stranded Interacting Protein 1	2.55933

FSHB	Follicle Stimulating Hormone Subunit Beta	2.55918
WDR59	WD Repeat Domain 59	2.55857
ITPR2	Inositol 1,4,5-Trisphosphate Receptor Type 2	2.55763
PSIP1	PC4 And SFRS1 Interacting Protein 1	2.55724
KRT10-AS1	KRT10 Antisense RNA 1	2.55633
IL27	Interleukin 27	2.55598
COBL	Cordon-Bleu WH2 Repeat Protein	2.55541
CAPN12	Calpain 12	2.55537
NBPF10	NBPF Member 10	2.55527
NRM	Nurim	2.55517
APMAP	Adipocyte Plasma Membrane Associated Protein	2.55480
MED24	Mediator Complex Subunit 24	2.55450
CBX4	Chromobox 4	2.55412
POLL	DNA Polymerase Lambda	2.55382
ENSG00000285800		2.55369
CRTC2	CREB Regulated Transcription Coactivator 2	2.55235
NUP160	Nucleoporin 160	2.55198
MICALL1	MICAL Like 1	2.55175
TRIR	Telomerase RNA Component Interacting RNase	2.55058
ZNF22	Zinc Finger Protein 22	2.55024
SEMA3E	Semaphorin 3E	2.54948
ZNF44	Zinc Finger Protein 44	2.54915
CBX1	Chromobox 1	2.54889
DOCK6	Dedicator Of Cytokinesis 6	2.54849
GOLGA2	Golgin A2	2.54805
C19orf48	Chromosome 19 Open Reading Frame 48	2.54727
UTP14A	UTP14A Small Subunit Processome Component	2.54618
SESN2	Sestrin 2	2.54606
ZNF225	Zinc Finger Protein 225	2.54583
PTGES3	Prostaglandin E Synthase 3	2.54557
ZNF569	Zinc Finger Protein 569	2.54457
PARD6B	Par-6 Family Cell Polarity Regulator Beta	2.54435
TM9SF3	Transmembrane 9 Superfamily Member 3	2.54354
ALPI	Alkaline Phosphatase, Intestinal	2.54192
MIR133A2	MicroRNA 133a-2	2.54183
FAM83H	Family With Sequence Similarity 83 Member H	2.54160
TUBD1	Tubulin Delta 1	2.54151
KDF1	Keratinocyte Differentiation Factor 1	2.54139
DHX16	DEAH-Box Helicase 16	2.54087

KIAA0753	KIAA0753	2.54044
MMS19	MMS19 Homolog, Cytosolic Iron-Sulfur Assembly Component	2.54024
EMP2	Epithelial Membrane Protein 2	2.53921
RASGEF1A	RasGEF Domain Family Member 1A	2.53914
RPH3AL	Rabphilin 3A Like (Without C2 Domains)	2.53897
PREP	Prolyl Endopeptidase	2.53815
SUSD1	Sushi Domain Containing 1	2.53798
CALM3	Calmodulin 3	2.53779
SIRT5	Sirtuin 5	2.53721
SSH1	Slingshot Protein Phosphatase 1	2.53704
PLAGL2	PLAG1 Like Zinc Finger 2	2.53590
ZNF546	Zinc Finger Protein 546	2.53590
UBE2Q1	Ubiquitin Conjugating Enzyme E2 Q1	2.53556
KLRD1	Killer Cell Lectin Like Receptor D1	2.53529
GRIA3	Glutamate Ionotropic Receptor AMPA Type Subunit 3	2.53324
AURKAIP1	Aurora Kinase A Interacting Protein 1	2.53277
TAX1BP3	Tax1 Binding Protein 3	2.53274
DEPDC1B	DEP Domain Containing 1B	2.53257
IFITM3	Interferon Induced Transmembrane Protein 3	2.53192
CIAPIN1	Cytokine Induced Apoptosis Inhibitor 1	2.53124
MAU2	MAU2 Sister Chromatid Cohesion Factor	2.53116
SUSD6	Sushi Domain Containing 6	2.53047
DUS2	Dihydrouridine Synthase 2	2.53013
PDCD10	Programmed Cell Death 10	2.52914
WDR88	WD Repeat Domain 88	2.52909
ARHGEF10	Rho Guanine Nucleotide Exchange Factor 10	2.52861
SLC35C1	Solute Carrier Family 35 Member C1	2.52858
ORM1	Orosomucoid 1	2.52812
ELF5	E74 Like ETS Transcription Factor 5	2.52780
ARHGEF10L	Rho Guanine Nucleotide Exchange Factor 10 Like	2.52769
SFRP5	Secreted Frizzled Related Protein 5	2.52752
SLC29A3	Solute Carrier Family 29 Member 3	2.52671
ABCB9	ATP Binding Cassette Subfamily B Member 9	2.52534
MGST1	Microsomal Glutathione S-Transferase 1	2.52523
PRICKLE4	Prickle Planar Cell Polarity Protein 4	2.52497
H3-4	H3.4 Histone	2.52496
FAM131A	Family With Sequence Similarity 131 Member A	2.52491
IRGM	Immunity Related GTPase M	2.52476
CHM	CHM Rab Escort Protein	2.52398

CYP2W1	Cytochrome P450 Family 2 Subfamily W Member 1	2.52256
ELF4	E74 Like ETS Transcription Factor 4	2.52230
GRM4	Glutamate Metabotropic Receptor 4	2.52122
GORASP1	Golgi Reassembly Stacking Protein 1	2.52110
TMSB10	Thymosin Beta 10	2.52085
LINC00319	Long Intergenic Non-Protein Coding RNA 319	2.52061
PCDH7	Protocadherin 7	2.51934
PHETA1	PH Domain Containing Endocytic Trafficking Adaptor 1	2.51784
VPS33B	VPS33B Late Endosome And Lysosome Associated	2.51669
RCAN1	Regulator Of Calcineurin 1	2.51662
RHOXF2	Rhox Homeobox Family Member 2	2.51621
DAD1	Defender Against Cell Death 1	2.51576
CNOT8	CCR4-NOT Transcription Complex Subunit 8	2.51492
NSMCE1	NSE1 Homolog, SMC5-SMC6 Complex Component	2.51489
ODF3	Outer Dense Fiber Of Sperm Tails 3	2.51414
CA5A	Carbonic Anhydrase 5A	2.51378
CYP2R1	Cytochrome P450 Family 2 Subfamily R Member 1	2.51232
PPP2R5A	Protein Phosphatase 2 Regulatory Subunit B'Alpha	2.51201
MYT1L	Myelin Transcription Factor 1 Like	2.51166
CLIC6	Chloride Intracellular Channel 6	2.51107
ORAI3	ORAI Calcium Release-Activated Calcium Modulator 3	2.51063
LOC10192812(	Uncharacterized LOC101928120	2.51040
RAB5A	RAB5A, Member RAS Oncogene Family	2.50999
HNRNPC	Heterogeneous Nuclear Ribonucleoprotein C	2.50923
RBM23	RNA Binding Motif Protein 23	2.50874
AHCTF1	AT-Hook Containing Transcription Factor 1	2.50866
SPTLC1	Serine Palmitoyltransferase Long Chain Base Subunit 1	2.50799
PURG	Purine Rich Element Binding Protein G	2.50787
ENSG00000228363		2.50758
WASHC5	WASH Complex Subunit 5	2.50756
SP110	SP110 Nuclear Body Protein	2.50554
MANF	Mesencephalic Astrocyte Derived Neurotrophic Factor	2.50546
OTUD5	OTU Deubiquitinase 5	2.50537
DRD1	Dopamine Receptor D1	2.50443

NCR3	Natural Cytotoxicity Triggering Receptor 3	2.50351
ZMPSTE24	Zinc Metallopeptidase STE24	2.50322
RFLNA	Refilin A	2.50180
MND1	Meiotic Nuclear Divisions 1	2.50171
KRT24	Keratin 24	2.50143
CHD3	Chromodomain Helicase DNA Binding Protein 3	2.50125
FNBP4	Formin Binding Protein 4	2.50091
FXR1	FMR1 Autosomal Homolog 1	2.50082
ERVK-6	Endogenous Retrovirus Group K Member 6, Envelope	2.50029
SETD7	SET Domain Containing 7, Histone Lysine Methyltransferase	2.49983
GUCY2D	Guanylate Cyclase 2D, Retinal	2.49780
MED15	Mediator Complex Subunit 15	2.49767
INTS11	Integrator Complex Subunit 11	2.49737
LFS3	Li-Fraumeni Syndrome 3	2.49649
CACUL1	CDK2 Associated Cullin Domain 1	2.49644
UROS	Uroporphyrinogen III Synthase	2.49610
ANTXR2	ANTXR Cell Adhesion Molecule 2	2.49587
NFKBIE	NFKB Inhibitor Epsilon	2.49540
SLC12A1	Solute Carrier Family 12 Member 1	2.49517
SFRP4	Secreted Frizzled Related Protein 4	2.49485
MYO18A	Myosin XVIIIA	2.49374
VAC14	VAC14 Component Of PIKFYVE Complex	2.49356
CYB5D2	Cytochrome B5 Domain Containing 2	2.49344
ERRFI1	ERBB Receptor Feedback Inhibitor 1	2.49319
YPEL2	Yippee Like 2	2.49276
ATP6V0A2	ATPase H+ Transporting V0 Subunit A2	2.49186
FBXO30	F-Box Protein 30	2.49064
GUCY2F	Guanylate Cyclase 2F, Retinal	2.49048
CLN8	CLN8 Transmembrane ER And ERGIC Protein	2.48994
MAN2C1	Mannosidase Alpha Class 2C Member 1	2.48968
ABCF1	ATP Binding Cassette Subfamily F Member 1	2.48953
RAB11FIP1	RAB11 Family Interacting Protein 1	2.48933
EPM2A	EPM2A Glucan Phosphatase, Laforin	2.48930
EMP1	Epithelial Membrane Protein 1	2.48908
NAAA	N-Acylethanolamine Acid Amidase	2.48885
ITIH5	Inter-Alpha-Trypsin Inhibitor Heavy Chain 5	2.48848
CCDC57	Coiled-Coil Domain Containing 57	2.48813
CHMP2B	Charged Multivesicular Body Protein 2B	2.48798

ITIH4	Inter-Alpha-Trypsin Inhibitor Heavy Chain 4	2.48773
VKORC1	Vitamin K Epoxide Reductase Complex Subunit 1	2.48767
CDK2AP2	Cyclin Dependent Kinase 2 Associated Protein 2	2.48732
TMEM106A	Transmembrane Protein 106A	2.48680
TSPAN7	Tetraspanin 7	2.48570
BABAM1	BRISC And BRCA1 A Complex Member 1	2.48564
MIR18B	MicroRNA 18b	2.48372
FAM167A	Family With Sequence Similarity 167 Member A	2.48360
HNRNPD	Heterogeneous Nuclear Ribonucleoprotein D	2.48326
GM2A	GM2 Ganglioside Activator	2.48229
CEP131	Centrosomal Protein 131	2.48221
DNAH11	Dynein Axonemal Heavy Chain 11	2.48146
NR1I3	Nuclear Receptor Subfamily 1 Group I Member 3	2.48100
METTL4	Methyltransferase Like 4	2.48099
PINK1	PTEN Induced Kinase 1	2.48060
NUP62	Nucleoporin 62	2.48028
RAB17	RAB17, Member RAS Oncogene Family	2.48023
GLS2	Glutaminase 2	2.47955
DENND2B	DENN Domain Containing 2B	2.47909
SETX	Senataxin	2.47825
GTF3C1	General Transcription Factor IIIC Subunit 1	2.47824
SEPTIN2	Septin 2	2.47822
GBF1	Golgi Brefeldin A Resistant Guanine Nucleotide Exchange Factor 1	2.47717
SLC12A2	Solute Carrier Family 12 Member 2	2.47685
GPHN	Gephyrin	2.47638
ANLN	Anillin Actin Binding Protein	2.47622
C1QTNF4	C1q And TNF Related 4	2.47600
DKK4	Dickkopf WNT Signaling Pathway Inhibitor 4	2.47575
SFXN2	Sideroflexin 2	2.47435
WDR73	WD Repeat Domain 73	2.47389
BDKRB2	Bradykinin Receptor B2	2.47349
SERPINA6	Serpin Family A Member 6	2.47322
ASH2L	ASH2 Like, Histone Lysine Methyltransferase Complex Subunit	2.47285
YARS1	Tyrosyl-TRNA Synthetase 1	2.47246
CDR2L	Cerebellar Degeneration Related Protein 2 Like	2.47192
SULT2B1	Sulfotransferase Family 2B Member 1	2.47146

STK32C	Serine/Threonine Kinase 32C	2.47084
SULT1A2	Sulfotransferase Family 1A Member 2	2.47028
B4GALNT2	Beta-1,4-N-Acetyl-Galactosaminyltransferase 2	2.46842
TP53INP2	Tumor Protein P53 Inducible Nuclear Protein 2	2.46820
KRT81	Keratin 81	2.46726
CPSF3	Cleavage And Polyadenylation Specific Factor 3	2.46675
TACR3	Tachykinin Receptor 3	2.46549
VMP1	Vacuole Membrane Protein 1	2.46464
CHRNB1	Cholinergic Receptor Nicotinic Beta 1 Subunit	2.46332
RUFY1	RUN And FYVE Domain Containing 1	2.46091
P3H4	Prolyl 3-Hydroxylase Family Member 4 (Inactive)	2.46032
YJU2	YJU2 Splicing Factor Homolog	2.45997
ARHGAP1	Rho GTPase Activating Protein 1	2.45839
PLXNB2	Plexin B2	2.45822
MTF1	Metal Regulatory Transcription Factor 1	2.45820
SLC5A2	Solute Carrier Family 5 Member 2	2.45803
MIR1271	MicroRNA 1271	2.45771
RSPH14	Radial Spoke Head 14 Homolog	2.45746
COMMD1	Copper Metabolism Domain Containing 1	2.45687
IFT172	Intraflagellar Transport 172	2.45681
ACP2	Acid Phosphatase 2, Lysosomal	2.45653
TOR2A	Torsin Family 2 Member A	2.45477
SEC61G	SEC61 Translocon Subunit Gamma	2.45372
CRHR2	Corticotropin Releasing Hormone Receptor 2	2.45343
MED23	Mediator Complex Subunit 23	2.45186
ACSF2	Acyl-CoA Synthetase Family Member 2	2.45177
HLA-DRB3	Major Histocompatibility Complex, Class II, DR Beta 3	2.45071
EEF1B2	Eukaryotic Translation Elongation Factor 1 Beta 2	2.45051
SENP1	SUMO Specific Peptidase 1	2.44927
CLPS	Colipase	2.44875
BAG5	BAG Cochaperone 5	2.44743
AGPAT1	1-Acylglycerol-3-Phosphate O-Acyltransferase 1	2.44686
TCEA3	Transcription Elongation Factor A3	2.44677
GRM7	Glutamate Metabotropic Receptor 7	2.44588
KIF23	Kinesin Family Member 23	2.44552
ARFIP2	ADP Ribosylation Factor Interacting Protein 2	2.44516
RAB1A	RAB1A, Member RAS Oncogene Family	2.44449

CORO1A	Coronin 1A	2.44434
lnc-ZFP36L1-9		2.44422
ARIH2	Ariadne RBR E3 Ubiquitin Protein Ligase 2	2.44378
KLF12	Kruppel Like Factor 12	2.44364
MRNIP	MRN Complex Interacting Protein	2.44272
TRIM4	Tripartite Motif Containing 4	2.44243
HLA-DMB	Major Histocompatibility Complex, Class II, DM Beta	2.44178
MIR3677	MicroRNA 3677	2.44094
PSTPIP1	Proline-Serine-Threonine Phosphatase Interacting Protein 1	2.44088
NXF1	Nuclear RNA Export Factor 1	2.43969
PITPNM2	Phosphatidylinositol Transfer Protein Membrane Associated 2	2.43938
SCN2A	Sodium Voltage-Gated Channel Alpha Subunit 2	2.43933
GMPR2	Guanosine Monophosphate Reductase 2	2.43855
RARRES2	Retinoic Acid Receptor Responder 2	2.43749
SLC26A10	Solute Carrier Family 26 Member 10	2.43716
RASGRP4	RAS Guanyl Releasing Protein 4	2.43682
ETNK1	Ethanolamine Kinase 1	2.43669
UTP4	UTP4 Small Subunit Processome Component	2.43668
S1PR3	Sphingosine-1-Phosphate Receptor 3	2.43618
LOC10050554[illegible]	Uncharacterized LOC100505549	2.43564
SHC4	SHC Adaptor Protein 4	2.43553
SPRY1	Sprouty RTK Signaling Antagonist 1	2.43502
TRIM7	Tripartite Motif Containing 7	2.43406
TANC2	Tetratricopeptide Repeat, Ankyrin Repeat And Coiled-Coil Containing 2	2.43319
DHX30	DExH-Box Helicase 30	2.43281
CROCC	Ciliary Rootlet Coiled-Coil, Rootletin	2.43266
PVR	PVR Cell Adhesion Molecule	2.43237
TUBGCP2	Tubulin Gamma Complex Associated Protein 2	2.43232
STK11IP	Serine/Threonine Kinase 11 Interacting Protein	2.43056
ODF1	Outer Dense Fiber Of Sperm Tails 1	2.43054
B3GNTL1	UDP-GlcNAc:BetaGal Beta-1,3-N-Acetylglucosaminyltransferase Like 1	2.43040
SIX3	SIX Homeobox 3	2.43015
FGR	FGR Proto-Oncogene, Src Family Tyrosine Kinase	2.43006
PRPH2	Peripherin 2	2.42961
SLC22A16	Solute Carrier Family 22 Member 16	2.42889
VARS2	Valyl-TRNA Synthetase 2, Mitochondrial	2.42869

TOMM40	Translocase Of Outer Mitochondrial Membrane 40	2.42843
TMPRSS9	Transmembrane Serine Protease 9	2.42768
ST3GAL4	ST3 Beta-Galactoside Alpha-2,3-Sialyltransferase 4	2.42761
GSPT1	G1 To S Phase Transition 1	2.42750
MYMK	Myomaker, Myoblast Fusion Factor	2.42719
ADD3	Adducin 3	2.42640
ATAD3A	ATPase Family AAA Domain Containing 3A	2.42636
ASIC1	Acid Sensing Ion Channel Subunit 1	2.42604
NUP93	Nucleoporin 93	2.42580
SYNE2	Spectrin Repeat Containing Nuclear Envelope Protein 2	2.42537
ENSG00000272950		2.42466
LMTK3	Lemur Tyrosine Kinase 3	2.42307
MAFG	MAF BZIP Transcription Factor G	2.42304
POLDIP3	DNA Polymerase Delta Interacting Protein 3	2.42147
DEFA6	Defensin Alpha 6	2.42066
FOXI1	Forkhead Box I1	2.42023
WEE2	WEE2 Oocyte Meiosis Inhibiting Kinase	2.42008
RPS8	Ribosomal Protein S8	2.41977
PTAFR	Platelet Activating Factor Receptor	2.41919
ACSS1	Acyl-CoA Synthetase Short Chain Family Member 1	2.41836
LINC00593	Long Intergenic Non-Protein Coding RNA 593	2.41782
FCN1	Ficolin 1	2.41727
CHCHD10	Coiled-Coil-Helix-Coiled-Coil-Helix Domain Containing 10	2.41705
MYRF	Myelin Regulatory Factor	2.41664
KIFC1	Kinesin Family Member C1	2.41623
LGALS7B	Galectin 7B	2.41617
DGKD	Diacylglycerol Kinase Delta	2.41600
GGCT	Gamma-Glutamylcyclotransferase	2.41585
CCL25	C-C Motif Chemokine Ligand 25	2.41546
MIR194-2	MicroRNA 194-2	2.41534
PWWP3A	PWWP Domain Containing 3A, DNA Repair Factor	2.41528
BOD1	Biorientation Of Chromosomes In Cell Division 1	2.41508
WFIKKN2	WAP, Follistatin/Kazal, Immunoglobulin, Kunitz And Netrin Domain Containing 2	2.41494
HYAL3	Hyaluronidase 3	2.41384
EXD3	Exonuclease 3'-5' Domain Containing 3	2.41372
INHBE	Inhibin Subunit Beta E	2.41256
PYGB	Glycogen Phosphorylase B	2.41250

GCOM1	GCOM1, MYZAP-POLR2M Combined Locus	2.41243
NMRAL1	NmrA Like Redox Sensor 1	2.41187
ZNF45	Zinc Finger Protein 45	2.41183
KDM5D	Lysine Demethylase 5D	2.41164
PNPLA6	Patatin Like Phospholipase Domain Containing 6	2.41142
OPRD1	Opioid Receptor Delta 1	2.41109
MIR1469	MicroRNA 1469	2.40996
HNRNPR	Heterogeneous Nuclear Ribonucleoprotein R	2.40964
TDGF1P3	Teratocarcinoma-Derived Growth Factor 1 Pseudogene 3	2.40960
PPP3CC	Protein Phosphatase 3 Catalytic Subunit Gamma	2.40922
PNKP	Polynucleotide Kinase 3'-Phosphatase	2.40688
FBXO46	F-Box Protein 46	2.40637
DNAI1	Dynein Axonemal Intermediate Chain 1	2.40527
B3GNT8	UDP-GlcNAc:BetaGal Beta-1,3-N-Acetylglucosaminyltransferase 8	2.40519
MSTN	Myostatin	2.40510
CA11	Carbonic Anhydrase 11	2.40468
RPL29	Ribosomal Protein L29	2.40439
HMCN1	Hemicentin 1	2.40345
ACBD3	Acyl-CoA Binding Domain Containing 3	2.40321
ATP5PD	ATP Synthase Peripheral Stalk Subunit D	2.40317
BABAM2	BRISC And BRCA1 A Complex Member 2	2.40280
KRT76	Keratin 76	2.40278
CDC42EP4	CDC42 Effector Protein 4	2.40228
TLCD3A	TLC Domain Containing 3A	2.40207
PRM2	Protamine 2	2.40197
CCR8	C-C Motif Chemokine Receptor 8	2.40195
SAMD14	Sterile Alpha Motif Domain Containing 14	2.40145
TPRA1	Transmembrane Protein Adipocyte Associated 1	2.40142
TRIB2	Tribbles Pseudokinase 2	2.40089
TAF11	TATA-Box Binding Protein Associated Factor 11	2.40070
RUVBL2	RuvB Like AAA ATPase 2	2.39991
ARL4C	ADP Ribosylation Factor Like GTPase 4C	2.39917
RPL8	Ribosomal Protein L8	2.39876
FHL3	Four And A Half LIM Domains 3	2.39874
PTPRF	Protein Tyrosine Phosphatase Receptor Type F	2.39855

MOK	MOK Protein Kinase	2.39789
PSMB4	Proteasome 20S Subunit Beta 4	2.39741
DDX56	DEAD-Box Helicase 56	2.39655
COL13A1	Collagen Type XIII Alpha 1 Chain	2.39603
TPSAB1	Tryptase Alpha/Beta 1	2.39594
NRARP	NOTCH Regulated Ankyrin Repeat Protein	2.39559
RAB11A	RAB11A, Member RAS Oncogene Family	2.39558
CREBRF	CREB3 Regulatory Factor	2.39512
ENPP3	Ectonucleotide Pyrophosphatase/Phosphodiesterase 3	2.39509
CLDN5	Claudin 5	2.39376
DENND2D	DENN Domain Containing 2D	2.39354
HEXD	Hexosaminidase D	2.39349
SOGA1	Suppressor Of Glucose, Autophagy Associated 1	2.39316
M6PR	Mannose-6-Phosphate Receptor, Cation Dependent	2.39219
ENSG00000267152		2.39188
PHGDH	Phosphoglycerate Dehydrogenase	2.39143
SLC11A2	Solute Carrier Family 11 Member 2	2.39117
ARAP1	ArfGAP With RhoGAP Domain, Ankyrin Repeat And PH Domain 1	2.39031
RPL9	Ribosomal Protein L9	2.38956
GRB14	Growth Factor Receptor Bound Protein 14	2.38924
CALCOCO1	Calcium Binding And Coiled-Coil Domain 1	2.38909
CCT5	Chaperonin Containing TCP1 Subunit 5	2.38899
UBXN6	UBX Domain Protein 6	2.38889
SLC25A25	Solute Carrier Family 25 Member 25	2.38829
DNALI1	Dynein Axonemal Light Intermediate Chain 1	2.38821
ARHGAP21	Rho GTPase Activating Protein 21	2.38779
PBX2	PBX Homeobox 2	2.38739
ZNF236	Zinc Finger Protein 236	2.38721
SUMO3	Small Ubiquitin Like Modifier 3	2.38699
MDFIC2	MyoD Family Inhibitor Domain Containing 2	2.38697
RAB20	RAB20, Member RAS Oncogene Family	2.38634
BCL6B	BCL6B Transcription Repressor	2.38509
C1orf116	Chromosome 1 Open Reading Frame 116	2.38506
ESRP1	Epithelial Splicing Regulatory Protein 1	2.38394
ADAM8	ADAM Metallopeptidase Domain 8	2.38368
CD27-AS1	CD27 Antisense RNA 1	2.38337
C9orf43	Chromosome 9 Open Reading Frame 43	2.38312

ZFAND2A	Zinc Finger AN1-Type Containing 2A	2.38312
GRAMD4	GRAM Domain Containing 4	2.38264
EIF2D	Eukaryotic Translation Initiation Factor 2D	2.38075
DDX59	DEAD-Box Helicase 59	2.38029
ADCY5	Adenylate Cyclase 5	2.38008
BZW1	Basic Leucine Zipper And W2 Domains 1	2.37937
ADAM23	ADAM Metallopeptidase Domain 23	2.37850
MYL12B	Myosin Light Chain 12B	2.37846
PRR11	Proline Rich 11	2.37818
NDRG3	NDRG Family Member 3	2.37797
CTDSP1	CTD Small Phosphatase 1	2.37774
RPS5	Ribosomal Protein S5	2.37705
N4BP2	NEDD4 Binding Protein 2	2.37618
CD207	CD207 Molecule	2.37561
SSBP3-AS1	SSBP3 Antisense RNA 1	2.37554
SRP68	Signal Recognition Particle 68	2.37399
TCF19	Transcription Factor 19	2.37376
SF3A3	Splicing Factor 3a Subunit 3	2.37365
RSRC2	Arginine And Serine Rich Coiled-Coil 2	2.37060
NBPF20	NBPF Member 20	2.36947
ANAPC2	Anaphase Promoting Complex Subunit 2	2.36942
XRRA1	X-Ray Radiation Resistance Associated 1	2.36930
GPS2	G Protein Pathway Suppressor 2	2.36926
TDP1	Tyrosyl-DNA Phosphodiesterase 1	2.36893
GRWD1	Glutamate Rich WD Repeat Containing 1	2.36890
ZBTB8B	Zinc Finger And BTB Domain Containing 8B	2.36878
MFSD13A	Major Facilitator Superfamily Domain Containing 13A	2.36873
RFT1	RFT1 Homolog	2.36863
ING2	Inhibitor Of Growth Family Member 2	2.36791
ITLN1	Intelectin 1	2.36780
CTDSP2	CTD Small Phosphatase 2	2.36744
LINC00513	Long Intergenic Non-Protein Coding RNA 513	2.36640
GTF3C5	General Transcription Factor IIIC Subunit 5	2.36625
PRCD	Photoreceptor Disc Component	2.36590
GOLPH3L	Golgi Phosphoprotein 3 Like	2.36571
SMG6	SMG6 Nonsense Mediated MRNA Decay Factor	2.36550
ZFPL1	Zinc Finger Protein Like 1	2.36550
CERS2	Ceramide Synthase 2	2.36541
KLF1	Kruppel Like Factor 1	2.36484

FGG	Fibrinogen Gamma Chain	2.36351
PHLDA3	Pleckstrin Homology Like Domain Family A Member 3	2.36350
SRP14	Signal Recognition Particle 14	2.36274
MVB12A	Multivesicular Body Subunit 12A	2.36245
PCDH8	Protocadherin 8	2.36222
COPG2	COPI Coat Complex Subunit Gamma 2	2.36206
USP5	Ubiquitin Specific Peptidase 5	2.36181
DEFB103B	Defensin Beta 103B	2.36158
VEZF1	Vascular Endothelial Zinc Finger 1	2.36098
CCDC40	Coiled-Coil Domain Containing 40	2.36019
PRR13	Proline Rich 13	2.35974
CLEC16A	C-Type Lectin Domain Containing 16A	2.35966
HRK	Harakiri, BCL2 Interacting Protein	2.35962
ERH	ERH MRNA Splicing And Mitosis Factor	2.35881
ADCY2	Adenylate Cyclase 2	2.35872
RNU1-103P	RNA, U1 Small Nuclear 103, Pseudogene	2.35854
lnc-PAX8-2		2.35854
MIR1976	MicroRNA 1976	2.35755
SLC22A1	Solute Carrier Family 22 Member 1	2.35670
DSN1	DSN1 Component Of MIS12 Kinetochore Complex	2.35651
SMOC2	SPARC Related Modular Calcium Binding 2	2.35605
PFDN5	Prefoldin Subunit 5	2.35557
MYZAP	Myocardial Zonula Adherens Protein	2.35549
TRIB1	Tribbles Pseudokinase 1	2.35547
BCRP3	BCR Pseudogene 3	2.35541
BAZ2A	Bromodomain Adjacent To Zinc Finger Domain 2A	2.35533
DOCK3	Dedicator Of Cytokinesis 3	2.35509
WDR82	WD Repeat Domain 82	2.35505
MAP4K1	Mitogen-Activated Protein Kinase Kinase Kinase Kinase 1	2.35448
GRIK3	Glutamate Ionotropic Receptor Kainate Type Subunit 3	2.35415
PARVA	Parvin Alpha	2.35391
NKX6-3	NK6 Homeobox 3	2.35385
DCAF7	DDB1 And CUL4 Associated Factor 7	2.35365
DDX11-AS1	DDX11 Antisense RNA 1	2.35316
EFNA5	Ephrin A5	2.35273
RNH1	Ribonuclease/Angiogenin Inhibitor 1	2.35265
PRKCZ-AS1	PRKCZ Antisense RNA 1	2.35217
KIF3A	Kinesin Family Member 3A	2.35172
ADPRH	ADP-Ribosylarginine Hydrolase	2.35136
FHAD1	Forkhead Associated Phosphopeptide Binding Domain 1	2.35136

PIGV	Phosphatidylinositol Glycan Anchor Biosynthesis Class V	2.35068
NANOS3	Nanos C2HC-Type Zinc Finger 3	2.35059
H4C15	H4 Clustered Histone 15	2.34982
RNF7	Ring Finger Protein 7	2.34980
ABTB1	Ankyrin Repeat And BTB Domain Containing 1	2.34957
TLE3	TLE Family Member 3, Transcriptional Corepressor	2.34772
LDC1P	Leucine Decarboxylase 1, Pseudogene	2.34761
LETM1	Leucine Zipper And EF-Hand Containing Transmembrane Protein 1	2.34739
NINJ2	Ninjurin 2	2.34709
METTL23	Methyltransferase Like 23	2.34709
KIDINS220	Kinase D Interacting Substrate 220	2.34632
CEBPZ	CCAAT Enhancer Binding Protein Zeta	2.34616
UPF3A	UPF3A Regulator Of Nonsense Mediated MRNA Decay	2.34583
PDS5A	PDS5 Cohesin Associated Factor A	2.34483
CPB2	Carboxypeptidase B2	2.34469
STAG3L2	Stromal Antigen 3-Like 2 (Pseudogene)	2.34465
NMI	N-Myc And STAT Interactor	2.34389
ARID3B	AT-Rich Interaction Domain 3B	2.34382
MAPRE3	Microtubule Associated Protein RP/EB Family Member 3	2.34369
RTN4IP1	Reticulon 4 Interacting Protein 1	2.34361
SELPLG	Selectin P Ligand	2.34324
DEF6	DEF6 Guanine Nucleotide Exchange Factor	2.34320
MIR613	MicroRNA 613	2.34183
CD48	CD48 Molecule	2.34150
OGFRP1	Opioid Growth Factor Receptor Pseudogene 1	2.34050
SPAG4	Sperm Associated Antigen 4	2.33967
PSMB2	Proteasome 20S Subunit Beta 2	2.33959
YIF1B	Yip1 Interacting Factor Homolog B, Membrane Trafficking Protein	2.33959
MFF	Mitochondrial Fission Factor	2.33904
PGS1	Phosphatidylglycerophosphate Synthase 1	2.33898
MCF2L	MCF.2 Cell Line Derived Transforming Sequence Like	2.33855
PTGR2	Prostaglandin Reductase 2	2.33837
TBC1D2B	TBC1 Domain Family Member 2B	2.33822
HTR2C	5-Hydroxytryptamine Receptor 2C	2.33778
ALG13	ALG13 UDP-N-Acetylglucosaminyltransferase Subunit	2.33637
TRAPPC9	Trafficking Protein Particle Complex Subunit 9	2.33562

ZNF283	Zinc Finger Protein 283	2.33548
TFAP2B	Transcription Factor AP-2 Beta	2.33473
XCR1	X-C Motif Chemokine Receptor 1	2.33405
MGST2	Microsomal Glutathione S-Transferase 2	2.33378
MBD6	Methyl-CpG Binding Domain Protein 6	2.33320
ZNF223	Zinc Finger Protein 223	2.33315
C19orf44	Chromosome 19 Open Reading Frame 44	2.33217
ANO9	Anoctamin 9	2.33135
TOLLIP	Toll Interacting Protein	2.33125
MRPL11	Mitochondrial Ribosomal Protein L11	2.33103
GMPPB	GDP-Mannose Pyrophosphorylase B	2.33076
DAO	D-Amino Acid Oxidase	2.33062
ACOX1	Acyl-CoA Oxidase 1	2.33044
PDE4D	Phosphodiesterase 4D	2.33008
ZNF740	Zinc Finger Protein 740	2.32967
RASGRF1	Ras Protein Specific Guanine Nucleotide Releasing Factor 1	2.32886
ARHGEF37	Rho Guanine Nucleotide Exchange Factor 37	2.32751
GNB4	G Protein Subunit Beta 4	2.32750
MPP1	Membrane Palmitoylated Protein 1	2.32747
ZFP30	ZFP30 Zinc Finger Protein	2.32677
ZWILCH	Zwilch Kinetochore Protein	2.32622
VPS37B	VPS37B Subunit Of ESCRT-I	2.32601
FABP5P3	Fatty Acid Binding Protein 5 Pseudogene 3	2.32494
EGILA	EGFR Interacting LncRNA	2.32494
PITRM1	Pitrilysin Metallopeptidase 1	2.32490
SLC1A2	Solute Carrier Family 1 Member 2	2.32398
CES1	Carboxylesterase 1	2.32391
GSTZ1	Glutathione S-Transferase Zeta 1	2.32322
KPNB1	Karyopherin Subunit Beta 1	2.32296
SNRPD3	Small Nuclear Ribonucleoprotein D3 Polypeptide	2.32266
AKR7A3	Aldo-Keto Reductase Family 7 Member A3	2.32192
ANKLE2	Ankyrin Repeat And LEM Domain Containing 2	2.32184
CDK5RAP1	CDK5 Regulatory Subunit Associated Protein 1	2.32171
IHO1	Interactor Of HORMAD1 1	2.32111
DCBLD2	Discoidin, CUB And LCCL Domain Containing 2	2.32059
ILKAP	ILK Associated Serine/Threonine Phosphatase	2.32023
NTF4	Neurotrophin 4	2.31955
SLC30A5	Solute Carrier Family 30 Member 5	2.31882
SRCAP	Snf2 Related CREBBP Activator Protein	2.31868

TPP1	Tripeptidyl Peptidase 1	2.31862
PPIH	Peptidylprolyl Isomerase H	2.31828
USP3	Ubiquitin Specific Peptidase 3	2.31732
SF1	Splicing Factor 1	2.31723
CBX8	Chromobox 8	2.31720
TMEM120A	Transmembrane Protein 120A	2.31698
ODAD1	Outer Dynein Arm Docking Complex Subunit 1	2.31670
ATP2C2	ATPase Secretory Pathway Ca2+ Transporting 2	2.31668
CLIC4	Chloride Intracellular Channel 4	2.31568
FAM102A	Family With Sequence Similarity 102 Member A	2.31477
UBE4B	Ubiquitination Factor E4B	2.31461
SPESP1	Sperm Equatorial Segment Protein 1	2.31450
KIAA1109	KIAA1109	2.31400
PEX16	Peroxisomal Biogenesis Factor 16	2.31385
TMEM220	Transmembrane Protein 220	2.31376
OMA1	OMA1 Zinc Metallopeptidase	2.31358
SLC39A3	Solute Carrier Family 39 Member 3	2.31350
TPH2	Tryptophan Hydroxylase 2	2.31269
SMG9	SMG9 Nonsense Mediated MRNA Decay Factor	2.31231
TRIM66	Tripartite Motif Containing 66	2.31227
GNAZ	G Protein Subunit Alpha Z	2.31226
FYCO1	FYVE And Coiled-Coil Domain Autophagy Adaptor 1	2.31106
MIR548C	MicroRNA 548c	2.31087
C11orf24	Chromosome 11 Open Reading Frame 24	2.31054
ISYNA1	Inositol-3-Phosphate Synthase 1	2.31050
MAVS	Mitochondrial Antiviral Signaling Protein	2.31036
SFI1	SFI1 Centrin Binding Protein	2.31024
PIGQ	Phosphatidylinositol Glycan Anchor Biosynthesis Class Q	2.31022
SUN2	Sad1 And UNC84 Domain Containing 2	2.31006
DAGLB	Diacylglycerol Lipase Beta	2.30920
EIF3K	Eukaryotic Translation Initiation Factor 3 Subunit K	2.30907
MTG2	Mitochondrial Ribosome Associated GTPase 2	2.30900
TMEM161A	Transmembrane Protein 161A	2.30795
H4C14	H4 Clustered Histone 14	2.30764
HOXA7	Homeobox A7	2.30712
GOLT1A	Golgi Transport 1A	2.30678
SND1	Staphylococcal Nuclease And Tudor Domain Containing 1	2.30646
TTC3	Tetratricopeptide Repeat Domain 3	2.30639

COG2	Component Of Oligomeric Golgi Complex 2	2.30587
REC8	REC8 Meiotic Recombination Protein	2.30570
THOC5	THO Complex 5	2.30545
LAD1	Ladinin 1	2.30544
OXER1	Oxoeicosanoid Receptor 1	2.30402
EAPP	E2F Associated Phosphoprotein	2.30284
NBEA	Neurobeachin	2.30258
KLHL36	Kelch Like Family Member 36	2.30160
SH2B1	SH2B Adaptor Protein 1	2.30148
ZDHHC12	Zinc Finger DHHC-Type Palmitoyltransferase 12	2.30099
SECISBP2	SECIS Binding Protein 2	2.30094
KRT83	Keratin 83	2.30081
VIRMA	Vir Like M6A Methyltransferase Associated	2.30018
SMG7	SMG7 Nonsense Mediated MRNA Decay Factor	2.30008
GRK4	G Protein-Coupled Receptor Kinase 4	2.29870
SOX21	SRY-Box Transcription Factor 21	2.29848
AFAP1L1	Actin Filament Associated Protein 1 Like 1	2.29829
GOLGA3	Golgin A3	2.29787
RPS11	Ribosomal Protein S11	2.29780
MYCBP	MYC Binding Protein	2.29780
PACC1	Proton Activated Chloride Channel 1	2.29608
ACAD9	Acyl-CoA Dehydrogenase Family Member 9	2.29539
ATP6V1A	ATPase H+ Transporting V1 Subunit A	2.29505
MELTF	Melanotransferrin	2.29504
HOOK2	Hook Microtubule Tethering Protein 2	2.29433
SIAH1	Siah E3 Ubiquitin Protein Ligase 1	2.29405
SP4	Sp4 Transcription Factor	2.29345
PTOV1	PTOV1 Extended AT-Hook Containing Adaptor Protein	2.29335
SLC16A5	Solute Carrier Family 16 Member 5	2.29309
TCP10L	T-Complex 10 Like	2.29265
GPATCH3	G-Patch Domain Containing 3	2.29168
PLEKHG6	Pleckstrin Homology And RhoGEF Domain Containing G6	2.29149
LEFTY1	Left-Right Determination Factor 1	2.29109
KCNK5	Potassium Two Pore Domain Channel Subfamily K Member 5	2.29102
FBXO47	F-Box Protein 47	2.28968
LOC11108994	GSDMB 5' Regulatory Region	2.28946
TNFAIP8	TNF Alpha Induced Protein 8	2.28934
AMOT	Angiomotin	2.28833
PAAF1	Proteasomal ATPase Associated Factor 1	2.28776
OAZ1	Ornithine Decarboxylase Antizyme 1	2.28753

QRICH1	Glutamine Rich 1	2.28731
ZNF274	Zinc Finger Protein 274	2.28622
lnc-CHD9-4		2.28601
MTSS2	MTSS I-BAR Domain Containing 2	2.28580
TMEM92	Transmembrane Protein 92	2.28546
STX1A	Syntaxin 1A	2.28543
EHHADH	Enoyl-CoA Hydratase And 3-Hydroxyacyl CoA Dehydrogenase	2.28532
BMP5	Bone Morphogenetic Protein 5	2.28525
CPSF7	Cleavage And Polyadenylation Specific Factor 7	2.28525
INPP5K	Inositol Polyphosphate-5-Phosphatase K	2.28488
IMPA2	Inositol Monophosphatase 2	2.28466
COPRS	Coordinator Of PRMT5 And Differentiation Stimulator	2.28451
ZNF618	Zinc Finger Protein 618	2.28402
FAM47E	Family With Sequence Similarity 47 Member E	2.28398
OXSR1	Oxidative Stress Responsive Kinase 1	2.28367
LSM7	LSM7 Homolog, U6 Small Nuclear RNA And MRNA Degradation Associated	2.28364
EFNA2	Ephrin A2	2.28346
DRG2	Developmentally Regulated GTP Binding Protein 2	2.28338
WDR97	WD Repeat Domain 97	2.28336
NRF1	Nuclear Respiratory Factor 1	2.28329
DDOST	Dolichyl-Diphosphooligosaccharide--Protein Glycosyltransferase Non-Catalytic Subunit	2.28295
RGS22	Regulator Of G Protein Signaling 22	2.28281
SEM1	SEM1 26S Proteasome Subunit	2.28265
PLB1	Phospholipase B1	2.28200
CACNG5	Calcium Voltage-Gated Channel Auxiliary Subunit Gamma 5	2.28198
LAMTOR2	Late Endosomal/Lysosomal Adaptor, MAPK And MTOR Activator 2	2.28120
SRRT	Serrate, RNA Effector Molecule	2.28119
SUCLA2	Succinate-CoA Ligase ADP-Forming Subunit Beta	2.28106
DHX40	DEAH-Box Helicase 40	2.28094
CHERP	Calcium Homeostasis Endoplasmic Reticulum Protein	2.28070
SPOCK2	SPARC (Osteonectin), Cwcv And Kazal Like Domains Proteoglycan 2	2.27975
SPTBN4	Spectrin Beta, Non-Erythrocytic 4	2.27954
GPATCH1	G-Patch Domain Containing 1	2.27933
EXOSC10	Exosome Component 10	2.27913

CAP2	Cyclase Associated Actin Cytoskeleton Regulatory Protein 2	2.27883
MRPL20	Mitochondrial Ribosomal Protein L20	2.27877
UTS2R	Urotensin 2 Receptor	2.27862
TMEM116	Transmembrane Protein 116	2.27812
RUSF1	RUS Family Member 1	2.27801
SOX7	SRY-Box Transcription Factor 7	2.27800
ZNF234	Zinc Finger Protein 234	2.27741
ZFYVE9	Zinc Finger FYVE-Type Containing 9	2.27723
LINGO1	Leucine Rich Repeat And Ig Domain Containing 1	2.27717
STIP1	Stress Induced Phosphoprotein 1	2.27706
PRRX2	Paired Related Homeobox 2	2.27686
WDR1	WD Repeat Domain 1	2.27672
CCDC134	Coiled-Coil Domain Containing 134	2.27625
ABLIM1	Actin Binding LIM Protein 1	2.27620
ATG4D	Autophagy Related 4D Cysteine Peptidase	2.27593
SLC26A1	Solute Carrier Family 26 Member 1	2.27566
RFXANK	Regulatory Factor X Associated Ankyrin Containing Protein	2.27542
FMNL1	Formin Like 1	2.27505
COG4	Component Of Oligomeric Golgi Complex 4	2.27450
TMEM63A	Transmembrane Protein 63A	2.27449
AMOTL2	Angiomotin Like 2	2.27432
GUCY1A2	Guanylate Cyclase 1 Soluble Subunit Alpha 2	2.27429
ZNF789	Zinc Finger Protein 789	2.27410
TRIM3	Tripartite Motif Containing 3	2.27406
RNU4ATAC	RNA, U4atac Small Nuclear (U12-Dependent Splicing)	2.27403
GARRE1	Granule Associated Rac And RHOG Effector 1	2.27398
TNPO2	Transportin 2	2.27374
NIP7	Nucleolar Pre-RRNA Processing Protein NIP7	2.27348
SHC2	SHC Adaptor Protein 2	2.27325
NARF	Nuclear Prelamin A Recognition Factor	2.27255
JAGN1	Jagunal Homolog 1	2.27213
RPL39L	Ribosomal Protein L39 Like	2.27150
KCNE5	Potassium Voltage-Gated Channel Subfamily E Regulatory Subunit 5	2.27122
LARP7	La Ribonucleoprotein 7, Transcriptional Regulator	2.27071
PHYHD1	Phytanoyl-CoA Dioxygenase Domain Containing 1	2.27056
RAP1B	RAP1B, Member Of RAS Oncogene Family	2.27055

GNG5	G Protein Subunit Gamma 5	2.27021
RPS2	Ribosomal Protein S2	2.26976
DUOXA1	Dual Oxidase Maturation Factor 1	2.26947
STX11	Syntaxin 11	2.26928
TXLNA	Taxilin Alpha	2.26777
SHB	SH2 Domain Containing Adaptor Protein B	2.26749
B4GALT1	Beta-1,4-Galactosyltransferase 1	2.26719
GALNT6	Polypeptide N-Acetylgalactosaminyltransferase 6	2.26717
CLRN3	Clarin 3	2.26648
LDHD	Lactate Dehydrogenase D	2.26609
PDE2A	Phosphodiesterase 2A	2.26574
SPTBN2	Spectrin Beta, Non-Erythrocytic 2	2.26553
POLE3	DNA Polymerase Epsilon 3, Accessory Subunit	2.26546
H2AZ2	H2A.Z Variant Histone 2	2.26480
GRINA	Glutamate Ionotropic Receptor NMDA Type Subunit Associated Protein 1	2.26247
UBQLN1	Ubiquilin 1	2.26174
EML1	EMAP Like 1	2.26165
SLC25A47	Solute Carrier Family 25 Member 47	2.26114
CCL8	C-C Motif Chemokine Ligand 8	2.26047
RIPK2	Receptor Interacting Serine/Threonine Kinase 2	2.26030
PRRC2B	Proline Rich Coiled-Coil 2B	2.25989
FOXRED2	FAD Dependent Oxidoreductase Domain Containing 2	2.25953
LRRC59	Leucine Rich Repeat Containing 59	2.25951
SZT2	SZT2 Subunit Of KICSTOR Complex	2.25895
SNRPB2	Small Nuclear Ribonucleoprotein Polypeptide B2	2.25852
TNNT3	Troponin T3, Fast Skeletal Type	2.25834
NEDD8	NEDD8 Ubiquitin Like Modifier	2.25692
CCDC112	Coiled-Coil Domain Containing 112	2.25635
TAF6	TATA-Box Binding Protein Associated Factor 6	2.25548
HSALNG0058161		2.25493
RASD1	Ras Related Dexamethasone Induced 1	2.25491
PHRF1	PHD And Ring Finger Domains 1	2.25474
PNPLA2	Patatin Like Phospholipase Domain Containing 2	2.25452
TMEM33	Transmembrane Protein 33	2.25446
TBC1D8	TBC1 Domain Family Member 8	2.25428
MROH7	Maestro Heat Like Repeat Family Member 7	2.25426
ASCC2	Activating Signal Cointegrator 1 Complex Subunit 2	2.25421

GAREM2	GRB2 Associated Regulator Of MAPK1 Subtype 2	2.25296
PUS1	Pseudouridine Synthase 1	2.25285
UACA	Uveal Autoantigen With Coiled-Coil Domains And Ankyrin Repeats	2.25273
RPL23A	Ribosomal Protein L23a	2.25220
SPNS2	Sphingolipid Transporter 2	2.25195
SLAMF7	SLAM Family Member 7	2.25135
POLR1G	RNA Polymerase I Subunit G	2.25126
DDX11	DEAD/H-Box Helicase 11	2.25113
SYT2	Synaptotagmin 2	2.25112
HLA-DOB	Major Histocompatibility Complex, Class II, DO Beta	2.25106
HNRNPH2	Heterogeneous Nuclear Ribonucleoprotein H2	2.25085
LSM14A	LSM14A MRNA Processing Body Assembly Factor	2.25080
SCN8A	Sodium Voltage-Gated Channel Alpha Subunit 8	2.25050
KCNH4	Potassium Voltage-Gated Channel Subfamily H Member 4	2.24947
MYNN	Myoneurin	2.24902
GRHL3	Grainyhead Like Transcription Factor 3	2.24863
GALNT1	Polypeptide N-Acetylgalactosaminyltransferase 1	2.24862
PPIP5K1	Diphosphoinositol Pentakisphosphate Kinase 1	2.24829
PGD	Phosphogluconate Dehydrogenase	2.24770
CTSF	Cathepsin F	2.24626
PIK3IP1	Phosphoinositide-3-Kinase Interacting Protein 1	2.24596
UCN3	Urocortin 3	2.24460
MX2	MX Dynamin Like GTPase 2	2.24392
HSALNG0111460		2.24391
LOC10537126:	Uncharacterized LOC105371265	2.24391
SETD3	SET Domain Containing 3, Actin Histidine Methyltransferase	2.24333
DTX2	Deltex E3 Ubiquitin Ligase 2	2.24321
STX3	Syntaxin 3	2.24284
EHD2	EH Domain Containing 2	2.24255
MFSD11	Major Facilitator Superfamily Domain Containing 11	2.24217
NVL	Nuclear VCP Like	2.24158
MYO9A	Myosin IXA	2.24151
USH2A-AS2	USH2A Antisense RNA 2	2.24099
BARX2	BARX Homeobox 2	2.24053
SMARCD3	SWI/SNF Related, Matrix Associated, Actin Dependent Regulator Of Chromatin, Subfamily D, Member 3	2.23878

EXTL1	Exostosin Like Glycosyltransferase 1	2.23807
LINC00974	Long Intergenic Non-Protein Coding RNA 974	2.23699
RBM12	RNA Binding Motif Protein 12	2.23651
ZBTB38	Zinc Finger And BTB Domain Containing 38	2.23594
ADAMTSL4	ADAMTS Like 4	2.23521
WDR46	WD Repeat Domain 46	2.23460
TTC37	Tetratricopeptide Repeat Domain 37	2.23459
CSRP1	Cysteine And Glycine Rich Protein 1	2.23299
KRI1	KRI1 Homolog	2.23291
NR6A1	Nuclear Receptor Subfamily 6 Group A Member 1	2.23260
PWWP2B	PWWP Domain Containing 2B	2.23240
PNMA1	PNMA Family Member 1	2.23209
SEC63	SEC63 Homolog, Protein Translocation Regulator	2.23203
SLIT1	Slit Guidance Ligand 1	2.23140
PPIL4	Peptidylprolyl Isomerase Like 4	2.23106
MRPL52	Mitochondrial Ribosomal Protein L52	2.23071
PALM3	Paralemmin 3	2.23023
PLXNA2	Plexin A2	2.23013
SNN	Stannin	2.22956
ABHD11	Abhydrolase Domain Containing 11	2.22939
CST1	Cystatin SN	2.22900
TUBA1C	Tubulin Alpha 1c	2.22873
UTP15	UTP15 Small Subunit Processome Component	2.22847
INTS1	Integrator Complex Subunit 1	2.22803
PEAK3	PEAK Family Member 3	2.22770
CISD3	CDGSH Iron Sulfur Domain 3	2.22753
PIAS4	Protein Inhibitor Of Activated STAT 4	2.22735
EFS	Embryonal Fyn-Associated Substrate	2.22725
EPS15L1	Epidermal Growth Factor Receptor Pathway Substrate 15 Like 1	2.22674
SYMPK	Symplekin	2.22659
KLHL25	Kelch Like Family Member 25	2.22659
ZNF227	Zinc Finger Protein 227	2.22589
NUMBL	NUMB Like Endocytic Adaptor Protein	2.22542
USP37	Ubiquitin Specific Peptidase 37	2.22467
SMCR8	SMCR8-C9orf72 Complex Subunit	2.22425
RFX5	Regulatory Factor X5	2.22328
SLC6A1	Solute Carrier Family 6 Member 1	2.22328
MPPE1	Metallophosphoesterase 1	2.22303
PLBD1-AS1	PLBD1 Antisense RNA 1	2.22279
MYOZ3	Myozenin 3	2.22279
MKNK2	MAPK Interacting Serine/Threonine Kinase 2	2.22237
AKNA	AT-Hook Transcription Factor	2.22232

GDI2	GDP Dissociation Inhibitor 2	2.22152
DNAJC7	DnaJ Heat Shock Protein Family (Hsp40) Member C7	2.22139
MAFK	MAF BZIP Transcription Factor K	2.22137
NGDN	Neuroguidin	2.22119
FBXO16	F-Box Protein 16	2.22083
ATP5F1B	ATP Synthase F1 Subunit Beta	2.22068
RAB36	RAB36, Member RAS Oncogene Family	2.21991
EXOSC6	Exosome Component 6	2.21969
IQGAP3	IQ Motif Containing GTPase Activating Protein 3	2.21911
ENSG00000262837		2.21907
ENSG00000270184		2.21907
MN298678-016		2.21907
SAR1A	Secretion Associated Ras Related GTPase 1A	2.21897
ZNF844	Zinc Finger Protein 844	2.21886
CAP1	Cyclase Associated Actin Cytoskeleton Regulatory Protein 1	2.21867
RPLP2	Ribosomal Protein Lateral Stalk Subunit P2	2.21841
TMEM59	Transmembrane Protein 59	2.21765
KBTBD4	Kelch Repeat And BTB Domain Containing 4	2.21660
CINP	Cyclin Dependent Kinase 2 Interacting Protein	2.21655
KDM7A	Lysine Demethylase 7A	2.21576
SSH3	Slingshot Protein Phosphatase 3	2.21510
RASA3	RAS P21 Protein Activator 3	2.21478
EIF4ENIF1	Eukaryotic Translation Initiation Factor 4E Nuclear Import Factor 1	2.21463
MYO5B	Myosin VB	2.21453
ZNF397	Zinc Finger Protein 397	2.21447
POLE4	DNA Polymerase Epsilon 4, Accessory Subunit	2.21444
LTK	Leukocyte Receptor Tyrosine Kinase	2.21436
GSTM4	Glutathione S-Transferase Mu 4	2.21379
EPGN	Epithelial Mitogen	2.21366
IFITM2	Interferon Induced Transmembrane Protein 2	2.21364
GIGYF2	GRB10 Interacting GYF Protein 2	2.21354
FBXW11	F-Box And WD Repeat Domain Containing 11	2.21336
CNOT6	CCR4-NOT Transcription Complex Subunit 6	2.21326
SELENOS	Selenoprotein S	2.21321
ELP1	Elongator Acetyltransferase Complex Subunit 1	2.21315

RBM22	RNA Binding Motif Protein 22	2.21309
PADI4	Peptidyl Arginine Deiminase 4	2.21306
HOXC5	Homeobox C5	2.21295
POMT2	Protein O-Mannosyltransferase 2	2.21215
HOOK1	Hook Microtubule Tethering Protein 1	2.21204
GIPC3	GIPC PDZ Domain Containing Family Member 3	2.21099
LEMD2	LEM Domain Nuclear Envelope Protein 2	2.21071
TAF2	TATA-Box Binding Protein Associated Factor 2	2.21000
ERLIN2	ER Lipid Raft Associated 2	2.20991
SUV39H2	Suppressor Of Variegation 3-9 Homolog 2	2.20983
TLK2	Tousled Like Kinase 2	2.20976
TARBP1	TAR (HIV-1) RNA Binding Protein 1	2.20973
STK38	Serine/Threonine Kinase 38	2.20930
TRIM39	Tripartite Motif Containing 39	2.20887
C3orf35	Chromosome 3 Open Reading Frame 35	2.20822
RGS9	Regulator Of G Protein Signaling 9	2.20789
ASPN	Asporin	2.20746
RHOV	Ras Homolog Family Member V	2.20662
CNN2	Calponin 2	2.20626
GIT1	GIT ArfGAP 1	2.20608
RNF180	Ring Finger Protein 180	2.20515
PACSIN3	Protein Kinase C And Casein Kinase Substrate In Neurons 3	2.20499
ENSG00000272953		2.20383
ZNF136	Zinc Finger Protein 136	2.20381
NT5C	5', 3'-Nucleotidase, Cytosolic	2.20307
TSR1	TSR1 Ribosome Maturation Factor	2.20234
LIMS2	LIM Zinc Finger Domain Containing 2	2.20217
UBTD2	Ubiquitin Domain Containing 2	2.20208
TAF6L	TATA-Box Binding Protein Associated Factor 6 Like	2.20146
NOC3L	NOC3 Like DNA Replication Regulator	2.20130
PTPRCAP	Protein Tyrosine Phosphatase Receptor Type C Associated Protein	2.20039
TMBIM1	Transmembrane BAX Inhibitor Motif Containing 1	2.19966
LOC10192971(	Uncharacterized LOC101929710	2.19937
TOP3B	DNA Topoisomerase III Beta	2.19919
NUP88	Nucleoporin 88	2.19888
CDK18	Cyclin Dependent Kinase 18	2.19850
CCDC85C	Coiled-Coil Domain Containing 85C	2.19812
CUL9	Cullin 9	2.19736
RBM25	RNA Binding Motif Protein 25	2.19727
DOP1B	DOP1 Leucine Zipper Like Protein B	2.19721

CSF2RB	Colony Stimulating Factor 2 Receptor Subunit Beta	2.19712
RAB33A	RAB33A, Member RAS Oncogene Family	2.19608
SPPL2B	Signal Peptide Peptidase Like 2B	2.19521
PLXNA1	Plexin A1	2.19497
ZNF222	Zinc Finger Protein 222	2.19410
CNTROB	Centrobin, Centriole Duplication And Spindle Assembly Protein	2.19399
GAGE12F	G Antigen 12F	2.19390
NACC2	NACC Family Member 2	2.19371
SH3GLB1	SH3 Domain Containing GRB2 Like, Endophilin B1	2.19341
ARIH1	Ariadne RBR E3 Ubiquitin Protein Ligase 1	2.19326
RPL13A	Ribosomal Protein L13a	2.19253
AMN	Amnion Associated Transmembrane Protein	2.19180
NFXL1	Nuclear Transcription Factor, X-Box Binding Like 1	2.19145
BRF2	BRF2 RNA Polymerase III Transcription Initiation Factor Subunit	2.19113
KRT78	Keratin 78	2.19083
SHANK3	SH3 And Multiple Ankyrin Repeat Domains 3	2.19060
MIR629	MicroRNA 629	2.19054
C1orf210	Chromosome 1 Open Reading Frame 210	2.19045
ZNF788P	Zinc Finger Family Member 788, Pseudogene	2.19045
RAB11FIP3	RAB11 Family Interacting Protein 3	2.19019
LFNG	LFNG O-Fucosylpeptide 3-Beta-N-Acetylglucosaminyltransferase	2.18978
EIF2B1	Eukaryotic Translation Initiation Factor 2B Subunit Alpha	2.18961
ADAMTS2	ADAM Metallopeptidase With Thrombospondin Type 1 Motif 2	2.18944
PSMC6	Proteasome 26S Subunit, ATPase 6	2.18896
HKDC1	Hexokinase Domain Containing 1	2.18850
MLC1	Modulator Of VRAC Current 1	2.18826
FBXO8	F-Box Protein 8	2.18804
KATNAL1	Katanin Catalytic Subunit A1 Like 1	2.18761
TCEANC2	Transcription Elongation Factor A N-Terminal And Central Domain Containing 2	2.18744
UNC119	Unc-119 Lipid Binding Chaperone	2.18741
CDH6	Cadherin 6	2.18738
MFSD4A	Major Facilitator Superfamily Domain Containing 4A	2.18713

OPRL1	Opioid Related Nociceptin Receptor 1	2.18536
PELATON	Plaque Enriched LncRNA In Atherosclerotic And Inflammatory Bowel Macrophage Regulation	2.18453
LOC11136514	NOS2 5' Regulatory Region	2.18410
SEPSECS	Sep (O-Phosphoserine) TRNA:Sec (Selenocysteine) TRNA Synthase	2.18367
TNRC6B	Trinucleotide Repeat Containing Adaptor 6B	2.18270
LOC10192955	Uncharacterized LOC101929552	2.18249
RN7SL381P	RNA, 7SL, Cytoplasmic 381, Pseudogene	2.18249
MINK1	Misshapen Like Kinase 1	2.18207
BCL2L10	BCL2 Like 10	2.17961
DNAJC17	DnaJ Heat Shock Protein Family (Hsp40) Member C17	2.17922
SRSF10	Serine And Arginine Rich Splicing Factor 10	2.17912
MT-TI	Mitochondrially Encoded TRNA-Ile (AUU/C)	2.17834
BCL7B	BAF Chromatin Remodeling Complex Subunit BCL7B	2.17773
TBX6	T-Box Transcription Factor 6	2.17723
ST3GAL5	ST3 Beta-Galactoside Alpha-2,3-Sialyltransferase 5	2.17697
RNF19A	Ring Finger Protein 19A, RBR E3 Ubiquitin Protein Ligase	2.17597
SLC30A4	Solute Carrier Family 30 Member 4	2.17554
ORM2	Orosomucoid 2	2.17508
WIPF2	WAS/WASL Interacting Protein Family Member 2	2.17500
ZNF264	Zinc Finger Protein 264	2.17423
ASIC3	Acid Sensing Ion Channel Subunit 3	2.17422
YTHDC2	YTH Domain Containing 2	2.17365
CCDC80	Coiled-Coil Domain Containing 80	2.17259
NNT	Nicotinamide Nucleotide Transhydrogenase	2.17189
KATNIP	Katanin Interacting Protein	2.17177
SOAT1	Sterol O-Acyltransferase 1	2.17176
SMIM20	Small Integral Membrane Protein 20	2.17092
PPA1	Inorganic Pyrophosphatase 1	2.17086
RASIP1	Ras Interacting Protein 1	2.16997
HTR6	5-Hydroxytryptamine Receptor 6	2.16995
STX12	Syntaxin 12	2.16917
VGLL4	Vestigial Like Family Member 4	2.16907
SGMS1	Sphingomyelin Synthase 1	2.16837
ZNF564	Zinc Finger Protein 564	2.16835
MIR612	MicroRNA 612	2.16781
CDC42EP1	CDC42 Effector Protein 1	2.16773

FOXO6	Forkhead Box O6	2.16769
TRPC1	Transient Receptor Potential Cation Channel Subfamily C Member 1	2.16730
MIR539	MicroRNA 539	2.16721
HPCAL1	Hippocalcin Like 1	2.16721
CHIA	Chitinase Acidic	2.16693
ZSCAN32	Zinc Finger And SCAN Domain Containing 32	2.16680
ATP2B4	ATPase Plasma Membrane Ca2+ Transporting 4	2.16671
SLC25A29	Solute Carrier Family 25 Member 29	2.16589
RORC	RAR Related Orphan Receptor C	2.16502
FAAP20	FA Core Complex Associated Protein 20	2.16477
SNRNP27	Small Nuclear Ribonucleoprotein U4/U6.U5 Subunit 27	2.16402
LYAR	Ly1 Antibody Reactive	2.16395
CNC2	Carney Complex Type 2, Multiple Neoplasia And Lentiginosis	2.16380
SPSB1	SplA/Ryanodine Receptor Domain And SOCS Box Containing 1	2.16374
ZMYM4	Zinc Finger MYM-Type Containing 4	2.16365
ZNF778	Zinc Finger Protein 778	2.16359
PTCHD1-AS	PTCHD1 Antisense RNA (Head To Head)	2.16333
ZNHIT2	Zinc Finger HIT-Type Containing 2	2.16328
ADAMTS9	ADAM Metallopeptidase With Thrombospondin Type 1 Motif 9	2.16301
B4GALNT1	Beta-1,4-N-Acetyl-Galactosaminyltransferase 1	2.16292
VIPR2	Vasoactive Intestinal Peptide Receptor 2	2.16243
NCCRP1	NCCRP1, F-Box Associated Domain Containing	2.16221
BTG4	BTG Anti-Proliferation Factor 4	2.16181
ING5	Inhibitor Of Growth Family Member 5	2.16123
PBX4	PBX Homeobox 4	2.16024
POU2F2	POU Class 2 Homeobox 2	2.16001
DYNLRB1	Dynein Light Chain Roadblock-Type 1	2.15978
SVBP	Small Vasohibin Binding Protein	2.15975
SKA1	Spindle And Kinetochore Associated Complex Subunit 1	2.15923
PSMB3	Proteasome 20S Subunit Beta 3	2.15823
SNURF	SNRPN Upstream Open Reading Frame	2.15799
PTRH1	Peptidyl-TRNA Hydrolase 1 Homolog	2.15654
MAST1	Microtubule Associated Serine/Threonine Kinase 1	2.15615
FPR1	Formyl Peptide Receptor 1	2.15578
SHC3	SHC Adaptor Protein 3	2.15562
TADA2B	Transcriptional Adaptor 2B	2.15531

NFX1	Nuclear Transcription Factor, X-Box Binding 1	2.15474
GPAT3	Glycerol-3-Phosphate Acyltransferase 3	2.15456
PTGR1	Prostaglandin Reductase 1	2.15439
ERAL1	Era Like 12S Mitochondrial RRNA Chaperone 1	2.15421
GNB1L	G Protein Subunit Beta 1 Like	2.15378
ANKRD65	Ankyrin Repeat Domain 65	2.15348
ZNF395	Zinc Finger Protein 395	2.15307
OCEL1	Occludin/ELL Domain Containing 1	2.15231
IFT46	Intraflagellar Transport 46	2.15203
C19orf12	Chromosome 19 Open Reading Frame 12	2.15037
TADA3	Transcriptional Adaptor 3	2.14957
GSTM5	Glutathione S-Transferase Mu 5	2.14946
NCSTN	Nicastrin	2.14937
ADAMTS7P3	ADAMTS7 Pseudogene 3	2.14936
AGFG1	ArfGAP With FG Repeats 1	2.14933
RPL32	Ribosomal Protein L32	2.14879
ZNF540	Zinc Finger Protein 540	2.14860
EMP3	Epithelial Membrane Protein 3	2.14850
KCTD2	Potassium Channel Tetramerization Domain Containing 2	2.14825
ASAH2	N-Acylsphingosine Amidohydrolase 2	2.14812
MEPE	Matrix Extracellular Phosphoglycoprotein	2.14804
MIR647	MicroRNA 647	2.14630
APBB1	Amyloid Beta Precursor Protein Binding Family B Member 1	2.14628
CSAD	Cysteine Sulfinic Acid Decarboxylase	2.14579
GRPEL1	GrpE Like 1, Mitochondrial	2.14544
LOC642361	Uncharacterized LOC642361	2.14514
ENSG00000263680		2.14514
lnc-SOX9-7		2.14514
RF00017-1894		2.14514
SUPT16H	SPT16 Homolog, Facilitates Chromatin Remodeling Subunit	2.14454
LOC10737231[illegible]	OSGEP/APEX1 Bi-Directional Promoter Region	2.14433
HSD17B8	Hydroxysteroid 17-Beta Dehydrogenase 8	2.14396
NAGA	Alpha-N-Acetylgalactosaminidase	2.14387
ITGA7	Integrin Subunit Alpha 7	2.14283
CD2BP2	CD2 Cytoplasmic Tail Binding Protein 2	2.14230
H2AC6	H2A Clustered Histone 6	2.14216
UBE4A	Ubiquitination Factor E4A	2.14184
NGRN	Neugrin, Neurite Outgrowth Associated	2.14180
IL31	Interleukin 31	2.14153
MIR505	MicroRNA 505	2.14145

EFNB3	Ephrin B3	2.13923
GSDMD	Gasdermin D	2.13838
UBE2M	Ubiquitin Conjugating Enzyme E2 M	2.13774
CLASP1	Cytoplasmic Linker Associated Protein 1	2.13738
IGIP	IgA Inducing Protein	2.13707
PRSS36	Serine Protease 36	2.13622
ZNF574	Zinc Finger Protein 574	2.13602
ACACB	Acetyl-CoA Carboxylase Beta	2.13597
NPIPB2	Nuclear Pore Complex Interacting Protein Family Member B2	2.13591
PTTG1IP	PTTG1 Interacting Protein	2.13583
FABP12	Fatty Acid Binding Protein 12	2.13525
RASSF4	Ras Association Domain Family Member 4	2.13462
INPP5J	Inositol Polyphosphate-5-Phosphatase J	2.13447
MPHOSPH9	M-Phase Phosphoprotein 9	2.13411
GTF2H2C	GTF2H2 Family Member C	2.13411
POLA2	DNA Polymerase Alpha 2, Accessory Subunit	2.13326
H4C8	H4 Clustered Histone 8	2.13316
ST13	ST13 Hsp70 Interacting Protein	2.13299
DEFA1B	Defensin Alpha 1B	2.13274
TNS2	Tensin 2	2.13267
MIR4316	MicroRNA 4316	2.13249
OTX1	Orthodenticle Homeobox 1	2.13244
GRIK2	Glutamate Ionotropic Receptor Kainate Type Subunit 2	2.13223
PRKAG1	Protein Kinase AMP-Activated Non-Catalytic Subunit Gamma 1	2.13203
LRG1	Leucine Rich Alpha-2-Glycoprotein 1	2.13198
ANKRD40	Ankyrin Repeat Domain 40	2.13135
ZNRF1	Zinc And Ring Finger 1	2.13097
RGS2	Regulator Of G Protein Signaling 2	2.13078
SUPT4H1	SPT4 Homolog, DSIF Elongation Factor Subunit	2.13060
SF3B3	Splicing Factor 3b Subunit 3	2.13051
TBC1D9B	TBC1 Domain Family Member 9B	2.13035
MARVELD2	MARVEL Domain Containing 2	2.13006
PAM16	Presequence Translocase Associated Motor 16	2.12980
CCNL2	Cyclin L2	2.12976
MGAT1	Alpha-1,3-Mannosyl-Glycoprotein 2-Beta-N-Acetylglucosaminyltransferase	2.12887
CDHR2	Cadherin Related Family Member 2	2.12886
SLC23A2	Solute Carrier Family 23 Member 2	2.12874
FN3KRP	Fructosamine 3 Kinase Related Protein	2.12849
CPEB4	Cytoplasmic Polyadenylation Element Binding Protein 4	2.12840

MIR630	MicroRNA 630	2.12743
BRIX1	Biogenesis Of Ribosomes BRX1	2.12716
PCNX3	Pecanex 3	2.12709
ADCY6	Adenylate Cyclase 6	2.12706
DNASE2	Deoxyribonuclease 2, Lysosomal	2.12683
UBL3	Ubiquitin Like 3	2.12674
LY96	Lymphocyte Antigen 96	2.12613
DOK7	Docking Protein 7	2.12600
AHCYL1	Adenosylhomocysteinase Like 1	2.12587
GABARAP	GABA Type A Receptor-Associated Protein	2.12573
RASSF10	Ras Association Domain Family Member 10	2.12520
NAV1	Neuron Navigator 1	2.12407
ANGPT4	Angiopoietin 4	2.12360
ANP32A	Acidic Nuclear Phosphoprotein 32 Family Member A	2.12340
WDR83	WD Repeat Domain 83	2.12310
H4C3	H4 Clustered Histone 3	2.12287
SLC38A10	Solute Carrier Family 38 Member 10	2.12260
NEUROD2	Neuronal Differentiation 2	2.12242
HNRNPDL	Heterogeneous Nuclear Ribonucleoprotein D Like	2.12238
PDK4	Pyruvate Dehydrogenase Kinase 4	2.12230
GTF2A1	General Transcription Factor IIA Subunit 1	2.12198
LGALS8	Galectin 8	2.12165
PPM1E	Protein Phosphatase, Mg2+/Mn2+ Dependent 1E	2.12153
ZNRD2	Zinc Ribbon Domain Containing 2	2.12128
PKDREJ	Polycystin Family Receptor For Egg Jelly	2.12076
CD300E	CD300e Molecule	2.12054
ETNK2	Ethanolamine Kinase 2	2.12039
LOC10013069	Uncharacterized LOC100130691	2.11985
VASH2	Vasohibin 2	2.11967
ENSG00000226281		2.11947
ALG9	ALG9 Alpha-1,2-Mannosyltransferase	2.11921
CARD6	Caspase Recruitment Domain Family Member 6	2.11905
CRADD	CASP2 And RIPK1 Domain Containing Adaptor With Death Domain	2.11811
CREBL2	CAMP Responsive Element Binding Protein Like 2	2.11799
KCNQ1-AS1	KCNQ1 Antisense RNA 1	2.11758
GLUD1	Glutamate Dehydrogenase 1	2.11725
MXRA8	Matrix Remodeling Associated 8	2.11599
WWP1	WW Domain Containing E3 Ubiquitin Protein Ligase 1	2.11545

TBC1D14	TBC1 Domain Family Member 14	2.11424
ZNF284	Zinc Finger Protein 284	2.11406
MPIG6B	Megakaryocyte And Platelet Inhibitory Receptor G6b	2.11390
PDCD11	Programmed Cell Death 11	2.11317
AGTRAP	Angiotensin II Receptor Associated Protein	2.11213
DUSP9	Dual Specificity Phosphatase 9	2.11202
S100A3	S100 Calcium Binding Protein A3	2.11163
NEU3	Neuraminidase 3	2.11070
EDRF1	Erythroid Differentiation Regulatory Factor 1	2.11017
TP53AIP1	Tumor Protein P53 Regulated Apoptosis Inducing Protein 1	2.10914
FKBP4	FKBP Prolyl Isomerase 4	2.10908
UNKL	Unk Like Zinc Finger	2.10811
SPART	Spartin	2.10782
ENSG00000259275		2.10697
ENSG00000255872		2.10697
RF00017-937		2.10697
ZNF146	Zinc Finger Protein 146	2.10646
SMURF2	SMAD Specific E3 Ubiquitin Protein Ligase 2	2.10642
SUPT6H	SPT6 Homolog, Histone Chaperone And Transcription Elongation Factor	2.10637
TSPAN14	Tetraspanin 14	2.10615
CTSW	Cathepsin W	2.10605
CSNK1G2	Casein Kinase 1 Gamma 2	2.10604
ABRAXAS2	Abraxas 2, BRISC Complex Subunit	2.10534
WDR83OS	WD Repeat Domain 83 Opposite Strand	2.10531
SLC45A4	Solute Carrier Family 45 Member 4	2.10522
MIR153-1	MicroRNA 153-1	2.10519
MIP	Major Intrinsic Protein Of Lens Fiber	2.10493
IL12RB2	Interleukin 12 Receptor Subunit Beta 2	2.10490
ZNF691	Zinc Finger Protein 691	2.10490
EGFL8	EGF Like Domain Multiple 8	2.10489
MORN1	MORN Repeat Containing 1	2.10488
HARS2	Histidyl-TRNA Synthetase 2, Mitochondrial	2.10436
ATXN1	Ataxin 1	2.10423
MASP2	MBL Associated Serine Protease 2	2.10413
MTRFR	Mitochondrial Translation Release Factor In Rescue	2.10350
SUGP1	SURP And G-Patch Domain Containing 1	2.10277
BRPF3	Bromodomain And PHD Finger Containing 3	2.10084
SLC2A9	Solute Carrier Family 2 Member 9	2.10081

SHANK1	SH3 And Multiple Ankyrin Repeat Domains 1	2.10071
EVI5	Ecotropic Viral Integration Site 5	2.09996
PARP6	Poly(ADP-Ribose) Polymerase Family Member 6	2.09995
HECA	Hdc Homolog, Cell Cycle Regulator	2.09929
CUTC	CutC Copper Transporter	2.09923
MEGF8	Multiple EGF Like Domains 8	2.09908
B3GNT3	UDP-GlcNAc:BetaGal Beta-1,3-N-Acetylglucosaminyltransferase 3	2.09896
TMEM258	Transmembrane Protein 258	2.09891
ASPG	Asparaginase	2.09888
DDX47	DEAD-Box Helicase 47	2.09884
WDR81	WD Repeat Domain 81	2.09843
KCNAB2	Potassium Voltage-Gated Channel Subfamily A Regulatory Beta Subunit 2	2.09842
FN3K	Fructosamine 3 Kinase	2.09757
PCCA	Propionyl-CoA Carboxylase Subunit Alpha	2.09719
SLC2A6	Solute Carrier Family 2 Member 6	2.09695
PHF2	PHD Finger Protein 2	2.09683
OSGEP	O-Sialoglycoprotein Endopeptidase	2.09673
FCGRT	Fc Fragment Of IgG Receptor And Transporter	2.09608
TMEM115	Transmembrane Protein 115	2.09587
UBE2F	Ubiquitin Conjugating Enzyme E2 F (Putative)	2.09431
SMC5	Structural Maintenance Of Chromosomes 5	2.09394
DNAH1	Dynein Axonemal Heavy Chain 1	2.09381
HMGB3	High Mobility Group Box 3	2.09373
ATP2B1	ATPase Plasma Membrane Ca2+ Transporting 1	2.09348
C1QBP	Complement C1q Binding Protein	2.09348
NPTX2	Neuronal Pentraxin 2	2.09325
ATXN7L1	Ataxin 7 Like 1	2.09299
AP5Z1	Adaptor Related Protein Complex 5 Subunit Zeta 1	2.09264
HOXB5	Homeobox B5	2.09232
ZNF471	Zinc Finger Protein 471	2.09211
SPARCL1	SPARC Like 1	2.09200
CYP7A1	Cytochrome P450 Family 7 Subfamily A Member 1	2.09132
DDX20	DEAD-Box Helicase 20	2.09099
CPSF4	Cleavage And Polyadenylation Specific Factor 4	2.09036
ZW10	Zw10 Kinetochore Protein	2.09001
LUC7L	LUC7 Like	2.08984
PNO1	Partner Of NOB1 Homolog	2.08929

LCN1	Lipocalin 1	2.08926
GRM5	Glutamate Metabotropic Receptor 5	2.08916
CDK15	Cyclin Dependent Kinase 15	2.08778
NDUFAF3	NADH:Ubiquinone Oxidoreductase Complex Assembly Factor 3	2.08705
ARG2	Arginase 2	2.08679
RGCC	Regulator Of Cell Cycle	2.08674
COL12A1	Collagen Type XII Alpha 1 Chain	2.08642
HP1BP3	Heterochromatin Protein 1 Binding Protein 3	2.08466
IER2	Immediate Early Response 2	2.08456
COL16A1	Collagen Type XVI Alpha 1 Chain	2.08411
MTNR1B	Melatonin Receptor 1B	2.08373
CDIPT	CDP-Diacylglycerol--Inositol 3-Phosphatidyltransferase	2.08345
SLC2A8	Solute Carrier Family 2 Member 8	2.08302
RER1	Retention In Endoplasmic Reticulum Sorting Receptor 1	2.08300
TAOK2	TAO Kinase 2	2.08292
ZC3H7B	Zinc Finger CCCH-Type Containing 7B	2.08249
GDPD5	Glycerophosphodiester Phosphodiesterase Domain Containing 5	2.08249
LINC00908	Long Intergenic Non-Protein Coding RNA 908	2.08249
APEH	Acylaminoacyl-Peptide Hydrolase	2.08241
IFI6	Interferon Alpha Inducible Protein 6	2.08215
PHKA2	Phosphorylase Kinase Regulatory Subunit Alpha 2	2.08210
MIR484	MicroRNA 484	2.08171
ALDH1A2	Aldehyde Dehydrogenase 1 Family Member A2	2.08111
TRIM65	Tripartite Motif Containing 65	2.08042
SLCO1A2	Solute Carrier Organic Anion Transporter Family Member 1A2	2.07936
SH2D4B	SH2 Domain Containing 4B	2.07924
HNRNPH3	Heterogeneous Nuclear Ribonucleoprotein H3	2.07838
MID2	Midline 2	2.07832
STAG3L1	Stromal Antigen 3-Like 1 (Pseudogene)	2.07831
SHOX2	Short Stature Homeobox 2	2.07809
C11orf80	Chromosome 11 Open Reading Frame 80	2.07774
SLC30A2	Solute Carrier Family 30 Member 2	2.07679
SAP18	Sin3A Associated Protein 18	2.07662
ZP3	Zona Pellucida Glycoprotein 3	2.07658
IDE	Insulin Degrading Enzyme	2.07644
NEUROG1	Neurogenin 1	2.07634
QTRT1	Queuine TRNA-Ribosyltransferase Catalytic Subunit 1	2.07586

KMT5A	Lysine Methyltransferase 5A	2.07552
NEMP1	Nuclear Envelope Integral Membrane Protein 1	2.07552
ROBO2	Roundabout Guidance Receptor 2	2.07528
ARHGAP27	Rho GTPase Activating Protein 27	2.07467
RIMS2	Regulating Synaptic Membrane Exocytosis 2	2.07434
RNF144B	Ring Finger Protein 144B	2.07411
AUP1	AUP1 Lipid Droplet Regulating VLDL Assembly Factor	2.07406
GPN2	GPN-Loop GTPase 2	2.07305
MLLT6	MLLT6, PHD Finger Containing	2.07260
ZNF79	Zinc Finger Protein 79	2.07193
EMX2	Empty Spiracles Homeobox 2	2.07191
PURB	Purine Rich Element Binding Protein B	2.07180
CDC42BPA	CDC42 Binding Protein Kinase Alpha	2.07108
RF00017-7410		2.07077
FBXO42	F-Box Protein 42	2.07063
RNF2	Ring Finger Protein 2	2.07036
SV2A	Synaptic Vesicle Glycoprotein 2A	2.07011
TECPR1	Tectonin Beta-Propeller Repeat Containing 1	2.07002
USP33	Ubiquitin Specific Peptidase 33	2.06948
ERVFRD-1	Endogenous Retrovirus Group FRD Member 1, Envelope	2.06937
ZNF687	Zinc Finger Protein 687	2.06885
DDX46	DEAD-Box Helicase 46	2.06859
PLXDC1	Plexin Domain Containing 1	2.06858
MSL1	MSL Complex Subunit 1	2.06852
EEA1	Early Endosome Antigen 1	2.06849
TANGO6	Transport And Golgi Organization 6 Homolog	2.06819
SLC5A7	Solute Carrier Family 5 Member 7	2.06798
ENSG00000251409		2.06792
LOC10192726:	Uncharacterized LOC101927263	2.06792
HSALNG0108407		2.06792
NLRP2	NLR Family Pyrin Domain Containing 2	2.06754
TXNL4B	Thioredoxin Like 4B	2.06750
ZCCHC10	Zinc Finger CCHC-Type Containing 10	2.06697
SNRNP40	Small Nuclear Ribonucleoprotein U5 Subunit 40	2.06695
NR2F6	Nuclear Receptor Subfamily 2 Group F Member 6	2.06684
ANKRD13D	Ankyrin Repeat Domain 13D	2.06652
HCG22	HLA Complex Group 22 (Gene/Pseudogene)	2.06647
PPM1G	Protein Phosphatase, Mg2+/Mn2+ Dependent 1G	2.06563

MIR520B	MicroRNA 520b	2.06556
CKAP5	Cytoskeleton Associated Protein 5	2.06547
BCAP31	B Cell Receptor Associated Protein 31	2.06523
DHDDS	Dehydrodolichyl Diphosphate Synthase Subunit	2.06483
TMEM125	Transmembrane Protein 125	2.06465
TRAPPC1	Trafficking Protein Particle Complex Subunit 1	2.06437
ICAM3	Intercellular Adhesion Molecule 3	2.06352
NCDN	Neurochondrin	2.06351
CAPS	Calcyphosine	2.06316
SYBU	Syntabulin	2.06316
SYT11	Synaptotagmin 11	2.06288
NRBP1	Nuclear Receptor Binding Protein 1	2.06117
TAS1R3	Taste 1 Receptor Member 3	2.06095
ZBED6	Zinc Finger BED-Type Containing 6	2.06081
MAN2A1	Mannosidase Alpha Class 2A Member 1	2.06011
SP2	Sp2 Transcription Factor	2.05941
RNF40	Ring Finger Protein 40	2.05916
DNM3	Dynamin 3	2.05884
OGA	O-GlcNAcase	2.05883
FAM20C	FAM20C Golgi Associated Secretory Pathway Kinase	2.05870
SLITRK3	SLIT And NTRK Like Family Member 3	2.05837
CSRNP1	Cysteine And Serine Rich Nuclear Protein 1	2.05804
FAM126B	Family With Sequence Similarity 126 Member B	2.05785
MARVELD3	MARVEL Domain Containing 3	2.05775
CTU2	Cytosolic Thiouridylase Subunit 2	2.05751
DIMT1	DIMT1 RRNA Methyltransferase And Ribosome Maturation Factor	2.05739
HCP5	HLA Complex P5	2.05731
FAM117A	Family With Sequence Similarity 117 Member A	2.05702
VPS13D	Vacuolar Protein Sorting 13 Homolog D	2.05690
CHAMP1	Chromosome Alignment Maintaining Phosphoprotein 1	2.05648
DLX2	Distal-Less Homeobox 2	2.05616
NELFB	Negative Elongation Factor Complex Member B	2.05603
TIGD6	Tigger Transposable Element Derived 6	2.05578
NUP210	Nucleoporin 210	2.05537
POMGNT1	Protein O-Linked Mannose N-Acetylglucosaminyltransferase 1 (Beta 1,2-)	2.05512
UCHL5	Ubiquitin C-Terminal Hydrolase L5	2.05491

HAPLN3	Hyaluronan And Proteoglycan Link Protein 3	2.05467
HRH3	Histamine Receptor H3	2.05357
MICALL2	MICAL Like 2	2.05314
GPATCH2L	G-Patch Domain Containing 2 Like	2.05308
ESD	Esterase D	2.05296
CCL28	C-C Motif Chemokine Ligand 28	2.05242
SLC23A1	Solute Carrier Family 23 Member 1	2.05170
PRR12	Proline Rich 12	2.05163
UTP18	UTP18 Small Subunit Processome Component	2.05109
ANKRD28	Ankyrin Repeat Domain 28	2.05097
RNF20	Ring Finger Protein 20	2.05054
NYX	Nyctalopin	2.04984
LENG8	Leukocyte Receptor Cluster Member 8	2.04858
IFT27	Intraflagellar Transport 27	2.04831
GTSE1	G2 And S-Phase Expressed 1	2.04796
LPIN1	Lipin 1	2.04776
TMEM63B	Transmembrane Protein 63B	2.04760
DHRS3	Dehydrogenase/Reductase 3	2.04744
TMCC1	Transmembrane And Coiled-Coil Domain Family 1	2.04709
SGPL1	Sphingosine-1-Phosphate Lyase 1	2.04689
CDH15	Cadherin 15	2.04566
GPRC5C	G Protein-Coupled Receptor Class C Group 5 Member C	2.04555
BLOC1S1	Biogenesis Of Lysosomal Organelles Complex 1 Subunit 1	2.04499
ZNF875	Zinc Finger Protein 875	2.04390
HAP1	Huntingtin Associated Protein 1	2.04338
DEFB121	Defensin Beta 121	2.04296
PPP1R18	Protein Phosphatase 1 Regulatory Subunit 18	2.04210
ATXN2L	Ataxin 2 Like	2.04188
TMEM222	Transmembrane Protein 222	2.04187
TRIM44	Tripartite Motif Containing 44	2.04175
MRPS12	Mitochondrial Ribosomal Protein S12	2.04133
APOL3	Apolipoprotein L3	2.04069
PDXDC1	Pyridoxal Dependent Decarboxylase Domain Containing 1	2.04054
CNOT1	CCR4-NOT Transcription Complex Subunit 1	2.04052
MMP21	Matrix Metallopeptidase 21	2.04007
DIXDC1	DIX Domain Containing 1	2.04001
TTF2	Transcription Termination Factor 2	2.03984
GSDMC	Gasdermin C	2.03955
CXCL17	C-X-C Motif Chemokine Ligand 17	2.03873
PSMB10	Proteasome 20S Subunit Beta 10	2.03827
SBF2	SET Binding Factor 2	2.03808

NELL1	Neural EGFL Like 1	2.03789
ZBTB8A	Zinc Finger And BTB Domain Containing 8A	2.03747
CHRM1	Cholinergic Receptor Muscarinic 1	2.03721
CCL24	C-C Motif Chemokine Ligand 24	2.03650
CEACAM8	CEA Cell Adhesion Molecule 8	2.03605
RSU1	Ras Suppressor Protein 1	2.03569
PRMT6	Protein Arginine Methyltransferase 6	2.03538
CPD	Carboxypeptidase D	2.03503
KCNK9	Potassium Two Pore Domain Channel Subfamily K Member 9	2.03486
PPIL2	Peptidylprolyl Isomerase Like 2	2.03352
NICN1	Nicolin 1	2.03333
FLII	FLII Actin Remodeling Protein	2.03306
TSPAN15	Tetraspanin 15	2.03269
ROBO4	Roundabout Guidance Receptor 4	2.03253
AHRR	Aryl-Hydrocarbon Receptor Repressor	2.03252
GPSM3	G Protein Signaling Modulator 3	2.03226
SUMF1	Sulfatase Modifying Factor 1	2.03172
HLA-DOA	Major Histocompatibility Complex, Class II, DO Alpha	2.03091
SLX1B	SLX1 Homolog B, Structure-Specific Endonuclease Subunit	2.03056
NEURL4	Neuralized E3 Ubiquitin Protein Ligase 4	2.03055
LINC02381	Long Intergenic Non-Protein Coding RNA 2381	2.02973
GNG2	G Protein Subunit Gamma 2	2.02964
PLAAT4	Phospholipase A And Acyltransferase 4	2.02917
BAAT	Bile Acid-CoA:Amino Acid N-Acyltransferase	2.02910
GPRC6A	G Protein-Coupled Receptor Class C Group 6 Member A	2.02891
DENND1A	DENN Domain Containing 1A	2.02869
LXN	Latexin	2.02859
CDC42EP3	CDC42 Effector Protein 3	2.02812
CNTF	Ciliary Neurotrophic Factor	2.02805
SGSM3-AS1	SGSM3 Antisense RNA 1	2.02793
lnc-ABR-6-001		2.02793
ZBTB4	Zinc Finger And BTB Domain Containing 4	2.02717
USE1	Unconventional SNARE In The ER 1	2.02712
CCDC32	Coiled-Coil Domain Containing 32	2.02700
CNP	2',3'-Cyclic Nucleotide 3' Phosphodiesterase	2.02682
ADAM1A	ADAM Metallopeptidase Domain 1A (Pseudogene)	2.02620
RPL3P2	Ribosomal Protein L3 Pseudogene 2	2.02620
L13712-014		2.02620

RF00017-1156		2.02620
RF00017-2611		2.02620
LINC01550	Long Intergenic Non-Protein Coding RNA 1550	2.02554
SRP9P1	Signal Recognition Particle 9 Pseudogene 1	2.02554
LINC01848	Long Intergenic Non-Protein Coding RNA 1848	2.02554
RPL12P1	Ribosomal Protein L12 Pseudogene 1	2.02554
POM121L13P	POM121 Transmembrane Nucleoporin Like 13, Pseudogene	2.02554
MTCO3P10	MT-CO3 Pseudogene 10	2.02554
ANAPC4	Anaphase Promoting Complex Subunit 4	2.02461
FLG-AS1	FLG Antisense RNA 1	2.02388
INO80D	INO80 Complex Subunit D	2.02376
CDNF	Cerebral Dopamine Neurotrophic Factor	2.02359
ACAD10	Acyl-CoA Dehydrogenase Family Member 10	2.02308
WWOX-AS1	WWOX Antisense RNA 1	2.02283
UNK	Unk Zinc Finger	2.02280
FOXD3	Forkhead Box D3	2.02231
SLC2A12	Solute Carrier Family 2 Member 12	2.02228
CERS1	Ceramide Synthase 1	2.02167
TRIM46	Tripartite Motif Containing 46	2.02148
PSMD5	Proteasome 26S Subunit, Non-ATPase 5	2.02094
DPH2	Diphthamide Biosynthesis 2	2.02077
DNPH1	2'-Deoxynucleoside 5'-Phosphate N-Hydrolase 1	2.02060
DYNLT1	Dynein Light Chain Tctex-Type 1	2.02053
ZNF394	Zinc Finger Protein 394	2.02046
POFUT2	Protein O-Fucosyltransferase 2	2.02020
DDX55	DEAD-Box Helicase 55	2.01976
PARP4	Poly(ADP-Ribose) Polymerase Family Member 4	2.01911
DRAM2	DNA Damage Regulated Autophagy Modulator 2	2.01867
DHRS2	Dehydrogenase/Reductase 2	2.01860
EIF2B4	Eukaryotic Translation Initiation Factor 2B Subunit Delta	2.01825
SPAG5	Sperm Associated Antigen 5	2.01759
BAG4	BAG Cochaperone 4	2.01728
FAM193A	Family With Sequence Similarity 193 Member A	2.01723
ALOX5AP	Arachidonate 5-Lipoxygenase Activating Protein	2.01710
PTGDR	Prostaglandin D2 Receptor	2.01690
NHLRC1	NHL Repeat Containing E3 Ubiquitin Protein Ligase 1	2.01666

APOBEC3A	Apolipoprotein B MRNA Editing Enzyme Catalytic Subunit 3A	2.01652
CHTOP	Chromatin Target Of PRMT1	2.01649
HES6	Hes Family BHLH Transcription Factor 6	2.01624
PLEKHH3	Pleckstrin Homology, MyTH4 And FERM Domain Containing H3	2.01573
HYI	Hydroxypyruvate Isomerase (Putative)	2.01499
ZNF20	Zinc Finger Protein 20	2.01437
PMS2P3	PMS1 Homolog 2, Mismatch Repair System Component Pseudogene 3	2.01422
HHEX	Hematopoietically Expressed Homeobox	2.01399
MAP3K12	Mitogen-Activated Protein Kinase Kinase Kinase 12	2.01371
SLC27A3	Solute Carrier Family 27 Member 3	2.01360
PHC2	Polyhomeotic Homolog 2	2.01360
AGFG2	ArfGAP With FG Repeats 2	2.01315
POLR2B	RNA Polymerase II Subunit B	2.01279
MED8	Mediator Complex Subunit 8	2.01249
PDE6C	Phosphodiesterase 6C	2.01248
KRT84	Keratin 84	2.01241
LBP	Lipopolysaccharide Binding Protein	2.01233
DNAJC18	DnaJ Heat Shock Protein Family (Hsp40) Member C18	2.01201
C8orf17	Chromosome 8 Putative Open Reading Frame 17	2.01137
C2	Complement C2	2.01066
CRMP1	Collapsin Response Mediator Protein 1	2.00894
AGMAT	Agmatinase	2.00893
CANT1	Calcium Activated Nucleotidase 1	2.00830
CACNA2D4	Calcium Voltage-Gated Channel Auxiliary Subunit Alpha2delta 4	2.00702
PXDC1	PX Domain Containing 1	2.00699
ZNF490	Zinc Finger Protein 490	2.00693
SOCS4	Suppressor Of Cytokine Signaling 4	2.00652
CCNK	Cyclin K	2.00593
SPNS3	Sphingolipid Transporter 3 (Putative)	2.00589
MT-TW	Mitochondrially Encoded TRNA-Trp (UGA/G)	2.00573
LRRC74A	Leucine Rich Repeat Containing 74A	2.00488
TRPM5	Transient Receptor Potential Cation Channel Subfamily M Member 5	2.00452
EDA2R	Ectodysplasin A2 Receptor	2.00408
LBX1	Ladybird Homeobox 1	2.00356
EIF4EBP3	Eukaryotic Translation Initiation Factor 4E Binding Protein 3	2.00346
RNF130	Ring Finger Protein 130	2.00298
PI4KB	Phosphatidylinositol 4-Kinase Beta	2.00268

KNTC1	Kinetochore Associated 1	2.00252
FLVCR2	FLVCR Heme Transporter 2	2.00237
ERO1A	Endoplasmic Reticulum Oxidoreductase 1 Alpha	2.00194
KATNB1	Katanin Regulatory Subunit B1	2.00176
PPT1	Palmitoyl-Protein Thioesterase 1	2.00110
NSUN5P1	NSUN5 Pseudogene 1	2.00106
DYNC1I2	Dynein Cytoplasmic 1 Intermediate Chain 2	2.00075
LZIC	Leucine Zipper And CTNNBIP1 Domain Containing	2.00001
RPAP3	RNA Polymerase II Associated Protein 3	1.99900
LIPH	Lipase H	1.99823
FOXD1	Forkhead Box D1	1.99810
LTB	Lymphotoxin Beta	1.99782
FMO3	Flavin Containing Dimethylaniline Monoxygenase 3	1.99755
AKAP8L	A-Kinase Anchoring Protein 8 Like	1.99649
DEFA3	Defensin Alpha 3	1.99638
ZNF221	Zinc Finger Protein 221	1.99573
CAPN7	Calpain 7	1.99461
FBXL6	F-Box And Leucine Rich Repeat Protein 6	1.99423
CHKB	Choline Kinase Beta	1.99363
TBC1D22A	TBC1 Domain Family Member 22A	1.99335
ARHGAP35	Rho GTPase Activating Protein 35	1.99294
IER3IP1	Immediate Early Response 3 Interacting Protein 1	1.99294
TESMIN	Testis Expressed Metallothionein Like Protein	1.99293
ACOT11	Acyl-CoA Thioesterase 11	1.99203
CPNE7	Copine 7	1.99188
SPSB2	SplA/Ryanodine Receptor Domain And SOCS Box Containing 2	1.99169
MIR135A2	MicroRNA 135a-2	1.99167
ZNF555	Zinc Finger Protein 555	1.99079
DNAJC9	DnaJ Heat Shock Protein Family (Hsp40) Member C9	1.99078
RBBP9	RB Binding Protein 9, Serine Hydrolase	1.99035
GPR37L1	G Protein-Coupled Receptor 37 Like 1	1.99025
STK39	Serine/Threonine Kinase 39	1.98995
BICRAL	BRD4 Interacting Chromatin Remodeling Complex Associated Protein Like	1.98983
PLAAT1	Phospholipase A And Acyltransferase 1	1.98970
SLC35E2B	Solute Carrier Family 35 Member E2B	1.98958
ZNF699	Zinc Finger Protein 699	1.98928
ZNF764	Zinc Finger Protein 764	1.98889
TMEM41A	Transmembrane Protein 41A	1.98884

GNL2	G Protein Nucleolar 2	1.98828
ZNF444	Zinc Finger Protein 444	1.98808
LINC01426	Long Intergenic Non-Protein Coding RNA 1426	1.98789
DR1	Down-Regulator Of Transcription 1	1.98738
TRABD	TraB Domain Containing	1.98693
ENSG00000270055		1.98693
ENSG00000265136		1.98693
ATP9A	ATPase Phospholipid Transporting 9A (Putative)	1.98664
POLR2M	RNA Polymerase II Subunit M	1.98615
SLC43A2	Solute Carrier Family 43 Member 2	1.98604
SNORA21	Small Nucleolar RNA, H/ACA Box 21	1.98544
ZNF664	Zinc Finger Protein 664	1.98474
IQCE	IQ Motif Containing E	1.98456
TUBE1	Tubulin Epsilon 1	1.98441
RNF10	Ring Finger Protein 10	1.98401
MBOAT7	Membrane Bound O-Acyltransferase Domain Containing 7	1.98394
TTC22	Tetratricopeptide Repeat Domain 22	1.98377
AGPS	Alkylglycerone Phosphate Synthase	1.98371
MOGS	Mannosyl-Oligosaccharide Glucosidase	1.98291
SH2D3C	SH2 Domain Containing 3C	1.98257
HAAO	3-Hydroxyanthranilate 3,4-Dioxygenase	1.98223
RAVER1	Ribonucleoprotein, PTB Binding 1	1.98185
IRF9	Interferon Regulatory Factor 9	1.98170
UBN1	Ubinuclein 1	1.98168
SRBD1	S1 RNA Binding Domain 1	1.98088
SEMA6B	Semaphorin 6B	1.98075
GDPGP1	GDP-D-Glucose Phosphorylase 1	1.98021
COL6A2	Collagen Type VI Alpha 2 Chain	1.98014
CASTOR3	CASTOR Family Member 3	1.97995
TDRD10	Tudor Domain Containing 10	1.97974
TOR1AIP1	Torsin 1A Interacting Protein 1	1.97775
ZFP91	ZFP91 Zinc Finger Protein, Atypical E3 Ubiquitin Ligase	1.97718
GLIS3	GLIS Family Zinc Finger 3	1.97667
RASSF6	Ras Association Domain Family Member 6	1.97638
HMBOX1	Homeobox Containing 1	1.97622
CENATAC	Centrosomal AT-AC Splicing Factor	1.97593
GCKR	Glucokinase Regulator	1.97571
GRAMD1B	GRAM Domain Containing 1B	1.97561
PRKG2	Protein Kinase CGMP-Dependent 2	1.97539
TIMM17A	Translocase Of Inner Mitochondrial Membrane 17A	1.97518
APOC4	Apolipoprotein C4	1.97433
MAOB	Monoamine Oxidase B	1.97384

CCDC18	Coiled-Coil Domain Containing 18	1.97350
BMP3	Bone Morphogenetic Protein 3	1.97315
ZNF513	Zinc Finger Protein 513	1.97313
TMEM140	Transmembrane Protein 140	1.97265
AEBP1	AE Binding Protein 1	1.97260
MBD3	Methyl-CpG Binding Domain Protein 3	1.97256
TTLL10	Tubulin Tyrosine Ligase Like 10	1.97176
AMD1	Adenosylmethionine Decarboxylase 1	1.97160
MIR652	MicroRNA 652	1.97109
KIF3B	Kinesin Family Member 3B	1.97088
CYTH2	Cytohesin 2	1.97087
SETMAR	SET Domain And Mariner Transposase Fusion Gene	1.97054
LOC10783285[illegible]	SIRT1 Promoter Region	1.96983
CGNL1	Cingulin Like 1	1.96971
TTC23	Tetratricopeptide Repeat Domain 23	1.96958
SERPINA7	Serpin Family A Member 7	1.96926
CHST6	Carbohydrate Sulfotransferase 6	1.96875
CTNNAL1	Catenin Alpha Like 1	1.96811
PTPRA	Protein Tyrosine Phosphatase Receptor Type A	1.96808
KIAA0513	KIAA0513	1.96805
MIR1207	MicroRNA 1207	1.96762
SLC29A4	Solute Carrier Family 29 Member 4	1.96713
ATG2B	Autophagy Related 2B	1.96691
GPR4	G Protein-Coupled Receptor 4	1.96651
VPS37C	VPS37C Subunit Of ESCRT-I	1.96644
DCTPP1	DCTP Pyrophosphatase 1	1.96608
CHI3L2	Chitinase 3 Like 2	1.96399
SLC19A3	Solute Carrier Family 19 Member 3	1.96377
ULK3	Unc-51 Like Kinase 3	1.96373
VARS1	Valyl-TRNA Synthetase 1	1.96307
UBE2G2	Ubiquitin Conjugating Enzyme E2 G2	1.96259
ZNF625	Zinc Finger Protein 625	1.96207
WTIP	WT1 Interacting Protein	1.96147
KLC2	Kinesin Light Chain 2	1.96131
ADCY7	Adenylate Cyclase 7	1.96128
CRIPT	CXXC Repeat Containing Interactor Of PDZ3 Domain	1.96109
RNF213-AS1	RNF213 Antisense RNA 1	1.96109
ZFP82	ZFP82 Zinc Finger Protein	1.96072
UQCR10	Ubiquinol-Cytochrome C Reductase, Complex III Subunit X	1.95970
NLRP6	NLR Family Pyrin Domain Containing 6	1.95959
POLR1A	RNA Polymerase I Subunit A	1.95936
IFIT1	Interferon Induced Protein With Tetratricopeptide Repeats 1	1.95932
TSSK3	Testis Specific Serine Kinase 3	1.95913

MMRN2	Multimerin 2	1.95891
EIF1AD	Eukaryotic Translation Initiation Factor 1A Domain Containing	1.95849
USO1	USO1 Vesicle Transport Factor	1.95753
CCNF	Cyclin F	1.95746
GATD3B	Glutamine Amidotransferase Like Class 1 Domain Containing 3B	1.95712
FAM149B1	Family With Sequence Similarity 149 Member B1	1.95703
SLCO6A1	Solute Carrier Organic Anion Transporter Family Member 6A1	1.95678
CHD6	Chromodomain Helicase DNA Binding Protein 6	1.95524
FRA7G	Fragile Site, Aphidicolin Type, Common, Fra(7)(Q31.2)	1.95507
ACSF3	Acyl-CoA Synthetase Family Member 3	1.95500
CMKLR1	Chemerin Chemokine-Like Receptor 1	1.95413
RDH5	Retinol Dehydrogenase 5	1.95379
TALDO1	Transaldolase 1	1.95290
CACNA1D	Calcium Voltage-Gated Channel Subunit Alpha1 D	1.95258
SNRNP25	Small Nuclear Ribonucleoprotein U11/U12 Subunit 25	1.95241
MAF1	MAF1 Homolog, Negative Regulator Of RNA Polymerase III	1.95230
MYSM1	Myb Like, SWIRM And MPN Domains 1	1.95181
H2AJ	H2A.J Histone	1.95084
HEXIM1	HEXIM P-TEFb Complex Subunit 1	1.95080
COPS7A	COP9 Signalosome Subunit 7A	1.95035
TRIP6	Thyroid Hormone Receptor Interactor 6	1.95022
DGKI	Diacylglycerol Kinase Iota	1.95020
ROMO1	Reactive Oxygen Species Modulator 1	1.94910
NLRC5	NLR Family CARD Domain Containing 5	1.94901
SARNP	SAP Domain Containing Ribonucleoprotein	1.94875
ATOH8	Atonal BHLH Transcription Factor 8	1.94838
DENND3	DENN Domain Containing 3	1.94836
LARGE2	LARGE Xylosyl- And Glucuronyltransferase 2	1.94814
SEC22B	SEC22 Homolog B, Vesicle Trafficking Protein	1.94764
SSX2IP	SSX Family Member 2 Interacting Protein	1.94667
KPNA6	Karyopherin Subunit Alpha 6	1.94601
MOB1A	MOB Kinase Activator 1A	1.94534
GLMP	Glycosylated Lysosomal Membrane Protein	1.94504

VANGL2	VANGL Planar Cell Polarity Protein 2	1.94500
HLA-K	Major Histocompatibility Complex, Class I, K (Pseudogene)	1.94483
lnc-PGPEP1L-26		1.94483
ENSG00000274995		1.94483
VPS13C	Vacuolar Protein Sorting 13 Homolog C	1.94427
CELSR2	Cadherin EGF LAG Seven-Pass G-Type Receptor 2	1.94384
IMMT	Inner Membrane Mitochondrial Protein	1.94354
MIDEAS	Mitotic Deacetylase Associated SANT Domain Protein	1.94319
L1TD1	LINE1 Type Transposase Domain Containing 1	1.94302
SNX29	Sorting Nexin 29	1.94290
BORCS7	BLOC-1 Related Complex Subunit 7	1.94260
MIR940	MicroRNA 940	1.94163
CMTM3	CKLF Like MARVEL Transmembrane Domain Containing 3	1.94160
MBNL1-AS1	MBNL1 Antisense RNA 1	1.94111
SPRR1A	Small Proline Rich Protein 1A	1.94063
DUSP10	Dual Specificity Phosphatase 10	1.94028
TMC1	Transmembrane Channel Like 1	1.94027
CSGALNACT1	Chondroitin Sulfate N-Acetylgalactosaminyltransferase 1	1.94022
GCSH	Glycine Cleavage System Protein H	1.93940
RBM14	RNA Binding Motif Protein 14	1.93892
KMT2E-AS1	KMT2E Antisense RNA 1	1.93885
PLLP	Plasmolipin	1.93874
KDELR2	KDEL Endoplasmic Reticulum Protein Retention Receptor 2	1.93872
FDXR	Ferredoxin Reductase	1.93801
EXOC7	Exocyst Complex Component 7	1.93773
ABCA7	ATP Binding Cassette Subfamily A Member 7	1.93765
KANK1	KN Motif And Ankyrin Repeat Domains 1	1.93752
PHKG2	Phosphorylase Kinase Catalytic Subunit Gamma 2	1.93744
KANSL2	KAT8 Regulatory NSL Complex Subunit 2	1.93742
BSDC1	BSD Domain Containing 1	1.93734
SNX10	Sorting Nexin 10	1.93724
HRNR	Hornerin	1.93596
VPS8	VPS8 Subunit Of CORVET Complex	1.93585
PSORS1C1	Psoriasis Susceptibility 1 Candidate 1	1.93541
NT5M	5',3'-Nucleotidase, Mitochondrial	1.93442
MIR379	MicroRNA 379	1.93378
SEPTIN4	Septin 4	1.93368

NUCKS1	Nuclear Casein Kinase And Cyclin Dependent Kinase Substrate 1	1.93317
ENTPD5	Ectonucleoside Triphosphate Diphosphohydrolase 5 (Inactive)	1.93311
MOB2	MOB Kinase Activator 2	1.93307
LOC10050571:	Uncharacterized LOC100505715	1.93265
ENSG00000270679		1.93265
HSALNG0094037		1.93265
EPS8L3	EPS8 Like 3	1.93258
ZNF507	Zinc Finger Protein 507	1.93255
SRGN	Serglycin	1.93252
EXTL3	Exostosin Like Glycosyltransferase 3	1.93236
CABLES2	Cdk5 And Abl Enzyme Substrate 2	1.93224
BVES	Blood Vessel Epicardial Substance	1.93200
RYR2	Ryanodine Receptor 2	1.93183
PAQR3	Progestin And AdipoQ Receptor Family Member 3	1.93080
GPD1	Glycerol-3-Phosphate Dehydrogenase 1	1.93049
RPL30	Ribosomal Protein L30	1.93042
HSDL2	Hydroxysteroid Dehydrogenase Like 2	1.93036
RPL18A	Ribosomal Protein L18a	1.93033
LRRC23	Leucine Rich Repeat Containing 23	1.93029
DHX58	DExH-Box Helicase 58	1.93025
FBXO3	F-Box Protein 3	1.93016
STX4	Syntaxin 4	1.92975
PRXL2C	Peroxiredoxin Like 2C	1.92963
TAS2R38	Taste 2 Receptor Member 38	1.92960
AGPAT3	1-Acylglycerol-3-Phosphate O-Acyltransferase 3	1.92927
SHOC1	Shortage In Chiasmata 1	1.92927
CLCC1	Chloride Channel CLIC Like 1	1.92924
RPAP1	RNA Polymerase II Associated Protein 1	1.92902
WBP2	WW Domain Binding Protein 2	1.92882
CORO2B	Coronin 2B	1.92866
PCDHB15	Protocadherin Beta 15	1.92826
PFDN4	Prefoldin Subunit 4	1.92798
PIGL	Phosphatidylinositol Glycan Anchor Biosynthesis Class L	1.92780
IL36G	Interleukin 36 Gamma	1.92676
STRIP1	Striatin Interacting Protein 1	1.92673
ARF4	ADP Ribosylation Factor 4	1.92655
LINC00243	Long Intergenic Non-Protein Coding RNA 243	1.92634
CNMD	Chondromodulin	1.92624
ZNF346	Zinc Finger Protein 346	1.92589
ADRB1	Adrenoceptor Beta 1	1.92577
KRT86	Keratin 86	1.92577
MIR1290	MicroRNA 1290	1.92562

TRARG1	Trafficking Regulator Of GLUT4 (SLC2A4) 1	1.92474
COMMD5	COMM Domain Containing 5	1.92470
GPANK1	G-Patch Domain And Ankyrin Repeats 1	1.92460
CLTB	Clathrin Light Chain B	1.92412
SLC35E2A	Solute Carrier Family 35 Member E2A	1.92362
PI16	Peptidase Inhibitor 16	1.92339
PHF12	PHD Finger Protein 12	1.92217
ENOX1	Ecto-NOX Disulfide-Thiol Exchanger 1	1.92188
SMIM28	Small Integral Membrane Protein 28	1.92181
PEDS1-UBE2V	PEDS1-UBE2V1 Readthrough	1.92176
CDK13	Cyclin Dependent Kinase 13	1.92166
YEATS4	YEATS Domain Containing 4	1.92145
HAUS5	HAUS Augmin Like Complex Subunit 5	1.92113
SERP2	Stress Associated Endoplasmic Reticulum Protein Family Member 2	1.92036
ZNF354A	Zinc Finger Protein 354A	1.92005
ARHGEF19	Rho Guanine Nucleotide Exchange Factor 19	1.91917
GPX8	Glutathione Peroxidase 8 (Putative)	1.91872
GDF1	Growth Differentiation Factor 1	1.91855
NSF	N-Ethylmaleimide Sensitive Factor, Vesicle Fusing ATPase	1.91852
MIR493	MicroRNA 493	1.91793
FXYD1	FXYD Domain Containing Ion Transport Regulator 1	1.91790
MED20	Mediator Complex Subunit 20	1.91759
RNFT1	Ring Finger Protein, Transmembrane 1	1.91729
PCDHA7	Protocadherin Alpha 7	1.91712
PYCR2	Pyrroline-5-Carboxylate Reductase 2	1.91704
AARS2	Alanyl-TRNA Synthetase 2, Mitochondrial	1.91683
SPATA9	Spermatogenesis Associated 9	1.91663
MTFR1L	Mitochondrial Fission Regulator 1 Like	1.91544
PRKX	Protein Kinase X-Linked	1.91538
CPXCR1	CPX Chromosome Region Candidate 1	1.91485
ASB8	Ankyrin Repeat And SOCS Box Containing 8	1.91460
TMEM240	Transmembrane Protein 240	1.91428
SMIM38	Small Integral Membrane Protein 38	1.91390
PUS7	Pseudouridine Synthase 7	1.91345
AQP9	Aquaporin 9	1.91324
ACTR1A	Actin Related Protein 1A	1.91279
KIF1C	Kinesin Family Member 1C	1.91269
CCNDBP1	Cyclin D1 Binding Protein 1	1.91261
SMC2	Structural Maintenance Of Chromosomes 2	1.91224

RPL36AL	Ribosomal Protein L36a Like	1.91218
CALML6	Calmodulin Like 6	1.91207
EPS15	Epidermal Growth Factor Receptor Pathway Substrate 15	1.91166
RMND1	Required For Meiotic Nuclear Division 1 Homolog	1.91018
MAP4K4	Mitogen-Activated Protein Kinase Kinase Kinase Kinase 4	1.90950
ACSBG2	Acyl-CoA Synthetase Bubblegum Family Member 2	1.90950
MYG1	MYG1 Exonuclease	1.90950
DOK1	Docking Protein 1	1.90889
NRG4	Neuregulin 4	1.90881
AP3D1	Adaptor Related Protein Complex 3 Subunit Delta 1	1.90878
CLCN3	Chloride Voltage-Gated Channel 3	1.90856
H2BS1	H2B.S Histone 1	1.90813
SMPD2	Sphingomyelin Phosphodiesterase 2	1.90811
SRR	Serine Racemase	1.90793
SYTL1	Synaptotagmin Like 1	1.90777
FUT1	Fucosyltransferase 1 (H Blood Group)	1.90727
SH3BP1	SH3 Domain Binding Protein 1	1.90695
BAIAP2L2	BAR/IMD Domain Containing Adaptor Protein 2 Like 2	1.90631
C1orf74	Chromosome 1 Open Reading Frame 74	1.90585
SMIM3	Small Integral Membrane Protein 3	1.90558
TIAL1	TIA1 Cytotoxic Granule Associated RNA Binding Protein Like 1	1.90552
ORC5	Origin Recognition Complex Subunit 5	1.90547
SLC5A6	Solute Carrier Family 5 Member 6	1.90463
SLC52A2	Solute Carrier Family 52 Member 2	1.90399
TAC3	Tachykinin Precursor 3	1.90362
RFNG	RFNG O-Fucosylpeptide 3-Beta-N-Acetylglucosaminyltransferase	1.90331
PLAC9	Placenta Associated 9	1.90331
FARP2	FERM, ARH/RhoGEF And Pleckstrin Domain Protein 2	1.90325
ZNF646	Zinc Finger Protein 646	1.90303
ZG16B	Zymogen Granule Protein 16B	1.90275
MIR616	MicroRNA 616	1.90259
GLDN	Gliomedin	1.90257
FBXW2	F-Box And WD Repeat Domain Containing 2	1.90178
ENSG00000268810		1.90155
ENSG00000268743		1.90155
lnc-IQCH-7		1.90155
HSALNG0125884		1.90155
lnc-RIPOR3-2		1.90155
MK280269-030		1.90155

HSALNG0058163		1.90155
THEM4	Thioesterase Superfamily Member 4	1.90097
CRYBG1	Crystallin Beta-Gamma Domain Containing 1	1.90051
ADORA2B	Adenosine A2b Receptor	1.90050
ASAP2	ArfGAP With SH3 Domain, Ankyrin Repeat And PH Domain 2	1.90048
COA8	Cytochrome C Oxidase Assembly Factor 8	1.90034
CHST11	Carbohydrate Sulfotransferase 11	1.89963
TEN1	TEN1 Subunit Of CST Complex	1.89934
GDA	Guanine Deaminase	1.89909
TEDDM1	Transmembrane Epididymal Protein 1	1.89852
CCT6A	Chaperonin Containing TCP1 Subunit 6A	1.89795
HINFP	Histone H4 Transcription Factor	1.89759
SUB1	SUB1 Regulator Of Transcription	1.89700
PPP4C	Protein Phosphatase 4 Catalytic Subunit	1.89582
MIF-AS1	MIF Antisense RNA 1	1.89540
INTS3	Integrator Complex Subunit 3	1.89423
DAND5	DAN Domain BMP Antagonist Family Member 5	1.89358
IDH3A	Isocitrate Dehydrogenase (NAD(+)) 3 Catalytic Subunit Alpha	1.89324
EXOSC4	Exosome Component 4	1.89322
ADAT2	Adenosine Deaminase TRNA Specific 2	1.89244
UPK3B	Uroplakin 3B	1.89241
WDR6	WD Repeat Domain 6	1.89145
CD93	CD93 Molecule	1.89118
C3AR1	Complement C3a Receptor 1	1.89107
MCTS2P	MCTS Family Member 2, Pseudogene	1.89104
CRYBB3	Crystallin Beta B3	1.89072
LAYN	Layilin	1.89007
DNAJA1	DnaJ Heat Shock Protein Family (Hsp40) Member A1	1.88954
SMC1B	Structural Maintenance Of Chromosomes 1B	1.88947
TNIP2	TNFAIP3 Interacting Protein 2	1.88902
BAHCC1	BAH Domain And Coiled-Coil Containing 1	1.88901
SMU1	SMU1 DNA Replication Regulator And Spliceosomal Factor	1.88850
RPL34	Ribosomal Protein L34	1.88840
KRBOX4	KRAB Box Domain Containing 4	1.88830
SNORD89	Small Nucleolar RNA, C/D Box 89	1.88827
L2HGDH	L-2-Hydroxyglutarate Dehydrogenase	1.88812
SLC5A4	Solute Carrier Family 5 Member 4	1.88786
GGT6	Gamma-Glutamyltransferase 6	1.88775
MIR384	MicroRNA 384	1.88747

PARP10	Poly(ADP-Ribose) Polymerase Family Member 10	1.88678
ZNF500	Zinc Finger Protein 500	1.88675
FAM167A-AS1	FAM167A Antisense RNA 1	1.88664
IGKC	Immunoglobulin Kappa Constant	1.88645
CCDC85B	Coiled-Coil Domain Containing 85B	1.88620
PLSCR3	Phospholipid Scramblase 3	1.88486
DGKZ	Diacylglycerol Kinase Zeta	1.88482
DDX52	DExD-Box Helicase 52	1.88479
TSC22D2	TSC22 Domain Family Member 2	1.88478
OARD1	O-Acyl-ADP-Ribose Deacylase 1	1.88448
CD3E	CD3e Molecule	1.88439
HCG18	HLA Complex Group 18	1.88425
ZNF235	Zinc Finger Protein 235	1.88336
ZNF624	Zinc Finger Protein 624	1.88323
CAPZA1	Capping Actin Protein Of Muscle Z-Line Subunit Alpha 1	1.88293
SSNA1	SS Nuclear Autoantigen 1	1.88274
NME4	NME/NM23 Nucleoside Diphosphate Kinase 4	1.88196
VPS28	VPS28 Subunit Of ESCRT-I	1.88164
MEAK7	MTOR Associated Protein, Eak-7 Homolog	1.88131
BTN3A2	Butyrophilin Subfamily 3 Member A2	1.88130
LOC11263702[illegible]	ABO +36.0 Downstream Enhancer	1.88127
NUFIP1	Nuclear FMR1 Interacting Protein 1	1.88122
ADCYAP1R1	ADCYAP Receptor Type I	1.88118
RAB4B	RAB4B, Member RAS Oncogene Family	1.88058
HRH4	Histamine Receptor H4	1.88026
RCE1	Ras Converting CAAX Endopeptidase 1	1.88006
BAG2	BAG Cochaperone 2	1.87988
PEX10	Peroxisomal Biogenesis Factor 10	1.87929
MYT1	Myelin Transcription Factor 1	1.87881
RHOT2	Ras Homolog Family Member T2	1.87780
FCSK	Fucose Kinase	1.87772
EFCAB13	EF-Hand Calcium Binding Domain 13	1.87761
TTLL9	Tubulin Tyrosine Ligase Like 9	1.87716
METTL1	Methyltransferase Like 1	1.87675
NDUFS1	NADH:Ubiquinone Oxidoreductase Core Subunit S1	1.87666
MAL2	Mal, T Cell Differentiation Protein 2	1.87656
PSD	Pleckstrin And Sec7 Domain Containing	1.87652
IL37	Interleukin 37	1.87633
MAD2L1BP	MAD2L1 Binding Protein	1.87590
DNAI2	Dynein Axonemal Intermediate Chain 2	1.87571
COP1	COP1 E3 Ubiquitin Ligase	1.87568
TOB1-AS1	TOB1 Antisense RNA 1	1.87554
POU3F2	POU Class 3 Homeobox 2	1.87494

PIGO	Phosphatidylinositol Glycan Anchor Biosynthesis Class O	1.87493
LOC11814275[illegible]	GUCA1ANB-GUCA1A Readthrough	1.87470
THAP11	THAP Domain Containing 11	1.87419
WDR90	WD Repeat Domain 90	1.87374
DUS3L	Dihydrouridine Synthase 3 Like	1.87372
CDC42EP2	CDC42 Effector Protein 2	1.87353
CSHL1	Chorionic Somatomammotropin Hormone Like 1	1.87283
OSBPL5	Oxysterol Binding Protein Like 5	1.87266
DUSP8	Dual Specificity Phosphatase 8	1.87262
PNPO	Pyridoxamine 5'-Phosphate Oxidase	1.87224
ZBTB7B	Zinc Finger And BTB Domain Containing 7B	1.87175
PPAN	Peter Pan Homolog	1.87124
RAB37	RAB37, Member RAS Oncogene Family	1.87077
MIR1225	MicroRNA 1225	1.87056
FFAR2	Free Fatty Acid Receptor 2	1.87052
LPO	Lactoperoxidase	1.87038
CYBRD1	Cytochrome B Reductase 1	1.86896
RAPGEF6	Rap Guanine Nucleotide Exchange Factor 6	1.86851
ZNF593	Zinc Finger Protein 593	1.86818
UPP1	Uridine Phosphorylase 1	1.86804
CLDN25	Claudin 25	1.86725
SLC48A1	Solute Carrier Family 48 Member 1	1.86655
NACA	Nascent Polypeptide Associated Complex Subunit Alpha	1.86652
MFHAS1	Malignant Fibrous Histiocytoma Amplified Sequence 1	1.86652
TRIM2	Tripartite Motif Containing 2	1.86651
GATM	Glycine Amidinotransferase	1.86648
INO80	INO80 Complex ATPase Subunit	1.86631
PHACTR4	Phosphatase And Actin Regulator 4	1.86629
MED27	Mediator Complex Subunit 27	1.86523
ATP5PO	ATP Synthase Peripheral Stalk Subunit OSCP	1.86518
HCRTR2	Hypocretin Receptor 2	1.86476
AVPR1B	Arginine Vasopressin Receptor 1B	1.86464
RPP25	Ribonuclease P And MRP Subunit P25	1.86450
ZNF280B	Zinc Finger Protein 280B	1.86412
PITPNA	Phosphatidylinositol Transfer Protein Alpha	1.86392
GYG2	Glycogenin 2	1.86371
ADAMTS4	ADAM Metallopeptidase With Thrombospondin Type 1 Motif 4	1.86358
CDC34	Cell Division Cycle 34, Ubiqiutin Conjugating Enzyme	1.86266

ENOSF1	Enolase Superfamily Member 1	1.86266
IFT122	Intraflagellar Transport 122	1.86255
U2AF2	U2 Small Nuclear RNA Auxiliary Factor 2	1.86245
EIF3G	Eukaryotic Translation Initiation Factor 3 Subunit G	1.86217
HEATR1	HEAT Repeat Containing 1	1.86140
MIR6090	MicroRNA 6090	1.85992
SOCS5	Suppressor Of Cytokine Signaling 5	1.85965
PTAR1	Protein Prenyltransferase Alpha Subunit Repeat Containing 1	1.85901
KIFC3	Kinesin Family Member C3	1.85889
MAGEE2	MAGE Family Member E2	1.85815
SLC28A2	Solute Carrier Family 28 Member 2	1.85784
SERINC5	Serine Incorporator 5	1.85751
RCOR2	REST Corepressor 2	1.85751
CYP2G1P	Cytochrome P450 Family 2 Subfamily G Member 1, Pseudogene	1.85697
ENSG00000269843		1.85697
ENSG00000268030		1.85697
piR-34822-157		1.85697
BTG3	BTG Anti-Proliferation Factor 3	1.85658
DNM1	Dynamin 1	1.85620
CBLC	Cbl Proto-Oncogene C	1.85588
LAMA5-AS1	LAMA5 Antisense RNA 1	1.85514
ADAM28	ADAM Metallopeptidase Domain 28	1.85382
lnc-DYM-3		1.85270
POLR3C	RNA Polymerase III Subunit C	1.85266
MRPL27	Mitochondrial Ribosomal Protein L27	1.85247
KIF26B	Kinesin Family Member 26B	1.85218
SLC25A33	Solute Carrier Family 25 Member 33	1.85216
BACE1	Beta-Secretase 1	1.85185
KREMEN1	Kringle Containing Transmembrane Protein 1	1.85058
DBNDD1	Dysbindin Domain Containing 1	1.85040
TTC28	Tetratricopeptide Repeat Domain 28	1.85007
CCDC122	Coiled-Coil Domain Containing 122	1.84986
NUP54	Nucleoporin 54	1.84939
FBXO28	F-Box Protein 28	1.84924
ATP5MG	ATP Synthase Membrane Subunit G	1.84924
ARHGEF7	Rho Guanine Nucleotide Exchange Factor 7	1.84908
CNNM2	Cyclin And CBS Domain Divalent Metal Cation Transport Mediator 2	1.84891
TMEM45B	Transmembrane Protein 45B	1.84879
SCAF1	SR-Related CTD Associated Factor 1	1.84850
PKP3	Plakophilin 3	1.84818
PARP14	Poly(ADP-Ribose) Polymerase Family Member 14	1.84811

COX19	Cytochrome C Oxidase Assembly Factor COX19	1.84801
UBE2D2	Ubiquitin Conjugating Enzyme E2 D2	1.84767
RBM17	RNA Binding Motif Protein 17	1.84710
UNC13A	Unc-13 Homolog A	1.84704
PVALB	Parvalbumin	1.84689
PBDC1	Polysaccharide Biosynthesis Domain Containing 1	1.84679
CDC42BPB	CDC42 Binding Protein Kinase Beta	1.84659
TCEAL1	Transcription Elongation Factor A Like 1	1.84648
PIKFYVE	Phosphoinositide Kinase, FYVE-Type Zinc Finger Containing	1.84529
SETD1B	SET Domain Containing 1B, Histone Lysine Methyltransferase	1.84524
HDAC11	Histone Deacetylase 11	1.84478
NAA16	N-Alpha-Acetyltransferase 16, NatA Auxiliary Subunit	1.84475
ZNF181	Zinc Finger Protein 181	1.84426
ALDH1B1	Aldehyde Dehydrogenase 1 Family Member B1	1.84376
CAMK1	Calcium/Calmodulin Dependent Protein Kinase I	1.84359
ABCD4	ATP Binding Cassette Subfamily D Member 4	1.84338
MYO15A	Myosin XVA	1.84331
ADPRM	ADP-Ribose/CDP-Alcohol Diphosphatase, Manganese Dependent	1.84318
CLEC18A	C-Type Lectin Domain Family 18 Member A	1.84318
SCARF2	Scavenger Receptor Class F Member 2	1.84249
PDIA5	Protein Disulfide Isomerase Family A Member 5	1.84233
TRG	T Cell Receptor Gamma Locus	1.84227
GPC5	Glypican 5	1.84197
HOXC4	Homeobox C4	1.84173
PLEKHO2	Pleckstrin Homology Domain Containing O2	1.84096
PANK4	Pantothenate Kinase 4 (Inactive)	1.84078
INTS4	Integrator Complex Subunit 4	1.83981
SH3RF2	SH3 Domain Containing Ring Finger 2	1.83910
MAP7D1	MAP7 Domain Containing 1	1.83788
STX7	Syntaxin 7	1.83769
DERL3	Derlin 3	1.83757
USP21	Ubiquitin Specific Peptidase 21	1.83742
PMF1	Polyamine Modulated Factor 1	1.83590
COG3	Component Of Oligomeric Golgi Complex 3	1.83542
R3HDM2	R3H Domain Containing 2	1.83519

ATPAF2	ATP Synthase Mitochondrial F1 Complex Assembly Factor 2	1.83420
C1orf194	Chromosome 1 Open Reading Frame 194	1.83405
SYNC	Syncoilin, Intermediate Filament Protein	1.83404
HYKK	Hydroxylysine Kinase	1.83399
CCRL2	C-C Motif Chemokine Receptor Like 2	1.83365
RPL36	Ribosomal Protein L36	1.83339
CPLX2	Complexin 2	1.83299
DPT	Dermatopontin	1.83291
NOP9	NOP9 Nucleolar Protein	1.83245
ZNF285	Zinc Finger Protein 285	1.83214
ZNF45-AS1	ZNF45 Antisense RNA 1	1.83214
ENSG00000240731		1.83214
ENSG00000227775		1.83214
FAM131C	Family With Sequence Similarity 131 Member C	1.83191
FAM76A	Family With Sequence Similarity 76 Member A	1.83088
FCMR	Fc Fragment Of IgM Receptor	1.83088
ANKRD16	Ankyrin Repeat Domain 16	1.83081
PCDH17	Protocadherin 17	1.83057
INSRR	Insulin Receptor Related Receptor	1.83034
PGGHG	Protein-Glucosylgalactosylhydroxylysine Glucosidase	1.83031
CYP3A7	Cytochrome P450 Family 3 Subfamily A Member 7	1.83000
PGM1	Phosphoglucomutase 1	1.82921
EIF4H	Eukaryotic Translation Initiation Factor 4H	1.82910
NPY5R	Neuropeptide Y Receptor Y5	1.82709
METRNL	Meteorin Like, Glial Cell Differentiation Regulator	1.82650
EP400	E1A Binding Protein P400	1.82644
VPS51	VPS51 Subunit Of GARP Complex	1.82603
TEPSIN	TEPSIN Adaptor Related Protein Complex 4 Accessory Protein	1.82443
GNA15	G Protein Subunit Alpha 15	1.82435
MYO15B	Myosin XVB	1.82401
DNASE1L1	Deoxyribonuclease 1 Like 1	1.82391
EMC6	ER Membrane Protein Complex Subunit 6	1.82375
AATF	Apoptosis Antagonizing Transcription Factor	1.82270
SPINDOC	Spindlin Interactor And Repressor Of Chromatin Binding	1.82265
PLEKHM1	Pleckstrin Homology And RUN Domain Containing M1	1.82264

CEP85	Centrosomal Protein 85	1.82253
FBXL7	F-Box And Leucine Rich Repeat Protein 7	1.82218
RAB34	RAB34, Member RAS Oncogene Family	1.82149
S100A13	S100 Calcium Binding Protein A13	1.82090
CNIH4	Cornichon Family AMPA Receptor Auxiliary Protein 4	1.82057
CKM	Creatine Kinase, M-Type	1.82048
ITGA6-AS1	ITGA6 Antisense RNA 1	1.82036
S1PR2	Sphingosine-1-Phosphate Receptor 2	1.82008
RAB18	RAB18, Member RAS Oncogene Family	1.81986
CLEC12A	C-Type Lectin Domain Family 12 Member A	1.81978
MAFF	MAF BZIP Transcription Factor F	1.81903
TRIB3	Tribbles Pseudokinase 3	1.81893
RAB6B	RAB6B, Member RAS Oncogene Family	1.81891
KMT5C	Lysine Methyltransferase 5C	1.81870
CHST8	Carbohydrate Sulfotransferase 8	1.81863
CDH24	Cadherin 24	1.81842
MIR105-1	MicroRNA 105-1	1.81808
UNC5A	Unc-5 Netrin Receptor A	1.81787
CD6	CD6 Molecule	1.81780
DHODH	Dihydroorotate Dehydrogenase (Quinone)	1.81777
ECHS1	Enoyl-CoA Hydratase, Short Chain 1	1.81743
ARRDC2	Arrestin Domain Containing 2	1.81738
CRYBB2	Crystallin Beta B2	1.81695
PTCD1	Pentatricopeptide Repeat Domain 1	1.81680
AZU1	Azurocidin 1	1.81582
TTC9	Tetratricopeptide Repeat Domain 9	1.81564
CHMP6	Charged Multivesicular Body Protein 6	1.81542
ZNF428	Zinc Finger Protein 428	1.81492
DCP1B	Decapping MRNA 1B	1.81359
SLC24A1	Solute Carrier Family 24 Member 1	1.81355
SFMBT1	Scm Like With Four Mbt Domains 1	1.81324
HTR2B	5-Hydroxytryptamine Receptor 2B	1.81315
PPP2R3C	Protein Phosphatase 2 Regulatory Subunit B"Gamma	1.81292
TSKU	Tsukushi, Small Leucine Rich Proteoglycan	1.81276
RHOBTB3	Rho Related BTB Domain Containing 3	1.81275
YLPM1	YLP Motif Containing 1	1.81267
NDUFA11	NADH:Ubiquinone Oxidoreductase Subunit A11	1.81244
MRPL54	Mitochondrial Ribosomal Protein L54	1.81228
ITPRIP-AS1	ITPRIP Antisense RNA 1	1.81221

RNF4	Ring Finger Protein 4	1.81213
PRKAB2	Protein Kinase AMP-Activated Non-Catalytic Subunit Beta 2	1.81160
NCBP1	Nuclear Cap Binding Protein Subunit 1	1.81155
CCT8	Chaperonin Containing TCP1 Subunit 8	1.81145
GOSR2	Golgi SNAP Receptor Complex Member 2	1.81112
SMIM11A	Small Integral Membrane Protein 11A	1.81098
TMEM220-AS	TMEM220 Antisense RNA 1	1.81098
RPL36P4	Ribosomal Protein L36 Pseudogene 4	1.81098
ENSG00000259314		1.81098
ENSG00000267748		1.81098
ENSG00000285220		1.81098
SNORD151	Small Nucleolar RNA, C/D Box 151	1.81098
HSALNG0079123		1.81098
MN298114-127		1.81098
HSALNG0102091		1.81098
AK4	Adenylate Kinase 4	1.81078
PYGL	Glycogen Phosphorylase L	1.81053
EXOSC2	Exosome Component 2	1.81030
ODAD4	Outer Dynein Arm Docking Complex Subunit 4	1.81026
AMOTL1	Angiomotin Like 1	1.81001
FRAS1	Fraser Extracellular Matrix Complex Subunit 1	1.80924
KHDRBS3	KH RNA Binding Domain Containing, Signal Transduction Associated 3	1.80887
PAK5	P21 (RAC1) Activated Kinase 5	1.80874
C4BPA	Complement Component 4 Binding Protein Alpha	1.80872
TNRC18	Trinucleotide Repeat Containing 18	1.80833
FBXW9	F-Box And WD Repeat Domain Containing 9	1.80775
SOWAHB	Sosondowah Ankyrin Repeat Domain Family Member B	1.80632
CYC1	Cytochrome C1	1.80632
PELI3	Pellino E3 Ubiquitin Protein Ligase Family Member 3	1.80630
EMILIN1	Elastin Microfibril Interfacer 1	1.80593
KLHDC4	Kelch Domain Containing 4	1.80591
NUDT13	Nudix Hydrolase 13	1.80591
RELCH	RAB11 Binding And LisH Domain, Coiled-Coil And HEAT Repeat Containing	1.80588
DIAPH3	Diaphanous Related Formin 3	1.80546
CNR2	Cannabinoid Receptor 2	1.80505
MIR544A	MicroRNA 544a	1.80496
MYOZ2	Myozenin 2	1.80366
SYNM	Synemin	1.80330

BSPRY	B-Box And SPRY Domain Containing	1.80288
SUOX	Sulfite Oxidase	1.80243
APCS	Amyloid P Component, Serum	1.80157
EPB41L4B	Erythrocyte Membrane Protein Band 4.1 Like 4B	1.80124
DNAJC5	DnaJ Heat Shock Protein Family (Hsp40) Member C5	1.80121
NADK	NAD Kinase	1.80088
TMEM256	Transmembrane Protein 256	1.80080
PARP2	Poly(ADP-Ribose) Polymerase 2	1.80060
SEL1L	SEL1L Adaptor Subunit Of ERAD E3 Ubiquitin Ligase	1.79918
ACTRT3	Actin Related Protein T3	1.79898
NDUFA6	NADH:Ubiquinone Oxidoreductase Subunit A6	1.79851
APH1A	Aph-1 Homolog A, Gamma-Secretase Subunit	1.79849
CPT1B	Carnitine Palmitoyltransferase 1B	1.79836
SYTL3	Synaptotagmin Like 3	1.79726
GTF2IRD2	GTF2I Repeat Domain Containing 2	1.79725
ZIK1	Zinc Finger Protein Interacting With K Protein 1	1.79689
SAC3D1	SAC3 Domain Containing 1	1.79653
GLT8D1	Glycosyltransferase 8 Domain Containing 1	1.79642
EHBP1L1	EH Domain Binding Protein 1 Like 1	1.79630
H1-2	H1.2 Linker Histone, Cluster Member	1.79588
SLC9A3	Solute Carrier Family 9 Member A3	1.79579
UBIAD1	UbiA Prenyltransferase Domain Containing 1	1.79573
SHF	Src Homology 2 Domain Containing F	1.79539
DELE1	DAP3 Binding Cell Death Enhancer 1	1.79513
DDR1-DT	DDR1 Divergent Transcript	1.79513
NFATC3	Nuclear Factor Of Activated T Cells 3	1.79511
AP5B1	Adaptor Related Protein Complex 5 Subunit Beta 1	1.79484
PRELP	Proline And Arginine Rich End Leucine Rich Repeat Protein	1.79462
PSMD8	Proteasome 26S Subunit, Non-ATPase 8	1.79462
NRXN2	Neurexin 2	1.79405
HSALNG0121462		1.79394
HSALNG0121463-001		1.79394
THAP7	THAP Domain Containing 7	1.79359
C13orf46	Chromosome 13 Open Reading Frame 46	1.79353
KIR2DL3	Killer Cell Immunoglobulin Like Receptor, Two Ig Domains And Long Cytoplasmic Tail 3	1.79322

LINC02883	Long Intergenic Non-Protein Coding RNA 2883	1.79312
DNAJC6	DnaJ Heat Shock Protein Family (Hsp40) Member C6	1.79284
ANAPC11	Anaphase Promoting Complex Subunit 11	1.79276
DDX50	DExD-Box Helicase 50	1.79210
GTPBP2	GTP Binding Protein 2	1.79173
TBCE	Tubulin Folding Cofactor E	1.79161
RGMA	Repulsive Guidance Molecule BMP Co-Receptor A	1.79151
ACVR2B	Activin A Receptor Type 2B	1.79141
PRMT5-DT	PRMT5 Divergent Transcript	1.79079
SCAMP2	Secretory Carrier Membrane Protein 2	1.79040
HIPK3	Homeodomain Interacting Protein Kinase 3	1.79035
LARP4	La Ribonucleoprotein 4	1.78979
CADM3	Cell Adhesion Molecule 3	1.78924
TCEAL7	Transcription Elongation Factor A Like 7	1.78913
CXXC4	CXXC Finger Protein 4	1.78911
CACNA1B	Calcium Voltage-Gated Channel Subunit Alpha1 B	1.78893
DXO	Decapping Exoribonuclease	1.78859
FKBP15	FKBP Prolyl Isomerase Family Member 15	1.78850
C11orf53	Chromosome 11 Open Reading Frame 53	1.78844
USP42	Ubiquitin Specific Peptidase 42	1.78754
KIAA0586	KIAA0586	1.78749
UBAP2	Ubiquitin Associated Protein 2	1.78743
PPP1CC	Protein Phosphatase 1 Catalytic Subunit Gamma	1.78723
SAMD10	Sterile Alpha Motif Domain Containing 10	1.78714
HLA-DRB6	Major Histocompatibility Complex, Class II, DR Beta 6 (Pseudogene)	1.78644
FKRP	Fukutin Related Protein	1.78640
UBE2D3	Ubiquitin Conjugating Enzyme E2 D3	1.78611
SLC35A4	Solute Carrier Family 35 Member A4	1.78599
CFAP58	Cilia And Flagella Associated Protein 58	1.78594
ZYX	Zyxin	1.78593
TCTN2	Tectonic Family Member 2	1.78513
IQSEC1	IQ Motif And Sec7 Domain ArfGEF 1	1.78475
LINC01114	Long Intergenic Non-Protein Coding RNA 1114	1.78463
NAA20	N-Alpha-Acetyltransferase 20, NatB Catalytic Subunit	1.78462
NME7	NME/NM23 Family Member 7	1.78460

ARMC7	Armadillo Repeat Containing 7	1.78460
KCNK6	Potassium Two Pore Domain Channel Subfamily K Member 6	1.78393
MGAT5B	Alpha-1,6-Mannosylglycoprotein 6-Beta-N-Acetylglucosaminyltransferase B	1.78387
RBM42	RNA Binding Motif Protein 42	1.78374
KIAA0232	KIAA0232	1.78374
SRRM2	Serine/Arginine Repetitive Matrix 2	1.78357
HLA-DQB2	Major Histocompatibility Complex, Class II, DQ Beta 2	1.78311
CCDC61	Coiled-Coil Domain Containing 61	1.78300
COX8A	Cytochrome C Oxidase Subunit 8A	1.78276
PAQR7	Progestin And AdipoQ Receptor Family Member 7	1.78269
LSINCT5	Long Stress-Induced Non-Coding Transcript 5	1.78256
MFNG	MFNG O-Fucosylpeptide 3-Beta-N-Acetylglucosaminyltransferase	1.78180
SYNGR1	Synaptogyrin 1	1.78118
B3GALT4	Beta-1,3-Galactosyltransferase 4	1.78042
LMF2	Lipase Maturation Factor 2	1.78031
PIGN	Phosphatidylinositol Glycan Anchor Biosynthesis Class N	1.77896
SMIM2	Small Integral Membrane Protein 2	1.77894
ZNF226	Zinc Finger Protein 226	1.77874
FSTL1	Follistatin Like 1	1.77728
FBXO27	F-Box Protein 27	1.77685
KBTBD12	Kelch Repeat And BTB Domain Containing 12	1.77685
LILRB4	Leukocyte Immunoglobulin Like Receptor B4	1.77651
FFAR4	Free Fatty Acid Receptor 4	1.77622
TMEM209	Transmembrane Protein 209	1.77600
ANKRD27	Ankyrin Repeat Domain 27	1.77586
RTN3	Reticulon 3	1.77567
UHMK1	U2AF Homology Motif Kinase 1	1.77510
SEMA6D	Semaphorin 6D	1.77505
RSPRY1	Ring Finger And SPRY Domain Containing 1	1.77501
CCNB1IP1	Cyclin B1 Interacting Protein 1	1.77492
SNHG32	Small Nucleolar RNA Host Gene 32	1.77396
C11orf49	Chromosome 11 Open Reading Frame 49	1.77383
SPRR1B	Small Proline Rich Protein 1B	1.77372
MT1A	Metallothionein 1A	1.77338
WBP4	WW Domain Binding Protein 4	1.77316
CWC25	CWC25 Spliceosome Associated Protein Homolog	1.77280

PSMG3-AS1	PSMG3 Antisense RNA 1 (Head To Head)	1.77260
NEB	Nebulin	1.77226
PPP1R16A	Protein Phosphatase 1 Regulatory Subunit 16A	1.77223
RNF31	Ring Finger Protein 31	1.77194
BHLHE23	Basic Helix-Loop-Helix Family Member E23	1.77183
LOC10537104[illegible]	Uncharacterized LOC105371049	1.77086
ADRA1D	Adrenoceptor Alpha 1D	1.77059
FERMT3	FERM Domain Containing Kindlin 3	1.76962
MICU1	Mitochondrial Calcium Uptake 1	1.76935
RIN1	Ras And Rab Interactor 1	1.76849
APH1B	Aph-1 Homolog B, Gamma-Secretase Subunit	1.76847
RNF19B	Ring Finger Protein 19B	1.76815
RPL36AP37	Ribosomal Protein L36a Pseudogene 37	1.76815
ATAD3C	ATPase Family AAA Domain Containing 3C	1.76773
MTHFD2	Methylenetetrahydrofolate Dehydrogenase (NADP+ Dependent) 2, Methenyltetrahydrofolate Cyclohydrolase	1.76768
NLRP5	NLR Family Pyrin Domain Containing 5	1.76762
DYRK3	Dual Specificity Tyrosine Phosphorylation Regulated Kinase 3	1.76748
IL17RB	Interleukin 17 Receptor B	1.76717
GDAP1L1	Ganglioside Induced Differentiation Associated Protein 1 Like 1	1.76711
KYAT1	Kynurenine Aminotransferase 1	1.76676
SLC37A1	Solute Carrier Family 37 Member 1	1.76634
MLXIP	MLX Interacting Protein	1.76611
ELMO3	Engulfment And Cell Motility 3	1.76514
MIXL1	Mix Paired-Like Homeobox	1.76513
WDR74	WD Repeat Domain 74	1.76498
GCNT4	Glucosaminyl (N-Acetyl) Transferase 4	1.76450
PDE8B	Phosphodiesterase 8B	1.76422
ABALON	Apoptotic BCL2L1-Antisense Long Non-Coding RNA	1.76419
CATSPER3	Cation Channel Sperm Associated 3	1.76404
MAPK1IP1L	Mitogen-Activated Protein Kinase 1 Interacting Protein 1 Like	1.76361
HLA-J	Major Histocompatibility Complex, Class I, J (Pseudogene)	1.76342
LOC10192753[illegible]	Uncharacterized LOC101927533	1.76342
HLA-W	Major Histocompatibility Complex, Class I, W (Pseudogene)	1.76342
ENSG00000262147		1.76342

ENSG00000226332		1.76342
ENSG00000260290		1.76342
piR-52490		1.76342
HSALNG0102093		1.76342
MK280607-096		1.76342
piR-50208		1.76342
TMOD1	Tropomodulin 1	1.76285
MIR1236	MicroRNA 1236	1.76274
DGKA	Diacylglycerol Kinase Alpha	1.76234
PCGF6	Polycomb Group Ring Finger 6	1.76197
IRGQ	Immunity Related GTPase Q	1.76153
ZBTB32	Zinc Finger And BTB Domain Containing 32	1.76151
RBBP8NL	RBBP8 N-Terminal Like	1.76135
PCGF1	Polycomb Group Ring Finger 1	1.76133
LOC11000631[illegible]	Serine/Threonine Kinase 11 Intron 1 Alu-Mediated Recombination Region	1.76108
LOC11000631[illegible]	Serine/Threonine Kinase 11 Intron 3 Alu-Mediated Recombination Region	1.76108
FBXL2	F-Box And Leucine Rich Repeat Protein 2	1.76030
GALNT4	Polypeptide N-Acetylgalactosaminyltransferase 4	1.75983
PLEKHG3	Pleckstrin Homology And RhoGEF Domain Containing G3	1.75973
RGS1	Regulator Of G Protein Signaling 1	1.75952
LPCAT3	Lysophosphatidylcholine Acyltransferase 3	1.75944
FSD1	Fibronectin Type III And SPRY Domain Containing 1	1.75741
RGSL1	Regulator Of G Protein Signaling Like 1	1.75678
NALCN	Sodium Leak Channel, Non-Selective	1.75667
YIPF3	Yip1 Domain Family Member 3	1.75657
METTL16	Methyltransferase Like 16	1.75584
PRR15L	Proline Rich 15 Like	1.75557
SLC4A9	Solute Carrier Family 4 Member 9	1.75557
NAT9	N-Acetyltransferase 9 (Putative)	1.75536
BTLA	B And T Lymphocyte Associated	1.75452
CCDC9	Coiled-Coil Domain Containing 9	1.75436
ATP6V0C	ATPase H+ Transporting V0 Subunit C	1.75375
SAYSD1	SAYSVFN Motif Domain Containing 1	1.75273
CCZ1	CCZ1 Homolog, Vacuolar Protein Trafficking And Biogenesis Associated	1.75242
IQCC	IQ Motif Containing C	1.75184
DOK2	Docking Protein 2	1.75125
MYL12A	Myosin Light Chain 12A	1.75074
SDF2	Stromal Cell Derived Factor 2	1.75069
TRAF3IP1	TRAF3 Interacting Protein 1	1.75051
SLC39A13	Solute Carrier Family 39 Member 13	1.75046

ATG2A	Autophagy Related 2A	1.75039
SLC18A1	Solute Carrier Family 18 Member A1	1.75022
MIF4GD	MIF4G Domain Containing	1.75000
DOLK	Dolichol Kinase	1.74979
PLAAT3	Phospholipase A And Acyltransferase 3	1.74975
RRP36	Ribosomal RNA Processing 36	1.74963
SYNGR2	Synaptogyrin 2	1.74892
ZNF526	Zinc Finger Protein 526	1.74873
GAN	Gigaxonin	1.74872
AKAP1	A-Kinase Anchoring Protein 1	1.74802
GABARAPL3	GABA Type A Receptor Associated Protein Like 3 Pseudogene	1.74791
CLK3	CDC Like Kinase 3	1.74756
SERTAD1	SERTA Domain Containing 1	1.74740
EGR3	Early Growth Response 3	1.74730
PSMG3	Proteasome Assembly Chaperone 3	1.74704
CLDN16	Claudin 16	1.74675
TIMM10	Translocase Of Inner Mitochondrial Membrane 10	1.74637
PKNOX2	PBX/Knotted 1 Homeobox 2	1.74613
STAG3L5P	Stromal Antigen 3-Like 5 Pseudogene	1.74607
FUBP3	Far Upstream Element Binding Protein 3	1.74553
TMEM126B	Transmembrane Protein 126B	1.74535
GLYR1	Glyoxylate Reductase 1 Homolog	1.74527
TRPC5	Transient Receptor Potential Cation Channel Subfamily C Member 5	1.74491
THOC6	THO Complex 6	1.74435
C15orf48	Chromosome 15 Open Reading Frame 48	1.74429
GNAO1	G Protein Subunit Alpha O1	1.74428
INO80E	INO80 Complex Subunit E	1.74405
MDP1	Magnesium Dependent Phosphatase 1	1.74400
SLC34A3	Solute Carrier Family 34 Member 3	1.74350
DYDC2	DPY30 Domain Containing 2	1.74271
INPP5A	Inositol Polyphosphate-5-Phosphatase A	1.74238
PICK1	Protein Interacting With PRKCA 1	1.74197
TRNP1	TMF1 Regulated Nuclear Protein 1	1.74196
SLC1A4	Solute Carrier Family 1 Member 4	1.74144
RMDN3	Regulator Of Microtubule Dynamics 3	1.74119
TMPRSS15	Transmembrane Serine Protease 15	1.74118
AZIN2	Antizyme Inhibitor 2	1.74102
TBC1D10B	TBC1 Domain Family Member 10B	1.74040
UBXN1	UBX Domain Protein 1	1.74023
CEP41	Centrosomal Protein 41	1.74000
ACTL6B	Actin Like 6B	1.73982
PPT2	Palmitoyl-Protein Thioesterase 2	1.73966
NFE2	Nuclear Factor, Erythroid 2	1.73947
TJP3	Tight Junction Protein 3	1.73911

RERE-AS1	RERE Antisense RNA 1	1.73868
GFPT2	Glutamine-Fructose-6-Phosphate Transaminase 2	1.73835
SOCS6	Suppressor Of Cytokine Signaling 6	1.73788
MIR3679	MicroRNA 3679	1.73761
MIR802	MicroRNA 802	1.73754
MESP1	Mesoderm Posterior BHLH Transcription Factor 1	1.73748
PDF	Peptide Deformylase, Mitochondrial	1.73700
ACOT1	Acyl-CoA Thioesterase 1	1.73686
IFFO1	Intermediate Filament Family Orphan 1	1.73683
MYL6	Myosin Light Chain 6	1.73576
PART1	Prostate Androgen-Regulated Transcript 1	1.73491
PDHA2	Pyruvate Dehydrogenase E1 Subunit Alpha 2	1.73469
KLHL22	Kelch Like Family Member 22	1.73465
SEMA4G	Semaphorin 4G	1.73420
B4GAT1	Beta-1,4-Glucuronyltransferase 1	1.73321
LDHAL6A	Lactate Dehydrogenase A Like 6A	1.73312
WDR91	WD Repeat Domain 91	1.73285
FAM243A	Family With Sequence Similarity 243 Member A	1.73284
EHD1	EH Domain Containing 1	1.73279
SLX4IP	SLX4 Interacting Protein	1.73256
MIR374B	MicroRNA 374b	1.73236
CASP14	Caspase 14	1.73187
PAGE2B	PAGE Family Member 2B	1.73166
ANKS1A	Ankyrin Repeat And Sterile Alpha Motif Domain Containing 1A	1.73136
NPL	N-Acetylneuraminate Pyruvate Lyase	1.73133
OTUD3	OTU Deubiquitinase 3	1.73101
SELENOM	Selenoprotein M	1.73100
LAMB4	Laminin Subunit Beta 4	1.73079
LRPPRC	Leucine Rich Pentatricopeptide Repeat Containing	1.73022
TRIO	Trio Rho Guanine Nucleotide Exchange Factor	1.72958
H3C15	H3 Clustered Histone 15	1.72943
FBXO32	F-Box Protein 32	1.72891
PLEKHA2	Pleckstrin Homology Domain Containing A2	1.72884
NTAN1	N-Terminal Asparagine Amidase	1.72861
SLC38A1	Solute Carrier Family 38 Member 1	1.72849
DDAH2	Dimethylarginine Dimethylaminohydrolase 2	1.72835
CRAMP1	Cramped Chromatin Regulator Homolog 1	1.72834
NPEPPS	Aminopeptidase Puromycin Sensitive	1.72825

EEF1E1	Eukaryotic Translation Elongation Factor 1 Epsilon 1	1.72762
COPB2	COPI Coat Complex Subunit Beta 2	1.72742
GPR108	G Protein-Coupled Receptor 108	1.72720
CLDN9	Claudin 9	1.72713
CDKN2AIP	CDKN2A Interacting Protein	1.72675
RYR3	Ryanodine Receptor 3	1.72670
GNG4	G Protein Subunit Gamma 4	1.72651
RGMB	Repulsive Guidance Molecule BMP Co-Receptor B	1.72560
P3R3URF-PIK3	P3R3URF-PIK3R3 Readthrough	1.72518
INTS5	Integrator Complex Subunit 5	1.72475
ARL16	ADP Ribosylation Factor Like GTPase 16	1.72434
EEF1AKNMT	EEF1A Lysine And N-Terminal Methyltransferase	1.72429
ZNF518A	Zinc Finger Protein 518A	1.72425
C6orf47	Chromosome 6 Open Reading Frame 47	1.72359
TTC28-AS1	TTC28 Antisense RNA 1	1.72285
LOC10192800	Uncharacterized LOC101928004	1.72285
ENSG00000230092		1.72285
ENSG00000269737		1.72285
HSALNG0047835		1.72285
lnc-FAM109A-1		1.72285
KSR1	Kinase Suppressor Of Ras 1	1.72220
RRAD	RRAD, Ras Related Glycolysis Inhibitor And Calcium Channel Regulator	1.72169
MED22	Mediator Complex Subunit 22	1.72053
SNAP23	Synaptosome Associated Protein 23	1.72035
SARM1	Sterile Alpha And TIR Motif Containing 1	1.72014
LAMP3	Lysosomal Associated Membrane Protein 3	1.71996
LILRB2	Leukocyte Immunoglobulin Like Receptor B2	1.71974
HSD17B13	Hydroxysteroid 17-Beta Dehydrogenase 13	1.71947
PALM2AKAP2	PALM2 And AKAP2 Fusion	1.71933
PYM1	PYM Homolog 1, Exon Junction Complex Associated Factor	1.71883
CASKIN2	CASK Interacting Protein 2	1.71866
GTF2IRD2B	GTF2I Repeat Domain Containing 2B	1.71786
DDAH1	Dimethylarginine Dimethylaminohydrolase 1	1.71777
MRRF	Mitochondrial Ribosome Recycling Factor	1.71776
FOXJ3	Forkhead Box J3	1.71761
VAMP7	Vesicle Associated Membrane Protein 7	1.71738
PNMA8A	PNMA Family Member 8A	1.71717

PITX1-AS1	PITX1 Antisense RNA 1	1.71707
TTC32	Tetratricopeptide Repeat Domain 32	1.71631
SDAD1	SDA1 Domain Containing 1	1.71596
TCF25	Transcription Factor 25	1.71518
ZNF781	Zinc Finger Protein 781	1.71414
ETF1P1	Eukaryotic Translation Termination Factor 1 Pseudogene 1	1.71414
HLA-U	Major Histocompatibility Complex, Class I, U (Pseudogene)	1.71414
ENSG00000268707		1.71414
ENSG00000234389		1.71414
ENSG00000232807		1.71414
PAIP1P1	Poly(A) Binding Protein Interacting Protein 1 Pseudogene 1	1.71414
HNRNPA1P35	Heterogeneous Nuclear Ribonucleoprotein A1 Pseudogene 35	1.71414
RNU6-312P	RNA, U6 Small Nuclear 312, Pseudogene	1.71414
RNU6-1180P	RNA, U6 Small Nuclear 1180, Pseudogene	1.71414
lnc-TFEB-4		1.71414
ENSG00000230521		1.71414
lnc-SMPD3-4		1.71414
PPP2R5E	Protein Phosphatase 2 Regulatory Subunit B'Epsilon	1.71344
LIX1L-AS1	LIX1L Antisense RNA 1	1.71313
DMWD	DM1 Locus, WD Repeat Containing	1.71259
NFAT5	Nuclear Factor Of Activated T Cells 5	1.71240
C8orf82	Chromosome 8 Open Reading Frame 82	1.71234
RAB11B-AS1	RAB11B Antisense RNA 1	1.71232
PPP6R2	Protein Phosphatase 6 Regulatory Subunit 2	1.71214
COLGALT1	Collagen Beta(1-O)Galactosyltransferase 1	1.71187
MAMDC2	MAM Domain Containing 2	1.71187
MIR935	MicroRNA 935	1.71077
REXO4	REX4 Homolog, 3'-5' Exonuclease	1.71023
NR1D1	Nuclear Receptor Subfamily 1 Group D Member 1	1.71004
TAF1C	TATA-Box Binding Protein Associated Factor, RNA Polymerase I Subunit C	1.70955
ALG12	ALG12 Alpha-1,6-Mannosyltransferase	1.70906
TJAP1	Tight Junction Associated Protein 1	1.70882
TANGO2	Transport And Golgi Organization 2 Homolog	1.70817
CYB5R2	Cytochrome B5 Reductase 2	1.70805
PCOLCE	Procollagen C-Endopeptidase Enhancer	1.70791
SEC23IP	SEC23 Interacting Protein	1.70738
CIPC	CLOCK Interacting Pacemaker	1.70726

NME6	NME/NM23 Nucleoside Diphosphate Kinase 6	1.70710
DUSP19	Dual Specificity Phosphatase 19	1.70679
NPFF	Neuropeptide FF-Amide Peptide Precursor	1.70661
SEC22A	SEC22 Homolog A, Vesicle Trafficking Protein	1.70656
COX20	Cytochrome C Oxidase Assembly Factor COX20	1.70656
SRSF9	Serine And Arginine Rich Splicing Factor 9	1.70648
BCAT1	Branched Chain Amino Acid Transaminase 1	1.70643
ZDHHC18	Zinc Finger DHHC-Type Palmitoyltransferase 18	1.70541
TPRKB	TP53RK Binding Protein	1.70534
TENT5A	Terminal Nucleotidyltransferase 5A	1.70482
RNASE4	Ribonuclease A Family Member 4	1.70469
INPP4B	Inositol Polyphosphate-4-Phosphatase Type II B	1.70454
DPP8	Dipeptidyl Peptidase 8	1.70417
LOC10828117	SOX2 5' Regulatory Region	1.70376
GNRHR2	Gonadotropin Releasing Hormone Receptor 2 (Pseudogene)	1.70327
ENSG00000124593		1.70318
SNX6	Sorting Nexin 6	1.70311
LINC00476	Long Intergenic Non-Protein Coding RNA 476	1.70284
ZNF250	Zinc Finger Protein 250	1.70197
CEACAM4	CEA Cell Adhesion Molecule 4	1.70149
PANX2	Pannexin 2	1.70136
PPP1R3C	Protein Phosphatase 1 Regulatory Subunit 3C	1.70102
YIF1A	Yip1 Interacting Factor Homolog A, Membrane Trafficking Protein	1.70081
SAE1	SUMO1 Activating Enzyme Subunit 1	1.70073
SLC30A10	Solute Carrier Family 30 Member 10	1.69990
CORO7	Coronin 7	1.69990
PRRT1B	Proline Rich Transmembrane Protein 1B	1.69963
KIAA0754	KIAA0754	1.69954
C5orf38	Chromosome 5 Open Reading Frame 38	1.69929
SLC7A8	Solute Carrier Family 7 Member 8	1.69918
VAT1	Vesicle Amine Transport 1	1.69917
LAMTOR3	Late Endosomal/Lysosomal Adaptor, MAPK And MTOR Activator 3	1.69878
ADAD1	Adenosine Deaminase Domain Containing 1	1.69868
KRTCAP3	Keratinocyte Associated Protein 3	1.69867
SMYD4	SET And MYND Domain Containing 4	1.69806

TNFAIP8L3	TNF Alpha Induced Protein 8 Like 3	1.69802
MED16	Mediator Complex Subunit 16	1.69799
IL17C	Interleukin 17C	1.69779
LMF1	Lipase Maturation Factor 1	1.69771
KIF20A	Kinesin Family Member 20A	1.69768
ABCB8	ATP Binding Cassette Subfamily B Member 8	1.69749
ENY2	ENY2 Transcription And Export Complex 2 Subunit	1.69720
NMUR1	Neuromedin U Receptor 1	1.69672
PRICKLE2	Prickle Planar Cell Polarity Protein 2	1.69654
ZNF787	Zinc Finger Protein 787	1.69566
SNRPD2	Small Nuclear Ribonucleoprotein D2 Polypeptide	1.69565
IFIT3	Interferon Induced Protein With Tetratricopeptide Repeats 3	1.69529
SEMA4C	Semaphorin 4C	1.69522
DDX31	DEAD-Box Helicase 31	1.69451
C6orf132	Chromosome 6 Open Reading Frame 132	1.69447
MIR216B	MicroRNA 216b	1.69440
CSDE1	Cold Shock Domain Containing E1	1.69438
TPRN	Taperin	1.69399
UCK1	Uridine-Cytidine Kinase 1	1.69381
CCDC137	Coiled-Coil Domain Containing 137	1.69381
GLRA1	Glycine Receptor Alpha 1	1.69321
PRR22	Proline Rich 22	1.69309
TMC4	Transmembrane Channel Like 4	1.69309
FBXL12	F-Box And Leucine Rich Repeat Protein 12	1.69262
MATN3	Matrilin 3	1.69257
ZNF133	Zinc Finger Protein 133	1.69228
CASTOR1	Cytosolic Arginine Sensor For MTORC1 Subunit 1	1.69220
CCDC117	Coiled-Coil Domain Containing 117	1.69205
ZNF768	Zinc Finger Protein 768	1.69195
TECPR2	Tectonin Beta-Propeller Repeat Containing 2	1.69175
WLS	Wnt Ligand Secretion Mediator	1.69108
PLOD3	Procollagen-Lysine,2-Oxoglutarate 5-Dioxygenase 3	1.69090
LSG1	Large 60S Subunit Nuclear Export GTPase 1	1.69056
AMDHD2	Amidohydrolase Domain Containing 2	1.69038
EDC4	Enhancer Of MRNA Decapping 4	1.69033
ZNF3	Zinc Finger Protein 3	1.69015
TSPAN10	Tetraspanin 10	1.69015
BAZ1A	Bromodomain Adjacent To Zinc Finger Domain 1A	1.68973

SH3PXD2A-AS	SH3PXD2A Antisense RNA 1	1.68844
ELMOD3	ELMO Domain Containing 3	1.68793
ISG20L2	Interferon Stimulated Exonuclease Gene 20 Like 2	1.68781
PPFIA3	PTPRF Interacting Protein Alpha 3	1.68720
TRIM52	Tripartite Motif Containing 52	1.68719
EXOC3L4	Exocyst Complex Component 3 Like 4	1.68684
GDAP2	Ganglioside Induced Differentiation Associated Protein 2	1.68605
DYDC1	DPY30 Domain Containing 1	1.68605
TTLL1	Tubulin Tyrosine Ligase Like 1	1.68601
NUBP2	Nucleotide Binding Protein 2	1.68585
DGAT1	Diacylglycerol O-Acyltransferase 1	1.68584
IP6K2	Inositol Hexakisphosphate Kinase 2	1.68553
LY6E	Lymphocyte Antigen 6 Family Member E	1.68546
TEX19	Testis Expressed 19	1.68505
UBE3B	Ubiquitin Protein Ligase E3B	1.68499
TMEM268	Transmembrane Protein 268	1.68473
LRRC32	Leucine Rich Repeat Containing 32	1.68346
ALAS1	5'-Aminolevulinate Synthase 1	1.68317
PLEKHG4	Pleckstrin Homology And RhoGEF Domain Containing G4	1.68300
POLR2C	RNA Polymerase II Subunit C	1.68254
FBP2	Fructose-Bisphosphatase 2	1.68226
GCNT2	Glucosaminyl (N-Acetyl) Transferase 2 (I Blood Group)	1.68159
SIGLEC8	Sialic Acid Binding Ig Like Lectin 8	1.68152
JPT1	Jupiter Microtubule Associated Homolog 1	1.68142
TRIM59	Tripartite Motif Containing 59	1.68131
LRRC41	Leucine Rich Repeat Containing 41	1.67928
FSIP1	Fibrous Sheath Interacting Protein 1	1.67906
MIR543	MicroRNA 543	1.67876
MAGI1-IT1	MAGI1 Intronic Transcript 1	1.67779
UTP20	UTP20 Small Subunit Processome Component	1.67720
FAF2	Fas Associated Factor Family Member 2	1.67640
ZBTB12	Zinc Finger And BTB Domain Containing 12	1.67636
LINC00479	Long Intergenic Non-Protein Coding RNA 479	1.67632
PHF14	PHD Finger Protein 14	1.67617
DNAJB11	DnaJ Heat Shock Protein Family (Hsp40) Member B11	1.67561
LOC11269475(	Uncharaterized LOC112694756	1.67555
MYO1G	Myosin IG	1.67513
CST5	Cystatin D	1.67491

FHOD1	Formin Homology 2 Domain Containing 1	1.67376
LTB4R2	Leukotriene B4 Receptor 2	1.67376
STOX1	Storkhead Box 1	1.67370
VPREB1	V-Set Pre-B Cell Surrogate Light Chain 1	1.67367
TM6SF2	Transmembrane 6 Superfamily Member 2	1.67358
FRMD8	FERM Domain Containing 8	1.67206
TMEM39B	Transmembrane Protein 39B	1.67206
TMEM233	Transmembrane Protein 233	1.67206
GSTA5	Glutathione S-Transferase Alpha 5	1.67202
PTPMT1	Protein Tyrosine Phosphatase Mitochondrial 1	1.67191
KANK2	KN Motif And Ankyrin Repeat Domains 2	1.67172
PITPNM1	Phosphatidylinositol Transfer Protein Membrane Associated 1	1.67171
CADM4	Cell Adhesion Molecule 4	1.67156
FOXJ2	Forkhead Box J2	1.67142
MIR526B	MicroRNA 526b	1.67054
TRAPPC3	Trafficking Protein Particle Complex Subunit 3	1.67036
MTF2	Metal Response Element Binding Transcription Factor 2	1.66986
COPS8	COP9 Signalosome Subunit 8	1.66955
STK40	Serine/Threonine Kinase 40	1.66933
FIBP	FGF1 Intracellular Binding Protein	1.66873
EXD2	Exonuclease 3'-5' Domain Containing 2	1.66846
MRTO4	MRT4 Homolog, Ribosome Maturation Factor	1.66830
BTF3	Basic Transcription Factor 3	1.66827
ARHGEF18	Rho/Rac Guanine Nucleotide Exchange Factor 18	1.66726
MED13	Mediator Complex Subunit 13	1.66711
TALAM1	TALAM1 Transcript, MALAT1 Antisense RNA	1.66697
ZNF512	Zinc Finger Protein 512	1.66691
MIR1179	MicroRNA 1179	1.66687
SLC2A5	Solute Carrier Family 2 Member 5	1.66656
HLA-H	Major Histocompatibility Complex, Class I, H (Pseudogene)	1.66645
HAS3	Hyaluronan Synthase 3	1.66577
SLC6A5	Solute Carrier Family 6 Member 5	1.66558
ATP5MJ	ATP Synthase Membrane Subunit J	1.66542
DOCK7	Dedicator Of Cytokinesis 7	1.66501
TMIGD2	Transmembrane And Immunoglobulin Domain Containing 2	1.66495

MAP3K4	Mitogen-Activated Protein Kinase Kinase Kinase 4	1.66495
NAXD	NAD(P)HX Dehydratase	1.66476
ARPIN	Actin Related Protein 2/3 Complex Inhibitor	1.66464
PSMC2	Proteasome 26S Subunit, ATPase 2	1.66445
PCNPP1	PEST Containing Nuclear Protein Pseudogene 1	1.66409
piR-38259		1.66409
piR-48007		1.66409
piR-50346		1.66409
piR-51449		1.66409
piR-56480-015		1.66409
HSALNG0094038		1.66409
piR-36455		1.66409
piR-51327		1.66409
MUCL3	Mucin Like 3	1.66403
IRX4	Iroquois Homeobox 4	1.66386
NPR3	Natriuretic Peptide Receptor 3	1.66361
SLC26A11	Solute Carrier Family 26 Member 11	1.66356
TACO1	Translational Activator Of Cytochrome C Oxidase I	1.66349
ZNF160	Zinc Finger Protein 160	1.66345
RAB3IL1	RAB3A Interacting Protein Like 1	1.66310
FAM241B	Family With Sequence Similarity 241 Member B	1.66306
NBPF18P	NBPF Member 18, Pseudogene	1.66304
SNX17	Sorting Nexin 17	1.66300
ZNF281	Zinc Finger Protein 281	1.66294
CNBD2	Cyclic Nucleotide Binding Domain Containing 2	1.66292
MZF1-AS1	MZF1 Antisense RNA 1	1.66292
ENSG00000259212		1.66292
ENSG00000225555		1.66292
ENSG00000236352		1.66292
ENSG00000219410		1.66292
piR-35524		1.66292
RF00017-1898		1.66292
ZBTB48	Zinc Finger And BTB Domain Containing 48	1.66240
GDF7	Growth Differentiation Factor 7	1.66203
CFAP57	Cilia And Flagella Associated Protein 57	1.66167
NUDT21	Nudix Hydrolase 21	1.66136
PRORP	Protein Only RNase P Catalytic Subunit	1.66131
ZNF446	Zinc Finger Protein 446	1.66128
ZFYVE19	Zinc Finger FYVE-Type Containing 19	1.66127
USP4	Ubiquitin Specific Peptidase 4	1.66092
SLC7A9	Solute Carrier Family 7 Member 9	1.66084
ARID3A	AT-Rich Interaction Domain 3A	1.66052

WDR3	WD Repeat Domain 3	1.66014
CYB561A3	Cytochrome B561 Family Member A3	1.66006
BMS1	BMS1 Ribosome Biogenesis Factor	1.65982
CLEC2L	C-Type Lectin Domain Family 2 Member L	1.65964
MFSD4B	Major Facilitator Superfamily Domain Containing 4B	1.65964
GJD3	Gap Junction Protein Delta 3	1.65961
ZNF780B	Zinc Finger Protein 780B	1.65961
NINL	Ninein Like	1.65920
MIR203B	MicroRNA 203b	1.65890
MIR9-1HG	MIR9-1 Host Gene	1.65890
CTNNA1-AS1	CTNNA1 Antisense RNA 1	1.65862
CHMP1B	Charged Multivesicular Body Protein 1B	1.65850
CEP20	Centrosomal Protein 20	1.65829
NUDCD3	NudC Domain Containing 3	1.65762
SLC25A10	Solute Carrier Family 25 Member 10	1.65752
SLC6A13	Solute Carrier Family 6 Member 13	1.65682
EIF4G2	Eukaryotic Translation Initiation Factor 4 Gamma 2	1.65678
NUFIP2	Nuclear FMR1 Interacting Protein 2	1.65662
METTL25B	Methyltransferase Like 25B	1.65658
ARL6IP4	ADP Ribosylation Factor Like GTPase 6 Interacting Protein 4	1.65653
QPCTL	Glutaminyl-Peptide Cyclotransferase Like	1.65562
NEURL1B	Neuralized E3 Ubiquitin Protein Ligase 1B	1.65558
GNL1	G Protein Nucleolar 1 (Putative)	1.65520
AUNIP	Aurora Kinase A And Ninein Interacting Protein	1.65465
IFRD1	Interferon Related Developmental Regulator 1	1.65430
CNTN2	Contactin 2	1.65428
ARPC3	Actin Related Protein 2/3 Complex Subunit 3	1.65406
EDC3	Enhancer Of MRNA Decapping 3	1.65397
CALB1	Calbindin 1	1.65364
WDTC1	WD And Tetratricopeptide Repeats 1	1.65355
THEMIS	Thymocyte Selection Associated	1.65268
TMEM214	Transmembrane Protein 214	1.65261
WDR24	WD Repeat Domain 24	1.65214
OSR1	Odd-Skipped Related Transcription Factor 1	1.65185
HGFAC	HGF Activator	1.65145
SERPINA4	Serpin Family A Member 4	1.65111
ZNF93	Zinc Finger Protein 93	1.65075
ZNF587	Zinc Finger Protein 587	1.65050

PROX2	Prospero Homeobox 2	1.65031
CARMN	Cardiac Mesoderm Enhancer-Associated Non-Coding RNA	1.64913
BPHL	Biphenyl Hydrolase Like	1.64903
DOCK9	Dedicator Of Cytokinesis 9	1.64895
MBOAT2	Membrane Bound O-Acyltransferase Domain Containing 2	1.64890
SNX32	Sorting Nexin 32	1.64863
FCAMR	Fc Fragment Of IgA And IgM Receptor	1.64811
DGLUCY	D-Glutamate Cyclase	1.64811
MTNR1A	Melatonin Receptor 1A	1.64810
ILF2	Interleukin Enhancer Binding Factor 2	1.64789
FAM177A1	Family With Sequence Similarity 177 Member A1	1.64741
DLX3	Distal-Less Homeobox 3	1.64659
LETM2	Leucine Zipper And EF-Hand Containing Transmembrane Protein 2	1.64653
NPBWR2	Neuropeptides B And W Receptor 2	1.64597
SQOR	Sulfide Quinone Oxidoreductase	1.64587
LYZ	Lysozyme	1.64575
BTBD2	BTB Domain Containing 2	1.64534
MIR8085	MicroRNA 8085	1.64522
KCTD5	Potassium Channel Tetramerization Domain Containing 5	1.64429
ZNF75A	Zinc Finger Protein 75a	1.64378
ASF1B	Anti-Silencing Function 1B Histone Chaperone	1.64369
JPT2	Jupiter Microtubule Associated Homolog 2	1.64313
PLCH2	Phospholipase C Eta 2	1.64301
ANKRD54	Ankyrin Repeat Domain 54	1.64280
TM4SF1-AS1	TM4SF1 Antisense RNA 1	1.64260
DAPK1-IT1	DAPK1 Intronic Transcript 1	1.64260
SCAMP4	Secretory Carrier Membrane Protein 4	1.64259
MRS2	Magnesium Transporter MRS2	1.64228
ATP13A1	ATPase 13A1	1.64215
SELENOH	Selenoprotein H	1.64072
ATAT1	Alpha Tubulin Acetyltransferase 1	1.64036
CFAP20	Cilia And Flagella Associated Protein 20	1.64003
TRNT1	TRNA Nucleotidyl Transferase 1	1.63983
GJA4	Gap Junction Protein Alpha 4	1.63885
IRAG1	Inositol 1,4,5-Triphosphate Receptor Associated 1	1.63811
ADAMTS12	ADAM Metallopeptidase With Thrombospondin Type 1 Motif 12	1.63746
SLC35F5	Solute Carrier Family 35 Member F5	1.63709
C6orf136	Chromosome 6 Open Reading Frame 136	1.63692
TIPIN	TIMELESS Interacting Protein	1.63611

GPR89A	G Protein-Coupled Receptor 89A	1.63608
GOLGA2P10	GOLGA2 Pseudogene 10	1.63606
TRPC3	Transient Receptor Potential Cation Channel Subfamily C Member 3	1.63589
ZNF302	Zinc Finger Protein 302	1.63531
STUM	Stum, Mechanosensory Transduction Mediator Homolog	1.63531
VPS25	Vacuolar Protein Sorting 25 Homolog	1.63489
ENSG00000183308		1.63484
CLP1	Cleavage Factor Polyribonucleotide Kinase Subunit 1	1.63447
GEMIN7	Gem Nuclear Organelle Associated Protein 7	1.63447
CDK6-AS1	CDK6 Antisense RNA 1	1.63434
KLHL18	Kelch Like Family Member 18	1.63414
ANKRD10	Ankyrin Repeat Domain 10	1.63414
DLGAP4	DLG Associated Protein 4	1.63401
RPUSD1	RNA Pseudouridine Synthase Domain Containing 1	1.63397
DPYSL3	Dihydropyrimidinase Like 3	1.63210
RGL1	Ral Guanine Nucleotide Dissociation Stimulator Like 1	1.63161
LST1	Leukocyte Specific Transcript 1	1.63068
KCNK2	Potassium Two Pore Domain Channel Subfamily K Member 2	1.63026
FCRL3	Fc Receptor Like 3	1.63004
STRN	Striatin	1.62982
YPEL4	Yippee Like 4	1.62945
RBM34	RNA Binding Motif Protein 34	1.62945
ZBTB40	Zinc Finger And BTB Domain Containing 40	1.62930
ADGRG3	Adhesion G Protein-Coupled Receptor G3	1.62868
SMOC1	SPARC Related Modular Calcium Binding 1	1.62860
ZSCAN21	Zinc Finger And SCAN Domain Containing 21	1.62846
DEGS1	Delta 4-Desaturase, Sphingolipid 1	1.62844
PPP1R37	Protein Phosphatase 1 Regulatory Subunit 37	1.62833
SLC35B2	Solute Carrier Family 35 Member B2	1.62793
LRFN4	Leucine Rich Repeat And Fibronectin Type III Domain Containing 4	1.62783
QDPR	Quinoid Dihydropteridine Reductase	1.62733
ZNF410	Zinc Finger Protein 410	1.62728
P2RY6	Pyrimidinergic Receptor P2Y6	1.62710
TMEM181	Transmembrane Protein 181	1.62708
EBI3	Epstein-Barr Virus Induced 3	1.62706

ZBTB8OS	Zinc Finger And BTB Domain Containing 8 Opposite Strand	1.62630
YRDC	YrdC N6-Threonylcarbamoyltransferase Domain Containing	1.62551
MIR1228	MicroRNA 1228	1.62494
CD300A	CD300a Molecule	1.62450
HIVEP3	HIVEP Zinc Finger 3	1.62435
EMC8	ER Membrane Protein Complex Subunit 8	1.62434
ZBTB7C	Zinc Finger And BTB Domain Containing 7C	1.62434
FLG2	Filaggrin Family Member 2	1.62385
BSCL2	BSCL2 Lipid Droplet Biogenesis Associated, Seipin	1.62303
ADAMTS5	ADAM Metallopeptidase With Thrombospondin Type 1 Motif 5	1.62296
GEMIN5	Gem Nuclear Organelle Associated Protein 5	1.62293
EID2	EP300 Interacting Inhibitor Of Differentiation 2	1.62283
DRC3	Dynein Regulatory Complex Subunit 3	1.62283
CASP8AP2	Caspase 8 Associated Protein 2	1.62240
F2RL2	Coagulation Factor II Thrombin Receptor Like 2	1.62196
SLC6A18	Solute Carrier Family 6 Member 18	1.62173
MIR1247	MicroRNA 1247	1.62168
LINC00663	Long Intergenic Non-Protein Coding RNA 663	1.62148
VPS35	VPS35 Retromer Complex Component	1.62136
DBI	Diazepam Binding Inhibitor, Acyl-CoA Binding Protein	1.62128
MIR448	MicroRNA 448	1.62079
TOR4A	Torsin Family 4 Member A	1.62023
LRRN2	Leucine Rich Repeat Neuronal 2	1.61978
ESRP2	Epithelial Splicing Regulatory Protein 2	1.61948
GPR162	G Protein-Coupled Receptor 162	1.61881
HES2	Hes Family BHLH Transcription Factor 2	1.61866
NEUROG3	Neurogenin 3	1.61800
MRPL12	Mitochondrial Ribosomal Protein L12	1.61766
RGS16	Regulator Of G Protein Signaling 16	1.61764
PSORS1C2	Psoriasis Susceptibility 1 Candidate 2	1.61710
ATP8B3	ATPase Phospholipid Transporting 8B3	1.61691
ELOVL1	ELOVL Fatty Acid Elongase 1	1.61673
DLGAP1-AS1	DLGAP1 Antisense RNA 1	1.61632
BICD2	BICD Cargo Adaptor 2	1.61605
MOSPD3	Motile Sperm Domain Containing 3	1.61582
PMS2P9	PMS1 Homolog 2, Mismatch Repair System Component Pseudogene 9	1.61581

FOXB1	Forkhead Box B1	1.61484
MEPCE	Methylphosphate Capping Enzyme	1.61435
PRXL2A	Peroxiredoxin Like 2A	1.61377
TM4SF18	Transmembrane 4 L Six Family Member 18	1.61372
HMG20A	High Mobility Group 20A	1.61322
FTSJ3	FtsJ RNA 2'-O-Methyltransferase 3	1.61322
ELOA	Elongin A	1.61320
FAM180B	Family With Sequence Similarity 180 Member B	1.61289
MK279980		1.61250
piR-48820-008		1.61250
ANKDD1A	Ankyrin Repeat And Death Domain Containing 1A	1.61201
NMD3	NMD3 Ribosome Export Adaptor	1.61182
GABBR1	Gamma-Aminobutyric Acid Type B Receptor Subunit 1	1.61147
PRR14	Proline Rich 14	1.61110
PET100	PET100 Cytochrome C Oxidase Chaperone	1.61108
MLEC	Malectin	1.61023
LAX1	Lymphocyte Transmembrane Adaptor 1	1.60982
TMEM63C	Transmembrane Protein 63C	1.60978
CEP104	Centrosomal Protein 104	1.60976
PMFBP1	Polyamine Modulated Factor 1 Binding Protein 1	1.60952
ZNF571	Zinc Finger Protein 571	1.60952
C16orf95-DT	C16orf95 Divergent Transcript	1.60952
ENSG00000267275		1.60952
ENSG00000228559		1.60952
MAPK8IP1P1	Mitogen-Activated Protein Kinase 8 Interacting Protein 1 Pseudogene 1	1.60952
LOC10536951[illegible]	Uncharacterized LOC105369519	1.60952
ENSG00000262133		1.60952
ENSG00000267114		1.60952
ENSG00000250240		1.60952
RNU4-36P	RNA, U4 Small Nuclear 36, Pseudogene	1.60952
ENSG00000199332		1.60952
ENSG00000226963		1.60952
TREHP1	Trehalase Pseudogene 1	1.60952
LOC10192887[illegible]	Uncharacterized LOC101928875	1.60952
piR-33614-032		1.60952
ACBD3-AS1	ACBD3 Antisense RNA 1	1.60933
MRGPRF	MAS Related GPR Family Member F	1.60857
COPG1	COPI Coat Complex Subunit Gamma 1	1.60855
PTPN4	Protein Tyrosine Phosphatase Non-Receptor Type 4	1.60833
DIRAS1	DIRAS Family GTPase 1	1.60815
VAMP5	Vesicle Associated Membrane Protein 5	1.60802

MIR596	MicroRNA 596	1.60796
TRIT1	TRNA Isopentenyltransferase 1	1.60784
SOX12	SRY-Box Transcription Factor 12	1.60737
ZNF112	Zinc Finger Protein 112	1.60715
ENGASE	Endo-Beta-N-Acetylglucosaminidase	1.60707
TUBB4B	Tubulin Beta 4B Class IVb	1.60697
TMX2-CTNND	TMX2-CTNND1 Readthrough (NMD Candidate)	1.60677
ADAMTSL2	ADAMTS Like 2	1.60672
KIF3C	Kinesin Family Member 3C	1.60639
PDE3B	Phosphodiesterase 3B	1.60637
CLN6	CLN6 Transmembrane ER Protein	1.60623
BBLN	Bublin Coiled Coil Protein	1.60601
TSPO2	Translocator Protein 2	1.60586
TFCP2L1	Transcription Factor CP2 Like 1	1.60520
AVPR1A	Arginine Vasopressin Receptor 1A	1.60500
KDM3B	Lysine Demethylase 3B	1.60491
MRPL9	Mitochondrial Ribosomal Protein L9	1.60490
MPDU1	Mannose-P-Dolichol Utilization Defect 1	1.60484
SLC31A2	Solute Carrier Family 31 Member 2	1.60472
PEBP4	Phosphatidylethanolamine Binding Protein 4	1.60469
AEN	Apoptosis Enhancing Nuclease	1.60452
VWCE	Von Willebrand Factor C And EGF Domains	1.60432
TWSG1	Twisted Gastrulation BMP Signaling Modulator 1	1.60423
MEA1	Male-Enhanced Antigen 1	1.60395
ZDHHC8	Zinc Finger DHHC-Type Palmitoyltransferase 8	1.60386
CDV3	CDV3 Homolog	1.60342
NOL3	Nucleolar Protein 3	1.60313
BRD1	Bromodomain Containing 1	1.60291
MMP25	Matrix Metallopeptidase 25	1.60220
HINT1	Histidine Triad Nucleotide Binding Protein 1	1.60204
HSALNG0102089		1.60196
H2AC4	H2A Clustered Histone 4	1.60173
ZNF76	Zinc Finger Protein 76	1.60138
NOL4L	Nucleolar Protein 4 Like	1.60122
TAF1B	TATA-Box Binding Protein Associated Factor, RNA Polymerase I Subunit B	1.60064
MIR105-2	MicroRNA 105-2	1.60052
ATP6V1G2	ATPase H+ Transporting V1 Subunit G2	1.60041
ACSS3	Acyl-CoA Synthetase Short Chain Family Member 3	1.59970
LNX2	Ligand Of Numb-Protein X 2	1.59945

C2CD3	C2 Domain Containing 3 Centriole Elongation Regulator	1.59912
PLXNB3	Plexin B3	1.59890
BAHD1	Bromo Adjacent Homology Domain Containing 1	1.59862
LYPD5	LY6/PLAUR Domain Containing 5	1.59824
ZMIZ2	Zinc Finger MIZ-Type Containing 2	1.59810
CKMT1B	Creatine Kinase, Mitochondrial 1B	1.59804
NSUN6	NOP2/Sun RNA Methyltransferase 6	1.59741
CREBZF	CREB/ATF BZIP Transcription Factor	1.59670
SMIM10L2A	Small Integral Membrane Protein 10 Like 2A	1.59669
RPP40	Ribonuclease P/MRP Subunit P40	1.59602
AFMID	Arylformamidase	1.59572
FAM234A	Family With Sequence Similarity 234 Member A	1.59490
TOM1	Target Of Myb1 Membrane Trafficking Protein	1.59482
SAT2	Spermidine/Spermine N1-Acetyltransferase Family Member 2	1.59477
LOC338694	Uncharacterized LOC338694	1.59460
ZNF414	Zinc Finger Protein 414	1.59332
GPALPP1	GPALPP Motifs Containing 1	1.59332
HEMK1	HemK Methyltransferase Family Member 1	1.59322
FAM78A	Family With Sequence Similarity 78 Member A	1.59322
FAM78B	Family With Sequence Similarity 78 Member B	1.59322
PKD1L1-AS1	PKD1L1 Antisense RNA 1	1.59322
JMJD8	Jumonji Domain Containing 8	1.59319
B3GAT3	Beta-1,3-Glucuronyltransferase 3	1.59313
QRSL1	Glutaminyl-TRNA Amidotransferase Subunit QRSL1	1.59307
MBOAT4	Membrane Bound O-Acyltransferase Domain Containing 4	1.59283
PHLDB3	Pleckstrin Homology Like Domain Family B Member 3	1.59220
RANGAP1	Ran GTPase Activating Protein 1	1.59208
LINC01354	Long Intergenic Non-Protein Coding RNA 1354	1.59155
PSMD3	Proteasome 26S Subunit, Non-ATPase 3	1.59114
P4HA1	Prolyl 4-Hydroxylase Subunit Alpha 1	1.59090
IRX3	Iroquois Homeobox 3	1.59087
MIR551B	MicroRNA 551b	1.59007
PKD1L2	Polycystin 1 Like 2 (Gene/Pseudogene)	1.58912
ARHGEF25	Rho Guanine Nucleotide Exchange Factor 25	1.58898

NDUFAF1	NADH:Ubiquinone Oxidoreductase Complex Assembly Factor 1	1.58768
TKFC	Triokinase And FMN Cyclase	1.58765
HOXC8	Homeobox C8	1.58739
CSDC2	Cold Shock Domain Containing C2	1.58732
FGD5-AS1	FGD5 Antisense RNA 1	1.58704
B4GALT1-AS1	B4GALT1 Antisense RNA 1	1.58546
CHSY1	Chondroitin Sulfate Synthase 1	1.58500
LOC10013309	Uncharacterized LOC100133091	1.58472
FUT7	Fucosyltransferase 7	1.58464
ITPKA	Inositol-Trisphosphate 3-Kinase A	1.58417
VTI1B	Vesicle Transport Through Interaction With T-SNAREs 1B	1.58339
MARCHF2	Membrane Associated Ring-CH-Type Finger 2	1.58269
ZBTB21	Zinc Finger And BTB Domain Containing 21	1.58228
TMEM184A	Transmembrane Protein 184A	1.58228
CHMP5	Charged Multivesicular Body Protein 5	1.58221
TWF2	Twinfilin Actin Binding Protein 2	1.58221
ERBIN	Erbb2 Interacting Protein	1.58198
POPDC3	Popeye Domain Containing 3	1.58064
PARPBP	PARP1 Binding Protein	1.58001
C2orf68	Chromosome 2 Open Reading Frame 68	1.57995
IL25	Interleukin 25	1.57995
MCM3AP-AS1	MCM3AP Antisense RNA 1	1.57975
ALS2	Alsin Rho Guanine Nucleotide Exchange Factor ALS2	1.57942
NLGN2	Neuroligin 2	1.57906
LDLR-AS1	LDLR Antisense RNA 1	1.57891
LSM10	LSM10, U7 Small Nuclear RNA Associated	1.57875
FBLN2	Fibulin 2	1.57853
NCKIPSD	NCK Interacting Protein With SH3 Domain	1.57841
YPEL3	Yippee Like 3	1.57792
PLA2R1	Phospholipase A2 Receptor 1	1.57791
ANKRD9	Ankyrin Repeat Domain 9	1.57756
RBKS	Ribokinase	1.57684
CLN8-AS1	CLN8 Antisense RNA 1	1.57684
HDHD3	Haloacid Dehalogenase Like Hydrolase Domain Containing 3	1.57654
TPBGL	Trophoblast Glycoprotein Like	1.57654
FOXO3B	Forkhead Box O3B	1.57654
CROCCP3	CROCC Pseudogene 3	1.57654
SCRN1	Secernin 1	1.57599
ZNF436	Zinc Finger Protein 436	1.57564
SEPTIN12	Septin 12	1.57564
TMEM54	Transmembrane Protein 54	1.57516

CEP350	Centrosomal Protein 350	1.57513
GABRA6	Gamma-Aminobutyric Acid Type A Receptor Subunit Alpha6	1.57504
OPTC	Opticin	1.57471
ZNF439	Zinc Finger Protein 439	1.57452
TPTEP2-CSNK	TPTEP2-CSNK1E Readthrough	1.57426
NECTIN1	Nectin Cell Adhesion Molecule 1	1.57421
SCUBE1	Signal Peptide, CUB Domain And EGF Like Domain Containing 1	1.57354
GRIP2	Glutamate Receptor Interacting Protein 2	1.57351
TSACC	TSSK6 Activating Cochaperone	1.57351
SPATA32	Spermatogenesis Associated 32	1.57351
DOCK5	Dedicator Of Cytokinesis 5	1.57335
ZNF443	Zinc Finger Protein 443	1.57279
SEPTIN8	Septin 8	1.57256
SRSF7	Serine And Arginine Rich Splicing Factor 7	1.57250
OGFOD3	2-Oxoglutarate And Iron Dependent Oxygenase Domain Containing 3	1.57175
PSORS1C3	Psoriasis Susceptibility 1 Candidate 3	1.57175
SPSB3	SplA/Ryanodine Receptor Domain And SOCS Box Containing 3	1.57118
MIR1303	MicroRNA 1303	1.57091
ZNF417	Zinc Finger Protein 417	1.57079
RCCD1	RCC1 Domain Containing 1	1.57056
TIMM13	Translocase Of Inner Mitochondrial Membrane 13	1.57052
IRF2BP1	Interferon Regulatory Factor 2 Binding Protein 1	1.56974
GPATCH4	G-Patch Domain Containing 4	1.56892
MIR7108	MicroRNA 7108	1.56889
SLC27A4	Solute Carrier Family 27 Member 4	1.56802
INTS14	Integrator Complex Subunit 14	1.56773
OSGIN1	Oxidative Stress Induced Growth Inhibitor 1	1.56763
SLC7A10	Solute Carrier Family 7 Member 10	1.56711
GCAT	Glycine C-Acetyltransferase	1.56703
FBRS	Fibrosin	1.56703
ALDH4A1	Aldehyde Dehydrogenase 4 Family Member A1	1.56682
INTS2	Integrator Complex Subunit 2	1.56677
CLEC1B	C-Type Lectin Domain Family 1 Member B	1.56676
FFAR1	Free Fatty Acid Receptor 1	1.56497
RAB11FIP2	RAB11 Family Interacting Protein 2	1.56490
EIF1	Eukaryotic Translation Initiation Factor 1	1.56485

SDR39U1	Short Chain Dehydrogenase/Reductase Family 39U Member 1	1.56470
MBOAT1	Membrane Bound O-Acyltransferase Domain Containing 1	1.56443
ALDH7A1	Aldehyde Dehydrogenase 7 Family Member A1	1.56416
TMEM94	Transmembrane Protein 94	1.56403
REPS1	RALBP1 Associated Eps Domain Containing 1	1.56402
MRPL38	Mitochondrial Ribosomal Protein L38	1.56401
SUPT7L	SPT7 Like, STAGA Complex Subunit Gamma	1.56366
PPRC1	PPARG Related Coactivator 1	1.56256
FAM219B	Family With Sequence Similarity 219 Member B	1.56256
PSMD2	Proteasome 26S Subunit Ubiquitin Receptor, Non-ATPase 2	1.56129
ZC3H12A	Zinc Finger CCCH-Type Containing 12A	1.56042
MRM3	Mitochondrial RRNA Methyltransferase 3	1.56005
CENPM	Centromere Protein M	1.55979
SMARCAD1	SWI/SNF-Related, Matrix-Associated Actin-Dependent Regulator Of Chromatin, Subfamily A, Containing DEAD/H Box 1	1.55938
GPR55	G Protein-Coupled Receptor 55	1.55931
LINC00970	Long Intergenic Non-Protein Coding RNA 970	1.55761
AQP5-AS1	AQP5 And AQP2 Antisense RNA 2	1.55725
SLC27A1	Solute Carrier Family 27 Member 1	1.55712
GLG1	Golgi Glycoprotein 1	1.55650
CPM	Carboxypeptidase M	1.55622
TLDC2	TBC/LysM-Associated Domain Containing 2	1.55606
LRMDA	Leucine Rich Melanocyte Differentiation Associated	1.55555
CPO	Carboxypeptidase O	1.55500
TRPC4AP	Transient Receptor Potential Cation Channel Subfamily C Member 4 Associated Protein	1.55468
HCG20	HLA Complex Group 20	1.55363
HCG25	HLA Complex Group 25	1.55363
MRTFA-AS1	MRTFA Antisense RNA 1	1.55363
ENSG00000250751		1.55363
ENSG00000272501		1.55363
ENSG00000266903		1.55363
ENSG00000226438		1.55363
ENSG00000269148		1.55363

lnc-PXYLP1-3		1.55363
lnc-TFDP2-12		1.55363
piR-59591		1.55363
lnc-TMEM238L-5		1.55363
RF00017-1978		1.55363
AGO4	Argonaute RISC Component 4	1.55362
SUDS3	SDS3 Homolog, SIN3A Corepressor Complex Component	1.55317
BEST2	Bestrophin 2	1.55224
GMDS	GDP-Mannose 4,6-Dehydratase	1.55167
TMEM9B	TMEM9 Domain Family Member B	1.55164
JADE2	Jade Family PHD Finger 2	1.54985
SRRM1P1	Serine/Arginine Repetitive Matrix 1 Pseudogene 1	1.54934
ZNF839	Zinc Finger Protein 839	1.54897
INTS9	Integrator Complex Subunit 9	1.54838
MIPOL1	Mirror-Image Polydactyly 1	1.54807
LINC01128	Long Intergenic Non-Protein Coding RNA 1128	1.54804
CCL23	C-C Motif Chemokine Ligand 23	1.54796
BATF	Basic Leucine Zipper ATF-Like Transcription Factor	1.54779
PDE9A	Phosphodiesterase 9A	1.54769
ARL4D	ADP Ribosylation Factor Like GTPase 4D	1.54754
DDX19B	DEAD-Box Helicase 19B	1.54739
ZNF678	Zinc Finger Protein 678	1.54703
TAF5	TATA-Box Binding Protein Associated Factor 5	1.54605
DGAT2	Diacylglycerol O-Acyltransferase 2	1.54578
MRPL20-DT	MRPL20 Divergent Transcript	1.54527
FBXL5	F-Box And Leucine Rich Repeat Protein 5	1.54356
LOC10050669	Uncharacterized LOC100506691	1.54301
VPS53	VPS53 Subunit Of GARP Complex	1.54242
RANGRF	RAN Guanine Nucleotide Release Factor	1.54238
MED9	Mediator Complex Subunit 9	1.54227
FAM104A	Family With Sequence Similarity 104 Member A	1.54210
PPAT	Phosphoribosyl Pyrophosphate Amidotransferase	1.54208
GIGYF1	GRB10 Interacting GYF Protein 1	1.54195
SLC25A35	Solute Carrier Family 25 Member 35	1.54157
KHNYN	KH And NYN Domain Containing	1.54157
JPH3	Junctophilin 3	1.54136
NUB1	Negative Regulator Of Ubiquitin Like Proteins 1	1.54061
ZNF404	Zinc Finger Protein 404	1.54038
NECAP2	NECAP Endocytosis Associated 2	1.54034

SYNJ1	Synaptojanin 1	1.54031
TRMT61B	TRNA Methyltransferase 61B	1.53966
NAGPA	N-Acetylglucosamine-1-Phosphodiester Alpha-N-Acetylglucosaminidase	1.53910
USP20	Ubiquitin Specific Peptidase 20	1.53893
PRRT1	Proline Rich Transmembrane Protein 1	1.53889
CNN1	Calponin 1	1.53831
SNRNP48	Small Nuclear Ribonucleoprotein U11/U12 Subunit 48	1.53804
PKHD1L1	PKHD1 Like 1	1.53804
ATP5IF1	ATP Synthase Inhibitory Factor Subunit 1	1.53787
HS3ST2	Heparan Sulfate-Glucosamine 3-Sulfotransferase 2	1.53787
TRIM62	Tripartite Motif Containing 62	1.53701
ZNF174	Zinc Finger Protein 174	1.53680
SFXN3	Sideroflexin 3	1.53623
APOC2	Apolipoprotein C2	1.53608
ARHGEF17	Rho Guanine Nucleotide Exchange Factor 17	1.53594
FAM87B	Family With Sequence Similarity 87 Member B	1.53582
LINC01786	Long Intergenic Non-Protein Coding RNA 1786	1.53582
ENSG00000258634		1.53582
HSALNG0091217		1.53582
HSALNG0091218		1.53582
KCNJ6	Potassium Inwardly Rectifying Channel Subfamily J Member 6	1.53570
ZSWIM6	Zinc Finger SWIM-Type Containing 6	1.53554
PRB2	Proline Rich Protein BstNI Subfamily 2	1.53544
ZNF233	Zinc Finger Protein 233	1.53512
SDE2	SDE2 Telomere Maintenance Homolog	1.53465
ADK	Adenosine Kinase	1.53417
INCA1	Inhibitor Of CDK, Cyclin A1 Interacting Protein 1	1.53379
HDGFL2	HDGF Like 2	1.53368
TC2N	Tandem C2 Domains, Nuclear	1.53350
NPIPB13	Nuclear Pore Complex Interacting Protein Family, Member B13	1.53326
COL5A1-AS1	COL5A1 Antisense RNA 1	1.53326
BAK1P1	BCL2 Antagonist/Killer 1 Pseudogene 1	1.53326
MRPS25	Mitochondrial Ribosomal Protein S25	1.53306
NIF3L1	NGG1 Interacting Factor 3 Like 1	1.53289
LY6G6D	Lymphocyte Antigen 6 Family Member G6D	1.53278
SLC25A15	Solute Carrier Family 25 Member 15	1.53248
ZBTB1	Zinc Finger And BTB Domain Containing 1	1.53179

NUCB1	Nucleobindin 1	1.53116
MIR665	MicroRNA 665	1.53100
YIPF2	Yip1 Domain Family Member 2	1.53021
ENSG00000267640		1.52928
GPR142	G Protein-Coupled Receptor 142	1.52894
POMZP3	POM121 And ZP3 Fusion	1.52894
LSMEM2	Leucine Rich Single-Pass Membrane Protein 2	1.52894
MIR579	MicroRNA 579	1.52894
MIR718	MicroRNA 718	1.52818
LAS1L	LAS1 Like Ribosome Biogenesis Factor	1.52812
CENPT	Centromere Protein T	1.52799
PAICS	Phosphoribosylaminoimidazole Carboxylase And Phosphoribosylaminoimidazolesuccinocarboxamide Synthase	1.52780
MYL6B	Myosin Light Chain 6B	1.52757
IL36RN	Interleukin 36 Receptor Antagonist	1.52741
MDS2	Myelodysplastic Syndrome 2 Translocation Associated	1.52734
ERFE	Erythroferrone	1.52725
MRPL24	Mitochondrial Ribosomal Protein L24	1.52723
TEF	TEF Transcription Factor, PAR BZIP Family Member	1.52721
FBF1	Fas Binding Factor 1	1.52721
SNX1	Sorting Nexin 1	1.52696
UCKL1	Uridine-Cytidine Kinase 1 Like 1	1.52670
EXOSC1	Exosome Component 1	1.52668
ZNF552	Zinc Finger Protein 552	1.52639
SFR1	SWI5 Dependent Homologous Recombination Repair Protein 1	1.52607
NRGN	Neurogranin	1.52592
PPP1R14B	Protein Phosphatase 1 Regulatory Inhibitor Subunit 14B	1.52508
MMP24	Matrix Metallopeptidase 24	1.52425
FBXO34	F-Box Protein 34	1.52400
ZNF550	Zinc Finger Protein 550	1.52400
ZNF776	Zinc Finger Protein 776	1.52400
CARD16	Caspase Recruitment Domain Family Member 16	1.52361
SLC25A26	Solute Carrier Family 25 Member 26	1.52309
MGST3	Microsomal Glutathione S-Transferase 3	1.52307
ARHGAP17	Rho GTPase Activating Protein 17	1.52303
C1GALT1	Core 1 Synthase, Glycoprotein-N-Acetylgalactosamine 3-Beta-Galactosyltransferase 1	1.52295
ZDHHC5	Zinc Finger DHHC-Type Palmitoyltransferase 5	1.52294
FRG1	FSHD Region Gene 1	1.52246

LOC10936367(	BMP2 5' Regulatory Region	1.52244
RHOF	Ras Homolog Family Member F, Filopodia Associated	1.52193
RRN3	RRN3 Homolog, RNA Polymerase I Transcription Factor	1.52173
FAM83F	Family With Sequence Similarity 83 Member F	1.52119
DNMBP	Dynamin Binding Protein	1.52096
ASGR2	Asialoglycoprotein Receptor 2	1.52078
MYADM	Myeloid Associated Differentiation Marker	1.52037
CCL15	C-C Motif Chemokine Ligand 15	1.52009
OTUD7B	OTU Deubiquitinase 7B	1.51986
ADCK5	AarF Domain Containing Kinase 5	1.51975
APBA1	Amyloid Beta Precursor Protein Binding Family A Member 1	1.51957
C11orf68	Chromosome 11 Open Reading Frame 68	1.51953
HAUS1	HAUS Augmin Like Complex Subunit 1	1.51926
SOSTDC1	Sclerostin Domain Containing 1	1.51926
ADGRA2	Adhesion G Protein-Coupled Receptor A2	1.51904
BICDL1	BICD Family Like Cargo Adaptor 1	1.51880
KIR3DL2	Killer Cell Immunoglobulin Like Receptor, Three Ig Domains And Long Cytoplasmic Tail 2	1.51867
TNP1	Transition Protein 1	1.51841
ZNF70	Zinc Finger Protein 70	1.51815
FMNL2	Formin Like 2	1.51773
PDZK1	PDZ Domain Containing 1	1.51742
DTX3	Deltex E3 Ubiquitin Ligase 3	1.51739
GPR34	G Protein-Coupled Receptor 34	1.51732
ATAD1	ATPase Family AAA Domain Containing 1	1.51723
MIR410	MicroRNA 410	1.51700
LHX4	LIM Homeobox 4	1.51603
SSBP1	Single Stranded DNA Binding Protein 1	1.51587
ADH4	Alcohol Dehydrogenase 4 (Class II), Pi Polypeptide	1.51518
SYT8	Synaptotagmin 8	1.51516
GJA8	Gap Junction Protein Alpha 8	1.51486
WDR55	WD Repeat Domain 55	1.51444
TXNRD3	Thioredoxin Reductase 3	1.51421
UTP11	UTP11 Small Subunit Processome Component	1.51387
KIF22	Kinesin Family Member 22	1.51340
GDE1	Glycerophosphodiester Phosphodiesterase 1	1.51336

ITPRIP	Inositol 1,4,5-Trisphosphate Receptor Interacting Protein	1.51321
ZSCAN22	Zinc Finger And SCAN Domain Containing 22	1.51321
CHRM2	Cholinergic Receptor Muscarinic 2	1.51307
S100A16	S100 Calcium Binding Protein A16	1.51301
MIR762HG	MIR762 Host Gene	1.51224
PHYHIPL	Phytanoyl-CoA 2-Hydroxylase Interacting Protein Like	1.51219
ACYP1	Acylphosphatase 1	1.51200
WAPL	WAPL Cohesin Release Factor	1.51173
GMFG	Glia Maturation Factor Gamma	1.51156
LINC00324	Long Intergenic Non-Protein Coding RNA 324	1.51139
ZZEF1	Zinc Finger ZZ-Type And EF-Hand Domain Containing 1	1.51114
CAMLG	Calcium Modulating Ligand	1.51103
PLEKHM3	Pleckstrin Homology Domain Containing M3	1.51086
LAGE3	L Antigen Family Member 3	1.51029
GGA2	Golgi Associated, Gamma Adaptin Ear Containing, ARF Binding Protein 2	1.51009
RCC1L	RCC1 Like	1.51004
AAMDC	Adipogenesis Associated Mth938 Domain Containing	1.51002
FKBPL	FKBP Prolyl Isomerase Like	1.50989
KICS2	KICSTOR Subunit 2	1.50968
MUSTN1	Musculoskeletal, Embryonic Nuclear Protein 1	1.50968
IARS1	Isoleucyl-TRNA Synthetase 1	1.50942
SKOR1	SKI Family Transcriptional Corepressor 1	1.50913
CENPB	Centromere Protein B	1.50913
BNIP1	BCL2 Interacting Protein 1	1.50912
PSMC1	Proteasome 26S Subunit, ATPase 1	1.50893
YIPF5	Yip1 Domain Family Member 5	1.50888
ATP6V1G1	ATPase H+ Transporting V1 Subunit G1	1.50887
MEIS3	Meis Homeobox 3	1.50868
RAMP3	Receptor Activity Modifying Protein 3	1.50866
SOX14	SRY-Box Transcription Factor 14	1.50840
C4BPB	Complement Component 4 Binding Protein Beta	1.50816
DCUN1D2	Defective In Cullin Neddylation 1 Domain Containing 2	1.50788
UFL1	UFM1 Specific Ligase 1	1.50776
H2AW	H2A.W Histone	1.50753
CC2D2B	Coiled-Coil And C2 Domain Containing 2B	1.50743

MCMBP	Minichromosome Maintenance Complex Binding Protein	1.50707
CLDN17	Claudin 17	1.50686
UBE2Z	Ubiquitin Conjugating Enzyme E2 Z	1.50605
KLF7	Kruppel Like Factor 7	1.50576
ZNF627	Zinc Finger Protein 627	1.50535
C1orf147	Chromosome 1 Open Reading Frame 147	1.50535
RAMP2-AS1	RAMP2 Antisense RNA 1	1.50502
MAST3	Microtubule Associated Serine/Threonine Kinase 3	1.50440
GBGT1	Globoside Alpha-1,3-N-Acetylgalactosaminyltransferase 1 (FORS Blood Group)	1.50395
CCDC69	Coiled-Coil Domain Containing 69	1.50395
GCSAM	Germinal Center Associated Signaling And Motility	1.50364
SLC8A3	Solute Carrier Family 8 Member A3	1.50363
MYO1A	Myosin IA	1.50348
KRT80	Keratin 80	1.50292
TMEM255B	Transmembrane Protein 255B	1.50292
ZNF677	Zinc Finger Protein 677	1.50275
CLSTN1	Calsyntenin 1	1.50250
SNORD10	Small Nucleolar RNA, C/D Box 10	1.50247
MRPS18A	Mitochondrial Ribosomal Protein S18A	1.50238
THBS3-AS1	THBS3 Antisense RNA 1	1.50231
PTPRZ1	Protein Tyrosine Phosphatase Receptor Type Z1	1.50225
WDR87	WD Repeat Domain 87	1.50214
HSP90AA2P	Heat Shock Protein 90 Alpha Family Class A Member 2, Pseudogene	1.50044
VCPKMT	Valosin Containing Protein Lysine Methyltransferase	1.50042
ZFP62	ZFP62 Zinc Finger Protein	1.50042
RTN2	Reticulon 2	1.50032
ZFP1	ZFP1 Zinc Finger Protein	1.49981
USP49	Ubiquitin Specific Peptidase 49	1.49892
AGO3	Argonaute RISC Catalytic Component 3	1.49892
DCDC2B	Doublecortin Domain Containing 2B	1.49865
ARL2	ADP Ribosylation Factor Like GTPase 2	1.49834
NDUFB3	NADH:Ubiquinone Oxidoreductase Subunit B3	1.49802
TRMT10C	TRNA Methyltransferase 10C, Mitochondrial RNase P Subunit	1.49777
GABPA	GA Binding Protein Transcription Factor Subunit Alpha	1.49775
KCTD17	Potassium Channel Tetramerization Domain Containing 17	1.49767
SAPCD1	Suppressor APC Domain Containing 1	1.49738

NAGS	N-Acetylglutamate Synthase	1.49706
DUSP7	Dual Specificity Phosphatase 7	1.49690
IQCN	IQ Motif Containing N	1.49681
GDPD3	Glycerophosphodiester Phosphodiesterase Domain Containing 3	1.49660
SRCIN1	SRC Kinase Signaling Inhibitor 1	1.49647
LAT2	Linker For Activation Of T Cells Family Member 2	1.49622
IFI30	IFI30 Lysosomal Thiol Reductase	1.49615
KRT34	Keratin 34	1.49601
MIEF1	Mitochondrial Elongation Factor 1	1.49601
SUGP2	SURP And G-Patch Domain Containing 2	1.49516
TAOK1	TAO Kinase 1	1.49507
IRGC	Immunity Related GTPase Cinema	1.49486
TMEM91	Transmembrane Protein 91	1.49486
ZNF527	Zinc Finger Protein 527	1.49486
HCG4B	HLA Complex Group 4B	1.49486
SNORA48	Small Nucleolar RNA, H/ACA Box 48	1.49486
ZNF230-DT	ZNF230 Divergent Transcript	1.49486
ENSG00000228395		1.49486
CICP5	Capicua Transcriptional Repressor Pseudogene 5	1.49486
ENSG00000204055		1.49486
ENSG00000249856		1.49486
ENSG00000186019		1.49486
lnc-DEGS2-3		1.49486
ENSG00000237669		1.49486
AF288738		1.49486
HSALNG0126379		1.49486
MN298114-235		1.49486
HSALNG0108403		1.49486
FGF11	Fibroblast Growth Factor 11	1.49486
DHX33	DEAH-Box Helicase 33	1.49462
WDR25	WD Repeat Domain 25	1.49423
ZNF143	Zinc Finger Protein 143	1.49423
ATP1A4	ATPase Na+/K+ Transporting Subunit Alpha 4	1.49399
EIF5B	Eukaryotic Translation Initiation Factor 5B	1.49382
ACADS	Acyl-CoA Dehydrogenase Short Chain	1.49379
ZFYVE21	Zinc Finger FYVE-Type Containing 21	1.49378
HLA-V	Major Histocompatibility Complex, Class I, V (Pseudogene)	1.49269
HIPK1	Homeodomain Interacting Protein Kinase 1	1.49256
CLHC1	Clathrin Heavy Chain Linker Domain Containing 1	1.49245
H2AC7	H2A Clustered Histone 7	1.49172

MIR545	MicroRNA 545	1.49160
NDOR1	NADPH Dependent Diflavin Oxidoreductase 1	1.49155
IMP3	IMP U3 Small Nucleolar Ribonucleoprotein 3	1.49120
DMC1	DNA Meiotic Recombinase 1	1.49087
CACNA2D3	Calcium Voltage-Gated Channel Auxiliary Subunit Alpha2delta 3	1.49022
TTC21A	Tetratricopeptide Repeat Domain 21A	1.49011
RPP21	Ribonuclease P/MRP Subunit P21	1.49011
PIRT	Phosphoinositide Interacting Regulator Of Transient Receptor Potential Channels	1.48992
PLA2G2E	Phospholipase A2 Group IIE	1.48878
ATG101	Autophagy Related 101	1.48878
C1orf127	Chromosome 1 Open Reading Frame 127	1.48868
NT5C3B	5'-Nucleotidase, Cytosolic IIIB	1.48868
DRG1	Developmentally Regulated GTP Binding Protein 1	1.48851
NABP2	Nucleic Acid Binding Protein 2	1.48767
EMID1	EMI Domain Containing 1	1.48739
TBL3	Transducin Beta Like 3	1.48721
NFIL3	Nuclear Factor, Interleukin 3 Regulated	1.48657
JPH4	Junctophilin 4	1.48640
MARCHF8	Membrane Associated Ring-CH-Type Finger 8	1.48634
SIAE	Sialic Acid Acetylesterase	1.48607
VAMP3	Vesicle Associated Membrane Protein 3	1.48588
SHMT2	Serine Hydroxymethyltransferase 2	1.48587
CFAP251	Cilia And Flagella Associated Protein 251	1.48567
NCAPH	Non-SMC Condensin I Complex Subunit H	1.48553
RPL13AP20	Ribosomal Protein L13a Pseudogene 20	1.48538
RAPGEFL1	Rap Guanine Nucleotide Exchange Factor Like 1	1.48483
ZNF551	Zinc Finger Protein 551	1.48374
TRAJ56	T Cell Receptor Alpha Joining 56	1.48374
SCOC	Short Coiled-Coil Protein	1.48372
PSMB1	Proteasome 20S Subunit Beta 1	1.48364
CCDC157	Coiled-Coil Domain Containing 157	1.48294
CCDC174	Coiled-Coil Domain Containing 174	1.48225
ACAP3	ArfGAP With Coiled-Coil, Ankyrin Repeat And PH Domains 3	1.48225
DTX1	Deltex E3 Ubiquitin Ligase 1	1.48196
SNORD94	Small Nucleolar RNA, C/D Box 94	1.48195
OXLD1	Oxidoreductase Like Domain Containing 1	1.48190

NRDE2	NRDE-2, Necessary For RNA Interference, Domain Containing	1.48164
MEOX1	Mesenchyme Homeobox 1	1.48163
RPL7L1	Ribosomal Protein L7 Like 1	1.48107
ANGPTL6	Angiopoietin Like 6	1.48072
E4F1	E4F Transcription Factor 1	1.48033
SEL1L3	SEL1L Family Member 3	1.48010
GPC2	Glypican 2	1.47889
OR9Q1	Olfactory Receptor Family 9 Subfamily Q Member 1	1.47820
PPP1R27	Protein Phosphatase 1 Regulatory Subunit 27	1.47820
ZNF326	Zinc Finger Protein 326	1.47791
PNPLA4	Patatin Like Phospholipase Domain Containing 4	1.47779
C10orf95	Chromosome 10 Open Reading Frame 95	1.47738
SCARF1	Scavenger Receptor Class F Member 1	1.47673
SORBS2	Sorbin And SH3 Domain Containing 2	1.47616
LARS2	Leucyl-TRNA Synthetase 2, Mitochondrial	1.47612
PRPS2	Phosphoribosyl Pyrophosphate Synthetase 2	1.47584
WBP11	WW Domain Binding Protein 11	1.47584
VWA7	Von Willebrand Factor A Domain Containing 7	1.47578
MIR519D	MicroRNA 519d	1.47463
TRIM69	Tripartite Motif Containing 69	1.47454
CABIN1	Calcineurin Binding Protein 1	1.47391
TXNL1	Thioredoxin Like 1	1.47362
DCXR	Dicarbonyl And L-Xylulose Reductase	1.47360
MIR1915	MicroRNA 1915	1.47356
CD300LD	CD300 Molecule Like Family Member D	1.47328
MIR520E	MicroRNA 520e	1.47321
ARMC12	Armadillo Repeat Containing 12	1.47300
ENSG00000287725		1.47300
ALDH16A1	Aldehyde Dehydrogenase 16 Family Member A1	1.47269
NAXE	NAD(P)HX Epimerase	1.47216
C9orf152	Chromosome 9 Open Reading Frame 152	1.47216
SHISA8	Shisa Family Member 8	1.47216
CST2	Cystatin SA	1.47124
NOXO1	NADPH Oxidase Organizer 1	1.47069
DOHH	Deoxyhypusine Hydroxylase	1.47060
ZNF792	Zinc Finger Protein 792	1.47060
TMEM234	Transmembrane Protein 234	1.47060

APOBEC3H	Apolipoprotein B MRNA Editing Enzyme Catalytic Subunit 3H	1.47036
PLD3	Phospholipase D Family Member 3	1.47016
DNAJC8	DnaJ Heat Shock Protein Family (Hsp40) Member C8	1.46979
CTR9	CTR9 Homolog, Paf1/RNA Polymerase II Complex Component	1.46957
MDN1	Midasin AAA ATPase 1	1.46946
DPY19L4	Dpy-19 Like 4	1.46942
POLRMT	RNA Polymerase Mitochondrial	1.46921
ZNF30	Zinc Finger Protein 30	1.46917
DACT2	Dishevelled Binding Antagonist Of Beta Catenin 2	1.46915
ACTRT2	Actin Related Protein T2	1.46904
RHBG	Rh Family B Glycoprotein	1.46849
ZNF239	Zinc Finger Protein 239	1.46849
ATP6V0B	ATPase H+ Transporting V0 Subunit B	1.46845
MTIF3	Mitochondrial Translational Initiation Factor 3	1.46822
MIR329-1	MicroRNA 329-1	1.46785
MCFD2	Multiple Coagulation Factor Deficiency 2, ER Cargo Receptor Complex Subunit	1.46717
THAP10	THAP Domain Containing 10	1.46611
SEPHS2	Selenophosphate Synthetase 2	1.46606
RAB3IP	RAB3A Interacting Protein	1.46589
STAU1	Staufen Double-Stranded RNA Binding Protein 1	1.46541
PITPNA-AS1	PITPNA Antisense RNA 1	1.46530
COPE	COPI Coat Complex Subunit Epsilon	1.46524
FGFRL1	Fibroblast Growth Factor Receptor Like 1	1.46499
ENSG00000260954		1.46475
AMPD3	Adenosine Monophosphate Deaminase 3	1.46473
H2AC1	H2A Clustered Histone 1	1.46471
IL26	Interleukin 26	1.46459
MIGA2	Mitoguardin 2	1.46429
HLA-DPB2	Major Histocompatibility Complex, Class II, DP Beta 2 (Pseudogene)	1.46418
LOC11125564[illegible]	TNFRSF10B 5' Regulatory Region	1.46360
LRFN2	Leucine Rich Repeat And Fibronectin Type III Domain Containing 2	1.46337
CLNS1A	Chloride Nucleotide-Sensitive Channel 1A	1.46328
KCNC4	Potassium Voltage-Gated Channel Subfamily C Member 4	1.46288
ERO1B	Endoplasmic Reticulum Oxidoreductase 1 Beta	1.46272
MEX3A	Mex-3 RNA Binding Family Member A	1.46188

PCK2	Phosphoenolpyruvate Carboxykinase 2, Mitochondrial	1.46179
FBN3	Fibrillin 3	1.46151
RAB32	RAB32, Member RAS Oncogene Family	1.46069
TMEM39A	Transmembrane Protein 39A	1.46055
MT1E	Metallothionein 1E	1.46041
FBRSL1	Fibrosin Like 1	1.46028
RMDN2	Regulator Of Microtubule Dynamics 2	1.45989
PDPR	Pyruvate Dehydrogenase Phosphatase Regulatory Subunit	1.45981
URM1	Ubiquitin Related Modifier 1	1.45936
CD164L2	CD164 Molecule Like 2	1.45936
TEX44	Testis Expressed 44	1.45936
IVD	Isovaleryl-CoA Dehydrogenase	1.45850
MARVELD1	MARVEL Domain Containing 1	1.45775
CHRNE	Cholinergic Receptor Nicotinic Epsilon Subunit	1.45762
GPR137	G Protein-Coupled Receptor 137	1.45672
GMFB	Glia Maturation Factor Beta	1.45605
C5orf24	Chromosome 5 Open Reading Frame 24	1.45540
MPST	Mercaptopyruvate Sulfurtransferase	1.45533
LYPLA2	Lysophospholipase 2	1.45531
DCAF6	DDB1 And CUL4 Associated Factor 6	1.45462
HECTD3	HECT Domain E3 Ubiquitin Protein Ligase 3	1.45403
FAM9C	Family With Sequence Similarity 9 Member C	1.45349
TXNDC5	Thioredoxin Domain Containing 5	1.45340
HLA-F-AS1	HLA-F Antisense RNA 1	1.45331
GTPBP1	GTP Binding Protein 1	1.45313
TNNT1	Troponin T1, Slow Skeletal Type	1.45241
NUDT2	Nudix Hydrolase 2	1.45238
UBASH3A	Ubiquitin Associated And SH3 Domain Containing A	1.45233
ST6GALNAC2	ST6 N-Acetylgalactosaminide Alpha-2,6-Sialyltransferase 2	1.45230
DECR2	2,4-Dienoyl-CoA Reductase 2	1.45204
ZNF232	Zinc Finger Protein 232	1.45196
SLC9A3R2	SLC9A3 Regulator 2	1.45161
MIR631	MicroRNA 631	1.45159
QARS1	Glutaminyl-TRNA Synthetase 1	1.45150
AP2A2	Adaptor Related Protein Complex 2 Subunit Alpha 2	1.45140
RBM26	RNA Binding Motif Protein 26	1.45093
TRIM47	Tripartite Motif Containing 47	1.45071
SCIN	Scinderin	1.45061
ADPRS	ADP-Ribosylserine Hydrolase	1.45050

PDCD6IP	Programmed Cell Death 6 Interacting Protein	1.45025
H1-1	H1.1 Linker Histone, Cluster Member	1.44944
MIR3196	MicroRNA 3196	1.44922
RBMS2	RNA Binding Motif Single Stranded Interacting Protein 2	1.44898
ZBTB33	Zinc Finger And BTB Domain Containing 33	1.44896
C5orf15	Chromosome 5 Open Reading Frame 15	1.44888
MEX3D	Mex-3 RNA Binding Family Member D	1.44884
LGSN	Lengsin, Lens Protein With Glutamine Synthetase Domain	1.44855
ALPK3	Alpha Kinase 3	1.44855
ANAPC13	Anaphase Promoting Complex Subunit 13	1.44795
MIR1296	MicroRNA 1296	1.44771
RHBDD1	Rhomboid Domain Containing 1	1.44765
POLD4	DNA Polymerase Delta 4, Accessory Subunit	1.44756
C16orf87	Chromosome 16 Open Reading Frame 87	1.44743
LBX2-AS1	LBX2 Antisense RNA 1	1.44738
IFT43	Intraflagellar Transport 43	1.44700
C11orf1	Chromosome 11 Open Reading Frame 1	1.44696
GCFC2	GC-Rich Sequence DNA-Binding Factor 2	1.44684
MED29	Mediator Complex Subunit 29	1.44656
LINC01436	Long Intergenic Non-Protein Coding RNA 1436	1.44635
SLC25A44	Solute Carrier Family 25 Member 44	1.44611
SGCB	Sarcoglycan Beta	1.44554
ADH6	Alcohol Dehydrogenase 6 (Class V)	1.44549
QSOX2	Quiescin Sulfhydryl Oxidase 2	1.44507
SEC14L3	SEC14 Like Lipid Binding 3	1.44489
KIR2DL1	Killer Cell Immunoglobulin Like Receptor, Two Ig Domains And Long Cytoplasmic Tail 1	1.44478
NIT2	Nitrilase Family Member 2	1.44450
TM4SF4	Transmembrane 4 L Six Family Member 4	1.44378
CHST15	Carbohydrate Sulfotransferase 15	1.44373
TNPO1	Transportin 1	1.44367
RGS19	Regulator Of G Protein Signaling 19	1.44365
SPON1	Spondin 1	1.44327
ENSG00000236263		1.44327
DNAH2	Dynein Axonemal Heavy Chain 2	1.44302
MIR487A	MicroRNA 487a	1.44284
STOML1	Stomatin Like 1	1.44277

FAM229A	Family With Sequence Similarity 229 Member A	1.44268
SPATA21	Spermatogenesis Associated 21	1.44246
CLIC3	Chloride Intracellular Channel 3	1.44184
NOL10	Nucleolar Protein 10	1.44103
SPNS1	Sphingolipid Transporter 1 (Putative)	1.44069
EML3	EMAP Like 3	1.43884
TRMT112	TRNA Methyltransferase Subunit 11-2	1.43856
WDR47	WD Repeat Domain 47	1.43850
CA14	Carbonic Anhydrase 14	1.43817
ADGRF1	Adhesion G Protein-Coupled Receptor F1	1.43757
RABGEF1	RAB Guanine Nucleotide Exchange Factor 1	1.43704
PADI2	Peptidyl Arginine Deiminase 2	1.43679
ZNF774	Zinc Finger Protein 774	1.43632
MIR758	MicroRNA 758	1.43594
PLS1	Plastin 1	1.43573
SLC35E1	Solute Carrier Family 35 Member E1	1.43551
ATG16L2	Autophagy Related 16 Like 2	1.43428
NPR2	Natriuretic Peptide Receptor 2	1.43396
HOXC-AS3	HOXC Cluster Antisense RNA 3	1.43360
NAA38	N-Alpha-Acetyltransferase 38, NatC Auxiliary Subunit	1.43331
DGKQ	Diacylglycerol Kinase Theta	1.43288
PAQR6	Progestin And AdipoQ Receptor Family Member 6	1.43280
MIR601	MicroRNA 601	1.43275
WFIKKN1	WAP, Follistatin/Kazal, Immunoglobulin, Kunitz And Netrin Domain Containing 1	1.43274
TEX51	Testis Expressed 51	1.43274
LINC02569	Long Intergenic Non-Protein Coding RNA 2569	1.43274
CYP2T1P	Cytochrome P450 Family 2 Subfamily T Member 1, Pseudogene	1.43274
ENSG00000268938		1.43274
ENSG00000265749		1.43274
ENSG00000269191		1.43274
RPL15P21	Ribosomal Protein L15 Pseudogene 21	1.43274
RNU6-1065P	RNA, U6 Small Nuclear 1065, Pseudogene	1.43274
ENSG00000275322		1.43274
ENSG00000235007		1.43274
lnc-PGPEP1L-27		1.43274
lnc-PTPN1-7		1.43274
HSALNG0085324		1.43274
lnc-DLX2-9		1.43274
LOC10537279	Uncharacterized LOC105372791	1.43274

IFI35	Interferon Induced Protein 35	1.43223
CDCP2	CUB Domain Containing Protein 2	1.43199
WASH4P	WASP Family Homolog 4, Pseudogene	1.43184
TCTN1	Tectonic Family Member 1	1.43167
JAKMIP3	Janus Kinase And Microtubule Interacting Protein 3	1.43167
ZNF541	Zinc Finger Protein 541	1.43167
BORCS5	BLOC-1 Related Complex Subunit 5	1.43152
MAN1A2	Mannosidase Alpha Class 1A Member 2	1.43110
MIR4513	MicroRNA 4513	1.43087
CCDC102A	Coiled-Coil Domain Containing 102A	1.43034
ZNF805	Zinc Finger Protein 805	1.43034
DET1	DET1 Partner Of COP1 E3 Ubiquitin Ligase	1.43022
DGCR11	DiGeorge Syndrome Critical Region Gene 11	1.42892
HERC3	HECT And RLD Domain Containing E3 Ubiquitin Protein Ligase 3	1.42875
MPP2	Membrane Palmitoylated Protein 2	1.42869
UNC5CL	Unc-5 Family C-Terminal Like	1.42865
GABPB2	GA Binding Protein Transcription Factor Subunit Beta 2	1.42839
TMEM212	Transmembrane Protein 212	1.42839
DYNC1LI1	Dynein Cytoplasmic 1 Light Intermediate Chain 1	1.42836
GTF2IP1	General Transcription Factor IIi Pseudogene 1	1.42727
BET1L	Bet1 Golgi Vesicular Membrane Trafficking Protein Like	1.42665
DUSP16	Dual Specificity Phosphatase 16	1.42640
GPBP1L1	GC-Rich Promoter Binding Protein 1 Like 1	1.42629
C2CD2L	C2CD2 Like	1.42618
APEX2	Apurinic/Apyrimidinic Endodeoxyribonuclease 2	1.42538
RNF212	Ring Finger Protein 212	1.42528
CCNYL1	Cyclin Y Like 1	1.42525
MIR607	MicroRNA 607	1.42516
CYP26C1	Cytochrome P450 Family 26 Subfamily C Member 1	1.42488
DEPP1	DEPP1 Autophagy Regulator	1.42421
AQP11	Aquaporin 11	1.42385
FAM169A	Family With Sequence Similarity 169 Member A	1.42352
ZNF576	Zinc Finger Protein 576	1.42352
ACADVL	Acyl-CoA Dehydrogenase Very Long Chain	1.42308
SEC13	SEC13 Homolog, Nuclear Pore And COPII Coat Complex Component	1.42299

DLGAP1	DLG Associated Protein 1	1.42266
SYF2	SYF2 Pre-MRNA Splicing Factor	1.42218
ETF1	Eukaryotic Translation Termination Factor 1	1.42210
TTBK2	Tau Tubulin Kinase 2	1.42196
P4HA3	Prolyl 4-Hydroxylase Subunit Alpha 3	1.42183
HEBP2	Heme Binding Protein 2	1.42161
ARRDC1	Arrestin Domain Containing 1	1.42161
CAB39	Calcium Binding Protein 39	1.42152
SLIRP	SRA Stem-Loop Interacting RNA Binding Protein	1.42150
DPF3	Double PHD Fingers 3	1.42134
SESN1	Sestrin 1	1.42122
NFU1	NFU1 Iron-Sulfur Cluster Scaffold	1.42093
ZNF700	Zinc Finger Protein 700	1.42058
HSPA12A	Heat Shock Protein Family A (Hsp70) Member 12A	1.42047
ARHGAP10	Rho GTPase Activating Protein 10	1.42046
KLHL26	Kelch Like Family Member 26	1.42018
VCAN-AS1	VCAN Antisense RNA 1	1.42007
ASGR1	Asialoglycoprotein Receptor 1	1.42004
USP11	Ubiquitin Specific Peptidase 11	1.41895
CBLN3	Cerebellin 3 Precursor	1.41885
LINC01394	Long Intergenic Non-Protein Coding RNA 1394	1.41885
CTRB1	Chymotrypsinogen B1	1.41855
MIR876	MicroRNA 876	1.41846
PABPC4	Poly(A) Binding Protein Cytoplasmic 4	1.41824
CIDEB	Cell Death Inducing DFFA Like Effector B	1.41807
SLC25A45	Solute Carrier Family 25 Member 45	1.41795
ZNF845	Zinc Finger Protein 845	1.41791
RENBP	Renin Binding Protein	1.41722
POGLUT1	Protein O-Glucosyltransferase 1	1.41710
TINAGL1	Tubulointerstitial Nephritis Antigen Like 1	1.41705
JDP2	Jun Dimerization Protein 2	1.41649
MIR298	MicroRNA 298	1.41631
ZSCAN25	Zinc Finger And SCAN Domain Containing 25	1.41564
SNHG19	Small Nucleolar RNA Host Gene 19	1.41521
PPP1R36	Protein Phosphatase 1 Regulatory Subunit 36	1.41471
ANAPC7	Anaphase Promoting Complex Subunit 7	1.41390
PHF5A	PHD Finger Protein 5A	1.41350
CORIN	Corin, Serine Peptidase	1.41258
EPHX3	Epoxide Hydrolase 3	1.41241

HCLS1	Hematopoietic Cell-Specific Lyn Substrate 1	1.41233
BLVRA	Biliverdin Reductase A	1.41229
ABHD4	Abhydrolase Domain Containing 4, N-Acyl Phospholipase B	1.41181
ZNF586	Zinc Finger Protein 586	1.41181
RBM41	RNA Binding Motif Protein 41	1.41181
CDPF1	Cysteine Rich DPF Motif Domain Containing 1	1.41181
TLL2	Tolloid Like 2	1.41159
SLC25A36	Solute Carrier Family 25 Member 36	1.41127
DCAF4	DDB1 And CUL4 Associated Factor 4	1.41126
NSMCE4A	NSE4 Homolog A, SMC5-SMC6 Complex Component	1.41121
MIR219A2	MicroRNA 219a-2	1.41057
GTPBP8	GTP Binding Protein 8 (Putative)	1.41045
SEZ6L	Seizure Related 6 Homolog Like	1.41039
ANKRD49	Ankyrin Repeat Domain 49	1.41031
TMEM259	Transmembrane Protein 259	1.40998
MIR3174	MicroRNA 3174	1.40994
H2AC19	H2A Clustered Histone 19	1.40981
ARHGAP29	Rho GTPase Activating Protein 29	1.40966
TPT1-AS1	TPT1 Antisense RNA 1	1.40942
SLC50A1	Solute Carrier Family 50 Member 1	1.40927
SPIRE2	Spire Type Actin Nucleation Factor 2	1.40910
UBA5	Ubiquitin Like Modifier Activating Enzyme 5	1.40859
MON1A	MON1 Homolog A, Secretory Trafficking Associated	1.40831
RSAD1	Radical S-Adenosyl Methionine Domain Containing 1	1.40793
ENO3	Enolase 3	1.40752
TMED8	Transmembrane P24 Trafficking Protein Family Member 8	1.40747
MIR1297	MicroRNA 1297	1.40746
NDUFS7	NADH:Ubiquinone Oxidoreductase Core Subunit S7	1.40702
ENSG00000285130		1.40685
TMCC2	Transmembrane And Coiled-Coil Domain Family 2	1.40680
SPG21	SPG21 Abhydrolase Domain Containing, Maspardin	1.40644
KDELR1	KDEL Endoplasmic Reticulum Protein Retention Receptor 1	1.40637
PPP1R35	Protein Phosphatase 1 Regulatory Subunit 35	1.40636
ACP7	Acid Phosphatase 7, Tartrate Resistant (Putative)	1.40636

LONRF1	LON Peptidase N-Terminal Domain And Ring Finger 1	1.40603
COBLL1	Cordon-Bleu WH2 Repeat Protein Like 1	1.40550
TMED4	Transmembrane P24 Trafficking Protein 4	1.40550
NIBAN3	Niban Apoptosis Regulator 3	1.40550
TOB2	Transducer Of ERBB2, 2	1.40506
RNPEPL1	Arginyl Aminopeptidase Like 1	1.40501
RNASEK	Ribonuclease K	1.40480
DDX42	DEAD-Box Helicase 42	1.40466
RBM27	RNA Binding Motif Protein 27	1.40466
PDE7A	Phosphodiesterase 7A	1.40458
REXO1	RNA Exonuclease 1 Homolog	1.40392
MIR6872	MicroRNA 6872	1.40346
CPEB1	Cytoplasmic Polyadenylation Element Binding Protein 1	1.40323
CPLX3	Complexin 3	1.40292
EIF3C	Eukaryotic Translation Initiation Factor 3 Subunit C	1.40274
ST3GAL6	ST3 Beta-Galactoside Alpha-2,3-Sialyltransferase 6	1.40249
PLEKHD1	Pleckstrin Homology And Coiled-Coil Domain Containing D1	1.40239
HCG4	HLA Complex Group 4	1.40239
TRPA1	Transient Receptor Potential Cation Channel Subfamily A Member 1	1.40217
TDRD9	Tudor Domain Containing 9	1.40208
SEC62	SEC62 Homolog, Preprotein Translocation Factor	1.40207
NSUN5	NOP2/Sun RNA Methyltransferase 5	1.40201
DYNLL2	Dynein Light Chain LC8-Type 2	1.40196
GLP2R	Glucagon Like Peptide 2 Receptor	1.40176
DNAH14	Dynein Axonemal Heavy Chain 14	1.40158
SRXN1	Sulfiredoxin 1	1.40013
ZNF503	Zinc Finger Protein 503	1.40002
ETV3	ETS Variant Transcription Factor 3	1.39872
B4GALNT4	Beta-1,4-N-Acetyl-Galactosaminyltransferase 4	1.39864
NT5DC2	5'-Nucleotidase Domain Containing 2	1.39852
NYNRIN	NYN Domain And Retroviral Integrase Containing	1.39850
PRICKLE1	Prickle Planar Cell Polarity Protein 1	1.39821
C1orf174	Chromosome 1 Open Reading Frame 174	1.39815
C1orf198	Chromosome 1 Open Reading Frame 198	1.39815
IL6-AS1	IL6 Antisense RNA 1	1.39739
SURF2	Surfeit 2	1.39655

AKAP5	A-Kinase Anchoring Protein 5	1.39645
IP6K1	Inositol Hexakisphosphate Kinase 1	1.39621
TBC1D10C	TBC1 Domain Family Member 10C	1.39621
COPG2IT1	COPG2 Imprinted Transcript 1	1.39617
MTFMT	Mitochondrial Methionyl-TRNA Formyltransferase	1.39576
KCNQ1DN	KCNQ1 Downstream Neighbor	1.39532
NAALADL1	N-Acetylated Alpha-Linked Acidic Dipeptidase Like 1	1.39513
C6orf201	Chromosome 6 Open Reading Frame 201	1.39513
MICALCL	MICAL C-Terminal Like	1.39513
GGTA1	Glycoprotein Alpha-Galactosyltransferase 1 (Inactive)	1.39513
GUSBP3	GUSB Pseudogene 3	1.39513
ENSG00000254732		1.39513
LY86-AS1	LY86 Antisense RNA 1	1.39505
MKRN1	Makorin Ring Finger Protein 1	1.39458
ANKRD22	Ankyrin Repeat Domain 22	1.39396
CHRNG	Cholinergic Receptor Nicotinic Gamma Subunit	1.39338
EXOC2	Exocyst Complex Component 2	1.39335
MAGI3	Membrane Associated Guanylate Kinase, WW And PDZ Domain Containing 3	1.39333
PINLYP	Phospholipase A2 Inhibitor And LY6/PLAUR Domain Containing	1.39265
NDFIP2	Nedd4 Family Interacting Protein 2	1.39261
SPATA2	Spermatogenesis Associated 2	1.39251
P2RX4	Purinergic Receptor P2X 4	1.39237
GLRB	Glycine Receptor Beta	1.39221
CYB5R1	Cytochrome B5 Reductase 1	1.39217
CNOT4	CCR4-NOT Transcription Complex Subunit 4	1.39211
TTC31	Tetratricopeptide Repeat Domain 31	1.39211
TICRR	TOPBP1 Interacting Checkpoint And Replication Regulator	1.39162
SEMA6A	Semaphorin 6A	1.39155
RPS19BP1	Ribosomal Protein S19 Binding Protein 1	1.39148
GALP	Galanin Like Peptide	1.39111
MXD3	MAX Dimerization Protein 3	1.39040
RAB22A	RAB22A, Member RAS Oncogene Family	1.39032
STBD1	Starch Binding Domain 1	1.38990
SCARNA4	Small Cajal Body-Specific RNA 4	1.38965
ANKRD52	Ankyrin Repeat Domain 52	1.38930
FAM83G	Family With Sequence Similarity 83 Member G	1.38930

NGB	Neuroglobin	1.38902
NDUFA4	NDUFA4 Mitochondrial Complex Associated	1.38848
FASTK	Fas Activated Serine/Threonine Kinase	1.38810
HIVEP2	HIVEP Zinc Finger 2	1.38757
ENSG00000260051		1.38747
ENSG00000261505		1.38747
TGS1	Trimethylguanosine Synthase 1	1.38596
NPLOC4	NPL4 Homolog, Ubiquitin Recognition Factor	1.38515
NUP153	Nucleoporin 153	1.38511
DLGAP1-AS2	DLGAP1 Antisense RNA 2	1.38502
NCBP3	Nuclear Cap Binding Subunit 3	1.38467
THOP1	Thimet Oligopeptidase 1	1.38455
RAB5B	RAB5B, Member RAS Oncogene Family	1.38426
KIF13B	Kinesin Family Member 13B	1.38426
CYP21A1P	Cytochrome P450 Family 21 Subfamily A Member 1, Pseudogene	1.38415
CRNKL1	Crooked Neck Pre-MRNA Splicing Factor 1	1.38406
ATP6AP2	ATPase H+ Transporting Accessory Protein 2	1.38329
NAT14	N-Acetyltransferase 14 (Putative)	1.38269
KCTD10	Potassium Channel Tetramerization Domain Containing 10	1.38219
ZNF33B	Zinc Finger Protein 33B	1.38194
ASB16-AS1	ASB16 Antisense RNA 1	1.38194
RANBP10	RAN Binding Protein 10	1.38169
ZNF23	Zinc Finger Protein 23	1.38141
RUFY4	RUN And FYVE Domain Containing 4	1.38123
MYORG	Myogenesis Regulating Glycosidase (Putative)	1.38123
DNAJC12	DnaJ Heat Shock Protein Family (Hsp40) Member C12	1.38120
POU3F1	POU Class 3 Homeobox 1	1.38116
NME3	NME/NM23 Nucleoside Diphosphate Kinase 3	1.38067
LOC10672841[illegible]	LEP 5' Regulatory Region	1.37978
FAM120AOS	Family With Sequence Similarity 120A Opposite Strand	1.37972
MT1X	Metallothionein 1X	1.37955
PSME1	Proteasome Activator Subunit 1	1.37888
P2RX5	Purinergic Receptor P2X 5	1.37886
ENSA	Endosulfine Alpha	1.37886
ME1	Malic Enzyme 1	1.37793
REM2	RRAD And GEM Like GTPase 2	1.37770
ZNF606	Zinc Finger Protein 606	1.37770

FAM187B	Family With Sequence Similarity 187 Member B	1.37770
NPPA-AS1	NPPA Antisense RNA 1	1.37770
ENSG00000222022		1.37770
SUGCT	Succinyl-CoA:Glutarate-CoA Transferase	1.37726
LINC00184	Long Intergenic Non-Protein Coding RNA 184	1.37689
MIR486-2	MicroRNA 486-2	1.37651
TAF7	TATA-Box Binding Protein Associated Factor 7	1.37639
GPRIN1	G Protein Regulated Inducer Of Neurite Outgrowth 1	1.37636
WASHC2C	WASH Complex Subunit 2C	1.37636
HES7	Hes Family BHLH Transcription Factor 7	1.37622
ASB6	Ankyrin Repeat And SOCS Box Containing 6	1.37604
CLPP	Caseinolytic Mitochondrial Matrix Peptidase Proteolytic Subunit	1.37580
MMP28	Matrix Metallopeptidase 28	1.37572
HOXC11	Homeobox C11	1.37555
SCFD1	Sec1 Family Domain Containing 1	1.37553
C16orf96	Chromosome 16 Open Reading Frame 96	1.37532
MIR329-2	MicroRNA 329-2	1.37508
PAPSS1	3'-Phosphoadenosine 5'-Phosphosulfate Synthase 1	1.37496
TTC7B	Tetratricopeptide Repeat Domain 7B	1.37445
C19orf54	Chromosome 19 Open Reading Frame 54	1.37445
PEF1	Penta-EF-Hand Domain Containing 1	1.37434
ZNF382	Zinc Finger Protein 382	1.37347
NUDT19	Nudix Hydrolase 19	1.37291
SWT1	SWT1 RNA Endoribonuclease Homolog	1.37261
CYB5B	Cytochrome B5 Type B	1.37233
CD160	CD160 Molecule	1.37233
CAND2	Cullin Associated And Neddylation Dissociated 2 (Putative)	1.37127
NUDCD1	NudC Domain Containing 1	1.37122
ST7L	Suppression Of Tumorigenicity 7 Like	1.37118
FBXL3	F-Box And Leucine Rich Repeat Protein 3	1.37109
SPDYE1	Speedy/RINGO Cell Cycle Regulator Family Member E1	1.37090
RIC8A	RIC8 Guanine Nucleotide Exchange Factor A	1.37064

PAFAH1B3	Platelet Activating Factor Acetylhydrolase 1b Catalytic Subunit 3	1.37025
GDPD4	Glycerophosphodiester Phosphodiesterase Domain Containing 4	1.37020
XCL2	X-C Motif Chemokine Ligand 2	1.37000
PDK3	Pyruvate Dehydrogenase Kinase 3	1.36962
EPB41L5	Erythrocyte Membrane Protein Band 4.1 Like 5	1.36886
GRM8	Glutamate Metabotropic Receptor 8	1.36882
SNX27	Sorting Nexin 27	1.36866
ZNF296	Zinc Finger Protein 296	1.36659
SRP54-AS1	SRP54 Antisense RNA 1	1.36659
NOP53-AS1	NOP53 Antisense RNA 1	1.36659
ENSG00000267834		1.36659
YWHAQP6	YWHAQ Pseudogene 6	1.36659
RN7SL688P	RNA, 7SL, Cytoplasmic 688, Pseudogene	1.36659
SNRPEP4	SNRPE Pseudogene 4	1.36659
ENSG00000258982		1.36659
SEPTIN7P13	Septin 7 Pseudogene 13	1.36659
ENSG00000284876		1.36659
ENSG00000286463		1.36659
FJ601684-278		1.36659
HSALNG0113303		1.36659
HSALNG0113304		1.36659
HSALNG0087292		1.36659
HSALNG0020382		1.36659
HSALNG0018036		1.36659
lnc-PSD4-4		1.36659
HSALNG0018038		1.36659
GPATCH8	G-Patch Domain Containing 8	1.36646
GCNT1	Glucosaminyl (N-Acetyl) Transferase 1	1.36567
TNRC6A	Trinucleotide Repeat Containing Adaptor 6A	1.36505
CPN2	Carboxypeptidase N Subunit 2	1.36495
SGSM2	Small G Protein Signaling Modulator 2	1.36495
DNAJA4	DnaJ Heat Shock Protein Family (Hsp40) Member A4	1.36495
AVIL	Advillin	1.36455
ADGRG5	Adhesion G Protein-Coupled Receptor G5	1.36455
CRELD1	Cysteine Rich With EGF Like Domains 1	1.36429
OPRK1	Opioid Receptor Kappa 1	1.36426
BMP8A	Bone Morphogenetic Protein 8a	1.36418
HEPH	Hephaestin	1.36408
MIR515-2	MicroRNA 515-2	1.36398
TSEN15	TRNA Splicing Endonuclease Subunit 15	1.36396

TLR8-AS1	TLR8 Antisense RNA 1	1.36394
SLC44A1	Solute Carrier Family 44 Member 1	1.36302
RAB40C	RAB40C, Member RAS Oncogene Family	1.36298
ZNF689	Zinc Finger Protein 689	1.36252
TMEM167B	Transmembrane Protein 167B	1.36252
MASTL	Microtubule Associated Serine/Threonine Kinase Like	1.36194
SUPT20H	SPT20 Homolog, SAGA Complex Component	1.36149
UBE2E3	Ubiquitin Conjugating Enzyme E2 E3	1.36136
ATP13A3	ATPase 13A3	1.36119
KLHDC8A	Kelch Domain Containing 8A	1.36006
DCAKD	Dephospho-CoA Kinase Domain Containing	1.35982
H1-8	H1.8 Linker Histone	1.35975
NOL6	Nucleolar Protein 6	1.35968
NLRP12	NLR Family Pyrin Domain Containing 12	1.35949
PKN2	Protein Kinase N2	1.35886
KLF16	Kruppel Like Factor 16	1.35856
CAMSAP3	Calmodulin Regulated Spectrin Associated Protein Family Member 3	1.35825
DPY30	Dpy-30 Histone Methyltransferase Complex Regulatory Subunit	1.35816
C1QTNF2	C1q And TNF Related 2	1.35746
ZBED8	Zinc Finger BED-Type Containing 8	1.35746
PCYT2	Phosphate Cytidylyltransferase 2, Ethanolamine	1.35671
CSPP1	Centrosome And Spindle Pole Associated Protein 1	1.35640
ANGPTL7	Angiopoietin Like 7	1.35639
NUBP1	Nucleotide Binding Protein 1	1.35627
CHAF1B	Chromatin Assembly Factor 1 Subunit B	1.35608
SLC5A10	Solute Carrier Family 5 Member 10	1.35595
DUSP15	Dual Specificity Phosphatase 15	1.35595
TMEM126A	Transmembrane Protein 126A	1.35579
IFT80	Intraflagellar Transport 80	1.35577
TLE5	TLE Family Member 5, Transcriptional Modulator	1.35544
SH3GLB2	SH3 Domain Containing GRB2 Like, Endophilin B2	1.35542
TIMD4	T Cell Immunoglobulin And Mucin Domain Containing 4	1.35466
OASL	2'-5'-Oligoadenylate Synthetase Like	1.35408
UBE2L6	Ubiquitin Conjugating Enzyme E2 L6	1.35405
DIP2A	Disco Interacting Protein 2 Homolog A	1.35349
PKMYT1	Protein Kinase, Membrane Associated Tyrosine/Threonine 1	1.35320

SKAP1	Src Kinase Associated Phosphoprotein 1	1.35287
ZC3H4	Zinc Finger CCCH-Type Containing 4	1.35280
ZBTB25	Zinc Finger And BTB Domain Containing 25	1.35276
CTBP1-DT	CTBP1 Divergent Transcript	1.35276
ZNF701	Zinc Finger Protein 701	1.35192
GAMT	Guanidinoacetate N-Methyltransferase	1.35105
PROZ	Protein Z, Vitamin K Dependent Plasma Glycoprotein	1.35081
ADAM33	ADAM Metallopeptidase Domain 33	1.35050
SLC44A3	Solute Carrier Family 44 Member 3	1.35001
CEPT1	Choline/Ethanolamine Phosphotransferase 1	1.34896
NDUFS6	NADH:Ubiquinone Oxidoreductase Subunit S6	1.34856
THAP12	THAP Domain Containing 12	1.34772
CNIH3	Cornichon Family AMPA Receptor Auxiliary Protein 3	1.34695
LOXL3	Lysyl Oxidase Like 3	1.34683
PSME2	Proteasome Activator Subunit 2	1.34654
ENSG00000253102		1.34612
NDUFB8	NADH:Ubiquinone Oxidoreductase Subunit B8	1.34602
TBC1D1	TBC1 Domain Family Member 1	1.34590
UBXN11	UBX Domain Protein 11	1.34590
KLHDC10	Kelch Domain Containing 10	1.34585
ZNF688	Zinc Finger Protein 688	1.34585
NUTM2B-AS1	NUTM2B Antisense RNA 1	1.34585
RPS10P7	Ribosomal Protein S10 Pseudogene 7	1.34585
ENSG00000285245		1.34585
HSF2BP	Heat Shock Transcription Factor 2 Binding Protein	1.34439
PRR3	Proline Rich 3	1.34439
ATP23	ATP23 Metallopeptidase And ATP Synthase Assembly Factor Homolog	1.34439
TPK1	Thiamin Pyrophosphokinase 1	1.34413
MIR4729	MicroRNA 4729	1.34367
NSUN3	NOP2/Sun RNA Methyltransferase 3	1.34289
IPO9	Importin 9	1.34253
SUGT1	SGT1 Homolog, MIS12 Kinetochore Complex Assembly Cochaperone	1.34231
SRSF11	Serine And Arginine Rich Splicing Factor 11	1.34215
POLR2I	RNA Polymerase II Subunit I	1.34184
COPS7B	COP9 Signalosome Subunit 7B	1.34184
VIPAS39	VPS33B Interacting Protein, Apical-Basolateral Polarity Regulator, Spe-39 Homolog	1.34157
MRPL46	Mitochondrial Ribosomal Protein L46	1.34108

PBLD	Phenazine Biosynthesis Like Protein Domain Containing	1.34072
SPTBN5	Spectrin Beta, Non-Erythrocytic 5	1.34048
BTBD16	BTB Domain Containing 16	1.34018
NOC4L	Nucleolar Complex Associated 4 Homolog	1.34016
C1orf35	Chromosome 1 Open Reading Frame 35	1.34016
OSBPL2	Oxysterol Binding Protein Like 2	1.33934
RNF169	Ring Finger Protein 169	1.33856
FOXN2	Forkhead Box N2	1.33774
RPL37A	Ribosomal Protein L37a	1.33673
ELMOD2	ELMO Domain Containing 2	1.33638
HIGD2A	HIG1 Hypoxia Inducible Domain Family Member 2A	1.33586
KCTD12	Potassium Channel Tetramerization Domain Containing 12	1.33567
TRPV2	Transient Receptor Potential Cation Channel Subfamily V Member 2	1.33552
PGRMC2	Progesterone Receptor Membrane Component 2	1.33538
RXFP4	Relaxin Family Peptide/INSL5 Receptor 4	1.33489
EVA1B	Eva-1 Homolog B	1.33489
RASSF8	Ras Association Domain Family Member 8	1.33391
ARL13B	ADP Ribosylation Factor Like GTPase 13B	1.33322
MIR524	MicroRNA 524	1.33290
ANGEL1	Angel Homolog 1	1.33236
KRTAP10-1	Keratin Associated Protein 10-1	1.33211
PCYOX1L	Prenylcysteine Oxidase 1 Like	1.33200
UFM1	Ubiquitin Fold Modifier 1	1.33143
GGN	Gametogenetin	1.33038
PIH1D1	PIH1 Domain Containing 1	1.33032
PDCD2	Programmed Cell Death 2	1.33027
ZBTB2	Zinc Finger And BTB Domain Containing 2	1.33015
RBMS3	RNA Binding Motif Single Stranded Interacting Protein 3	1.33007
MIR509-3	MicroRNA 509-3	1.32894
B3GALT5-AS1	B3GALT5 Antisense RNA 1	1.32885
SEC11C	SEC11 Homolog C, Signal Peptidase Complex Subunit	1.32800
SLFN5	Schlafen Family Member 5	1.32789
KLHL11	Kelch Like Family Member 11	1.32742
SMG1	SMG1 Nonsense Mediated MRNA Decay Associated PI3K Related Kinase	1.32706
CYP2U1	Cytochrome P450 Family 2 Subfamily U Member 1	1.32685

GSTT2B	Glutathione S-Transferase Theta 2B	1.32660
TRIM13	Tripartite Motif Containing 13	1.32650
OLFML1	Olfactomedin Like 1	1.32585
EML2	EMAP Like 2	1.32582
TTC38	Tetratricopeptide Repeat Domain 38	1.32534
NAP1L3	Nucleosome Assembly Protein 1 Like 3	1.32507
LRRC14	Leucine Rich Repeat Containing 14	1.32429
SMCR5	Smith-Magenis Syndrome Chromosome Region, Candidate 5	1.32429
SMIM2-IT1	SMIM2 Intronic Transcript 1	1.32429
VAC14-AS1	VAC14 Antisense RNA 1	1.32429
PRR14L	Proline Rich 14 Like	1.32410
ZNF134	Zinc Finger Protein 134	1.32410
EVI5L	Ecotropic Viral Integration Site 5 Like	1.32392
VPS26A	VPS26, Retromer Complex Component A	1.32213
C1orf21	Chromosome 1 Open Reading Frame 21	1.32203
CNOT2	CCR4-NOT Transcription Complex Subunit 2	1.32089
SBF1	SET Binding Factor 1	1.32050
PSME3IP1	Proteasome Activator Subunit 3 Interacting Protein 1	1.32041
CA7	Carbonic Anhydrase 7	1.32022
SUCLG1	Succinate-CoA Ligase GDP/ADP-Forming Subunit Alpha	1.32021
P2RY1	Purinergic Receptor P2Y1	1.32011
ZNF597	Zinc Finger Protein 597	1.31998
TNFAIP8L1	TNF Alpha Induced Protein 8 Like 1	1.31992
TMPRSS5	Transmembrane Serine Protease 5	1.31982
SWSAP1	SWIM-Type Zinc Finger 7 Associated Protein 1	1.31982
ARPC5L	Actin Related Protein 2/3 Complex Subunit 5 Like	1.31854
PRR19	Proline Rich 19	1.31822
SNORD42B	Small Nucleolar RNA, C/D Box 42B	1.31731
RAB3GAP2	RAB3 GTPase Activating Non-Catalytic Protein Subunit 2	1.31724
PAQR4	Progestin And AdipoQ Receptor Family Member 4	1.31675
LINC00629	Long Intergenic Non-Protein Coding RNA 629	1.31655
VASN	Vasorin	1.31616
GALNT16	Polypeptide N-Acetylgalactosaminyltransferase 16	1.31569
SH3BGRL3	SH3 Domain Binding Glutamate Rich Protein Like 3	1.31547
WNK4	WNK Lysine Deficient Protein Kinase 4	1.31477
MIR4492	MicroRNA 4492	1.31360

KCNU1	Potassium Calcium-Activated Channel Subfamily U Member 1	1.31351
LMAN1L	Lectin, Mannose Binding 1 Like	1.31328
ADAT1	Adenosine Deaminase TRNA Specific 1	1.31314
PLA1A	Phospholipase A1 Member A	1.31303
PIGT	Phosphatidylinositol Glycan Anchor Biosynthesis Class T	1.31289
ZNF324	Zinc Finger Protein 324	1.31192
WRAP73	WD Repeat Containing, Antisense To TP73	1.31192
CRYBB1	Crystallin Beta B1	1.31168
TOR1AIP2	Torsin 1A Interacting Protein 2	1.31157
SMPD3	Sphingomyelin Phosphodiesterase 3	1.31142
CLN5	CLN5 Intracellular Trafficking Protein	1.31137
ZNF785	Zinc Finger Protein 785	1.31130
LINC01588	Long Intergenic Non-Protein Coding RNA 1588	1.31130
TMEM92-AS1	TMEM92 Antisense RNA 1	1.31130
TMEM51-AS2	TMEM51 Antisense RNA 2	1.31130
TRAIP	TRAF Interacting Protein	1.31118
MPI	Mannose Phosphate Isomerase	1.31089
ZFYVE16	Zinc Finger FYVE-Type Containing 16	1.31077
SIMC1	SUMO Interacting Motifs Containing 1	1.31066
CATSPERE	Catsper Channel Auxiliary Subunit Epsilon	1.31056
ZNF14	Zinc Finger Protein 14	1.30962
HSALNG0007483		1.30943
RILP	Rab Interacting Lysosomal Protein	1.30939
MIR603	MicroRNA 603	1.30904
TMEM184B	Transmembrane Protein 184B	1.30887
ABHD8	Abhydrolase Domain Containing 8	1.30887
APOLD1	Apolipoprotein L Domain Containing 1	1.30866
P4HTM	Prolyl 4-Hydroxylase, Transmembrane	1.30833
CGREF1	Cell Growth Regulator With EF-Hand Domain 1	1.30826
KCNJ12	Potassium Inwardly Rectifying Channel Subfamily J Member 12	1.30706
RBM45	RNA Binding Motif Protein 45	1.30698
APOBEC1	Apolipoprotein B MRNA Editing Enzyme Catalytic Subunit 1	1.30688
TMUB2	Transmembrane And Ubiquitin Like Domain Containing 2	1.30601
H2BC9	H2B Clustered Histone 9	1.30597
SIGLEC1	Sialic Acid Binding Ig Like Lectin 1	1.30540
MDGA2	MAM Domain Containing Glycosylphosphatidylinositol Anchor 2	1.30508
KDM4D	Lysine Demethylase 4D	1.30386
ARMC2	Armadillo Repeat Containing 2	1.30378

CDKN2AIPNL	CDKN2A Interacting Protein N-Terminal Like	1.30332
BMERB1	BMERB Domain Containing 1	1.30332
RPL32P3	Ribosomal Protein L32 Pseudogene 3	1.30332
PXN-AS1	PXN Antisense RNA 1	1.30332
NUP50	Nucleoporin 50	1.30324
KRT79	Keratin 79	1.30324
RAB3D	RAB3D, Member RAS Oncogene Family	1.30282
CCS	Copper Chaperone For Superoxide Dismutase	1.30244
RBMX2	RNA Binding Motif Protein X-Linked 2	1.30240
PARG	Poly(ADP-Ribose) Glycohydrolase	1.30214
SNX11	Sorting Nexin 11	1.30210
LOC10272426!	Uncharacterized LOC102724265	1.30199
ENSG00000268836		1.30199
ENSG00000269482		1.30199
lnc-DVL1-2		1.30199
HSALNG0000138		1.30199
lnc-DVL1-1		1.30199
HSALNG0000137		1.30199
HSALNG0000139		1.30199
HSALNG0000142		1.30199
piR-35674-001		1.30199
NONHSAG000093.2		1.30199
CREM	CAMP Responsive Element Modulator	1.30105
MICOS10-NBL	MICOS10-NBL1 Readthrough	1.30067
HSPBP1	HSPA (Hsp70) Binding Protein 1	1.30017
MTO1	Mitochondrial TRNA Translation Optimization 1	1.30016
MIR3677HG	MIR3677 And MIR940 Host Gene	1.30012
ARL8B	ADP Ribosylation Factor Like GTPase 8B	1.29958
CTF1	Cardiotrophin 1	1.29928
ATP6V1C1	ATPase H+ Transporting V1 Subunit C1	1.29841
ESYT1	Extended Synaptotagmin 1	1.29841
PTGIR	Prostaglandin I2 Receptor	1.29801
ITIH3	Inter-Alpha-Trypsin Inhibitor Heavy Chain 3	1.29712
GJC1	Gap Junction Protein Gamma 1	1.29697
VSX1	Visual System Homeobox 1	1.29600
NSUN4	NOP2/Sun RNA Methyltransferase 4	1.29587
ARPC4	Actin Related Protein 2/3 Complex Subunit 4	1.29570
PCSK1N	Proprotein Convertase Subtilisin/Kexin Type 1 Inhibitor	1.29554
IQCH-AS1	IQCH Antisense RNA 1	1.29552
PTPRVP	Protein Tyrosine Phosphatase Receptor Type V, Pseudogene	1.29552

LOC10099684[illegible]	Uncharacterized LOC100996842	1.29552
LOC10192772[illegible]	Uncharacterized LOC101927727	1.29552
ENSG00000254873		1.29552
RPL5P30	Ribosomal Protein L5 Pseudogene 30	1.29552
FTLP14	Ferritin Light Chain Pseudogene 14	1.29552
ENSG00000255176		1.29552
ENSG00000212579		1.29552
ENSG00000252040		1.29552
ENSG00000255384		1.29552
ENSG00000252640		1.29552
lnc-BCL3-1		1.29552
lnc-ZCCHC24-7		1.29552
HSALNG0057834		1.29552
lnc-TMEM238L-2		1.29552
RF00017-4977		1.29552
RF00017-950		1.29552
HSALNG0079128		1.29552
piR-39628		1.29552
HSALNG0109608		1.29552
C15orf39	Chromosome 15 Open Reading Frame 39	1.29505
SIGIRR	Single Ig And TIR Domain Containing	1.29503
ABHD3	Abhydrolase Domain Containing 3, Phospholipase	1.29463
CARNS1	Carnosine Synthase 1	1.29463
TAC4	Tachykinin Precursor 4	1.29463
HOATZ	HOATZ Cilia And Flagella Associated Protein	1.29463
ENSG00000255835		1.29463
TRAJ23	T Cell Receptor Alpha Joining 23	1.29463
FOXL3	Forkhead Box L3	1.29463
SRP9	Signal Recognition Particle 9	1.29403
NAPRT	Nicotinate Phosphoribosyltransferase	1.29394
NPC2	NPC Intracellular Cholesterol Transporter 2	1.29341
MIR610	MicroRNA 610	1.29264
MIR650	MicroRNA 650	1.29242
TIAF1	TGFB1-Induced Anti-Apoptotic Factor 1	1.29231
STKLD1	Serine/Threonine Kinase Like Domain Containing 1	1.29231
ZNF598	Zinc Finger Protein 598, E3 Ubiquitin Ligase	1.29224
MIS12	MIS12 Kinetochore Complex Component	1.29219
SELENOK	Selenoprotein K	1.29185
MIR938	MicroRNA 938	1.29168
TPPP	Tubulin Polymerization Promoting Protein	1.29159

NDUFC1	NADH:Ubiquinone Oxidoreductase Subunit C1	1.29155
HMGCL	3-Hydroxy-3-Methylglutaryl-CoA Lyase	1.29118
IL1RAP	Interleukin 1 Receptor Accessory Protein	1.29116
MIR660	MicroRNA 660	1.29113
CYB561	Cytochrome B561	1.29099
CEBPG	CCAAT Enhancer Binding Protein Gamma	1.29099
ATCAY	ATCAY Kinesin Light Chain Interacting Caytaxin	1.29074
MIR4516	MicroRNA 4516	1.28911
RAD54L2	RAD54 Like 2	1.28794
EP400P1	EP400 Pseudogene 1	1.28794
ZNF584	Zinc Finger Protein 584	1.28741
MIR552	MicroRNA 552	1.28715
LINC00703	Long Intergenic Non-Protein Coding RNA 703	1.28702
TTLL11	Tubulin Tyrosine Ligase Like 11	1.28685
INPP5B	Inositol Polyphosphate-5-Phosphatase B	1.28643
SOCS7	Suppressor Of Cytokine Signaling 7	1.28514
TRIM23	Tripartite Motif Containing 23	1.28393
DYNC2I2	Dynein 2 Intermediate Chain 2	1.28377
MIR1237	MicroRNA 1237	1.28361
MAPK15	Mitogen-Activated Protein Kinase 15	1.28340
SNAPC4	Small Nuclear RNA Activating Complex Polypeptide 4	1.28303
SAR1B	Secretion Associated Ras Related GTPase 1B	1.28301
ACSS2	Acyl-CoA Synthetase Short Chain Family Member 2	1.28294
MIR488	MicroRNA 488	1.28260
CPAMD8	C3 And PZP Like Alpha-2-Macroglobulin Domain Containing 8	1.28257
ATXN7L3	Ataxin 7 Like 3	1.28240
DNAJB9	DnaJ Heat Shock Protein Family (Hsp40) Member B9	1.28232
KLHL12	Kelch Like Family Member 12	1.28173
ZNF559	Zinc Finger Protein 559	1.28148
ZNF747	Zinc Finger Protein 747	1.28148
TSGA10IP	Testis Specific 10 Interacting Protein	1.28148
TTYH1	Tweety Family Member 1	1.27973
TOP1MT	DNA Topoisomerase I Mitochondrial	1.27963
SNX25	Sorting Nexin 25	1.27946
GCHFR	GTP Cyclohydrolase I Feedback Regulator	1.27937
CPLANE2	Ciliogenesis And Planar Polarity Effector 2	1.27937

RAI14	Retinoic Acid Induced 14	1.27898
TROAP	Trophinin Associated Protein	1.27898
CRLF1	Cytokine Receptor Like Factor 1	1.27855
AUH	AU RNA Binding Methylglutaconyl-CoA Hydratase	1.27806
CEACAM16	CEA Cell Adhesion Molecule 16, Tectorial Membrane Component	1.27806
LRRC27	Leucine Rich Repeat Containing 27	1.27792
PLTP	Phospholipid Transfer Protein	1.27607
NTNG2	Netrin G2	1.27605
MT1F	Metallothionein 1F	1.27577
MRPL43	Mitochondrial Ribosomal Protein L43	1.27520
ZNF407	Zinc Finger Protein 407	1.27520
MIR1178	MicroRNA 1178	1.27520
OR4D1	Olfactory Receptor Family 4 Subfamily D Member 1	1.27518
KAZALD1	Kazal Type Serine Peptidase Inhibitor Domain 1	1.27476
DFFB	DNA Fragmentation Factor Subunit Beta	1.27329
ENSG00000197670		1.27273
AC116366.1		1.27273
WIZ	WIZ Zinc Finger	1.27227
SMIM29	Small Integral Membrane Protein 29	1.27137
SLC25A24	Solute Carrier Family 25 Member 24	1.27124
PIPOX	Pipecolic Acid And Sarcosine Oxidase	1.27084
MIR4681	MicroRNA 4681	1.27070
ATP5F1E	ATP Synthase F1 Subunit Epsilon	1.27049
ZNF211	Zinc Finger Protein 211	1.27024
RSRP1	Arginine And Serine Rich Protein 1	1.27021
EIF2A	Eukaryotic Translation Initiation Factor 2A	1.26995
C9orf78	Chromosome 9 Open Reading Frame 78	1.26941
DPH7	Diphthamide Biosynthesis 7	1.26914
ABCA2	ATP Binding Cassette Subfamily A Member 2	1.26861
SLC4A5	Solute Carrier Family 4 Member 5	1.26842
CDRT1	CMT1A Duplicated Region Transcript 1	1.26840
SELENOO	Selenoprotein O	1.26840
MIR518C	MicroRNA 518c	1.26821
RBP7	Retinol Binding Protein 7	1.26793
DDI2	DNA Damage Inducible 1 Homolog 2	1.26793
DGUOK-AS1	DGUOK Antisense RNA 1	1.26779
SLC2A1-AS1	SLC2A1 Antisense RNA 1	1.26779
BANF1P1	BANF1 Pseudogene 1	1.26779
FBXO38	F-Box Protein 38	1.26771
LIN37	Lin-37 DREAM MuvB Core Complex Component	1.26729

SPIDR	Scaffold Protein Involved In DNA Repair	1.26690
MPEG1	Macrophage Expressed 1	1.26658
AFG3L1P	AFG3 Like Matrix AAA Peptidase Subunit 1, Pseudogene	1.26658
NCS1	Neuronal Calcium Sensor 1	1.26633
RGS4	Regulator Of G Protein Signaling 4	1.26601
TMA7	Translation Machinery Associated 7 Homolog	1.26456
STAMBP	STAM Binding Protein	1.26416
ZNF189	Zinc Finger Protein 189	1.26411
LBHD1	LBH Domain Containing 1	1.26362
LINC02474	Long Intergenic Non-Protein Coding RNA 2474	1.26344
LINC01705	Long Intergenic Non-Protein Coding RNA 1705	1.26344
MIR877	MicroRNA 877	1.26309
LINC00461	Long Intergenic Non-Protein Coding RNA 461	1.26295
CA6	Carbonic Anhydrase 6	1.26213
WNK3	WNK Lysine Deficient Protein Kinase 3	1.26210
SLC47A1	Solute Carrier Family 47 Member 1	1.26122
COX7A1	Cytochrome C Oxidase Subunit 7A1	1.26102
C1orf43	Chromosome 1 Open Reading Frame 43	1.26102
TERB2	Telomere Repeat Binding Bouquet Formation Protein 2	1.26073
MALL	Mal, T Cell Differentiation Protein Like	1.26054
CHP1	Calcineurin Like EF-Hand Protein 1	1.26030
TSPOAP1	TSPO Associated Protein 1	1.26002
CA5B	Carbonic Anhydrase 5B	1.25941
KRT6C	Keratin 6C	1.25888
LONP2	Lon Peptidase 2, Peroxisomal	1.25860
WIPI2	WD Repeat Domain, Phosphoinositide Interacting 2	1.25836
CDKL1	Cyclin Dependent Kinase Like 1	1.25806
RILPL1	Rab Interacting Lysosomal Protein Like 1	1.25802
IPO4	Importin 4	1.25798
ZNF543	Zinc Finger Protein 543	1.25790
ZNF433	Zinc Finger Protein 433	1.25790
ABHD17A	Abhydrolase Domain Containing 17A, Depalmitoylase	1.25790
SLC35G5	Solute Carrier Family 35 Member G5	1.25790
ZC3H6	Zinc Finger CCCH-Type Containing 6	1.25782
MAP1S	Microtubule Associated Protein 1S	1.25705
BZW2	Basic Leucine Zipper And W2 Domains 2	1.25645
LINC00917	Long Intergenic Non-Protein Coding RNA 917	1.25609

CATIP-AS1	CATIP Antisense RNA 1	1.25609
H1-10	H1.10 Linker Histone	1.25598
DEFB124	Defensin Beta 124	1.25591
MTFP1	Mitochondrial Fission Process 1	1.25566
PFDN6	Prefoldin Subunit 6	1.25552
CHML	CHM Like Rab Escort Protein	1.25524
CCDC181	Coiled-Coil Domain Containing 181	1.25496
BLOC1S1-RDH	BLOC1S1-RDH5 Readthrough	1.25466
LOXL1-AS1	LOXL1 Antisense RNA 1	1.25437
C1QTNF6	C1q And TNF Related 6	1.25410
SNX33	Sorting Nexin 33	1.25367
OSCP1	Organic Solute Carrier Partner 1	1.25367
ZNF763	Zinc Finger Protein 763	1.25367
RELT	RELT TNF Receptor	1.25356
FADS6	Fatty Acid Desaturase 6	1.25356
RPUSD2	RNA Pseudouridine Synthase Domain Containing 2	1.25356
BOP1	BOP1 Ribosomal Biogenesis Factor	1.25315
GMIP	GEM Interacting Protein	1.25303
ZC3H3	Zinc Finger CCCH-Type Containing 3	1.25303
HRC	Histidine Rich Calcium Binding Protein	1.25267
LINC00632	Long Intergenic Non-Protein Coding RNA 632	1.25260
WASH3P	WASP Family Homolog 3, Pseudogene	1.25234
C1QC	Complement C1q C Chain	1.25146
ASRGL1	Asparaginase And Isoaspartyl Peptidase 1	1.25137
MIR1231	MicroRNA 1231	1.25120
CDC42EP5	CDC42 Effector Protein 5	1.25112
UPB1	Beta-Ureidopropionase 1	1.25104
ISX	Intestine Specific Homeobox	1.25049
TMEM80	Transmembrane Protein 80	1.24990
SMTNL1	Smoothelin Like 1	1.24990
SMG5	SMG5 Nonsense Mediated MRNA Decay Factor	1.24944
FNDC1	Fibronectin Type III Domain Containing 1	1.24861
ELOVL7	ELOVL Fatty Acid Elongase 7	1.24840
PSMD11	Proteasome 26S Subunit, Non-ATPase 11	1.24807
KCND2	Potassium Voltage-Gated Channel Subfamily D Member 2	1.24733
NKX6-1	NK6 Homeobox 1	1.24731
ZNF32	Zinc Finger Protein 32	1.24689
DBNDD2	Dysbindin Domain Containing 2	1.24656
PAOX	Polyamine Oxidase	1.24634
ERMAP	Erythroblast Membrane Associated Protein (Scianna Blood Group)	1.24577

SPDYE12P	Speedy/RINGO Cell Cycle Regulator Family Member E12, Pseudogene	1.24516
KLHL10	Kelch Like Family Member 10	1.24495
SLC4A4	Solute Carrier Family 4 Member 4	1.24491
SORBS3	Sorbin And SH3 Domain Containing 3	1.24484
MTCH2	Mitochondrial Carrier 2	1.24448
PARP3	Poly(ADP-Ribose) Polymerase Family Member 3	1.24417
TAT	Tyrosine Aminotransferase	1.24398
CST4	Cystatin S	1.24392
CENPV	Centromere Protein V	1.24392
POLR3D	RNA Polymerase III Subunit D	1.24376
CALML3-AS1	CALML3 Antisense RNA 1	1.24376
ST6GALNAC4	ST6 N-Acetylgalactosaminide Alpha-2,6-Sialyltransferase 4	1.24369
PACRGL	Parkin Coregulated Like	1.24337
STRN4	Striatin 4	1.24313
CNPY2	Canopy FGF Signaling Regulator 2	1.24313
LILRB3	Leukocyte Immunoglobulin Like Receptor B3	1.24311
LRIF1	Ligand Dependent Nuclear Receptor Interacting Factor 1	1.24291
G0S2	G0/G1 Switch 2	1.24254
ROPN1L	Rhophilin Associated Tail Protein 1 Like	1.24230
EVA1C	Eva-1 Homolog C	1.24219
UBE2H	Ubiquitin Conjugating Enzyme E2 H	1.24208
CEP57L1	Centrosomal Protein 57 Like 1	1.24183
ITPA	Inosine Triphosphatase	1.24181
SLC38A2	Solute Carrier Family 38 Member 2	1.24130
ZNF548	Zinc Finger Protein 548	1.24122
TRIM39-RPP21	TRIM39-RPP21 Readthrough	1.24122
MIR570	MicroRNA 570	1.24102
ZNF333	Zinc Finger Protein 333	1.24096
ZNF256	Zinc Finger Protein 256	1.24096
HOXC-AS1	HOXC Cluster Antisense RNA 1	1.23992
H2BC5	H2B Clustered Histone 5	1.23889
SLC18A3	Solute Carrier Family 18 Member A3	1.23873
LINC01410	Long Intergenic Non-Protein Coding RNA 1410	1.23858
GAREM1	GRB2 Associated Regulator Of MAPK1 Subtype 1	1.23854
ALKBH6	AlkB Homolog 6	1.23838
SLC2A11	Solute Carrier Family 2 Member 11	1.23830
PVRIG	PVR Related Immunoglobulin Domain Containing	1.23818
TMEM245	Transmembrane Protein 245	1.23762
LAMTOR1	Late Endosomal/Lysosomal Adaptor, MAPK And MTOR Activator 1	1.23750

NCAPH2	Non-SMC Condensin II Complex Subunit H2	1.23722
SIGLEC15	Sialic Acid Binding Ig Like Lectin 15	1.23720
RNF25	Ring Finger Protein 25	1.23718
YJEFN3	YjeF N-Terminal Domain Containing 3	1.23718
KIFC2	Kinesin Family Member C2	1.23670
SIVA1	SIVA1 Apoptosis Inducing Factor	1.23668
CYP4F11	Cytochrome P450 Family 4 Subfamily F Member 11	1.23626
TLK1	Tousled Like Kinase 1	1.23601
RTF1	RTF1 Homolog, Paf1/RNA Polymerase II Complex Component	1.23576
SNPH	Syntaphilin	1.23576
TIMM9	Translocase Of Inner Mitochondrial Membrane 9	1.23536
JMY	Junction Mediating And Regulatory Protein, P53 Cofactor	1.23449
SERPINA10	Serpin Family A Member 10	1.23435
FOXS1	Forkhead Box S1	1.23418
GRIN3B	Glutamate Ionotropic Receptor NMDA Type Subunit 3B	1.23381
MUC22	Mucin 22	1.23332
MTPAP	Mitochondrial Poly(A) Polymerase	1.23266
LRP10	LDL Receptor Related Protein 10	1.23207
SPRED3	Sprouty Related EVH1 Domain Containing 3	1.23113
RETSAT	Retinol Saturase	1.23053
PLVAP	Plasmalemma Vesicle Associated Protein	1.23039
CCNI	Cyclin I	1.23016
HOXD9	Homeobox D9	1.23006
GLYCTK	Glycerate Kinase	1.22971
MAN2B2	Mannosidase Alpha Class 2B Member 2	1.22886
FAM114A2	Family With Sequence Similarity 114 Member A2	1.22886
ANKRD50	Ankyrin Repeat Domain 50	1.22871
BEST4	Bestrophin 4	1.22858
ENDOU	Endonuclease, Poly(U) Specific	1.22803
S1PR5	Sphingosine-1-Phosphate Receptor 5	1.22767
ZNF16	Zinc Finger Protein 16	1.22767
PDXDC2P-NPI	Nuclear Pore Complex-Interacting Protein	1.22752
AGBL3	AGBL Carboxypeptidase 3	1.22746
RCOR3	REST Corepressor 3	1.22725
DCAF10	DDB1 And CUL4 Associated Factor 10	1.22725
BHLHE41	Basic Helix-Loop-Helix Family Member E41	1.22725
DECR1	2,4-Dienoyl-CoA Reductase 1	1.22702
MIR5684	MicroRNA 5684	1.22672
MORF4L2	Mortality Factor 4 Like 2	1.22651

PLA2G2D	Phospholipase A2 Group IID	1.22577
DCP1A	Decapping MRNA 1A	1.22554
HAGH	Hydroxyacylglutathione Hydrolase	1.22467
ZADH2	Zinc Binding Alcohol Dehydrogenase Domain Containing 2	1.22415
PILRB	Paired Immunoglobin Like Type 2 Receptor Beta	1.22415
GTF3C3	General Transcription Factor IIIC Subunit 3	1.22401
MRGPRX1	MAS Related GPR Family Member X1	1.22352
SGPP1	Sphingosine-1-Phosphate Phosphatase 1	1.22303
CELF6	CUGBP Elav-Like Family Member 6	1.22300
SSH2	Slingshot Protein Phosphatase 2	1.22294
PPM1M	Protein Phosphatase, Mg2+/Mn2+ Dependent 1M	1.22250
DNPEP	Aspartyl Aminopeptidase	1.22221
TMEM88	Transmembrane Protein 88	1.22221
DEFB116	Defensin Beta 116	1.22151
MGAT4B	Alpha-1,3-Mannosyl-Glycoprotein 4-Beta-N-Acetylglucosaminyltransferase B	1.22083
GLI4	GLI Family Zinc Finger 4	1.22075
PCK1	Phosphoenolpyruvate Carboxykinase 1	1.22060
PSMD13	Proteasome 26S Subunit, Non-ATPase 13	1.22053
SNORA66	Small Nucleolar RNA, H/ACA Box 66	1.22038
HAPLN2	Hyaluronan And Proteoglycan Link Protein 2	1.22013
CPT1C	Carnitine Palmitoyltransferase 1C	1.21925
SACM1L	SAC1 Like Phosphatidylinositide Phosphatase	1.21853
ZNF816	Zinc Finger Protein 816	1.21824
SH2D6	SH2 Domain Containing 6	1.21824
SRRM5	Serine/Arginine Repetitive Matrix 5	1.21824
SCARNA21	Small Cajal Body-Specific RNA 21	1.21824
MYRF-AS1	MYRF Antisense RNA 1	1.21824
LINC01271	Long Intergenic Non-Protein Coding RNA 1271	1.21824
URAHP	Urate (Hydroxyiso-) Hydrolase, Pseudogene	1.21824
ZNF285B	Zinc Finger Protein 285B (Pseudogene)	1.21824
ENSG00000255197		1.21824
ENSG00000262089		1.21824
ENSG00000268601		1.21824
ENSG00000234139		1.21824
RPL35P2	Ribosomal Protein L35 Pseudogene 2	1.21824
ENSG00000272374		1.21824
ENSG00000267682		1.21824
RNU6-850P	RNA, U6 Small Nuclear 850, Pseudogene	1.21824

NRBF2P5	Nuclear Receptor Binding Factor 2 Pseudogene 5	1.21824
lnc-KCNN4-1		1.21824
lnc-CEBPB-9		1.21824
HSALNG0111457-001		1.21824
HSALNG0111457-002		1.21824
piR-39348		1.21824
RF00017-6678		1.21824
MN309188		1.21824
piR-43099-059		1.21824
SNODB1980		1.21824
HSALNG0084553		1.21824
CSGALNACT2	Chondroitin Sulfate N-Acetylgalactosaminyltransferase 2	1.21787
SARAF	Store-Operated Calcium Entry Associated Regulatory Factor	1.21776
ZNF418	Zinc Finger Protein 418	1.21719
NPM3	Nucleophosmin/Nucleoplasmin 3	1.21718
OAZ2	Ornithine Decarboxylase Antizyme 2	1.21688
SCRN2	Secernin 2	1.21688
CXorf38	Chromosome X Open Reading Frame 38	1.21688
C12orf43	Chromosome 12 Open Reading Frame 43	1.21660
C1D	C1D Nuclear Receptor Corepressor	1.21649
ADPGK	ADP Dependent Glucokinase	1.21631
MFRP	Membrane Frizzled-Related Protein	1.21612
RRP1	Ribosomal RNA Processing 1	1.21605
SNX3	Sorting Nexin 3	1.21573
HCN3	Hyperpolarization Activated Cyclic Nucleotide Gated Potassium Channel 3	1.21498
SSC4D	Scavenger Receptor Cysteine Rich Family Member With 4 Domains	1.21481
TGOLN2	Trans-Golgi Network Protein 2	1.21480
C8G	Complement C8 Gamma Chain	1.21477
POC1B	POC1 Centriolar Protein B	1.21422
INO80B	INO80 Complex Subunit B	1.21420
OPHN1	Oligophrenin 1	1.21409
GPR39	G Protein-Coupled Receptor 39	1.21362
GHRLOS	Ghrelin Opposite Strand/Antisense RNA	1.21361
STK16	Serine/Threonine Kinase 16	1.21324
ZNF566	Zinc Finger Protein 566	1.21300
DNAI4	Dynein Axonemal Intermediate Chain 4	1.21297
PITPNC1	Phosphatidylinositol Transfer Protein Cytoplasmic 1	1.21259
POLR3GL	RNA Polymerase III Subunit GL	1.21215
MOAP1	Modulator Of Apoptosis 1	1.21146
RPL39P5	Ribosomal Protein L39 Pseudogene 5	1.21135

PTPRQ	Protein Tyrosine Phosphatase Receptor Type Q	1.21075
ZNF322	Zinc Finger Protein 322	1.21017
WASH2P	WASP Family Homolog 2, Pseudogene	1.21015
APOBEC2	Apolipoprotein B MRNA Editing Enzyme Catalytic Subunit 2	1.20994
CEACAM21	CEA Cell Adhesion Molecule 21	1.20968
PIANP	PILR Alpha Associated Neural Protein	1.20964
THBS4-AS1	THBS4 Antisense RNA 1	1.20964
DTWD2	DTW Domain Containing 2	1.20964
UHRF1BP1	UHRF1 Binding Protein 1	1.20961
HSDL1	Hydroxysteroid Dehydrogenase Like 1	1.20961
SNAI3	Snail Family Transcriptional Repressor 3	1.20942
MRPL13	Mitochondrial Ribosomal Protein L13	1.20922
NAE1	NEDD8 Activating Enzyme E1 Subunit 1	1.20878
CENPO	Centromere Protein O	1.20867
ZBTB22	Zinc Finger And BTB Domain Containing 22	1.20864
DISP2	Dispatched RND Transporter Family Member 2	1.20855
KCTD15	Potassium Channel Tetramerization Domain Containing 15	1.20851
TTC6	Tetratricopeptide Repeat Domain 6	1.20830
NFE2L1	Nuclear Factor, Erythroid 2 Like 1	1.20802
CAVIN2	Caveolae Associated Protein 2	1.20656
ADGRE2	Adhesion G Protein-Coupled Receptor E2	1.20649
TP53I11	Tumor Protein P53 Inducible Protein 11	1.20647
ZACN	Zinc Activated Ion Channel	1.20560
REG3G	Regenerating Family Member 3 Gamma	1.20529
ENSG00000260022		1.20496
ENSG00000201207		1.20496
MG828730-053		1.20496
CORT	Cortistatin	1.20487
CRELD2	Cysteine Rich With EGF Like Domains 2	1.20449
RBPMS2	RNA Binding Protein, MRNA Processing Factor 2	1.20445
LCA5L	Lebercilin LCA5 Like	1.20445
GSG1L	GSG1 Like	1.20445
RPP25L	Ribonuclease P/MRP Subunit P25 Like	1.20445
MFSD10	Major Facilitator Superfamily Domain Containing 10	1.20409
KIFBP	Kinesin Family Binding Protein	1.20400
GALR2	Galanin Receptor 2	1.20335
TMEM147	Transmembrane Protein 147	1.20297
MIR663B	MicroRNA 663b	1.20291

CHRM5	Cholinergic Receptor Muscarinic 5	1.20271
LSM3	LSM3 Homolog, U6 Small Nuclear RNA And MRNA Degradation Associated	1.20248
KNSTRN	Kinetochore Localized Astrin (SPAG5) Binding Protein	1.20232
AP1AR	Adaptor Related Protein Complex 1 Associated Regulatory Protein	1.20211
ZNF317	Zinc Finger Protein 317	1.20201
HHIPL1	HHIP Like 1	1.20201
HSH2D	Hematopoietic SH2 Domain Containing	1.20201
FAM106A	Family With Sequence Similarity 106 Member A	1.20201
HORMAD2-AS	HORMAD2 And MTMR3 Antisense RNA 1	1.20201
CRIP1	Cysteine Rich Protein 1	1.20194
PRLHR	Prolactin Releasing Hormone Receptor	1.20190
MGME1	Mitochondrial Genome Maintenance Exonuclease 1	1.20180
PDE4DIP	Phosphodiesterase 4D Interacting Protein	1.20165
LINC01485	Long Intergenic Non-Protein Coding RNA 1485	1.20150
KLF15	Kruppel Like Factor 15	1.20130
DAGLA	Diacylglycerol Lipase Alpha	1.20111
MIR3191	MicroRNA 3191	1.20097
FBXL15	F-Box And Leucine Rich Repeat Protein 15	1.20076
MYO1F	Myosin IF	1.20021
SLN	Sarcolipin	1.20019
CHRAC1	Chromatin Accessibility Complex Subunit 1	1.19884
OR10AD1	Olfactory Receptor Family 10 Subfamily AD Member 1	1.19860
MIR3619	MicroRNA 3619	1.19849
NAA50	N-Alpha-Acetyltransferase 50, NatE Catalytic Subunit	1.19719
ACBD5	Acyl-CoA Binding Domain Containing 5	1.19694
PAN3	Poly(A) Specific Ribonuclease Subunit PAN3	1.19692
TBC1D16	TBC1 Domain Family Member 16	1.19692
USPL1	Ubiquitin Specific Peptidase Like 1	1.19692
MIR5188	MicroRNA 5188	1.19686
AP1G2-AS1	AP1G2 Antisense RNA 1	1.19682
ZNF280C	Zinc Finger Protein 280C	1.19550
MIR933	MicroRNA 933	1.19535
FDX2	Ferredoxin 2	1.19441

RRP12	Ribosomal RNA Processing 12 Homolog	1.19421
SLC23A3	Solute Carrier Family 23 Member 3	1.19397
ELF2	E74 Like ETS Transcription Factor 2	1.19369
CCDC66	Coiled-Coil Domain Containing 66	1.19310
VWA1	Von Willebrand Factor A Domain Containing 1	1.19295
TMCO6	Transmembrane And Coiled-Coil Domains 6	1.19283
PKIA	CAMP-Dependent Protein Kinase Inhibitor Alpha	1.19268
ZC3H14	Zinc Finger CCCH-Type Containing 14	1.19251
NOX5	NADPH Oxidase 5	1.19186
CLEC11A	C-Type Lectin Domain Containing 11A	1.19171
SYDE1	Synapse Defective Rho GTPase Homolog 1	1.19101
DCHS2	Dachsous Cadherin-Related 2	1.19069
TMEM138	Transmembrane Protein 138	1.19056
ZFAND2B	Zinc Finger AN1-Type Containing 2B	1.19039
MRPL34	Mitochondrial Ribosomal Protein L34	1.19028
MIR587	MicroRNA 587	1.18996
POLR1B	RNA Polymerase I Subunit B	1.18952
CATSPERD	Cation Channel Sperm Associated Auxiliary Subunit Delta	1.18939
SNHG18	Small Nucleolar RNA Host Gene 18	1.18912
CNPY3	Canopy FGF Signaling Regulator 3	1.18826
LINC01317	Long Intergenic Non-Protein Coding RNA 1317	1.18781
LENG9	Leukocyte Receptor Cluster Member 9	1.18777
CHPF2	Chondroitin Polymerizing Factor 2	1.18604
GMPR	Guanosine Monophosphate Reductase	1.18601
FUNDC1	FUN14 Domain Containing 1	1.18594
TAMM41	TAM41 Mitochondrial Translocator Assembly And Maintenance Homolog	1.18594
MRPL37	Mitochondrial Ribosomal Protein L37	1.18577
ATP5MF	ATP Synthase Membrane Subunit F	1.18548
CSPG5	Chondroitin Sulfate Proteoglycan 5	1.18533
OR4X1	Olfactory Receptor Family 4 Subfamily X Member 1	1.18533
SH3D21	SH3 Domain Containing 21	1.18533
A3GALT2	Alpha 1,3-Galactosyltransferase 2	1.18533
MBLAC1	Metallo-Beta-Lactamase Domain Containing 1	1.18533
MASCRNA	MALAT1-Associated Small Cytoplasmic RNA	1.18533
LINC01816	Long Intergenic Non-Protein Coding RNA 1816	1.18513
LINC02201	Long Intergenic Non-Protein Coding RNA 2201	1.18513

LINC02573	Long Intergenic Non-Protein Coding RNA 2573	1.18513
LINC02264	Long Intergenic Non-Protein Coding RNA 2264	1.18513
MOB3A	MOB Kinase Activator 3A	1.18493
CCDC182	Coiled-Coil Domain Containing 182	1.18493
TAF1L	TATA-Box Binding Protein Associated Factor 1 Like	1.18490
SMPD4	Sphingomyelin Phosphodiesterase 4	1.18474
RABGAP1	RAB GTPase Activating Protein 1	1.18472
YME1L1	YME1 Like 1 ATPase	1.18424
JAM3	Junctional Adhesion Molecule 3	1.18374
CLEC4G	C-Type Lectin Domain Family 4 Member G	1.18373
FBXW12	F-Box And WD Repeat Domain Containing 12	1.18365
ZNF57	Zinc Finger Protein 57	1.18364
STMN2	Stathmin 2	1.18341
MIR520F	MicroRNA 520f	1.18337
BCAN	Brevican	1.18260
ZNF440	Zinc Finger Protein 440	1.18260
PROCA1	Protein Interacting With Cyclin A1	1.18260
LOC10896116	POU5F1 5' Regulatory Region	1.18152
MIR942	MicroRNA 942	1.18125
GPR107	G Protein-Coupled Receptor 107	1.18062
FAM162A	Family With Sequence Similarity 162 Member A	1.18012
ZNF841	Zinc Finger Protein 841	1.17949
FHIP2B	FHF Complex Subunit HOOK Interacting Protein 2B	1.17907
MT-TG	Mitochondrially Encoded TRNA-Gly (GGN)	1.17892
CER1	Cerberus 1, DAN Family BMP Antagonist	1.17843
APOBEC3C	Apolipoprotein B MRNA Editing Enzyme Catalytic Subunit 3C	1.17837
DBP	D-Box Binding PAR BZIP Transcription Factor	1.17768
KEL	Kell Metallo-Endopeptidase (Kell Blood Group)	1.17761
EAF2	ELL Associated Factor 2	1.17744
MIR664A	MicroRNA 664a	1.17743
MIR592	MicroRNA 592	1.17707
ZBTB24	Zinc Finger And BTB Domain Containing 24	1.17683
SYS1	SYS1 Golgi Trafficking Protein	1.17637
DRAP1	DR1 Associated Protein 1	1.17634
C2orf42	Chromosome 2 Open Reading Frame 42	1.17613
HAL	Histidine Ammonia-Lyase	1.17586

MYOSLID	Myocardin-Induced Smooth Muscle LncRNA, Inducer Of Differentiation	1.17495
RAVER2	Ribonucleoprotein, PTB Binding 2	1.17444
MEF2B	Myocyte Enhancer Factor 2B	1.17371
DNAJC25	DnaJ Heat Shock Protein Family (Hsp40) Member C25	1.17302
LINC00242	Long Intergenic Non-Protein Coding RNA 242	1.17202
TIMM29	Translocase Of Inner Mitochondrial Membrane 29	1.17165
WDR35	WD Repeat Domain 35	1.17114
OSTM1	Osteoclastogenesis Associated Transmembrane Protein 1	1.17097
MARCHF6	Membrane Associated Ring-CH-Type Finger 6	1.17097
MIR3193	MicroRNA 3193	1.17043
IFT20	Intraflagellar Transport 20	1.17034
TEPP	Testis, Prostate And Placenta Expressed	1.17031
AK6	Adenylate Kinase 6	1.16961
CDC123	Cell Division Cycle 123	1.16949
CAB39L	Calcium Binding Protein 39 Like	1.16916
PALM	Paralemmin	1.16767
TIGD5	Tigger Transposable Element Derived 5	1.16683
MYG1-AS1	MYG1 Antisense RNA 1	1.16683
IZUMO1	Izumo Sperm-Egg Fusion 1	1.16669
SNORD48	Small Nucleolar RNA, C/D Box 48	1.16669
TMEM143	Transmembrane Protein 143	1.16611
SBNO2	Strawberry Notch Homolog 2	1.16611
CYB5D1	Cytochrome B5 Domain Containing 1	1.16611
PRXL2B	Peroxiredoxin Like 2B	1.16611
ARAP1-AS1	ARAP1 Antisense RNA 1	1.16563
THORLNC	Testis Associated Oncogenic LncRNA	1.16441
TMEM199	Transmembrane Protein 199	1.16396
HASPIN	Histone H3 Associated Protein Kinase	1.16271
FGD4	FYVE, RhoGEF And PH Domain Containing 4	1.16265
FAM189A2	Family With Sequence Similarity 189 Member A2	1.16188
ASIC2	Acid Sensing Ion Channel Subunit 2	1.16162
ANKZF1	Ankyrin Repeat And Zinc Finger Peptidyl TRNA Hydrolase 1	1.16133
ZNF814	Zinc Finger Protein 814	1.16110
CA10	Carbonic Anhydrase 10	1.16055
CISD1	CDGSH Iron Sulfur Domain 1	1.16011
CIDEC	Cell Death Inducing DFFA Like Effector C	1.16003
PTGFR	Prostaglandin F Receptor	1.15967
NCR3LG1	Natural Killer Cell Cytotoxicity Receptor 3 Ligand 1	1.15961

SCARA3	Scavenger Receptor Class A Member 3	1.15946
ZNF665	Zinc Finger Protein 665	1.15906
DNAJC3	DnaJ Heat Shock Protein Family (Hsp40) Member C3	1.15885
VPS50	VPS50 Subunit Of EARP/GARPII Complex	1.15859
IL36B	Interleukin 36 Beta	1.15828
ELOVL2	ELOVL Fatty Acid Elongase 2	1.15743
RWDD4	RWD Domain Containing 4	1.15742
NPDC1	Neural Proliferation, Differentiation And Control 1	1.15660
SPINK4	Serine Peptidase Inhibitor Kazal Type 4	1.15660
ZNF707	Zinc Finger Protein 707	1.15604
C9orf24	Chromosome 9 Open Reading Frame 24	1.15604
C9orf50	Chromosome 9 Open Reading Frame 50	1.15604
MIR4714	MicroRNA 4714	1.15604
IWS1	Interacts With SUPT6H, CTD Assembly Factor 1	1.15537
ATP6AP1	ATPase H+ Transporting Accessory Protein 1	1.15532
RUSC1	RUN And SH3 Domain Containing 1	1.15503
GMPS	Guanine Monophosphate Synthase	1.15448
SLC35B4	Solute Carrier Family 35 Member B4	1.15409
ROBO3	Roundabout Guidance Receptor 3	1.15307
MAP7D3	MAP7 Domain Containing 3	1.15255
FBXW5	F-Box And WD Repeat Domain Containing 5	1.15239
RABL2A	RAB, Member Of RAS Oncogene Family Like 2A	1.15199
SCGN	Secretagogin, EF-Hand Calcium Binding Protein	1.15163
MED25	Mediator Complex Subunit 25	1.15117
NR2C2AP	Nuclear Receptor 2C2 Associated Protein	1.15110
APBA2	Amyloid Beta Precursor Protein Binding Family A Member 2	1.15106
KLHL17	Kelch Like Family Member 17	1.15052
NFASC	Neurofascin	1.15017
METTL26	Methyltransferase Like 26	1.15013
ZFYVE28	Zinc Finger FYVE-Type Containing 28	1.15002
SLC43A3	Solute Carrier Family 43 Member 3	1.14956
ZNF823	Zinc Finger Protein 823	1.14751
SLC38A11	Solute Carrier Family 38 Member 11	1.14751
GRAMD2A	GRAM Domain Containing 2A	1.14751
C20orf203	Chromosome 20 Open Reading Frame 203	1.14751
ENSG00000203325		1.14751
SERINC2	Serine Incorporator 2	1.14687

WSB2	WD Repeat And SOCS Box Containing 2	1.14687
TFAP2E	Transcription Factor AP-2 Epsilon	1.14645
PUS3	Pseudouridine Synthase 3	1.14637
ZNF628	Zinc Finger Protein 628	1.14608
SLC35G6	Solute Carrier Family 35 Member G6	1.14582
HNRNPA3P1	Heterogeneous Nuclear Ribonucleoprotein A3 Pseudogene 1	1.14582
GRIK5	Glutamate Ionotropic Receptor Kainate Type Subunit 5	1.14533
HMGN4	High Mobility Group Nucleosomal Binding Domain 4	1.14527
PSMG2	Proteasome Assembly Chaperone 2	1.14471
PLEKHM2	Pleckstrin Homology And RUN Domain Containing M2	1.14467
SYT13	Synaptotagmin 13	1.14432
EPN2	Epsin 2	1.14416
LINC00662	Long Intergenic Non-Protein Coding RNA 662	1.14376
XPNPEP1	X-Prolyl Aminopeptidase 1	1.14347
CHPF	Chondroitin Polymerizing Factor	1.14334
BTBD18	BTB Domain Containing 18	1.14325
RPL23AP32	Ribosomal Protein L23a Pseudogene 32	1.14325
EIF2AK1	Eukaryotic Translation Initiation Factor 2 Alpha Kinase 1	1.14305
ATP6V0A4	ATPase H+ Transporting V0 Subunit A4	1.14297
DLG4	Discs Large MAGUK Scaffold Protein 4	1.14136
DAAM2	Dishevelled Associated Activator Of Morphogenesis 2	1.14066
LINC01173	Long Intergenic Non-Protein Coding RNA 1173	1.14021
ENSG00000260661		1.14021
lnc-OARD1-1		1.14021
lnc-MUC1-2		1.14021
lnc-ABO-33		1.14021
lnc-MUC1-1		1.14021
HSALNG0007476		1.14021
HSALNG0007478		1.14021
HSALNG0007480		1.14021
lnc-THBS3-2		1.14021
MIR589	MicroRNA 589	1.13978
LINC00337	Long Intergenic Non-Protein Coding RNA 337	1.13921
STK35	Serine/Threonine Kinase 35	1.13897
MIR1204	MicroRNA 1204	1.13874
MSANTD2	Myb/SANT DNA Binding Domain Containing 2	1.13873

AWAT2	Acyl-CoA Wax Alcohol Acyltransferase 2	1.13865
ABCA13	ATP Binding Cassette Subfamily A Member 13	1.13830
CDRT4	CMT1A Duplicated Region Transcript 4	1.13830
SMIM5	Small Integral Membrane Protein 5	1.13830
CTD-3080P12.:	Uncharacterized LOC101928857	1.13830
PMS2P2	PMS1 Homolog 2, Mismatch Repair System Component Pseudogene 2	1.13827
POTEKP	POTE Ankyrin Domain Family Member K, Pseudogene	1.13818
TSPYL5	TSPY Like 5	1.13796
SOX15	SRY-Box Transcription Factor 15	1.13780
DHX15	DEAH-Box Helicase 15	1.13707
PPME1	Protein Phosphatase Methylesterase 1	1.13696
LEPROT	Leptin Receptor Overlapping Transcript	1.13554
NECAP1	NECAP Endocytosis Associated 1	1.13527
MIR1275	MicroRNA 1275	1.13436
TMEM160	Transmembrane Protein 160	1.13407
LINC00477	Long Intergenic Non-Protein Coding RNA 477	1.13382
C17orf49	Chromosome 17 Open Reading Frame 49	1.13316
LSS	Lanosterol Synthase	1.13287
ZNF83	Zinc Finger Protein 83	1.13276
ZNF808	Zinc Finger Protein 808	1.13276
SLC22A31	Solute Carrier Family 22 Member 31	1.13276
ENSG00000250173		1.13276
ENSG00000226647		1.13276
STK19B	Serine/Threonine Kinase 19B (Pseudogene)	1.13276
RNY4P10	RNY4 Pseudogene 10	1.13276
ENSG00000263508		1.13276
ENSG00000258302		1.13276
ENSG00000266114		1.13276
ENSG00000261888		1.13276
ENSG00000232909		1.13276
ENSG00000222529		1.13276
ENSG00000271581		1.13276
ENSG00000225507		1.13276
ENSG00000245869		1.13276
ENSG00000255836		1.13276
ENSG00000232615		1.13276
lnc-TPCN2-5		1.13276
lnc-BCL3-5		1.13276
lnc-FAM109A-2		1.13276
lnc-IFT46-2		1.13276
lnc-DEGS2-7		1.13276
ENSG00000239392		1.13276

piR-47976		1.13276
piR-37687		1.13276
piR-43164-042		1.13276
piR-47961		1.13276
HSALNG0130801		1.13276
piR-59928-060		1.13276
LOC10536962	Uncharacterized LOC105369625	1.13276
HSALNG0106718		1.13276
SEZ6	Seizure Related 6 Homolog	1.13268
XKR8	XK Related 8	1.13233
MAP3K10	Mitogen-Activated Protein Kinase Kinase Kinase 10	1.13204
SERPINA12	Serpin Family A Member 12	1.13189
LINC02871	Long Intergenic Non-Protein Coding RNA 2871	1.13183
AP1M2	Adaptor Related Protein Complex 1 Subunit Mu 2	1.13165
MSBP2	Minisatellite Binding Protein 2	1.13137
C17orf107	Chromosome 17 Open Reading Frame 107	1.13133
ADAMTSL4-A	ADAMTSL4 Antisense RNA 2	1.13133
MIR6886	MicroRNA 6886	1.13133
SPATA5L1	Spermatogenesis Associated 5 Like 1	1.13070
ABT1	Activator Of Basal Transcription 1	1.13022
CDCA4	Cell Division Cycle Associated 4	1.13020
SH2D2A	SH2 Domain Containing 2A	1.12985
NUP58	Nucleoporin 58	1.12909
MIR34AHG	MIR34A Host Gene	1.12904
RABEP2	Rabaptin, RAB GTPase Binding Effector Protein 2	1.12892
BSN	Bassoon Presynaptic Cytomatrix Protein	1.12852
BLOC1S4	Biogenesis Of Lysosomal Organelles Complex 1 Subunit 4	1.12843
OR4D2	Olfactory Receptor Family 4 Subfamily D Member 2	1.12812
MIR939	MicroRNA 939	1.12768
MIR593	MicroRNA 593	1.12768
ZPBP2	Zona Pellucida Binding Protein 2	1.12767
IKZF4	IKAROS Family Zinc Finger 4	1.12737
TENT2	Terminal Nucleotidyltransferase 2	1.12732
SPTSSA	Serine Palmitoyltransferase Small Subunit A	1.12667
COQ5	Coenzyme Q5, Methyltransferase	1.12657
POLR3H	RNA Polymerase III Subunit H	1.12657
AP3S2	Adaptor Related Protein Complex 3 Subunit Sigma 2	1.12657
ZSCAN20	Zinc Finger And SCAN Domain Containing 20	1.12657
CMTR2	Cap Methyltransferase 2	1.12657

LRCOL1	Leucine Rich Colipase Like 1	1.12657
RPS3AP26	RPS3A Pseudogene 26	1.12657
ACOT7	Acyl-CoA Thioesterase 7	1.12648
ZBED4	Zinc Finger BED-Type Containing 4	1.12612
CAMTA2	Calmodulin Binding Transcription Activator 2	1.12587
RPS27AP2	RPS27A Pseudogene 2	1.12572
CPDP1	Carboxypeptidase D Pseudogene 1	1.12572
HMG20B	High Mobility Group 20B	1.12507
PCDH9	Protocadherin 9	1.12464
MRPL41	Mitochondrial Ribosomal Protein L41	1.12380
FAM66A	Family With Sequence Similarity 66 Member A	1.12335
SNED1	Sushi, Nidogen And EGF Like Domains 1	1.12322
SF3B5	Splicing Factor 3b Subunit 5	1.12131
DBF4B	DBF4 Zinc Finger B	1.12073
NSUN7	NOP2/Sun RNA Methyltransferase Family Member 7	1.12056
FBXO2	F-Box Protein 2	1.12040
STRN3	Striatin 3	1.12036
MIR1284	MicroRNA 1284	1.11945
WSCD2	WSC Domain Containing 2	1.11944
PODXL2	Podocalyxin Like 2	1.11897
ZBTB5	Zinc Finger And BTB Domain Containing 5	1.11897
MYLIP	Myosin Regulatory Light Chain Interacting Protein	1.11890
SFSWAP	Splicing Factor SWAP	1.11890
C8orf76	Chromosome 8 Open Reading Frame 76	1.11872
ZDHHC6	Zinc Finger DHHC-Type Palmitoyltransferase 6	1.11862
RAB26	RAB26, Member RAS Oncogene Family	1.11829
MED6	Mediator Complex Subunit 6	1.11806
COG5	Component Of Oligomeric Golgi Complex 5	1.11790
HSPB7	Heat Shock Protein Family B (Small) Member 7	1.11763
MIR598	MicroRNA 598	1.11757
IGK	Immunoglobulin Kappa Locus	1.11743
AKR1D1	Aldo-Keto Reductase Family 1 Member D1	1.11724
ANKFY1	Ankyrin Repeat And FYVE Domain Containing 1	1.11717
NLRC4	NLR Family CARD Domain Containing 4	1.11630
INSIG2	Insulin Induced Gene 2	1.11628
ECH1	Enoyl-CoA Hydratase 1	1.11578

PCDHA2	Protocadherin Alpha 2	1.11539
CSNK1G1	Casein Kinase 1 Gamma 1	1.11499
SCCPDH	Saccharopine Dehydrogenase (Putative)	1.11443
CCDC59	Coiled-Coil Domain Containing 59	1.11443
ZC3H10	Zinc Finger CCCH-Type Containing 10	1.11421
FBXL20	F-Box And Leucine Rich Repeat Protein 20	1.11361
ARHGAP9	Rho GTPase Activating Protein 9	1.11267
MRPL21	Mitochondrial Ribosomal Protein L21	1.11267
ZCCHC7	Zinc Finger CCHC-Type Containing 7	1.11267
PDXP	Pyridoxal Phosphatase	1.11243
FAM111A	FAM111 Trypsin Like Peptidase A	1.11190
FOLR3	Folate Receptor Gamma	1.11160
AANAT	Aralkylamine N-Acetyltransferase	1.11097
RPAIN	RPA Interacting Protein	1.11066
ZNF24	Zinc Finger Protein 24	1.11022
C14orf28	Chromosome 14 Open Reading Frame 28	1.10874
IFNL1	Interferon Lambda 1	1.10836
TMEM30B	Transmembrane Protein 30B	1.10773
LOC10825468[illegible]	PAX5 Promoter Region	1.10707
TBCC	Tubulin Folding Cofactor C	1.10702
DDRGK1	DDRGK Domain Containing 1	1.10695
LRRC37A3	Leucine Rich Repeat Containing 37 Member A3	1.10638
TIMM8A	Translocase Of Inner Mitochondrial Membrane 8A	1.10610
DGCR6L	DiGeorge Syndrome Critical Region Gene 6 Like	1.10589
MEF2C-AS1	MEF2C Antisense RNA 1	1.10583
NCOA7-AS1	NCOA7 Antisense RNA 1	1.10583
PHGR1	Proline, Histidine And Glycine Rich 1	1.10583
EXOC3	Exocyst Complex Component 3	1.10553
LGALS12	Galectin 12	1.10538
HERC1	HECT And RLD Domain Containing E3 Ubiquitin Protein Ligase Family Member 1	1.10532
ZSWIM5	Zinc Finger SWIM-Type Containing 5	1.10532
C1orf159	Chromosome 1 Open Reading Frame 159	1.10532
MYBPC1	Myosin Binding Protein C1	1.10524
GPR137C	G Protein-Coupled Receptor 137C	1.10521
IGES	Immunoglobulin E Concentration, Serum	1.10505
ADSS2	Adenylosuccinate Synthase 2	1.10454
TEKT2	Tektin 2	1.10382
DNAJC14	DnaJ Heat Shock Protein Family (Hsp40) Member C14	1.10382

PAM	Peptidylglycine Alpha-Amidating Monooxygenase	1.10370
PTPN18	Protein Tyrosine Phosphatase Non-Receptor Type 18	1.10190
AJM1	Apical Junction Component 1 Homolog	1.10164
TBC1D13	TBC1 Domain Family Member 13	1.10091
RAB44	RAB44, Member RAS Oncogene Family	1.10091
C5AR2	Complement Component 5a Receptor 2	1.10052
KPTN	Kaptin, Actin Binding Protein	1.10032
GPX7	Glutathione Peroxidase 7	1.09973
B3GALT6	Beta-1,3-Galactosyltransferase 6	1.09925
TBCB	Tubulin Folding Cofactor B	1.09924
ATL3	Atlastin GTPase 3	1.09911
MIR4317	MicroRNA 4317	1.09897
RPRML	Reprimo Like	1.09883
METTL18	Methyltransferase Like 18	1.09848
TRPM6	Transient Receptor Potential Cation Channel Subfamily M Member 6	1.09831
SEC24A	SEC24 Homolog A, COPII Coat Complex Component	1.09817
PUSL1	Pseudouridine Synthase Like 1	1.09817
TAT-AS1	TAT Antisense RNA 1	1.09777
ZBED5	Zinc Finger BED-Type Containing 5	1.09742
NCOA5	Nuclear Receptor Coactivator 5	1.09720
FCER1A	Fc Fragment Of IgE Receptor Ia	1.09599
SLC2A14	Solute Carrier Family 2 Member 14	1.09586
BRK1	BRICK1 Subunit Of SCAR/WAVE Actin Nucleating Complex	1.09555
CAAP1	Caspase Activity And Apoptosis Inhibitor 1	1.09537
TOX4	TOX High Mobility Group Box Family Member 4	1.09487
TIGIT	T Cell Immunoreceptor With Ig And ITIM Domains	1.09457
PARP12	Poly(ADP-Ribose) Polymerase Family Member 12	1.09420
MEGF6	Multiple EGF Like Domains 6	1.09420
SLC15A3	Solute Carrier Family 15 Member 3	1.09392
UBXN8	UBX Domain Protein 8	1.09392
MIR3936	MicroRNA 3936	1.09392
MIR3591	MicroRNA 3591	1.09392
MIR4668	MicroRNA 4668	1.09392
LINC02288	Long Intergenic Non-Protein Coding RNA 2288	1.09392
CTRB2	Chymotrypsinogen B2	1.09359
NDC1	NDC1 Transmembrane Nucleoporin	1.09337
KCNIP2	Potassium Voltage-Gated Channel Interacting Protein 2	1.09332

MYH2	Myosin Heavy Chain 2	1.09307
INKA1	Inka Box Actin Regulator 1	1.09303
TRS-AGA2-6	TRNA-Ser (Anticodon AGA) 2-6	1.09206
TRI-AAT4-1	TRNA-Ile (Anticodon AAT) 4-1	1.09206
TRT-AGT1-2	TRNA-Thr (Anticodon AGT) 1-2	1.09206
PDRG1	P53 And DNA Damage Regulated 1	1.09175
DESI2	Desumoylating Isopeptidase 2	1.09140
DDX51	DEAD-Box Helicase 51	1.09089
KIR2DS4	Killer Cell Immunoglobulin Like Receptor, Two Ig Domains And Short Cytoplasmic Tail 4	1.09046
SPTY2D1	SPT2 Chromatin Protein Domain Containing 1	1.09029
ZNF17	Zinc Finger Protein 17	1.09027
OSER1	Oxidative Stress Responsive Serine Rich 1	1.09027
THUMPD3-AS	THUMPD3 Antisense RNA 1	1.09027
MT-TC	Mitochondrially Encoded TRNA-Cys (UGU/C)	1.09024
TRIM73	Tripartite Motif Containing 73	1.08998
SNORD117	Small Nucleolar RNA, C/D Box 117	1.08987
LINC01229	Long Intergenic Non-Protein Coding RNA 1229	1.08987
ENSG00000224532		1.08987
ENSG00000272221		1.08987
ENSG00000272236		1.08987
RIPPLY2	Ripply Transcriptional Repressor 2	1.08979
DONSON	DNA Replication Fork Stabilization Factor DONSON	1.08932
PNPLA7	Patatin Like Phospholipase Domain Containing 7	1.08932
ATXN7L2	Ataxin 7 Like 2	1.08883
CCDC30	Coiled-Coil Domain Containing 30	1.08883
GTF2H2B	General Transcription Factor IIH Subunit 2B (Pseudogene)	1.08883
TPTEP2	TPTE Pseudogene 2	1.08883
OSBPL8	Oxysterol Binding Protein Like 8	1.08851
ZC3HC1	Zinc Finger C3HC-Type Containing 1	1.08826
WARS2	Tryptophanyl TRNA Synthetase 2, Mitochondrial	1.08756
ZNF132	Zinc Finger Protein 132	1.08747
POU4F3	POU Class 4 Homeobox 3	1.08733
MRPS18B	Mitochondrial Ribosomal Protein S18B	1.08661
GFRA3	GDNF Family Receptor Alpha 3	1.08632
GATA2-AS1	GATA2 Antisense RNA 1	1.08613
CMTM7	CKLF Like MARVEL Transmembrane Domain Containing 7	1.08545
NEDD1	NEDD1 Gamma-Tubulin Ring Complex Targeting Factor	1.08483

ARPP19	CAMP Regulated Phosphoprotein 19	1.08432
TNK1	Tyrosine Kinase Non Receptor 1	1.08340
BOLA3	BolA Family Member 3	1.08323
CMBL	Carboxymethylenebutenolidase Homolog	1.08317
FBXO22	F-Box Protein 22	1.08312
THOC3	THO Complex 3	1.08296
FXYD4	FXYD Domain Containing Ion Transport Regulator 4	1.08137
ZNF441	Zinc Finger Protein 441	1.08137
REX1BD	Required For Excision 1-B Domain Containing	1.08137
LINC00930	Long Intergenic Non-Protein Coding RNA 930	1.08137
ANO6	Anoctamin 6	1.08121
MIR4429	MicroRNA 4429	1.08116
METTL21A	Methyltransferase Like 21A	1.08112
SPRYD3	SPRY Domain Containing 3	1.08112
CCDC184	Coiled-Coil Domain Containing 184	1.08112
IGLL5	Immunoglobulin Lambda Like Polypeptide 5	1.08103
DOLPP1	Dolichyldiphosphatase 1	1.08073
SLC7A6OS	Solute Carrier Family 7 Member 6 Opposite Strand	1.08073
SIKE1	Suppressor Of IKBKE 1	1.08055
KRT74	Keratin 74	1.08024
EDEM1	ER Degradation Enhancing Alpha-Mannosidase Like Protein 1	1.08003
IL17RE	Interleukin 17 Receptor E	1.08003
ZMYND19	Zinc Finger MYND-Type Containing 19	1.08003
MIR1294	MicroRNA 1294	1.07954
TMUB1	Transmembrane And Ubiquitin Like Domain Containing 1	1.07932
MISP3	MISP Family Member 3	1.07932
SAP30L-AS1	SAP30L Antisense RNA 1 (Head To Head)	1.07932
LHX6	LIM Homeobox 6	1.07868
ITGA10	Integrin Subunit Alpha 10	1.07788
CLEC10A	C-Type Lectin Domain Containing 10A	1.07751
MIR573	MicroRNA 573	1.07722
SLC38A6	Solute Carrier Family 38 Member 6	1.07691
TBX10	T-Box Transcription Factor 10	1.07668
LY6G6C	Lymphocyte Antigen 6 Family Member G6C	1.07599
LY6G5C	Lymphocyte Antigen 6 Family Member G5C	1.07599
LY6G6E	Lymphocyte Antigen 6 Family Member G6E	1.07599

MSH5-SAPCD1	MSH5-SAPCD1 Readthrough (NMD Candidate)	1.07599
ENSG00000204422		1.07599
MIR941-1	MicroRNA 941-1	1.07584
ADAM20	ADAM Metallopeptidase Domain 20	1.07506
SLC25A17	Solute Carrier Family 25 Member 17	1.07412
MIR892A	MicroRNA 892a	1.07396
LRRC55	Leucine Rich Repeat Containing 55	1.07391
ENSG00000224810		1.07339
CAPN11	Calpain 11	1.07331
SPDYE14	Speedy/RINGO Cell Cycle Regulator Family Member E14	1.07331
MIR575	MicroRNA 575	1.07310
DYNLT2	Dynein Light Chain Tctex-Type 2	1.07306
TRAPPC11	Trafficking Protein Particle Complex Subunit 11	1.07303
C1QTNF5	C1q And TNF Related 5	1.07220
OPALIN	Oligodendrocytic Myelin Paranodal And Inner Loop Protein	1.07123
RPRD2	Regulation Of Nuclear Pre-MRNA Domain Containing 2	1.07037
DHX57	DExH-Box Helicase 57	1.07037
PHF21B	PHD Finger Protein 21B	1.07012
INSIG1	Insulin Induced Gene 1	1.06979
lnc-TBC1D3K-1		1.06973
lnc-HNF1B-3		1.06973
NONHSAG045678.2-002		1.06973
RAB2B	RAB2B, Member RAS Oncogene Family	1.06886
MACROH2A2	MacroH2A.2 Histone	1.06873
RPUSD3	RNA Pseudouridine Synthase D3	1.06861
FAM83E	Family With Sequence Similarity 83 Member E	1.06861
ZNF34	Zinc Finger Protein 34	1.06854
C22orf31	Chromosome 22 Open Reading Frame 31	1.06854
CAND1.11	Uncharacterized LOC100130460	1.06854
GZF1	GDNF Inducible Zinc Finger Protein 1	1.06782
MAPK8IP3	Mitogen-Activated Protein Kinase 8 Interacting Protein 3	1.06743
SPRR2A	Small Proline Rich Protein 2A	1.06743
ZHX1	Zinc Fingers And Homeoboxes 1	1.06739
KREMEN2	Kringle Containing Transmembrane Protein 2	1.06730
SLC6A12	Solute Carrier Family 6 Member 12	1.06723
FRRS1L	Ferric Chelate Reductase 1 Like	1.06723
SLC9A3-AS1	SLC9A3 Antisense RNA 1	1.06704
MTMR9	Myotubularin Related Protein 9	1.06694
CSTL1	Cystatin Like 1	1.06669

SPCS2	Signal Peptidase Complex Subunit 2	1.06654
P2RX1	Purinergic Receptor P2X 1	1.06639
LINC01355	Long Intergenic Non-Protein Coding RNA 1355	1.06544
OLFML2B	Olfactomedin Like 2B	1.06524
FBXL18	F-Box And Leucine Rich Repeat Protein 18	1.06512
SLC6A16	Solute Carrier Family 6 Member 16	1.06462
DHRS13	Dehydrogenase/Reductase 13	1.06445
ZNF19	Zinc Finger Protein 19	1.06445
RRN3P2	RRN3 Pseudogene 2	1.06445
CCDC163	Coiled-Coil Domain Containing 163	1.06445
HPN-AS1	HPN Antisense RNA 1	1.06445
RPS10P11	Ribosomal Protein S10 Pseudogene 11	1.06445
ALPK1	Alpha Kinase 1	1.06444
PCDHGB3	Protocadherin Gamma Subfamily B, 3	1.06384
PPDPF	Pancreatic Progenitor Cell Differentiation And Proliferation Factor	1.06376
GINS4	GINS Complex Subunit 4	1.06271
VPS13B-DT	VPS13B Divergent Transcript	1.06264
TMEM26	Transmembrane Protein 26	1.06241
TTLL4	Tubulin Tyrosine Ligase Like 4	1.06159
CYP26B1	Cytochrome P450 Family 26 Subfamily B Member 1	1.06145
PCDHGB6	Protocadherin Gamma Subfamily B, 6	1.06139
JSRP1	Junctional Sarcoplasmic Reticulum Protein 1	1.06138
MEG8	Maternally Expressed 8, Small Nucleolar RNA Host Gene	1.06134
RBM7	RNA Binding Motif Protein 7	1.06113
CMC2	C-X9-C Motif Containing 2	1.06047
TRIM52-AS1	TRIM52 Antisense RNA 1 (Head To Head)	1.06039
SETDB2	SET Domain Bifurcated Histone Lysine Methyltransferase 2	1.05987
ARF3	ADP Ribosylation Factor 3	1.05963
GIPC2	GIPC PDZ Domain Containing Family Member 2	1.05924
C11orf21	Chromosome 11 Open Reading Frame 21	1.05917
ZDHHC14	Zinc Finger DHHC-Type Palmitoyltransferase 14	1.05887
ISCU	Iron-Sulfur Cluster Assembly Enzyme	1.05842
CNTD1	Cyclin N-Terminal Domain Containing 1	1.05838
ERICH6B	Glutamate Rich 6B	1.05838
HACD3	3-Hydroxyacyl-CoA Dehydratase 3	1.05815
KIN	Kin17 DNA And RNA Binding Protein	1.05770
LOC10894276(	NANOG 5' Regulatory Region	1.05735

MAP9	Microtubule Associated Protein 9	1.05698
TTC9C	Tetratricopeptide Repeat Domain 9C	1.05665
GNG12-AS1	GNG12, DIRAS3 And WLS Antisense RNA 1	1.05665
DEFB104A	Defensin Beta 104A	1.05644
FFAR3	Free Fatty Acid Receptor 3	1.05574
TMEM170A	Transmembrane Protein 170A	1.05574
SZRD1	SUZ RNA Binding Domain Containing 1	1.05574
RNMT	RNA Guanine-7 Methyltransferase	1.05567
IL21-AS1	IL21 Antisense RNA 1	1.05541
ALDH5A1	Aldehyde Dehydrogenase 5 Family Member A1	1.05534
USP47	Ubiquitin Specific Peptidase 47	1.05516
SEC1P	Secretory Blood Group 1, Pseudogene	1.05469
EPOP	Elongin BC And Polycomb Repressive Complex 2 Associated Protein	1.05380
NAPB	NSF Attachment Protein Beta	1.05350
LINC00641	Long Intergenic Non-Protein Coding RNA 641	1.05129
EVI2B	Ecotropic Viral Integration Site 2B	1.05121
MSC	Musculin	1.05061
KCNN1	Potassium Calcium-Activated Channel Subfamily N Member 1	1.05056
TRIM15	Tripartite Motif Containing 15	1.05032
PPIC	Peptidylprolyl Isomerase C	1.04979
TTYH2	Tweety Family Member 2	1.04961
FNTB	Farnesyltransferase, CAAX Box, Beta	1.04949
IFIT2	Interferon Induced Protein With Tetratricopeptide Repeats 2	1.04925
MIR504	MicroRNA 504	1.04914
DUSP28	Dual Specificity Phosphatase 28	1.04897
NOTCH2NLA	Notch 2 N-Terminal Like A	1.04863
ATP5MK	ATP Synthase Membrane Subunit K	1.04815
ZDHHC21	Zinc Finger DHHC-Type Palmitoyltransferase 21	1.04806
C9orf116	Chromosome 9 Open Reading Frame 116	1.04806
MIR4641	MicroRNA 4641	1.04806
LELP1	Late Cornified Envelope Like Proline Rich 1	1.04791
MIR658	MicroRNA 658	1.04755
HSBP1	Heat Shock Factor Binding Protein 1	1.04741
TPGS1	Tubulin Polyglutamylase Complex Subunit 1	1.04726
IFITM9P	Interferon Induced Transmembrane Protein 9 Pseudogene	1.04685
L3MBTL2	L3MBTL Histone Methyl-Lysine Binding Protein 2	1.04527

CARD18	Caspase Recruitment Domain Family Member 18	1.04504
NKX3-2	NK3 Homeobox 2	1.04489
PLGLB1	Plasminogen Like B1	1.04349
LINC01793	Long Intergenic Non-Protein Coding RNA 1793	1.04324
LINC01555	Long Intergenic Non-Protein Coding RNA 1555	1.04234
TESC	Tescalcin	1.04168
GSKIP	GSK3B Interacting Protein	1.04155
CTDSPL2	CTD Small Phosphatase Like 2	1.04096
PCBP3	Poly(RC) Binding Protein 3	1.04096
ZNF629	Zinc Finger Protein 629	1.04063
TBCA	Tubulin Folding Cofactor A	1.04029
ZAN	Zonadhesin	1.04024
MTRF1	Mitochondrial Translation Release Factor 1	1.03979
KIF27	Kinesin Family Member 27	1.03972
HLX	H2.0 Like Homeobox	1.03968
PPP1R3D	Protein Phosphatase 1 Regulatory Subunit 3D	1.03937
CHST1	Carbohydrate Sulfotransferase 1	1.03906
DOC2B	Double C2 Domain Beta	1.03906
TMEM179B	Transmembrane Protein 179B	1.03906
DPY19L3	Dpy-19 Like C-Mannosyltransferase 3	1.03856
ZBTB39	Zinc Finger And BTB Domain Containing 39	1.03856
NKIRAS2	NFKB Inhibitor Interacting Ras Like 2	1.03824
TMCO4	Transmembrane And Coiled-Coil Domains 4	1.03824
GAL3ST4	Galactose-3-O-Sulfotransferase 4	1.03791
MSRB2	Methionine Sulfoxide Reductase B2	1.03752
FGD5	FYVE, RhoGEF And PH Domain Containing 5	1.03639
ADIRF	Adipogenesis Regulatory Factor	1.03608
CACNA1I	Calcium Voltage-Gated Channel Subunit Alpha1 I	1.03582
ZNF761	Zinc Finger Protein 761	1.03573
ZNF525	Zinc Finger Protein 525	1.03573
ALOX12-AS1	ALOX12 Antisense RNA 1	1.03573
LINC01164	Long Intergenic Non-Protein Coding RNA 1164	1.03573
SAPCD1-AS1	SAPCD1 Antisense RNA 1	1.03573
MICE	MHC Class I Polypeptide-Related Sequence E (Pseudogene)	1.03573
BZW1-AS1	BZW1 Antisense RNA 1	1.03573
KRT18P4	Keratin 18 Pseudogene 4	1.03573
UBE2D3P3	Ubiquitin Conjugating Enzyme E2 D3 Pseudogene 3	1.03573

ENSG00000255730		1.03573
ENSG00000267480		1.03573
ENSG00000262528		1.03573
ENSG00000228779		1.03573
ENSG00000228925		1.03573
LOC10798689	Uncharacterized LOC107986898	1.03573
ENSG00000272040		1.03573
ENSG00000201555		1.03573
lnc-CEACAM20-2		1.03573
lnc-SF3A3-1		1.03573
ENSG00000228510		1.03573
HSALNG0002679		1.03573
HSALNG0126489		1.03573
HSALNG0126491		1.03573
piR-42694-103		1.03573
RF00017-4929		1.03573
HSALNG0087578		1.03573
MN308994		1.03573
RF00017-4926		1.03573
LOC10537575	Uncharacterized LOC105375751	1.03573
ENSG00000227758		1.03573
HSALNG0074702		1.03573
HSALNG0125407		1.03573
RF00017-2612		1.03573
HSALNG0114550		1.03573
RAB3A	RAB3A, Member RAS Oncogene Family	1.03559
NPTXR	Neuronal Pentraxin Receptor	1.03543
CNOT10	CCR4-NOT Transcription Complex Subunit 10	1.03526
EAF1	ELL Associated Factor 1	1.03478
MIR619	MicroRNA 619	1.03409
PRRT3-AS1	PRRT3 Antisense RNA 1	1.03409
HSD17B14	Hydroxysteroid 17-Beta Dehydrogenase 14	1.03018
ARHGEF40	Rho Guanine Nucleotide Exchange Factor 40	1.03018
TSEN2	TRNA Splicing Endonuclease Subunit 2	1.03006
LRCH4	Leucine Rich Repeats And Calponin Homology Domain Containing 4	1.02978
ATP5F1D	ATP Synthase F1 Subunit Delta	1.02978
KIAA1958	KIAA1958	1.02965
LINGO2	Leucine Rich Repeat And Ig Domain Containing 2	1.02924
LINC00939	Long Intergenic Non-Protein Coding RNA 939	1.02913
MIR4447	MicroRNA 4447	1.02913
RNF38	Ring Finger Protein 38	1.02895
ENSG00000273219		1.02827

BTN3A1	Butyrophilin Subfamily 3 Member A1	1.02816
DPYSL4	Dihydropyrimidinase Like 4	1.02816
CRYBA1	Crystallin Beta A1	1.02780
MIR4761	MicroRNA 4761	1.02777
HHLA2	HERV-H LTR-Associating 2	1.02702
GAPVD1	GTPase Activating Protein And VPS9 Domains 1	1.02663
LURAP1	Leucine Rich Adaptor Protein 1	1.02643
LINC01137	Long Intergenic Non-Protein Coding RNA 1137	1.02643
B3GNT2	UDP-GlcNAc:BetaGal Beta-1,3-N-Acetylglucosaminyltransferase 2	1.02640
MIR1-1HG-AS	MIR1-1HG Antisense RNA 1	1.02615
PICSAR	P38 Inhibited Cutaneous Squamous Cell Carcinoma Associated LincRNA	1.02614
EBPL	EBP Like	1.02472
ATP6V0E1	ATPase H+ Transporting V0 Subunit E1	1.02436
MIR659	MicroRNA 659	1.02436
CORO2A	Coronin 2A	1.02393
SLC47A2	Solute Carrier Family 47 Member 2	1.02390
STOML3	Stomatin Like 3	1.02380
ZMAT2	Zinc Finger Matrin-Type 2	1.02365
TIMMDC1	Translocase Of Inner Mitochondrial Membrane Domain Containing 1	1.02360
RTP5	Receptor Transporter Protein 5 (Putative)	1.02311
ARHGAP33	Rho GTPase Activating Protein 33	1.02304
ZNF135	Zinc Finger Protein 135	1.02304
ZNF337	Zinc Finger Protein 337	1.02304
RBIS	Ribosomal Biogenesis Factor	1.02304
MIR634	MicroRNA 634	1.02282
RNU12-2P	RNA, U12 Small Nuclear 2, Pseudogene	1.02261
AGAP3	ArfGAP With GTPase Domain, Ankyrin Repeat And PH Domain 3	1.02257
DISP3	Dispatched RND Transporter Family Member 3	1.02250
MIR520D	MicroRNA 520d	1.02225
POLR2J	RNA Polymerase II Subunit J	1.02143
CNTFR	Ciliary Neurotrophic Factor Receptor	1.02126
TXNDC12	Thioredoxin Domain Containing 12	1.02089
OR1J2	Olfactory Receptor Family 1 Subfamily J Member 2	1.02085
CHMP7	Charged Multivesicular Body Protein 7	1.02014
LENG1	Leukocyte Receptor Cluster Member 1	1.02014
CLDN22	Claudin 22	1.02004
CLDN24	Claudin 24	1.02004
ATP5MC2	ATP Synthase Membrane Subunit C Locus 2	1.01984
NKRF	NFKB Repressing Factor	1.01983

DPCD	Deleted In Primary Ciliary Dyskinesia Homolog (Mouse)	1.01973
ATG9A	Autophagy Related 9A	1.01973
POP7	POP7 Homolog, Ribonuclease P/MRP Subunit	1.01958
LCN1P1	Lipocalin 1 Pseudogene 1	1.01939
MRFAP1	Morf4 Family Associated Protein 1	1.01873
MIR1-1HG	MIR1-1 Host Gene	1.01825
LOC285593	Uncharacterized LOC285593	1.01825
MIR1182	MicroRNA 1182	1.01806
SUPV3L1	Suv3 Like RNA Helicase	1.01791
DEUP1	Deuterosome Assembly Protein 1	1.01688
ZNF28	Zinc Finger Protein 28	1.01670
CLK4	CDC Like Kinase 4	1.01513
TAF10	TATA-Box Binding Protein Associated Factor 10	1.01513
PKIG	CAMP-Dependent Protein Kinase Inhibitor Gamma	1.01497
DUSP18	Dual Specificity Phosphatase 18	1.01497
DIRAS2	DIRAS Family GTPase 2	1.01497
THAP6	THAP Domain Containing 6	1.01497
RIBC2	RIB43A Domain With Coiled-Coils 2	1.01497
TMEM183A	Transmembrane Protein 183A	1.01497
NSG2	Neuronal Vesicle Trafficking Associated 2	1.01497
CSKMT	Citrate Synthase Lysine Methyltransferase	1.01497
SPDYE5	Speedy/RINGO Cell Cycle Regulator Family Member E5	1.01497
LINC02363	Long Intergenic Non-Protein Coding RNA 2363	1.01497
CERS6	Ceramide Synthase 6	1.01480
BCAT2	Branched Chain Amino Acid Transaminase 2	1.01446
SINHCAF	SIN3-HDAC Complex Associated Factor	1.01430
DYNC1I1	Dynein Cytoplasmic 1 Intermediate Chain 1	1.01397
PELI1	Pellino E3 Ubiquitin Protein Ligase 1	1.01382
FAM220A	Family With Sequence Similarity 220 Member A	1.01354
MIR3978	MicroRNA 3978	1.01313
PARP16	Poly(ADP-Ribose) Polymerase Family Member 16	1.01310
GPR155	G Protein-Coupled Receptor 155	1.01305
B3GNT7	UDP-GlcNAc:BetaGal Beta-1,3-N-Acetylglucosaminyltransferase 7	1.01261
SCLY	Selenocysteine Lyase	1.01194

RAB8B	RAB8B, Member RAS Oncogene Family	1.01194
RBAK	RB Associated KRAB Zinc Finger	1.01194
ZNF420	Zinc Finger Protein 420	1.01194
FAM114A1	Family With Sequence Similarity 114 Member A1	1.01194
PCDHB3	Protocadherin Beta 3	1.01194
ZNF568	Zinc Finger Protein 568	1.01194
RNU6ATAC	RNA, U6atac Small Nuclear (U12-Dependent Splicing)	1.01129
TTC33	Tetratricopeptide Repeat Domain 33	1.01079
RETREG2	Reticulophagy Regulator Family Member 2	1.01045
CHSY3	Chondroitin Sulfate Synthase 3	1.01036
MANSC1	MANSC Domain Containing 1	1.01030
QRICH2	Glutamine Rich 2	1.01030
PRKAG2-AS1	PRKAG2 Antisense RNA 1	1.01030
LINC00592	Long Intergenic Non-Protein Coding RNA 592	1.01030
SDCBP2-AS1	SDCBP2 Antisense RNA 1	1.01030
VPS37D	VPS37D Subunit Of ESCRT-I	1.01002
KCNRG	Potassium Channel Regulator	1.00979
TTI1	TELO2 Interacting Protein 1	1.00936
DUSP11	Dual Specificity Phosphatase 11	1.00936
ZBTB34	Zinc Finger And BTB Domain Containing 34	1.00936
NCLN	Nicalin	1.00923
SGTA	Small Glutamine Rich Tetratricopeptide Repeat Co-Chaperone Alpha	1.00913
TRPT1	TRNA Phosphotransferase 1	1.00850
BTN2A2	Butyrophilin Subfamily 2 Member A2	1.00838
PDE3A	Phosphodiesterase 3A	1.00816
FEM1A	Fem-1 Homolog A	1.00792
LOC11125564:	TNFRSF10A 5' Regulatory Region	1.00781
MDM1	Mdm1 Nuclear Protein	1.00778
ZNF280D	Zinc Finger Protein 280D	1.00778
TPGS2	Tubulin Polyglutamylase Complex Subunit 2	1.00699
CFDP1	Craniofacial Development Protein 1	1.00583
CPEB2	Cytoplasmic Polyadenylation Element Binding Protein 2	1.00548
LOC11125852(	NOS1 1c Alternate Promoter	1.00548
LMAN2	Lectin, Mannose Binding 2	1.00545
LOC11183267(	Alpha Fetoprotein (AFP) 5' Regulatory Region	1.00426
WDR31	WD Repeat Domain 31	1.00335
ZNF511	Zinc Finger Protein 511	1.00335
DNAJC4	DnaJ Heat Shock Protein Family (Hsp40) Member C4	1.00335

SPDYE16	Speedy/RINGO Cell Cycle Regulator Family Member E16	1.00335
LRRC37BP1	Leucine Rich Repeat Containing 37B Pseudogene 1	1.00335
DERL2	Derlin 2	1.00312
IPO13	Importin 13	1.00275
YAE1	YAE1 Maturation Factor Of ABCE1	1.00187
PLXNA3	Plexin A3	1.00119
CCDC43	Coiled-Coil Domain Containing 43	1.00107
NRDC	Nardilysin Convertase	1.00047
SEPTIN6	Septin 6	0.99999
FBXO39	F-Box Protein 39	0.99976
TEX43	Testis Expressed 43	0.99923
DMXL1	Dmx Like 1	0.99919
CLDN19	Claudin 19	0.99900
MOBP	Myelin Associated Oligodendrocyte Basic Protein	0.99900
CHRDL1	Chordin Like 1	0.99880
C3orf18	Chromosome 3 Open Reading Frame 18	0.99830
SMDT1	Single-Pass Membrane Protein With Aspartate Rich Tail 1	0.99830
GPR62	G Protein-Coupled Receptor 62	0.99830
PRSS45P	Serine Protease 45, Pseudogene	0.99830
LCNL1	Lipocalin Like 1	0.99830
BTBD19	BTB Domain Containing 19	0.99830
MIR640	MicroRNA 640	0.99830
RPS19P3	Ribosomal Protein S19 Pseudogene 3	0.99830
ENSG00000256514		0.99830
PDIA4	Protein Disulfide Isomerase Family A Member 4	0.99776
SLC39A5	Solute Carrier Family 39 Member 5	0.99760
KIAA1191	KIAA1191	0.99557
NDUFB2	NADH:Ubiquinone Oxidoreductase Subunit B2	0.99541
CD300LF	CD300 Molecule Like Family Member F	0.99384
TMOD4	Tropomodulin 4	0.99380
CENPX	Centromere Protein X	0.99349
CEP68	Centrosomal Protein 68	0.99325
RNF181	Ring Finger Protein 181	0.99306
FBXO45	F-Box Protein 45	0.99200
FLJ22447	Uncharacterized LOC400221	0.99115
BCKDK	Branched Chain Keto Acid Dehydrogenase Kinase	0.99068
GPSM1	G Protein Signaling Modulator 1	0.99065
KIF9	Kinesin Family Member 9	0.99037
CCDC153	Coiled-Coil Domain Containing 153	0.99037
EEF1A1P5	Eukaryotic Translation Elongation Factor 1 Alpha 1 Pseudogene 5	0.99026

BLOC1S2	Biogenesis Of Lysosomal Organelles Complex 1 Subunit 2	0.98908
GIMAP5	GTPase, IMAP Family Member 5	0.98651
CRIP2	Cysteine Rich Protein 2	0.98627
REPIN1	Replication Initiator 1	0.98627
CEMP1	Cementum Protein 1	0.98627
WBP1	WW Domain Binding Protein 1	0.98559
MRPL32	Mitochondrial Ribosomal Protein L32	0.98541
INO80C	INO80 Complex Subunit C	0.98541
MIR1256	MicroRNA 1256	0.98537
PDSS1	Decaprenyl Diphosphate Synthase Subunit 1	0.98500
CRYBB2P1	Crystallin Beta B2 Pseudogene 1	0.98481
SPEM1	Spermatid Maturation 1	0.98410
ORMDL3	ORMDL Sphingolipid Biosynthesis Regulator 3	0.98338
FAM118A	Family With Sequence Similarity 118 Member A	0.98305
CCDC51	Coiled-Coil Domain Containing 51	0.98305
LINC00114	Long Intergenic Non-Protein Coding RNA 114	0.98305
LSP1P4	LSP1 Pseudogene 4	0.98177
TBC1D9	TBC1 Domain Family Member 9	0.98114
C12orf4	Chromosome 12 Open Reading Frame 4	0.98059
NDEL1	NudE Neurodevelopment Protein 1 Like 1	0.98056
SEC62-AS1	SEC62 Antisense RNA 1	0.98014
SNUPN	Snurportin 1	0.97988
SYPL1	Synaptophysin Like 1	0.97986
PTMS	Parathymosin	0.97947
ARHGAP4	Rho GTPase Activating Protein 4	0.97945
CLDN12	Claudin 12	0.97879
TECR	Trans-2,3-Enoyl-CoA Reductase	0.97851
TRMT5	TRNA Methyltransferase 5	0.97808
PCDHB4	Protocadherin Beta 4	0.97808
MED18	Mediator Complex Subunit 18	0.97804
C19orf53	Chromosome 19 Open Reading Frame 53	0.97794
TMEM150A	Transmembrane Protein 150A	0.97758
TYW5	TRNA-YW Synthesizing Protein 5	0.97758
ANKRD2	Ankyrin Repeat Domain 2	0.97729
MCRIP1	MAPK Regulated Corepressor Interacting Protein 1	0.97699
MT-RNR2	Mitochondrially Encoded 16S RRNA	0.97543
HAUS3	HAUS Augmin Like Complex Subunit 3	0.97517
FLYWCH2	FLYWCH Family Member 2	0.97515
DIPK2A	Divergent Protein Kinase Domain 2A	0.97450
ADM5	Adrenomedullin 5 (Putative)	0.97428

FOLH1B	Folate Hydrolase 1B	0.97356
MIR641	MicroRNA 641	0.97348
CFAP157	Cilia And Flagella Associated Protein 157	0.97328
RADIL	Rap Associating With DIL Domain	0.97317
ZFYVE27	Zinc Finger FYVE-Type Containing 27	0.97268
MIR3664	MicroRNA 3664	0.97260
FAM136A	Family With Sequence Similarity 136 Member A	0.97222
TYSND1	Trypsin Like Peroxisomal Matrix Peptidase 1	0.97175
ZNF74	Zinc Finger Protein 74	0.97086
PKD1L1	Polycystin 1 Like 1, Transient Receptor Potential Channel Interacting	0.97086
TMEM102	Transmembrane Protein 102	0.97026
TMEM44	Transmembrane Protein 44	0.97026
ASPRV1	Aspartic Peptidase Retroviral Like 1	0.96996
EARS2	Glutamyl-TRNA Synthetase 2, Mitochondrial	0.96903
MIR646	MicroRNA 646	0.96880
YOD1	YOD1 Deubiquitinase	0.96837
FAM222B	Family With Sequence Similarity 222 Member B	0.96756
DNAJB2	DnaJ Heat Shock Protein Family (Hsp40) Member B2	0.96754
PRSS27	Serine Protease 27	0.96749
MFSD5	Major Facilitator Superfamily Domain Containing 5	0.96749
ZNF177	Zinc Finger Protein 177	0.96749
AMY2A	Amylase Alpha 2A	0.96720
UBL4A	Ubiquitin Like 4A	0.96720
TRAPPC5	Trafficking Protein Particle Complex Subunit 5	0.96673
FMNL3	Formin Like 3	0.96559
SLBP	Stem-Loop Binding Protein	0.96547
USP19	Ubiquitin Specific Peptidase 19	0.96449
AFTPH	Aftiphilin	0.96436
H2BC4	H2B Clustered Histone 4	0.96403
ZFAND4	Zinc Finger AN1-Type Containing 4	0.96326
PJA1	Praja Ring Finger Ubiquitin Ligase 1	0.96238
AOAH	Acyloxyacyl Hydrolase	0.96195
MIR561	MicroRNA 561	0.96149
ITM2C	Integral Membrane Protein 2C	0.96133
PSMB8-AS1	PSMB8 Antisense RNA 1 (Head To Head)	0.96090
ENSG00000250264		0.96090
NELFE	Negative Elongation Factor Complex Member E	0.96084

CC2D1A	Coiled-Coil And C2 Domain Containing 1A	0.96056
POLR3G	RNA Polymerase III Subunit G	0.95982
BCL2L15	BCL2 Like 15	0.95920
LSM4	LSM4 Homolog, U6 Small Nuclear RNA And MRNA Degradation Associated	0.95814
MIR567	MicroRNA 567	0.95722
ZNF207	Zinc Finger Protein 207	0.95713
SCAF11	SR-Related CTD Associated Factor 11	0.95713
ZMAT5	Zinc Finger Matrin-Type 5	0.95670
C1orf167	Chromosome 1 Open Reading Frame 167	0.95670
MIR3183	MicroRNA 3183	0.95670
MIR4740	MicroRNA 4740	0.95670
ADRA2C	Adrenoceptor Alpha 2C	0.95576
ADRA2B	Adrenoceptor Alpha 2B	0.95561
PCDHA9	Protocadherin Alpha 9	0.95556
MUL1	Mitochondrial E3 Ubiquitin Protein Ligase 1	0.95460
RAB9A	RAB9A, Member RAS Oncogene Family	0.95436
MIR3188	MicroRNA 3188	0.95398
SLC7A2	Solute Carrier Family 7 Member 2	0.95389
MIR3127	MicroRNA 3127	0.95383
ARHGAP19	Rho GTPase Activating Protein 19	0.95358
CFAP97	Cilia And Flagella Associated Protein 97	0.95358
GFRA2	GDNF Family Receptor Alpha 2	0.95329
METTL27	Methyltransferase Like 27	0.95279
AIFM3	Apoptosis Inducing Factor Mitochondria Associated 3	0.95186
MRPL48	Mitochondrial Ribosomal Protein L48	0.95183
RASL11A	RAS Like Family 11 Member A	0.95183
TCEAL2	Transcription Elongation Factor A Like 2	0.95167
MIR4286	MicroRNA 4286	0.95167
ATP13A2	ATPase Cation Transporting 13A2	0.94619
JAM2	Junctional Adhesion Molecule 2	0.94526
RHBDD3	Rhomboid Domain Containing 3	0.94484
PPCDC	Phosphopantothenoylcysteine Decarboxylase	0.94391
NXT1	Nuclear Transport Factor 2 Like Export Factor 1	0.94391
SCAMP5	Secretory Carrier Membrane Protein 5	0.94391
GHDC	GH3 Domain Containing	0.94391
RNF157	Ring Finger Protein 157	0.94391
PCDHGC4	Protocadherin Gamma Subfamily C, 4	0.94391
COMMD2	COMM Domain Containing 2	0.94391
TMEM69	Transmembrane Protein 69	0.94391

LRRC45	Leucine Rich Repeat Containing 45	0.94391
PI4KAP2	Phosphatidylinositol 4-Kinase Alpha Pseudogene 2	0.94391
LINC00205	Long Intergenic Non-Protein Coding RNA 205	0.94391
LOC10012961[illegible]	Uncharacterized LOC100129617	0.94391
LINC01679	Long Intergenic Non-Protein Coding RNA 1679	0.94361
TBC1D20	TBC1 Domain Family Member 20	0.94357
RHCG	Rh Family C Glycoprotein	0.94231
USP13	Ubiquitin Specific Peptidase 13	0.94197
LSM5	LSM5 Homolog, U6 Small Nuclear RNA And MRNA Degradation Associated	0.94176
GRIP1	Glutamate Receptor Interacting Protein 1	0.94108
EPS8L2	EPS8 Like 2	0.94090
PAQR5	Progestin And AdipoQ Receptor Family Member 5	0.94058
METTL17	Methyltransferase Like 17	0.94058
ALDH3B1	Aldehyde Dehydrogenase 3 Family Member B1	0.94030
NCAPD3	Non-SMC Condensin II Complex Subunit D3	0.94020
ENSG00000270096		0.93988
ENSG00000272657		0.93988
MIR4510	MicroRNA 4510	0.93926
NKD1	NKD Inhibitor Of WNT Signaling Pathway 1	0.93905
CYB5RL	Cytochrome B5 Reductase Like	0.93888
MRPL39	Mitochondrial Ribosomal Protein L39	0.93784
ZNF260	Zinc Finger Protein 260	0.93756
CRLS1	Cardiolipin Synthase 1	0.93735
MRPL53	Mitochondrial Ribosomal Protein L53	0.93708
AGPAT5	1-Acylglycerol-3-Phosphate O-Acyltransferase 5	0.93648
STXBP6	Syntaxin Binding Protein 6	0.93648
FILNC1	FOXO Induced Long Non-Coding RNA 1	0.93648
ZBED2	Zinc Finger BED-Type Containing 2	0.93597
MIR558	MicroRNA 558	0.93595
CEP170B	Centrosomal Protein 170B	0.93561
LOC11530816[illegible]	Uncharacterized LOC115308161	0.93414
TRIM50	Tripartite Motif Containing 50	0.93387
NUP210L	Nucleoporin 210 Like	0.93323
KCNK15	Potassium Two Pore Domain Channel Subfamily K Member 15	0.93302
KRT82	Keratin 82	0.93302
KRTAP2-1	Keratin Associated Protein 2-1	0.93302

SEC14L6	SEC14 Like Lipid Binding 6	0.93302
SPEM2	SPEM Family Member 2	0.93302
SNORA54	Small Nucleolar RNA, H/ACA Box 54	0.93302
IPO9-AS1	IPO9 Antisense RNA 1	0.93302
JOSD1	Josephin Domain Containing 1	0.93105
MFSD12	Major Facilitator Superfamily Domain Containing 12	0.93105
SPRYD7	SPRY Domain Containing 7	0.93100
ESAM	Endothelial Cell Adhesion Molecule	0.93047
LIN52	Lin-52 DREAM MuvB Core Complex Component	0.93018
SGSM1	Small G Protein Signaling Modulator 1	0.93017
BTN3A3	Butyrophilin Subfamily 3 Member A3	0.93017
MIER2	MIER Family Member 2	0.92967
CAPN13	Calpain 13	0.92950
GALNT17	Polypeptide N-Acetylgalactosaminyltransferase 17	0.92950
TMEM221	Transmembrane Protein 221	0.92950
KIF9-AS1	KIF9 Antisense RNA 1	0.92950
GSAP	Gamma-Secretase Activating Protein	0.92945
TRIM58	Tripartite Motif Containing 58	0.92910
MIR618	MicroRNA 618	0.92884
KLHDC2	Kelch Domain Containing 2	0.92837
PCDHGC5	Protocadherin Gamma Subfamily C, 5	0.92723
RAB19	RAB19, Member RAS Oncogene Family	0.92723
DNLZ	DNL-Type Zinc Finger	0.92723
ANKRD34A	Ankyrin Repeat Domain 34A	0.92723
FAM110D	Family With Sequence Similarity 110 Member D	0.92723
TNFSF12-TNFS	TNFSF12-TNFSF13 Readthrough	0.92723
C11orf91	Chromosome 11 Open Reading Frame 91	0.92723
LINC00304	Long Intergenic Non-Protein Coding RNA 304	0.92723
AMZ2P1	AMZ2 Pseudogene 1	0.92723
P3R3URF	PIK3R3 Upstream Open Reading Frame	0.92723
MIR6088	MicroRNA 6088	0.92723
INHCAP	Inhibitor Of Carbonic Anhydrase Pseudogene	0.92723
ENSG00000284969		0.92723
ENSG00000284989		0.92723
FAIM	Fas Apoptotic Inhibitory Molecule	0.92530
MIR633	MicroRNA 633	0.92419
PDE4C	Phosphodiesterase 4C	0.92281
SPATC1L	Spermatogenesis And Centriole Associated 1 Like	0.92281
C11orf52	Chromosome 11 Open Reading Frame 52	0.92281

TK2	Thymidine Kinase 2	0.92210
KCNMB3	Potassium Calcium-Activated Channel Subfamily M Regulatory Beta Subunit 3	0.92086
WDR41	WD Repeat Domain 41	0.92086
KHDC1	KH Domain Containing 1	0.92086
CPED1	Cadherin Like And PC-Esterase Domain Containing 1	0.92086
ROGDI	Rogdi Atypical Leucine Zipper	0.92064
ZNF347	Zinc Finger Protein 347	0.92064
TMEM145	Transmembrane Protein 145	0.92064
SNORD52	Small Nucleolar RNA, C/D Box 52	0.92064
PPP1R2P1	Protein Phosphatase 1 Regulatory Inhibitor Subunit 2 Pseudogene 1	0.92064
ADCY10P1	ADCY10 Pseudogene 1	0.92064
RPL13P5	Ribosomal Protein L13 Pseudogene 5	0.92064
CENATAC-DT	CENATAC Divergent Transcript	0.92064
RPL17P7	Ribosomal Protein L17 Pseudogene 7	0.92064
FTH1P1	Ferritin Heavy Chain 1 Pseudogene 1	0.92064
ENSG00000261338		0.92064
PHBP9	Prohibitin Pseudogene 9	0.92064
HSPD1P4	Heat Shock Protein Family D (Hsp60) Member 1 Pseudogene 4	0.92064
RNU1-88P	RNA, U1 Small Nuclear 88, Pseudogene	0.92064
ENSG00000234055		0.92064
RN7SL636P	RNA, 7SL, Cytoplasmic 636, Pseudogene	0.92064
ENSG00000273373		0.92064
ENSG00000267605		0.92064
ENSG00000223837		0.92064
RPS6P16	Ribosomal Protein S6 Pseudogene 16	0.92064
RN7SL444P	RNA, 7SL, Cytoplasmic 444, Pseudogene	0.92064
RNU6-510P	RNA, U6 Small Nuclear 510, Pseudogene	0.92064
ENSG00000252200		0.92064
ENSG00000285565		0.92064
ENSG00000285601		0.92064
ENSG00000235286		0.92064
lnc-CXCR5-2		0.92064
lnc-RPS25-6		0.92064
lnc-HSPA1B-1		0.92064
HSALNG0022244		0.92064
HSALNG0049332		0.92064
HSALNG0049336		0.92064
ENSG00000242198		0.92064
HSALNG0049754		0.92064
HSALNG0049335		0.92064
HSALNG0049333		0.92064
HSALNG0087588		0.92064

HSALNG0087589		0.92064
piR-54824-001		0.92064
piR-54824-002		0.92064
RF00017-4996		0.92064
RF02725		0.92064
RF00017-4994		0.92064
RF00495		0.92064
MN298114-198		0.92064
piR-34881		0.92064
RF00017-4998		0.92064
HSALNG0074700		0.92064
HSALNG0074703		0.92064
HSALNG0074704		0.92064
HSALNG0124555		0.92064
MK280266		0.92064
LOC10537265	Uncharacterized LOC105372657	0.92064
HE856076		0.92064
HSALNG0130761		0.92064
HSALNG0130762		0.92064
piR-58297-237		0.92064
POLR1E	RNA Polymerase I Subunit E	0.92038
NOL9	Nucleolar Protein 9	0.92038
ZNF746	Zinc Finger Protein 746	0.91950
ARL14	ADP Ribosylation Factor Like GTPase 14	0.91937
PPP2R2D	Protein Phosphatase 2 Regulatory Subunit Bdelta	0.91935
ZNF316	Zinc Finger Protein 316	0.91900
MIR1244-1	MicroRNA 1244-1	0.91900
UTP6	UTP6 Small Subunit Processome Component	0.91871
MEX3B	Mex-3 RNA Binding Family Member B	0.91854
LENEP	Lens Epithelial Protein	0.91841
TSEN34	TRNA Splicing Endonuclease Subunit 34	0.91783
RAPGEF4	Rap Guanine Nucleotide Exchange Factor 4	0.91748
SPAG7	Sperm Associated Antigen 7	0.91701
LOC11760000	CTSL Promoter Region	0.91639
SHISA5	Shisa Family Member 5	0.91621
SULT1A4	Sulfotransferase Family 1A Member 4	0.91491
SULT1C2	Sulfotransferase Family 1C Member 2	0.91315
LRRC20	Leucine Rich Repeat Containing 20	0.91315
CHCHD1	Coiled-Coil-Helix-Coiled-Coil-Helix Domain Containing 1	0.91315
CRACR2B	Calcium Release Activated Channel Regulator 2B	0.91307
ITGB2-AS1	ITGB2 Antisense RNA 1	0.91307
ADPGK-AS1	ADPGK Antisense RNA 1	0.91187

MTBP	MDM2 Binding Protein	0.91181
ZNF335	Zinc Finger Protein 335	0.91160
ZNF714	Zinc Finger Protein 714	0.91135
LINC00222	Long Intergenic Non-Protein Coding RNA 222	0.91106
SMIM15	Small Integral Membrane Protein 15	0.91063
PACS2	Phosphofurin Acidic Cluster Sorting Protein 2	0.91048
PLEKHA3	Pleckstrin Homology Domain Containing A3	0.90662
DHRSX	Dehydrogenase/Reductase X-Linked	0.90662
TTTY14	Testis-Specific Transcript, Y-Linked 14	0.90662
THUMPD3	THUMP Domain Containing 3	0.90632
TMEM50B	Transmembrane Protein 50B	0.90632
HROB	Homologous Recombination Factor With OB-Fold	0.90632
ESPNP	Espin Pseudogene	0.90632
MIR4674	MicroRNA 4674	0.90632
IDO2	Indoleamine 2,3-Dioxygenase 2	0.90627
LMO3	LIM Domain Only 3	0.90536
KCND1	Potassium Voltage-Gated Channel Subfamily D Member 1	0.90527
COG1	Component Of Oligomeric Golgi Complex 1	0.90505
CCDC88B	Coiled-Coil Domain Containing 88B	0.90505
ACY3	Aminoacylase 3	0.90447
PHETA2	PH Domain Containing Endocytic Trafficking Adaptor 2	0.90447
C6orf15	Chromosome 6 Open Reading Frame 15	0.90439
NFRKB	Nuclear Factor Related To KappaB Binding Protein	0.90399
RAB13	RAB13, Member RAS Oncogene Family	0.90398
AIG1	Androgen Induced 1	0.90352
GRIN2C	Glutamate Ionotropic Receptor NMDA Type Subunit 2C	0.90295
OSR2	Odd-Skipped Related Transciption Factor 2	0.90209
COPZ1	COPI Coat Complex Subunit Zeta 1	0.90137
GET1	Guided Entry Of Tail-Anchored Proteins Factor 1	0.90007
FAM86JP	Family With Sequence Similarity 86 Member J, Pseudogene	0.89971
MIR585	MicroRNA 585	0.89872
SNORD83B	Small Nucleolar RNA, C/D Box 83B	0.89862
ZC2HC1C	Zinc Finger C2HC-Type Containing 1C	0.89835
CFD	Complement Factor D	0.89835
TRMT12	TRNA Methyltransferase 12 Homolog	0.89804
NKX1-1	NK1 Homeobox 1	0.89792

UNC119B	Unc-119 Lipid Binding Chaperone B	0.89773
LYPLAL1	Lysophospholipase Like 1	0.89744
ZKSCAN5	Zinc Finger With KRAB And SCAN Domains 5	0.89682
ANKRD35	Ankyrin Repeat Domain 35	0.89682
PHOX2A	Paired Like Homeobox 2A	0.89669
LINC01140	Long Intergenic Non-Protein Coding RNA 1140	0.89657
SRPRB	SRP Receptor Subunit Beta	0.89637
PATL1	PAT1 Homolog 1, Processing Body MRNA Decay Factor	0.89626
POM121C	POM121 Transmembrane Nucleoporin C	0.89492
UQCC3	Ubiquinol-Cytochrome C Reductase Complex Assembly Factor 3	0.89427
LIPJ	Lipase Family Member J	0.89398
WASHC5-AS1	WASHC5 Antisense RNA 1	0.89398
ZNF219	Zinc Finger Protein 219	0.89341
RPLP0P6	Ribosomal Protein Lateral Stalk Subunit P0 Pseudogene 6	0.89341
KCNK10	Potassium Two Pore Domain Channel Subfamily K Member 10	0.89340
ARHGAP12	Rho GTPase Activating Protein 12	0.89281
COX16	Cytochrome C Oxidase Assembly Factor COX16	0.89236
KATNBL1	Katanin Regulatory Subunit B1 Like 1	0.89236
TENM2	Teneurin Transmembrane Protein 2	0.89175
GALNTL5	Polypeptide N-Acetylgalactosaminyltransferase Like 5	0.89162
FUT9	Fucosyltransferase 9	0.89047
GIMAP8	GTPase, IMAP Family Member 8	0.89021
KLHL35	Kelch Like Family Member 35	0.89021
TMEM132A	Transmembrane Protein 132A	0.89021
HSD17B1P1	Hydroxysteroid 17-Beta Dehydrogenase 1 Pseudogene 1	0.89021
MIR9898	MicroRNA 9898	0.89021
GPR37	G Protein-Coupled Receptor 37	0.88998
GPR17	G Protein-Coupled Receptor 17	0.88780
NPIPA5	Nuclear Pore Complex Interacting Protein Family Member A5	0.88780
MIR3605	MicroRNA 3605	0.88780
RNF138	Ring Finger Protein 138	0.88688
ZSCAN9	Zinc Finger And SCAN Domain Containing 9	0.88669
PUM3	Pumilio RNA Binding Family Member 3	0.88640
HOMER3	Homer Scaffold Protein 3	0.88606
PITHD1	PITH Domain Containing 1	0.88606
ZNF263	Zinc Finger Protein 263	0.88603

R3HCC1L	R3H Domain And Coiled-Coil Containing 1 Like	0.88603
TM2D2	TM2 Domain Containing 2	0.88603
BORCS6	BLOC-1 Related Complex Subunit 6	0.88603
ACAA1	Acetyl-CoA Acyltransferase 1	0.88577
BTAF1	B-TFIID TATA-Box Binding Protein Associated Factor 1	0.88414
ATP1B3	ATPase Na+/K+ Transporting Subunit Beta 3	0.88331
PRDM4	PR/SET Domain 4	0.88205
NFATC2IP	Nuclear Factor Of Activated T Cells 2 Interacting Protein	0.88147
LIX1L	Limb And CNS Expressed 1 Like	0.88147
DHX34	DExH-Box Helicase 34	0.88105
ABTB2	Ankyrin Repeat And BTB Domain Containing 2	0.87942
ANKRD61	Ankyrin Repeat Domain 61	0.87942
DYNLT4	Dynein Light Chain Tctex-Type 4	0.87942
MIR3128	MicroRNA 3128	0.87942
MIR4669	MicroRNA 4669	0.87942
LINC01939	Long Intergenic Non-Protein Coding RNA 1939	0.87847
ZNF205	Zinc Finger Protein 205	0.87816
DEDD	Death Effector Domain Containing	0.87751
NADK2	NAD Kinase 2, Mitochondrial	0.87718
FAM166A	Family With Sequence Similarity 166 Member A	0.87710
ACSM3	Acyl-CoA Synthetase Medium Chain Family Member 3	0.87586
ZNF580	Zinc Finger Protein 580	0.87586
MCRIP2	MAPK Regulated Corepressor Interacting Protein 2	0.87586
CALY	Calcyon Neuron Specific Vesicular Protein	0.87384
CFAP43	Cilia And Flagella Associated Protein 43	0.87384
MTRNR2L4	MT-RNR2 Like 4	0.87384
DDX24	DEAD-Box Helicase 24	0.87358
H2BC3	H2B Clustered Histone 3	0.87331
HGH1	HGH1 Homolog	0.87323
CBWD2	COBW Domain Containing 2	0.87323
MARF1	Meiosis Regulator And MRNA Stability Factor 1	0.87323
NEDD8-MDP1	NEDD8-MDP1 Readthrough	0.87323
RIPPLY3	Ripply Transcriptional Repressor 3	0.87293
AP1G2	Adaptor Related Protein Complex 1 Subunit Gamma 2	0.87262
AOX1	Aldehyde Oxidase 1	0.87198
POC1A	POC1 Centriolar Protein A	0.87133
COQ9	Coenzyme Q9	0.87112

GGTLC3	Gamma-Glutamyltransferase Light Chain Family Member 3	0.87008
RAB43	RAB43, Member RAS Oncogene Family	0.86970
USP29	Ubiquitin Specific Peptidase 29	0.86867
KIR2DL4	Killer Cell Immunoglobulin Like Receptor, Two Ig Domains And Long Cytoplasmic Tail 4	0.86863
CMPK1	Cytidine/Uridine Monophosphate Kinase 1	0.86744
GPR132	G Protein-Coupled Receptor 132	0.86720
INSYN1	Inhibitory Synaptic Factor 1	0.86675
WASHC2A	WASH Complex Subunit 2A	0.86675
CD53	CD53 Molecule	0.86663
ERP27	Endoplasmic Reticulum Protein 27	0.86663
SPPL3	Signal Peptide Peptidase Like 3	0.86663
GPR157	G Protein-Coupled Receptor 157	0.86663
COPZ2	COPI Coat Complex Subunit Zeta 2	0.86663
CNFN	Cornifelin	0.86663
GUCD1	Guanylyl Cyclase Domain Containing 1	0.86663
CEP95	Centrosomal Protein 95	0.86663
KRTAP3-2	Keratin Associated Protein 3-2	0.86663
FAM219A	Family With Sequence Similarity 219 Member A	0.86663
BOLA2B	BolA Family Member 2B	0.86663
TTC16	Tetratricopeptide Repeat Domain 16	0.86663
LINC00482	Long Intergenic Non-Protein Coding RNA 482	0.86663
GSN-AS1	GSN Antisense RNA 1	0.86663
FAM86C2P	Family With Sequence Similarity 86 Member C2, Pseudogene	0.86663
SERPINB9P1	Serpin Family B Member 9 Pseudogene 1	0.86663
KLHL6	Kelch Like Family Member 6	0.86588
TMEM132D-A	TMEM132D Antisense RNA 1	0.86476
RSRC1	Arginine And Serine Rich Coiled-Coil 1	0.86451
CIDEA	Cell Death Inducing DFFA Like Effector A	0.86285
NPY4R	Neuropeptide Y Receptor Y4	0.86204
FXYD2	FXYD Domain Containing Ion Transport Regulator 2	0.86106
LRRC49	Leucine Rich Repeat Containing 49	0.86006
C7orf26	Chromosome 7 Open Reading Frame 26	0.86006
IPP	Intracisternal A Particle-Promoted Polypeptide	0.85940
CALCB	Calcitonin Related Polypeptide Beta	0.85905
TAFA5	TAFA Chemokine Like Family Member 5	0.85860

PKD1P1	Polycystin 1, Transient Receptor Potential Channel Interacting Pseudogene 1	0.85853
ZSCAN18	Zinc Finger And SCAN Domain Containing 18	0.85817
PHACTR2-AS1	PHACTR2 Antisense RNA 1	0.85740
PNRC2	Proline Rich Nuclear Receptor Coactivator 2	0.85703
LBX2	Ladybird Homeobox 2	0.85656
PRRT3	Proline Rich Transmembrane Protein 3	0.85656
RHPN1	Rhophilin Rho GTPase Binding Protein 1	0.85592
COMTD1	Catechol-O-Methyltransferase Domain Containing 1	0.85565
PPP1R13B-DT	PPP1R13B Divergent Transcript	0.85565
PLCE1-AS1	PLCE1 Antisense RNA 1	0.85498
LINC00682	Long Intergenic Non-Protein Coding RNA 682	0.85489
LINC02590	Long Intergenic Non-Protein Coding RNA 2590	0.85489
LINC02594	Long Intergenic Non-Protein Coding RNA 2594	0.85489
LINC02859	Long Intergenic Non-Protein Coding RNA 2859	0.85489
MIR1229	MicroRNA 1229	0.85332
CMTM5	CKLF Like MARVEL Transmembrane Domain Containing 5	0.85279
SH3D19	SH3 Domain Containing 19	0.85266
SLC22A7	Solute Carrier Family 22 Member 7	0.85252
MIR1258	MicroRNA 1258	0.85091
SAMD1	Sterile Alpha Motif Domain Containing 1	0.85070
PAFAH2	Platelet Activating Factor Acetylhydrolase 2	0.84995
ZNF251	Zinc Finger Protein 251	0.84995
C1orf162	Chromosome 1 Open Reading Frame 162	0.84995
LRRN4CL	LRRN4 C-Terminal Like	0.84995
SNORD35B	Small Nucleolar RNA, C/D Box 35B	0.84995
MYMX	Myomixer, Myoblast Fusion Factor	0.84995
SPDYE17	Speedy/RINGO Cell Cycle Regulator Family Member E17	0.84995
ENSG00000284691		0.84995
SRSF6P2	SRSF6 Pseudogene 2	0.84995
ENSG00000284895		0.84995
PDCD7	Programmed Cell Death 7	0.84927
GJA9	Gap Junction Protein Alpha 9	0.84858
FIBCD1	Fibrinogen C Domain Containing 1	0.84773

ENTPD6	Ectonucleoside Triphosphate Diphosphohydrolase 6	0.84754
ZNF549	Zinc Finger Protein 549	0.84754
ZFR2	Zinc Finger RNA Binding Protein 2	0.84754
OR10H2	Olfactory Receptor Family 10 Subfamily H Member 2	0.84754
LYZL2	Lysozyme Like 2	0.84754
RABL2B	RAB, Member Of RAS Oncogene Family Like 2B	0.84754
OR10H4	Olfactory Receptor Family 10 Subfamily H Member 4	0.84754
NPIPA1	Nuclear Pore Complex Interacting Protein Family Member A1	0.84754
CCER2	Coiled-Coil Glutamate Rich Protein 2	0.84754
SCARNA6	Small Cajal Body-Specific RNA 6	0.84754
SNORD83A	Small Nucleolar RNA, C/D Box 83A	0.84754
MIR12129	MicroRNA 12129	0.84697
UXT	Ubiquitously Expressed Prefoldin Like Chaperone	0.84654
HIRIP3	HIRA Interacting Protein 3	0.84641
SELENOW	Selenoprotein W	0.84635
SLC22A17	Solute Carrier Family 22 Member 17	0.84565
DDX28	DEAD-Box Helicase 28	0.84558
NOCT	Nocturnin	0.84558
TAS2R3	Taste 2 Receptor Member 3	0.84442
DENND4A	DENN Domain Containing 4A	0.84290
RAB33B	RAB33B, Member RAS Oncogene Family	0.84265
TMEM52	Transmembrane Protein 52	0.84185
YBEY	YbeY Metalloendoribonuclease	0.84103
WAKMAR2	Wound And Keratinocyte Migration Associated LncRNA 2	0.84061
MTMR11	Myotubularin Related Protein 11	0.84053
CCDC15	Coiled-Coil Domain Containing 15	0.84038
CYP2D7	Cytochrome P450 Family 2 Subfamily D Member 7 (Gene/Pseudogene)	0.84038
MRPL42	Mitochondrial Ribosomal Protein L42	0.84030
H2BU1	H2B.U Histone 1	0.84030
PLEKHO1	Pleckstrin Homology Domain Containing O1	0.84024
BDH2	3-Hydroxybutyrate Dehydrogenase 2	0.83980
ADGRL1	Adhesion G Protein-Coupled Receptor L1	0.83950
MT1H	Metallothionein 1H	0.83874
TCEAL4	Transcription Elongation Factor A Like 4	0.83824
HFM1	Helicase For Meiosis 1	0.83792
TOMM6	Translocase Of Outer Mitochondrial Membrane 6	0.83482

ZNF767P	Zinc Finger Family Member 767, Pseudogene	0.83482
PDCD2L	Programmed Cell Death 2 Like	0.83403
H2BC18	H2B Clustered Histone 18	0.83340
CHMP2A	Charged Multivesicular Body Protein 2A	0.83215
MIR623	MicroRNA 623	0.82999
KIR3DL3	Killer Cell Immunoglobulin Like Receptor, Three Ig Domains And Long Cytoplasmic Tail 3	0.82998
TRIM41	Tripartite Motif Containing 41	0.82912
RNF121	Ring Finger Protein 121	0.82912
ZNF84	Zinc Finger Protein 84	0.82912
MRPL33	Mitochondrial Ribosomal Protein L33	0.82911
CAMKV	CaM Kinase Like Vesicle Associated	0.82852
ACTR10	Actin Related Protein 10	0.82829
MIR4665	MicroRNA 4665	0.82817
ENHO	Energy Homeostasis Associated	0.82791
MAMSTR	MEF2 Activating Motif And SAP Domain Containing Transcriptional Regulator	0.82759
ZNF578	Zinc Finger Protein 578	0.82759
TMEM207	Transmembrane Protein 207	0.82661
ANKMY1	Ankyrin Repeat And MYND Domain Containing 1	0.82424
LOC11046751[illegible]	CYP1A1 5' Regulatory Region	0.82340
RNF167	Ring Finger Protein 167	0.82318
NRBF2	Nuclear Receptor Binding Factor 2	0.82212
PGLS	6-Phosphogluconolactonase	0.82198
LGI3	Leucine Rich Repeat LGI Family Member 3	0.82198
CA3	Carbonic Anhydrase 3	0.82187
DSCR9	Down Syndrome Critical Region 9	0.82184
LRTOMT	Leucine Rich Transmembrane And O-Methyltransferase Domain Containing	0.82176
LGALS9C	Galectin 9C	0.82138
ARL9	ADP Ribosylation Factor Like GTPase 9	0.82138
THAP9-AS1	THAP9 Antisense RNA 1	0.82138
PHTF2	Putative Homeodomain Transcription Factor 2	0.82053
FOXA3	Forkhead Box A3	0.82024
NDUFAF8	NADH:Ubiquinone Oxidoreductase Complex Assembly Factor 8	0.82024
DHRS7B	Dehydrogenase/Reductase 7B	0.81970
MRM2	Mitochondrial RRNA Methyltransferase 2	0.81970
PCNP	PEST Proteolytic Signal Containing Nuclear Protein	0.81955
ST2	Suppression Of Tumorigenicity 2	0.81909

RAB21	RAB21, Member RAS Oncogene Family	0.81808
MRPL14	Mitochondrial Ribosomal Protein L14	0.81697
MIR4284	MicroRNA 4284	0.81599
HNRNPKP2	Heterogeneous Nuclear Ribonucleoprotein K Pseudogene 2	0.81599
HSPA13	Heat Shock Protein Family A (Hsp70) Member 13	0.81579
CLASP2	Cytoplasmic Linker Associated Protein 2	0.81572
AXDND1	Axonemal Dynein Light Chain Domain Containing 1	0.81565
POLR3F	RNA Polymerase III Subunit F	0.81508
ZNF415	Zinc Finger Protein 415	0.81508
PDIK1L	PDLIM1 Interacting Kinase 1 Like	0.81423
CCDC24	Coiled-Coil Domain Containing 24	0.81423
TOMM7	Translocase Of Outer Mitochondrial Membrane 7	0.81402
PABPC1L	Poly(A) Binding Protein Cytoplasmic 1 Like	0.81402
SYT16	Synaptotagmin 16	0.81401
ENSG00000223440		0.81360
ENSG00000225637		0.81360
CHRD	Chordin	0.81334
RBAKDN	RBAK Downstream Neighbor	0.81091
ENSG00000251246		0.81091
ENSG00000272305		0.81091
EIF5AL1	Eukaryotic Translation Initiation Factor 5A Like 1	0.81044
MIR6778	MicroRNA 6778	0.80888
NSUN5P2	NSUN5 Pseudogene 2	0.80868
GATD1	Glutamine Amidotransferase Like Class 1 Domain Containing 1	0.80772
PLEKHH2	Pleckstrin Homology, MyTH4 And FERM Domain Containing H2	0.80745
PCDHA3	Protocadherin Alpha 3	0.80745
MROH8	Maestro Heat Like Repeat Family Member 8	0.80745
ARRDC1-AS1	ARRDC1 Antisense RNA 1	0.80745
MTRNR2L8	MT-RNR2 Like 8	0.80745
RNASE7	Ribonuclease A Family Member 7	0.80705
PKDCC	Protein Kinase Domain Containing, Cytoplasmic	0.80612
ZNF85	Zinc Finger Protein 85	0.80612
PHOSPHO2	Phosphatase, Orphan 2	0.80612
LGALS9B	Galectin 9B	0.80612
LURAP1L	Leucine Rich Adaptor Protein 1 Like	0.80612
ZBTB41	Zinc Finger And BTB Domain Containing 41	0.80612

LIMS3	LIM Zinc Finger Domain Containing 3	0.80612
ZNF524	Zinc Finger Protein 524	0.80612
SPATA24	Spermatogenesis Associated 24	0.80612
MCUB	Mitochondrial Calcium Uniporter Dominant Negative Subunit Beta	0.80612
CBWD3	COBW Domain Containing 3	0.80612
ECSIT	ECSIT Signaling Integrator	0.80504
C1orf122	Chromosome 1 Open Reading Frame 122	0.80473
SCGB1C1	Secretoglobin Family 1C Member 1	0.80473
SREBF2-AS1	SREBF2 Antisense RNA 1	0.80473
TDRKH	Tudor And KH Domain Containing	0.80353
COMMD4	COMM Domain Containing 4	0.80353
LINC00504	Long Intergenic Non-Protein Coding RNA 504	0.80353
SHPK	Sedoheptulokinase	0.80160
ASCL3	Achaete-Scute Family BHLH Transcription Factor 3	0.80160
AGAP4	ArfGAP With GTPase Domain, Ankyrin Repeat And PH Domain 4	0.80036
CDIPTOSP	CDIP Transferase Opposite Strand, Pseudogene	0.80015
MXD4	MAX Dimerization Protein 4	0.80009
ZFAND5	Zinc Finger AN1-Type Containing 5	0.79982
ZSWIM4	Zinc Finger SWIM-Type Containing 4	0.79982
C19orf25	Chromosome 19 Open Reading Frame 25	0.79982
MIR190B	MicroRNA 190b	0.79850
SNORD105B	Small Nucleolar RNA, C/D Box 105B	0.79824
TBC1D3H	TBC1 Domain Family Member 3H	0.79753
SLC22A20P	Solute Carrier Family 22 Member 20, Pseudogene	0.79753
RILPL2	Rab Interacting Lysosomal Protein Like 2	0.79618
EIF3CL	Eukaryotic Translation Initiation Factor 3 Subunit C Like	0.79618
ACTR3C	Actin Related Protein 3C	0.79532
KIAA1614	KIAA1614	0.79457
TAS1R1	Taste 1 Receptor Member 1	0.79408
PCDHB5	Protocadherin Beta 5	0.79394
FAM86C1P	Family With Sequence Similarity 86 Member C1, Pseudogene	0.79394
MGAT3-AS1	MGAT3 Antisense RNA 1	0.79394
KLHL3	Kelch Like Family Member 3	0.79365
MIR632	MicroRNA 632	0.79321
RCAN2	Regulator Of Calcineurin 2	0.79139
YIPF1	Yip1 Domain Family Member 1	0.79123
BTNL3	Butyrophilin Like 3	0.79123
METTL6	Methyltransferase Like 6	0.79123

ELFN2	Extracellular Leucine Rich Repeat And Fibronectin Type III Domain Containing 2	0.79123
ZNF506	Zinc Finger Protein 506	0.79123
IL17REL	Interleukin 17 Receptor E Like	0.79123
PCDHGB4	Protocadherin Gamma Subfamily B, 4	0.79077
PCDHB12	Protocadherin Beta 12	0.79077
STMND1	Stathmin Domain Containing 1	0.79077
GUSBP2	GUSB Pseudogene 2	0.79077
STAG3L3	Stromal Antigen 3-Like 3 (Pseudogene)	0.79059
MSMO1	Methylsterol Monooxygenase 1	0.78974
OBP2B	Odorant Binding Protein 2B	0.78859
BRSK2	BR Serine/Threonine Kinase 2	0.78842
GSX1	GS Homeobox 1	0.78842
FHDC1	FH2 Domain Containing 1	0.78803
FAM204A	Family With Sequence Similarity 204 Member A	0.78803
MPV17L	MPV17 Mitochondrial Inner Membrane Protein Like	0.78803
C2CD4A	C2 Calcium Dependent Domain Containing 4A	0.78803
C2CD4B	C2 Calcium Dependent Domain Containing 4B	0.78803
EOLA2	Endothelium And Lymphocyte Associated ASCH Domain 2	0.78803
ARHGAP5-AS1	ARHGAP5 Antisense RNA 1 (Head To Head)	0.78803
CEBPZOS	CEBPZ Opposite Strand	0.78803
LINC01551	Long Intergenic Non-Protein Coding RNA 1551	0.78803
LINC00839	Long Intergenic Non-Protein Coding RNA 839	0.78803
ZRANB2-AS2	ZRANB2 Antisense RNA 2 (Head To Head)	0.78803
TAPT1-AS1	TAPT1 Antisense RNA 1 (Head To Head)	0.78803
LINC00595	Long Intergenic Non-Protein Coding RNA 595	0.78803
FAM222A-AS1	FAM222A Antisense RNA 1	0.78803
RBMS3-AS3	RBMS3 Antisense RNA 3	0.78803
NRSN2-AS1	NRSN2 Antisense RNA 1	0.78803
LCMT1-AS1	LCMT1 Antisense RNA 1	0.78803
LINC01479	Long Intergenic Non-Protein Coding RNA 1479	0.78803
UBR5-AS1	UBR5 Antisense RNA 1	0.78803
LOC10050714	Uncharacterized LOC100507144	0.78803
LINC01978	Long Intergenic Non-Protein Coding RNA 1978	0.78803
ZNF433-AS1	ZNF433 And ZNF878 Antisense RNA 1	0.78803

SNHG25	Small Nucleolar RNA Host Gene 25	0.78803
LINC02889	Long Intergenic Non-Protein Coding RNA 2889	0.78803
FAHD2CP	Fumarylacetoacetate Hydrolase Domain Containing 2C, Pseudogene	0.78803
LOC10099643	Uncharacterized LOC100996437	0.78803
LOC10192797	Uncharacterized LOC101927974	0.78803
LINC01977	Long Intergenic Non-Protein Coding RNA 1977	0.78803
LINC02643	Long Intergenic Non-Protein Coding RNA 2643	0.78803
LOC10028956	Uncharacterized LOC100289561	0.78803
LINC02014	Long Intergenic Non-Protein Coding RNA 2014	0.78803
RPL23AP42	Ribosomal Protein L23a Pseudogene 42	0.78803
LOC11226811	Uncharacterized LOC112268114	0.78803
GCSHP5	Glycine Cleavage System Protein H Pseudogene 5	0.78803
RAX2	Retina And Anterior Neural Fold Homeobox 2	0.78786
CAPNS2	Calpain Small Subunit 2	0.78633
DNAJB1P1	DnaJ Heat Shock Protein Family (Hsp40) Member B1 Pseudogene 1	0.78611
COMMD6	COMM Domain Containing 6	0.78566
GAS6-DT	GAS6 Divergent Transcript	0.78548
PLIN1	Perilipin 1	0.78468
KCNJ16	Potassium Inwardly Rectifying Channel Subfamily J Member 16	0.78443
LINC00691	Long Intergenic Non-Protein Coding RNA 691	0.78320
GTF3A	General Transcription Factor IIIA	0.78279
RNF112	Ring Finger Protein 112	0.78257
ZNF208	Zinc Finger Protein 208	0.78204
NETO2	Neuropilin And Tolloid Like 2	0.78203
RNFT2	Ring Finger Protein, Transmembrane 2	0.78194
ZKSCAN2	Zinc Finger With KRAB And SCAN Domains 2	0.78194
LOC10928656	TBX21 Promoter Region	0.78194
B4GALT4	Beta-1,4-Galactosyltransferase 4	0.78173
TTC5	Tetratricopeptide Repeat Domain 5	0.78173
ARFIP1	ADP Ribosylation Factor Interacting Protein 1	0.78173
ZNF12	Zinc Finger Protein 12	0.78173
TRIM10	Tripartite Motif Containing 10	0.78173
CMTR1	Cap Methyltransferase 1	0.78173
MRFAP1L1	Morf4 Family Associated Protein 1 Like 1	0.78173
EXO5	Exonuclease 5	0.78173
CCDC186	Coiled-Coil Domain Containing 186	0.78173

RPL13P12	Ribosomal Protein L13 Pseudogene 12	0.78173
MIR6732	MicroRNA 6732	0.78173
POU5F1P6	POU Class 5 Homeobox 1 Pseudogene 6	0.78173
PAIP2	Poly(A) Binding Protein Interacting Protein 2	0.78114
BTN2A1	Butyrophilin Subfamily 2 Member A1	0.78114
TRAPPC6A	Trafficking Protein Particle Complex Subunit 6A	0.78114
KLHL5	Kelch Like Family Member 5	0.78114
FCF1	FCF1 RRNA-Processing Protein	0.78114
TIGD7	Tigger Transposable Element Derived 7	0.78114
RELL2	RELT Like 2	0.78114
M1AP	Meiosis 1 Associated Protein	0.78114
C9orf47	Chromosome 9 Open Reading Frame 47	0.78114
IFITM10	Interferon Induced Transmembrane Protein 10	0.78114
ZNF705E	Zinc Finger Protein 705E	0.78114
ID2-AS1	ID2 Antisense RNA 1	0.78114
TSSC2	Tumor Suppressing Subtransferable Candidate 2 (Pseudogene)	0.78114
MIR5581	MicroRNA 5581	0.78114
IGFBPL1	Insulin Like Growth Factor Binding Protein Like 1	0.78106
ZNF544	Zinc Finger Protein 544	0.78106
BNIPL	BCL2 Interacting Protein Like	0.78106
MIR23AHG	MiR-23a/27a/24-2 Cluster Host Gene	0.78019
ISOC1	Isochorismatase Domain Containing 1	0.77980
CYP46A1	Cytochrome P450 Family 46 Subfamily A Member 1	0.77653
PM20D1	Peptidase M20 Domain Containing 1	0.77653
ANO2	Anoctamin 2	0.77631
SLC15A2	Solute Carrier Family 15 Member 2	0.77589
KCNH6	Potassium Voltage-Gated Channel Subfamily H Member 6	0.77471
CHMP1A	Charged Multivesicular Body Protein 1A	0.77471
SGF29	SAGA Complex Associated Factor 29	0.77471
MIR3178	MicroRNA 3178	0.77356
SYCN	Syncollin	0.77316
SP5	Sp5 Transcription Factor	0.77254
ENSG00000273088		0.77192
LRRC2	Leucine Rich Repeat Containing 2	0.77094
R3HDM4	R3H Domain Containing 4	0.77094
LINC02904	Long Intergenic Non-Protein Coding RNA 2904	0.77094
CHST12	Carbohydrate Sulfotransferase 12	0.77065
ADAM21	ADAM Metallopeptidase Domain 21	0.77065

LILRA6	Leukocyte Immunoglobulin Like Receptor A6	0.77065
ZNF468	Zinc Finger Protein 468	0.77065
NCAM1-AS1	NCAM1 Antisense RNA1	0.77065
RNF139-AS1	RNF139 Antisense RNA 1 (Head To Head)	0.77065
ADAM20P1	ADAM Metallopeptidase Domain 20 Pseudogene 1	0.77065
BAZ1A-AS1	BAZ1A Antisense RNA 1	0.77065
LINC01921	Long Intergenic Non-Protein Coding RNA 1921	0.77065
FTOP1	FTO Pseudogene 1	0.77065
LOC10537387	Uncharacterized LOC105373876	0.77065
ENSG00000261207		0.77065
ENSG00000231597		0.77065
ENSG00000227021		0.77065
ENSG00000271151		0.77065
ENSG00000223374		0.77065
BICD1P1	BICD Cargo Adaptor 1 Pseudogene 1	0.77065
ENSG00000253106		0.77065
ENSG00000274370		0.77065
lnc-DCST2-2		0.77065
lnc-TTLL1-2		0.77065
ENSG00000269001		0.77065
HSALNG0007458		0.77065
L13304-023		0.77065
NONHSAG003042.2		0.77065
RF00017-3460		0.77065
HSALNG0119503		0.77065
HSALNG0131520		0.77065
HSALNG0131515		0.77065
RF00017-3298		0.77065
LOC10798725	Uncharacterized LOC107987251	0.77065
HSALNG0124634		0.77065
HSALNG0100141		0.77065
LRRN1	Leucine Rich Repeat Neuronal 1	0.76988
MIPEPP2	Mitochondrial Intermediate Peptidase Pseudogene 2	0.76988
MEP1A	Meprin A Subunit Alpha	0.76920
DOK4	Docking Protein 4	0.76901
RBM4B	RNA Binding Motif Protein 4B	0.76901
LIF-AS1	LIF Antisense RNA 1	0.76901
CLEC5A	C-Type Lectin Domain Containing 5A	0.76809
TXLNB	Taxilin Beta	0.76702
ISM1	Isthmin 1	0.76700
TEX22	Testis Expressed 22	0.76539
IL34	Interleukin 34	0.76522
CKMT2	Creatine Kinase, Mitochondrial 2	0.76512
CYTH4	Cytohesin 4	0.76457

ORMDL2	ORMDL Sphingolipid Biosynthesis Regulator 2	0.76457
THAP3	THAP Domain Containing 3	0.76457
VSIG8	V-Set And Immunoglobulin Domain Containing 8	0.76457
ZSWIM1	Zinc Finger SWIM-Type Containing 1	0.76457
PGM5	Phosphoglucomutase 5	0.76447
RASAL3	RAS Protein Activator Like 3	0.76447
ZFP14	ZFP14 Zinc Finger Protein	0.76447
PCDHGA9	Protocadherin Gamma Subfamily A, 9	0.76447
SFTA2	Surfactant Associated 2	0.76447
STIMATE	STIM Activating Enhancer	0.76447
MST1L	Macrophage Stimulating 1 Like (Pseudogene)	0.76447
ZNF436-AS1	ZNF436 Antisense RNA 1	0.76447
LOC10013331	XRCC1 N-Terminal Domain Containing 1-Like	0.76447
RNF216P1	Ring Finger Protein 216 Pseudogene 1	0.76447
H2BC19P	H2B Clustered Histone 19, Pseudogene	0.76447
RNF185-AS1	RNF185 Antisense RNA 1	0.76447
NINJ2-AS1	NINJ2 Antisense RNA 1	0.76447
GTF2IP20	General Transcription Factor IIi Pseudogene 20	0.76447
ENSG00000236782		0.76447
ENSG00000286112		0.76447
ENSG00000288681		0.76447
GTF2IRD2P1	GTF2I Repeat Domain Containing 2 Pseudogene 1	0.76431
MT1JP	Metallothionein 1J, Pseudogene	0.76335
MIR644A	MicroRNA 644a	0.76335
LPGAT1	Lysophosphatidylglycerol Acyltransferase 1	0.76308
MPHOSPH6	M-Phase Phosphoprotein 6	0.76308
SNRPGP15	Small Nuclear Ribonucleoprotein Polypeptide G Pseudogene 15	0.76308
ACR	Acrosin	0.76281
AMER3	APC Membrane Recruitment Protein 3	0.76270
SLCO4A1-AS1	SLCO4A1 Antisense RNA 1	0.75939
MIR512-2	MicroRNA 512-2	0.75930
RHOU	Ras Homolog Family Member U	0.75898
TIMM10B	Translocase Of Inner Mitochondrial Membrane 10B	0.75875
CASKIN1	CASK Interacting Protein 1	0.75814
NME1-NME2	NME1-NME2 Readthrough	0.75814
NUGGC	Nuclear GTPase, Germinal Center Associated	0.75814
SDHAP3	Succinate Dehydrogenase Complex Flavoprotein Subunit A Pseudogene 3	0.75814
VNN2	Vanin 2	0.75797

AK5	Adenylate Kinase 5	0.75730
FAM3A	FAM3 Metabolism Regulating Signaling Molecule A	0.75686
HMCES	5-Hydroxymethylcytosine Binding, ES Cell Specific	0.75686
MMP23A	Matrix Metallopeptidase 23A (Pseudogene)	0.75686
OSBPL10	Oxysterol Binding Protein Like 10	0.75644
PCDHB9	Protocadherin Beta 9	0.75644
BFAR	Bifunctional Apoptosis Regulator	0.75624
SLC2A13	Solute Carrier Family 2 Member 13	0.75510
KASH5	KASH Domain Containing 5	0.75506
MIR4258	MicroRNA 4258	0.75317
MIR1265	MicroRNA 1265	0.75276
PHLDB2	Pleckstrin Homology Like Domain Family B Member 2	0.75167
DOC2A	Double C2 Domain Alpha	0.75051
KCNV1	Potassium Voltage-Gated Channel Modifier Subfamily V Member 1	0.75051
TIGD4	Tigger Transposable Element Derived 4	0.75051
DEFB135	Defensin Beta 135	0.75051
ARMH3	Armadillo Like Helical Domain Containing 3	0.75051
DNAAF8	Dynein Axonemal Assembly Factor 8	0.75051
PKD1P2	Polycystin 1, Transient Receptor Potential Channel Interacting Pseudogene 2	0.75051
PKD1P3	Polycystin 1, Transient Receptor Potential Channel Interacting Pseudogene 3	0.75051
LOC10050647	Uncharacterized LOC100506472	0.75051
MT1B	Metallothionein 1B	0.75033
BOLA2	BolA Family Member 2	0.75033
XK	X-Linked Kx Blood Group	0.74977
TSPAN5	Tetraspanin 5	0.74909
PMS2P5	PMS1 Homolog 2, Mismatch Repair System Component Pseudogene 5	0.74796
LINC00163	Long Intergenic Non-Protein Coding RNA 163	0.74450
LINC00165	Long Intergenic Non-Protein Coding RNA 165	0.74450
MED30	Mediator Complex Subunit 30	0.74441
TRAPPC14	Trafficking Protein Particle Complex Subunit 14	0.74428
TEDC1	Tubulin Epsilon And Delta Complex 1	0.74428
PLPP7	Phospholipid Phosphatase 7 (Inactive)	0.74344
DEDD2	Death Effector Domain Containing 2	0.74342
AGAP6	ArfGAP With GTPase Domain, Ankyrin Repeat And PH Domain 6	0.74342

ANKHD1-EIF4	ANKHD1-EIF4EBP3 Readthrough	0.74342
DZIP3	DAZ Interacting Zinc Finger Protein 3	0.74239
MIR3189	MicroRNA 3189	0.74239
MPP7	Membrane Palmitoylated Protein 7	0.74174
TLCD1	TLC Domain Containing 1	0.74147
FKBP6	FKBP Prolyl Isomerase Family Member 6 (Inactive)	0.73855
TAS1R2	Taste 1 Receptor Member 2	0.73611
SYNGR3	Synaptogyrin 3	0.73607
MIR4466	MicroRNA 4466	0.73495
RIT2	Ras Like Without CAAX 2	0.73405
ASB16	Ankyrin Repeat And SOCS Box Containing 16	0.73370
MEX3C	Mex-3 RNA Binding Family Member C	0.73079
ECEL1	Endothelin Converting Enzyme Like 1	0.73033
EF177379		0.72879
AP1G1	Adaptor Related Protein Complex 1 Subunit Gamma 1	0.72794
CYP20A1	Cytochrome P450 Family 20 Subfamily A Member 1	0.72780
TCTE1	T-Complex-Associated-Testis-Expressed 1	0.72676
PILRA	Paired Immunoglobin Like Type 2 Receptor Alpha	0.72676
BOLA1	BolA Family Member 1	0.72580
FAM32A	Family With Sequence Similarity 32 Member A	0.72580
SERTAD2	SERTA Domain Containing 2	0.72580
H2BC6	H2B Clustered Histone 6	0.72580
RAB29	RAB29, Member RAS Oncogene Family	0.72544
ELOF1	Elongation Factor 1 Homolog	0.72326
LOC10835381'	MIR1254-2-OTUD1 Intergenic CAGE-Defined Monocyte Enhancer	0.72309
GLB1L	Galactosidase Beta 1 Like	0.72294
KIR2DS1	Killer Cell Immunoglobulin Like Receptor, Two Ig Domains And Short Cytoplasmic Tail 1	0.72117
MT1L	Metallothionein 1L, Pseudogene	0.71957
MRGPRG-AS1	MRGPRG Antisense RNA 1	0.71594
BEX4	Brain Expressed X-Linked 4	0.71529
KCNJ4	Potassium Inwardly Rectifying Channel Subfamily J Member 4	0.71481
MOV10L1	Mov10 Like RISC Complex RNA Helicase 1	0.71481
INSM2	INSM Transcriptional Repressor 2	0.71401
SLFN12L	Schlafen Family Member 12 Like	0.71296
DLEU1-AS1	DLEU1 Antisense RNA 1	0.71230

PABIR1	PP2A Aalpha (PPP2R1A) And B55A (PPP2R2A) Interacting Phosphatase Regulator 1	0.71226
AARSD1	Alanyl-TRNA Synthetase Domain Containing 1	0.71007
ZNF777	Zinc Finger Protein 777	0.71007
CFAP45	Cilia And Flagella Associated Protein 45	0.71007
SLC7A3	Solute Carrier Family 7 Member 3	0.70985
IL27RA	Interleukin 27 Receptor Subunit Alpha	0.70770
GOLGA1	Golgin A1	0.70770
UNCX	UNC Homeobox	0.70770
LINC02575	Long Intergenic Non-Protein Coding RNA 2575	0.70770
NFE2L1-DT	NFE2L1 Divergent Transcript	0.70770
LINC01703	Long Intergenic Non-Protein Coding RNA 1703	0.70770
MIR10523	MicroRNA 10523	0.70770
IGDCC3	Immunoglobulin Superfamily DCC Subclass Member 3	0.70737
KIF18B	Kinesin Family Member 18B	0.70714
MIR4269	MicroRNA 4269	0.70670
H2BC1	H2B Clustered Histone 1	0.70591
STAC2	SH3 And Cysteine Rich Domain 2	0.70515
ACSM4	Acyl-CoA Synthetase Medium Chain Family Member 4	0.70515
MARCHF9	Membrane Associated Ring-CH-Type Finger 9	0.70515
PRAG1	PEAK1 Related, Kinase-Activating Pseudokinase 1	0.70422
CHAC2	ChaC Glutathione Specific Gamma-Glutamylcyclotransferase 2	0.70043
LINC02163	Long Intergenic Non-Protein Coding RNA 2163	0.69951
HSP90B2P	Heat Shock Protein 90 Beta Family Member 2, Pseudogene	0.69744
TPM3P4	Tropomyosin 3 Pseudogene 4	0.69744
SIRPB1	Signal Regulatory Protein Beta 1	0.69691
ZNF140	Zinc Finger Protein 140	0.69691
MRPL51	Mitochondrial Ribosomal Protein L51	0.69691
KRT36	Keratin 36	0.69691
LDLRAD2	Low Density Lipoprotein Receptor Class A Domain Containing 2	0.69691
PCDHGA4	Protocadherin Gamma Subfamily A, 4	0.69691
MIR4645	MicroRNA 4645	0.69691
MIR4734	MicroRNA 4734	0.69691
MIR4802	MicroRNA 4802	0.69691
MIR4692	MicroRNA 4692	0.69691
MIR4748	MicroRNA 4748	0.69691
RNU2-4P	RNA, U2 Small Nuclear 4, Pseudogene	0.69691

CSN3	Casein Kappa	0.69337
KCNK17	Potassium Two Pore Domain Channel Subfamily K Member 17	0.69236
FXYD7	FXYD Domain Containing Ion Transport Regulator 7	0.69236
PCDHA12	Protocadherin Alpha 12	0.69236
PCDHAC2	Protocadherin Alpha Subfamily C, 2	0.69236
PCDHA10	Protocadherin Alpha 10	0.69236
RAB42	RAB42, Member RAS Oncogene Family	0.69236
PCDHAC1	Protocadherin Alpha Subfamily C, 1	0.69236
NATD1	N-Acetyltransferase Domain Containing 1	0.69236
PPFIA4	PTPRF Interacting Protein Alpha 4	0.69068
FAM200A	Family With Sequence Similarity 200 Member A	0.69068
HTR7	5-Hydroxytryptamine Receptor 7	0.69026
MIDN	Midnolin	0.68651
RD3L	RD3 Like	0.68651
LINC00619	Long Intergenic Non-Protein Coding RNA 619	0.68641
GTF3C6	General Transcription Factor IIIC Subunit 6	0.68535
KDELR3	KDEL Endoplasmic Reticulum Protein Retention Receptor 3	0.68474
MT1M	Metallothionein 1M	0.68474
MIR6893	MicroRNA 6893	0.68474
CRYGD	Crystallin Gamma D	0.68454
GPR61	G Protein-Coupled Receptor 61	0.68412
MTG1	Mitochondrial Ribosome Associated GTPase 1	0.68412
PCDHB11	Protocadherin Beta 11	0.68412
TATDN2	TatD DNase Domain Containing 2	0.68412
PCDHGA2	Protocadherin Gamma Subfamily A, 2	0.68412
CIB3	Calcium And Integrin Binding Family Member 3	0.68412
KRTAP12-4	Keratin Associated Protein 12-4	0.68412
ZNF26	Zinc Finger Protein 26	0.68412
ZNF765	Zinc Finger Protein 765	0.68412
ZDHHC22	Zinc Finger DHHC-Type Palmitoyltransferase 22	0.68412
LRRC73	Leucine Rich Repeat Containing 73	0.68412
LRRC75A	Leucine Rich Repeat Containing 75A	0.68412
ARPC4-TTLL3	ARPC4-TTLL3 Readthrough	0.68412
BTN2A3P	Butyrophilin Subfamily 2 Member A3, Pseudogene	0.68412
OBSCN-AS1	OBSCN Antisense RNA 1	0.68412
FAM99B	Family With Sequence Similarity 99 Member B	0.68412

ZNF860	Zinc Finger Protein 860	0.68393
TMEM100	Transmembrane Protein 100	0.68244
REG1B	Regenerating Family Member 1 Beta	0.67949
CPNE2	Copine 2	0.67853
FCRL6	Fc Receptor Like 6	0.67853
TMEM242	Transmembrane Protein 242	0.67853
GASAL1	Growth Arrest Associated LncRNA 1	0.67853
ANKS3	Ankyrin Repeat And Sterile Alpha Motif Domain Containing 3	0.67568
HES3	Hes Family BHLH Transcription Factor 3	0.67544
LOC11267920	ABO Promoter Region	0.67532
PIWIL3	Piwi Like RNA-Mediated Gene Silencing 3	0.67443
UBE2E1	Ubiquitin Conjugating Enzyme E2 E1	0.67402
PCYOX1	Prenylcysteine Oxidase 1	0.67200
ZNF589	Zinc Finger Protein 589	0.67200
VPS26C	VPS26 Endosomal Protein Sorting Factor C	0.67185
LOC10537434	Uncharacterized LOC105374344	0.67185
MMP23B	Matrix Metallopeptidase 23B	0.67114
ARV1	ARV1 Homolog, Fatty Acid Homeostasis Modulator	0.67095
GJA3	Gap Junction Protein Alpha 3	0.66844
GGT2	Gamma-Glutamyltransferase 2	0.66817
UQCR11	Ubiquinol-Cytochrome C Reductase, Complex III Subunit XI	0.66744
LHFPL4	LHFPL Tetraspan Subfamily Member 4	0.66744
XKR6	XK Related 6	0.66744
ZNF554	Zinc Finger Protein 554	0.66744
TIGD3	Tigger Transposable Element Derived 3	0.66744
PCDHB8	Protocadherin Beta 8	0.66744
ZNF319	Zinc Finger Protein 319	0.66744
PCDHB13	Protocadherin Beta 13	0.66744
SNRNP35	Small Nuclear Ribonucleoprotein U11/U12 Subunit 35	0.66744
KIAA1549L	KIAA1549 Like	0.66744
SPINK8	Serine Peptidase Inhibitor Kazal Type 8 (Putative)	0.66744
KRTAP5-5	Keratin Associated Protein 5-5	0.66744
C9orf163	Chromosome 9 Putative Open Reading Frame 163	0.66744
PPP5D1	PPP5 Tetratricopeptide Repeat Domain Containing 1	0.66744
PCDHB18P	Protocadherin Beta 18 Pseudogene	0.66744
MIR3138	MicroRNA 3138	0.66744
EIF1AXP1	EIF1AX Pseudogene 1	0.66744
ENSG00000250644		0.66744

MDFIC	MyoD Family Inhibitor Domain Containing	0.66656
CELSR3	Cadherin EGF LAG Seven-Pass G-Type Receptor 3	0.66549
RABEPK	Rab9 Effector Protein With Kelch Motifs	0.66509
CHID1	Chitinase Domain Containing 1	0.66509
TMEM120B	Transmembrane Protein 120B	0.66509
ANAPC15	Anaphase Promoting Complex Subunit 15	0.66509
LOC10537299(	Uncharacterized LOC105372990	0.66509
TMED6	Transmembrane P24 Trafficking Protein 6	0.66425
MATN4	Matrilin 4	0.66419
MIR2392	MicroRNA 2392	0.66364
UBE2O	Ubiquitin Conjugating Enzyme E2 O	0.66289
MIR922	MicroRNA 922	0.66285
ADM2	Adrenomedullin 2	0.66206
LOC11059956'	D7S22 Minisatellite Repeat Instability Region	0.65922
MPZL2	Myelin Protein Zero Like 2	0.65773
MAPK8IP2	Mitogen-Activated Protein Kinase 8 Interacting Protein 2	0.65773
EFR3B	EFR3 Homolog B	0.65773
CLBA1	Clathrin Binding Box Of Aftiphilin Containing 1	0.65773
ATP1B4	ATPase Na+/K+ Transporting Family Member Beta 4	0.65675
SDR16C5	Short Chain Dehydrogenase/Reductase Family 16C Member 5	0.65448
SCNN1D	Sodium Channel Epithelial 1 Subunit Delta	0.65330
DNAJC30	DnaJ Heat Shock Protein Family (Hsp40) Member C30	0.65256
RNF17	Ring Finger Protein 17	0.65034
SRSF12	Serine And Arginine Rich Splicing Factor 12	0.64983
RESP18	Regulated Endocrine Specific Protein 18	0.64983
ZSCAN1	Zinc Finger And SCAN Domain Containing 1	0.64983
NKAIN4	Sodium/Potassium Transporting ATPase Interacting 4	0.64983
MIR6743	MicroRNA 6743	0.64983
MALRD1	MAM And LDL Receptor Class A Domain Containing 1	0.64941
MCHR1	Melanin Concentrating Hormone Receptor 1	0.64714
SNORD95	Small Nucleolar RNA, C/D Box 95	0.64545

TMEM52B	Transmembrane Protein 52B	0.64476
MYBPC2	Myosin Binding Protein C2	0.64428
SLC35B1	Solute Carrier Family 35 Member B1	0.64124
SEMA7A	Semaphorin 7A (John Milton Hagen Blood Group)	0.64080
RABAC1	Rab Acceptor 1	0.64035
SCMH1	Scm Polycomb Group Protein Homolog 1	0.63940
NDUFS5	NADH:Ubiquinone Oxidoreductase Subunit S5	0.63869
NCAPG2	Non-SMC Condensin II Complex Subunit G2	0.63790
SLC25A32	Solute Carrier Family 25 Member 32	0.63787
BHLHA9	Basic Helix-Loop-Helix Family Member A9	0.63787
DUSP5P1	Dual Specificity Phosphatase 5 Pseudogene 1	0.63653
ZDHHC15	Zinc Finger DHHC-Type Palmitoyltransferase 15	0.63542
TMEM38A	Transmembrane Protein 38A	0.63542
CD300C	CD300c Molecule	0.63542
IQSEC3	IQ Motif And Sec7 Domain ArfGEF 3	0.63542
PHYHIP	Phytanoyl-CoA 2-Hydroxylase Interacting Protein	0.63542
SP6	Sp6 Transcription Factor	0.63542
IRX6	Iroquois Homeobox 6	0.63542
PLIN5	Perilipin 5	0.63542
ACSM6	Acyl-CoA Synthetase Medium Chain Family Member 6	0.63542
ANKRD31	Ankyrin Repeat Domain 31	0.63542
SYNDIG1L	Synapse Differentiation Inducing 1 Like	0.63542
CFAP61	Cilia And Flagella Associated Protein 61	0.63542
RPEL1	Ribulose-5-Phosphate-3-Epimerase Like 1	0.63542
HTT-AS	HTT Antisense RNA	0.63542
SNORA59A	Small Nucleolar RNA, H/ACA Box 59A	0.63542
P2RX5-TAX1B	P2RX5-TAX1BP3 Readthrough (NMD Candidate)	0.63542
TNFRSF14-AS	TNFRSF14 Antisense RNA 1	0.63542
ENSG00000241269		0.63542
ENSG00000278924		0.63542
GRAP	GRB2 Related Adaptor Protein	0.63513
COLEC12	Collectin Subfamily Member 12	0.63464
XIAP-AS1	XIAP Antisense RNA 1	0.63417
LINC00470	Long Intergenic Non-Protein Coding RNA 470	0.63397
SLC24A3	Solute Carrier Family 24 Member 3	0.63318
SLC22A14	Solute Carrier Family 22 Member 14	0.63287

LINC02864	Long Intergenic Non-Protein Coding RNA 2864	0.63234
C17orf75	Chromosome 17 Open Reading Frame 75	0.63174
RNF208	Ring Finger Protein 208	0.63174
MIR6734	MicroRNA 6734	0.63174
NSRP1	Nuclear Speckle Splicing Regulatory Protein 1	0.62991
PIP4P1	Phosphatidylinositol-4,5-Bisphosphate 4-Phosphatase 1	0.62596
LNCOG	LncRNA Osteogenesis Associated	0.62580
HOXD8	Homeobox D8	0.62535
CASQ2	Calsequestrin 2	0.62311
MIR6730	MicroRNA 6730	0.62269
CPXM2	Carboxypeptidase X, M14 Family Member 2	0.62117
UAP1L1	UDP-N-Acetylglucosamine Pyrophosphorylase 1 Like 1	0.62117
SLC25A5-AS1	SLC25A5 Antisense RNA 1	0.62117
MIR6506	MicroRNA 6506	0.62117
METTL7B	Methyltransferase Like 7B	0.62095
SNX22	Sorting Nexin 22	0.62095
SLC10A3	Solute Carrier Family 10 Member 3	0.62095
C1orf131	Chromosome 1 Open Reading Frame 131	0.62095
PCDHGC3	Protocadherin Gamma Subfamily C, 3	0.61861
ANO3	Anoctamin 3	0.61140
ISM2	Isthmin 2	0.61124
TNNC2	Troponin C2, Fast Skeletal Type	0.61098
KCTD4	Potassium Channel Tetramerization Domain Containing 4	0.61071
ARL10	ADP Ribosylation Factor Like GTPase 10	0.61071
XS	X-Linked Suppressor Of LU Antigens	0.60913
IGFL4	IGF Like Family Member 4	0.60815
ZDHHC8P1	ZDHHC8 Pseudogene 1	0.60815
MIR1255A	MicroRNA 1255a	0.60815
KRT37	Keratin 37	0.60546
BORA	BORA Aurora Kinase A Activator	0.60455
MIR548G	MicroRNA 548g	0.60450
LINC01606	Long Intergenic Non-Protein Coding RNA 1606	0.60450
IGFL2-AS1	IGFL2 Antisense RNA 1	0.60450
MIR5003	MicroRNA 5003	0.60450
REEP2	Receptor Accessory Protein 2	0.60352
CLVS1	Clavesin 1	0.60351
EDEM2	ER Degradation Enhancing Alpha-Mannosidase Like Protein 2	0.60212

TRNAU1AP	TRNA Selenocysteine 1 Associated Protein 1	0.60212
ANKRD26P1	Ankyrin Repeat Domain 26 Pseudogene 1	0.60212
MIR4725	MicroRNA 4725	0.60212
GCA	Grancalcin	0.60147
CPTP	Ceramide-1-Phosphate Transfer Protein	0.60147
TRIM74	Tripartite Motif Containing 74	0.60147
HPYR1	Helicobacter Pylori Responsive 1	0.59993
SLCO3A1	Solute Carrier Organic Anion Transporter Family Member 3A1	0.59620
SUCNR1	Succinate Receptor 1	0.59593
RAB11FIP5	RAB11 Family Interacting Protein 5	0.59471
GPHA2	Glycoprotein Hormone Subunit Alpha 2	0.59345
RPTN	Repetin	0.59345
SSC5D	Scavenger Receptor Cysteine Rich Family Member With 5 Domains	0.59345
ZFP2	ZFP2 Zinc Finger Protein	0.59261
ZNF454	Zinc Finger Protein 454	0.59261
RPL41	Ribosomal Protein L41	0.59261
TMEM88B	Transmembrane Protein 88B	0.59261
MIR200CHG	MIR200C And MIR141 Host Gene	0.59261
PNRC1	Proline Rich Nuclear Receptor Coactivator 1	0.59195
CLEC4F	C-Type Lectin Domain Family 4 Member F	0.59148
PCIF1	Phosphorylated CTD Interacting Factor 1	0.59148
ZNF518B	Zinc Finger Protein 518B	0.59148
MRPS31P5	Mitochondrial Ribosomal Protein S31 Pseudogene 5	0.59148
TMEM25	Transmembrane Protein 25	0.58620
HES4	Hes Family BHLH Transcription Factor 4	0.58580
TM2D3	TM2 Domain Containing 3	0.58561
CMTM4	CKLF Like MARVEL Transmembrane Domain Containing 4	0.58515
MFSD8	Major Facilitator Superfamily Domain Containing 8	0.58435
AKNAD1	AKNA Domain Containing 1	0.58405
AIDA	Axin Interactor, Dorsalization Associated	0.58182
RNF175	Ring Finger Protein 175	0.58182
ZNF829	Zinc Finger Protein 829	0.58182
CYP27C1	Cytochrome P450 Family 27 Subfamily C Member 1	0.58182
TPRG1L	Tumor Protein P63 Regulated 1 Like	0.58182
ZNF865	Zinc Finger Protein 865	0.58182
MIR4632	MicroRNA 4632	0.58182

MIR4648	MicroRNA 4648	0.58182
MIR4710	MicroRNA 4710	0.58182
MIR4489	MicroRNA 4489	0.58182
UBTD1	Ubiquitin Domain Containing 1	0.58152
SYNE4	Spectrin Repeat Containing Nuclear Envelope Family Member 4	0.57789
AQP10	Aquaporin 10	0.57678
LEXM	Lymphocyte Expansion Molecule	0.57678
STPG3	Sperm-Tail PG-Rich Repeat Containing 3	0.57678
SNHG28	Small Nucleolar RNA Host Gene 28	0.57678
DUS4L-BCAP2	DUS4L-BCAP29 Readthrough	0.57671
NCF1C	Neutrophil Cytosolic Factor 1C Pseudogene	0.57628
REM1	RRAD And GEM Like GTPase 1	0.57571
LRRC37B	Leucine Rich Repeat Containing 37B	0.57571
CD8B	CD8b Molecule	0.57227
RPPH1	Ribonuclease P RNA Component H1	0.56999
SLC25A23	Solute Carrier Family 25 Member 23	0.56903
ASB13	Ankyrin Repeat And SOCS Box Containing 13	0.56903
C6orf89	Chromosome 6 Open Reading Frame 89	0.56903
KLHDC3	Kelch Domain Containing 3	0.56903
ZNF567	Zinc Finger Protein 567	0.56903
ZNF683	Zinc Finger Protein 683	0.56903
VSIG10	V-Set And Immunoglobulin Domain Containing 10	0.56903
ZNF114	Zinc Finger Protein 114	0.56903
RTP2	Receptor Transporter Protein 2	0.56903
ZNF594	Zinc Finger Protein 594	0.56903
C1orf54	Chromosome 1 Open Reading Frame 54	0.56903
ZNF385C	Zinc Finger Protein 385C	0.56903
KRTAP5-6	Keratin Associated Protein 5-6	0.56903
C6orf226	Chromosome 6 Open Reading Frame 226	0.56903
GRIFIN	Galectin-Related Inter-Fiber Protein	0.56903
PCBP1-AS1	PCBP1 Antisense RNA 1	0.56903
PCDHB17P	Protocadherin Beta 17 Pseudogene	0.56903
LINC02913	Long Intergenic Non-Protein Coding RNA 2913	0.56903
SNORA79B	Small Nucleolar RNA, H/ACA Box 79B	0.56903
GNAT3	G Protein Subunit Alpha Transducin 3	0.56692
PMM1	Phosphomannomutase 1	0.56595
SHE	Src Homology 2 Domain Containing E	0.56595
NCF1B	Neutrophil Cytosolic Factor 1B Pseudogene	0.56348
TMEM270	Transmembrane Protein 270	0.56121
STAG3L4	Stromal Antigen 3-Like 4 (Pseudogene)	0.56121

SPDYE7P	Speedy/RINGO Cell Cycle Regulator Family Member E7, Pseudogene	0.56121
SPDYE8	Speedy/RINGO Cell Cycle Regulator Family Member E8	0.56121
SPDYE15	Speedy/RINGO Cell Cycle Regulator Family Member E15	0.56121
SPDYE13	Speedy/RINGO Cell Cycle Regulator Family Member E13	0.56121
SPDYE9	Speedy/RINGO Cell Cycle Regulator Family Member E9	0.56121
WBSCR23	Williams-Beuren Syndrome Chromosome Region 23	0.56121
C3orf33	Chromosome 3 Open Reading Frame 33	0.55759
SP8	Sp8 Transcription Factor	0.55629
SHLD2	Shieldin Complex Subunit 2	0.55518
WFDC12	WAP Four-Disulfide Core Domain 12	0.55516
ENSG00000254192		0.55516
ISLR2	Immunoglobulin Superfamily Containing Leucine Rich Repeat 2	0.55235
RTP4	Receptor Transporter Protein 4	0.55235
FICD	FIC Domain Protein Adenylyltransferase	0.55235
PCDHB10	Protocadherin Beta 10	0.55235
PCDHGB2	Protocadherin Gamma Subfamily B, 2	0.55235
ARHGEF33	Rho Guanine Nucleotide Exchange Factor 33	0.55235
OR56A1	Olfactory Receptor Family 56 Subfamily A Member 1	0.55235
TEX38	Testis Expressed 38	0.55235
BBOF1	Basal Body Orientation Factor 1	0.55235
UQCRHL	Ubiquinol-Cytochrome C Reductase Hinge Protein Like	0.55235
TMEM250	Transmembrane Protein 250	0.55235
KRT87P	Keratin 87 Pseudogene	0.55235
SNORA4	Small Nucleolar RNA, H/ACA Box 4	0.55235
SNRPEP2	SNRPE Pseudogene 2	0.55235
MIR6076	MicroRNA 6076	0.55235
PTBP1P	Polypyrimidine Tract Binding Protein 1 Pseudogene	0.55235
ENSG00000255508		0.55235
ENSG00000280778		0.55235
ENSG00000285976		0.55235
ENSG00000285938		0.55235
NRAP	Nebulin Related Anchoring Protein	0.55062
ANP32A-IT1	ANP32A Intronic Transcript 1	0.54852
COL18A1-AS2	COL18A1 Antisense RNA 2	0.54852
PPM1A	Protein Phosphatase, Mg2+/Mn2+ Dependent 1A	0.54827

MAP2K4P1	Mitogen-Activated Protein Kinase Kinase 4 Pseudogene 1	0.54419
RSC1A1	Regulator Of Solute Carriers 1	0.54237
ZNF581	Zinc Finger Protein 581	0.54237
PCDHA11	Protocadherin Alpha 11	0.54237
ZNF846	Zinc Finger Protein 846	0.54237
DHRS12	Dehydrogenase/Reductase 12	0.54237
LINC02570	Long Intergenic Non-Protein Coding RNA 2570	0.54237
CCDC116	Coiled-Coil Domain Containing 116	0.53956
CCDC77	Coiled-Coil Domain Containing 77	0.53652
MT4	Metallothionein 4	0.53652
HACD4	3-Hydroxyacyl-CoA Dehydratase 4	0.53286
DPP7	Dipeptidyl Peptidase 7	0.53271
FLVCR1-DT	FLVCR1 Divergent Transcript	0.53271
GGT3P	Gamma-Glutamyltransferase 3 Pseudogene	0.53187
FKBP1B	FKBP Prolyl Isomerase 1B	0.53136
BCAP29	B Cell Receptor Associated Protein 29	0.52979
RNR2	RNA, Ribosomal 45S Cluster 2	0.52979
DCAF13	DDB1 And CUL4 Associated Factor 13	0.52810
FLRT1	Fibronectin Leucine Rich Transmembrane Protein 1	0.52569
ZFP37	ZFP37 Zinc Finger Protein	0.52569
ZNF862	Zinc Finger Protein 862	0.52569
IGBP1P1	Immunoglobulin (CD79A) Binding Protein 1 Pseudogene 1	0.52569
LGALS14	Galectin 14	0.52232
MFAP3	Microfibril Associated Protein 3	0.52095
SNORA49	Small Nucleolar RNA, H/ACA Box 49	0.52095
LINC00851	Long Intergenic Non-Protein Coding RNA 851	0.52095
IGHV3-11	Immunoglobulin Heavy Variable 3-11	0.52095
GTF2IP4	General Transcription Factor IIi Pseudogene 4	0.52095
SPDYE10P	Speedy/RINGO Cell Cycle Regulator Family Member E10, Pseudogene	0.52095
EIF4HP1	Eukaryotic Translation Initiation Factor 4H Pseudogene 1	0.52095
WBSCR2	Williams-Beuren Syndrome Chromosome Region 2	0.52095
CFM1	Cystic Fibrosis Modifier 1	0.52095
LOC108228208	7q11.23 Proximal Recombination Region	0.52095
LOC108228209	7q11.23 Distal Recombination Region	0.52095
LOC106029311	Williams-Beuren Syndrome Centromeric Block B Recombination Region	0.52095

LOC10602931	Williams-Beuren Syndrome Telomeric Block B Recombination Region	0.52095
B3GNT5	UDP-GlcNAc:BetaGal Beta-1,3-N-Acetylglucosaminyltransferase 5	0.51922
S100G	S100 Calcium Binding Protein G	0.51810
KIR2DL2	Killer Cell Immunoglobulin Like Receptor, Two Ig Domains And Long Cytoplasmic Tail 2	0.51391
KIR2DS2	Killer Cell Immunoglobulin Like Receptor, Two Ig Domains And Short Cytoplasmic Tail 2	0.51391
CPA6	Carboxypeptidase A6	0.50730
BEND3	BEN Domain Containing 3	0.50726
VGLL1	Vestigial Like Family Member 1	0.50571
SLCO5A1	Solute Carrier Organic Anion Transporter Family Member 5A1	0.50485
CLDN20	Claudin 20	0.49805
MIR4496	MicroRNA 4496	0.49736
CHRM4	Cholinergic Receptor Muscarinic 4	0.49223
MIR1323	MicroRNA 1323	0.48972
PANK3	Pantothenate Kinase 3	0.48543
GANC	Glucosidase Alpha, Neutral C	0.48543
RBPJL	Recombination Signal Binding Protein For Immunoglobulin Kappa J Region Like	0.48543
C1QTNF7	C1q And TNF Related 7	0.48543
ZBTB42	Zinc Finger And BTB Domain Containing 42	0.48543
TBC1D8B	TBC1 Domain Family Member 8B	0.48543
HEATR4	HEAT Repeat Containing 4	0.48543
CCDC144NL	CCDC144A N-Terminal Pseudogene	0.48543
TMEM187	Transmembrane Protein 187	0.48543
TMEM95	Transmembrane Protein 95	0.48543
SAP25	Sin3A Associated Protein 25	0.48543
CFAP46	Cilia And Flagella Associated Protein 46	0.48543
LINC00316	Long Intergenic Non-Protein Coding RNA 316	0.48543
SNORD96A	Small Nucleolar RNA, C/D Box 96A	0.48543
LRP4-AS1	LRP4 Antisense RNA 1	0.48543
LINC00942	Long Intergenic Non-Protein Coding RNA 942	0.48543
HLA-T	Major Histocompatibility Complex, Class I, T (Pseudogene)	0.48543
CHST2	Carbohydrate Sulfotransferase 2	0.48288
LOC11263702	ABO Upstream Enhancer	0.47527
ITLN2	Intelectin 2	0.47288
C1orf56	Chromosome 1 Open Reading Frame 56	0.46862
FKBP2	FKBP Prolyl Isomerase 2	0.46072
GID4	GID Complex Subunit 4 Homolog	0.46072

DLEU7	Deleted In Lymphocytic Leukemia 7	0.46072
SERPINE3	Serpin Family E Member 3	0.46072
SMCO3	Single-Pass Membrane Protein With Coiled-Coil Domains 3	0.46072
GPR119	G Protein-Coupled Receptor 119	0.45843
SLC44A5	Solute Carrier Family 44 Member 5	0.45736
GYPE	Glycophorin E (MNS Blood Group)	0.45486
DEXI	Dexi Homolog	0.45381
KLHL21	Kelch Like Family Member 21	0.45213
ANGEL2	Angel Homolog 2	0.45213
TSNAXIP1	Translin Associated Factor X Interacting Protein 1	0.45213
EFCAB12	EF-Hand Calcium Binding Domain 12	0.45213
TMEM131L	Transmembrane 131 Like	0.45213
PDZD3	PDZ Domain Containing 3	0.44989
CLEC4A	C-Type Lectin Domain Family 4 Member A	0.44560
SGPP2	Sphingosine-1-Phosphate Phosphatase 2	0.44486
PTTG3P	Pituitary Tumor-Transforming 3, Pseudogene	0.44486
GIMAP7	GTPase, IMAP Family Member 7	0.44262
CACFD1	Calcium Channel Flower Domain Containing 1	0.44262
ZCWPW1	Zinc Finger CW-Type And PWWP Domain Containing 1	0.44262
HSD11B1L	Hydroxysteroid 11-Beta Dehydrogenase 1 Like	0.44262
KANK3	KN Motif And Ankyrin Repeat Domains 3	0.44262
C1orf189	Chromosome 1 Open Reading Frame 189	0.44262
RSKR	Ribosomal Protein S6 Kinase Related	0.44262
SPINT1-AS1	SPINT1 Antisense RNA 1	0.44262
OR4C3	Olfactory Receptor Family 4 Subfamily C Member 3	0.43789
DHRS1	Dehydrogenase/Reductase 1	0.43184
NUDT3	Nudix Hydrolase 3	0.43184
CD300LG	CD300 Molecule Like Family Member G	0.43184
OXCT2	3-Oxoacid CoA-Transferase 2	0.43184
ISOC2	Isochorismatase Domain Containing 2	0.43184
LRR1	Leucine Rich Repeat Protein 1	0.43184
TRMT61A	TRNA Methyltransferase 61A	0.43184
SYCP2L	Synaptonemal Complex Protein 2 Like	0.43184
CCDC17	Coiled-Coil Domain Containing 17	0.43184
TMEM141	Transmembrane Protein 141	0.43184
LINC01599	Long Intergenic Non-Protein Coding RNA 1599	0.43184
CROCCP2	CROCC Pseudogene 2	0.43184

MIR3165	MicroRNA 3165	0.43184
MIR4640	MicroRNA 4640	0.43184
MIR4724	MicroRNA 4724	0.43184
MIR3529	MicroRNA 3529	0.43184
SLC26A5	Solute Carrier Family 26 Member 5	0.43066
KIR2DS5	Killer Cell Immunoglobulin Like Receptor, Two Ig Domains And Short Cytoplasmic Tail 5	0.42988
KLRG1	Killer Cell Lectin Like Receptor G1	0.42771
CNN3	Calponin 3	0.42674
MIR4728	MicroRNA 4728	0.42674
PDIA2	Protein Disulfide Isomerase Family A Member 2	0.42394
TRIM38	Tripartite Motif Containing 38	0.41904
GAL3ST3	Galactose-3-O-Sulfotransferase 3	0.41904
SLAMF8	SLAM Family Member 8	0.41904
CR1L	Complement C3b/C4b Receptor 1 Like	0.41904
ZNF329	Zinc Finger Protein 329	0.41904
DRAXIN	Dorsal Inhibitory Axon Guidance Protein	0.41904
PM20D2	Peptidase M20 Domain Containing 2	0.41904
ZNF320	Zinc Finger Protein 320	0.41904
LRRC61	Leucine Rich Repeat Containing 61	0.41904
ZSWIM3	Zinc Finger SWIM-Type Containing 3	0.41904
SLFNL1	Schlafen Like 1	0.41904
PPP1R14D	Protein Phosphatase 1 Regulatory Inhibitor Subunit 14D	0.41904
TMEM121	Transmembrane Protein 121	0.41904
THAP8	THAP Domain Containing 8	0.41904
AADACL4	Arylacetamide Deacetylase Like 4	0.41904
BRICD5	BRICHOS Domain Containing 5	0.41904
SYCE3	Synaptonemal Complex Central Element Protein 3	0.41904
ZBED6CL	ZBED6 C-Terminal Like	0.41904
C19orf73	Chromosome 19 Open Reading Frame 73	0.41904
NKAPD1	NKAP Domain Containing 1	0.41904
TMEM225B	Transmembrane Protein 225B	0.41904
PDPK2P	3-Phosphoinositide Dependent Protein Kinase 2, Pseudogene	0.41904
GIMAP3P	GTPase, IMAP Family Member 3 Pseudogene	0.41904
LEAP2	Liver Enriched Antimicrobial Peptide 2	0.41838
TAS2R46	Taste 2 Receptor Member 46	0.41667
DUS4L	Dihydrouridine Synthase 4 Like	0.41120
KIR3DS1	Killer Cell Immunoglobulin Like Receptor, Three Ig Domains And Short Cytoplasmic Tail 1	0.41120
TIGD1	Tigger Transposable Element Derived 1	0.40590

KCTD7	Potassium Channel Tetramerization Domain Containing 7	0.40434
H2AC9P	H2A Clustered Histone 9, Pseudogene	0.40429
KIR2DS3	Killer Cell Immunoglobulin Like Receptor, Two Ig Domains And Short Cytoplasmic Tail 3	0.40429
KIR2DL5A	Killer Cell Immunoglobulin Like Receptor, Two Ig Domains And Long Cytoplasmic Tail 5A	0.40429
GPR156	G Protein-Coupled Receptor 156	0.40236
DPEP3	Dipeptidase 3	0.40236
FAM89B	Family With Sequence Similarity 89 Member B	0.40236
TRMT10B	TRNA Methyltransferase 10B	0.40236
TMEM59L	Transmembrane Protein 59 Like	0.40236
WDR38	WD Repeat Domain 38	0.40236
RNF223	Ring Finger Protein 223	0.40236
CLPSL2	Colipase Like 2	0.40236
STIMATE-MU:	STIMATE-MUSTN1 Readthrough	0.40236
LINC00265	Long Intergenic Non-Protein Coding RNA 265	0.40236
ADAMTSL4-A	ADAMTSL4 Antisense RNA 1	0.40236
SNAI3-AS1	SNAI3 Antisense RNA 1	0.40236
IBA57-DT	IBA57 Divergent Transcript	0.40236
LOC10013110'	Putative UPF0607 Protein ENSP00000383783	0.40236
MIR5587	MicroRNA 5587	0.40236
GUCA1ANB	GUCA1A Neighbor	0.40236
ERHP1	ERH Pseudogene 1	0.40236
ENSG00000173366		0.40236
RPLP0P3	Ribosomal Protein Lateral Stalk Subunit P0 Pseudogene 3	0.40236
ENSG00000251184		0.40236
ENSG00000243696		0.40236
ENSG00000283977		0.40236
LOC11484103:	Uncharacterized Protein LOC114841035	0.40236
HIKESHI	Heat Shock Protein Nuclear Import Factor Hikeshi	0.39911
PRND	Prion Like Protein Doppel	0.39881
TSPAN17	Tetraspanin 17	0.38806
DNAJC5B	DnaJ Heat Shock Protein Family (Hsp40) Member C5 Beta	0.38806
MIR4310	MicroRNA 4310	0.37462
FAT3	FAT Atypical Cadherin 3	0.36979
RAB6C-AS1	RAB6C Antisense RNA 1	0.36726
COG8	Component Of Oligomeric Golgi Complex 8	0.35992
GNPDA1	Glucosamine-6-Phosphate Deaminase 1	0.35077

KIR3DP1	Killer Cell Immunoglobulin Like Receptor, Three Ig Domains Pseudogene 1	0.33115
MIR6754	MicroRNA 6754	0.33048
ZGLP1	Zinc Finger GATA Like Protein 1	0.31707
CDX4	Caudal Type Homeobox 4	0.30515
ANO4	Anoctamin 4	0.28000
BVES-AS1	BVES Antisense RNA 1	0.26469
C1QTNF9	C1q And TNF Related 9	0.25190
NAPEPLD	N-Acyl Phosphatidylethanolamine Phospholipase D	0.24605
KIR2DP1	Killer Cell Immunoglobulin Like Receptor, Two Ig Domains Pseudogene 1	0.20782
ZPBP	Zona Pellucida Binding Protein	0.19992
MIR4435-2	MicroRNA 4435-2	0.18182
SPINK6	Serine Peptidase Inhibitor Kazal Type 6	0.14136
LINC01149	Long Intergenic Non-Protein Coding RNA 1149	0.12857
ZNF253	Zinc Finger Protein 253	0.11189
LINC00842	Long Intergenic Non-Protein Coding RNA 842	0.11189
LOC11267919[illegible]	ABO +5.8 Intron 1 Enhancer	0.11189
